# Apical Localization of RNA Polymerases Modulate Transcription Dynamics and Supercoiling Domains Revealed by Cryo-ET

**DOI:** 10.64898/2026.03.25.714350

**Published:** 2026-03-26

**Authors:** Meng Zhang, Cristhian Cañari-Chumpitaz, Jianfang Liu, Bibiana Onoa, Sinead de Cleir, Enze Cheng, Katherinne I. Requejo, Carlos Bustamante

**Affiliations:** 1 California Institute for Quantitative Biosciences, University of California, Berkeley, USA; 2 The Molecular Foundry, Lawrence Berkeley National Laboratory, Berkeley, USA; 3 Department of Chemistry, University of California Berkeley, Berkeley, CA, USA; 4 Department of Biology, Stanford University, Stanford, CA USA; 5 Innovative Genomics Institute, University of California Berkeley, Berkeley, CA, USA; 6 Department of Molecular and Cell Biology, University of California, Berkeley, USA; 7 Howard Hughes Medical Institute, University of California, Berkeley, USA; 8 Department of Physics, University of California, Berkeley, USA; 9 Department of Chemistry, University of California, Berkeley, USA; 10 Molecular Biophysics and Integrative Bioimaging Division, Lawrence Berkeley National Laboratory, USA; 11 Kavli Energy Nanoscience Institute, University of California, Berkeley, USA; 12 Jason Choy laboratory of single molecule biophysics, University of California, Berkeley, USA

**Keywords:** DNA Supercoiling, Cryo-Electron Tomography, Transcription Dynamics, RNA Polymerase, Apical Binding Localization, dCas9, Torsional Block, Protein-DNA Interactions, Topoisomerase, Twin Supercoiling Domains

## Abstract

The canonical B-form DNA helix and its protein interactions are well-characterized, yet the behavior of torsionally constrained DNA, ubiquitous in cells, remains underexplored. Using cryo-electron tomography (cryo-ET), we 3D-reconstructed entire negatively supercoiled DNA substrates with bound RNA polymerase (RNAP), revealing DNA supercoiling conformational diversity and its interplay with molecular motors. RNAP and DNA-binding proteins like dCas9 preferentially localize at plectoneme apices during transcription, acting as torsional blocks. Together, dCas9 and RNAP on opposing plasmid apices can segregate “twin-supercoiling domains” without the need for external DNA end-tethering. The generation of twin domains reveals as regions of reduced supercoiling and the presence of multiple transcribing RNAP complexes. Negative supercoiling and apex localization of RNAP favor initiation but disfavor elongation, leading to slow-moving RNAP clusters. Topoisomerase I relieves RNAP pauses by removing them from apical constraints; the resulting RNAP load-and-release process from the apex provides a molecular mechanism for the "transcriptional bursting" phenomenon.

## Introduction

The influence of DNA supercoiling in both prokaryotic and eukaryotic DNA transactions(Baranello et al., 2012)—including transcription, replication, and chromatin segregation(Gagua et al., 1981; Yan et al., 2018), underscores the role played by deviations from canonical B-form DNA in cellular metabolism. In particular, prokaryotic genomes, inherently accumulate negative torsional stress with an average supercoiling density of ~ −0.06. Accordingly, the interplay between DNA supercoiling and the activities of nuclear proteins, such as the collective transcriptional behavior of RNAPs on supercoiled templates, or the torsional regulation by DNA gyrase and topoisomerase, remains an area of active research(Fu et al., 2024; Patel et al., 2023; Tripathi et al., 2022; Vayssieres et al., 2024; Vidmar et al., 2024).

The need to visualize DNA supercoiling dynamics has spurred the development of diverse biophysical approaches. In vitro single-molecule fluorescence microscopy enables real-time tracking of DNA plectoneme formation(Janissen et al., 2023). Atomic Force Microscopy (AFM) have further allowed the detection of kinks generated in supercoiled DNA(Pyne et al., 2021). However, these approaches typically require to partially confine the movement of DNA or immobilize it on a surface. Cryo-ET experiments have been used to revisit this topic using small minicircles in a more native state(Amzallag et al., 2006; Demurtas et al., 2009; Irobalieva et al., 2015), but the ~300 bp minicircles were not of sufficient length to capture the complexity of the plectoneme structures(Vologodskii and Cozzarelli, 1994). In structural studies, DNA transcription has primarily focused on elucidating RNA polymerase (RNAP) states and cofactor-mediated regulation on linear templates(Abdella et al., 2021; Aibara et al., 2021; Chen et al., 2021; Gnatt et al., 2001; Kettenberger et al., 2004), while the supercoiling aspect of the template is often overlooked. The "twin supercoiling domain model”(Liu and Wang, 1987), that describes overwinding (positive supercoils) ahead of the RNAP and transcription-induced DNA unwinding (negative supercoils) at its wake, still lacks detailed 3D structural characterization despite strong biochemical support(Chong et al., 2014; Janissen et al., 2023; Kim et al., 2019; Leng et al., 2004; Ma et al., 2013). Consequently, questions persist regarding: What are the structures naturally adopted by supercoiled DNA in 3D? what is the spatial relationship between a transcribing polymerase and its supercoiled substrate? And how do supercoiling and transcription affect mutually each other?

In this cryo-ET study, we achieved 3D reconstruction of individual plasmid particles (~2 kbp) under their naturally occurring degree of negative supercoiling, allowing precise quantification of their 3D conformational dynamics. With this plasmid population, we stalled and initiated RNAP to systematically investigate the mutual influence between supercoiling and transcription. We determined the orientation of RNAPs on their templates and discovered that their preferred apical binding on negatively supercoiled DNA plectonemes persists during active transcription, functionally facilitating RNAP initiation but hindering elongation. Interestingly, this apical binding also applies to dCas9, turning it into a “soft” torsional block that can hold up to three turns of DNA rotation (ΔLk = −3). In these conditions, the simultaneous apical binding of dCas9 and RNAP on opposite apices of a plasmid leads to the formation of segregate topological domains during RNAP transcription, inducing non-plectonemic DNA loops and promoting multi-RNAP slow co-transcriptional events. Additionally, introducing Topoisomerase I (TopI) in the presence of RNAP only partially releases the torsion within the plasmid, which, however, is sufficient to disrupt RNAP's apical positioning and increase transcription elongation activity. The 'on-off' switch function of apical constraint may play a critical role in linking torsional stress to transcription regulation.

## Results

### Individual particle 3D reconstruction of cellular-extracted DNA supercoiled plasmids

**Figure 1:**
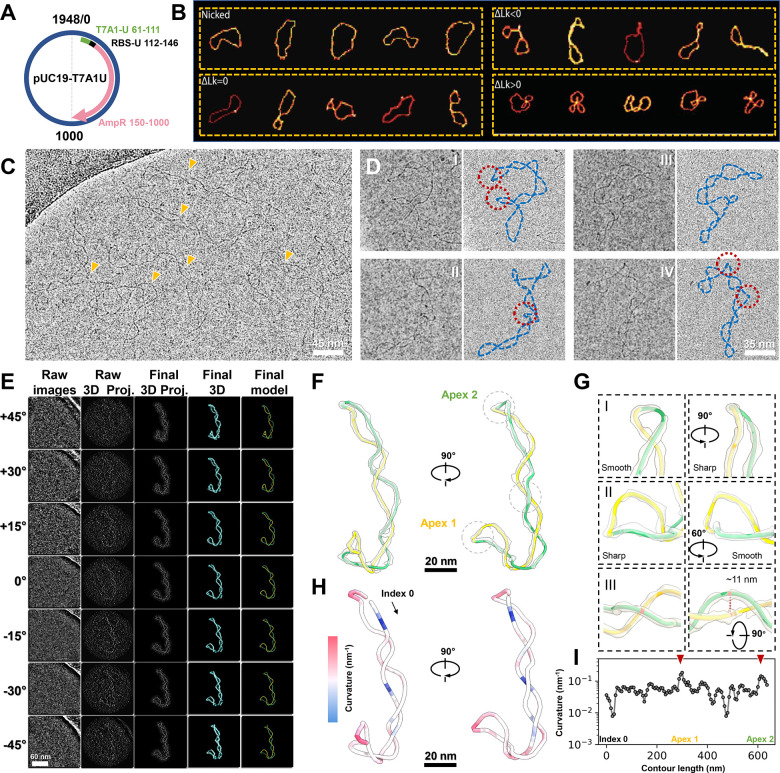
3D reconstruction of an individual negative-supercoiled (-sc.) DNA plasmid (**A**) The ~2 kb pUC19 plasmid construct features a T7A1U promoter (green), a U-less stalling site (black), and a transcriptional region (pink).(**B**) AFM images of representative pUC19 plasmid, illustrating various topological states, including nicked, neutral, −sc., and +sc. ΔLk variants. (**C-D**) Representative cryo-EM image and selected particles of – sc. pUC19 plasmid with ΔLk centered on −15, respectively. (**E**) Cryo-ET 3D reconstruction of a representative –sc. plasmid particle. Columns 1 through 5 illustrate the raw tilt series, raw 3D projection, masked 3D projection, final 3D density map, and fitted model, respectively, at corresponding tilting angles. (**F**) Zoom-in view of the final map and model reconstructed in E, displaying two intertwined DNA segments in yellow and green. (**G**) Different viewing perspectives affect the determination of DNA curvature (panels I and II) and spacing (panel III).(**H**) Color-coded map of the plasmid particle in F, highlighting large curvature region in red and small curvature in blue. (**I**) Tracing of the DNA curvature in H along the circular DNA plasmid.

To explore RNAP's interactions with its naturally occurring substrate, we first examined the structure and dynamics of a ~2 kbp modified pUC19 circular plasmid (Figure 1A) extracted and purified from stationary-phase *E. coli*. 2D gel analysis revealed that the two DNA strands of the isolated plasmid loop over each other 15 times fewer (ΔLk= −15) compared to their relaxed state (Lk=186), presenting a physiologically relevant supercoiling density (σ=ΔLk/Lk) of −0.08 (Figure S1A-B and S1D top). The size of pUC19 DNA molecules allows for the formation of multiple plectonemes, as observed by AFM, resulting in significant conformational deviation from nicked DNA and from positive supercoiled DNA (ΔLk >0) (Figure 1B). Although AFM offers adequate structural insights to distinguish between DNA populations from different origins, it is challenging to obtain the natural 3D structural diversity of DNA topology on surfaces. Therefore, we explored whether recent advancements in cryo-electron tomography (cryo-ET) could address these limitations by mapping the 3D topology of large plasmid molecules(Beel et al., 2021; Jentink et al., 2023; Zhang et al., 2024).

We initiated our cryo-EM imaging of plasmids under low salt (5 mM K^+^) to capture their loosely-coiled structures(Vologodskii and Cozzarelli, 1994) (Figure 1C), revealing predominantly long plectonemic conformations (Figure 1C, orange arrows). In certain regions, particles display "sharp kinks" (Figure 1D, red circles) similar to those captured by high resolution AFM(Pyne et al., 2021). Following a deep learning-based segmentation(Chen et al., 2019), local “per-particle” 3D reconstruction(Zhang et al., 2024), and IsoNet missing wedge correction(Liu et al., 2022) (Video S1 and Figure S2A), we were able to obtain the non-averaged 3D maps of individual plasmids (Figure 1E) at resolution of ~30–40 Å (Figure S1E). Using a DNA segment detection algorithm followed by manual curation (Figure S1F), it is possible to trace the complete 3D contour of each plasmid, enabling more accurate supercoiling topology quantification. For instance, for the molecule depicted in Figure 1E, it was possible to quantify the number of right-handed crossings (for negative supercoiling) between DNA helices in 3D space (Eastman et al., 2017), giving a value of Writhe (Wr) of −6.2. Such unambiguous identification would be difficult to obtain from 2D projections due to perspective effects (Figure S1G). For example, a smoothly curved DNA segment can exhibit a "sharp kink" in its 2D projection under specific views (Figure 1G, panels I), and, conversely, a sharp turn may be hidden in other orientations (Figure 1G, panels II). Therefore, we measured the 3D curvature (reciprocal of the radius of the osculating sphere at that point) along the DNA trajectory (Figure 1H) and the distance between the two intertwined DNA helixes in the plectonemic structure. (Figure 1G, panel III). The curvature distribution exhibits two peaks (Figure 1I, red arrows), corresponding to the two apices of the plectoneme (Figure 1G). These initial insights into plectonemic supercoiling characteristics laid the groundwork for subsequent study of the interplay between DNA supercoiling and other factors.

### Quantitation of DNA supercoiling 3D structural dynamics at various ionic strength

**Figure 2:**
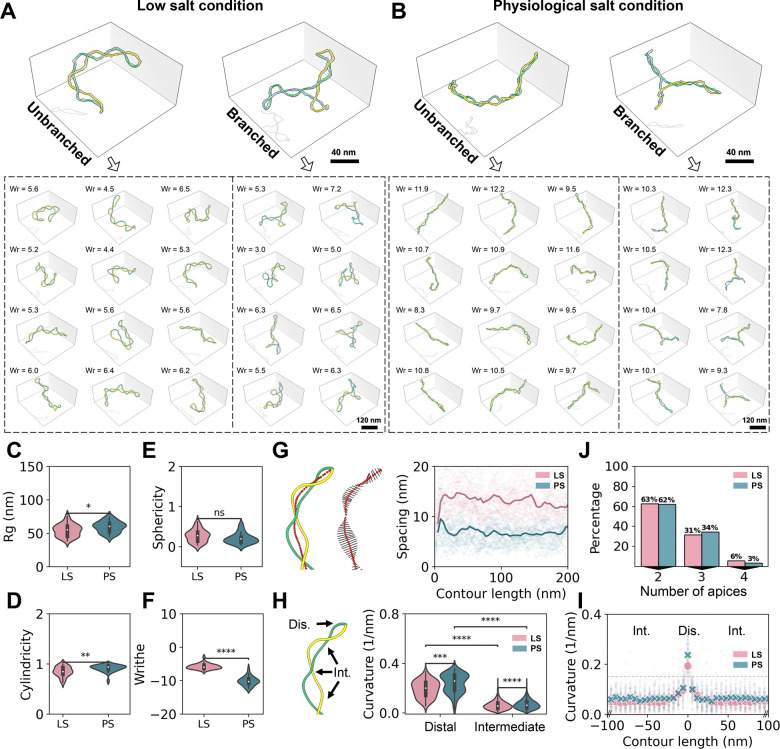
Structural analysis of –sc. plasmid under different salt conditions (**A-B**) Representative cryo-ET map and model of the –sc. plasmid (ΔLk centered on −15) under low salt (LS) and near physiological salt (PS) conditions, respectively. Two exemplary particles (unbranched and branch) are displayed on the top panel, with their collection presented on the bottom, featuring writhe numbers. (**C-F**) Statistical analysis of plasmid radius of gyration (Rg), cylindricity, sphericity, and writhe number using violin plots under LS (pink color) and PS (cyan color) conditions, respectively. (**G**) Schematic of plectoneme width quantification along the supercoiling axis, with the helical axis and spacing depicted as red dots and black bars (left panel). Tracing the averaged DNA spacing on LS and PS plasmids along the axis, starting from the apex (right panel). (**H**) Schematic indicating the distal and intermediate regions of a plectoneme (left panel), and statistics illustrating the change in DNA curvature under LS and PS conditions at the two regions. (**I**) DNA curvature distribution near the plectoneme apical region. Plasmids’ curvature were aligned and averaged at the apices (index zero). (**J**) Distribution of the number of apices of the plasmid in LS and PS conditions. Statistics are calculated using a Mann-Whitney test, where *p < 0.05, **p < 0.01, ***p < 0.001, ****p < 0.0001; and ns, not significant.

To investigate DNA supercoiling dynamics, we extended our analysis by collecting additional particles first in low salt (5 mM K^+^) (Figure 2A) and then in high salt condition (40 mM K^+^, 5 mM Mg^2+^) closer to physiological ionic strengths. (Figure 2B) (For all selected particles, see in Supplementary Particle Gallery). In higher salt, plasmids appeared more elongated, indicated by a larger radius of gyration (Rg) and increased cylindricity, while maintaining similar sphericity (Figure 2C-E, cylindricity and sphericity are values between 0 and 1, where their maximum indicate a thin rod and a perfect sphere, respectively). Additionally, the plasmids became more intertwined compared to their state in low salt (mean Wr shifted from −5.7 to −10.2, respectively Figure 2F), indicating that increased screening of the double helix phosphates favors the absorption of the negative supercoiling into writhe (Figure 2A-B, bottom) at the expense of changes (increase) in twist (ΔTw). These morphological changes are consistent with theoretical predictions(Vologodskii and Cozzarelli, 1994) in which enhanced electrostatic screening reduces intersegment repulsion and facilitates DNA crossing. Consistent with this interpretation, the average distance between the two intertwined DNA helices (plectoneme width) decreased from 13.2 to 8.4 nm (Figure 2G).

Altogether, high ionic strength increased the mean DNA curvature (Figure 2H), particularly at the distal ends (from 0.19 to 0.23 nm^−1^) compared to the intermediate region (from 0.06 to 0.07 nm^−1^). Applying a z-score of 2 (two standard deviations from the mean, Figure 2I, dashed line) to identify curvature outliers as potential kinks on the plasmid, we observed an average of 2 to 3 kinked regions per plasmid, with no significant difference between the two salt conditions. Notably, the apices of the plasmid under near-physiological salt conditions are consistently detected as kinks (Figure 2I). Because, some proteins can use indirect readout binding to specific DNA distortions that result from bulges, or lesions(Samee et al., 2019), we calculated the distribution of apex numbers for both conditions. Interestingly, the two ionic conditions yielded similar distributions, with ~60% of plasmids being unbranched (two apices, Figure 2J). Clearly, generation of new apices is energetically unfavorable.

### RNAP apical binding favors transcription initiation and affects the configuration of the supercoiled template.

**Figure 3:**
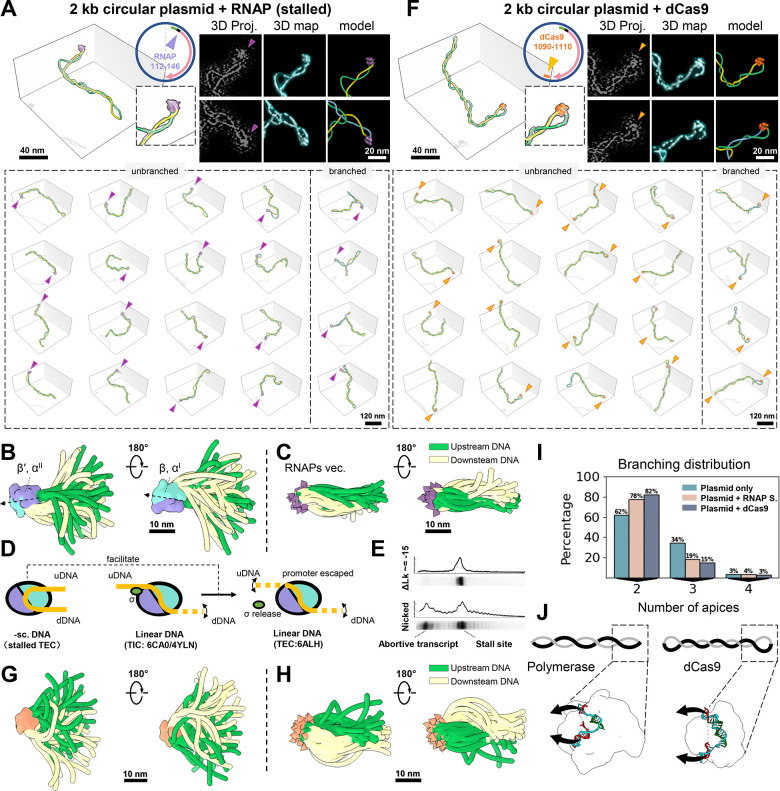
Conformational dynamics and regulatory effect of apically bound proteins on DNA supercoiling (**A**) Cryo-ET map and model of the –sc. plasmids bound with stalled RNAPs under PS condition. The top panel shows an exemplary particle (left) alongside zoomed-in views of the modeled RNAP stalling sites (right). The bottom panel features a collection of stalled TECs, with RNAP highlighted by purple arrowheads. (**B**) The superimposition of bound RNAPs reveal apical DNA dynamics. A dashed vector along the fissure composed of β′, α^II^ (purple) and β, α^I^ (cyan) subunits was used to depicted RNAP orientation. (**C**)The superimposition of the apical DNA segments shows the RNAP orientation dynamics (purple arrow heads). (**D**) Schematic highlighting different DNA geometries: TEC on –sc. DNA template (left), and TIC (middle) and TEC (right) on linear DNA templates. The dashed-orange line represents the flexible portion of the DNA. (**E**) An electrophoresis gel shows reduced abortive transcript levels on –sc. DNA compared to linear DNA toward the stalling site. (**F**) Control for A, with RNAP replaced by dCas9 (orange). (**G and H**) Superimposition of bound dCas9 and apical DNA, respectively, from all reconstructed particles. (**I**) Plasmid apex number distribution. (**J**) Schematic shows dCas9's smaller pocket curvature compared to that of RNAP, reshaping bound plasmids with enlarged distal ends and tightly intertwined body.

An earlier imaging study in the 1990s focusing on RNAP transcription on supercoiled DNA, unveiled an unexpected apical RNAP localization(ten Heggeler‐Bordier et al., 1992), an observation revisited recently by single-molecule experiments(Janissen et al., 2023). To verify and obtain structural insights into RNAP's apex binding localization, we incubated RNAP at 3:1 ratio to the pUC19 plasmid, which contains a single gene controlled by the strong T7A1 promoter (Figure 3A, top circle), in near-physiological salt. Stalled transcription elongation complexes (TECs) were obtained via UTP starvation, 20 nt downstream of the transcription start site (TSS). 3D reconstruction and modeling revealed that ~90% of RNAPs were located at the plectonemes' apices (Figure 3A). The majority (78%) of plasmids with an apically bound RNAP displayed an unbranched configuration. To determine the precise apical orientation of RNAP, we employed sub-tomogram averaging (STA) analysis using e2tomo(Chen et al., 2019). As a result of this analysis, the DNA entry and exit sites were blurred in the map that resulted from the average of all RNAP particles (both DNA bound and unbound) in the tomograms (14 Å resolution, Figure S3A, top). However, selectively using plasmid-bound RNAPs for STA (17 Å resolution) unveiled an additional DNA density protrusion (Figure S3A, bottom, black arrow), allowing the determination of the downstream DNA direction. This feature is notably absent in RNAP models (4YLN, 6CA0, 6JBQ, and 6N60) using linear DNA (Braffman et al., 2019; Fang et al., 2019; Narayanan et al., 2018; Zuo and Steitz, 2015), which only display a segment of upstream DNA (Figure S3B, orange color). Mapping STA-determined RNAP orientation onto their respective plasmid models (Figure 3A, top) allows the recovery of DNA information otherwise averaged out. Thus, superimposing all RNAPs at apical regions highlighted DNA's conformational dynamics (Figure 3B), while superimposing DNA at the same region revealed relatively restricted RNAP apical localization (Figure 3C).

In transcription, negative DNA supercoiling is believed to facilitate initiation by enhancing the kinetics of open complex formation through DNA unwinding(Ueshima et al., 1989; Wood and Lebowitz, 1984). However, its impact on other initiation events, such as promoter search and escape, remains unclear. Here, we find that the consensus DNA trajectory of the upstream DNA resulting from their superposition (Figure 3C) in apical stalled TECs (Figure 3D, left) significantly differs from that observed previously (4YLN and 6CA0 in Figure 3D, middle) in transcription initiation complexes (TIC) bound with a σ-factor to linear DNA (by ~110° bending, Figure S3C). Remarkably, we found that promoter escape was more efficient in supercoiled DNA, evidenced by a reduced rate of abortive initiation (Figure 3E). The significant change in the orientation of the upstream DNA in our complexes suggests that the apical localization on negatively supercoiled DNA helps disrupt the DNA-σ-factor interaction, facilitating promoter escape by RNAP (Kang et al., 2017) and its transition into elongation (6ALH) (Figure 3D, right).

Having studied the apical binding characteristics of RNAP, we wondered whether this feature is exclusive to RNAP or shared by other DNA-binding proteins. In the same imaging system, we substituted RNAP with catalytically inactive dCas9 accompanied by a sgRNA targeting our pUC19 plasmid's transcription termination site (TTS) (Figure 3F, top circle). We chose dCas9 for its RNAP-like DNA binding (i.e. forming a bubble via DNA-RNA hybrid) and its detectability (~10 nm) in cryo-ET. Interestingly, dCas9 also exhibits apical binding on negatively supercoiled plasmids (Figure 3F and Figure S3D). However, quantitative 3D analysis revealed differences between dCas9- and RNAP-plasmid complexes, with the former displaying more elongated, tightly wound plectonemes, as indicated by increased Rg and cylindricity, and decreased sphericity, with negative writhe increasing from −9.5 to −11.8 (Figure S4A-D). Notably, dCas9-bound apices exhibited smaller curvature than RNAP (from 0.25 to 0.22 nm^−1^) and greater DNA duplex spacing at the apex (from 7.7 to 10.3 nm) (Figure S4E-H), resulting in enlarged distal loops. Mapping dCas9 orientation highlighted these distinctions (Figure 3G-H), especially in entry-exit DNA orientation dynamics compared to those of RNAP (Figure 3B-C), with dCas9 binding leading to a slightly higher proportion (82%) of unbranched plectonemes than RNAP (78%) (Figure 3I). Why do two DNA-binding proteins with similar mechanisms affect plectoneme reshaping differently? By comparing RNAP(Finn et al., 2000) and dCas9(Josephs et al., 2015) binding pockets through their PDB structures, we found that despite its larger size (15 vs. 10 nm), RNAP has a more acute binding pocket with a sharper curvature than dCas9 (Figure 3J). Indeed, the corresponding pocket curvatures of dCas9 and RNAP match the DNA apex curvatures seen with their complexes (Figure 3J, boxes), suggesting that the resulting apex configuration significantly influences the overall supercoiling structure. These observations also indicate that apex binding behavior is not exclusive to RNAP but is a structural attribute of DNA supercoiling accommodating various shapes of binding proteins which, in turn, affect differently the overall configuration of the supercoiled template.

### Transcription induces branched supercoiling while preserving RNAP's apical localization

RNAP's translocation along the DNA helix necessitates rotational movement by either the DNA or RNAP to keep the DNA template in register within the RNAP active site. Simultaneous rotational constraints on both RNAP and DNA, such as RNAP tethering to cellular structures(Brogna et al., 2002; Liu et al., 2018) and DNA confinement within dense genome architecture(Gilbert and Allan, 2014; Luijsterburg et al., 2008b; Worcel et al., 1981), can lead to the formation of twin supercoiling domains (Liu and Wang, 1987). However, such external constraints might not always be present in various cellular contexts. It is therefore intriguing to investigate whether intrinsic constraints in TECs resulting from RNAP’s apical localization and a negative supercoiling background(Ljungman and Hanawalt, 1995; Luijsterburg et al., 2008a; Matsumoto and Hirose, 2004) can sustain transcription and contribute to topological-twin-domain delineation.

**Figure 4:**
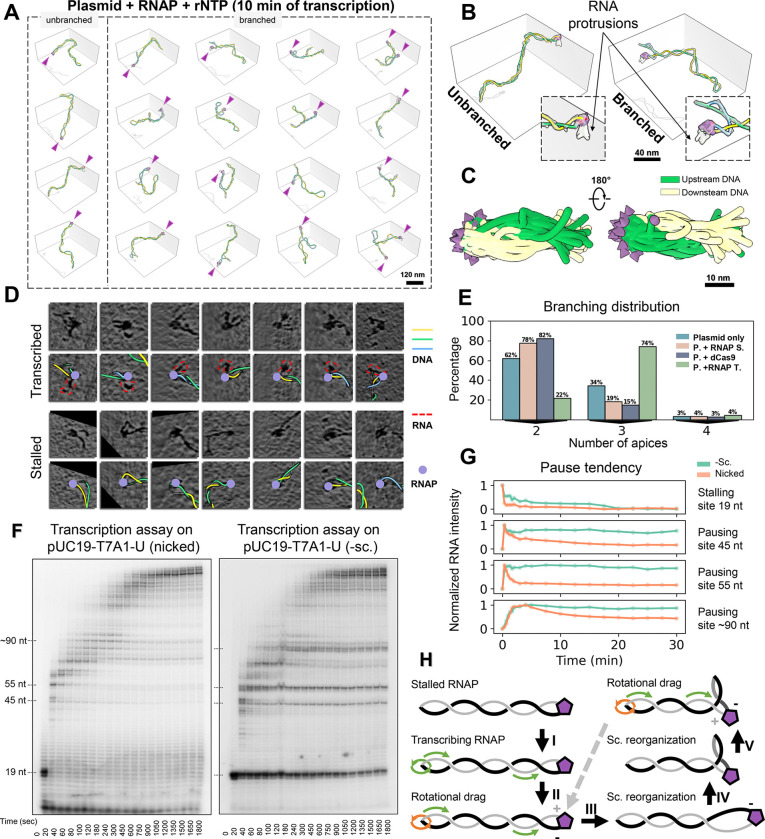
DNA Supercoiling morphology altered by non-equilibrium transcription (**A**) Cryo-ET 3D map and model of –sc. plasmid in the presence of RNAP (purple) after 10 min of transcription under PS conditions. (**B**) Zoom-in image of two representative apically bound RNAPs, with nascent RNA protrusion densities highlighted by black arrows. (**C**) The superimposition of apical DNA segments. (**D**) Z-dimensional slice (10 nm thickness) of sub-tomograms showing plasmid bound RNAPs after (top) and before (bottom) adding full set of NTPs. Each slice are superimposed with its fitted plasmid model. RNA density is marked by red dashed circles. (**E**) Plasmid apex number distribution. (**F-G**) Electrophoresis analysis of RNAP transcription on nicked and –sc. templates over time, with quantification of their pause release dynamics, respectively. (**H**) Schematic illustration of transcription-induced new plectoneme formation on –sc. plasmid. RNAPs are represented as purple pentagons. DNA translational and rotational motion is indicated by green arrows, with orange color denoting drags. Plus and minus signs indicate positive and negative DNA torsion, respectively.

To investigate the above scenario, we added the full set of NTPs (100 μM) to the stalled TECs and imaged the sample 10 minutes after resuming transcription. Remarkably, the 3D reconstruction reveals the continued apical positioning of RNAP in transcribing complexes (Figure 4A-C), with nascent RNA density intermittently observed around RNAP in both 3D maps (Figure 4B) and 2D slices (Figure 4D top, red contours), but absent in stalled RNAP (Figure 4D bottom). However, the limited resolution of cryo-ET, along with conformational heterogeneity of RNA(Ding et al., 2023) prevented the tracing of complete RNA transcripts, permitting only the mapping of the RNA protrusion direction relative to RNAP (Figure S4I). Quantitative analysis indicated that, in this non-equilibrium state, the plasmid predominantly appeared in a multi-plectoneme form for the first time (over 74% of the structures), in contrast with the effects of ionic strength and static protein binding (Figure 4E). As a result, there was a decrease in average plasmid Rg and cylindricity (Figure S4J-K). Thus, intrinsic negative supercoiling constraints, together with an active translocation of the DNA relative to an RNAP, is sufficient to promote the formation of new domains. However, the writhe between RNAP-transcribed and stalled plasmids was similar (−9.5 vs. −9.8) (Figure S4M), and there is no significant difference in Wr density (Wr/branch length) across all branches (−0.134 vs. −0.129 nm^−1^, Figure S4N). This observation suggests that persistent twin supercoiling domains are not generated during active, non-equilibrium propagation of torsion, which instead promotes the generation of new domains while conserving the negative writhe.

DNA torsional stress resulting from transcription of torsionally constrained templates is known to impede transcription elongation, causing RNAP stalling and increased backtracking as shown in single-molecule assays(Ma et al., 2013). Our results above suggest that RNAP’s apical positioning can instead favor transcription initiation. Given the persistent apical localization of RNAP observed in our experiments during active transcription elongation, we wondered if transcription rate was also affected in these conditions. Using radiolabeled α-^32^P-ATP, single-round in vitro transcription assays on negatively supercoiled DNA displayed significantly slower elongation kinetics (Figure 4F), as well as increased pause frequency and duration (Figure 4G), than those performed on nicked DNA. We note that, in our bulk transcriptional assay, the topological constraint arises from the natural steady-state degree of negative supercoiling of the plasmids extracted from the cell. The slower elongation kinetics supports the notion that the apical localization of RNAP hinders its rotation around DNA, and that the inefficient rotation of the larger DNA molecule becomes rate-limiting to transcription (ten Heggeler‐Bordier et al., 1992). Building on these findings, we propose a model for how transcription takes place in intrinsically negatively supercoiled DNA (Figure 4H): (I) The persistent apical positioning of RNAP requires DNA to rotate during transcription as it is thread through the enzyme. (II) However, the propagation of DNA rotation throughout the entire plasmid structure is slow (Nelson, 1999; Thomen et al., 2002), so that transcription induces transient (+) torsional stress ahead of the enzyme and (−) torsional stress at its wake. (III) In topologically constrained DNA, the (+) torsional stress is rapidly absorbed by the negatively supercoiled background, and therefore, diffusion of (+) and (−) supercoils through the molecule to cancel each other at the other end of the plasmid does not take place. (IV) Meanwhile, since high salt conditions favor writhe over twist in ΔLk distribution (Figure 2F), the extra (−) torsional stress generated at the wake of the polymerase promotes the formation of a new negative plectoneme branch. (V) This dynamic may promote continuous growth of the nascent plectoneme, evidenced by the observation of various plectoneme branch lengths (from 5 to 234 nm) centered on 70 nm (Figure S4O).

### Dual apical protein binding promotes the formation of topological domains

The results above have highlighted RNAP's role in generating torsional stress during non-equilibrium transcription of supercoiled DNA. Torsional stress during transcription can also accumulate due to the presence of torsional roadblocks, such as DNA-binding proteins and transcription factors(Chatterjee et al., 2021; Horberg and Reymer, 2020), leading to the formation of topological domains that can affect RNAP molecules distant kilobases away(Kouzine et al., 2013). Nonetheless, the mechanism through which small DNA-binding proteins impose rotational constraints and favor the generation of topological domains remains elusive.

To gain insight into this process, we employed dCas9 again but in this case for its capability of delaying DNA rotation, as reported in magnetic tweezers assays(Aldag et al., 2021). To confirm that dCas9 can serve as a programmable torsional roadblock, we performed DNA relaxation experiments using topoisomerase I (TopI) in the presence and absence of dCas9 bound to the TTS (Figure 3F, top circle). The relaxation activity of TopI involves rotation of the DNA helix until an equilibrium state (ΔLk=0) is achieved.

Our results demonstrate that dCas9 delays the relaxation kinetics of pUC19 plasmids, as topoisomers (with ΔLk from −3 to −1) still persist after a 30-minute reaction (Figure S5A). Thus, dCas9 impedes free DNA rotation in the low-torsion regime, acting as a “soft” torsional block. To investigate this mechanism, we performed coarse-grained molecular dynamics simulations using oxDNA(Sulc et al., 2012) to model the relaxation dynamics. Specifically, we tracked the trajectory towards relaxed state of equilibrated negatively supercoiled DNA template by placing a single nick at different locations along it. Results reveal that imposing an apical constraint on one of the two apices apparently slows supercoiling torsional relaxation dynamic when the nick is positioned between them (Video S2 and Figure S5B, Model I, II, and III). However, when the nick is placed directly on the apex, relaxation becomes slightly faster, even with the other apex being constrained. (Figure S5B, Model IV). As Model IV is the only scenario where torsional propagation encounters no apices (with the apex positioned at the very end), it suggests that the apex, particularly the confined apex (mimicking dCas9 binding), hinders free DNA rotation.

**Figure 5:**
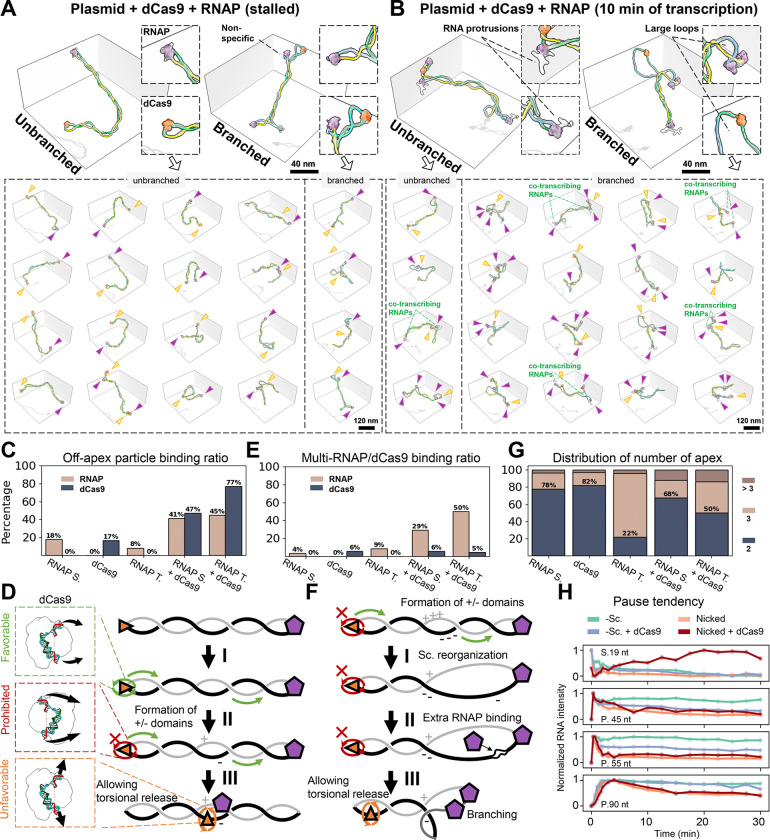
Plectoneme apical constraint induces multi-RNAP transcription on –sc. template (**A**) Cryo-ET 3D maps and models showing –sc. plasmids simultaneously bound with stalled RNAP (purple) and dCas9 (orange) under PS condition. Representative unbranched and branched particles displayed in the top panel, with their respective particle collections presented in the bottom panel. (**B**) RNAP release from stalling in A observed after 10 minutes of transcription. (**C**) Comparison of off-apex particle ratios for RNAP and dCas9. (**D**) Schematic displaying three apical configurations of dCas9 (left) and illustrating the accumulation and release of torsion in a –sc. plasmid due to simultaneous bindings of dCas9 (orange triangles) and RNAP (purple pentagons) on apices (right). (**E**) Quantification of multi-RNAP and dCas9 binding plasmids ratio within the population. (**F**) Schematics depicting the active RNAP transcription induced topological domains and the supercoiling rearrangement in the presence of torsional block of dCas9. (**G**) Quantification of plasmids‘ apex number. (**H**) Assessment of RNAP pause release in the presence of dCas9 during transcription on nicked and –sc. templates over time via electrophoresis. The RNA band intensities were quantified at the stalling site and three strong pausing sites.

Next, transcription was initiated on pUC19 plasmids harboring a dCas9 bound to the same TTS of the plasmid (Figure 3F, top circle). In the presence of dCas9, we first imaged the stalled TECs with 20 nt transcribed (Figure 5A). Results show that RNAP and dCas9 mainly localize to opposite ends of a DNA plectoneme (Figure 5A, top and bottom, columns 1–3), in agreement with their respective individual binding conformations (Figure 3A and 3F). However, we also observed a significant increase in non-apical binding cases (Figure 5A, bottom panel, columns 4–5 and Figure S6A) for both RNAP and dCas9 (from 18% to 41% and from 17% to 47%, respectively, Figure 5C). These observations suggest that accumulation of (+) torsional stress between the front of RNAP and dCas9 and (−) stress between dCas9 and the back of RNAP resulting from transcription induces partial escape of the proteins from the apices, as the system tries to minimize its energy. Figure 5D summarizes these results: (I) Apical RNAP transcription requires DNA rotation and, when present, dCas9 rotation. (II) However, dCas9 rotation at the apex is restricted, which is reflected in the fact that we never observed reversed dCas9 apical binding conformations. (III) The release of dCas9 from the apex to the plectoneme's intermediate region permits DNA rotation again, allowing the (+) and (−) domains generated in front and in the wake of the enzyme to cancel each other. Lastly, we note that because the torsional block function of dCas9 results from its apical location, it could apply to other nucleoproteins (Leng and McMacken, 2002).

### Torsional block enhances cooperative RNAP transcription

As shown above, (+) and (−) torsional stresses differentially accumulate in each half of the plasmid delineated by RNAP and dCas9, leading to the partial displacement of the proteins from their apical positions. Accordingly, we anticipate that dCas9 will be found more often off-apex after 10 minutes of active RNAP transcription (Figure 5B). Indeed, the off-apex ratio of RNAP and dCas9 both increased after this period of transcription (Figure S6B), with dCas9 reaching up to 77% (Figure 5C). Given a negative supercoiling background (ΔLk = −15), the “twin-domain” model should result in the domain in front of the polymerase to be less negatively supercoiled and the domain at its back becoming hyper-negatively supercoiled. Evidence supporting the creation of torsionally imbalanced domains is as follows: (1) We observe the presence of floppy (non-plectonemic) DNA loops among the actively transcribing complexes (Figure 5B), a feature rarely seen in stalled TEC/dCas9 systems (Figure 5A), supporting the generation of less negatively supercoiled domains (Figure 5F, panel I). Quantitative analysis confirmed this observation: although the overall morphology of the plasmids remained similar (Figure S6C-E), the transcribing dCas9-TEC complexes displayed plectonemic branches with low Wr density (Figure S6K, red arrow) resulting in a decrease in the mean Wr (from −10.9 to −9.8, p < 0.05) (Figure S6F), the emergence of smaller apex curvature (from 2.5 to 2.3 nm^−1^, p < 0.05), and larger DNA spacing apexes (from 8.5 to 11.1 nm, p < 0.001, Figure S6G-J). (2) Although the expected hyper-negative plectonemes in transcribing dCas9-TEC complexes are not evident from the analysis (Figure S6K), a significant increase in the binding of multiple RNAPs (from 20% to 50%, Figure 5B and 5E) observed in these conditions probably reflects enhanced negative supercoiling: since RNAP binding unwinds ~10 bp of the DNA helix, the resultant extra negative supercoiling could be absorbed by additional regions of melted DNA to which additional RNAP can bind (Figure 5F, II). Moreover, upon transcription, the proportion of branched-form plasmid increased from 32% in the absence of dCas9 to 50% in its presence (Figure 5G), offering an alternative mechanism to absorb negative supercoils (Figure 5F, III). A similar trend is also observed in coarse-grained simulations where we mimic the torsional stress produced by RNAP by using a rotating harmonic trap. The MD simulation compares the differences between a free apex and a constrained apex in managing (+) and (−) torsion on either side (Video S3). Only the constrained apex successfully replicates the large loop and branching phenomena observed experimentally.

Leveraging dCas9's gRNA-directed DNA binding, we tentatively utilized it as a fiducial marker to map the position and orientation of RNAPs on our plasmid construct (Figure S6L). Some RNAPs were located outside the transcriptional region (brown arrows) or oriented in the upstream direction (blue arrows) both in the transcribing and the non-transcribing region. We interpret them as reflecting non-specifically bound RNAPs whose binding is enhanced by the additional negative torsional stress resulting from transcription. Conversely, most RNAPs were positioned within the transcriptional region, oriented downstream (pink arrows), revealing in some cases more than one transcribing RNAPs. Direct observation of two RNAPs on the same template simultaneously displaying nascent RNA protrusions (Figure 5B, green labels) supports this interpretation. These findings suggest that the formation of imbalanced topological domains by the presence of dCas9 in a negatively supercoiled template induces cooperative RNAP transcription, potentially enhancing transcriptional kinetics.

To corroborate this hypothesis, we performed a bulk transcription assay in the presence of dCas9 using radioactively labeled α-^32^P-ATP (Figure S7A-B). Our results indicate that, during transcription on the negatively supercoiled template, while pause frequency remains similar, pause escape is faster in the presence of dCas9, particularly at the 45 nt and 55 nt pause sites (Figure 5H, second and third panel, blue curve). The observation that pause escape is more efficient with the dCas9 torsional block suggests that the accumulation of (+) torsion in front of RNAP alleviates its apical constraint on negatively-supercoiled template, facilitating transcription elongation, although not to the level of nicked templates (Figure 5H, all panels, orange curve). In the case of transcription in the presence of both dCas9 and nicked DNA (with the nick site located downstream of RNAP), transcription resembles that of nicked DNA alone, except for an increase in pause duration at the initial stall site (Figure 5H, top panel, red curve). This effect probably reflects transcription re-initiation events. Accordingly, in the presence of the nick, RNAP leaves the promoter more quickly when facing a relax template in front, while accumulating negative supercoiling behind it with dCas9 that facilitates the initiation of a new RNAP, both effects ultimately combining to increase the amount of transcript. Together, these results suggest that negative supercoiling domains favor transcription initiation, while relaxed DNA (ΔLk=0) favors transcription elongation.

### TopI removes the apical constraint of RNAP facilitating DNA transcription

**Figure 6:**
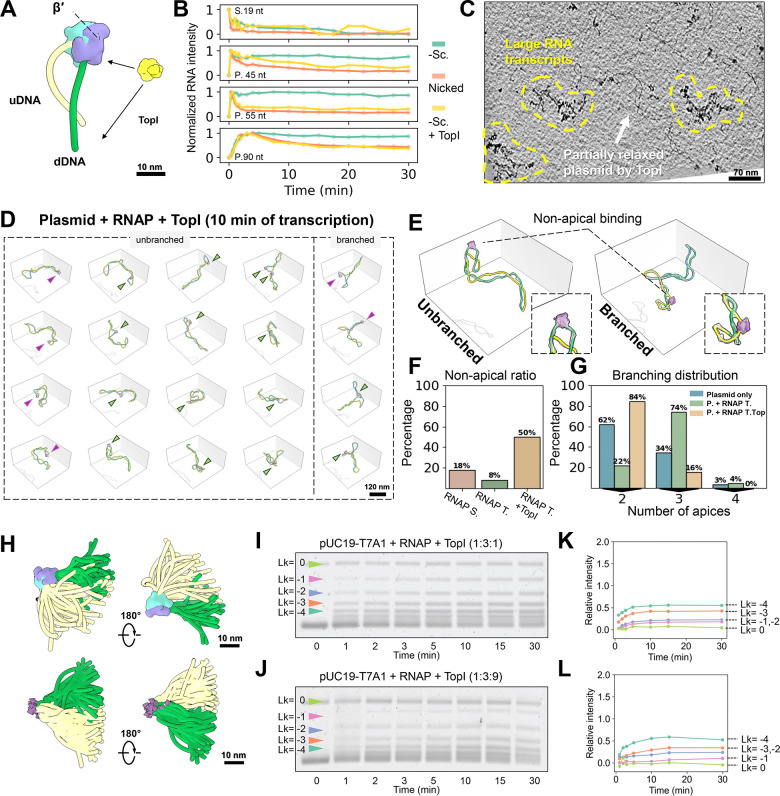
Topoisomerase I releases RNAP from apical constraint during transcription (**A**) Schematic representation of TopI binding to RNAP β′ subunit or DNA. (**B**) Assessment of RNAP pause release in the presence of TopI via electrophoresis. (**C**) Cryo-ET image of a z-dimensional slab (50 nm thickness) of the sample after 10 minutes of transcription of the pUC19-T7A1 in the presence of TopI. (**D**) Cryo-ET 3D reconstructions of the above plasmid in the presence of RNAP and TopI after 10 min of transcription. The left and right panels display unbranched vs. branched particles, respectively. Apical and non-apical binding RNAP are distinguished with purple and green arrowheads, respectively. (**E**) Zoom-in image of two representative plasmid-bound RNAPs that escape from the apical location of the plasmid. (**F-G**) Quantification of the plasmid’s apex number and non-apical RNAP ratio, respectively. (**H**) The superimposition of plasmid-bound RNAPs (top panel) and RNAP’s upstream and downstream DNA segments (bottom panel). (**I-J**) DNA supercoiling relaxation assay in the presence of RNAP and TopI at ratios of 3:1 and 1:3, respectively. (**K-L**) Quantification of DNA Linking number in I and J, respectively.

Although the presence of a torsional roadblock like dCas9 promotes cooperative RNAP transcription, it could not fully replicate the transcriptional dynamics seen on nicked circular plasmids. Accordingly, we sought to determine if TopI, known to interact with both DNA and the β’ subunit of RNAP in complex(Cheng et al., 2003; Sutormin et al., 2022) (Figure 6A) could alleviate the apical constraints on RNAP by relaxing the DNA plectoneme and thereby relieve RNAP from its pauses.

We first performed bulk studies to explore the impact of TopI DNA relaxation on RNAP. Bulk transcription assays on negatively supercoiled plasmid with TopI confirmed improved transcription (Figure 6B, yellow curve, and Figure S7C), exhibiting kinetics similar to those of nicked templates without torsional stress (Figure 6B, orange curve). Based on these results, we conducted a cryo-ET imaging study of RNAP transcription in the presence of TopI (RNAP:TopI:plasmid=3:1:1). TECs, after 10 minutes of transcription post-release from stalling, revealed a significant presence of large RNA transcripts (Figure 6C). In the cryo-ET sample, TopI activity alone is sufficient to displace 50% of RNAPs from their apical localization, even in the absence of a torsional block (Figure 6D, green arrows, and Figure 6E), yielding the highest off-apex ratio across all tested conditions (Figure 6F). Quantitative analysis further revealed significant structural differences between the apices of TopI-relaxed and unrelaxed plasmids, with a marked reduction in curvature (from 0.25 to 0.18 nm^−1^, p < 0.0001) and an increase in spacing (from 7.3 to 15.2 nm, p < 0.0001) (Figure S7H-K). We attribute this prominent apex structural change to the relaxation of the plectoneme, which exhibited a mean writhe (Wr) of −6.4 (Figure S7G), approaching the Wr level of −6.1 seen in low salt conditions (Figure 2A). Dynamic analysis of all TECs orientations on TopI-induced partially relaxed plasmids reveals highly dynamic upstream and downstream DNA arms (Figure 6H), in contrast to RNAP localized at the apex (Figure 4C).

Interestingly, while TopI facilitates RNAP transcription, RNAP also modulates TopI activity. In bulk, in the absence of RNAP, TopI at a 1:1 ratio to the plasmid demonstrated rapid relaxation kinetics (ΔLk from −15 to −1 and 0) within minutes (Figure S5A, left). Introducing RNAP (plasmid:RNAP:TopI =1:3:1) significantly slowed the relaxation kinetics (Figure 6I). The 1D gel analysis showed topoisomer bands clustering at ΔLk < −4 even after 30 minutes (Figure 6K). 2D gel electrophoresis further resolved these bands, revealing an accumulation of topoisomers at ΔLk = −6 to −9 (Figure S1C-D). Further experiments showed that the RNAP effect was not TopI dose-dependent, as similar relaxation kinetics were observed even with excess TopI (plasmid:RNAP:TopI =1:3:9) (Figure 6J and 6L). These results are similar to those observed in dCas9-mediated downregulation of TopI in bulk assays (Figure S5A, right). However, the torsional block effect of RNAP appears to be more pronounced than that of dCas9 (dCas9-topoisomers clustered at ΔLk = −1 to −3), likely due to its ability to induce sharper kinks in DNA and its interaction with TopI. Overall, these results suggest that alleviating torsional stress during transcription significantly facilitates RNAP progression by “turning off” its apical constraints.

## Discussion

Advanced structural methods have successfully captured the conformations of both proteins and nucleic acids at atomic resolution by focusing on stable, highly populated states that often correspond to equilibrium conformations. In particular, studies of nucleoprotein complexes have been primarily aimed at understanding how proteins locally affect DNA; the broader effects of DNA-binding proteins on the overall DNA conformation have often been overlooked. Here we have shown that cryo-ET coupled with current image processing techniques, make it possible to describe the global 3D architectures of highly dynamic DNA/protein complexes heretofore significantly underexplored. By 3D reconstruction of the entire DNA backbone of cell-extracted plasmids harboring natural negative supercoiling densities, we precisely quantify the conformational dynamics of plectonemes. This capability has allowed us to study how molecular motors, such as RNAP, influence the 3D arrangement of DNA through the torsional stress induced by transcription. Conversely, we determine how the torsional stress arising from the resulting DNA topology affects RNAP activity. We find that transcribing and non-transcribing elongation complexes tend to localize at the apices of plectonemes; this localization hinders their rotation during active transcription and favors the adoption of non-equilibrium structures displaying additional plectonemes.

We provide a structural insight into RNAP’s plectoneme apex binding configuration(Janissen et al., 2023; ten Heggeler‐Bordier et al., 1992). The apex localization is not unique to RNAP, but is also observed for other proteins capable of opening DNA duplexes, such as dCas9. The pocket site of DNA binding proteins restructures the apex curvature of DNA plectonemes, imposing a global conformational change on the DNA, reflected in the generation of different writhe numbers. Our results suggest that DNA supercoiling can function as a platform that facilitates not only the accommodation of different binding proteins but also enables communication between them through modifications in DNA topology. Importantly, we find that dCas9 or stalled-RNAP bound at the apex operate as torsional blocks preventing the rotation of both DNA and these proteins. During transcription, RNAP allows DNA rotation and, as a result, the cumbersome rotation of large DNA molecules becomes rate-limiting. Additionally, in the presence of dCas9 or stalled RNAP, their apical localization retains up to 3–9 turns of DNA (ΔLk = −3 to −9) during TopI relaxation experiments. The interaction of enzymes such as dCas9 with actively transcribing supercoiled DNA serves as a valuable proxy for studying torsion-related regulation by transcriptional factors (TFs).

According to the twin domain model, transcribing RNAP induces extra DNA overwinding ahead of itself and underwinding at its wake, effectively segregating the DNA template into isolated topological domains. However, we note that the development of topological domains arising from transcription in the presence of dCas9 has additional consequences. For instance, in our system, involving the negative supercoiling density present in the cell, DNA underwinding can sometimes appear in the form of melted DNA, allowing recruiting of several RNAP molecules to the plasmid, while DNA overwinding is absorbed by the negative supercoiling background (ΔLk = −15), resulting in the formation of large non-plectonemic DNA loops. These observations reflect a more physiologically relevant scenario, where DNA is kept at a cellular level of negative-supercoiling while being free of surface fixation or tethering.

We show that negative supercoiling plays an important regulatory role in transcription: it assists promoter escape by reducing the extent of abortive initiation (Figure 3E), and the formation of hyper-negative supercoiling domain increases the binding of RNAPs on both transcriptional and non-transcriptional regions (Figure S6L). We hypothesize that the resulting increase in RNAP binding could expedite promoter search, and the establishment of transcriptional cascades, as we only observe co-transcribing RNAPs (with RNA protrusions) after the formation of topological domains (Figure 5B). On the other hand, negative supercoiling can also impede transcription, as evidenced by the slower scape from pauses by RNAPs during their elongation phase (Figure S1C), a conclusion also raised by single-molecule study (Ma et al., 2013). However, unlike single-molecule studies that focus on the accumulation of negative supercoiling in RNAP-DNA end-constrained systems, our work emphasizes conditions where RNAP and DNA are free, favoring the formation of DNA plectonemes that constrain RNAP to the apex and reduce transcriptional dynamics. Nevertheless, this negative supercoiling (or plectoneme)-induced RNAP pausing can be rescued by TopI, which restores elongation to a level comparable to that of transcription on nicked DNA. Interestingly, we observed that during these rescue experiments, TopI activity is downregulated, preventing full DNA relaxation. This downregulation allows the system to recuperate a level of negative supercoiling density close to that present in the non-transcriptional state, reducing its need of gyrase activity and, thus, conserving energy.

**Figure 7:**
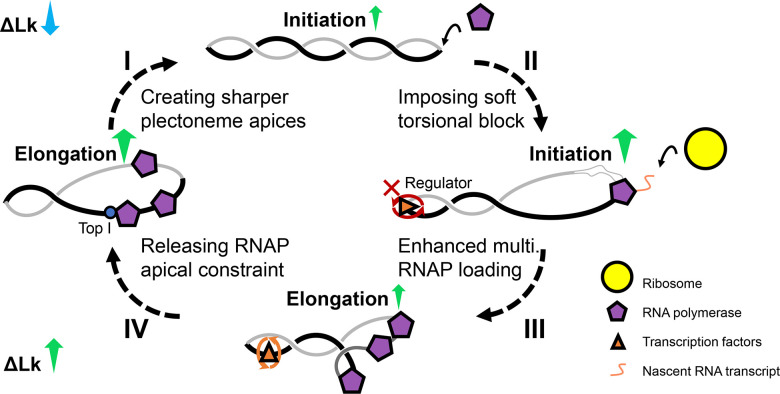
Mechanistic model of supercoiling modulation in transcriptional bursting cycles Schematic diagram illustrating the transcription cycle modulated by supercoiling and apical binding: (I) Enhancement of –sc. in the plasmid by enzymes like DNA gyrase imposes apical constraints on RNAP, facilitating transcription initiation. (II) Apically initiated RNAP facilitates spatial RNA-DNA separation, while apically bound regulatory proteins promote topological domains during transcription. (III) Hyper-negative supercoiling domain prompts multi-RNAP loading. Removal of torsional blocks prevents unbound torsional buildup during transcription. (IV) Activation of enzyme, such as TopI, partially releases negative torsion in the plasmid, sufficient to dislodge RNAP from apices and facilitate transcription elongation. The green and blue arrows indicate upregulation and downregulation, respectively.

The opposite effect of negative supercoiling on RNAP transcription—favoring initiation but hindering elongation—supports a scenario of transcriptional pulses, reminiscent of the “transcriptional bursting” phenomenon observed in vivo(Golding et al., 2005; So et al., 2011). Single-molecule fluorescence assays have demonstrated a link between DNA supercoiling and transcriptional bursting(Chong et al., 2014): The burst cycle begins with transcription on relaxed DNA (on-state), with the accumulation of positive torsional stress limiting activity (off-state) and requiring rescue by DNA gyrase. Here, we propose an alternative model in which a negatively supercoiled template serves as the basal state, with RNAP apically constrained (off-state) and TopI acting to restore transcription activity (on-state). Specifically, at the onset of the cycle (Figure 7, I), DNA stores significant negative torsion, forming sharp apices that favor RNAP localization and slow elongation while generating additional plectonemes. This apical RNAP geometry promotes segregation of nascent RNA from the DNA body, facilitating RNA processing, such as ribosome binding. Subsequently (Figure 7, II), transcription factors (TFs) bind remaining apices as regulatory torsional blocks, creating topological domains that aid multi-RNAP initiation at the promoter. During slow transcription by apex-bound RNAPs (Figure 7, III), these torsional blocks can disengage, preventing unbound torsion accumulation. Finally (Figure 7, IV), DNA topoisomerases rescue the slow-moving RNAPs by partially relieving negative supercoils, triggering a transcriptional cascade. The requirement for topoisomerase for active transcription is supported by the direct interaction between *E. coli* TopI and RNAP(Cheng et al., 2003; Vidmar et al., 2024) and the observed recruitment of topoisomerase at transcription start sites(Sutormin et al., 2022). Our experimental results provide a structural basis for how DNA supercoiling and transcription affect each other, modulating their mutual effects at different stages, a modulation also observed in eukaryotes albeit through different mechanisms(Baranello et al., 2016). Further studies will be required to fully elucidate the detailed mechanisms underlying this model.

## Materials and Methods

### Oligonucleotides and RNA

All DNA oligonucleotides listed below were obtained from Integrated DNA Technologies (IDT). All oligonucleotides except primers for PCR were purified in house using denaturing urea polyacrylamide gels (PAGE) prepared from SequaGel UreaGel 29:1 Concentrate (National Diagnostics).

**Table:** 

DNA oligonucleotides		
	Source	Sequence (5’ to 3’)
CBD01	IDT	AATACTAGAATTCTTATCAAAAAGAGTATTGACTTAAAGTCT AACCTATAGGATACTTACAGCCGAAAAAAGCAACAAAAAAA TTGAAAAAGGAAGAGTATGAGTATTCAAC
CBD02	IDT	AATACTAGAATTCCTGCATTAATGAATCGGCC
CBD03	IDT	AAAAGCACCGACTCGGTGCCACTTTTTCAAGTTGATAACG GACTAGCCTTATTTTAACTTGCTATTTCTAGCTCTAAAAC
CBD04	IDT	TTCTAATACGACTCACTATAGTGGTCATGAGATTATCAAA AGTTTTAGAGCTAGAAATAG
RNA oligonucleotides		
CBR01	Lab	GUGGUCAUGAGAUUAUCAAAAGUUUUAGAGCUAGAAAU AGCAAGUUAAAAUAAGGCUAGUCCGUUAUCAACUUGAA AAAGUGGCACCGAGUCGGUGCUUUU

### Preparation of single guide RNAs (sgRNAs)

sgRNA specific oligonucleotides and scaffold oligonucleotides (CBD03 and CBD04) were ordered from IDT and purified in-house by polyacrylamide-gel electrophoresis (PAGE). In a 30 μL reaction, oligonucleotides (20 μL, 50 μM) were annealed and extended by T4 DNA polymerase in CutSmart 1X buffer to produce the dsDNA template. After heat inactivation, 10 μL of this reaction (~33.3 μM dsDNA template) were used for a 20 μL in vitro transcription reaction using HiScribe T7 In vitro Transcription Kit (New England Biolabs). In vitro transcription reactions were performed at 37°C for 6 hours, after which Dnase I was added to degrade the DNA template. sgRNAs were purified by urea PAGE, Bands corresponding to the desired synthetic oligonucleotides and RNA were cut out as gel slices, eluted overnight at room temperature in 2X PK buffer (200 mM Tris-HCl, pH 7.5, 25 mM EDTA, pH 8.0, 300 mM NaCl and 2% SDS (w/v)), phenol chloroform extracted and precipitated with 2X volume of 200-proof 100% ethanol (Koptec). Then, samples were air dried and suspended in UltraPure DNase/RNase-free distilled water (Invitrogen, Thermo Fisher Scientific).

### Cloning of Single promoter plasmid pUC19-T7A1-U

Single promoter plasmid pUC19-T7A1-U was generated using around-the-horn PCR from template plasmid pUC19 using primers (CBD01/CBD02) with extensions containing the T7A1 promoter sequence as well mutations in the initially transcribed region to form a U-less cassette. These primers also contained EcoRI recognition sites for subsequent digestion and circularization by ligation. PCR products were digested with EcoRI and DpnI and were further purified by agarose-gel extraction. Purified PCR products were ligated using T4 DNA Ligase and transformed into DH5α cells. Plasmids from positive clones were extracted using QIAGEN miniprep kit and their sequences were confirmed by DNA sequencing. For large scale production of plasmids, plasmids were retransformed into DH5α/pUC19-T7A1-U cells, grown in 1 L of LB media, and extracted using QIAGEN Maxiprep kit following manufacturer instructions. For bulk biochemical as well as cryo-ET structural studies, supercoiled plasmid pUC19-T7A1-U was further purified using low-melting agarose (SeaPlaque Agarose, Lonza) from 1% agarose gel (1X TAE with 1X SYBR Safe DNA Staining). Under these electrophoretic conditions nicked DNA was well separated from supercoiled DNA. The band corresponding to supercoiled DNA was excised and extracted using β-Agarase (New England Biolabs), followed by phenol-chloroform extraction and ethanol precipitation. To desalt and concentrate the samples, the purified plasmids were further purified with Monarch PCR & DNA Cleanup Kit (New England Biolabs) using manufacturer instructions. The isolated purified plasmid was stored at −20 °C until further use.

### Generation of different DNA topologies

To generate different DNA topologies (nicked, ΔLk=0, ΔLk < 0, and ΔLk>0) for AFM, plasmids were treated with different enzymes to obtain the desired topology and they were further purified by agarose gel extraction using the β-Agarose described above. To generate nicked topology, plasmids (20 μg) were incubated in a 100 μL reaction with nicking endonuclease Nt.BspQI (20 units) in NEB 3.1 Buffer for 6 hours at 50 °C. For ΔLk = 0 topology, nicked plasmids (20 μg) were re-ligated, they were incubated in a 20 μL mixture with T4 DNA ligase (800 units) in T4 1X Buffer at room temperature. For ΔLk < 0 topology, we used isolated plasmids with their native supercoiling degree as extracted from the stationary phase cells. For ΔLk > 0 topology, plasmids (600 fmol) were incubated in a 20 μL reaction with reverse gyrase TopR2 (0.1 μM), and ATP (1 mM) in Reverse Gyrase 1X Buffer (50 mM Tris–HCl pH 8.0, 20 mM MgCl_2_, 100 mM NaCl, 0.5 mM DTT, and 0.5 mM EDTA) for 1 hour at 75 °C. Once plasmid topologies were generated, plasmids were purified using the Monarch PCR DNA Clean-up Kit.

### Expression and purification of *Streptococcus pyogenes* catalytically inactive dCas9 and nickase Cas9 D10A (SpCas9 D10A)

Purification of SpCas9 D10A was performed using a reported protocol(Jinek et al., 2012). Briefly, plasmid MJ825 (Addgene, # 39315) or MJ841 (Addgene, # 39318) was transformed into BL21(DE3) cells. One liter of Terrific Broth culture containing 50 μg/mL kanamycin was grown at 37°C. Upon reaching OD 0.6, cells were induced with IPTG to a final concentration of 0.5 mM at 20°C and were grown overnight (16 hours). Cells were harvested by spinning at 5000 rpm for 10 minutes and the cell pellet was stored at −80 °C. Cells were resuspended in 50 mL Lysis Buffer (50 mM Tris-HCl pH 7.5, NaCl 500 mM, 5% (v/v) glycerol, 1 mM DTT, supplemented with 4 tablets of mini-EDTA free protease inhibitor) and lysed using a sonicator. The lysate was centrifuged for 30 minutes at 4°C at 20,000 g. The clarified supernatant was passed through a HisTrap 5 mL column, washed with buffer NiA (50 mM Tris-HCl pH 7.5, NaCl 500m M, 5% (v/v) glycerol, 1 mM DTT, 10 mM imidazole) and eluted on a linear gradient with buffer NiB (50 mM Tris-HCl pH 7.5, NaCl 500 mM, 5% (v/v) glycerol, 1 mM DTT, 300 mM imidazole). Positive fractions containing SpCas9 D10A (verified by SDS-PAGE) were combined, and 2 mL of TEV protease were added and dialyzed overnight on Dialysis buffer (50 mM Tris-HCl pH 7.5, NaCl 500 mM, 5% (v/v) glycerol, 1 mM DTT). The samples were further purified on HiTrap Heparin column using a linear gradient of KCl from 200 mM to 1000 mM (50 mM Tris-HCl pH 7.5, 5% (v/v) glycerol, 1 mM DTT), followed by size-exclusion chromatography on a Superdex S300 column using Gel Filtration buffer (20 mM Tris-HCl pH 7.5, KCl 200 mM, 5% (v/v) glycerol, 1 mM DTT).

### Expression and purification of S. sulfataricus reverse gyrase (TopR2)

The gene fragment expressing the reverse gyrase (TopR2) was obtained by PCR from Sulfolobus solfataricus genomic DNA (ATCC, 35092D-5). This fragment was inserted into an expression plasmid with His6 N-terminal tag with a TEV protease site (2Bc-T, Addgene #37236) using ligation independent cloning. Protein expression was carried with this plasmid (2Bc-T- TopR2) following a reported protocol(Bizard et al., 2011). Briefly, plasmid 2Bc-T-TopR2 was transformed into Rosetta (DE3) cells. One liter of 2xYT culture containing 100 μg/mL ampicillin, 34 μg/mL chloramphenicol, and 1% glucose was grown at 37°C. Upon reaching OD 0.5, cells were induced with IPTG to a final concentration of 1 mM at 37°C and were grown for 6 hours. Cells were harvested by spinning at 5000 rpm for 10 min and the cell pellet was stored at −80°C. The pellets of induced cells were resuspended in Lysis buffer (40 mM Tris–HCl pH 8.0, 100 mM NaCl, 1 mM DTT, 0.1 mM EDTA). After sonication on ice, the sample was centrifuged at 30,000 g for 30 minutes. The resulting supernatant underwent heat treatment at 75°C for 13 minutes and was clarified by centrifugation at 30,000 g for 20 minutes. Polyethylenimine was then added to achieve a final concentration of 0.3%, and the mixture was stirred for 1 hour before being centrifuged at 40,000 g for 30 minutes. The supernatant was adjusted to 70% (NH4)2SO4 saturation, stirred for 30 minutes at 4°C, and centrifuged at 20,000 g for 30 minutes. The proteins were dissolved in buffer B (Lysis Buffer with 200 mM NaCl and 10% (v/v) ethylene glycol). After dialysis against buffer B, the proteins were loaded onto a 5 mL HiTrap Heparin HP column preequilibrated with buffer B using an FPLC AKTA system (GE Healthcare). The column was washed with 50 mL of buffer B, and bound proteins were eluted with a NaCl gradient ranging from 0.2 to 1.2 M NaCl in buffer B.

### SpCas9 D10A nicking assay

To test the activity of the sgRNA and nickase dCas9, a nicking assay was developed to monitor activity by electrophoretic mobility of DNA in agarose gel. sgRNA (90 fmol) was incubated at room temperature for 5 min with SpCas9 D10A (90 fmol) in Cas9 Reaction Buffer (20 mM HEPES pH 6.5, 100 mM NaCl, 5 mM MgCl_2_, 0.1 mM EDTA). Supercoiled plasmid (9 fmol) was added, and the reaction was kept at 37°C for one hour. The samples were treated with Proteinase K and then loaded into an agarose gel (1% agarose, 1X TAE 2.5 μg/mL chloroquine).

### DNA topoisomerase relaxation assay

The DNA relaxation activity of *E. coli* DNA topoisomerase I on supercoiled pT7A1-U was tested under different reaction conditions to evaluate the effect of transcription. In all reaction conditions, a total reaction volume of ~40 μL containing ~200 ng (~ 0.157 pmol) of negatively supercoiled plasmid was used and relaxation was carried out in TB40 buffer (20 mM Tris pH 8.0, 40 mM KCl, 5 mM MgCl_2_, 0.02 mg/mL BSA, and 1 mM DTT). An incubation at 37°C for 20 minutes was done for all conditions prior to the addition of *E. coli* Topoisomerase I (New England Biolabs, M0301S, 0.720 pmol). (1) For Topoisomerase I only condition, topoisomerase was added after an incubation of 37°C for 20 minutes. (2) To test the effect of transcription initiation, *E.coli* RNA Polymerase Holoenzyme (New England Biolabs, M0551S, 1.700 pmol) was supplemented to the previous reaction condition during the 37°C incubation. (3) To test the effect of a stalled transcription complex, the previous reaction condition was supplemented with 10 μM of ATP, GTP, CTP (Thermo Scientific, R0481) and 50 μM of GpA dinucleotide (TriLink Biotechnologies). (4) To test the effect of active transcription elongation, NTPs were supplemented in combination with Topoisomerase I at concentration of 100 μM of ATP, GTP, CTP, UTP (Thermo Scientific, R0481). (5) To test for the effect of a dCas9: sgRNA complex, a sgRNA:dCas9 complex at a concentration of 1.55 μM was first formed by incubating 15.5 pmoles of sgRNA and 15.5 pmoles of dCas9 in TB40 buffer (10 μL) at 25°C for 10 minutes. Afterward, the sgRNA: dCas9 complex was incubated with the supercoiled plasmids for 20 minutes at 37 °C.

In each assay, after 20 minutes of incubation, *E. coli* Topoisomerase I (NEB, 0.72 pmol) was added to a total reaction volume of 40 μL and time-points were taken at 0, 1, 2, 3, 5, 10, 15, and 30 minutes and quenched in a 100 μL of a Stop Buffer containing 8 U Proteinase K (NEB), 4 μL Glycogen, and 86 μL of 2X PK buffer (Tris 200 mM pH 7.5, EDTA 25 mM, NaCl 300 mM, SDS 2%). The timepoints were extracted with phenol (pH > 7.5) and phenol/chloroform/isoamyl alcohol (25:24:1 Mixture, pH 6.7/8.0, FisherBioReagents^™^) and then precipitated with 70% ethanol and redissolved in EB buffer. The time points were electrophoresed using a 1.5% (w/v) agarose gel that was run at 80V for 3 h with 1X TAE buffer (40 mM Tris-acetate, pH 8.1, 2 mM EDTA) in ice and post-stained in a solution of SYBR Safe and water before being visualized using Typhoon imager.

### 2D agarose gel electrophoresis

#### DNA topoisomer ladder generation

Plasmid pUC19-AmpR was nicked with Nt.BspQI (New England Biolabs). Nicked pUC19-AmpR plasmids (400 ng) were religated in 1X T4 DNA ligase buffer with increasing amounts of ethidium bromide (0, 40, 80, 120, 160, 200, 400, and 800 ng) using 100 U of T4 DNA ligase (New England Biolabs) in 40 μL reactions. The reaction proceeded overnight at room temperature and was quenched with Proteinase K (NEB), followed by extraction using 2-butanol, phenol-chloroform extraction, and ethanol precipitation. A mixture of the different ligation reactions was made to generate the 2D topoisomer ladder.

#### Separation of DNA topoisomers in 2D agarose gel

A large 2% agarose slab was cast in 1X TAE containing 2.5 μg/mL chloroquine. 100 ng of plasmid was loaded on a narrow lane in the 2D gel and electrophoresis was carried out with the following conditions: 1) For the first dimension: 40V (22 hours) in 1X TAE 2.5 μg/mL chloroquine and 2) For the second dimension: 60V (8 hours) in 1X TAE 25 μg/mL chloroquine. The gel was then stained using SybrSafe before imaging on Typhoon FLA 9500.

### In vitro Transcription assay

To test the effect of DNA supercoiling, soft-torsional block by dCas9 and Topoisomerase I in single-round transcription elongation, we performed experiments using radiolabeled RNA. Stalled elongation complexes labeled with radiolabeled nascent RNA were formed by assembling *E.coli* RNA polymerase Holoenzyme (10 pmol, New England Biolabs) in pUC19-T7A1-U (1 pmol) TB40 buffer (20 mM Tris pH 8.0, 40 mM KCl, 5 mM MgCl_2_, 0.02 mg/mL BSA, and 1 mM DTT) in the presence of 10 μM of ATP, GTP, CTP (Thermo Scientific, R0481) and 50 μM of GpA dinucleotide (TriLink Biotechnologies) and α-P32-ATP (Perkin Elmer). The reactions were incubated for 20 min at 37 °C and then placed on ice. The excess of radiolabeled nucleotides was removed using a size-exclusion column Microspin G-25 (GE Healthcare). The stalled complexes were reinitiated by adding NTPs at 10 μM and reaction allowed to proceed at room temperature. Time points were quenched using 2X RNA formamide loading dye and taken at 0, 20, 40, 60, 80, 100, 120, 180, 240, 300, 450, 600, 750, 900, 1050, 1200, 1350, 1500, 1650, 1800 s. Quenched samples were ran in 10% denaturing urea gel 19:1 acrylamide/bisacrylamide, the resolved RNA were imaged on Typhoon FLA 9500 and the bands were quantified with ImageQuant TL 8.2.

The reactions were performed using nicked and supercoiled DNA templates. For conditions where dCas9 was used, dCas9:sgRNA targeting were incubated at 10 min after addition of *E.coli* RNAP Holoenzyme. When Topoisomerase I was used, *E.coli* Topoisomerase was added 10 min after addition of *E coli* RNAP and allowed to relax DNA before isolating stalled elongation complexes.

### Gel quantification

The gel images were initially adjusted using the GIMP cage transformation function to correct bending along the horizontal direction, facilitating subsequent auto-processing steps. The quantification of gel intensity was facilitated by using the Python script gel_lane_finder script(Aquilina and Dunn, 2023), which enable the automated annotation of gel lanes. After normalizing the gel background, the two-dimensional band was averaged in the horizontal direction, resulting in a one-dimensional array representing the pixel-wise intensity along the annotated gel lane. To standardize the intensity values, which may be influenced by variations in sample loading for each lane, the sum of pixel intensity in the one-dimensional array within each lane was employed to normalize the intensity at each pixel point. A 3D plot was then generated, incorporating normalized gel pixel intensity, pixel position, and lane number (representing time points). Specific representative pausing positions were traced across the temporal evolution of the transcription.

### Atomic Force Microscopy

#### Slow scan Atomic Force Microscopy (AFM)

To inspect the quality of the sample preparation, linear (1 nM) or circular DNA (1–2 nM) was diluted in 10 mM HEPES or MOPS pH 7.0 and 5 mM MgCl_2_. TEC complexes were diluted in TB40 buffer to a final concentration of DNA of 2 nM. Two microliters of DNA or TEC complexes were deposited on freshly cleaved mica and incubated at room temperature for 2 or 3 minutes. Mica was rinsed with 50 μL of water five times and dried under N_2_ gas flow. AFM measurements were performed with a Multimode AFM Nanoscope 8 (Bruker Co.). The samples were imaged in tapping mode; the silicon cantilevers (Nanosensors) were excited at their resonance frequency (280–350 kHz) with free amplitudes of 2–10 nm. The image amplitude (set point As) and free amplitude (A0) ratio (As/A0) was kept at 0.8. All samples were imaged at room temperature in air, at a relative humidity of 30%.

#### AFM Image processing

Raw static AFM images acquired in air were flattened and leveled using Gwyddion 2.5142. Individual frames from HS-AFM movies were pre-processed using customized algorithms written in Igor Pro (Wave Metrics Inc. Oregon). The noise was reduced by a Gaussian filtering followed by a flattening filter, then the entire molecule was tracked using a 2D correlation method to reduce lateral drift143. To extract quantitative information from each frame, a mask of the entire DNA molecule from each frame was extracted using a customized batch processing script written in Gwyddion.

### Cryo-ET sample preparation for transcriptional modulation of DNA supercoiling

#### Preparation of circular plasmid under low salt and high salt conditions

For the preliminary investigation into the conformational dynamics of DNA supercoiling in response to varying ionic strength, the above extract and purified pUC19(T7A1U) plasmid was diluted to a concentration of 100 nM under two distinct buffer conditions: low salt and physiological salt buffer (20 mM Tris-Cl, 0.5 mM DTT, and 8% Trehalose; 20 mM Tris-Cl, 40 mM KCl, 5 mM MgCl_2_, 0.5 mM DTT, and 3% Trehalose, respectively) preceding the application onto the EM grids.

#### Formation of RNAP-pUC19(T7A1U) stalled complex

To investigate the localization of RNAP on the plasmid, a 10 μL incubation reaction was prepared to induce RNAP stalling 20 nucleotides downstream from the TSS of the T7A1U promoter. This was achieved by employing UTP starvation in a system composed of 60 nM pUC19-T7A1U plasmid, 180 nM RNAP (3:1 ratio to the plasmid), 50 μM GpA dinucleotide primer (facilitating RNAP initiation), and 10 μM rNTP mix (excluding UTP). The reaction buffer consisted of 20 mM Tris-Cl, 40 mM KCl, 5 mM MgCl_2_, 0.5 mM DTT, and 8% Trehalose. This reaction was then allowed to incubate for 20 minutes at 37°C to create the RNAP stall complex before application onto the TEM grids.

#### Formation of dCas9-pUC19(T7A1U) complex

Following the same incubation protocol as described above, the RNAP in the system was replaced by dCas9, an alternative DNA-binding protein. To do so, the dCas9 protein and its guiding RNA (sgRNA) were first assembled in a separate vial at a 1:1 ratio, reaching a concentration of 500 nM. This assembly was achieved by incubating the dCas9-sgRNA mixture in a buffer containing 20 mM Tris-Cl, 40 mM KCl, 5 mM MgCl_2_, and 0.5 mM DTT for 10 minutes at room temperature. Subsequently, the incubated dCas9-sgRNA complex was introduced into the plasmid, yielding a system containing 60 nM pUC19-T7A1U, 60 nM dCas9-sgRNA (2:1 ratio to the plasmid), a GpA dinucleotide primer (50 μM) for initiation, and an rNTP mix (10 μM, excluding UTP). The reaction buffer composition remained consistent with 20 mM Tris-Cl, 40 mM KCl, 5 mM MgCl_2_, 0.5 mM DTT, and 8% Trehalose. This reaction mixture was further incubated for 20 minutes at 37°C before being applied to the TEM grids for further analysis.

#### Formation of transcribed (none-equilibrium) RNAP-pUC19(T7A1U) complex

To study the activation of RNAP transcription on the negatively supercoiled plasmid, the previous RNAP stalled complex was first prepared. At the end of its 20 minute incubation at 37°C, rNTP (including UTP) was added to the solution, adjusting the concentration of each nucleotide to reach 100 μM. The transcription reaction continued for 10 minutes at room temperature before being transferred onto ice prior to application onto the TEM grids.

#### Formation of stalled and transcribed RNAP-dCas9-pUC19(T7A1U) complexes

To study active RNAP transcription on the negatively supercoiled plasmid in the presence of dCas9 torsional block, the previous dCas9-pUC19(T7A1U) complex was first prepared. Subsequently, RNAP was introduced into the incubated solution at a 3:1:1 ratio to dCas9 and plasmid, with concentrations of 180 nM, 60 nM, and 60 nM, respectively, maintained under the same physiological salt condition. In this sequence, the addition of rNTP (10 μM final concentration, excluding UTP) allowed the accumulation of mild torsion as RNAP initiated transcription from the TSS to the U-less stalling site, spanning 20 nucleotides. To induce additional torsional stress in the system, rNTP (including UTP) was added to the stalled RNAP-dCas9-pUC19(T7A1U) complex at 100 μM final concentration. The sample was subjected to a 10 minutes incubation at room temperature for DNA transcription before being transferred to the EM grids.

#### Formation of transcribed RNAP-topI-pUC19(T7A1U) complexes

To relieve RNAP from its apical constraint, topoisomerase I was introduced into the transcription system. The stalled RNAP-pUC19(T7A1U) complex was initially prepared following the same established protocol. After a 20 minute incubation at 37°C, topoisomerase I was added to the solution at a ratio of 3:1:1 with respect to RNAP and plasmid at concentrations of 180 nM, 60 nM, and 60 nM, respectively, under the same physiological salt condition. Subsequently, rNTP (including UTP) was added to the solution mixture, reaching a final concentration of 100 μM, and the transcription persisted for 10 minutes at room temperature before application onto the EM grids.

### TEM specimen preparation

3 μL of a sample after the above incubation was deposited onto a glow-discharged (PELCO easiGlow^™^ Glow Discharge Cleaning System) 200 mesh Quantifoil gold grid (hole size ranging from 1 micron to 2 microns, Electron Microscopy Sciences) for 30 seconds. Following a 20-second on-grid incubation, the grid was plunge-frozen in liquid ethane at ~95% humidity and 15°C using a Leica EM GP rapid-plunging device (Leica, Buffalo Grove, IL, USA) after controlled blotting with filter paper (3–5 s). The resulting flash-frozen grids were then transferred into liquid nitrogen for storage.

### TEM data acquisition

The Cryo-EM data were acquired using a Titan Krios (FEI) transmission electron microscope operating at 300 kV high tension and equipped with a Gatan energy filter. Imaging was performed with a Gatan K3 Summit direct electron detection camera, employing a magnification of ~53 kx (where each pixel of the micrographs corresponds to 1.67 Å in specimens) in super-resolution and correlated double sampling (CDS) mode. Tilt series of the samples were captured from −55° to +55° at 5° increments using a dose symmetry scheme. Data collection was automated using SerialEM software(Schorb et al., 2019) to track the specimen and maintain a defocus of ~2.5 μm. The total dose for the tilt image series ranged from ~110–150 e-/Å^2^. At each tilt angle, a total of 8 frames were collected, with an exposure time of 0.25 s per frame.

### Cryo-ET tilt series preprocessing and 3D reconstruction

The beam-induced motion of cryo-EM frames was corrected using MotionCor2(Zheng et al., 2017). To improve the contrast of low-dose cryo-ET tilt series images, a deep learning-based denoising method (NOISE2NOISE(Buchholz et al., 2019)) was implemented, involving the division of motion-corrected movie frames into even and odd halves (Figure S2A, I). Predictions for even and odd frames for a single tilt were averaged together. The defocus value of the cryo-ET tilt series was determined using GCTF(Zhang, 2016). A carbon area perpendicular to the tilt axis was included during data collection to assist in contrast transfer function (CTF) detection and later tilt series alignment. The determined CTF phase and amplitude were corrected by TOMOCTF^(^Fernandez et al., 2006^)^, based on a strip-based periodogram averaging method (deltaD=1000, w1=0.7, w2=0.25). The tilt series were initially aligned using IMOD(Kremer et al., 1996) and subsequently imported into e2tomo software(Chen et al., 2019) for deep learning-based particle identification and segmentation (Figure S2A, II). Approximately 100 tomogram patches containing DNA features were cropped for manual annotation and subsequently submitted for training. This convolutional neural network (CNN) was then applied across all tomograms for DNA identification (Figure S2A, III).

### Individual particle 3D reconstruction refinement and modeling

After the deep learning-based DNA annotation, individual plasmid particles were manually labeled in the tomogram (Figure S2A, III, right). In brief, the DNA-annotated tomograms were binned by 4 (6.68 Apix) and divided into isolated small surface pieces within Chimera(Pettersen et al., 2004). Density segments from a single plasmid particle were then manually selected and grouped, followed by low-pass filtering to a 6 nm resolution to serve as the particle shape mask. The criteria for selecting plasmid particles from the annotated tomogram included: 1) the particle should display supercoiling, manifesting as a helical structure (cyan color) without nicking (red color) (Figure S2A, III, right), 2) plasmid particles should be isolated and not significantly entangled with others; and 3) particles not partially attached to the carbon area. The center of mass of the particle shape mask was calculated (Figure S2A, IV, left), and this information was used to crop 1400x1400 pixel local tilt series within initial low contrast large micrograph. This cropped tilt series, with a plasmid particle in the center, was binned by 7 and then submitted for local 3D reconstruction using functions from IPET (Zhang and Ren, 2012). To minimize artifacts caused by the limited tilt angle range, the 3D reconstructed maps were missing-wedge compensated using IsoNet software(Liu et al., 2022), employing the previously created low-resolution mask. The output map was then low-pass filtered to 3 nm, serving as the final map for the modeling.

In the construction of the 3D reconstructed maps for the supercoiled DNA, a spine line representing the particle's helical axis was initially computed (Figure S1F, first row, left). This involved applying a Gaussian kernel with a standard deviation of 6.7 nm to the map, followed by skeletonization using the 'lee' method from the scikit-image package. Along the determined spine line, a sampling cylinder with dimensions of 60 nm diameter and 15 nm height was generated, utilizing a 5 nm step. The DNA density within the cylinder was divided into two regions, and the weight centers for each region were recorded (Figure S1F, first row, right). Subsequently, the tracing centers were interconnected based on rotation and distance to the previous centers, with a subsequent round of manual correction undertaken to address misconnection cases, particularly in low-quality areas of the EM map (Figure S1F, second row, left). The resulting threaded points underwent final processing through smoothing and interpolation with a 2 nm spatial interval, yielding the final model for the 3D map of the supercoiled DNA (Figure S1F, second row, right).

### Evaluation of the cryo-ET 3D reconstruction resolution

The resolution for the individual particle reconstructions were estimated by two methods. 1) Data-to-Data based analysis: the Fourier Shell Correlation (FSC) was calculated between two independently reconstructed 3D maps, in which each map was based on one-half of the tilt-series (split by even and odd frames for each tilt). The frequencies at which the FSC curve first falls to values of 0.143 were used to represent the reconstruction resolution. 2) Data-to-Model based analysis: the FSC curve between the final 3D reconstruction and the density map converted from the corresponding fitting model was calculated. The frequencies at which the FSC curve fell below 0.5 was used to estimate the resolution. The density map of the fitting model was generated by pdb2mrc in EMAN software(Ludtke et al., 1999).

### Sub-tomogram averaging of RNAP and dCas9

The sub-tomogram averaging of RNAP and dCas9 particles was carried out utilizing the e2tomo package (v.299). Briefly, the CTF-corrected and imod-aligned raw tilt series were imported into the software, followed by e2tomo 3D reconstruction, yielding a 4×-binned tomogram (equivalent to 6.68 Å with an unbinned pixel size of 1.67 Å). Subsequently, approximately 100 particles for each protein type were manually selected, and ab-initio maps were reconstructed for the subsequent round of reference-based boxing. Employing a template matching threshold value (vthr) of 7.5, a total of 1781 and 875 particles were extracted for RNAP and dCas9, respectively. Particle cropping, alignment, and averaging procedures were executed using the e2spt_refine_new.py script. No symmetry was applied during particle alignment. Following 8 rounds of alignment (iters=p,p,p,t,p,p,t,r), resolutions of 14.1 Å and 17.6 Å were determined for RNAP and dCas9, respectively (0.143 cut-off), employing Fourier Shell Correlation (FSC) with odd and even particles from the masked, final average. By mapping the orientation-determined particles back to their tomogram using e2spt_mapptclstotomo.py, DNA-bound particles were selectively chosen if their center of mass was within a distance of <1 nm to any points of the plasmid model. A subset of 232 RNAP and 116 dCas9 particles was then subjected to another round refinement (iters=p,p,p,t,p), resulting in resolutions of 17.7 Å and 18.9 Å, respectively, evaluated under the same standards.

### Quantification of supercoiled DNA plasmid morphology

The quantification of the supercoiled DNA plasmid's morphology involved measurements of cylindricity, sphericity, radius of gyration, and writhe number. To quantify these characteristics, the eigenvalues of the gyration tensor were calculated and designated as r1, r2, and r3, with the relationship r1>r2>r3. The radius of gyration (R) was determined by the formula $\text{R}={\text{r1}}^{2}+{\text{r2}}^{2}+{\text{r3}}^{2}$. Sphericity was derived using the equation $1-({\text{r1}}^{2}-({\text{r2}}^{2}+{\text{r3}}^{2})/2)$, while cylindricity was calculated as $1-({\text{r2}}^{2}-{\text{r3}}^{2})$. The calculation of the writhe number was facilitated by the E-CAM polymer_data_analysis module, which implements a double integral computation method proposed by Klenin & Langowski(Klenin and Langowski, 2000).

### Determination of the curvature and apexes along the supercoiled DNA

To determine the curvature of the plasmid along its DNA trajectory, a loop iterated from index 1 to N-1 for each point on the DNA plasmid model. The curvature (κ) at each point (i) was computed with its two neighboring points (i-1 and i+1) using the formula κ=1/R=4S/fgh(Johnson, 1929), where R is the radius of the circle circumscribing a triangle formed by the three points. Here, S represents the area of the triangle, and f, g, and h are the side lengths opposite the vertices of the triangle. The curvature at each point along the DNA trajectory was color-coded using a gradient, with points exhibiting the largest curvature values near the distal ends of each plectoneme branch utilized as the apexes of the plasmid.

### Calculation of the distance between DNA along the plasmid's helical axis

To calculate the distance between DNA along the helical (writhe) axis, the points on the DNA plasmid model were sequentially sorted and segmented into different color groups using the apical points on the plasmid (Figure S4I, panel I). For each point (i) in a group, its nearest neighboring point in another color group was determined, and the center of mass as well as the distance between the two points were calculated. This calculation was repeated from point 1 to N for each segment, resulting in a list of centers and distances that were used to represent writhe helix axis and the distances between the DNA along the writhe helix (Figure S4I, panel II). Given the challenge of defining the start and end site for a circular plasmid, the apexes were utilized as the zero index to align the calculated distance list.

### Determination of the local writhe density of the plasmid

Utilizing the previously calculated writhe helical axis trace of the model (Figure S4I, panel II), the branches of the plasmid can be determined and split at the junction (Figure S4I, panel III). Each helical axis branch was color-coded, and these color codes were used to segment the points on the plasmid model based on their vicinity (Figure S4I, panel IV). To assess whether DNA supercoiling was evenly distributed within the plasmid branches, these branch segmentations were treated as smaller closed plasmids and subjected to writhe number calculation using the previously described method(Klenin and Langowski, 2000). As the length of the branches may vary, the calculated writhe number was normalized by dividing it by the length of the segment, yielding the local writhe density of branches from the plasmid.

### Construction of superimposed models and mapping RNAP position on plasmid

To align all models from the same experimental condition, orientation-determined particles within the same group were back-mapped to their respective tomograms. Concurrently, the plasmid model was also back-mapped to the tomogram, so that it allows the determination of the relative orientation between the plasmid and plasmid-bound particles (RNAP and dCas9). Given the current resolution limitations of cryo-ET in resolving DNA sequence information, the position of dCas9 was utilized as a marker to infer the TSS, assuming specificity in dCas9 binding as per the construct design. The entire length of the plasmid trace was normalized to 1950 base pairs, with the point nearest to the dCas9 particle designated as 1090 bp. As none of the particles are symmetrical, the upstream and downstream positions of the bound RNAP were determined relative to the plasmid.

### Corse-grained simulation of DNA supercoiling relaxation

Molecular dynamics (MD) simulations were conducted using the GPU-accelerated, sequence-dependent oxDNA2 model(Burns et al., 2016; Sulc et al., 2012). The simulation environment was set up under the canonical NVT ensemble (constant number of particles, volume, and temperature). To enhance computational efficiency, we employed a smaller, 525 bp minicircle DNA system with randomized sequences. The in-silico plasmid model was generated using TacoxDNA(Suma et al., 2019), with a DNA twist deficit of −5 and a writhe of 0, yielding a supercoiling density of −0.1. The circular DNA plasmid was initially energy minimized and relaxed using parameters tailored from oxDNA example simulations (available at dna.physics.ox.ac.uk; DOI: 10.5281/zenodo.4655472). These parameters included the following (thermostat = john, interaction_type = DNA2, newtonian_steps = 103, diff_coeff = 2.5, salt_concentration = 0.5, T = 300K, dt = 0.005, verlet_skin = 0.05, rcut = 2.0). The relaxation phase continued for 3x10^6^ steps (approximately 30 μs) until the formation of DNA plectonemes and the appearance of apices. After equilibration, the final frame structure was modified to create a nicked plasmid by introducing a single-strand break at base pair index 940 for models I and II, 828 for model III, and 564 for model IV. This nicked configuration enabled subsequent simulations of supercoiling torsional relaxation, designed to mimic the activity of Topoisomerase I (TopI).

Four DNA supercoiling torsional relaxation simulations were conducted starting from the nicked plasmid described previously. For model I, the nicked plasmid was allowed to relax without any external constraints for 3x10^7^ steps (equivalent to 300 μs). For the model II, III, and IV, a mutual force trap was applied to constrain the sharp apex region, covering approximately 20 base pairs. These traps, functioning as springs, were formed between base pairs at indices 798, 251 on one side and 817 and 232 on the other. The trap stiffness was set to 1, and r0 was defined as the initial distance between the mass centers of each pair of base pairs in the initial structure divided by a factor of 2. The second batch of simulations were also run 3x10^7^ steps. All simulations were repeated two times using the same relaxation parameters mentioned above. Movies were generated using the last run of each simulation at a rate of 1x10^5^ steps per frame.

The equilibrated final frame structure prior to the nick was also utilized to simulate the formation of DNA twin domains. In brief, two rotating harmonic traps were applied to the base pairs at indices 107 and 942, as well as indices 366 and 683, positioned within the intermediate region of the plectoneme. This approach segments plectoneme two sections: the first section contains an apex with constraints, while the second section features a free apex. This setup allows for a comparison of the effects of (+) torsional propagation on one strand, (−) torsional propagation on the other, and their mutual cancellation when the rotating trap is applied. The center of rotation was defined as the center of mass of the selected base pair, with the rotation axis determined as the unit vector extending from the center of the base pair to the center of mass of another base pair located 10 base pairs upstream. The stiffness of the trap was set to 10 in oxDNA, with a rotation rate of 1x10^5^. The confined apex was configured as described earlier. The simulation was run for 3x10^6^ steps and with one repeat. Movies were created with frames captured every 1x10^4^ steps.

### Lead Contact

Further information and requests for resources and reagents should be directed to and will be fulfilled by the Lead Contact, Carlos Bustamante (carlosb@berkeley.edu).

### Materials Availability

All unique reagents generated in this study are available from the lead contact with a completed Materials Transfer Agreement.

## Supplementary Material

Supplement 1

Supplement 2

Supplemental information

Document S1. Figures S1-S7

Supplementary Particle Gallery

Video S1. 3D reconstruction workflow of a representative negatively supercoiled 2 kbp plasmid particle

Video S2. Coarse-grained MD simulation of negatively supercoiled plasmid relaxation with and without apical constraint

Video S3. Coarse-grained MD simulation of negative DNA supercoiling in managing positive and negative torsion with and without apical constraints

## Data Availability

The cryo-ET maps of ~2 kb negatively supercoiled pUC19 plasmids under various conditions were montaged and deposited in the Electron Microscopy Data Bank (EMDB). These include maps of plasmids under low salt conditions (EMD-47843), near-physiological salt conditions without additional proteins (EMD-47844), with stalled RNA polymerase (EMD-47847), with dCas9 (EMD-47849), with RNA polymerase during active transcription (EMD-47850), with both stalled RNA polymerase and dCas9 (EMD-47851), with transcribed RNA polymerase and dCas9 (EMD-47853), and with transcribed RNA polymerase and Topoisomerase I (EMD-47855). The individual particle 3D reconstructed maps, models, FSC evaluations, original gel images, and analysis used to generate the statistical data presented in the figures have been deposited on GitHub Zenodo at (https://doi.org/10.5281/zenodo.14082680).
