# Supplementary material for: Apical Localization of RNA Polymerases Modulate Transcription Dynamics and Supercoiling Domains Revealed by Cryo-ET
Source: bioRxiv. 2026 Apr 16:2026.03.25.714350. Originally published 2026 Mar 26. Preprint. [Version 2] doi: 10.64898/2026.03.25.714350 (PMC13041910; doi:10.64898/2026.03.25.714350)
Supplement: 5 [file NIHPP2026.03.25.714350v2-supplement-5.pdf]

bioRxiv preprint doi: https://doi.org/10.64898/2026.03.25.714350; this version posted April 16, 2026. The copyright holder for this preprint (which was not certified by peer review) is the author/funder, who has granted bioRxiv a license to display the preprint in perpetuity. It is made available under aCC-BY 4.0 International license.

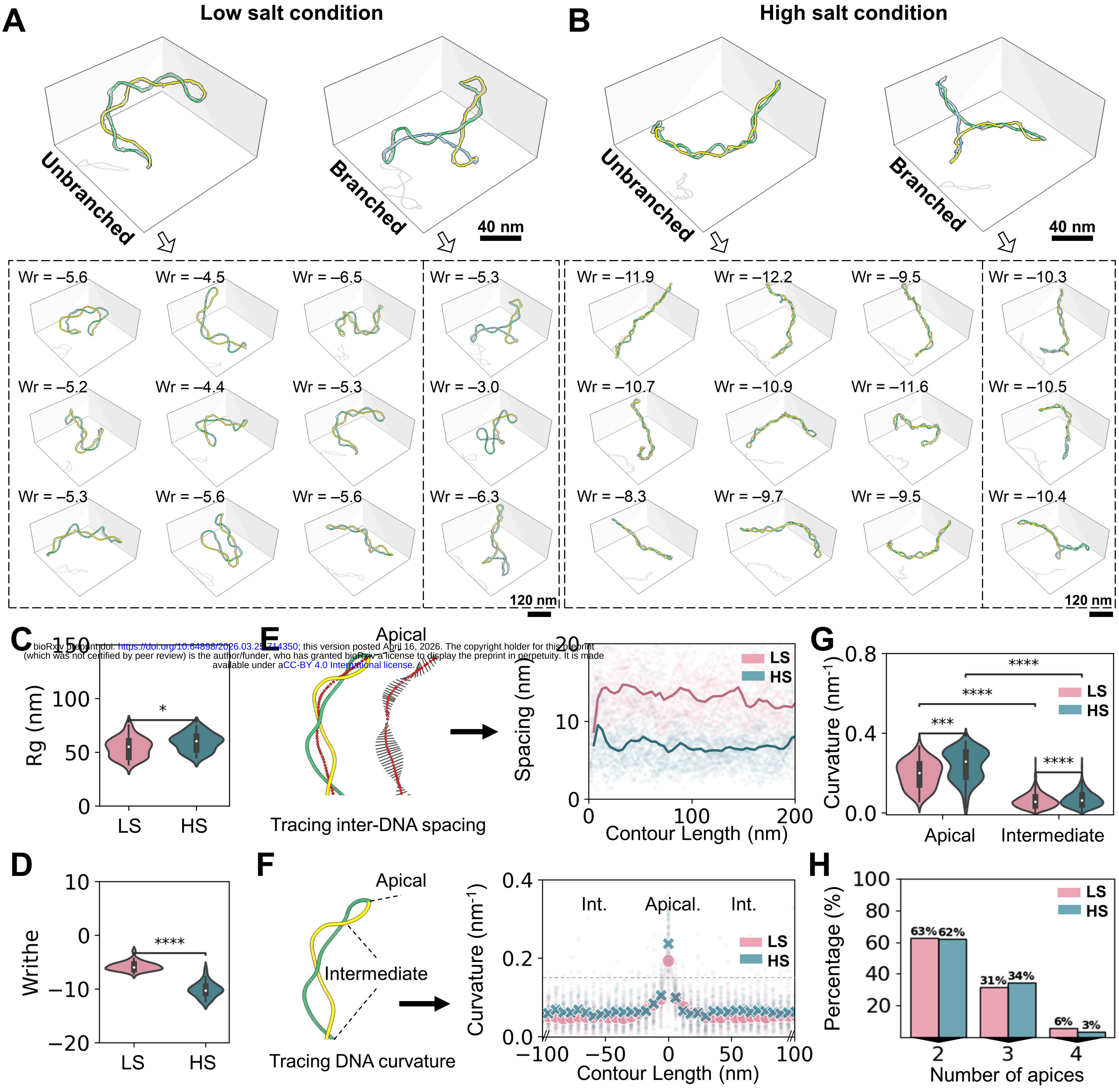

Figure S1: Structural analysis of –sc plasmid under low salt and high salt conditions, related to Figure 1
(**A-B**) Representative cryo-ET map and model of the –sc plasmid (ΔLk centered on -15) under low salt (LS) and high salt (HS) conditions, respectively. Two exemplary particles (unbranched and branch) are displayed on the top panel, with their collection presented on the bottom, featuring writhe numbers. (**C-D**) Statistical analysis of plasmid radius of gyration (Rg) and writhe number using violin plots, respectively; N=64. (**E**) Schematic of inter-DNA spacing quantification (black bars) along the plectoneme axis (red dots). The inter-DNA spacing of the plectoneme was plotted from the plasmid apex toward the intermediate region; N=158 (**F**) Schematic illustrating the apical and intermediate regions of a plectoneme, accompanied by DNA curvature quantification. (**G**) Statistical analysis of DNA curvature near the apical and intermediate regions of the plectoneme under LS and HS conditions; N=158 (**H**) Distribution of the number of apices of the plasmid in LS and HS conditions; N=64. Statistics are calculated using a Mann-Whitney test, where *p < 0.05, **p < 0.01, ***p < 0.001, ****p < 0.0001; and ns, not significant.

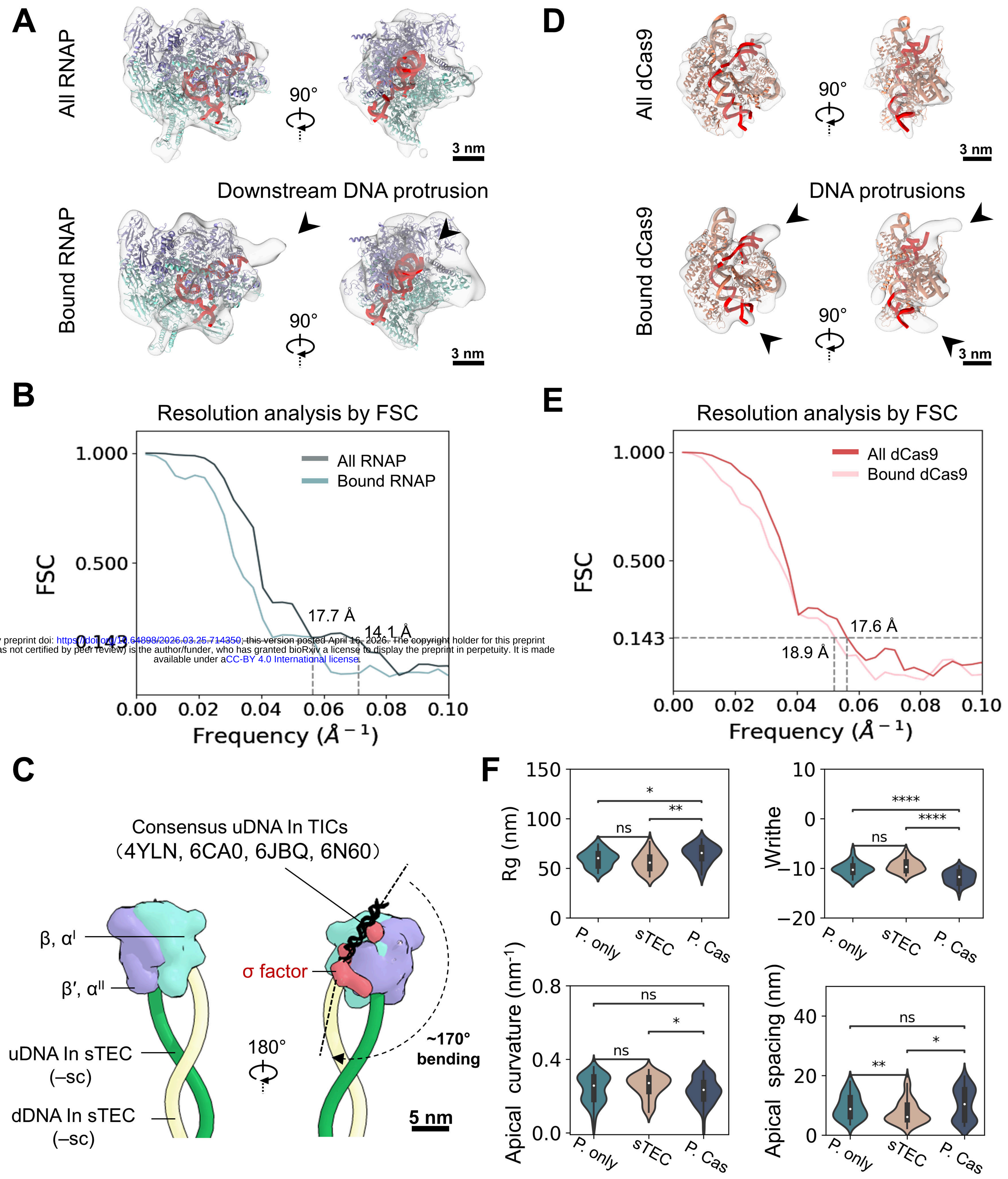

bioRxiv preprint doi: https://doi.org/10.64898/2026.03.25.714350; this version posted April 16, 2026. The copyright holder for this preprint (which was not certified by peer review) is the author/funder, who has granted bioRxiv a license to display the preprint in perpetuity. It is made available under aCC-BY 4.0 International license.

Figure S2: Sub-tomogram averaging analysis of apically bound RNAP and dCas9, related to Figure 2
(**A-B**) Two orthogonal views of the sub-tomogram averaged RNAP map docked with PDB structure 6ALH. Averaging all RNAPs selected from the tomogram (N=1781) yielded a 14.1 Å resolution map (top), while using only plasmid-bound RNAPs after inspection (N=232) resulted in a 17.7 Å resolution map (bottom). (**C**) Superimposition of consensus TIC upstream DNA (black helix) and the TIC σ-factor (red) from PDBs 4YLN, 6CA0, 6JBQ, and 6N60 onto the apically stalled TEC on –sc DNA reveals a ~170° upstream DNA bend. (**D-E**) Sub-tomogram averaged dCas9 map docked with PDB structure 6O0X. Averaging all dCas9 particles selected from the tomogram (N=875) yielded a 17.6 Å resolution map (top), while the plasmid-bound subset (N=116) achieved a resolution of 18.9 Å (bottom). Resolutions were estimated by measuring the Fourier shell correlation (FSC) between two independently determined half-maps at 0.143. (**F**) Statistical analysis of global plasmid Rg and writhe number (N=90), along with local DNA curvature and inter-DNA spacing at apical sites (N=207), for plasmid only, plasmid with stalled RNAP, and plasmid with dCas9, respectively. Statistics are calculated using a Mann-Whitney test, where $^{*}p < 0.05$, $^{**}p < 0.01$, $^{***}p < 0.001$, $^{****}p < 0.0001$; and ns, not significant.

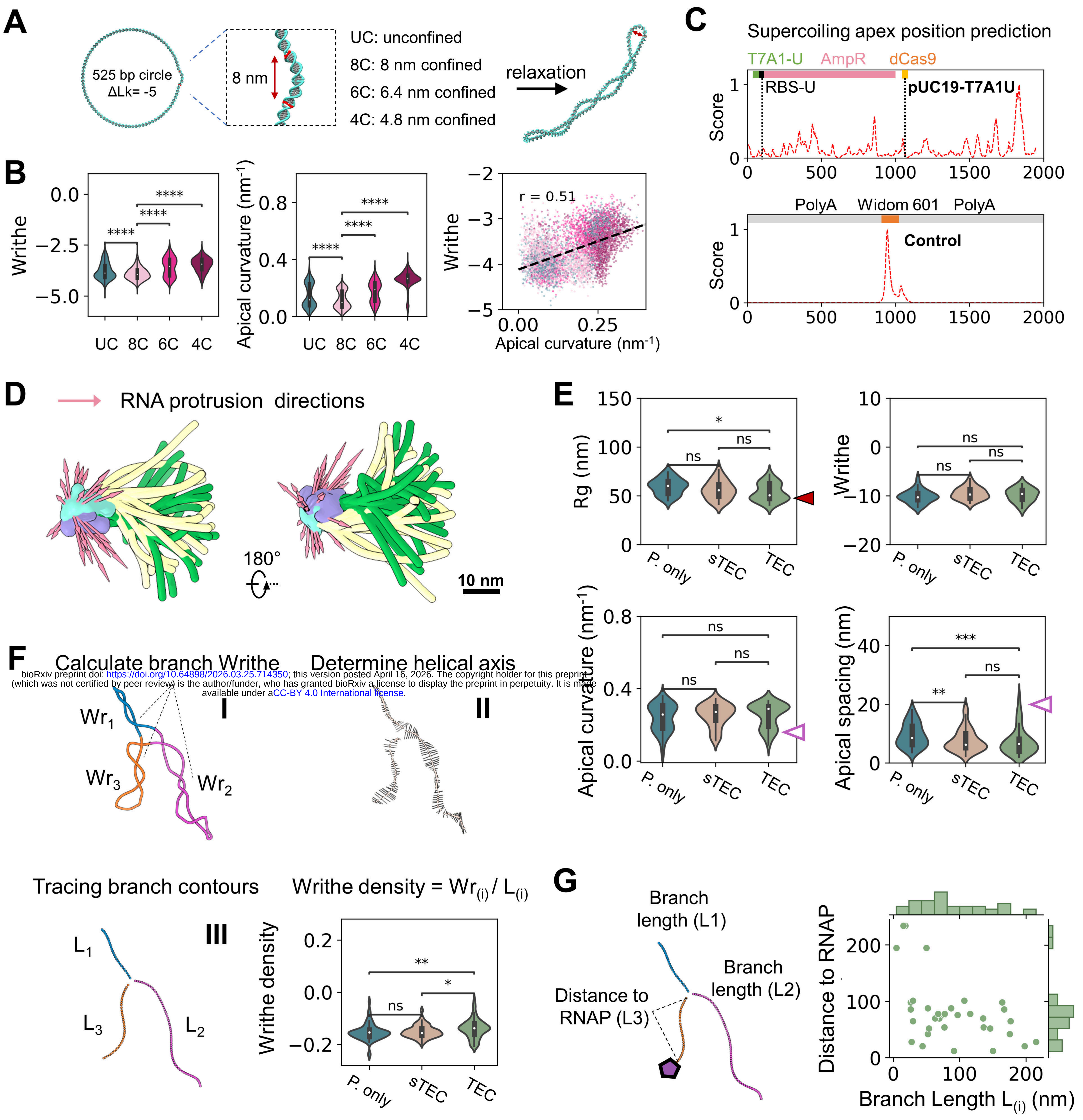

bioRxiv preprint doi: https://doi.org/10.64898/2026.03.25.714350; this version posted April 16, 2026. The copyright holder for this preprint (which was not certified by peer review) is the author/funder, who has granted bioRxiv a license to display the preprint in perpetuity. It is made available under aCC-BY 4.0 International license.

Figure S3: Quantification of plasmid geometry upon RNAP/dCas9 binding, related to Figure 3
(**A**) Generation of –sc DNA minicircles with a target site subjected to varying levels of constraint, defined by spring distances of 8, 6.4, and 4.8 nm, to modulate DNA relaxation. (**B**) Quantification of relaxed plasmid writhe, apical DNA curvature, and their correlation following MD simulation (sampled from frame 100 onward after relaxation; three replicates). (**C**) Predicted DNA plectoneme formation loci along the pUC19-T7A1U sequence. A control was generated using a polyA–Widom601–polyA sequence. (**D**) Overlay of apically bound RNAPs in TECs with RNA protrusion directions indicated by pink arrows. (**E**) Statistical analysis of global plasmid Rg and writhe number (N=79), along with local DNA curvature and inter-DNA spacing at apical sites (N=192) for sTECs and TECs. (**F**) Plectoneme branches were segmented (I), and their superhelical axes (II) and contour lengths (III) were determined. Writhe density was calculated as writhe number divided by segment contour length. Bottom right: writhe density distribution; N=187. (**G**) Plot of plectoneme branch length versus distance from the branch to the apical RNAP; N=38. Statistics are calculated using a Mann-Whitney test, where *p < 0.05, **p < 0.01, ***p < 0.001, ****p < 0.0001; and ns, not significant.

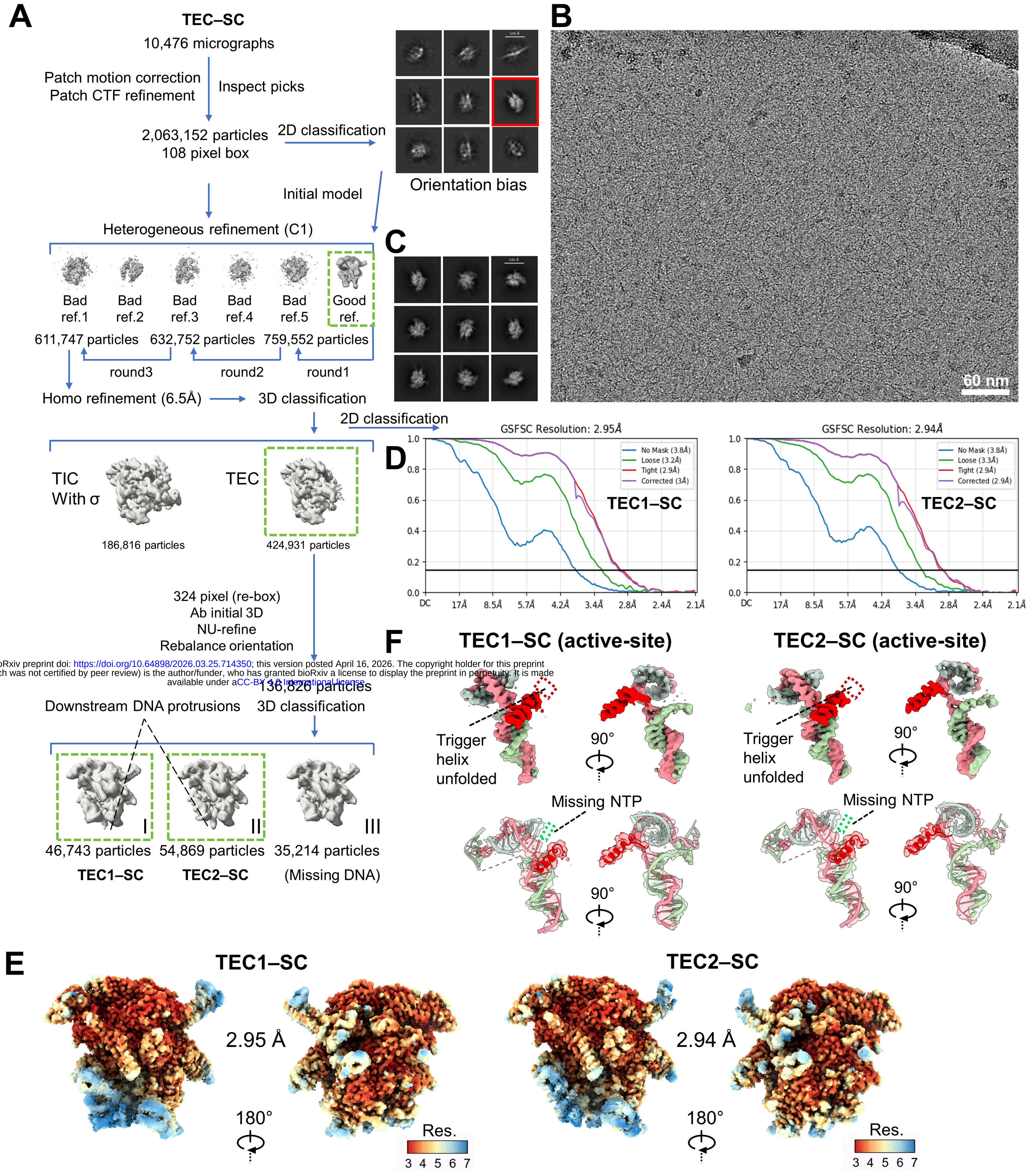

bioRxiv preprint doi: https://doi.org/10.64898/2026.03.25.714350; this version posted April 16, 2026. The copyright holder for this preprint (which was not certified by peer review) is the author/funder, who has granted bioRxiv a license to display the preprint in perpetuity. It is made available under aCC-BY 4.0 International license.

Figure S4. Cryo-EM analysis of the transcription elongation complex (TEC) on a –sc plasmid template, related to Figure 3

(**A**) Workflow of single-particle analysis and 3D reconstruction of TECs. Three final maps were obtained: the first (TEC1–SC, global resolution of 2.95 Å from 46,743 particles) and the second (TEC2–SC, global resolution of 2.94 Å from 54,869 particles) show particles with downstream DNA protrusion. The third class was not further analyzed due to the absence of DNA density, indicating likely unbound RNAPs. (**B**) Representative reference-free 2D class averages of TEC particles, displaying various TECs orientations. (**C**) Representative cryo-EM micrograph of TEC particles assembled on negatively supercoiled pUC19-T7A1U plasmid in vitreous ice. (**D**) Fourier shell correlation (FSC) curves between independently reconstructed even–odd maps. Resolution was estimated at the 0.143 FSC cutoff. (**E**) Cryo-EM density maps of TEC1–SC (left) and TEC2–SC (right), color-coded by local resolution, ranging from 3 Å (red) to yellow to 7 Å (blue). (**F**) Examination of RNAP active sites shows that both TEC1–SC and TEC2–SC are in a post-translocated state, with the trigger helix unfolded and the NTP absent.

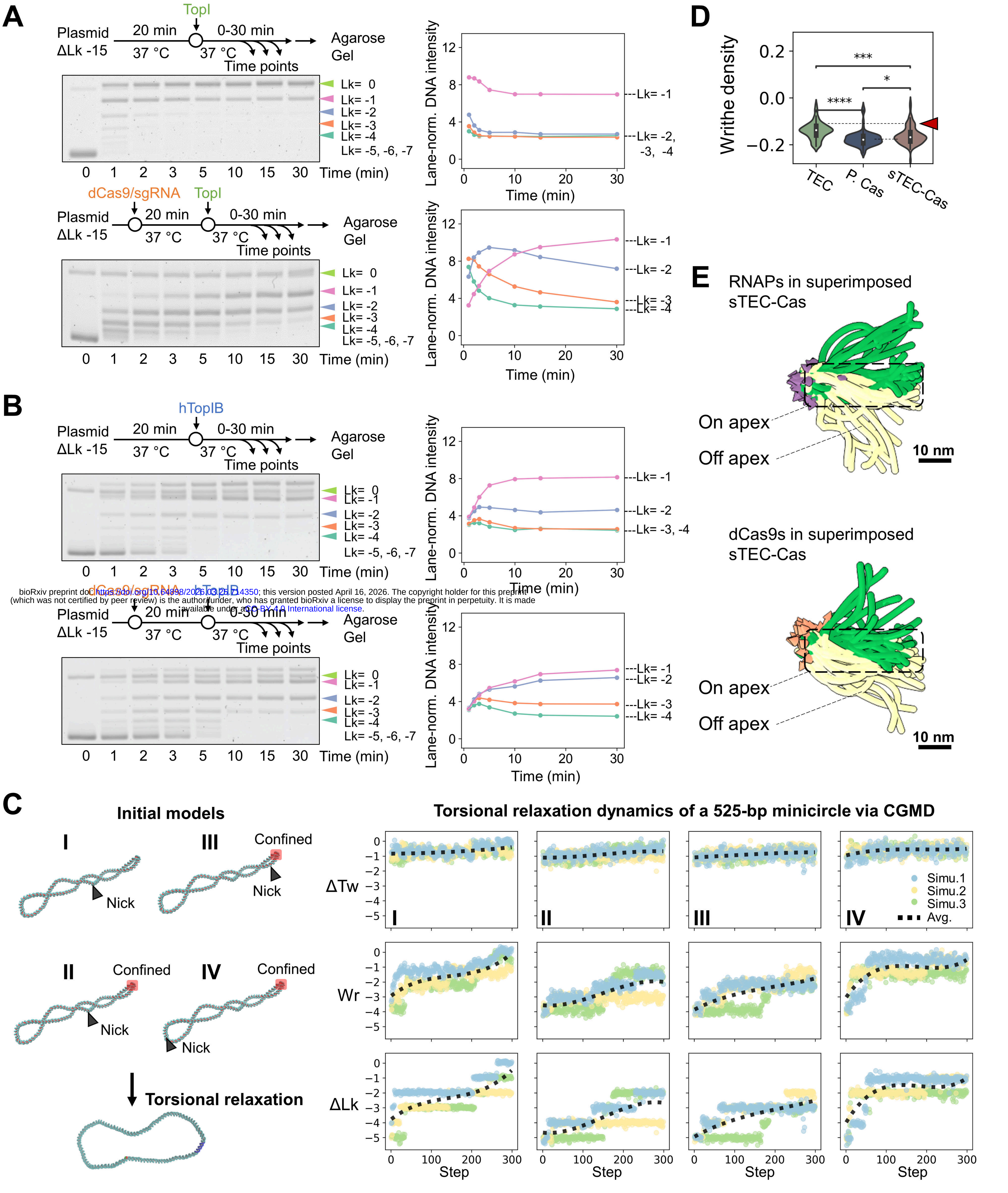

bioRxiv preprint doi: https://doi.org/10.64898/2026.03.25.714350; this version posted April 16, 2026. The copyright holder for this preprint (which was not certified by peer review) is the author/funder, who has granted bioRxiv a license to display the preprint in perpetuity. It is made available under aCC-BY 4.0 International license.

Figure S5: Apical binding of dCas9 hinders DNA rotation, related to Figure 4

(**A**) DNA supercoiling relaxation assay in the absence (top) and presence (bottom) of a dCas9. Right panel: quantification of ΔLk changes over time following TopI addition. (**B**) Control experiment of A, with TopI replaced by hTopIB for DNA supercoiling relaxation. (**C**) Coarse-grained MD simulations of 525-bp –sc (ΔLk = –5) minicircle plasmid relaxation dynamics, initiated with different nick locations. Simulations were conducted without apical confinement (Model I) and with apical restriction (Models II–IV). Topological parameters (ΔTw, Wr, and ΔLk) are shown in rows 1–3 for each model. Each simulation was performed in triplicate, with averages indicated by black dashed lines. (**D**) Statistical analysis of plectoneme branch writhe density distribution; N=226. (**E**) Superimposition of apical DNA segments illustrating the dynamics of stalled RNAP and dCas9 when co-present on the plasmid.

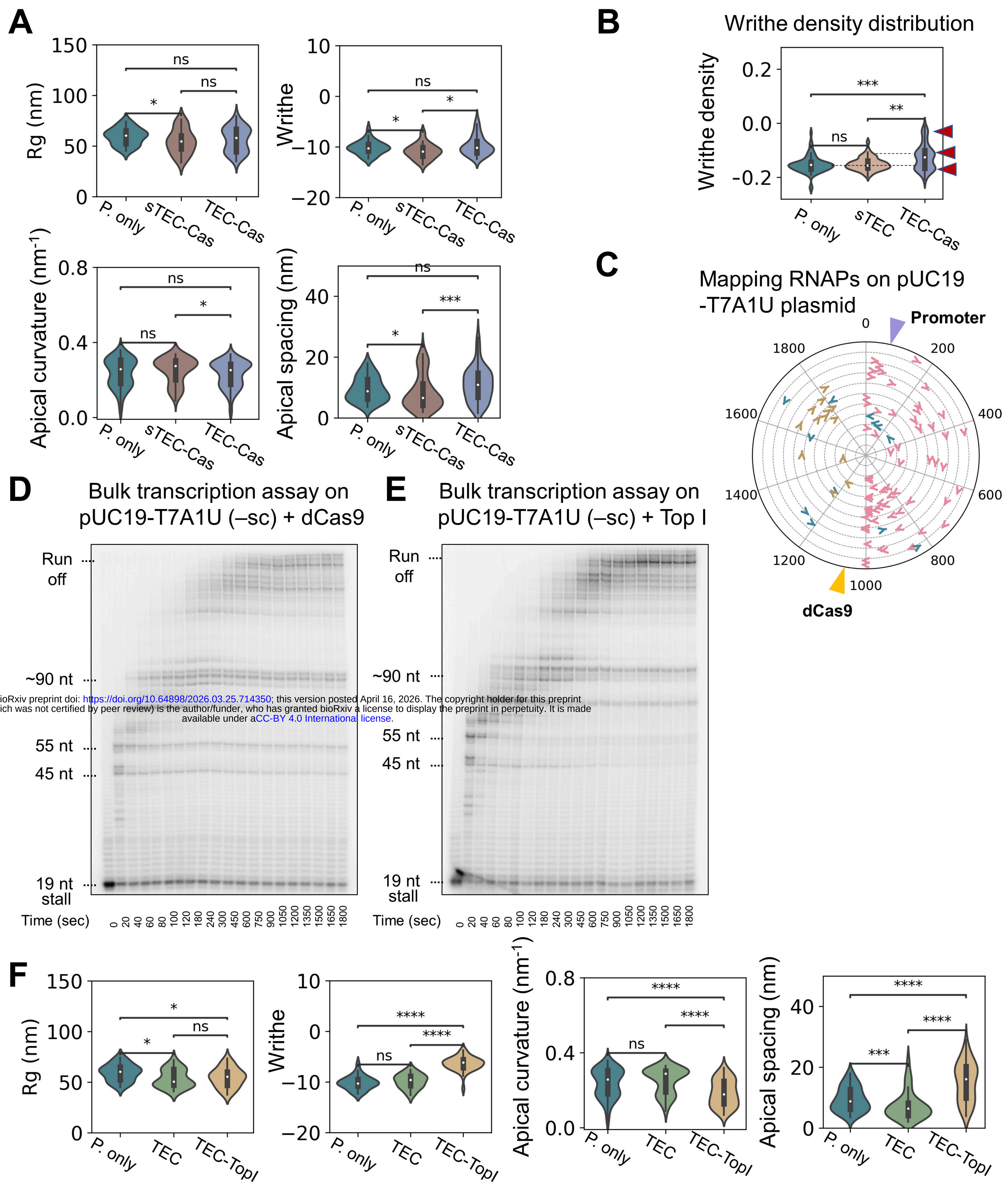

bioRxiv preprint doi: https://doi.org/10.64898/2026.03.25.714350; this version posted April 16, 2026. The copyright holder for this preprint (which was not certified by peer review) is the author/funder, who has granted bioRxiv a license to display the preprint in perpetuity. It is made available under aCC-BY 4.0 International license.

Figure S6: Quantification of plasmid morphology with co-presence of RNAP and dCas9, and of RNAP and TopI, related to Figures 4 and 5

(**A**) Statistical analysis of global plasmid Rg and writhe number (N=107), along with local DNA curvature and inter-DNA spacing at apical sites (N=270) for sTEC-Cas and TEC-Cas particles. (**B**) Statistical comparison of the plectoneme writhe density distribution; N=198. (**C**) Mapping all bound RNAPs on pUC19-T7A1U templates using dCas9 as fiducial. For visualization, different plasmids and their associated RNAPs are displayed at distinct radii in the polar plot. RNAP on the transcriptional region, oriented downstream, is marked in pink; RNAP on the non-transcriptional region or pointing upstream, is colored in tan and blue, respectively; N=89 (**D–E**) Electrophoresis assay of RNAP transcription on –sc pUC19-T7A1U templates in the presence of dCas9 and in the presence of TopI over time, respectively. (**F**) Same statistical measurements as in (A), performed on TEC-TopI particles (90 particles and 212 apices total) . All statistics are calculated using a Mann-Whitney test, where *p < 0.05, **p < 0.01, ***p < 0.001, ****p < 0.0001; and ns, not significant.

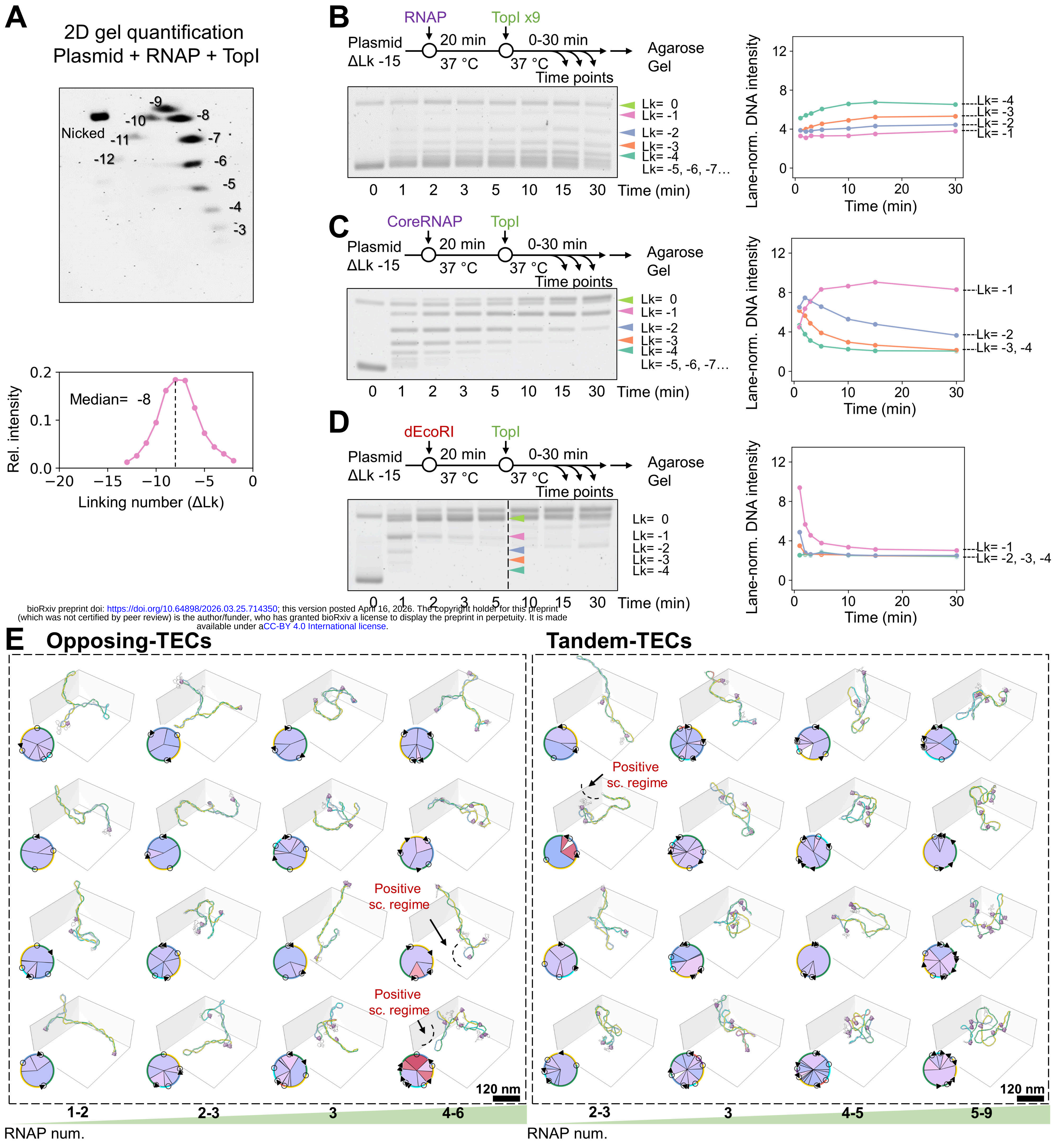

bioRxiv preprint doi: https://doi.org/10.64898/2026.03.25.714350; this version posted April 16, 2026. The copyright holder for this preprint (which was not certified by peer review) is the author/funder, who has granted bioRxiv a license to display the preprint in perpetuity. It is made available under aCC-BY 4.0 International license.

Figure S7: Top I and RNAP coupling slows supercoiling relaxation, and particle collections of dual-promoter TECs, related to Figures 5 and 6.

(**A**) 2D electrophoresis gel of –sc pUC19-T7A1U plasmid in the presence of RNAP and TopI, with quantification of the dominant ΔLk value. (**B**) 1D gel-based DNA supercoiling relaxation assay of plasmid in the presence of RNAP and a large excess of TopI. (**C**) DNA supercoiling relaxation assay of plasmid in the presence of RNAP core (lacking σ and unable to form stable transcription bubbles). (**D**) DNA supercoiling relaxation assay of plasmid in the presence of dEcoRI. 1D gel-based linking number quantification is shown in the right panel for B-D. (**E**) Particle collections of opposing-promoter TECs and tandem-promoter TECs, ordered by the number of bound RNAPs. Each dual-promoter TEC is also shown with its corresponding circular plasmid layout, annotated in red to indicate plectonemes extending into the positive supercoiling regime.
