# Supplementary material for: Apical Localization of RNA Polymerases Modulate Transcription Dynamics and Supercoiling Domains Revealed by Cryo-ET
Source: bioRxiv. 2026 Mar 26:2026.03.25.714350. Preprint. [Version 1] doi: 10.64898/2026.03.25.714350 (PMC13041910; doi:10.64898/2026.03.25.714350)
Supplement: Supplementary file 1 [file NIHPP2026.03.25.714350v1-supplement-2.pdf]

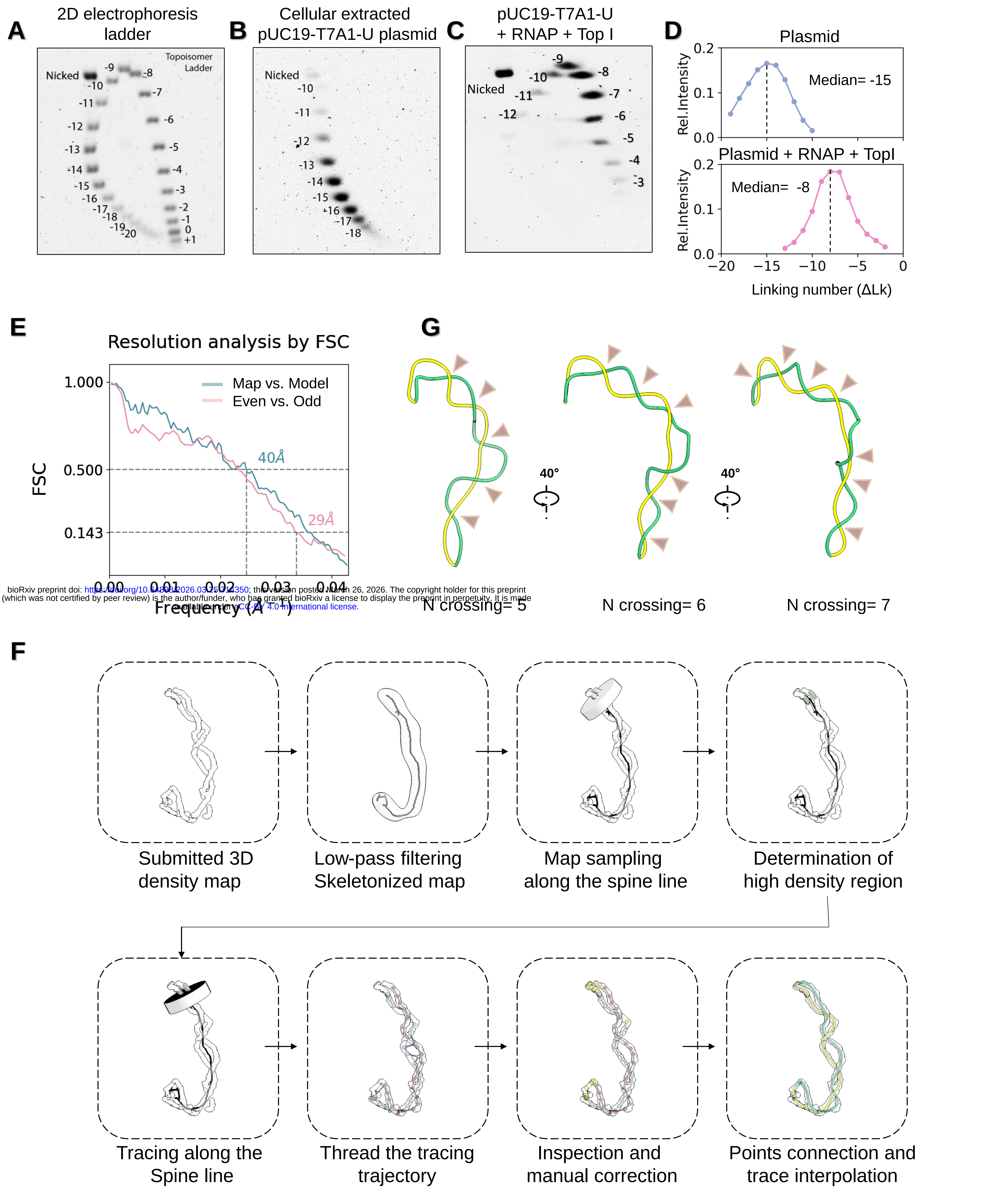

bioRxiv preprint doi: https://doi.org/10.64898/2026.03.25.712350; this version posted March 26, 2026. The copyright holder for this preprint (which was not certified by peer review) is the author/funder, who has granted bioRxiv a license to display the preprint in perpetuity. It is made available under a CC-BY 4.0 International license.

Figure S1: Plasmid topology quantification and modeling

(**A**) 2D electrophoresis ladder used to annotate the cellular-extracted T7A1U plasmid. (**B-C**) 2D electrophoresis gel of cellular-extracted T7A1U plasmid and the same plasmid with RNAP and TopI after 30 minutes of reaction, respectively. (**D**) Gel quantification of the dominant ΔLk number in B (top) and C (bottom). (**E**) Map resolution evaluation for a representative particle in Fig. 1E. Resolution was estimated by measuring the Fourier shell correlation (FSC) between two independently determined half-maps (even-odd dose frames, pink) at 0.143 and between the map and fitted model (blue) at 0.5. (**F**) Model fitting workflow, see also materials and methods. (**G**) Three rotational views of a pUC19-T7A1U plasmid particle showing the variation in the number of DNA crossings. The DNA crossing positions are indicated by tan-colored arrowheads.

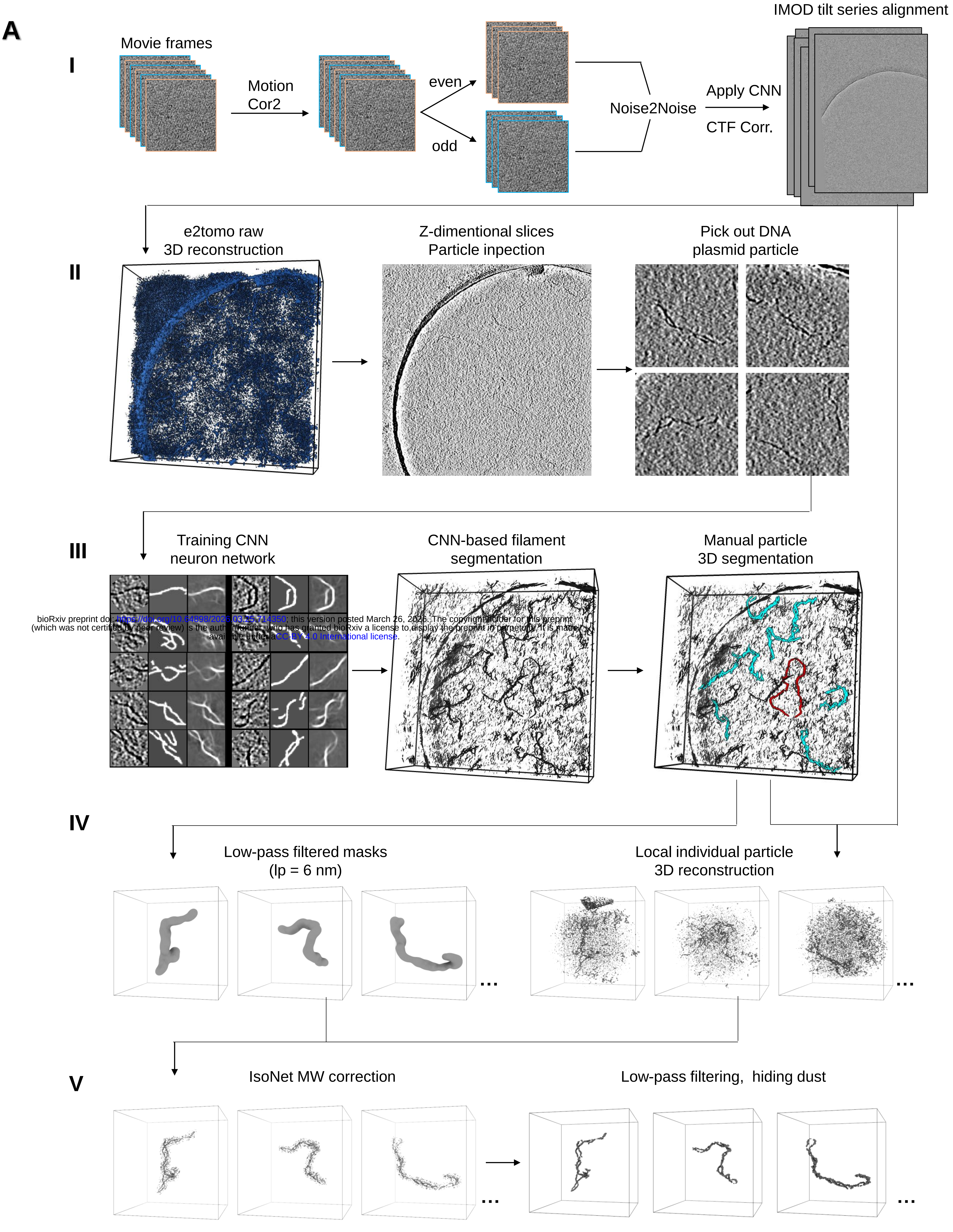

bioRxiv preprint doi: https://doi.org/10.64898/2026.03.25.714350; this version posted March 26, 2026. The copyright holder for this preprint (which was not certified by peer review) is the author/funder, who has granted bioRxiv a license to display the preprint in perpetuity. It is made available under aCC-BY 4.0 International license.

Figure S2: Cryo-ET 3D reconstruction work flow
(**A**) Tomographic 3D reconstruction workflow of the plasmid particle from the tilt series. I: The motion-corrected tilt series were split into even and odd sets and subjected to deep learning-based image denoising (N2N method). II: The denoised tilt series were initially patch tracking aligned in imod and submitted for full tomogram reconstruction in e2tomo. III: DNA segments selected from the large 3D map were used to train a CNN network for annotating all DNA elements in the tomogram. IV: A mask of the target particle was created to calculate its center of mass, which was then used to extract raw tilts from the initial low-contrast micrograph for localized 3D reconstruction of individual particles. V: The map was processed with IsoNet for missing wedge correction, followed by low-pass filtering to 3 nm and dust removal, resulting in final map for modeling.

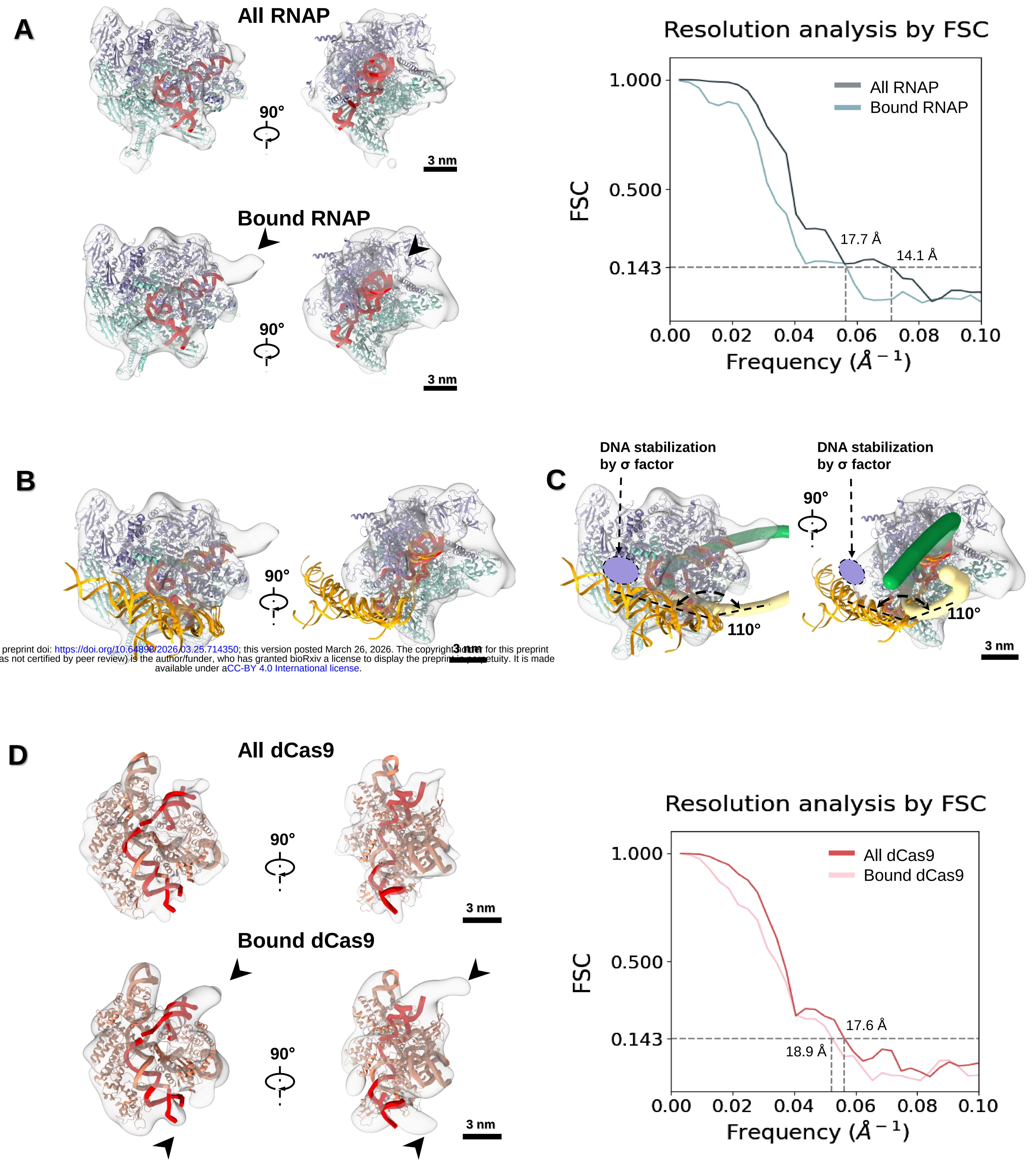

bioRxiv preprint doi: https://doi.org/10.64898/2026.03.25.714350; this version posted March 26, 2026. The copyright holder for this preprint (which was not certified by peer review) is the author/funder, who has granted bioRxiv a license to display the preprint in perpetuity. It is made available under aCC-BY 4.0 International license.

Figure S3: Sub-tomogram averaging analysis of apical bound RNAP and dCas9
(**A**) Two orthogonal views of the sub-tomogram averaged RNAP map fitted with PDB structure 6ALH. The total number of RNAPs (n =1781) selected from the tomogram yielded an averaged map resolution of 14.1 Å (top panel), while using only the plasmid-bound RNAP population (n =232) result in a resolution of 17.7 Å (bottom panel). (**B**) Superimposition of TIC DNA (4YLN, 6CA0, 6JBQ, and 6N60, labeled with gold color) with TEC DNA (6ALH, labeled with red color). Both complexes utilize a short linear DNA template. (**C**) Superimposition of the consensus DNA trajectory of the apically stalled TEC with –sc. DNA template onto B. The downstream and upstream DNA were color in green and yellow, respectively. (**D**) Sub-tomogram averaged dCas9 map fitted with PDB structure 6O0X. A total of 875 dCas9 molecules selected from the tomogram yielded an average map resolution of 17.6 Å (top panel), while the plasmid-bound subset (n = 116) achieved 18.9 Å (bottom panel). Resolutions were estimated by measuring the Fourier shell correlation (FSC) between two independently determined half-maps at 0.143.

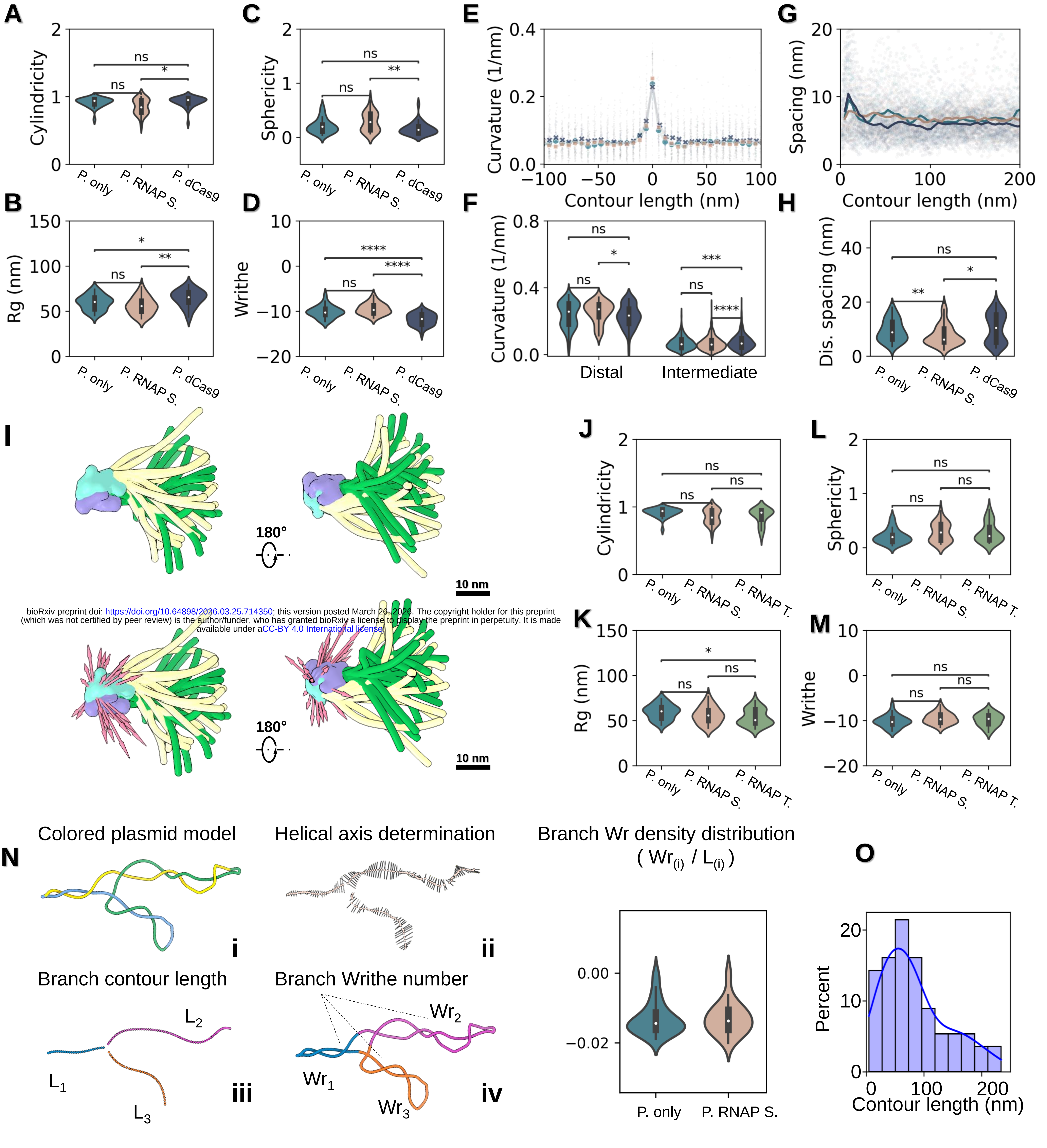

bioRxiv preprint doi: https://doi.org/10.64898/2026.03.25.714350; this version posted March 26, 2026. The copyright holder for this preprint (which was not certified by peer review) is the author/funder, who has granted bioRxiv a license to display the preprint in perpetuity. It is made available under aCC-BY 4.0 International license.

Figure S4: Quantification of plasmid geometry upon RNAP/dCas9 binding

(**A-D**) Statistical analysis of pUC19-T7A1U plasmid (P.) cylindricity, radius of gyration, sphericity, and writhe number with binding enzymes, respectively, in the presence of stalled RNAP (RNAP S.) or dCas9. (**E**) Tracing of plasmid curvature near apices with traces aligned to the apices (index zero). (**F**) Statistical comparison of plasmid apical and intermediate region curvature distribution. (**G**) Traces of the plectoneme width (DNA spacing) starting from each apex (index zero). (**H**) Statistical comparison of the plectoneme width distribution. (**I**) Overlay of apical bound RNAPs (top) and labeling their RNA protrusion directions in pink (bottom). (**J-M**) Statistical analyses mirror those in (**A-D**) for plasmids with transcribed RNAP (RNAP T.). (**N**) Schematics showing the segmentation of plectoneme branches (left four panels) followed by branch Writhe density calculation (right panel): (i) DNA colorization based apices, (ii) plectoneme central axis determination, (iii) branch segmentation and contour length calculation, and (iv) branch Writhe number calculation. The writhe density was calculated by dividing the writhe number of each branch segmentation by the contour length of the segment. (**O**) Plectoneme contour length distribution for TECs. Statistics are calculated using a Mann-Whitney test, where *p < 0.05, **p < 0.01, ***p < 0.001, ****p < 0.0001; and ns, not significant.

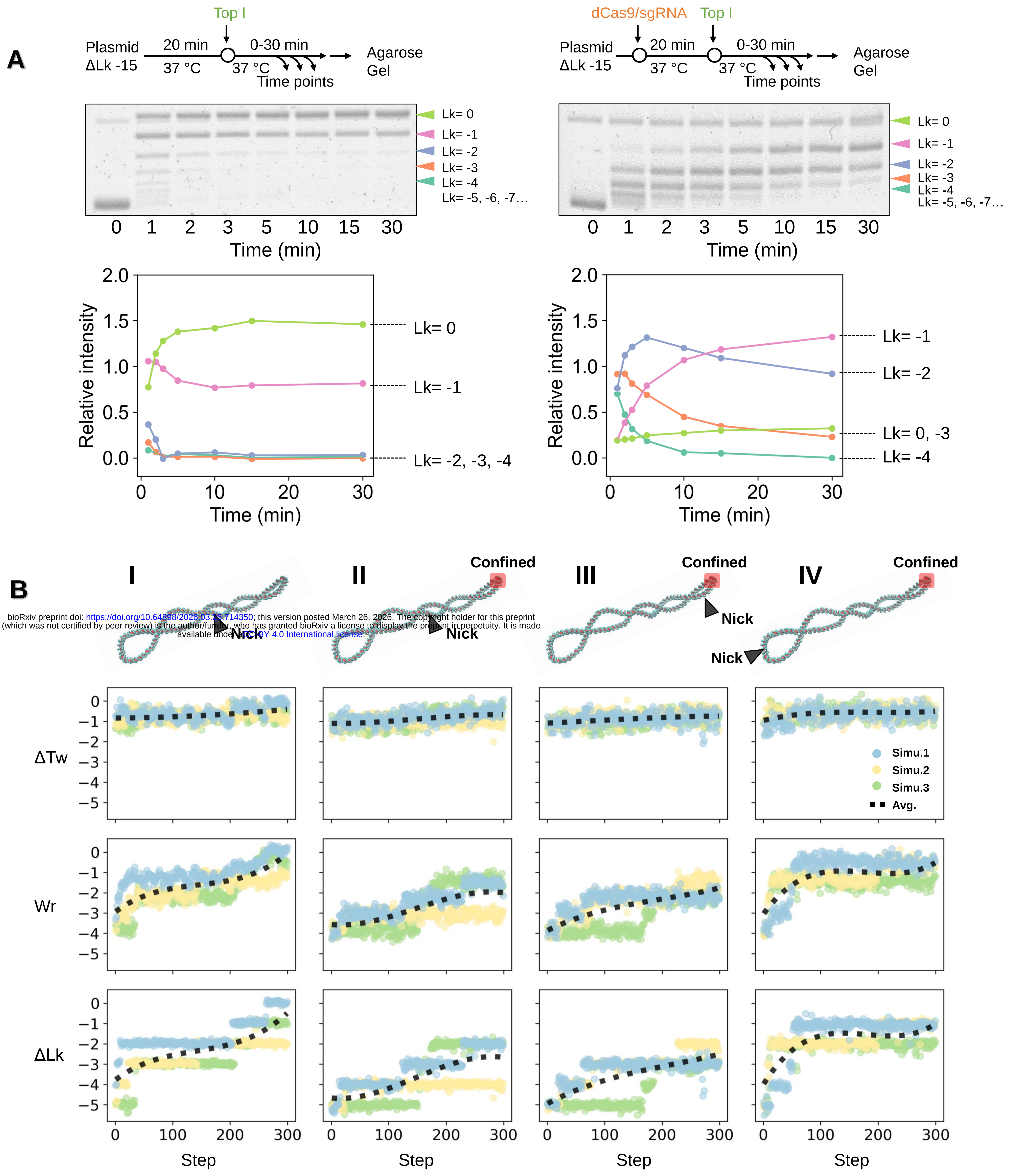

bioRxiv preprint doi: https://doi.org/10.64898/2026.03.25.714350; this version posted March 26, 2026. The copyright holder for this preprint (which was not certified by peer review) is the author/funder, who has granted bioRxiv a license to display the preprint in perpetuity. It is made available under aCC-BY 4.0 International license.

Figure S5: Apical binding of dCas9 hinders DNA rotation

(**A**) DNA supercoiling relaxation assay in the absence (left panel) and presence (right panel) of dCas9 rotation block. Electrophoresis gel shows the change in pUC19-T7A1U plasmid linking number over time after the addition of TopI (top panel). The linking numbers of the plasmid population from 0 to 4 are labeled with various colors. Quantification of the gel band intensity at the corresponding linking number is presented with the same color code (bottom panel). (**B**) Coarse-grained MD simulation of 525-bp circular plasmid relaxation (ΔLk = -5, row 1) with initial structures featuring various nick locations. Simulations were performed without apical restriction (model I) and with apical restriction (model II,III,IV). Topological evolutions (ΔTw, Wr, and ΔLk) are presented in rows 2-4, respectively, for each corresponding simulation. Each simulation was run in triplicate with their average marked by black dashed line.

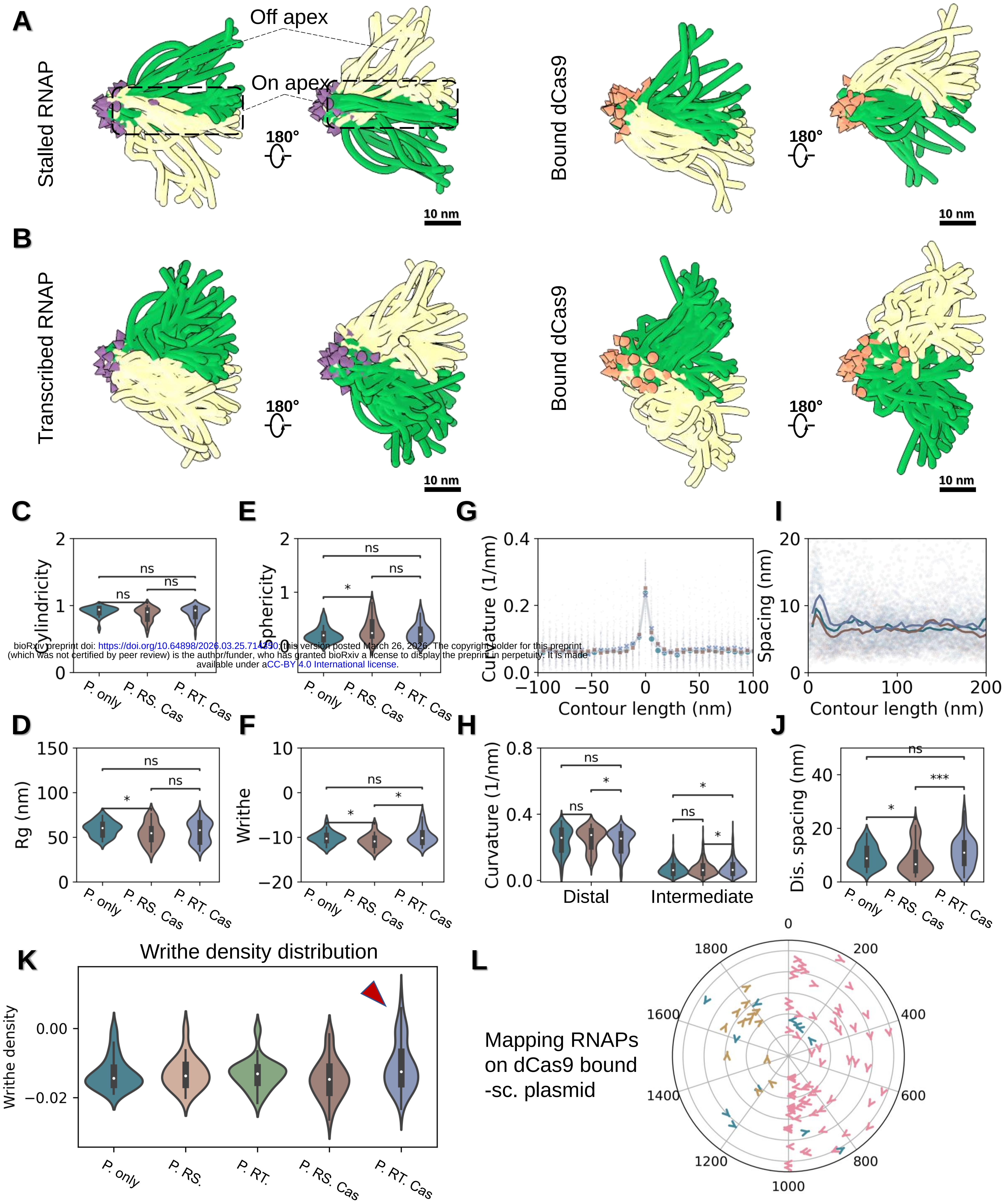

bioRxiv preprint doi: https://doi.org/10.64898/2026.03.25.714290; this version posted March 26, 2026. The copyright holder for this preprint (which was not certified by peer review) is the author/funder, who has granted bioRxiv a license to display the preprint in perpetuity. It is made available under aCC-BY 4.0 International license.

Figure S6: Quantification of plasmid morphology in the presence of both RNAP and dCas9 binding

(**A**) Two orthogonal views of the superimposed apical DNA segments of the plasmid, illustrating the dynamics of stalled-RNAP (purple vectors, left panel) and dCas9 (orange vectors, right panel) in their co-presence on the plasmid. (**B**) Two orthogonal views of the superimposed apical DNA segments of the plasmid, illustrating the dynamics of transcribed-RNAP (purple vectors) and dCas9 (orange vectors) in their co-presence on the plasmid. (**C-F**) Statistical analysis of plasmid (P.) cylindricity, radius of gyration, sphericity, and writhe number with binding enzymes, respectively, in the presence of transcribed/stalled RNAP (RT./RS.) and dCas9 (Cas). Statistics are calculated using a Mann-Whitney test, where *p < 0.05, **p < 0.01, ***p < 0.001, ****p < 0.0001; and ns, not significant. (**G**) Tracing of plasmid curvature near apices with traces aligned to the apices (index zero). (**H**) Statistical comparison of plasmid apical and intermediate region curvature distribution. (**I**) Traces of the plectoneme width (DNA spacing) starting from each apex (index zero). (**J**) Statistical comparison of the plectoneme width distribution. (**K**) Quantification of writhe density in plasmid branches. (**L**) Mapping all bound RNAPs on pUC19-T7A1U templates using dCas9 as a fiducial. RNAP on the transcriptional region, oriented downstream, is marked in pink; RNAP on the non-transcriptional region or pointing upstream, is colored in tan and blue, respectively.

bioRxiv preprint doi: https://doi.org/10.64898/2026.03.25.714350; this version posted March 26, 2026. The copyright holder for this preprint (which was not certified by peer review) is the author/funder, who has granted bioRxiv a license to display the preprint in perpetuity. It is made available under aCC-BY 4.0 International license.

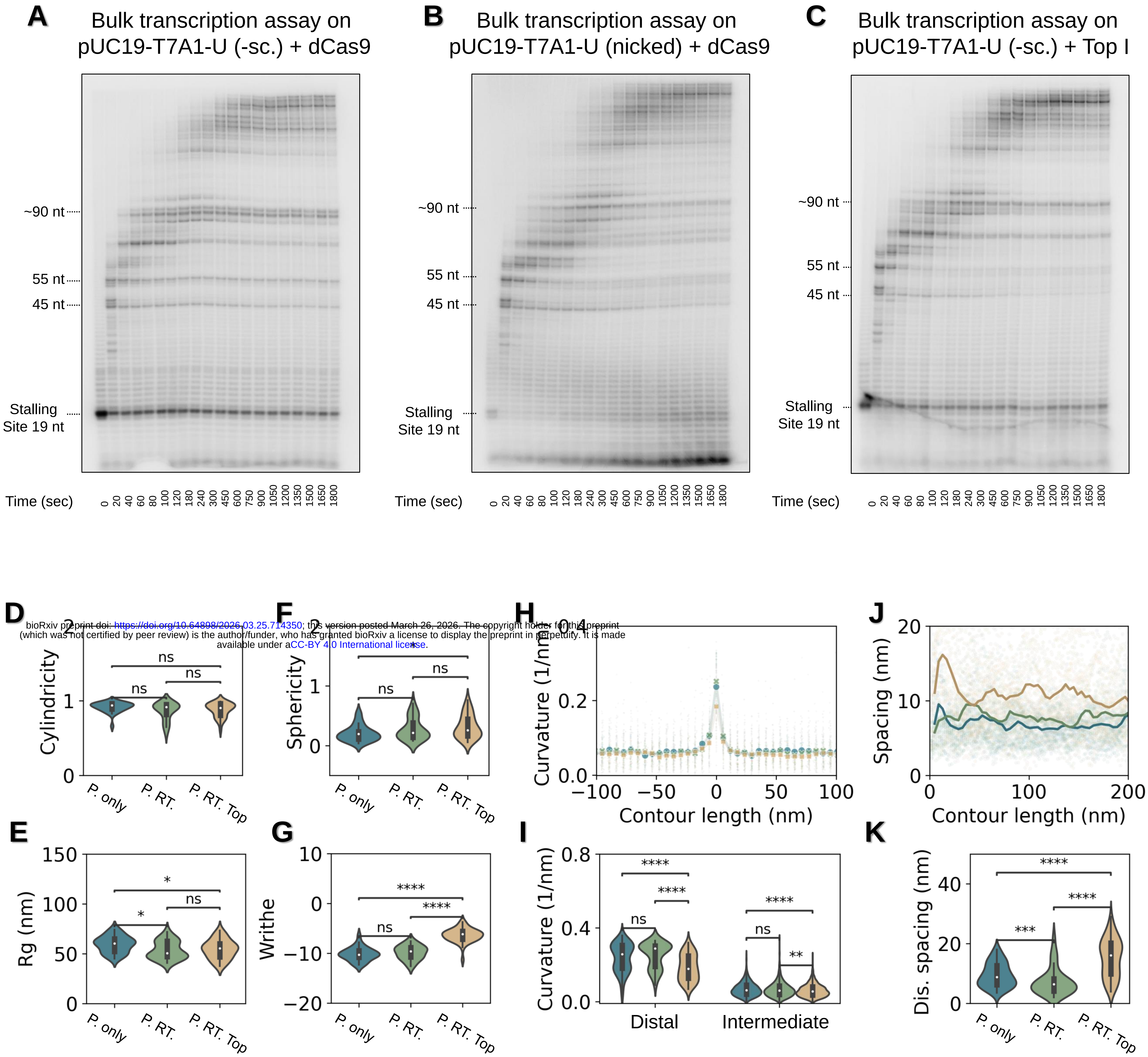

Figure S7: Top I and RNAP coupling slows supercoiling relaxation
(**A-C**) Electrophoresis assay of RNAP transcription on –sc. and nicked templates in the presence of dCas9 and on –sc. template in the presence of TopI over time, respectively. (**D-G**) Statistical analysis of plasmid (P.) cylindricity, radius of gyration, sphericity, and writhe number with binding enzymes, respectively, in the presence of transcribed RNAP (RT.) and Topoisomerase I (Top). (**H**) Tracing of plasmid curvature near apices with traces aligned to the apices (index zero). (**I**) Statistical comparison of plasmid apical and intermediate region curvature distribution. (**J**) Traces of the plectoneme width (DNA spacing) starting from each apex (index zero). (**K**) Statistical comparison of the plectoneme width distribution. Statistics are calculated using a Mann-Whitney test, where *p < 0.05, **p < 0.01, ***p < 0.001, ****p < 0.0001; and ns, not significant.
