# Supplementary material for: Apical Localization of RNA Polymerases Modulate Transcription Dynamics and Supercoiling Domains Revealed by Cryo-ET
Source: bioRxiv. 2026 Mar 26:2026.03.25.714350. Preprint. [Version 1] doi: 10.64898/2026.03.25.714350 (PMC13041910; doi:10.64898/2026.03.25.714350)
Supplement: Supplementary file 2 [file media-1.pdf]

Supplementary Particle Gallery for

Protein Binding to Apices of Plectonemic DNA Modulates Transcription Dynamics and Supercoiling Revealed by Cryo-ET

Meng Zhang[1,2,7]*, Cristhian Cañari-Chumpitaz[1,3,4,7]*, Jianfang Liu[2], Bibiana Onoa[1,5], Sinead de Cleir[6], Enze Cheng[8], Katherinne I. Requejo[1,7], Carlos Bustamante[1,6,7,8,9,10,11,12]**

[1] California Institute for Quantitative Biosciences, University of California, Berkeley, USA
[2] The Molecular Foundry, Lawrence Berkeley National Laboratory, Berkeley, USA
[3] Department of Chemistry, University of California Berkeley, Berkeley, CA, USA
[4] Department of Biology, Stanford University, Stanford, CA USA
[5] Innovative Genomics Institute, University of California Berkeley, Berkeley, CA, USA
[6] Department of Molecular and Cell Biology, University of California, Berkeley, USA
[7] Howard Hughes Medical Institute, University of California, Berkeley, USA
[8] Department of Physics, University of California, Berkeley, USA
[9] Department of Chemistry, University of California, Berkeley, USA
[10] Molecular Biophysics and Integrative Bioimaging Division, Lawrence Berkeley National Laboratory, USA
[11] Kavli Energy Nanoscience Institute, University of California, Berkeley, USA
[12]Jason Choy laboratory of single molecule biophysics, University of California, Berkeley, USA
* These authors contributed equally
** Correspondence should be addressed to: C. B. (carlosb@berkeley.edu)

Particle information table for all 3D reconstructed maps ………...2
Detailed 3D reconstruction steps and FSC analysis of plasmid particle in low salt condition ……...5
Detailed 3D reconstruction steps and FSC analysis of plasmid particle in near physiological salt condition ……………………………………………………………………………………………….22
Detailed 3D reconstruction steps and FSC analysis of plasmid particle in near physiological salt condition and in the presence of stalled RNAP……………………………………………………37
Detailed 3D reconstruction steps and FSC analysis of plasmid particle in near physiological salt condition and in the presence of dCas9………………………………………………………….51
Detailed 3D reconstruction steps and FSC analysis of plasmid particle in near physiological salt condition and in the presence of transcribed RNAP…………………………………………………68
Detailed 3D reconstruction steps and FSC analysis of plasmid particle in near physiological salt condition and in the co-presence of stalled RNAP and dCas9………………………………………..79
Detailed 3D reconstruction steps and FSC analysis of plasmid particle in near physiological salt condition and in the co-presence of transcribed RNAP and dCas9…………………………………..96
Detailed 3D reconstruction steps and FSC analysis of plasmid particle in near physiological salt condition and in the co-presence of transcribed RNAP and TopI……………………………………118

Par. id #	Name abbr.	Bound RNAP #	Bound dCas9 #	Salt condition	Tilt range (°)	Tilt step (°)	TEM /Cam.	Mag.	Apix (Å²)	Total dose (e-/Å²)	EMDB map ID #	Montage position (row, col)	Resolution (FSC=0.143) Even vs. Odd (Å)	Resolution (FSC=0.5) Map vs. Model (Å)
1	P. only LS	0	0	5 mM K+	-51 to 51	3	G3/K3	53K	1.67	183	EMD-47843	1,1	33	38.7
2	P. only LS	0	0	5 mM K+	-51 to 51	3	G3/K3	53K	1.67	183	EMD-47843	1,2	30	36
3	P. only LS	0	0	5 mM K+	-51 to 51	3	G3/K3	53K	1.67	183	EMD-47843	1,3	34.1	38.6
4	P. only LS	0	0	5 mM K+	-51 to 51	3	G3/K3	53K	1.67	183	EMD-47843	1,4	28.7	40.9
5	P. only LS	0	0	5 mM K+	-51 to 51	3	G3/K3	53K	1.67	183	EMD-47843	1,5	28.2	40
6	P. only LS	0	0	5 mM K+	-51 to 51	3	G3/K3	53K	1.67	183	EMD-47843	1,6	29.7	38.9
7	P. only LS	0	0	5 mM K+	-51 to 51	3	G3/K3	53K	1.67	183	EMD-47843	1,7	29.3	41.5
8	P. only LS	0	0	5 mM K+	-51 to 51	3	G3/K3	53K	1.67	183	EMD-47843	2,1	31.2	32.1
9	P. only LS	0	0	5 mM K+	-51 to 51	3	G3/K3	53K	1.67	183	EMD-47843	2,2	30.8	38.5
10	P. only LS	0	0	5 mM K+	-51 to 51	3	G3/K3	53K	1.67	183	EMD-47843	2,3	31	42.9
11	P. only LS	0	0	5 mM K+	-51 to 51	3	G3/K3	53K	1.67	183	EMD-47843	2,4	29.2	39.6
12	P. only LS	0	0	5 mM K+	-51 to 51	3	G3/K3	53K	1.67	183	EMD-47843	2,5	30.4	37.6
13	P. only LS	0	0	5 mM K+	-51 to 51	3	G3/K3	53K	1.67	183	EMD-47843	2,6	31.4	32.4
14	P. only LS	0	0	5 mM K+	-51 to 51	3	G3/K3	53K	1.67	183	EMD-47843	2,7	29.3	38.4
15	P. only LS	0	0	5 mM K+	-51 to 51	3	G3/K3	53K	1.67	183	EMD-47843	3,1	30.6	42.7
16	P. only LS	0	0	5 mM K+	-51 to 51	3	G3/K3	53K	1.67	183	EMD-47843	3,2	31.3	47.6
17	P. only LS	0	0	5 mM K+	-51 to 51	3	G3/K3	53K	1.67	183	EMD-47843	3,3	29.2	39.8
18	P. only LS	0	0	5 mM K+	-51 to 51	3	G3/K3	53K	1.67	183	EMD-47843	3,4	27.9	35
19	P. only LS	0	0	5 mM K+	-51 to 51	3	G3/K3	53K	1.67	183	EMD-47843	3,5	25.7	35.3
20	P. only LS	0	0	5 mM K+	-51 to 51	3	G3/K3	53K	1.67	183	EMD-47843	3,6	24.7	33.6
21	P. only LS	0	0	5 mM K+	-51 to 51	3	G3/K3	53K	1.67	183	EMD-47843	3,7	31.4	39.3
22	P. only LS	0	0	5 mM K+	-51 to 51	3	G3/K3	53K	1.67	183	EMD-47843	4,1	29.8	38.9
23	P. only LS	0	0	5 mM K+	-51 to 51	3	G3/K3	53K	1.67	183	EMD-47843	4,2	31.2	40
24	P. only LS	0	0	5 mM K+	-51 to 51	3	G3/K3	53K	1.67	183	EMD-47843	4,3	31.9	43.6
25	P. only LS	0	0	5 mM K+	-51 to 51	3	G3/K3	53K	1.67	183	EMD-47843	4,4	28.2	39.1
26	P. only LS	0	0	5 mM K+	-51 to 51	3	G3/K3	53K	1.67	183	EMD-47843	4,5	31.2	33
27	P. only LS	0	0	5 mM K+	-51 to 51	3	G3/K3	53K	1.67	183	EMD-47843	4,6	27.9	37
28	P. only LS	0	0	5 mM K+	-51 to 51	3	G3/K3	53K	1.67	183	EMD-47843	4,7	29.9	38
29	P. only LS	0	0	5 mM K+	-51 to 51	3	G3/K3	53K	1.67	183	EMD-47843	5,1	29	33.4
30	P. only LS	0	0	5 mM K+	-51 to 51	3	G3/K3	53K	1.67	183	EMD-47843	5,2	29	36.2
31	P. only LS	0	0	5 mM K+	-51 to 51	3	G3/K3	53K	1.67	183	EMD-47843	5,3	33.6	38.2
32	P. only LS	0	0	5 mM K+	-51 to 51	3	G3/K3	53K	1.67	183	EMD-47843	5,4	27.1	32.9
33	P. only LS	0	0	5 mM K+	-51 to 51	3	G3/K3	53K	1.67	183	EMD-47843	5,5	24.7	35.7
34	P. only LS	0	0	5 mM K+	-51 to 51	3	G3/K3	53K	1.67	183	EMD-47843	5,6	28.1	34.6
35	P. only LS	0	0	5 mM K+	-51 to 51	3	G3/K3	53K	1.67	183	EMD-47843	5,7	24.6	37.1
36	P. only PS	0	0	40 mM K+, 5 mM Mg2+	-51 to 51	3	G3/K3	53K	1.67	183	EMD-47844	1,1	27.6	34.2
37	P. only PS	0	0	40 mM K+, 5 mM Mg2+	-51 to 51	3	G3/K3	53K	1.67	183	EMD-47844	1,2	26.9	38.1
38	P. only PS	0	0	40 mM K+, 5 mM Mg2+	-51 to 51	3	G3/K3	53K	1.67	183	EMD-47844	1,3	27.8	36
39	P. only PS	0	0	40 mM K+, 5 mM Mg2+	-51 to 51	3	G3/K3	53K	1.67	183	EMD-47844	1,4	27.3	34.1
40	P. only PS	0	0	40 mM K+, 5 mM Mg2+	-51 to 51	3	G3/K3	53K	1.67	183	EMD-47844	1,5	29.5	36.6
41	P. only PS	0	0	40 mM K+, 5 mM Mg2+	-51 to 51	3	G3/K3	53K	1.67	183	EMD-47844	1,6	27	32.8
42	P. only PS	0	0	40 mM K+, 5 mM Mg2+	-51 to 51	3	G3/K3	53K	1.67	183	EMD-47844	2,1	26.3	38
43	P. only PS	0	0	40 mM K+, 5 mM Mg2+	-51 to 51	3	G3/K3	53K	1.67	183	EMD-47844	2,2	29	37.2
44	P. only PS	0	0	40 mM K+, 5 mM Mg2+	-51 to 51	3	G3/K3	53K	1.67	183	EMD-47844	2,3	26.3	42.8
45	P. only PS	0	0	40 mM K+, 5 mM Mg2+	-51 to 51	3	G3/K3	53K	1.67	183	EMD-47844	2,4	28.9	31.5
46	P. only PS	0	0	40 mM K+, 5 mM Mg2+	-51 to 51	3	G3/K3	53K	1.67	183	EMD-47844	2,5	27.8	35.1
47	P. only PS	0	0	40 mM K+, 5 mM Mg2+	-51 to 51	3	G3/K3	53K	1.67	183	EMD-47844	2,6	26.3	36.5
48	P. only PS	0	0	40 mM K+, 5 mM Mg2+	-51 to 51	3	G3/K3	53K	1.67	183	EMD-47844	3,1	28.1	32.3
49	P. only PS	0	0	40 mM K+, 5 mM Mg2+	-51 to 51	3	G3/K3	53K	1.67	183	EMD-47844	3,2	27.2	32.1
50	P. only PS	0	0	40 mM K+, 5 mM Mg2+	-51 to 51	3	G3/K3	53K	1.67	183	EMD-47844	3,3	29.7	35.6
51	P. only PS	0	0	40 mM K+, 5 mM Mg2+	-51 to 51	3	G3/K3	53K	1.67	183	EMD-47844	3,4	27.4	36.9
52	P. only PS	0	0	40 mM K+, 5 mM Mg2+	-51 to 51	3	G3/K3	53K	1.67	183	EMD-47844	3,5	27.3	34.6
53	P. only PS	0	0	40 mM K+, 5 mM Mg2+	-51 to 51	3	G3/K3	53K	1.67	183	EMD-47844	3,6	29.6	41.2
54	P. only PS	0	0	40 mM K+, 5 mM Mg2+	-51 to 51	3	G3/K3	53K	1.67	183	EMD-47844	4,1	27.3	31.7
55	P. only PS	0	0	40 mM K+, 5 mM Mg2+	-51 to 51	3	G3/K3	53K	1.67	183	EMD-47844	4,2	26.7	39.2
56	P. only PS	0	0	40 mM K+, 5 mM Mg2+	-51 to 51	3	G3/K3	53K	1.67	183	EMD-47844	4,3	26.7	42
57	P. only PS	0	0	40 mM K+, 5 mM Mg2+	-51 to 51	3	G3/K3	53K	1.67	183	EMD-47844	4,4	29.7	45.6
58	P. only PS	0	0	40 mM K+, 5 mM Mg2+	-51 to 51	3	G3/K3	53K	1.67	183	EMD-47844	4,5	26.9	42.4
59	P. only PS	0	0	40 mM K+, 5 mM Mg2+	-51 to 51	3	G3/K3	53K	1.67	183	EMD-47844	4,6	27	38.9
60	P. only PS	0	0	40 mM K+, 5 mM Mg2+	-51 to 51	3	G3/K3	53K	1.67	183	EMD-47844	5,1	28.8	41.1
61	P. only PS	0	0	40 mM K+, 5 mM Mg2+	-51 to 51	3	G3/K3	53K	1.67	183	EMD-47844	5,2	27.4	42.1
62	P. only PS	0	0	40 mM K+, 5 mM Mg2+	-51 to 51	3	G3/K3	53K	1.67	183	EMD-47844	5,3	28.1	35.4
63	P. only PS	0	0	40 mM K+, 5 mM Mg2+	-51 to 51	3	G3/K3	53K	1.67	183	EMD-47844	5,4	26.7	53.5
64	P. only PS	0	0	40 mM K+, 5 mM Mg2+	-51 to 51	3	G3/K3	53K	1.67	183	EMD-47844	5,5	28.2	47.7
65	P. RNAP S.	1	0	40 mM K+, 5 mM Mg2+	-51 to 51	3	G3/K3	53K	1.67	183	EMD-47847	1,1	27	44.7
66	P. RNAP S.	1	0	40 mM K+, 5 mM Mg2+	-51 to 51	3	G3/K3	53K	1.67	183	EMD-47847	1,2	26.9	35.6
67	P. RNAP S.	1	0	40 mM K+, 5 mM Mg2+	-51 to 51	3	G3/K3	53K	1.67	183	EMD-47847	1,3	28.7	45.3
68	P. RNAP S.	1	0	40 mM K+, 5 mM Mg2+	-51 to 51	3	G3/K3	53K	1.67	183	EMD-47847	1,4	28.3	48.3
69	P. RNAP S.	1	0	40 mM K+, 5 mM Mg2+	-51 to 51	3	G3/K3	53K	1.67	183	EMD-47847	1,5	25.9	51.5
70	P. RNAP S.	1	0	40 mM K+, 5 mM Mg2+	-51 to 51	3	G3/K3	53K	1.67	183	EMD-47847	1,6	28.4	44.8
71	P. RNAP S.	1	0	40 mM K+, 5 mM Mg2+	-51 to 51	3	G3/K3	53K	1.67	183	EMD-47847	2,1	28.2	42.8
72	P. RNAP S.	1	0	40 mM K+, 5 mM Mg2+	-51 to 51	3	G3/K3	53K	1.67	183	EMD-47847	2,2	27.3	40.1
73	P. RNAP S.	2	0	40 mM K+, 5 mM Mg2+	-51 to 51	3	G3/K3	53K	1.67	183	EMD-47847	2,3	25.3	41
74	P. RNAP S.	1	0	40 mM K+, 5 mM Mg2+	-51 to 51	3	G3/K3	53K	1.67	183	EMD-47847	2,4	28.9	53.2
75	P. RNAP S.	1	0	40 mM K+, 5 mM Mg2+	-51 to 51	3	G3/K3	53K	1.67	183	EMD-47847	2,5	28	45.3
76	P. RNAP S.	1	0	40 mM K+, 5 mM Mg2+	-51 to 51	3	G3/K3	53K	1.67	183	EMD-47847	2,6	27.4	35.6
77	P. RNAP S.	1	0	40 mM K+, 5 mM Mg2+	-51 to 51	3	G3/K3	53K	1.67	183	EMD-47847	3,1	27.2	45.3
78	P. RNAP S.	1	0	40 mM K+, 5 mM Mg2+	-51 to 51	3	G3/K3	53K	1.67	183	EMD-47847	3,2	30.7	46.3
79	P. RNAP S.	1	0	40 mM K+, 5 mM Mg2+	-51 to 51	3	G3/K3	53K	1.67	183	EMD-47847	3,3	27.7	41.2
80	P. RNAP S.	1	0	40 mM K+, 5 mM Mg2+	-51 to 51	3	G3/K3	53K	1.67	183	EMD-47847	3,4	28.7	41.8
81	P. RNAP S.	1	0	40 mM K+, 5 mM Mg2+	-51 to 51	3	G3/K3	53K	1.67	183	EMD-47847	3,5	29.1	54.3
82	P. RNAP S.	1	0	40 mM K+, 5 mM Mg2+	-51 to 51	3	G3/K3	53K	1.67	183	EMD-47847	3,6	27	41
83	P. RNAP S.	1	0	40 mM K+, 5 mM Mg2+	-51 to 51	3	G3/K3	53K	1.67	183	EMD-47847	4,1	30.2	46.8
84	P. RNAP S.	1	0	40 mM K+, 5 mM Mg2+	-51 to 51	3	G3/K3	53K	1.67	183	EMD-47847	4,2	29.5	37.8
85	P. RNAP S.	1	0	40 mM K+, 5 mM Mg2+	-51 to 51	3	G3/K3	53K	1.67	183	EMD-47847	4,3	30.9	38.5
86	P. RNAP S.	1	0	40 mM K+, 5 mM Mg2+	-51 to 51	3	G3/K3	53K	1.67	183	EMD-47847	4,4	26.6	45.5
87	P. RNAP S.	1	0	40 mM K+, 5 mM Mg2+	-51 to 51	3	G3/K3	53K	1.67	183	EMD-47847	4,5	26.5	48.3
88	P. RNAP S.	1	0	40 mM K+, 5 mM Mg2+	-51 to 51	3	G3/K3	53K	1.67	183	EMD-47847	4,6	25.6	43

Par. id	Name abbr.	Bound RNAP	Bound dCas9	Salt condition	Tilt range	Tilt step	TEM /Cam.	Mag.	Apix	Total dose	EMDB map ID	Montage position	Resolution (FSC=0.143)	Resolution (FSC=0.5)
#		#	#		(°)	(°)			(Å²)	(e-/Å²)	#	(row, col)	Even vs. Odd (Å)	Map vs. Model (Å)
89	P. RNAP S.	1	0	40 mM K+, 5 mM Mg2+	-51 to 51	3	G3/K3	53K	1.67	183	EMD-47847	5,1	28.1	59.7
90	P. RNAP S.	1	0	40 mM K+, 5 mM Mg2+	-51 to 51	3	G3/K3	53K	1.67	183	EMD-47847	5,2	28.8	43.8
91	P. RNAP S.	1	0	40 mM K+, 5 mM Mg2+	-51 to 51	3	G3/K3	53K	1.67	183	EMD-47847	5,3	29.8	37.7
92	P. dCas9	0	1	40 mM K+, 5 mM Mg2+	-51 to 51	3	G3/K3	53K	1.67	183	EMD-47849	1,1	27.1	47.2
93	P. dCas9	0	1	40 mM K+, 5 mM Mg2+	-51 to 51	3	G3/K3	53K	1.67	183	EMD-47849	1,2	26.1	39.6
94	P. dCas9	0	1	40 mM K+, 5 mM Mg2+	-51 to 51	3	G3/K3	53K	1.67	183	EMD-47849	1,3	25.7	42.7
95	P. dCas9	0	1	40 mM K+, 5 mM Mg2+	-51 to 51	3	G3/K3	53K	1.67	183	EMD-47849	1,4	27.3	36.7
96	P. dCas9	0	2	40 mM K+, 5 mM Mg2+	-51 to 51	3	G3/K3	53K	1.67	183	EMD-47849	1,5	28.1	31.7
97	P. dCas9	0	1	40 mM K+, 5 mM Mg2+	-51 to 51	3	G3/K3	53K	1.67	183	EMD-47849	1,6	25.5	33.1
98	P. dCas9	0	1	40 mM K+, 5 mM Mg2+	-51 to 51	3	G3/K3	53K	1.67	183	EMD-47849	1,7	26.3	40.5
99	P. dCas9	0	1	40 mM K+, 5 mM Mg2+	-51 to 51	3	G3/K3	53K	1.67	183	EMD-47849	2,1	28.5	30.3
100	P. dCas9	0	1	40 mM K+, 5 mM Mg2+	-51 to 51	3	G3/K3	53K	1.67	183	EMD-47849	2,2	26.6	41.4
101	P. dCas9	0	1	40 mM K+, 5 mM Mg2+	-51 to 51	3	G3/K3	53K	1.67	183	EMD-47849	2,3	28	32.8
102	P. dCas9	0	1	40 mM K+, 5 mM Mg2+	-51 to 51	3	G3/K3	53K	1.67	183	EMD-47849	2,4	27.2	31.6
103	P. dCas9	0	1	40 mM K+, 5 mM Mg2+	-51 to 51	3	G3/K3	53K	1.67	183	EMD-47849	2,5	28.1	30.8
104	P. dCas9	0	1	40 mM K+, 5 mM Mg2+	-51 to 51	3	G3/K3	53K	1.67	183	EMD-47849	2,6	27.9	32.9
105	P. dCas9	0	1	40 mM K+, 5 mM Mg2+	-51 to 51	3	G3/K3	53K	1.67	183	EMD-47849	2,7	28.6	34.5
106	P. dCas9	0	1	40 mM K+, 5 mM Mg2+	-51 to 51	3	G3/K3	53K	1.67	183	EMD-47849	3,1	26	32.1
107	P. dCas9	0	2	40 mM K+, 5 mM Mg2+	-51 to 51	3	G3/K3	53K	1.67	183	EMD-47849	3,2	26.2	36.2
108	P. dCas9	0	1	40 mM K+, 5 mM Mg2+	-51 to 51	3	G3/K3	53K	1.67	183	EMD-47849	3,3	27.1	31
109	P. dCas9	0	1	40 mM K+, 5 mM Mg2+	-51 to 51	3	G3/K3	53K	1.67	183	EMD-47849	3,4	27.3	30.6
110	P. dCas9	0	1	40 mM K+, 5 mM Mg2+	-51 to 51	3	G3/K3	53K	1.67	183	EMD-47849	3,5	27.7	32.6
111	P. dCas9	0	1	40 mM K+, 5 mM Mg2+	-51 to 51	3	G3/K3	53K	1.67	183	EMD-47849	3,6	27.8	35.2
112	P. dCas9	0	1	40 mM K+, 5 mM Mg2+	-51 to 51	3	G3/K3	53K	1.67	183	EMD-47849	3,7	28.2	30.9
113	P. dCas9	0	1	40 mM K+, 5 mM Mg2+	-51 to 51	3	G3/K3	53K	1.67	183	EMD-47849	4,1	28.4	31.7
114	P. dCas9	0	1	40 mM K+, 5 mM Mg2+	-51 to 51	3	G3/K3	53K	1.67	183	EMD-47849	4,2	28.3	30.2
115	P. dCas9	0	1	40 mM K+, 5 mM Mg2+	-51 to 51	3	G3/K3	53K	1.67	183	EMD-47849	4,3	27.7	29
116	P. dCas9	0	1	40 mM K+, 5 mM Mg2+	-51 to 51	3	G3/K3	53K	1.67	183	EMD-47849	4,4	27.8	28.8
117	P. dCas9	0	1	40 mM K+, 5 mM Mg2+	-51 to 51	3	G3/K3	53K	1.67	183	EMD-47849	4,5	26.6	31.6
118	P. dCas9	0	1	40 mM K+, 5 mM Mg2+	-51 to 51	3	G3/K3	53K	1.67	183	EMD-47849	4,6	26.9	30.9
119	P. dCas9	0	1	40 mM K+, 5 mM Mg2+	-51 to 51	3	G3/K3	53K	1.67	183	EMD-47849	4,7	26.7	33
120	P. dCas9	0	1	40 mM K+, 5 mM Mg2+	-51 to 51	3	G3/K3	53K	1.67	183	EMD-47849	5,1	28.8	30.8
121	P. dCas9	0	1	40 mM K+, 5 mM Mg2+	-51 to 51	3	G3/K3	53K	1.67	183	EMD-47849	5,2	26.2	36.1
122	P. dCas9	0	1	40 mM K+, 5 mM Mg2+	-51 to 51	3	G3/K3	53K	1.67	183	EMD-47849	5,3	25.8	34.6
123	P. dCas9	0	1	40 mM K+, 5 mM Mg2+	-51 to 51	3	G3/K3	53K	1.67	183	EMD-47849	5,4	27.1	33
124	P. dCas9	0	1	40 mM K+, 5 mM Mg2+	-51 to 51	3	G3/K3	53K	1.67	183	EMD-47849	5,5	26.2	32.4
125	P. dCas9	0	1	40 mM K+, 5 mM Mg2+	-51 to 51	3	G3/K3	53K	1.67	183	EMD-47849	5,6	25.7	30.9
126	P. RNAP T.	1	0	40 mM K+, 5 mM Mg2+	-51 to 51	3	G3/K3	53K	1.67	183	EMD-47850	1,1	27.7	37.2
127	P. RNAP T.	1	0	40 mM K+, 5 mM Mg2+	-51 to 51	3	G3/K3	53K	1.67	183	EMD-47850	1,2	28.6	33.4
128	P. RNAP T.	1	0	40 mM K+, 5 mM Mg2+	-51 to 51	3	G3/K3	53K	1.67	183	EMD-47850	1,3	28.3	34.6
129	P. RNAP T.	1	0	40 mM K+, 5 mM Mg2+	-51 to 51	3	G3/K3	53K	1.67	183	EMD-47850	1,4	28.1	40.9
130	P. RNAP T.	1	0	40 mM K+, 5 mM Mg2+	-51 to 51	3	G3/K3	53K	1.67	183	EMD-47850	1,5	27.5	44.3
131	P. RNAP T.	1	0	40 mM K+, 5 mM Mg2+	-51 to 51	3	G3/K3	53K	1.67	183	EMD-47850	2,1	25.5	32.9
132	P. RNAP T.	1	0	40 mM K+, 5 mM Mg2+	-51 to 51	3	G3/K3	53K	1.67	183	EMD-47850	2,2	26.9	34.6
133	P. RNAP T.	1	0	40 mM K+, 5 mM Mg2+	-51 to 51	3	G3/K3	53K	1.67	183	EMD-47850	2,3	27.7	35.1
134	P. RNAP T.	1	0	40 mM K+, 5 mM Mg2+	-51 to 51	3	G3/K3	53K	1.67	183	EMD-47850	2,4	27.9	34.7
135	P. RNAP T.	2	0	40 mM K+, 5 mM Mg2+	-51 to 51	3	G3/K3	53K	1.67	183	EMD-47850	2,5	27.7	35.7
136	P. RNAP T.	1	0	40 mM K+, 5 mM Mg2+	-51 to 51	3	G3/K3	53K	1.67	183	EMD-47850	3,1	26.5	32
137	P. RNAP T.	1	0	40 mM K+, 5 mM Mg2+	-51 to 51	3	G3/K3	53K	1.67	183	EMD-47850	3,2	28.7	31.4
138	P. RNAP T.	1	0	40 mM K+, 5 mM Mg2+	-51 to 51	3	G3/K3	53K	1.67	183	EMD-47850	3,3	27.3	33
139	P. RNAP T.	1	0	40 mM K+, 5 mM Mg2+	-51 to 51	3	G3/K3	53K	1.67	183	EMD-47850	3,4	28.5	34.5
140	P. RNAP T.	1	0	40 mM K+, 5 mM Mg2+	-51 to 51	3	G3/K3	53K	1.67	183	EMD-47850	3,5	26.9	33.3
141	P. RNAP T.	1	0	40 mM K+, 5 mM Mg2+	-51 to 51	3	G3/K3	53K	1.67	183	EMD-47850	4,1	26.5	33.8
142	P. RNAP T.	1	0	40 mM K+, 5 mM Mg2+	-55 to 55	5	G3/K3	53K	1.67	120	EMD-47850	4,2	27.4	35.6
143	P. RNAP T.	1	0	40 mM K+, 5 mM Mg2+	-51 to 51	3	G3/K3	53K	1.67	183	EMD-47850	4,3	28	37.8
144	P. RNAP T.	0	0	40 mM K+, 5 mM Mg2+	-51 to 51	3	G3/K3	53K	1.67	183	EMD-47850	4,4	27.9	36.4
145	P. RNAP T.	2	0	40 mM K+, 5 mM Mg2+	-51 to 51	3	G3/K3	53K	1.67	183	EMD-47850	4,5	26.3	33.3
146	P. RNAP T.	1	0	40 mM K+, 5 mM Mg2+	-51 to 51	3	G3/K3	53K	1.67	183	EMD-47850	5,1	26.5	34
147	P. RNAP T.	1	0	40 mM K+, 5 mM Mg2+	-51 to 51	3	G3/K3	53K	1.67	183	EMD-47850	5,2	27.8	35
148	P. RNAP T.	1	0	40 mM K+, 5 mM Mg2+	-51 to 51	3	G3/K3	53K	1.67	183	EMD-47850	5,3	29.3	33.3
149	P. RS. Cas	3	1	40 mM K+, 5 mM Mg2+	-55 to 55	5	G3/K3	53K	1.67	120	EMD-47851	1,1	26.8	45.5
150	P. RS. Cas	2	1	40 mM K+, 5 mM Mg2+	-55 to 55	5	G3/K3	53K	1.67	120	EMD-47851	1,2	27.8	41.2
151	P. RS. Cas	1	1	40 mM K+, 5 mM Mg2+	-55 to 35	5	G3/K3	53K	1.67	99	EMD-47851	1,3	25.4	45.4
152	P. RS. Cas	1	1	40 mM K+, 5 mM Mg2+	-55 to 35	5	G3/K3	53K	1.67	99	EMD-47851	1,4	26.5	39.8
153	P. RS. Cas	1	1	40 mM K+, 5 mM Mg2+	-55 to 35	5	G3/K3	53K	1.67	99	EMD-47851	1,5	25.7	45.7
154	P. RS. Cas	1	1	40 mM K+, 5 mM Mg2+	-55 to 35	5	G3/K3	53K	1.67	99	EMD-47851	1,6	26.3	43.5
155	P. RS. Cas	3	1	40 mM K+, 5 mM Mg2+	-55 to 55	5	G3/K3	53K	1.67	120	EMD-47851	1,7	26.1	58.8
156	P. RS. Cas	3	2	40 mM K+, 5 mM Mg2+	-55 to 55	5	G3/K3	53K	1.67	120	EMD-47851	2,1	25.8	57.4
157	P. RS. Cas	1	1	40 mM K+, 5 mM Mg2+	-55 to 35	5	G3/K3	53K	1.67	99	EMD-47851	2,2	24	39.7
158	P. RS. Cas	1	1	40 mM K+, 5 mM Mg2+	-55 to 55	5	G3/K3	53K	1.67	120	EMD-47851	2,3	27.4	46.3
159	P. RS. Cas	3	0	40 mM K+, 5 mM Mg2+	-55 to 55	5	G3/K3	53K	1.67	120	EMD-47851	2,4	28	54.3
160	P. RS. Cas	1	1	40 mM K+, 5 mM Mg2+	-55 to 55	5	G3/K3	53K	1.67	120	EMD-47851	2,5	26.7	46.6
161	P. RS. Cas	1	1	40 mM K+, 5 mM Mg2+	-55 to 55	5	G3/K3	53K	1.67	120	EMD-47851	2,6	26.6	40.5
162	P. RS. Cas	0	1	40 mM K+, 5 mM Mg2+	-55 to 55	5	G3/K3	53K	1.67	120	EMD-47851	2,7	26.6	34
163	P. RS. Cas	2	1	40 mM K+, 5 mM Mg2+	-55 to 55	5	G3/K3	53K	1.67	120	EMD-47851	3,1	27.9	48.3
164	P. RS. Cas	1	1	40 mM K+, 5 mM Mg2+	-55 to 55	5	G3/K3	53K	1.67	120	EMD-47851	3,2	26.9	38.4
165	P. RS. Cas	1	1	40 mM K+, 5 mM Mg2+	-55 to 55	5	G3/K3	53K	1.67	120	EMD-47851	3,3	26.5	47.6
166	P. RS. Cas	1	1	40 mM K+, 5 mM Mg2+	-55 to 45	5	G3/K3	53K	1.67	109	EMD-47851	3,4	27.4	34.5
167	P. RS. Cas	1	1	40 mM K+, 5 mM Mg2+	-55 to 55	5	G3/K3	53K	1.67	120	EMD-47851	3,5	25.2	44.5
168	P. RS. Cas	3	1	40 mM K+, 5 mM Mg2+	-55 to 45	5	G3/K3	53K	1.67	109	EMD-47851	3,6	24.9	43.7
169	P. RS. Cas	2	1	40 mM K+, 5 mM Mg2+	-55 to 45	5	G3/K3	53K	1.67	109	EMD-47851	3,7	28.1	43.2
170	P. RS. Cas	1	1	40 mM K+, 5 mM Mg2+	-55 to 40	5	G3/K3	53K	1.67	104	EMD-47851	4,1	26.8	34.6
171	P. RS. Cas	1	1	40 mM K+, 5 mM Mg2+	-55 to 40	5	G3/K3	53K	1.67	104	EMD-47851	4,2	25.8	37.4
172	P. RS. Cas	1	1	40 mM K+, 5 mM Mg2+	-55 to 30	5	G3/K3	53K	1.67	94	EMD-47851	4,3	25.9	38.5
173	P. RS. Cas	3	1	40 mM K+, 5 mM Mg2+	-55 to 45	5	G3/K3	53K	1.67	109	EMD-47851	4,4	26.3	41.2
174	P. RS. Cas	1	1	40 mM K+, 5 mM Mg2+	-55 to 45	5	G3/K3	53K	1.67	109	EMD-47851	4,5	27.2	37.7
175	P. RS. Cas	1	1	40 mM K+, 5 mM Mg2+	-55 to 30	5	G3/K3	53K	1.67	94	EMD-47851	4,6	27.8	44.4
176	P. RS. Cas	1	1	40 mM K+, 5 mM Mg2+	-55 to 45	5	G3/K3	53K	1.67	109	EMD-47851	4,7	171.4	34.9

Par. id #	Name abbr.	Bound RNAP #	Bound dCas9 #	Salt condition	Tilt range (°)	Tilt step (°)	TEM /Cam.	Mag.	Apix (Å^2)	Total dose (e-/Å^2)	EMDB map ID #	Montage position (row, col)	Resolution (FSC=0.143) Even vs. Odd (Å)	Resolution (FSC=0.5) Map vs. Model (Å)
177	P. RS. Cas	1	1	40 mM K+, 5 mM Mg2+	-55 to 55	5	G3/K3	53K	1.67	120	EMD-47851	5,1	26.7	41
178	P. RS. Cas	1	1	40 mM K+, 5 mM Mg2+	-55 to 55	5	G3/K3	53K	1.67	120	EMD-47851	5,2	26.9	35.7
179	P. RS. Cas	1	1	40 mM K+, 5 mM Mg2+	-55 to 55	5	G3/K3	53K	1.67	120	EMD-47851	5,3	27	35.9
180	P. RS. Cas	2	1	40 mM K+, 5 mM Mg2+	-55 to 35	5	G3/K3	53K	1.67	99	EMD-47851	5,4	28.1	44.6
181	P. RS. Cas	1	1	40 mM K+, 5 mM Mg2+	-55 to 35	5	G3/K3	53K	1.67	99	EMD-47851	5,5	26.3	40.6
182	P. RS. Cas	1	1	40 mM K+, 5 mM Mg2+	-55 to 45	5	G3/K3	53K	1.67	109	EMD-47851	5,6	27.9	43
183	P. RT. Cas	5	1	40 mM K+, 5 mM Mg2+	-55 to 55	5	G3/K3	53K	1.67	120	EMD-47853	1,1	27.1	46.6
184	P. RT. Cas	1	1	40 mM K+, 5 mM Mg2+	-55 to 55	5	G3/K3	53K	1.67	120	EMD-47853	1,2	28	35.1
185	P. RT. Cas	3	1	40 mM K+, 5 mM Mg2+	-55 to 55	5	G3/K3	53K	1.67	120	EMD-47853	1,3	29.1	40.5
186	P. RT. Cas	3	1	40 mM K+, 5 mM Mg2+	-55 to 55	5	G3/K3	53K	1.67	120	EMD-47853	1,4	27.6	45.1
187	P. RT. Cas	1	1	40 mM K+, 5 mM Mg2+	-55 to 55	5	G3/K3	53K	1.67	120	EMD-47853	1,5	28.1	39.7
188	P. RT. Cas	3	1	40 mM K+, 5 mM Mg2+	-55 to 55	5	G3/K3	53K	1.67	120	EMD-47853	1,6	27.3	41.3
189	P. RT. Cas	4	1	40 mM K+, 5 mM Mg2+	-55 to 55	5	G3/K3	53K	1.67	120	EMD-47853	1,7	27.2	37.4
190	P. RT. Cas	1	1	40 mM K+, 5 mM Mg2+	-55 to 55	5	G3/K3	53K	1.67	120	EMD-47853	1,8	28	39.6
191	P. RT. Cas	1	1	40 mM K+, 5 mM Mg2+	-55 to 55	5	G3/K3	53K	1.67	120	EMD-47853	1,9	27.4	41.1
192	P. RT. Cas	1	1	40 mM K+, 5 mM Mg2+	-50 to 40	5	G3/K3	53K	1.67	99	EMD-47853	2,1	28.2	37.8
193	P. RT. Cas	1	0	40 mM K+, 5 mM Mg2+	-50 to 40	5	G3/K3	53K	1.67	99	EMD-47853	2,2	27.6	38
194	P. RT. Cas	1	1	40 mM K+, 5 mM Mg2+	-50 to 40	5	G3/K3	53K	1.67	99	EMD-47853	2,3	27.1	41
195	P. RT. Cas	4	1	40 mM K+, 5 mM Mg2+	-50 to 40	5	G3/K3	53K	1.67	99	EMD-47853	2,4	27.5	44.8
196	P. RT. Cas	1	1	40 mM K+, 5 mM Mg2+	-50 to 40	5	G3/K3	53K	1.67	99	EMD-47853	2,5	26.9	43.7
197	P. RT. Cas	2	1	40 mM K+, 5 mM Mg2+	-50 to 40	5	G3/K3	53K	1.67	99	EMD-47853	2,6	26.3	39.8
198	P. RT. Cas	2	1	40 mM K+, 5 mM Mg2+	-50 to 40	5	G3/K3	53K	1.67	99	EMD-47853	2,7	27	41.8
199	P. RT. Cas	3	1	40 mM K+, 5 mM Mg2+	-50 to 40	5	G3/K3	53K	1.67	99	EMD-47853	2,8	28.6	49.2
200	P. RT. Cas	1	1	40 mM K+, 5 mM Mg2+	-55 to 55	5	G3/K3	53K	1.67	120	EMD-47853	2,9	27.2	40.3
201	P. RT. Cas	0	1	40 mM K+, 5 mM Mg2+	-50 to 40	5	G3/K3	53K	1.67	99	EMD-47853	3,1	29.3	40.4
202	P. RT. Cas	3	1	40 mM K+, 5 mM Mg2+	-50 to 40	5	G3/K3	53K	1.67	99	EMD-47853	3,2	26.5	44.8
203	P. RT. Cas	4	1	40 mM K+, 5 mM Mg2+	-55 to 55	5	G3/K3	53K	1.67	120	EMD-47853	3,3	27.4	47.7
204	P. RT. Cas	3	1	40 mM K+, 5 mM Mg2+	-55 to 55	5	G3/K3	53K	1.67	120	EMD-47853	3,4	27.8	43.1
205	P. RT. Cas	1	1	40 mM K+, 5 mM Mg2+	-55 to 55	5	G3/K3	53K	1.67	120	EMD-47853	3,5	26.1	33.7
206	P. RT. Cas	3	1	40 mM K+, 5 mM Mg2+	-55 to 55	5	G3/K3	53K	1.67	120	EMD-47853	3,6	27.3	42.1
207	P. RT. Cas	1	1	40 mM K+, 5 mM Mg2+	-55 to 50	5	G3/K3	53K	1.67	115	EMD-47853	3,7	28	37.2
208	P. RT. Cas	1	1	40 mM K+, 5 mM Mg2+	-55 to 50	5	G3/K3	53K	1.67	115	EMD-47853	3,8	29.3	43.1
209	P. RT. Cas	1	1	40 mM K+, 5 mM Mg2+	-55 to 55	5	G3/K3	53K	1.67	120	EMD-47853	3,9	27.8	47.5
210	P. RT. Cas	2	0	40 mM K+, 5 mM Mg2+	-55 to 55	5	G3/K3	53K	1.67	120	EMD-47853	4,1	27.1	34.9
211	P. RT. Cas	1	1	40 mM K+, 5 mM Mg2+	-55 to 55	5	G3/K3	53K	1.67	120	EMD-47853	4,2	26.7	39.2
212	P. RT. Cas	2	1	40 mM K+, 5 mM Mg2+	-55 to 55	5	G3/K3	53K	1.67	120	EMD-47853	4,3	27.3	45
213	P. RT. Cas	3	1	40 mM K+, 5 mM Mg2+	-55 to 55	5	G3/K3	53K	1.67	120	EMD-47853	4,4	28.2	50.9
214	P. RT. Cas	1	1	40 mM K+, 5 mM Mg2+	-55 to 55	5	G3/K3	53K	1.67	120	EMD-47853	4,5	26.7	37.1
215	P. RT. Cas	2	1	40 mM K+, 5 mM Mg2+	-55 to 55	5	G3/K3	53K	1.67	120	EMD-47853	4,6	28.7	36.8
216	P. RT. Cas	3	1	40 mM K+, 5 mM Mg2+	-55 to 55	5	G3/K3	53K	1.67	120	EMD-47853	4,7	29	48.7
217	P. RT. Cas	2	2	40 mM K+, 5 mM Mg2+	-55 to 55	5	G3/K3	53K	1.67	120	EMD-47853	4,8	27.3	39.8
218	P. RT. Cas	1	1	40 mM K+, 5 mM Mg2+	-55 to 55	5	G3/K3	53K	1.67	120	EMD-47853	4,9	24.4	39.8
219	P. RT. Cas	3	1	40 mM K+, 5 mM Mg2+	-55 to 55	5	G3/K3	53K	1.67	120	EMD-47853	5,1	29.2	48.6
220	P. RT. Cas	2	1	40 mM K+, 5 mM Mg2+	-55 to 55	5	G3/K3	53K	1.67	120	EMD-47853	5,2	27.6	43.6
221	P. RT. Cas	1	1	40 mM K+, 5 mM Mg2+	-55 to 55	5	G3/K3	53K	1.67	120	EMD-47853	5,3	27.7	41.2
222	P. RT. Cas	1	1	40 mM K+, 5 mM Mg2+	-55 to 55	5	G3/K3	53K	1.67	120	EMD-47853	5,4	27.6	49.9
223	P. RT. Cas	2	1	40 mM K+, 5 mM Mg2+	-55 to 55	5	G3/K3	53K	1.67	120	EMD-47853	5,5	27.6	35.9
224	P. RT. Cas	2	1	40 mM K+, 5 mM Mg2+	-55 to 55	5	G3/K3	53K	1.67	120	EMD-47853	5,6	27.7	46.3
225	P. RT. Cas	3	1	40 mM K+, 5 mM Mg2+	-55 to 55	5	G3/K3	53K	1.67	120	EMD-47853	5,7	28.2	47.4
226	P. RT. Cas	3	1	40 mM K+, 5 mM Mg2+	-55 to 55	5	G3/K3	53K	1.67	120	EMD-47853	5,8	26.8	91.1
227	P. RT. Top	1	0	40 mM K+, 5 mM Mg2+	-55 to 55	5	G3/K3	53K	1.67	120	EMD-47855	1,1	29.9	36.6
228	P. RT. Top	1	0	40 mM K+, 5 mM Mg2+	-55 to 55	5	G3/K3	53K	1.67	120	EMD-47855	1,2	30	35.7
229	P. RT. Top	1	0	40 mM K+, 5 mM Mg2+	-55 to 55	5	G3/K3	53K	1.67	120	EMD-47855	1,3	31.9	34.5
230	P. RT. Top	1	0	40 mM K+, 5 mM Mg2+	-55 to 55	5	G3/K3	53K	1.67	120	EMD-47855	1,4	31.3	35.3
231	P. RT. Top	2	0	40 mM K+, 5 mM Mg2+	-55 to 55	5	G3/K3	53K	1.67	120	EMD-47855	1,5	29.7	603.5
232	P. RT. Top	1	0	40 mM K+, 5 mM Mg2+	-55 to 55	5	G3/K3	53K	1.67	120	EMD-47855	1,6	31	34
233	P. RT. Top	1	0	40 mM K+, 5 mM Mg2+	-55 to 55	5	G3/K3	53K	1.67	120	EMD-47855	1,7	31	38.2
234	P. RT. Top	1	0	40 mM K+, 5 mM Mg2+	-55 to 55	5	G3/K3	53K	1.67	120	EMD-47855	1,8	32.3	39.4
235	P. RT. Top	1	0	40 mM K+, 5 mM Mg2+	-55 to 55	5	G3/K3	53K	1.67	120	EMD-47855	2,1	31.5	34.2
236	P. RT. Top	1	0	40 mM K+, 5 mM Mg2+	-55 to 55	5	G3/K3	53K	1.67	120	EMD-47855	2,2	33	33.7
237	P. RT. Top	1	0	40 mM K+, 5 mM Mg2+	-55 to 55	5	G3/K3	53K	1.67	120	EMD-47855	2,3	30.8	33.6
238	P. RT. Top	1	0	40 mM K+, 5 mM Mg2+	-55 to 55	5	G3/K3	53K	1.67	120	EMD-47855	2,4	32.5	33.3
239	P. RT. Top	1	0	40 mM K+, 5 mM Mg2+	-55 to 55	5	G3/K3	53K	1.67	120	EMD-47855	2,5	31.8	36
240	P. RT. Top	1	0	40 mM K+, 5 mM Mg2+	-55 to 55	5	G3/K3	53K	1.67	120	EMD-47855	2,6	33.2	32.9
241	P. RT. Top	1	0	40 mM K+, 5 mM Mg2+	-55 to 55	5	G3/K3	53K	1.67	120	EMD-47855	2,7	31.4	39.4
242	P. RT. Top	0	0	40 mM K+, 5 mM Mg2+	-55 to 55	5	G3/K3	53K	1.67	120	EMD-47855	2,8	28.2	32.3
243	P. RT. Top	1	0	40 mM K+, 5 mM Mg2+	-55 to 55	5	G3/K3	53K	1.67	120	EMD-47855	3,1	33.2	34.8
244	P. RT. Top	2	0	40 mM K+, 5 mM Mg2+	-55 to 55	5	G3/K3	53K	1.67	120	EMD-47855	3,2	31.5	37
245	P. RT. Top	1	0	40 mM K+, 5 mM Mg2+	-55 to 55	5	G3/K3	53K	1.67	120	EMD-47855	3,3	29.1	41.8
246	P. RT. Top	1	0	40 mM K+, 5 mM Mg2+	-55 to 55	5	G3/K3	53K	1.67	120	EMD-47855	3,4	28.1	32.9
247	P. RT. Top	0	0	40 mM K+, 5 mM Mg2+	-55 to 55	5	G3/K3	53K	1.67	120	EMD-47855	3,5	32.7	32.9
248	P. RT. Top	1	0	40 mM K+, 5 mM Mg2+	-55 to 55	5	G3/K3	53K	1.67	120	EMD-47855	3,6	27.5	35.6
249	P. RT. Top	1	0	40 mM K+, 5 mM Mg2+	-55 to 55	5	G3/K3	53K	1.67	120	EMD-47855	3,7	29.1	37.4
250	P. RT. Top	1	0	40 mM K+, 5 mM Mg2+	-55 to 55	5	G3/K3	53K	1.67	120	EMD-47855	3,8	28.2	39.3
251	P. RT. Top	1	0	40 mM K+, 5 mM Mg2+	-55 to 55	5	G3/K3	53K	1.67	120	EMD-47855	4,1	28	38.6
252	P. RT. Top	1	0	40 mM K+, 5 mM Mg2+	-55 to 55	5	G3/K3	53K	1.67	120	EMD-47855	4,2	29.4	38.8
253	P. RT. Top	1	0	40 mM K+, 5 mM Mg2+	-55 to 55	5	G3/K3	53K	1.67	120	EMD-47855	4,3	28.8	41
254	P. RT. Top	1	0	40 mM K+, 5 mM Mg2+	-55 to 55	5	G3/K3	53K	1.67	120	EMD-47855	4,4	30.7	35.4
255	P. RT. Top	1	0	40 mM K+, 5 mM Mg2+	-55 to 55	5	G3/K3	53K	1.67	120	EMD-47855	4,5	28.1	43.8
256	P. RT. Top	1	0	40 mM K+, 5 mM Mg2+	-55 to 55	5	G3/K3	53K	1.67	120	EMD-47855	4,6	30.6	39.3
257	P. RT. Top	1	0	40 mM K+, 5 mM Mg2+	-55 to 55	5	G3/K3	53K	1.67	120	EMD-47855	4,7	28.4	37.2
258	P. RT. Top	1	0	40 mM K+, 5 mM Mg2+	-55 to 55	5	G3/K3	53K	1.67	120	EMD-47855	4,8	29.2	38.7
259	P. RT. Top	2	0	40 mM K+, 5 mM Mg2+	-55 to 55	5	G3/K3	53K	1.67	120	EMD-47855	5,1	29.4	46.8
260	P. RT. Top	1	0	40 mM K+, 5 mM Mg2+	-55 to 55	5	G3/K3	53K	1.67	120	EMD-47855	5,2	30.9	34.1
261	P. RT. Top	1	0	40 mM K+, 5 mM Mg2+	-55 to 55	5	G3/K3	53K	1.67	120	EMD-47855	5,3	28.4	30.4
262	P. RT. Top	1	0	40 mM K+, 5 mM Mg2+	-55 to 55	5	G3/K3	53K	1.67	120	EMD-47855	5,4	30.2	33.7
263	P. RT. Top	1	0	40 mM K+, 5 mM Mg2+	-55 to 55	5	G3/K3	53K	1.67	120	EMD-47855	5,5	29.3	39.4
264	P. RT. Top	0	0	40 mM K+, 5 mM Mg2+	-55 to 55	5	G3/K3	53K	1.67	120	EMD-47855	5,6	32.1	35.6

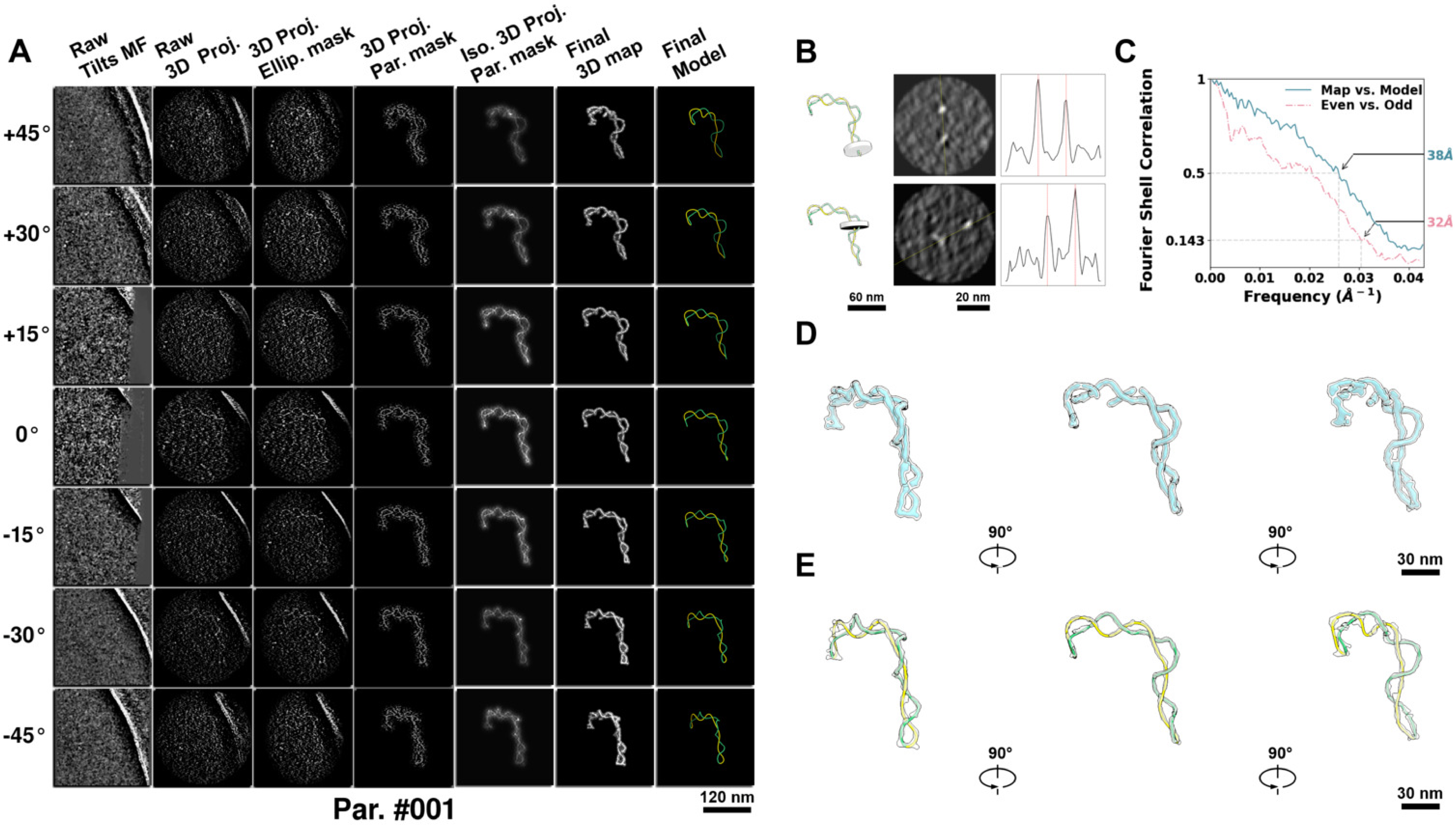

Supplementary Figure 1. Cryo-electron tomography (cryo-ET) 3D reconstruction of an individual plasmid particle in 5 mM K⁺. (A) 3D reconstruction of the plasmid particle (index no. 1). The first column shows seven representative tilt images from –45° to +45° in step of 15°. The second, third, and fourth columns show 3D projections of the particle with spherical, ellipsoidal (thinner along the z-dimension), and particle-shaped masks, respectively. The fifth column displays the 3D projections of the enhanced and IsoNet missing-wedge-corrected particle. The sixth and seventh columns present the final 3D map and the flexibly fitted model, respectively. (B) Two random cross-sectional views (12 nm thickness) of the plasmid density map along its plectoneme axis are shown in the left-middle panel. The intensity profile along the line crossing the two high-density DNA spots is displayed in the right panel. (C) Resolution assessment of the final 3D map using Fourier shell correlation (FSC). Two criteria are shown: FSC between two half-maps reconstructed from even and odd frames (evaluated at 0.143) and FSC between the final 3D map and the fitted model (evaluated at 0.5). (D) Zoomed-in views of the final 3D density map from panel A, displayed at two contour levels. (E) Superimposition of the high-contour level map from panel D onto its flexibly fitted model.

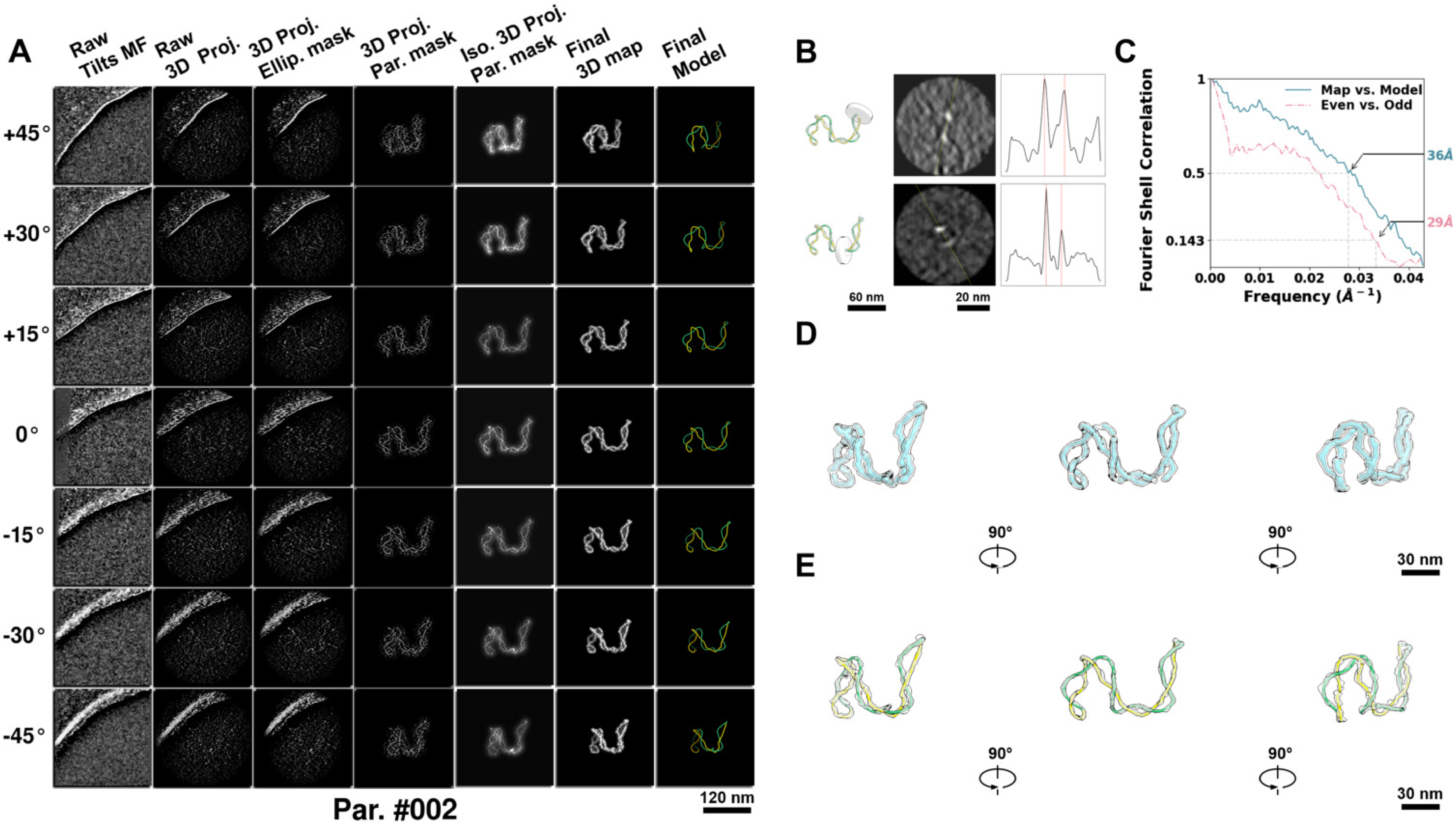

Supplementary Figure 2. Cryo-electron tomography (cryo-ET) 3D reconstruction of an individual plasmid particle in 5 mM K⁺. (A) 3D reconstruction of the plasmid particle (index no. 2). The first column shows seven representative tilt images from –45° to +45° in step of 15°. The second, third, and fourth columns show 3D projections of the particle with spherical, ellipsoidal (thinner along the z-dimension), and particle-shaped masks, respectively. The fifth column displays the 3D projections of the enhanced and IsoNet missing-wedge-corrected particle. The sixth and seventh columns present the final 3D map and the flexibly fitted model, respectively. (B) Two random cross-sectional views (12 nm thickness) of the plasmid density map along its plectoneme axis are shown in the left-middle panel. The intensity profile along the line crossing the two high-density DNA spots is displayed in the right panel. (C) Resolution assessment of the final 3D map using Fourier shell correlation (FSC). Two criteria are shown: FSC between two half-maps reconstructed from even and odd frames (evaluated at 0.143) and FSC between the final 3D map and the fitted model (evaluated at 0.5). (D) Zoomed-in views of the final 3D density map from panel A, displayed at two contour levels. (E) Superimposition of the high-contour level map from panel D onto its flexibly fitted model.

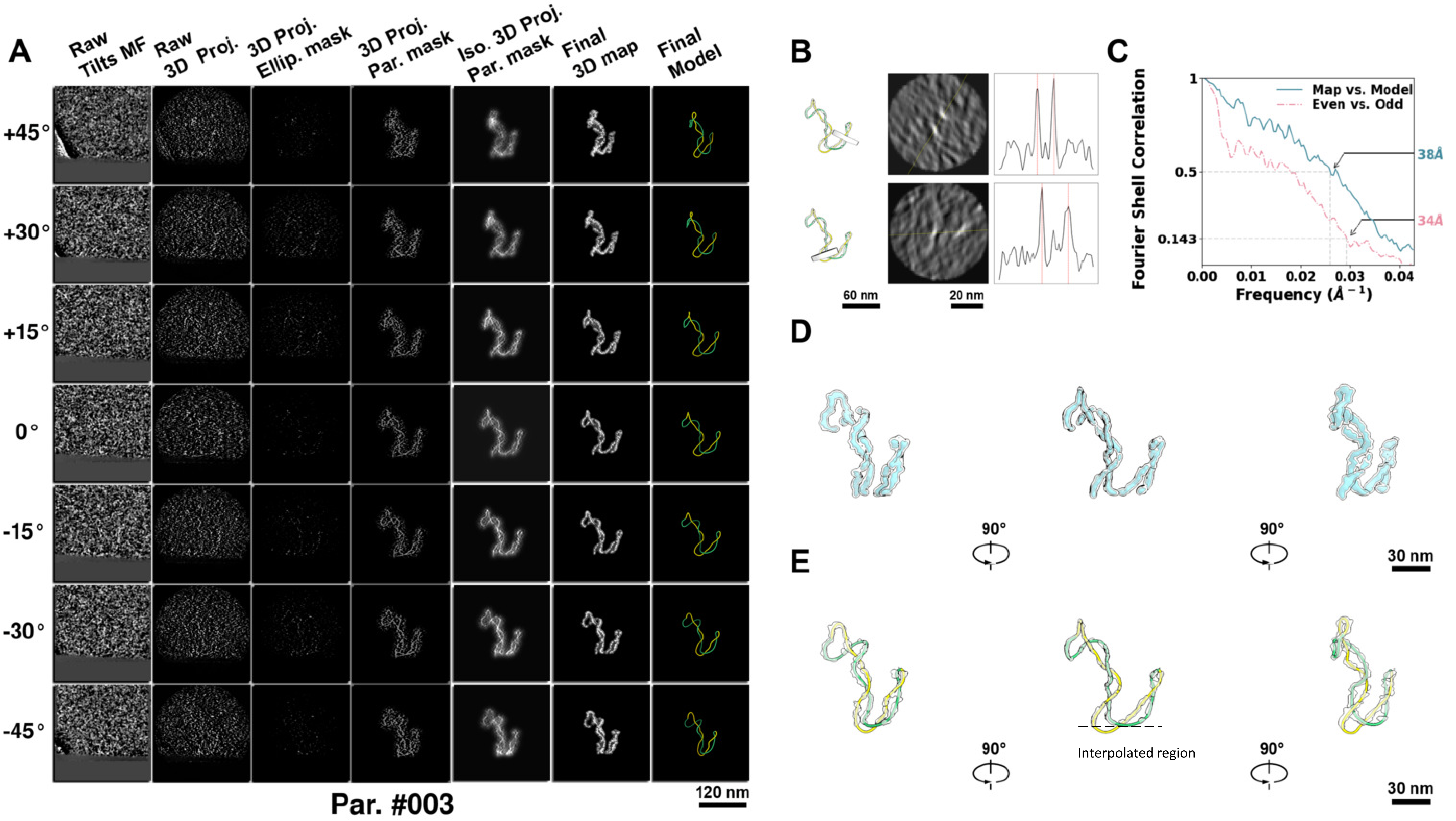

Supplementary Figure 3. Cryo-electron tomography (cryo-ET) 3D reconstruction of an individual plasmid particle in 5 mM K⁺.
(A) 3D reconstruction of the plasmid particle (index no. 3). The first column shows seven representative tilt images from –45° to +45° in step of 15°. The second, third, and fourth columns show 3D projections of the particle with spherical, ellipsoidal (thinner along the z-dimension), and particle-shaped masks, respectively. The fifth column displays the 3D projections of the enhanced and IsoNet missing-wedge-corrected particle. The sixth and seventh columns present the final 3D map and the flexibly fitted model, respectively. (B) Two random cross-sectional views (12 nm thickness) of the plasmid density map along its plectoneme axis are shown in the left-middle panel. The intensity profile along the line crossing the two high-density DNA spots is displayed in the right panel. (C) Resolution assessment of the final 3D map using Fourier shell correlation (FSC). Two criteria are shown: FSC between two half-maps reconstructed from even and odd frames (evaluated at 0.143) and FSC between the final 3D map and the fitted model (evaluated at 0.5). (D) Zoomed-in views of the final 3D density map from panel A, displayed at two contour levels. (E) Superimposition of the high-contour level map from panel D onto its flexibly fitted model.

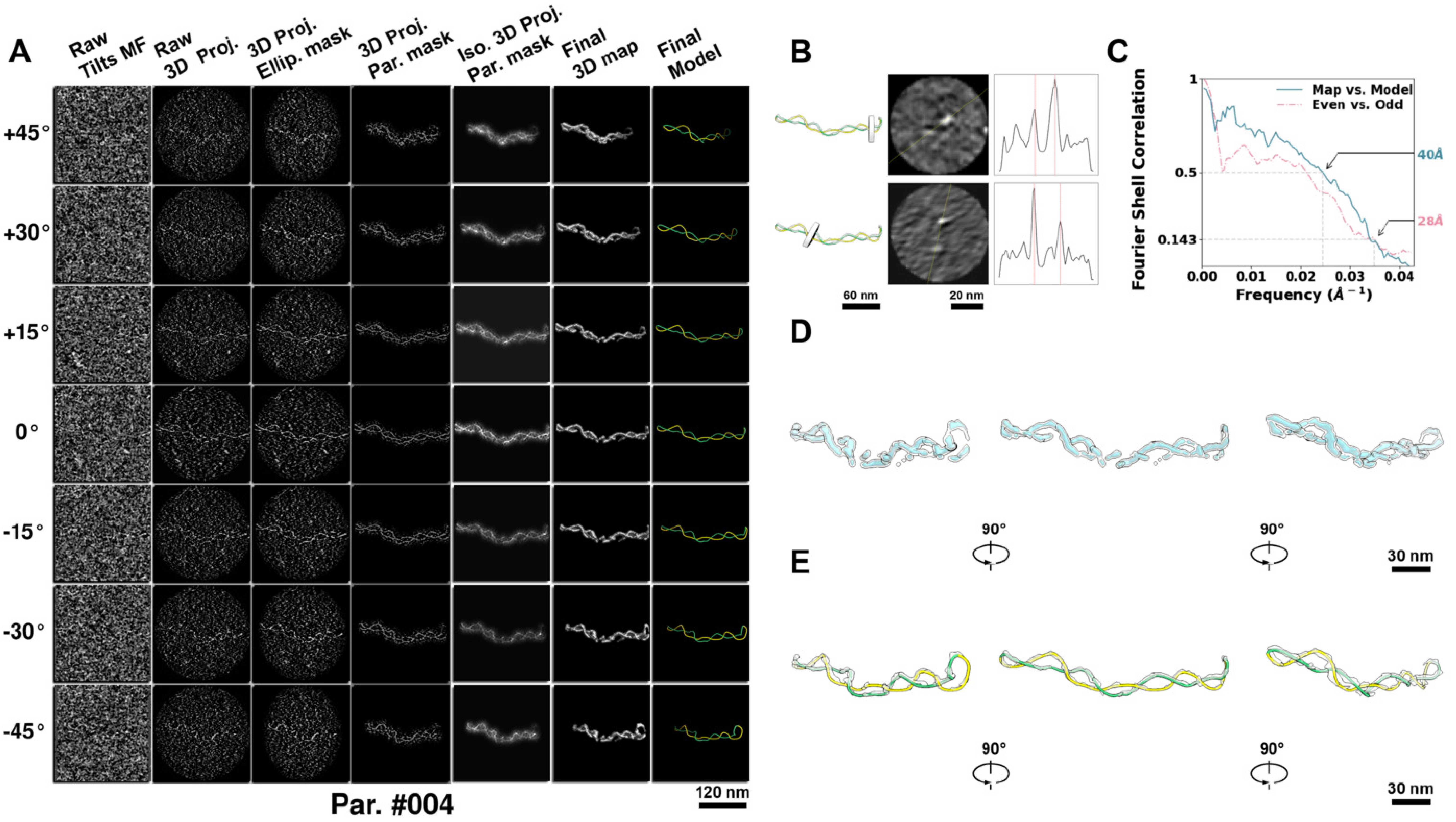

Supplementary Figure 4. Cryo-electron tomography (cryo-ET) 3D reconstruction of an individual plasmid particle in 5 mM K⁺.
(A) 3D reconstruction of the plasmid particle (index no. 4). The first column shows seven representative tilt images from –45° to +45° in step of 15°. The second, third, and fourth columns show 3D projections of the particle with spherical, ellipsoidal (thinner along the z-dimension), and particle-shaped masks, respectively. The fifth column displays the 3D projections of the enhanced and IsoNet missing-wedge-corrected particle. The sixth and seventh columns present the final 3D map and the flexibly fitted model, respectively. (B) Two random cross-sectional views (12 nm thickness) of the plasmid density map along its plectoneme axis are shown in the left-middle panel. The intensity profile along the line crossing the two high-density DNA spots is displayed in the right panel. (C) Resolution assessment of the final 3D map using Fourier shell correlation (FSC). Two criteria are shown: FSC between two half-maps reconstructed from even and odd frames (evaluated at 0.143) and FSC between the final 3D map and the fitted model (evaluated at 0.5). (D) Zoomed-in views of the final 3D density map from panel A, displayed at two contour levels. (E) Superimposition of the high-contour level map from panel D onto its flexibly fitted model.

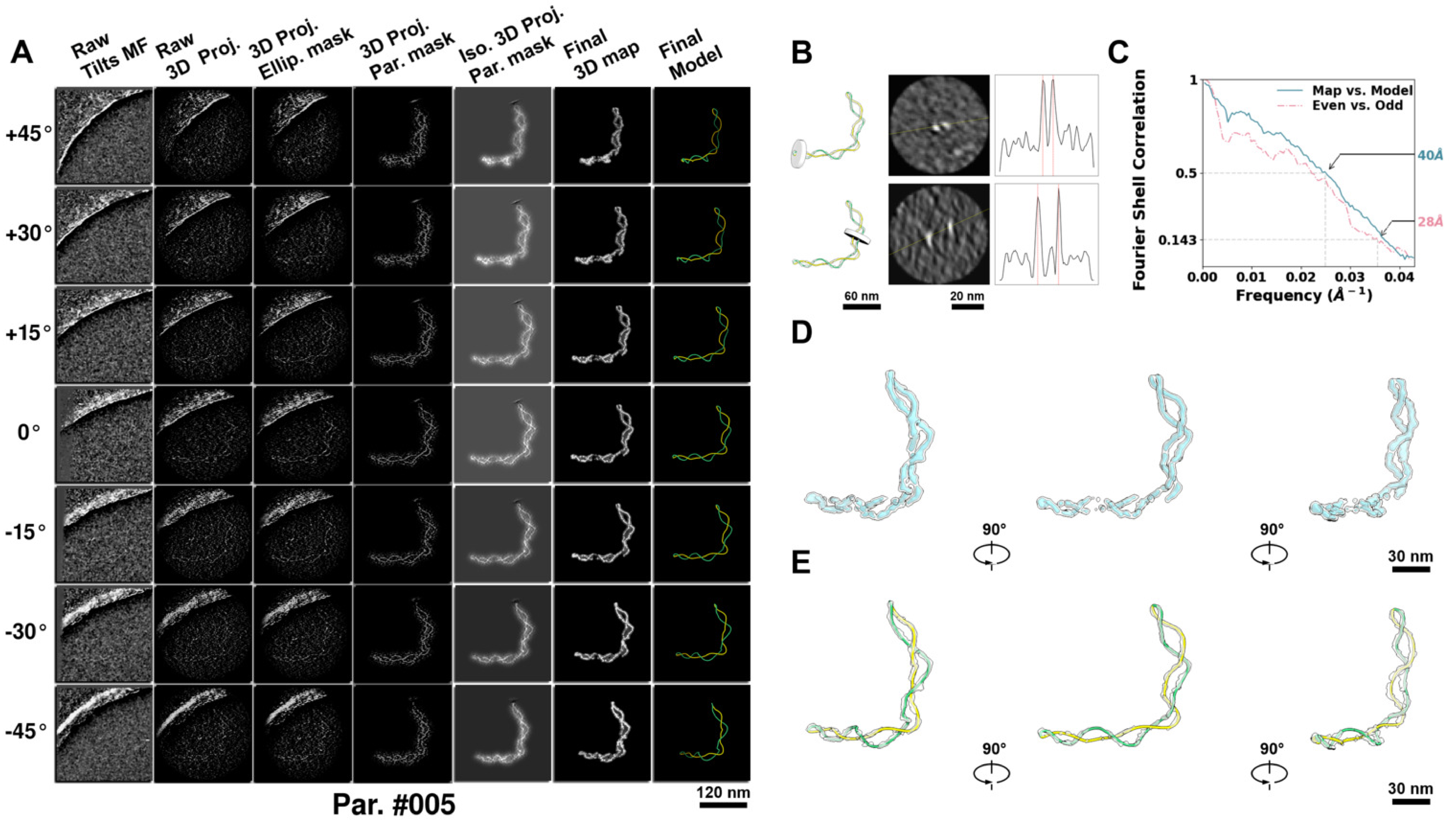

Supplementary Figure 5. Cryo-electron tomography (cryo-ET) 3D reconstruction of an individual plasmid particle in 5 mM K⁺. (A) 3D reconstruction of the plasmid particle (index no. 5). The first column shows seven representative tilt images from –45° to +45° in step of 15°. The second, third, and fourth columns show 3D projections of the particle with spherical, ellipsoidal (thinner along the z-dimension), and particle-shaped masks, respectively. The fifth column displays the 3D projections of the enhanced and IsoNet missing-wedge-corrected particle. The sixth and seventh columns present the final 3D map and the flexibly fitted model, respectively. (B) Two random cross-sectional views (12 nm thickness) of the plasmid density map along its plectoneme axis are shown in the left-middle panel. The intensity profile along the line crossing the two high-density DNA spots is displayed in the right panel. (C) Resolution assessment of the final 3D map using Fourier shell correlation (FSC). Two criteria are shown: FSC between two half-maps reconstructed from even and odd frames (evaluated at 0.143) and FSC between the final 3D map and the fitted model (evaluated at 0.5). (D) Zoomed-in views of the final 3D density map from panel A, displayed at two contour levels. (E) Superimposition of the high-contour level map from panel D onto its flexibly fitted model.

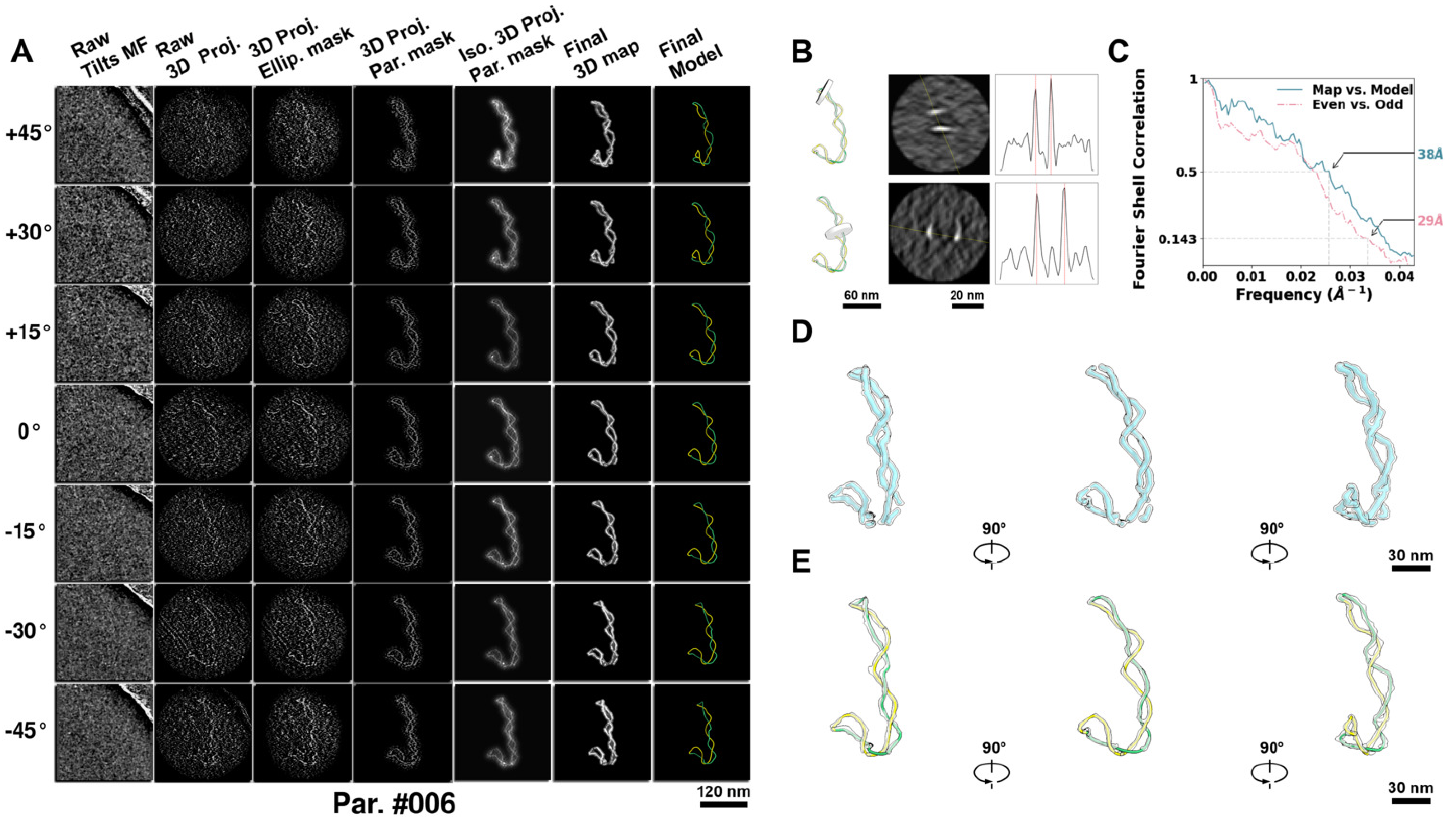

Supplementary Figure 6. Cryo-electron tomography (cryo-ET) 3D reconstruction of an individual plasmid particle in 5 mM K⁺. (A) 3D reconstruction of the plasmid particle (index no. 6). The first column shows seven representative tilt images from –45° to +45° in step of 15°. The second, third, and fourth columns show 3D projections of the particle with spherical, ellipsoidal (thinner along the z-dimension), and particle-shaped masks, respectively. The fifth column displays the 3D projections of the enhanced and IsoNet missing-wedge-corrected particle. The sixth and seventh columns present the final 3D map and the flexibly fitted model, respectively. (B) Two random cross-sectional views (12 nm thickness) of the plasmid density map along its plectoneme axis are shown in the left-middle panel. The intensity profile along the line crossing the two high-density DNA spots is displayed in the right panel. (C) Resolution assessment of the final 3D map using Fourier shell correlation (FSC). Two criteria are shown: FSC between two half-maps reconstructed from even and odd frames (evaluated at 0.143) and FSC between the final 3D map and the fitted model (evaluated at 0.5). (D) Zoomed-in views of the final 3D density map from panel A, displayed at two contour levels. (E) Superimposition of the high-contour level map from panel D onto its flexibly fitted model.

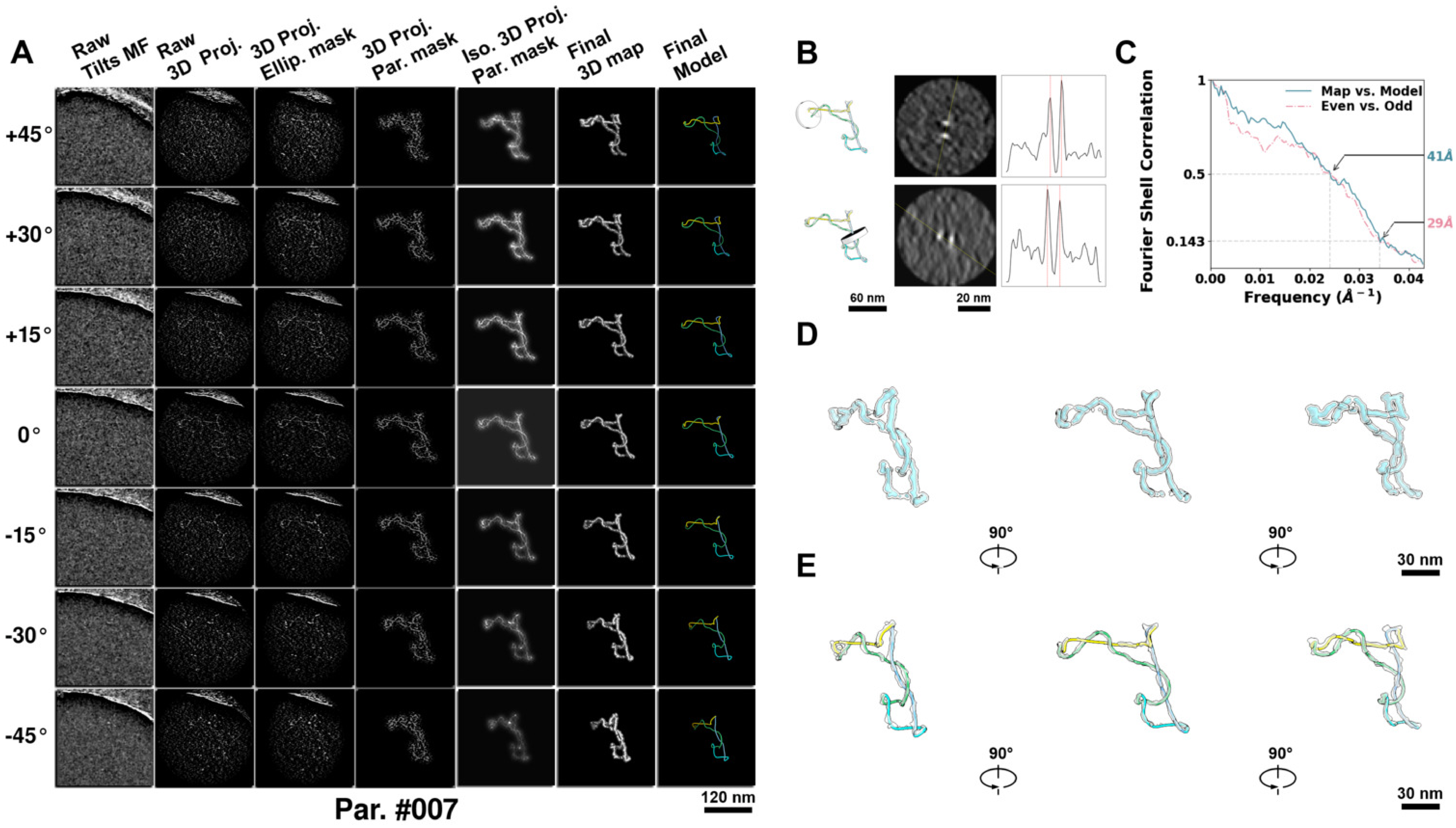

Supplementary Figure 7. Cryo-electron tomography (cryo-ET) 3D reconstruction of an individual plasmid particle in 5 mM K⁺. (A) 3D reconstruction of the plasmid particle (index no. 7). The first column shows seven representative tilt images from –45° to +45° in step of 15°. The second, third, and fourth columns show 3D projections of the particle with spherical, ellipsoidal (thinner along the z-dimension), and particle-shaped masks, respectively. The fifth column displays the 3D projections of the enhanced and IsoNet missing-wedge-corrected particle. The sixth and seventh columns present the final 3D map and the flexibly fitted model, respectively. (B) Two random cross-sectional views (12 nm thickness) of the plasmid density map along its plectoneme axis are shown in the left-middle panel. The intensity profile along the line crossing the two high-density DNA spots is displayed in the right panel. (C) Resolution assessment of the final 3D map using Fourier shell correlation (FSC). Two criteria are shown: FSC between two half-maps reconstructed from even and odd frames (evaluated at 0.143) and FSC between the final 3D map and the fitted model (evaluated at 0.5). (D) Zoomed-in views of the final 3D density map from panel A, displayed at two contour levels. (E) Superimposition of the high-contour level map from panel D onto its flexibly fitted model.

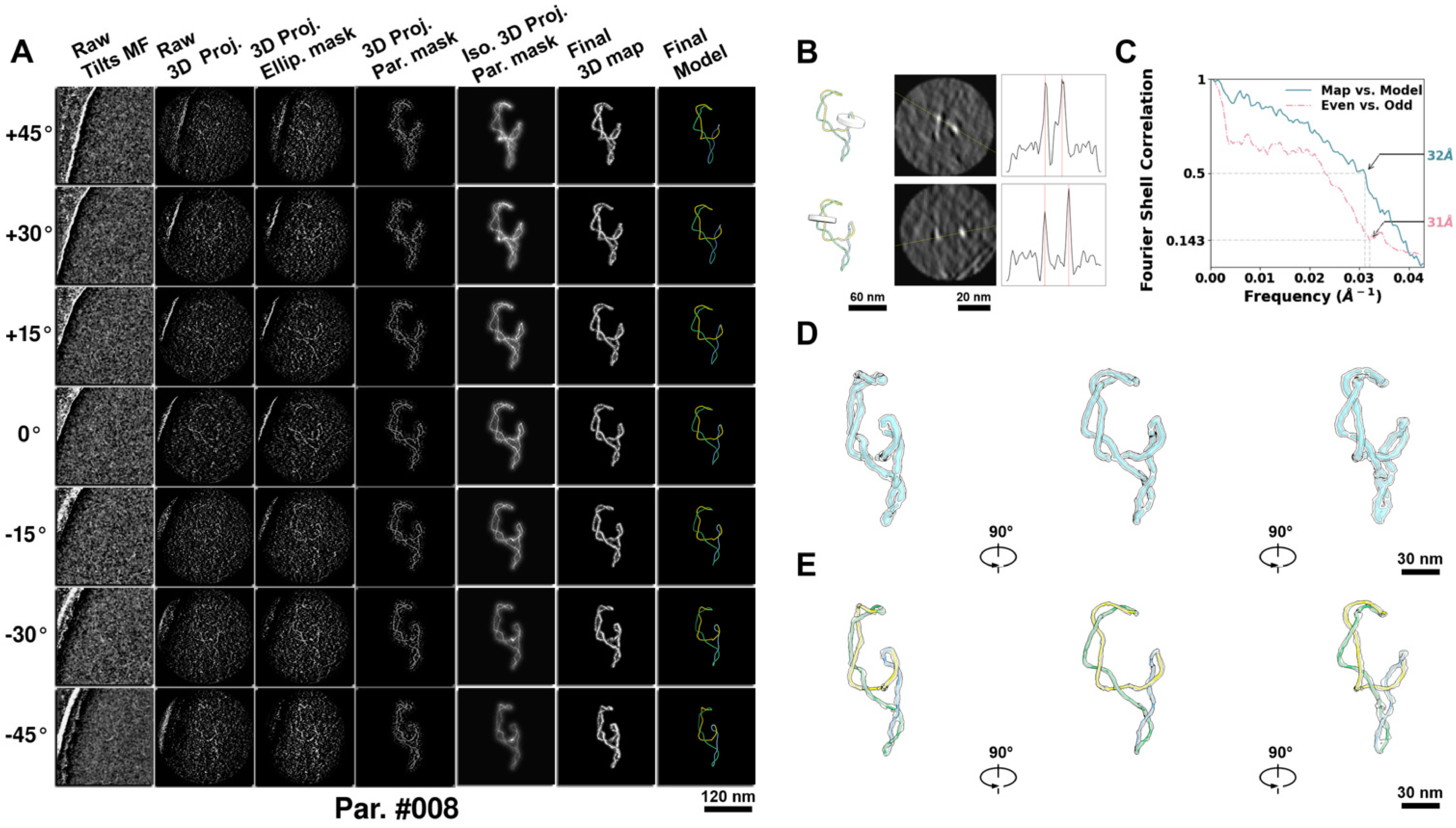

Supplementary Figure 8. Cryo-electron tomography (cryo-ET) 3D reconstruction of an individual plasmid particle in 5 mM K⁺. (A) 3D reconstruction of the plasmid particle (index no. 8). The first column shows seven representative tilt images from –45° to +45° in step of 15°. The second, third, and fourth columns show 3D projections of the particle with spherical, ellipsoidal (thinner along the z-dimension), and particle-shaped masks, respectively. The fifth column displays the 3D projections of the enhanced and IsoNet missing-wedge-corrected particle. The sixth and seventh columns present the final 3D map and the flexibly fitted model, respectively. (B) Two random cross-sectional views (12 nm thickness) of the plasmid density map along its plectoneme axis are shown in the left-middle panel. The intensity profile along the line crossing the two high-density DNA spots is displayed in the right panel. (C) Resolution assessment of the final 3D map using Fourier shell correlation (FSC). Two criteria are shown: FSC between two half-maps reconstructed from even and odd frames (evaluated at 0.143) and FSC between the final 3D map and the fitted model (evaluated at 0.5). (D) Zoomed-in views of the final 3D density map from panel A, displayed at two contour levels. (E) Superimposition of the high-contour level map from panel D onto its flexibly fitted model.

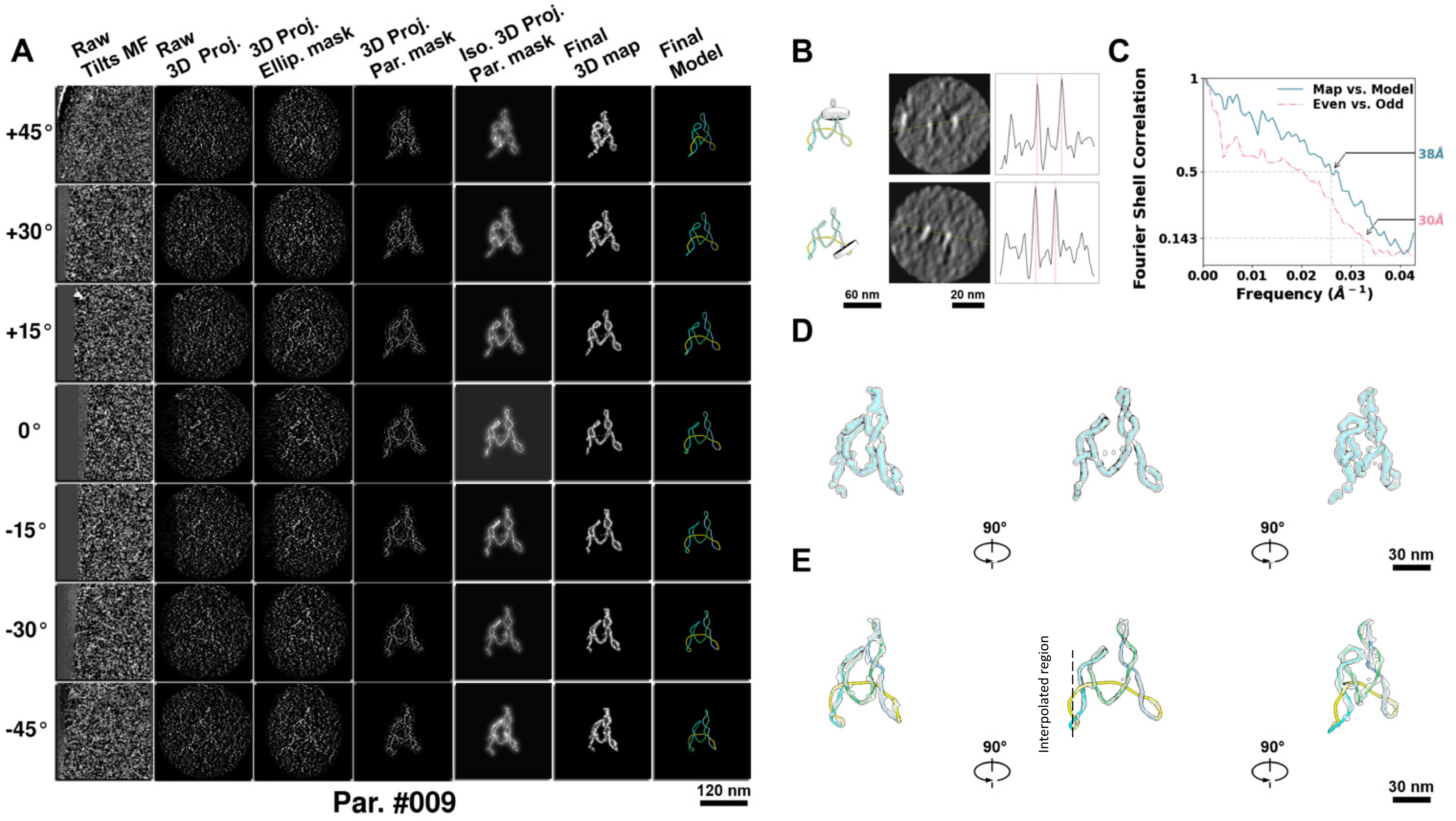

Supplementary Figure 9. Cryo-electron tomography (cryo-ET) 3D reconstruction of an individual plasmid particle in 5 mM K⁺.
(A) 3D reconstruction of the plasmid particle (index no. 9). The first column shows seven representative tilt images from –45° to +45° in step of 15°. The second, third, and fourth columns show 3D projections of the particle with spherical, ellipsoidal (thinner along the z-dimension), and particle-shaped masks, respectively. The fifth column displays the 3D projections of the enhanced and IsoNet missing-wedge-corrected particle. The sixth and seventh columns present the final 3D map and the flexibly fitted model, respectively. (B) Two random cross-sectional views (12 nm thickness) of the plasmid density map along its plectoneme axis are shown in the left-middle panel. The intensity profile along the line crossing the two high-density DNA spots is displayed in the right panel. (C) Resolution assessment of the final 3D map using Fourier shell correlation (FSC). Two criteria are shown: FSC between two half-maps reconstructed from even and odd frames (evaluated at 0.143) and FSC between the final 3D map and the fitted model (evaluated at 0.5). (D) Zoomed-in views of the final 3D density map from panel A, displayed at two contour levels. (E) Superimposition of the high-contour level map from panel D onto its flexibly fitted model.

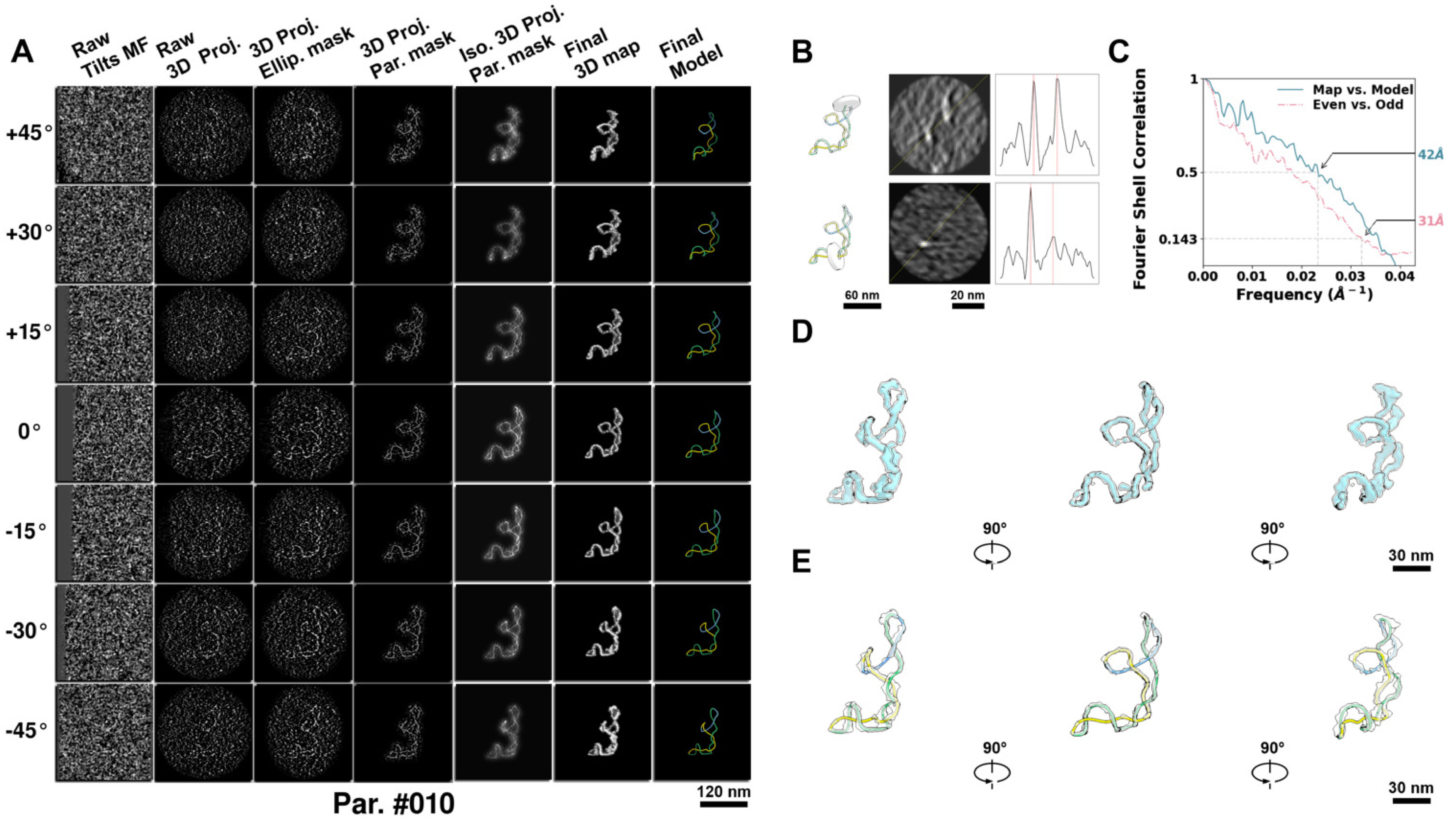

Supplementary Figure 10. Cryo-electron tomography (cryo-ET) 3D reconstruction of an individual plasmid particle in 5 mM K⁺.
(A) 3D reconstruction of the plasmid particle (index no. 10). The first column shows seven representative tilt images from –45° to +45° in step of 15°. The second, third, and fourth columns show 3D projections of the particle with spherical, ellipsoidal (thinner along the z-dimension), and particle-shaped masks, respectively. The fifth column displays the 3D projections of the enhanced and IsoNet missing-wedge-corrected particle. The sixth and seventh columns present the final 3D map and the flexibly fitted model, respectively. (B) Two random cross-sectional views (12 nm thickness) of the plasmid density map along its plectoneme axis are shown in the left-middle panel. The intensity profile along the line crossing the two high-density DNA spots is displayed in the right panel. (C) Resolution assessment of the final 3D map using Fourier shell correlation (FSC). Two criteria are shown: FSC between two half-maps reconstructed from even and odd frames (evaluated at 0.143) and FSC between the final 3D map and the fitted model (evaluated at 0.5). (D) Zoomed-in views of the final 3D density map from panel A, displayed at two contour levels. (E) Superimposition of the high-contour level map from panel D onto its flexibly fitted model.

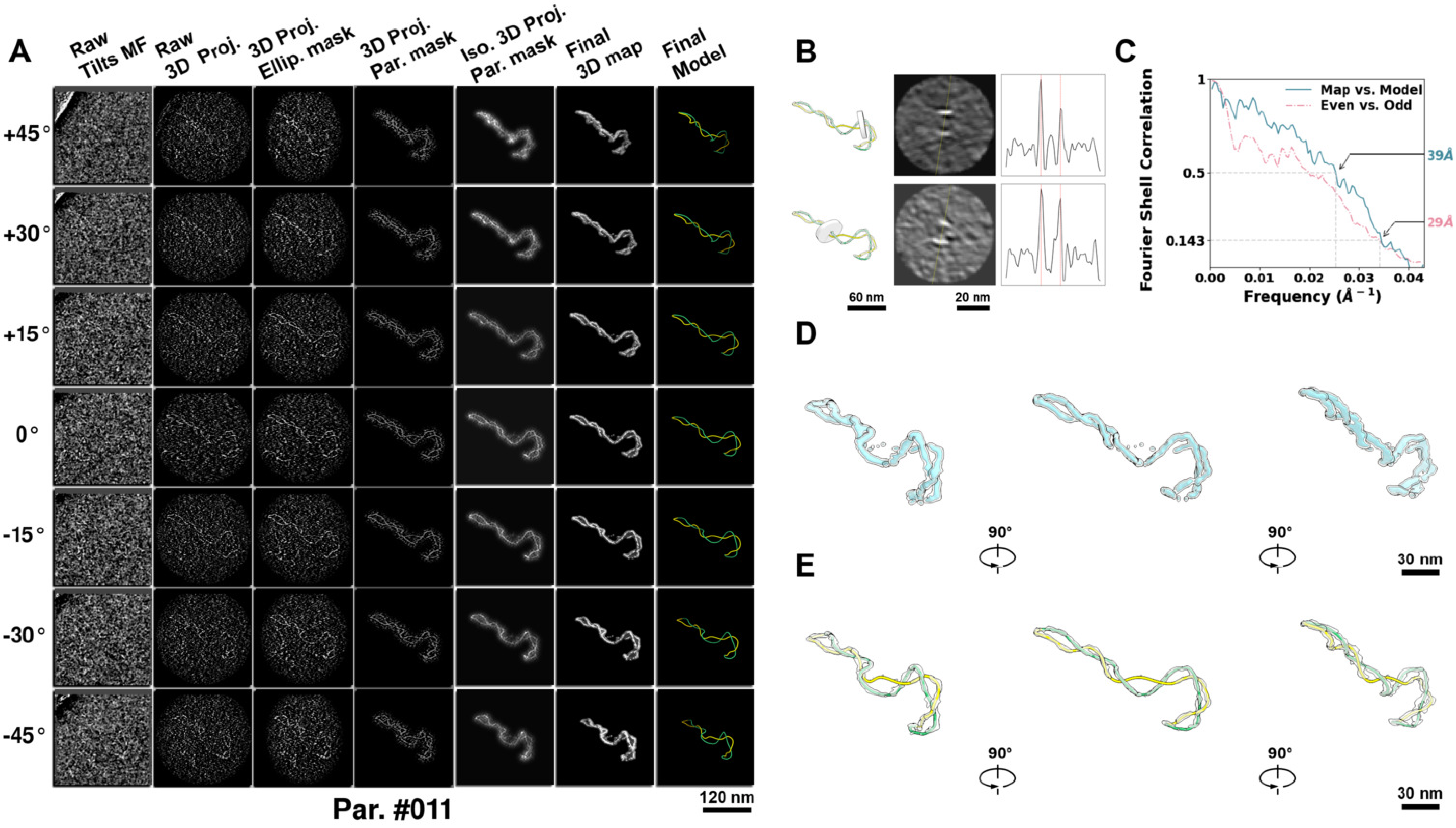

Supplementary Figure 11. Cryo-electron tomography (cryo-ET) 3D reconstruction of an individual plasmid particle in 5 mM K⁺.
(A) 3D reconstruction of the plasmid particle (index no. 11). The first column shows seven representative tilt images from –45° to +45° in step of 15°. The second, third, and fourth columns show 3D projections of the particle with spherical, ellipsoidal (thinner along the z-dimension), and particle-shaped masks, respectively. The fifth column displays the 3D projections of the enhanced and IsoNet missing-wedge-corrected particle. The sixth and seventh columns present the final 3D map and the flexibly fitted model, respectively. (B) Two random cross-sectional views (12 nm thickness) of the plasmid density map along its plectoneme axis are shown in the left-middle panel. The intensity profile along the line crossing the two high-density DNA spots is displayed in the right panel. (C) Resolution assessment of the final 3D map using Fourier shell correlation (FSC). Two criteria are shown: FSC between two half-maps reconstructed from even and odd frames (evaluated at 0.143) and FSC between the final 3D map and the fitted model (evaluated at 0.5). (D) Zoomed-in views of the final 3D density map from panel A, displayed at two contour levels. (E) Superimposition of the high-contour level map from panel D onto its flexibly fitted model.

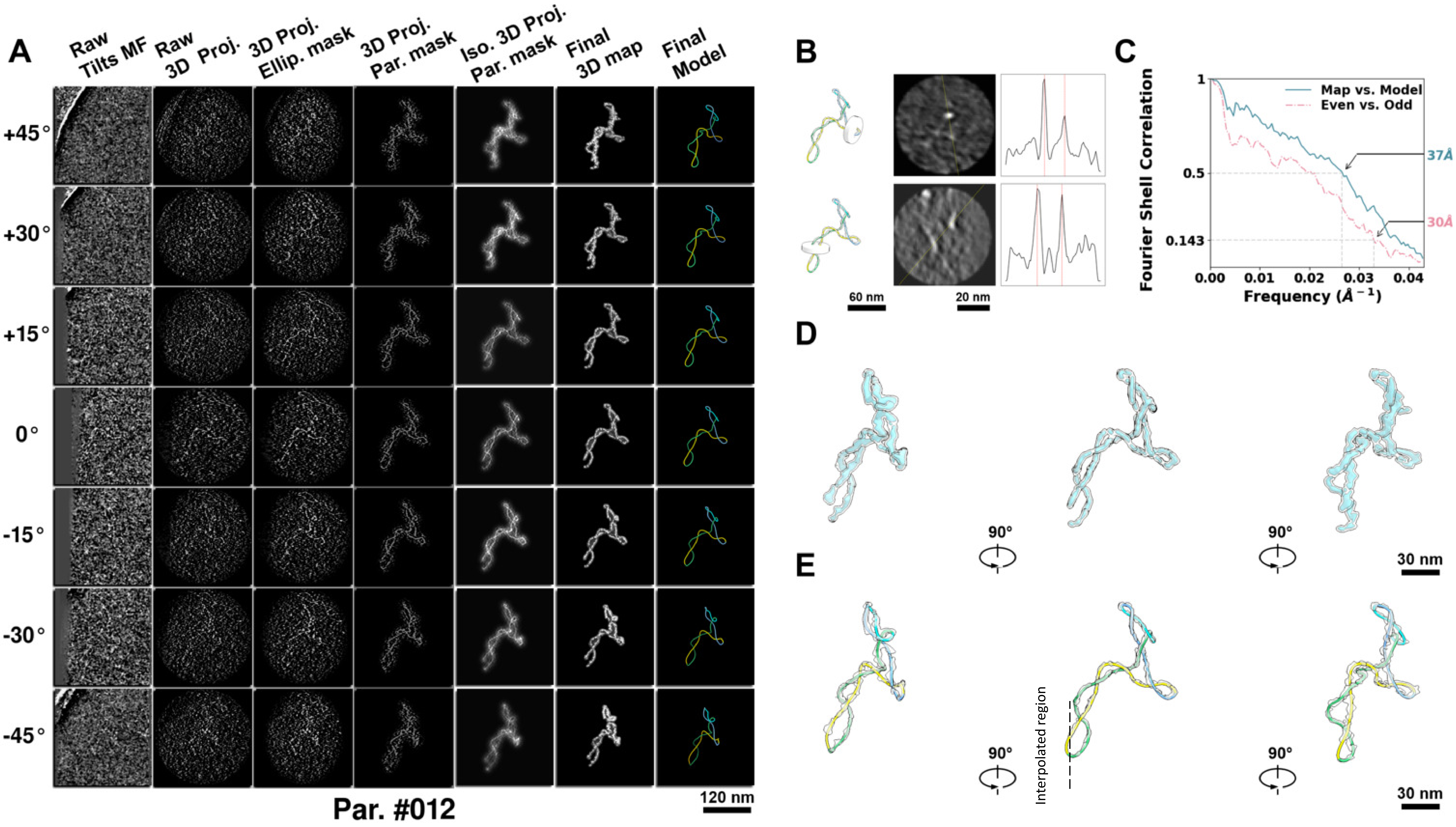

Supplementary Figure 12. Cryo-electron tomography (cryo-ET) 3D reconstruction of an individual plasmid particle in 5 mM K⁺.
(A) 3D reconstruction of the plasmid particle (index no. 12). The first column shows seven representative tilt images from –45° to +45° in step of 15°. The second, third, and fourth columns show 3D projections of the particle with spherical, ellipsoidal (thinner along the z-dimension), and particle-shaped masks, respectively. The fifth column displays the 3D projections of the enhanced and IsoNet missing-wedge-corrected particle. The sixth and seventh columns present the final 3D map and the flexibly fitted model, respectively. (B) Two random cross-sectional views (12 nm thickness) of the plasmid density map along its plectoneme axis are shown in the left-middle panel. The intensity profile along the line crossing the two high-density DNA spots is displayed in the right panel. (C) Resolution assessment of the final 3D map using Fourier shell correlation (FSC). Two criteria are shown: FSC between two half-maps reconstructed from even and odd frames (evaluated at 0.143) and FSC between the final 3D map and the fitted model (evaluated at 0.5). (D) Zoomed-in views of the final 3D density map from panel A, displayed at two contour levels. (E) Superimposition of the high-contour level map from panel D onto its flexibly fitted model.

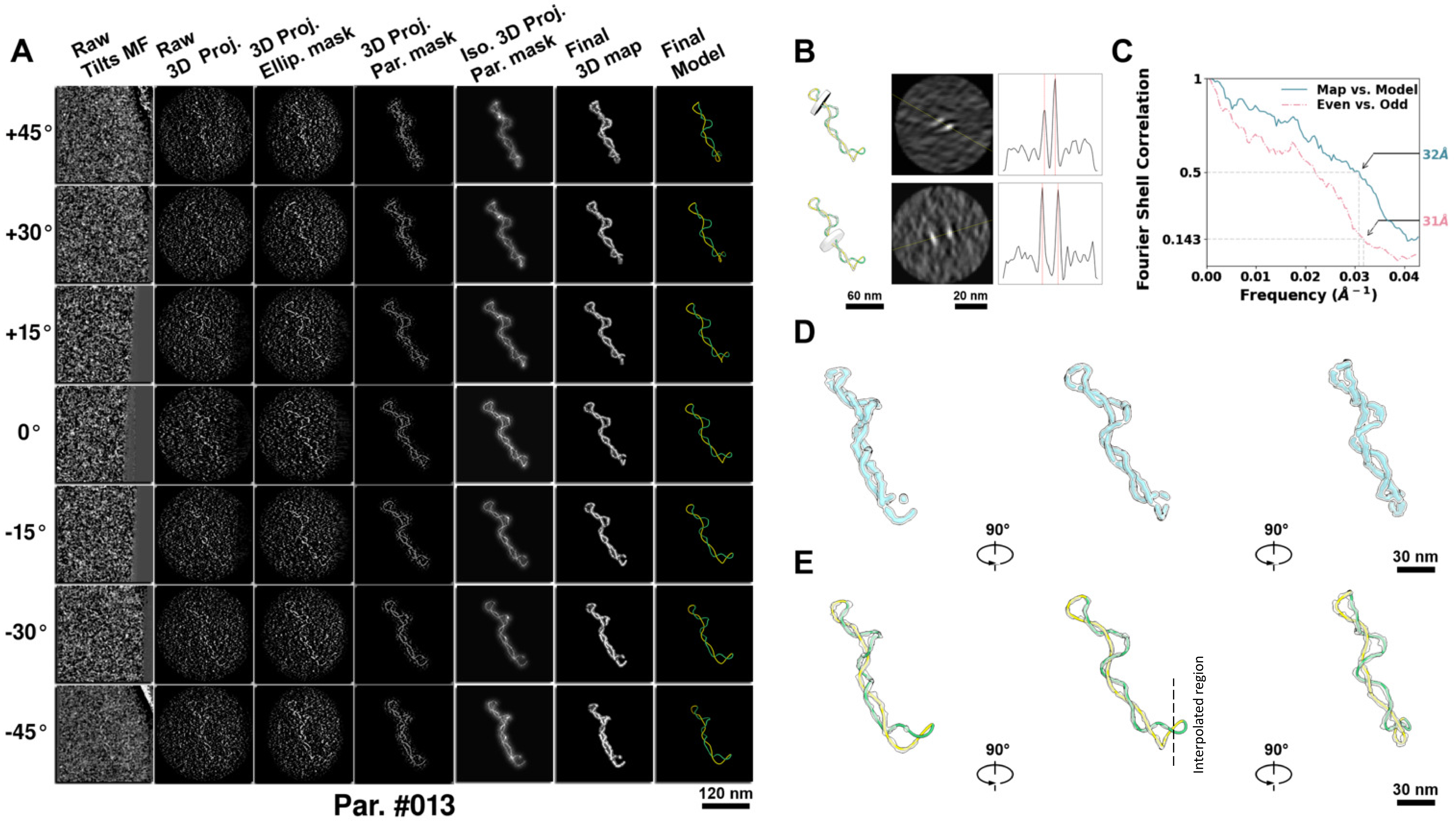

Supplementary Figure 13. Cryo-electron tomography (cryo-ET) 3D reconstruction of an individual plasmid particle in 5 mM K⁺. (A) 3D reconstruction of the plasmid particle (index no. 13). The first column shows seven representative tilt images from –45° to +45° in step of 15°. The second, third, and fourth columns show 3D projections of the particle with spherical, ellipsoidal (thinner along the z-dimension), and particle-shaped masks, respectively. The fifth column displays the 3D projections of the enhanced and IsoNet missing-wedge-corrected particle. The sixth and seventh columns present the final 3D map and the flexibly fitted model, respectively. (B) Two random cross-sectional views (12 nm thickness) of the plasmid density map along its plectoneme axis are shown in the left-middle panel. The intensity profile along the line crossing the two high-density DNA spots is displayed in the right panel. (C) Resolution assessment of the final 3D map using Fourier shell correlation (FSC). Two criteria are shown: FSC between two half-maps reconstructed from even and odd frames (evaluated at 0.143) and FSC between the final 3D map and the fitted model (evaluated at 0.5). (D) Zoomed-in views of the final 3D density map from panel A, displayed at two contour levels. (E) Superimposition of the high-contour level map from panel D onto its flexibly fitted model.

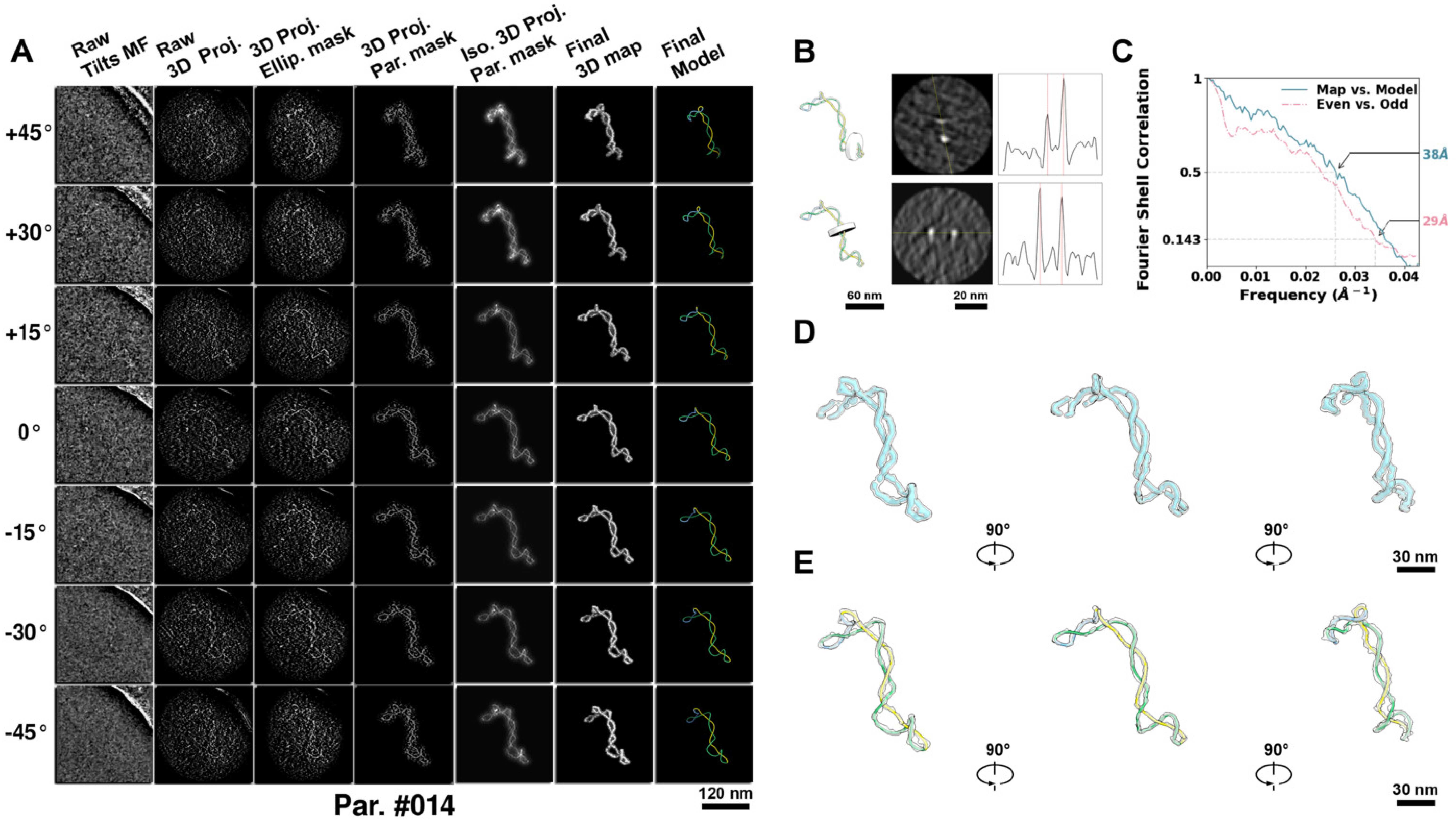

Supplementary Figure 14. Cryo-electron tomography (cryo-ET) 3D reconstruction of an individual plasmid particle in 5 mM K⁺. (A) 3D reconstruction of the plasmid particle (index no. 14). The first column shows seven representative tilt images from –45° to +45° in step of 15°. The second, third, and fourth columns show 3D projections of the particle with spherical, ellipsoidal (thinner along the z-dimension), and particle-shaped masks, respectively. The fifth column displays the 3D projections of the enhanced and IsoNet missing-wedge-corrected particle. The sixth and seventh columns present the final 3D map and the flexibly fitted model, respectively. (B) Two random cross-sectional views (12 nm thickness) of the plasmid density map along its plectoneme axis are shown in the left-middle panel. The intensity profile along the line crossing the two high-density DNA spots is displayed in the right panel. (C) Resolution assessment of the final 3D map using Fourier shell correlation (FSC). Two criteria are shown: FSC between two half-maps reconstructed from even and odd frames (evaluated at 0.143) and FSC between the final 3D map and the fitted model (evaluated at 0.5). (D) Zoomed-in views of the final 3D density map from panel A, displayed at two contour levels. (E) Superimposition of the high-contour level map from panel D onto its flexibly fitted model.

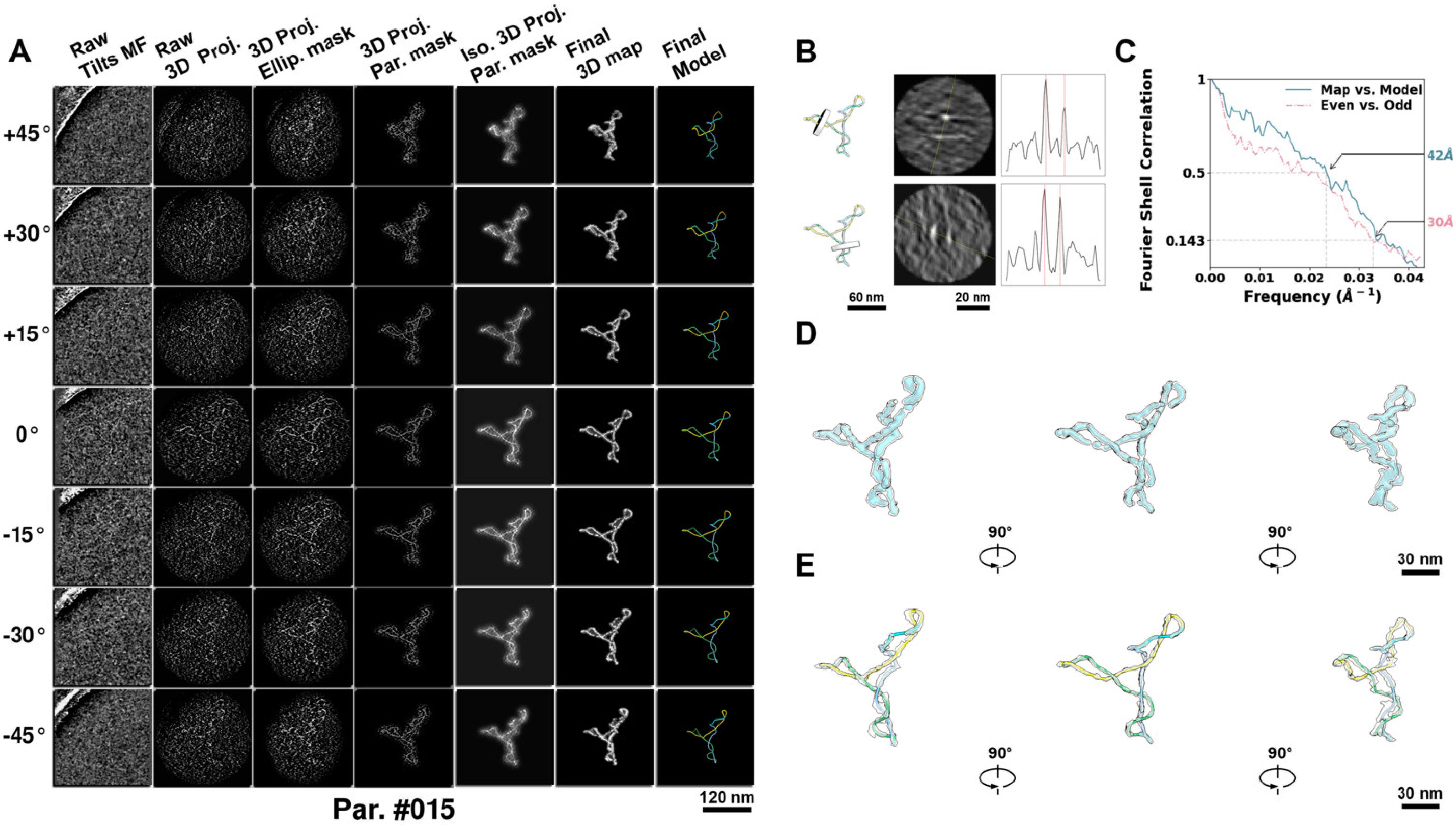

Supplementary Figure 15. Cryo-electron tomography (cryo-ET) 3D reconstruction of an individual plasmid particle in 5 mM K⁺. (A) 3D reconstruction of the plasmid particle (index no. 15). The first column shows seven representative tilt images from –45° to +45° in step of 15°. The second, third, and fourth columns show 3D projections of the particle with spherical, ellipsoidal (thinner along the z-dimension), and particle-shaped masks, respectively. The fifth column displays the 3D projections of the enhanced and IsoNet missing-wedge-corrected particle. The sixth and seventh columns present the final 3D map and the flexibly fitted model, respectively. (B) Two random cross-sectional views (12 nm thickness) of the plasmid density map along its plectoneme axis are shown in the left-middle panel. The intensity profile along the line crossing the two high-density DNA spots is displayed in the right panel. (C) Resolution assessment of the final 3D map using Fourier shell correlation (FSC). Two criteria are shown: FSC between two half-maps reconstructed from even and odd frames (evaluated at 0.143) and FSC between the final 3D map and the fitted model (evaluated at 0.5). (D) Zoomed-in views of the final 3D density map from panel A, displayed at two contour levels. (E) Superimposition of the high-contour level map from panel D onto its flexibly fitted model.

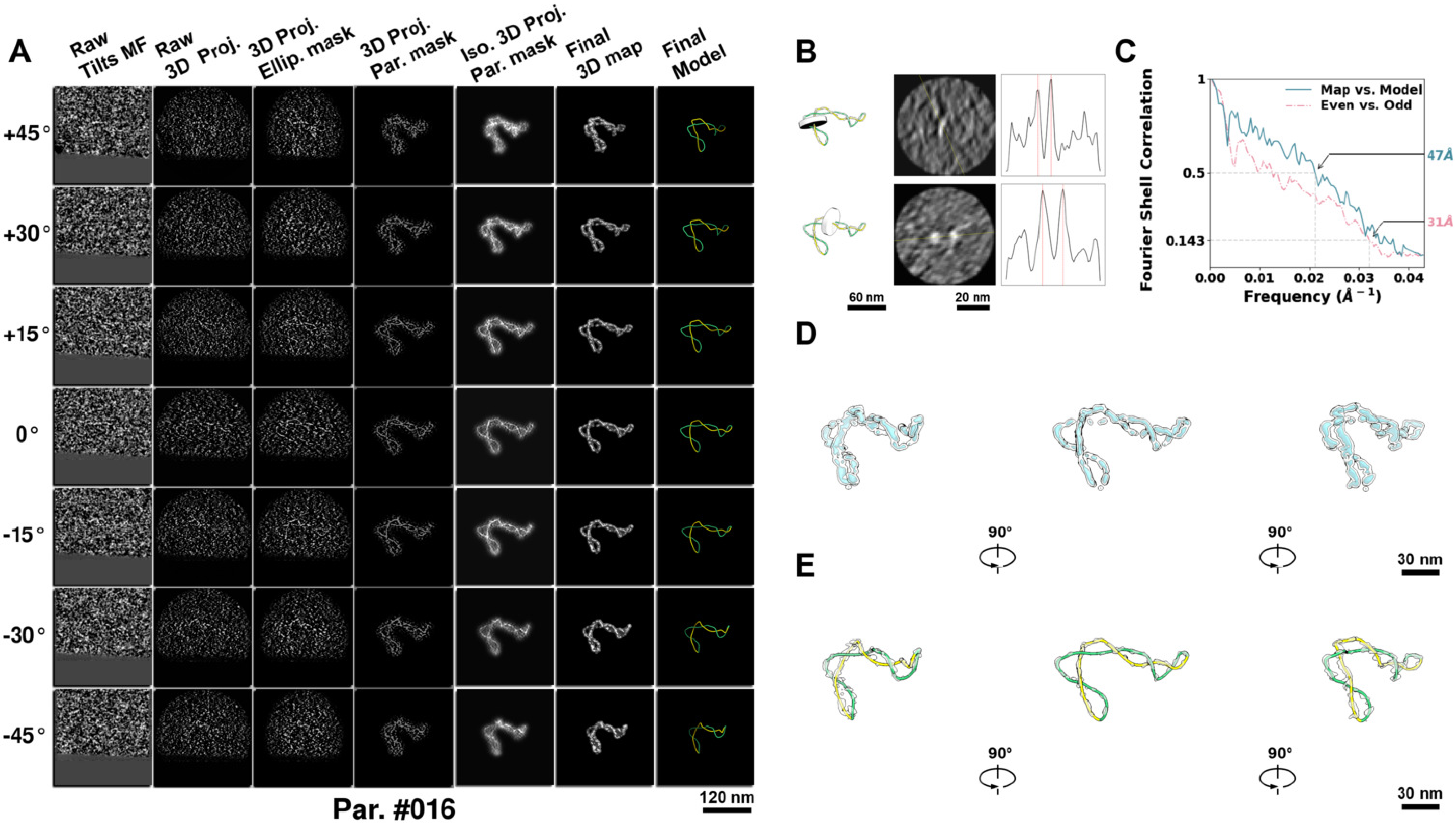

Supplementary Figure 16. Cryo-electron tomography (cryo-ET) 3D reconstruction of an individual plasmid particle in 5 mM K⁺. (A) 3D reconstruction of the plasmid particle (index no. 16). The first column shows seven representative tilt images from –45° to +45° in step of 15°. The second, third, and fourth columns show 3D projections of the particle with spherical, ellipsoidal (thinner along the z-dimension), and particle-shaped masks, respectively. The fifth column displays the 3D projections of the enhanced and IsoNet missing-wedge-corrected particle. The sixth and seventh columns present the final 3D map and the flexibly fitted model, respectively. (B) Two random cross-sectional views (12 nm thickness) of the plasmid density map along its plectoneme axis are shown in the left-middle panel. The intensity profile along the line crossing the two high-density DNA spots is displayed in the right panel. (C) Resolution assessment of the final 3D map using Fourier shell correlation (FSC). Two criteria are shown: FSC between two half-maps reconstructed from even and odd frames (evaluated at 0.143) and FSC between the final 3D map and the fitted model (evaluated at 0.5). (D) Zoomed-in views of the final 3D density map from panel A, displayed at two contour levels. (E) Superimposition of the high-contour level map from panel D onto its flexibly fitted model.

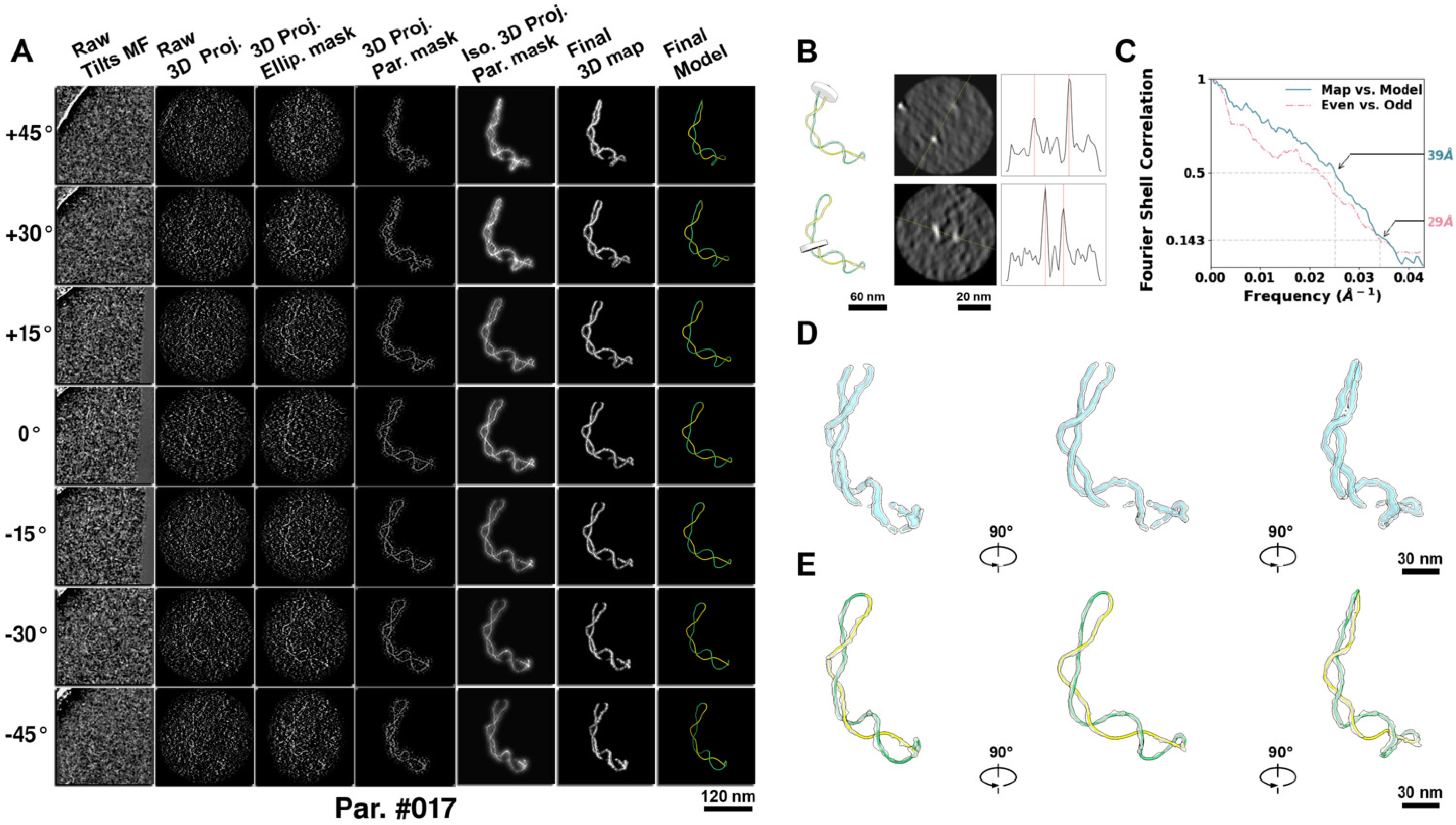

Supplementary Figure 17. Cryo-electron tomography (cryo-ET) 3D reconstruction of an individual plasmid particle in 5 mM K⁺.
(A) 3D reconstruction of the plasmid particle (index no. 17). The first column shows seven representative tilt images from –45° to +45° in step of 15°. The second, third, and fourth columns show 3D projections of the particle with spherical, ellipsoidal (thinner along the z-dimension), and particle-shaped masks, respectively. The fifth column displays the 3D projections of the enhanced and IsoNet missing-wedge-corrected particle. The sixth and seventh columns present the final 3D map and the flexibly fitted model, respectively. (B) Two random cross-sectional views (12 nm thickness) of the plasmid density map along its plectoneme axis are shown in the left-middle panel. The intensity profile along the line crossing the two high-density DNA spots is displayed in the right panel. (C) Resolution assessment of the final 3D map using Fourier shell correlation (FSC). Two criteria are shown: FSC between two half-maps reconstructed from even and odd frames (evaluated at 0.143) and FSC between the final 3D map and the fitted model (evaluated at 0.5). (D) Zoomed-in views of the final 3D density map from panel A, displayed at two contour levels. (E) Superimposition of the high-contour level map from panel D onto its flexibly fitted model.

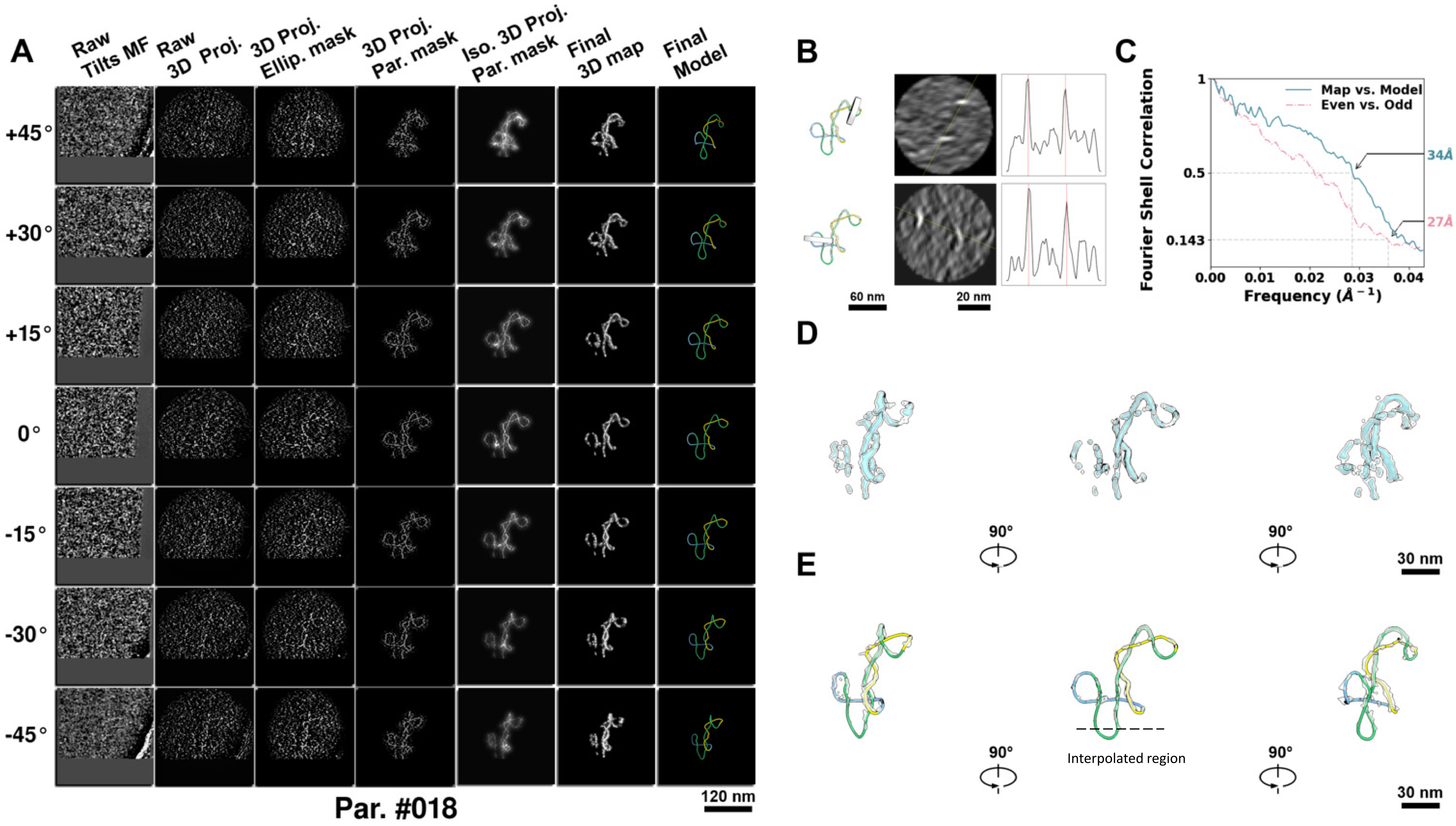

Supplementary Figure 18. Cryo-electron tomography (cryo-ET) 3D reconstruction of an individual plasmid particle in 5 mM K⁺.
(A) 3D reconstruction of the plasmid particle (index no. 18). The first column shows seven representative tilt images from –45° to +45° in step of 15°. The second, third, and fourth columns show 3D projections of the particle with spherical, ellipsoidal (thinner along the z-dimension), and particle-shaped masks, respectively. The fifth column displays the 3D projections of the enhanced and IsoNet missing-wedge-corrected particle. The sixth and seventh columns present the final 3D map and the flexibly fitted model, respectively. (B) Two random cross-sectional views (12 nm thickness) of the plasmid density map along its plectoneme axis are shown in the left-middle panel. The intensity profile along the line crossing the two high-density DNA spots is displayed in the right panel. (C) Resolution assessment of the final 3D map using Fourier shell correlation (FSC). Two criteria are shown: FSC between two half-maps reconstructed from even and odd frames (evaluated at 0.143) and FSC between the final 3D map and the fitted model (evaluated at 0.5). (D) Zoomed-in views of the final 3D density map from panel A, displayed at two contour levels. (E) Superimposition of the high-contour level map from panel D onto its flexibly fitted model.

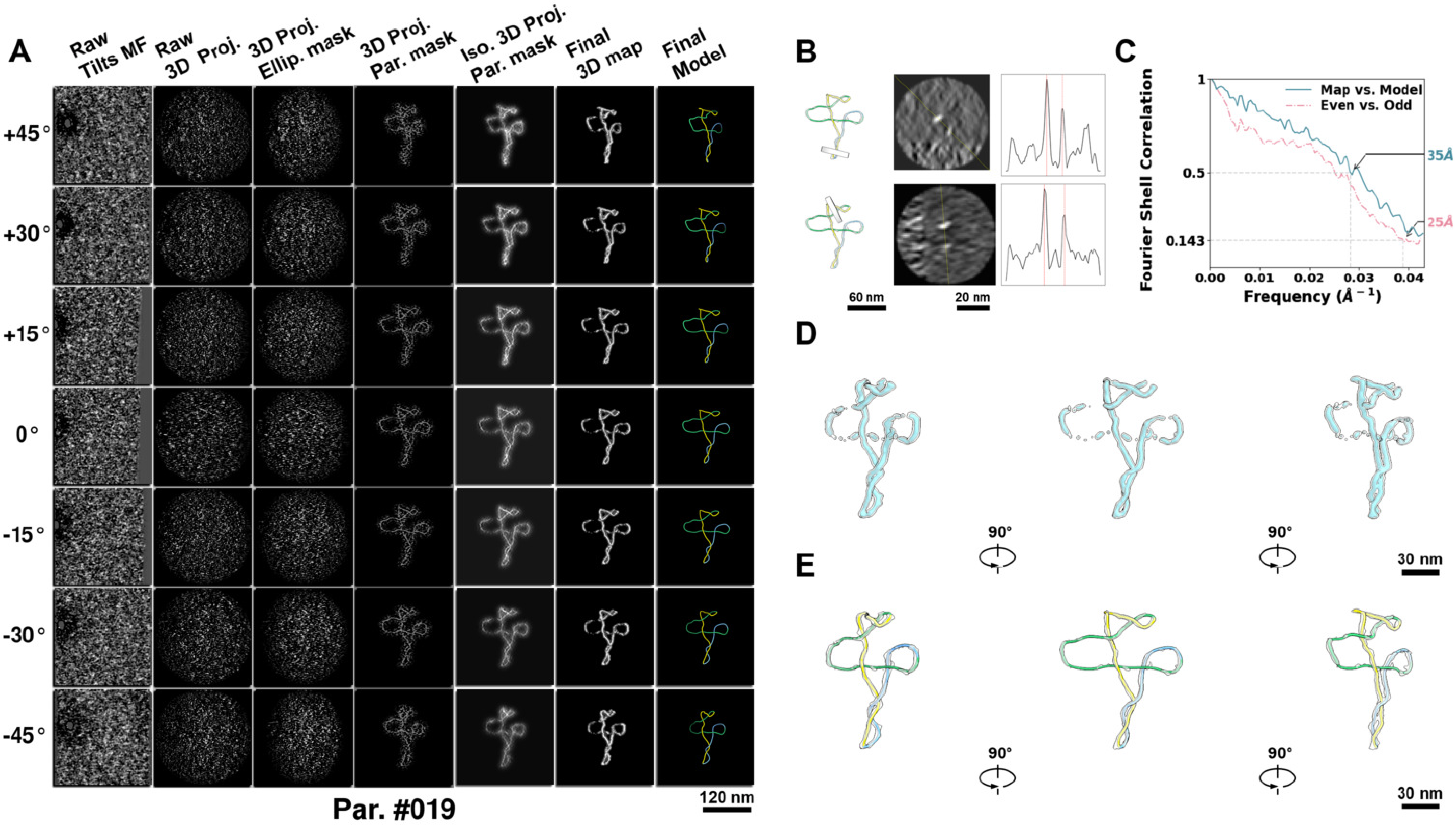

Supplementary Figure 19. Cryo-electron tomography (cryo-ET) 3D reconstruction of an individual plasmid particle in 5 mM K⁺. (A) 3D reconstruction of the plasmid particle (index no. 19). The first column shows seven representative tilt images from –45° to +45° in step of 15°. The second, third, and fourth columns show 3D projections of the particle with spherical, ellipsoidal (thinner along the z-dimension), and particle-shaped masks, respectively. The fifth column displays the 3D projections of the enhanced and IsoNet missing-wedge-corrected particle. The sixth and seventh columns present the final 3D map and the flexibly fitted model, respectively. (B) Two random cross-sectional views (12 nm thickness) of the plasmid density map along its plectoneme axis are shown in the left-middle panel. The intensity profile along the line crossing the two high-density DNA spots is displayed in the right panel. (C) Resolution assessment of the final 3D map using Fourier shell correlation (FSC). Two criteria are shown: FSC between two half-maps reconstructed from even and odd frames (evaluated at 0.143) and FSC between the final 3D map and the fitted model (evaluated at 0.5). (D) Zoomed-in views of the final 3D density map from panel A, displayed at two contour levels. (E) Superimposition of the high-contour level map from panel D onto its flexibly fitted model.

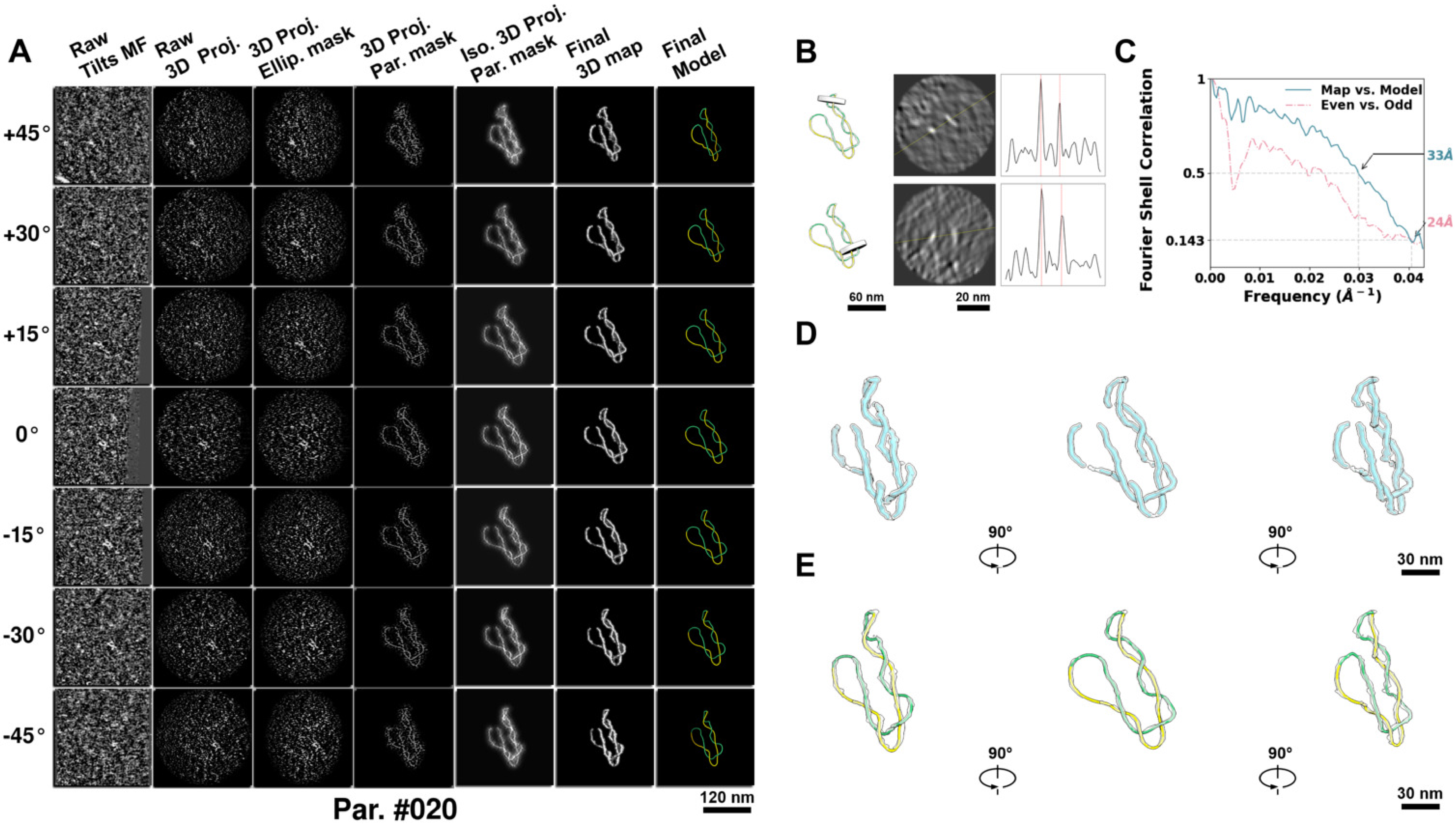

Supplementary Figure 20. Cryo-electron tomography (cryo-ET) 3D reconstruction of an individual plasmid particle in 5 mM K⁺. (A) 3D reconstruction of the plasmid particle (index no. 20). The first column shows seven representative tilt images from –45° to +45° in step of 15°. The second, third, and fourth columns show 3D projections of the particle with spherical, ellipsoidal (thinner along the z-dimension), and particle-shaped masks, respectively. The fifth column displays the 3D projections of the enhanced and IsoNet missing-wedge-corrected particle. The sixth and seventh columns present the final 3D map and the flexibly fitted model, respectively. (B) Two random cross-sectional views (12 nm thickness) of the plasmid density map along its plectoneme axis are shown in the left-middle panel. The intensity profile along the line crossing the two high-density DNA spots is displayed in the right panel. (C) Resolution assessment of the final 3D map using Fourier shell correlation (FSC). Two criteria are shown: FSC between two half-maps reconstructed from even and odd frames (evaluated at 0.143) and FSC between the final 3D map and the fitted model (evaluated at 0.5). (D) Zoomed-in views of the final 3D density map from panel A, displayed at two contour levels. (E) Superimposition of the high-contour level map from panel D onto its flexibly fitted model.

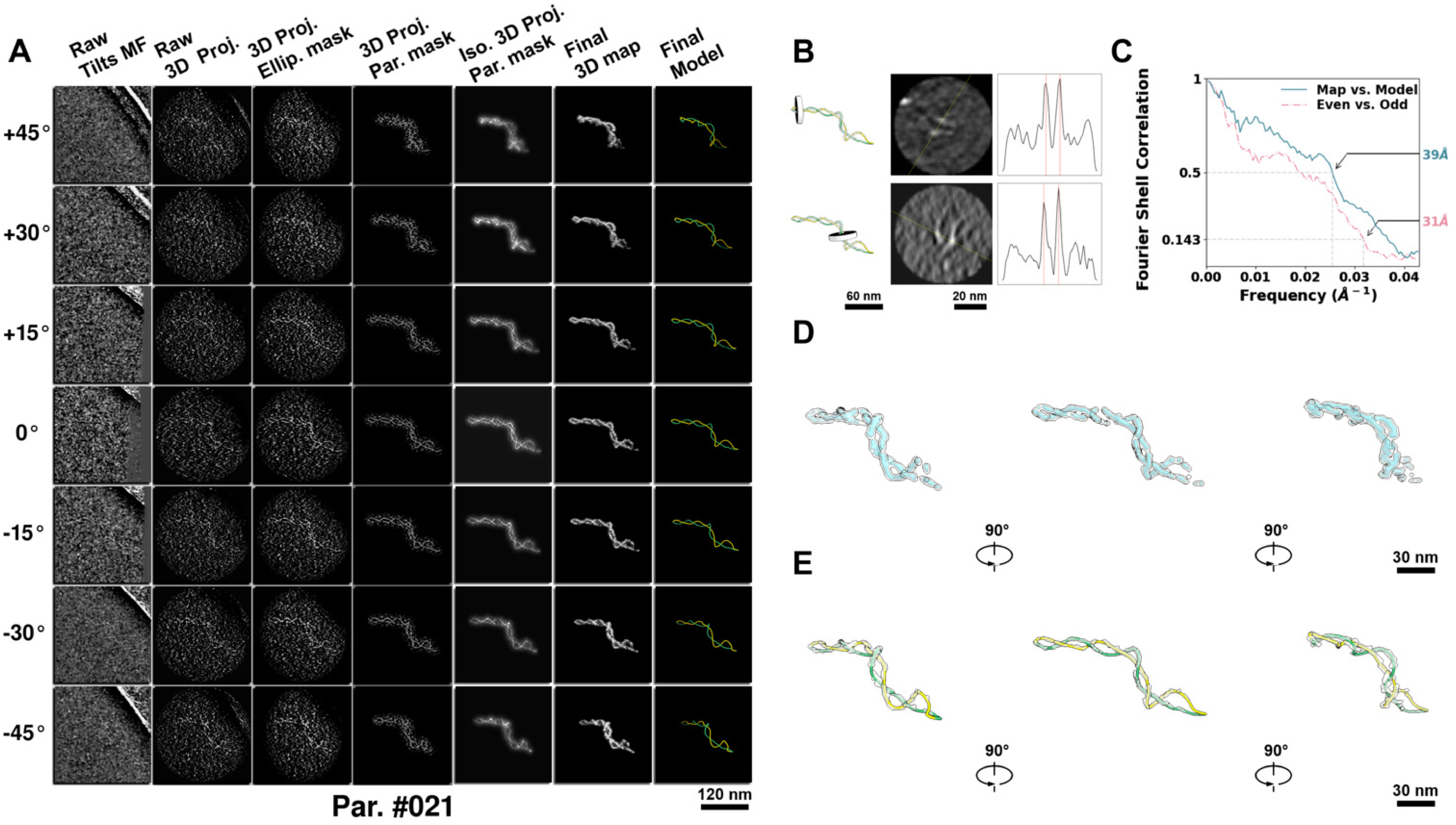

Supplementary Figure 21. Cryo-electron tomography (cryo-ET) 3D reconstruction of an individual plasmid particle in 5 mM K⁺. (A) 3D reconstruction of the plasmid particle (index no. 21). The first column shows seven representative tilt images from –45° to +45° in step of 15°. The second, third, and fourth columns show 3D projections of the particle with spherical, ellipsoidal (thinner along the z-dimension), and particle-shaped masks, respectively. The fifth column displays the 3D projections of the enhanced and IsoNet missing-wedge-corrected particle. The sixth and seventh columns present the final 3D map and the flexibly fitted model, respectively. (B) Two random cross-sectional views (12 nm thickness) of the plasmid density map along its plectoneme axis are shown in the left-middle panel. The intensity profile along the line crossing the two high-density DNA spots is displayed in the right panel. (C) Resolution assessment of the final 3D map using Fourier shell correlation (FSC). Two criteria are shown: FSC between two half-maps reconstructed from even and odd frames (evaluated at 0.143) and FSC between the final 3D map and the fitted model (evaluated at 0.5). (D) Zoomed-in views of the final 3D density map from panel A, displayed at two contour levels. (E) Superimposition of the high-contour level map from panel D onto its flexibly fitted model.

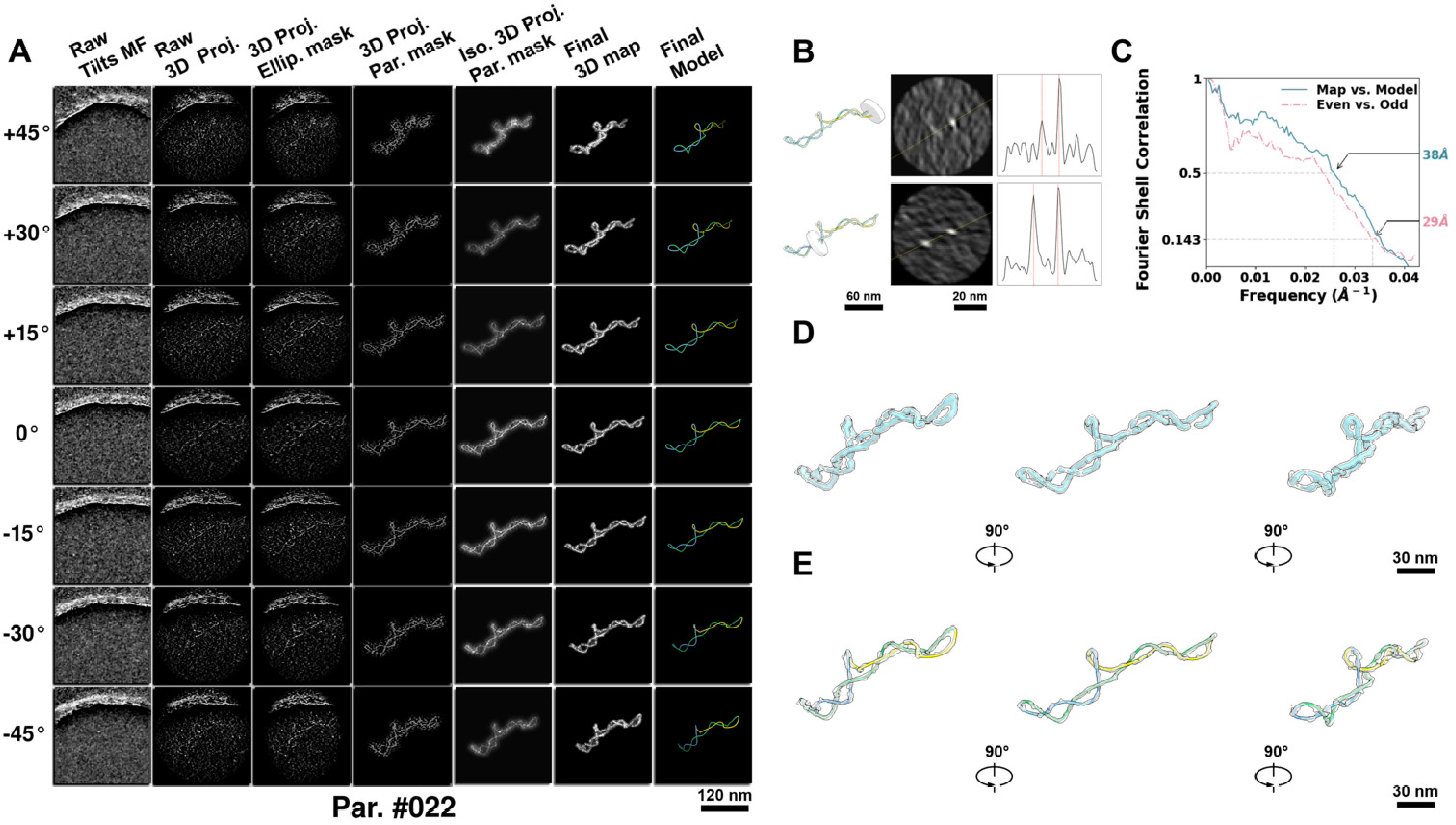

Supplementary Figure 22. Cryo-electron tomography (cryo-ET) 3D reconstruction of an individual plasmid particle in 5 mM K⁺. (A) 3D reconstruction of the plasmid particle (index no. 22). The first column shows seven representative tilt images from –45° to +45° in step of 15°. The second, third, and fourth columns show 3D projections of the particle with spherical, ellipsoidal (thinner along the z-dimension), and particle-shaped masks, respectively. The fifth column displays the 3D projections of the enhanced and IsoNet missing-wedge-corrected particle. The sixth and seventh columns present the final 3D map and the flexibly fitted model, respectively. (B) Two random cross-sectional views (12 nm thickness) of the plasmid density map along its plectoneme axis are shown in the left-middle panel. The intensity profile along the line crossing the two high-density DNA spots is displayed in the right panel. (C) Resolution assessment of the final 3D map using Fourier shell correlation (FSC). Two criteria are shown: FSC between two half-maps reconstructed from even and odd frames (evaluated at 0.143) and FSC between the final 3D map and the fitted model (evaluated at 0.5). (D) Zoomed-in views of the final 3D density map from panel A, displayed at two contour levels. (E) Superimposition of the high-contour level map from panel D onto its flexibly fitted model.

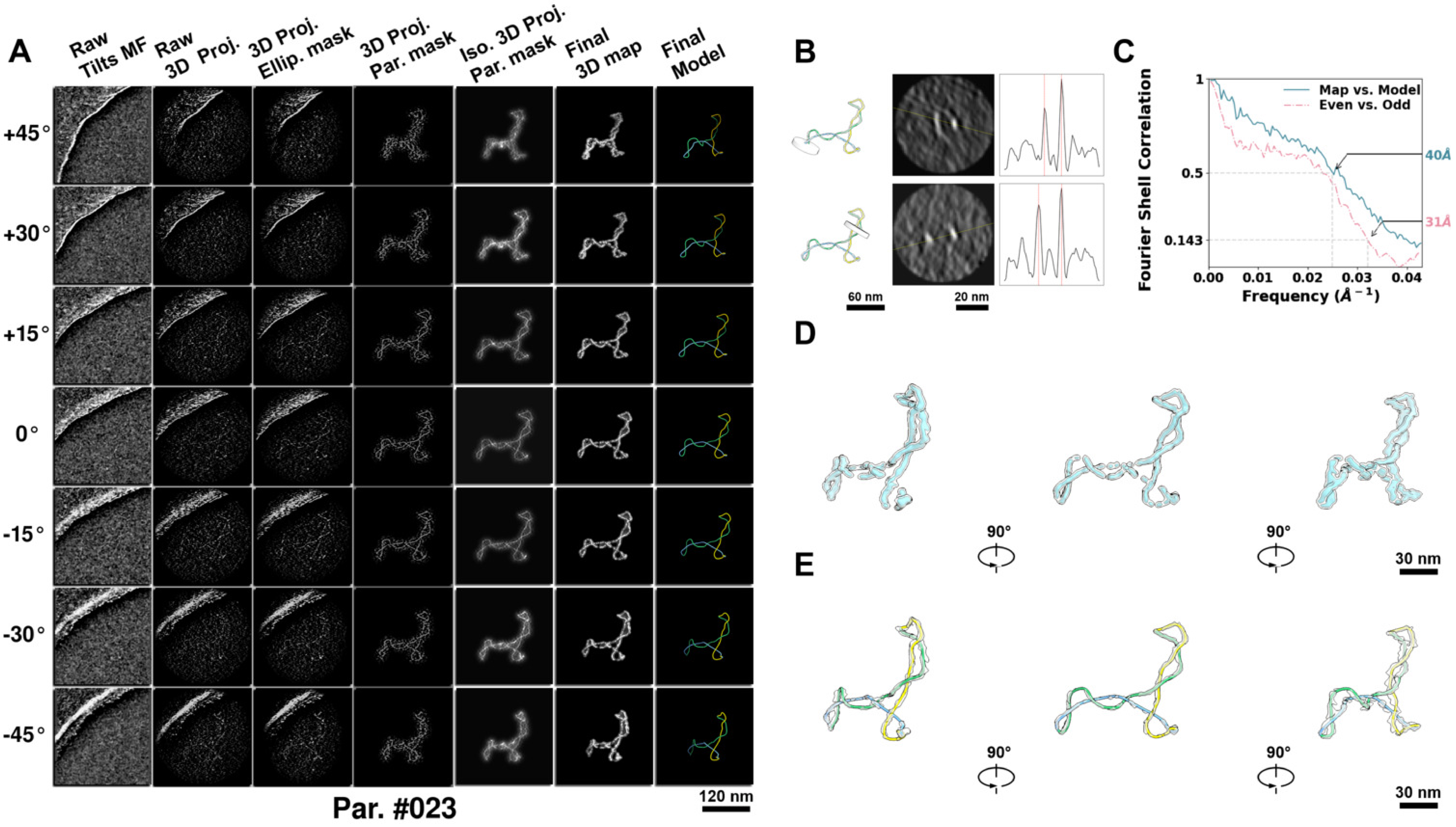

Supplementary Figure 23. Cryo-electron tomography (cryo-ET) 3D reconstruction of an individual plasmid particle in 5 mM K⁺. (A) 3D reconstruction of the plasmid particle (index no. 23). The first column shows seven representative tilt images from –45° to +45° in step of 15°. The second, third, and fourth columns show 3D projections of the particle with spherical, ellipsoidal (thinner along the z-dimension), and particle-shaped masks, respectively. The fifth column displays the 3D projections of the enhanced and IsoNet missing-wedge-corrected particle. The sixth and seventh columns present the final 3D map and the flexibly fitted model, respectively. (B) Two random cross-sectional views (12 nm thickness) of the plasmid density map along its plectoneme axis are shown in the left-middle panel. The intensity profile along the line crossing the two high-density DNA spots is displayed in the right panel. (C) Resolution assessment of the final 3D map using Fourier shell correlation (FSC). Two criteria are shown: FSC between two half-maps reconstructed from even and odd frames (evaluated at 0.143) and FSC between the final 3D map and the fitted model (evaluated at 0.5). (D) Zoomed-in views of the final 3D density map from panel A, displayed at two contour levels. (E) Superimposition of the high-contour level map from panel D onto its flexibly fitted model.

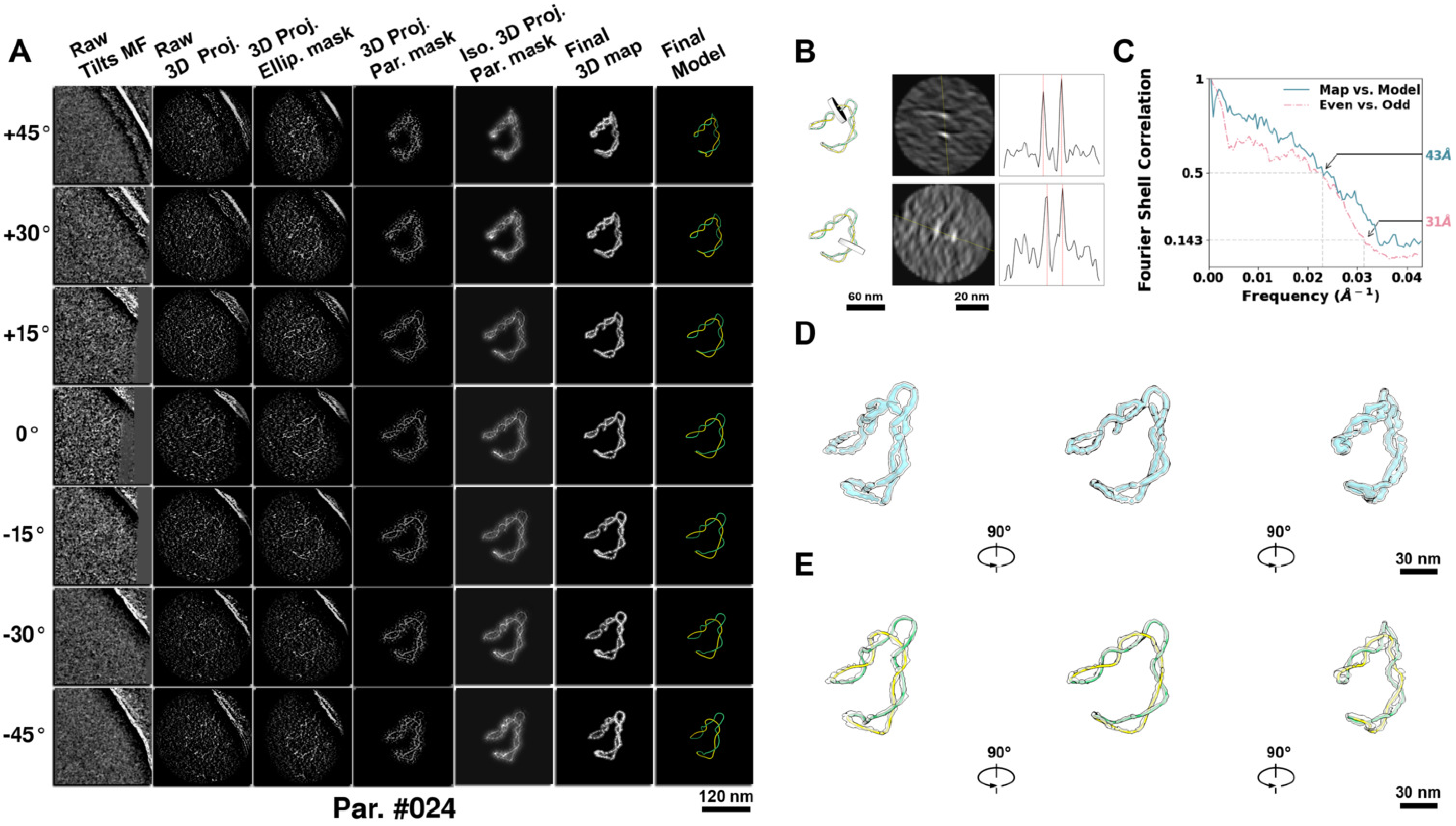

Supplementary Figure 24. Cryo-electron tomography (cryo-ET) 3D reconstruction of an individual plasmid particle in 5 mM K⁺. (A) 3D reconstruction of the plasmid particle (index no. 24). The first column shows seven representative tilt images from –45° to +45° in step of 15°. The second, third, and fourth columns show 3D projections of the particle with spherical, ellipsoidal (thinner along the z-dimension), and particle-shaped masks, respectively. The fifth column displays the 3D projections of the enhanced and IsoNet missing-wedge-corrected particle. The sixth and seventh columns present the final 3D map and the flexibly fitted model, respectively. (B) Two random cross-sectional views (12 nm thickness) of the plasmid density map along its plectoneme axis are shown in the left-middle panel. The intensity profile along the line crossing the two high-density DNA spots is displayed in the right panel. (C) Resolution assessment of the final 3D map using Fourier shell correlation (FSC). Two criteria are shown: FSC between two half-maps reconstructed from even and odd frames (evaluated at 0.143) and FSC between the final 3D map and the fitted model (evaluated at 0.5). (D) Zoomed-in views of the final 3D density map from panel A, displayed at two contour levels. (E) Superimposition of the high-contour level map from panel D onto its flexibly fitted model.

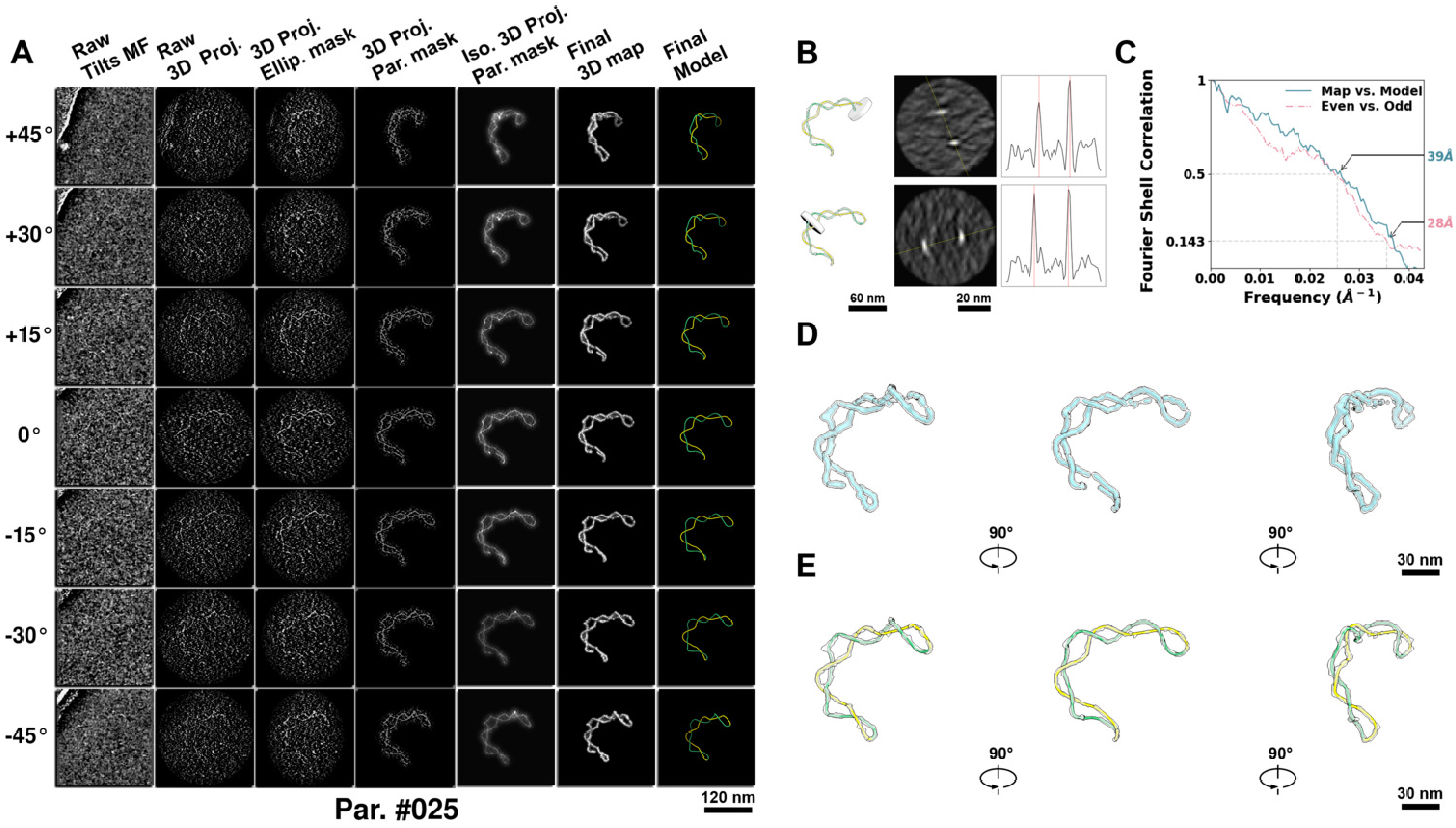

Supplementary Figure 25. Cryo-electron tomography (cryo-ET) 3D reconstruction of an individual plasmid particle in 5 mM K⁺. (A) 3D reconstruction of the plasmid particle (index no. 25). The first column shows seven representative tilt images from –45° to +45° in step of 15°. The second, third, and fourth columns show 3D projections of the particle with spherical, ellipsoidal (thinner along the z-dimension), and particle-shaped masks, respectively. The fifth column displays the 3D projections of the enhanced and IsoNet missing-wedge-corrected particle. The sixth and seventh columns present the final 3D map and the flexibly fitted model, respectively. (B) Two random cross-sectional views (12 nm thickness) of the plasmid density map along its plectoneme axis are shown in the left-middle panel. The intensity profile along the line crossing the two high-density DNA spots is displayed in the right panel. (C) Resolution assessment of the final 3D map using Fourier shell correlation (FSC). Two criteria are shown: FSC between two half-maps reconstructed from even and odd frames (evaluated at 0.143) and FSC between the final 3D map and the fitted model (evaluated at 0.5). (D) Zoomed-in views of the final 3D density map from panel A, displayed at two contour levels. (E) Superimposition of the high-contour level map from panel D onto its flexibly fitted model.

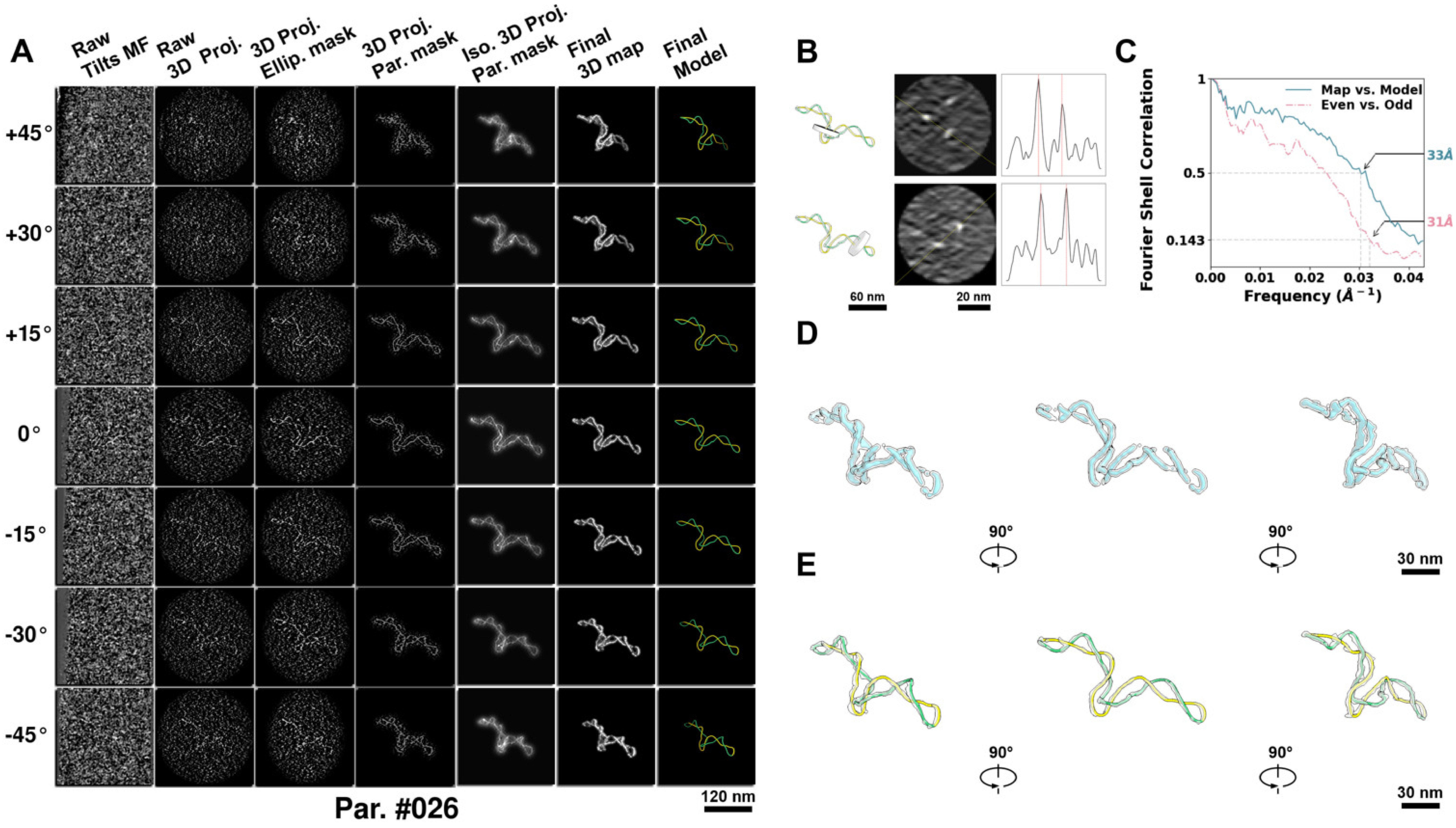

Supplementary Figure 26. Cryo-electron tomography (cryo-ET) 3D reconstruction of an individual plasmid particle in 5 mM K⁺. (A) 3D reconstruction of the plasmid particle (index no. 26). The first column shows seven representative tilt images from –45° to +45° in step of 15°. The second, third, and fourth columns show 3D projections of the particle with spherical, ellipsoidal (thinner along the z-dimension), and particle-shaped masks, respectively. The fifth column displays the 3D projections of the enhanced and IsoNet missing-wedge-corrected particle. The sixth and seventh columns present the final 3D map and the flexibly fitted model, respectively. (B) Two random cross-sectional views (12 nm thickness) of the plasmid density map along its plectoneme axis are shown in the left-middle panel. The intensity profile along the line crossing the two high-density DNA spots is displayed in the right panel. (C) Resolution assessment of the final 3D map using Fourier shell correlation (FSC). Two criteria are shown: FSC between two half-maps reconstructed from even and odd frames (evaluated at 0.143) and FSC between the final 3D map and the fitted model (evaluated at 0.5). (D) Zoomed-in views of the final 3D density map from panel A, displayed at two contour levels. (E) Superimposition of the high-contour level map from panel D onto its flexibly fitted model.

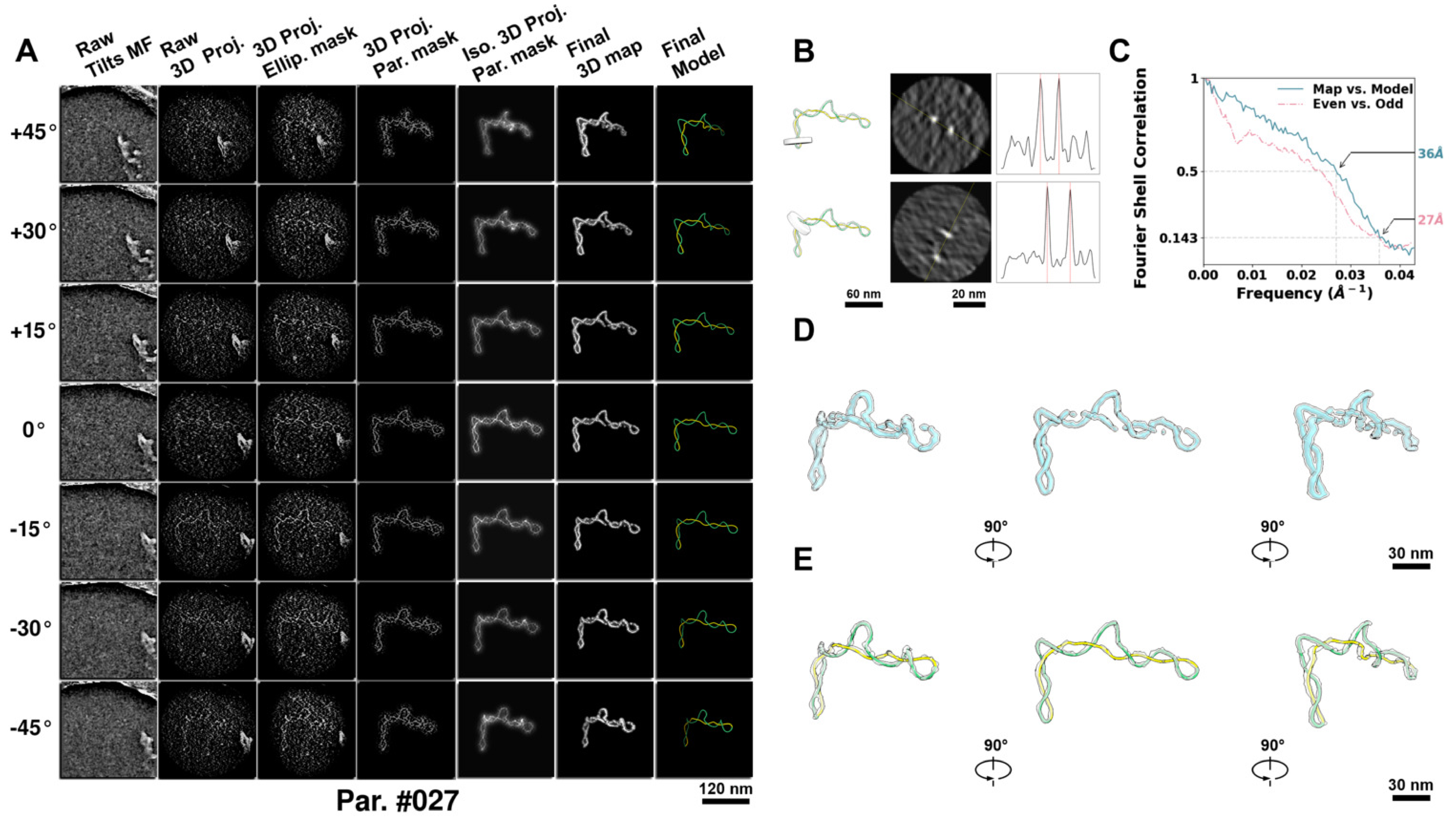

Supplementary Figure 27. Cryo-electron tomography (cryo-ET) 3D reconstruction of an individual plasmid particle in 5 mM K⁺. (A) 3D reconstruction of the plasmid particle (index no. 27). The first column shows seven representative tilt images from –45° to +45° in step of 15°. The second, third, and fourth columns show 3D projections of the particle with spherical, ellipsoidal (thinner along the z-dimension), and particle-shaped masks, respectively. The fifth column displays the 3D projections of the enhanced and IsoNet missing-wedge-corrected particle. The sixth and seventh columns present the final 3D map and the flexibly fitted model, respectively. (B) Two random cross-sectional views (12 nm thickness) of the plasmid density map along its plectoneme axis are shown in the left-middle panel. The intensity profile along the line crossing the two high-density DNA spots is displayed in the right panel. (C) Resolution assessment of the final 3D map using Fourier shell correlation (FSC). Two criteria are shown: FSC between two half-maps reconstructed from even and odd frames (evaluated at 0.143) and FSC between the final 3D map and the fitted model (evaluated at 0.5). (D) Zoomed-in views of the final 3D density map from panel A, displayed at two contour levels. (E) Superimposition of the high-contour level map from panel D onto its flexibly fitted model.

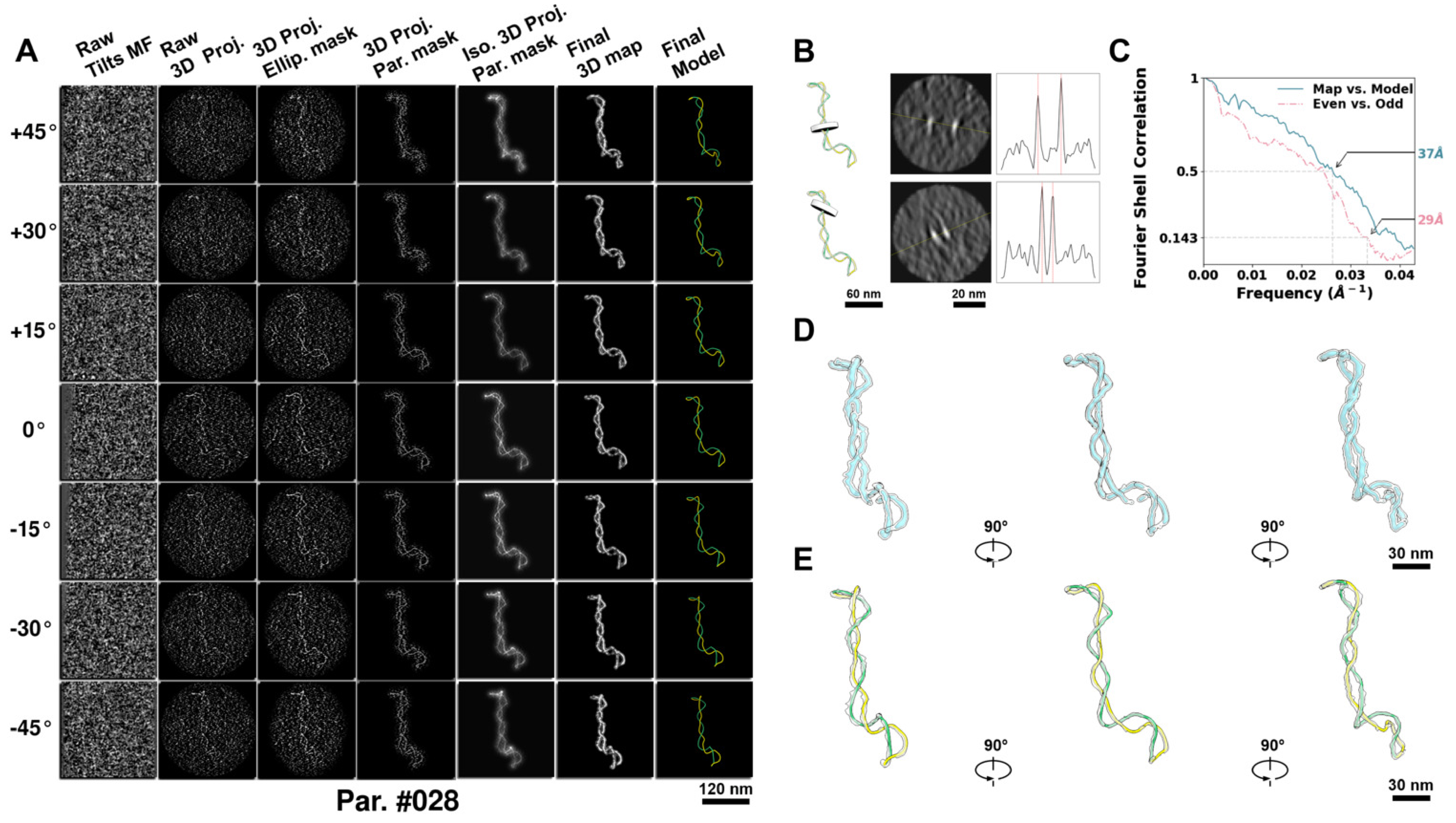

Supplementary Figure 28. Cryo-electron tomography (cryo-ET) 3D reconstruction of an individual plasmid particle in 5 mM K⁺. (A) 3D reconstruction of the plasmid particle (index no. 28). The first column shows seven representative tilt images from –45° to +45° in step of 15°. The second, third, and fourth columns show 3D projections of the particle with spherical, ellipsoidal (thinner along the z-dimension), and particle-shaped masks, respectively. The fifth column displays the 3D projections of the enhanced and IsoNet missing-wedge-corrected particle. The sixth and seventh columns present the final 3D map and the flexibly fitted model, respectively. (B) Two random cross-sectional views (12 nm thickness) of the plasmid density map along its plectoneme axis are shown in the left-middle panel. The intensity profile along the line crossing the two high-density DNA spots is displayed in the right panel. (C) Resolution assessment of the final 3D map using Fourier shell correlation (FSC). Two criteria are shown: FSC between two half-maps reconstructed from even and odd frames (evaluated at 0.143) and FSC between the final 3D map and the fitted model (evaluated at 0.5). (D) Zoomed-in views of the final 3D density map from panel A, displayed at two contour levels. (E) Superimposition of the high-contour level map from panel D onto its flexibly fitted model.

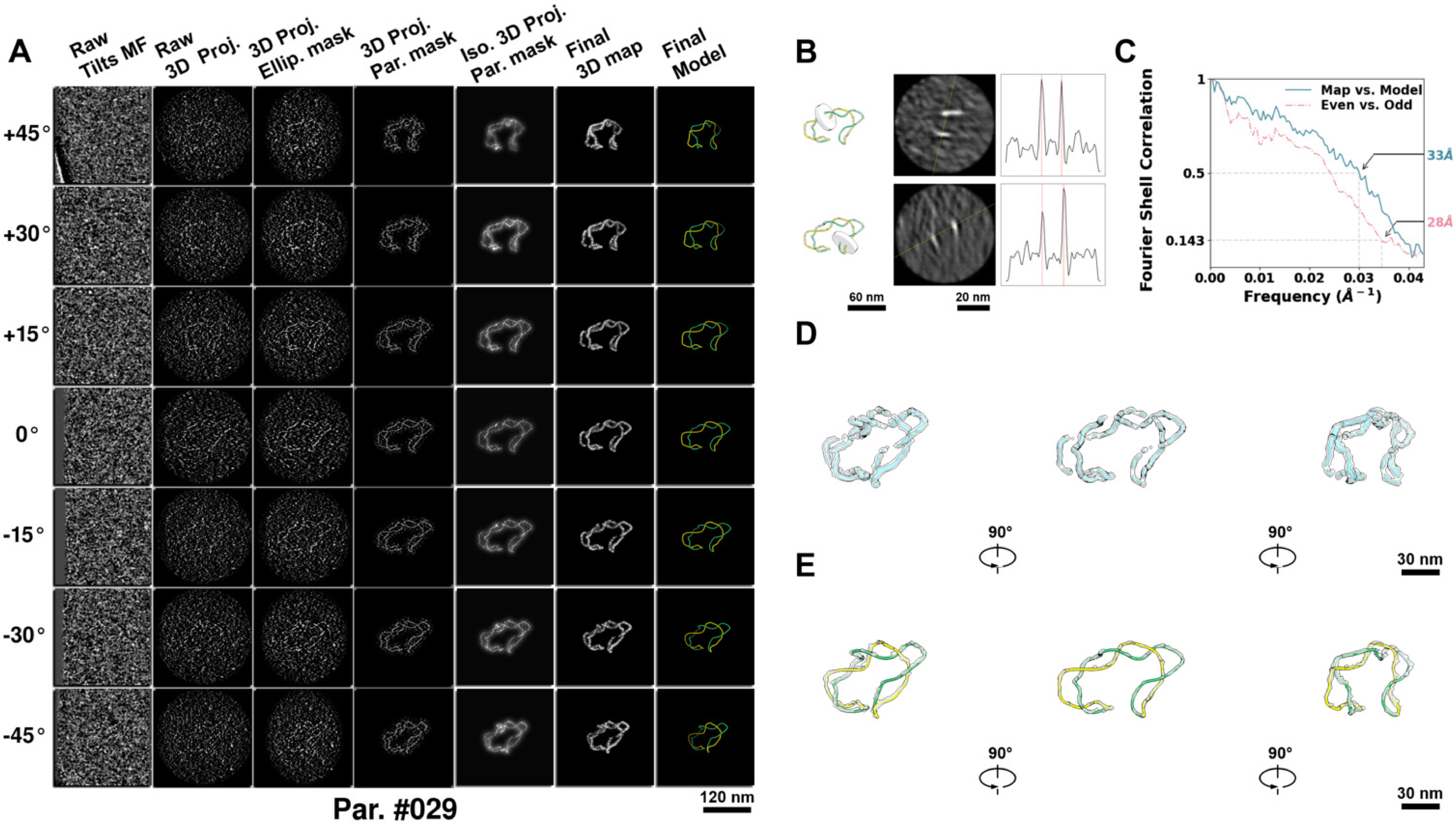

Supplementary Figure 29. Cryo-electron tomography (cryo-ET) 3D reconstruction of an individual plasmid particle in 5 mM K⁺.
(A) 3D reconstruction of the plasmid particle (index no. 29). The first column shows seven representative tilt images from –45° to +45° in step of 15°. The second, third, and fourth columns show 3D projections of the particle with spherical, ellipsoidal (thinner along the z-dimension), and particle-shaped masks, respectively. The fifth column displays the 3D projections of the enhanced and IsoNet missing-wedge-corrected particle. The sixth and seventh columns present the final 3D map and the flexibly fitted model, respectively. (B) Two random cross-sectional views (12 nm thickness) of the plasmid density map along its plectoneme axis are shown in the left-middle panel. The intensity profile along the line crossing the two high-density DNA spots is displayed in the right panel. (C) Resolution assessment of the final 3D map using Fourier shell correlation (FSC). Two criteria are shown: FSC between two half-maps reconstructed from even and odd frames (evaluated at 0.143) and FSC between the final 3D map and the fitted model (evaluated at 0.5). (D) Zoomed-in views of the final 3D density map from panel A, displayed at two contour levels. (E) Superimposition of the high-contour level map from panel D onto its flexibly fitted model.

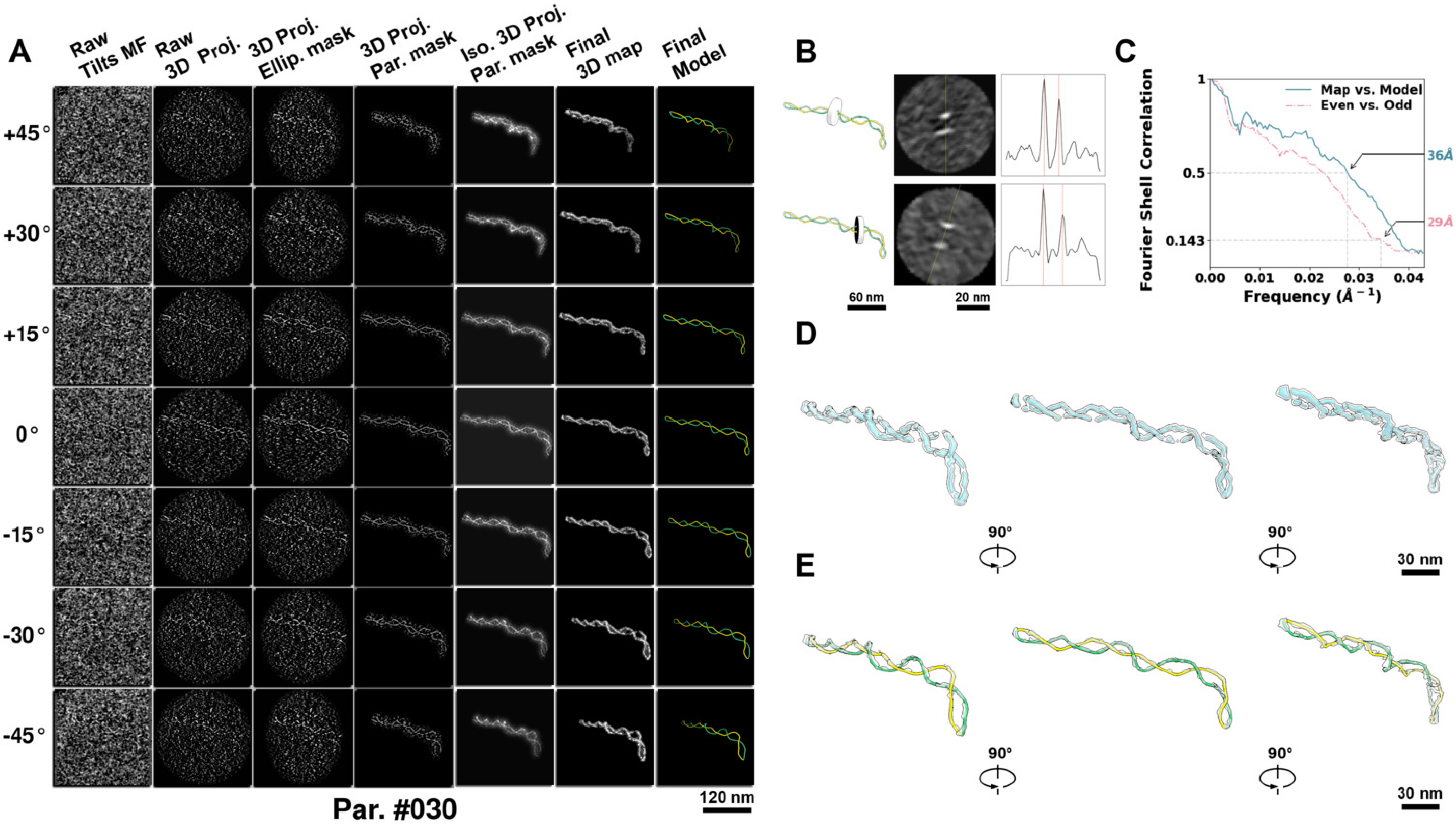

Supplementary Figure 30. Cryo-electron tomography (cryo-ET) 3D reconstruction of an individual plasmid particle in 5 mM K⁺.
(A) 3D reconstruction of the plasmid particle (index no. 30). The first column shows seven representative tilt images from –45° to +45° in step of 15°. The second, third, and fourth columns show 3D projections of the particle with spherical, ellipsoidal (thinner along the z-dimension), and particle-shaped masks, respectively. The fifth column displays the 3D projections of the enhanced and IsoNet missing-wedge-corrected particle. The sixth and seventh columns present the final 3D map and the flexibly fitted model, respectively. (B) Two random cross-sectional views (12 nm thickness) of the plasmid density map along its plectoneme axis are shown in the left-middle panel. The intensity profile along the line crossing the two high-density DNA spots is displayed in the right panel. (C) Resolution assessment of the final 3D map using Fourier shell correlation (FSC). Two criteria are shown: FSC between two half-maps reconstructed from even and odd frames (evaluated at 0.143) and FSC between the final 3D map and the fitted model (evaluated at 0.5). (D) Zoomed-in views of the final 3D density map from panel A, displayed at two contour levels. (E) Superimposition of the high-contour level map from panel D onto its flexibly fitted model.

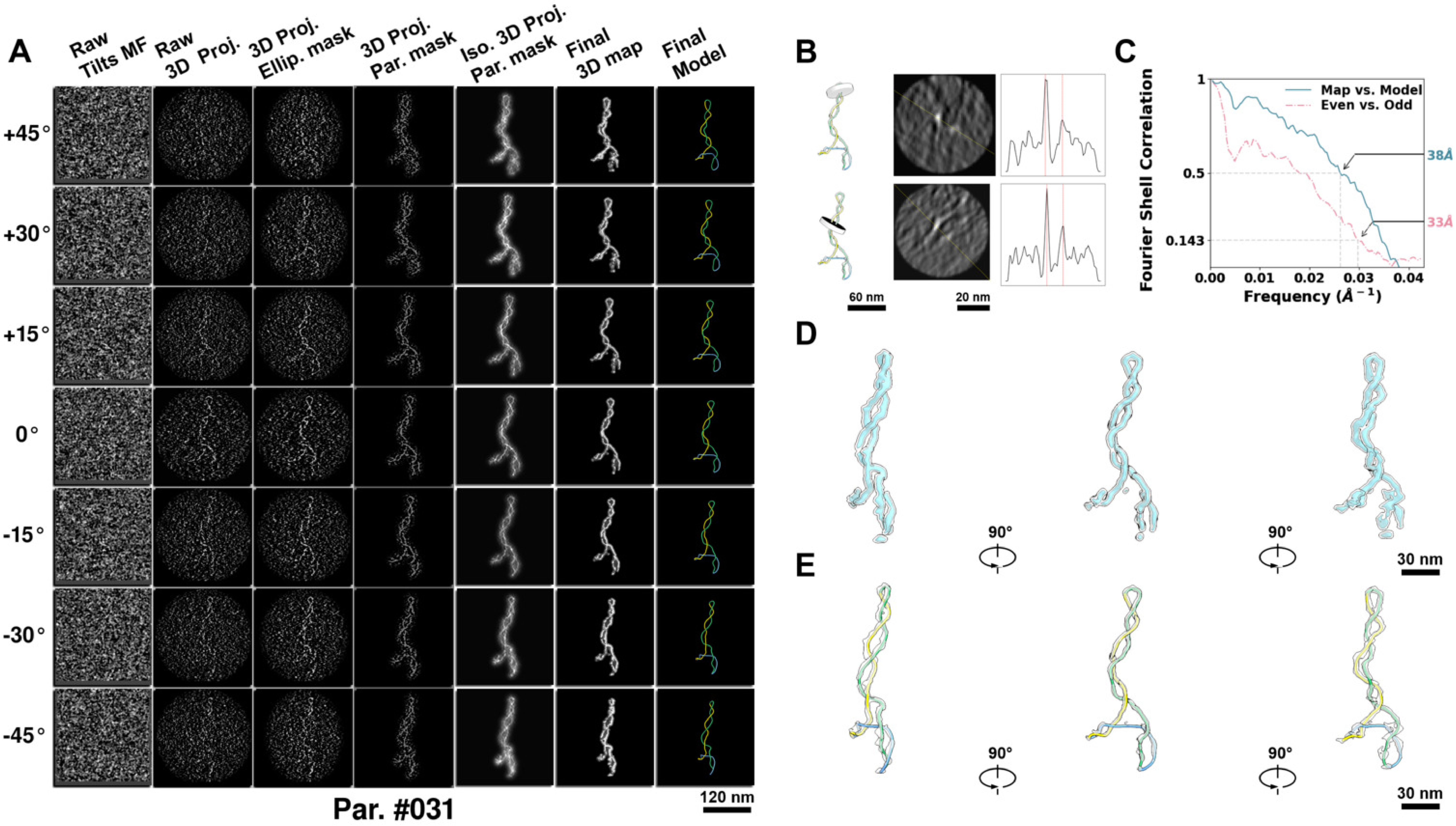

Supplementary Figure 31. Cryo-electron tomography (cryo-ET) 3D reconstruction of an individual plasmid particle in 5 mM K⁺. (A) 3D reconstruction of the plasmid particle (index no. 31). The first column shows seven representative tilt images from –45° to +45° in step of 15°. The second, third, and fourth columns show 3D projections of the particle with spherical, ellipsoidal (thinner along the z-dimension), and particle-shaped masks, respectively. The fifth column displays the 3D projections of the enhanced and IsoNet missing-wedge-corrected particle. The sixth and seventh columns present the final 3D map and the flexibly fitted model, respectively. (B) Two random cross-sectional views (12 nm thickness) of the plasmid density map along its plectoneme axis are shown in the left-middle panel. The intensity profile along the line crossing the two high-density DNA spots is displayed in the right panel. (C) Resolution assessment of the final 3D map using Fourier shell correlation (FSC). Two criteria are shown: FSC between two half-maps reconstructed from even and odd frames (evaluated at 0.143) and FSC between the final 3D map and the fitted model (evaluated at 0.5). (D) Zoomed-in views of the final 3D density map from panel A, displayed at two contour levels. (E) Superimposition of the high-contour level map from panel D onto its flexibly fitted model.

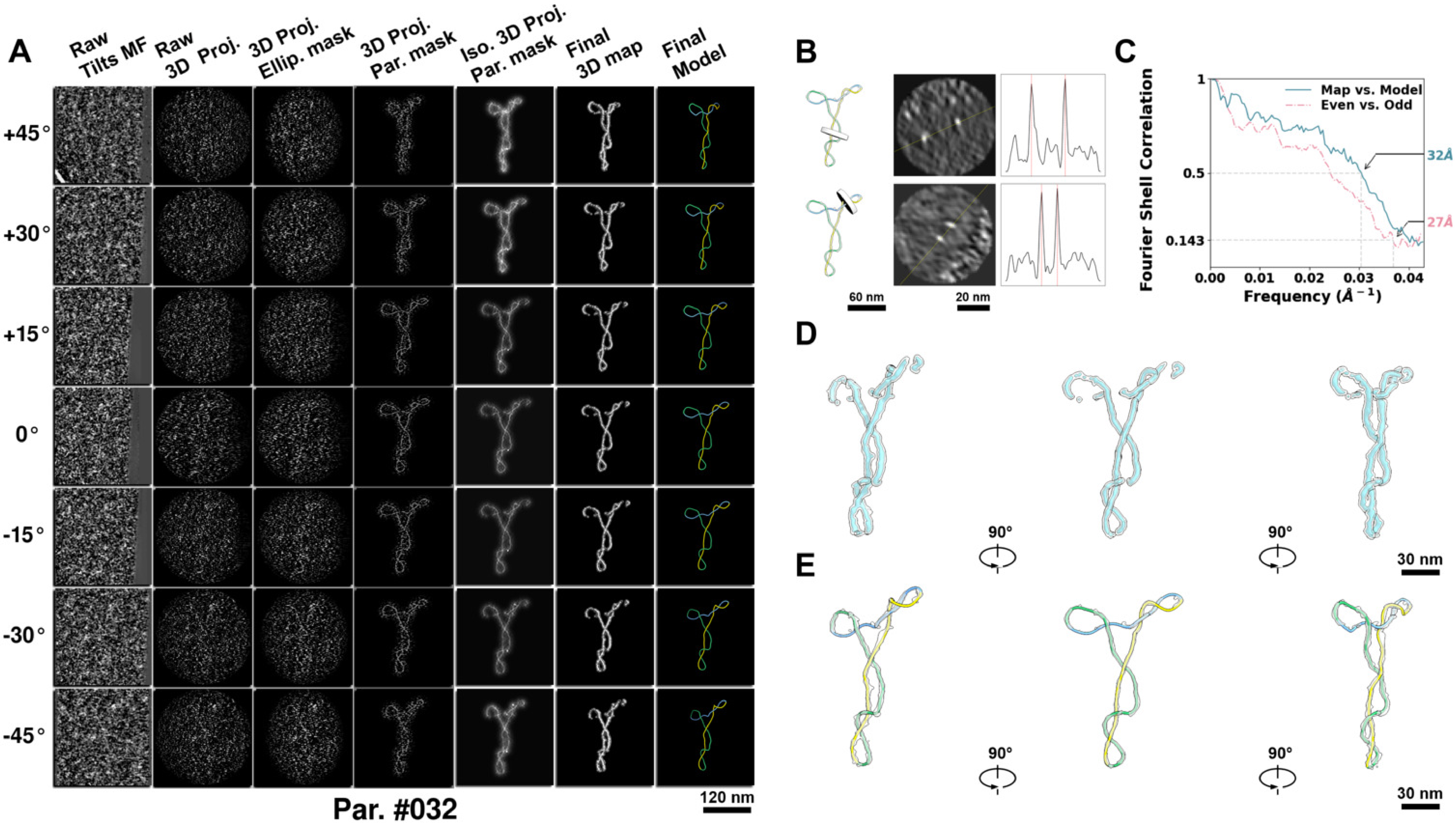

Supplementary Figure 32. Cryo-electron tomography (cryo-ET) 3D reconstruction of an individual plasmid particle in 5 mM K⁺. (A) 3D reconstruction of the plasmid particle (index no. 32). The first column shows seven representative tilt images from –45° to +45° in step of 15°. The second, third, and fourth columns show 3D projections of the particle with spherical, ellipsoidal (thinner along the z-dimension), and particle-shaped masks, respectively. The fifth column displays the 3D projections of the enhanced and IsoNet missing-wedge-corrected particle. The sixth and seventh columns present the final 3D map and the flexibly fitted model, respectively. (B) Two random cross-sectional views (12 nm thickness) of the plasmid density map along its plectoneme axis are shown in the left-middle panel. The intensity profile along the line crossing the two high-density DNA spots is displayed in the right panel. (C) Resolution assessment of the final 3D map using Fourier shell correlation (FSC). Two criteria are shown: FSC between two half-maps reconstructed from even and odd frames (evaluated at 0.143) and FSC between the final 3D map and the fitted model (evaluated at 0.5). (D) Zoomed-in views of the final 3D density map from panel A, displayed at two contour levels. (E) Superimposition of the high-contour level map from panel D onto its flexibly fitted model.

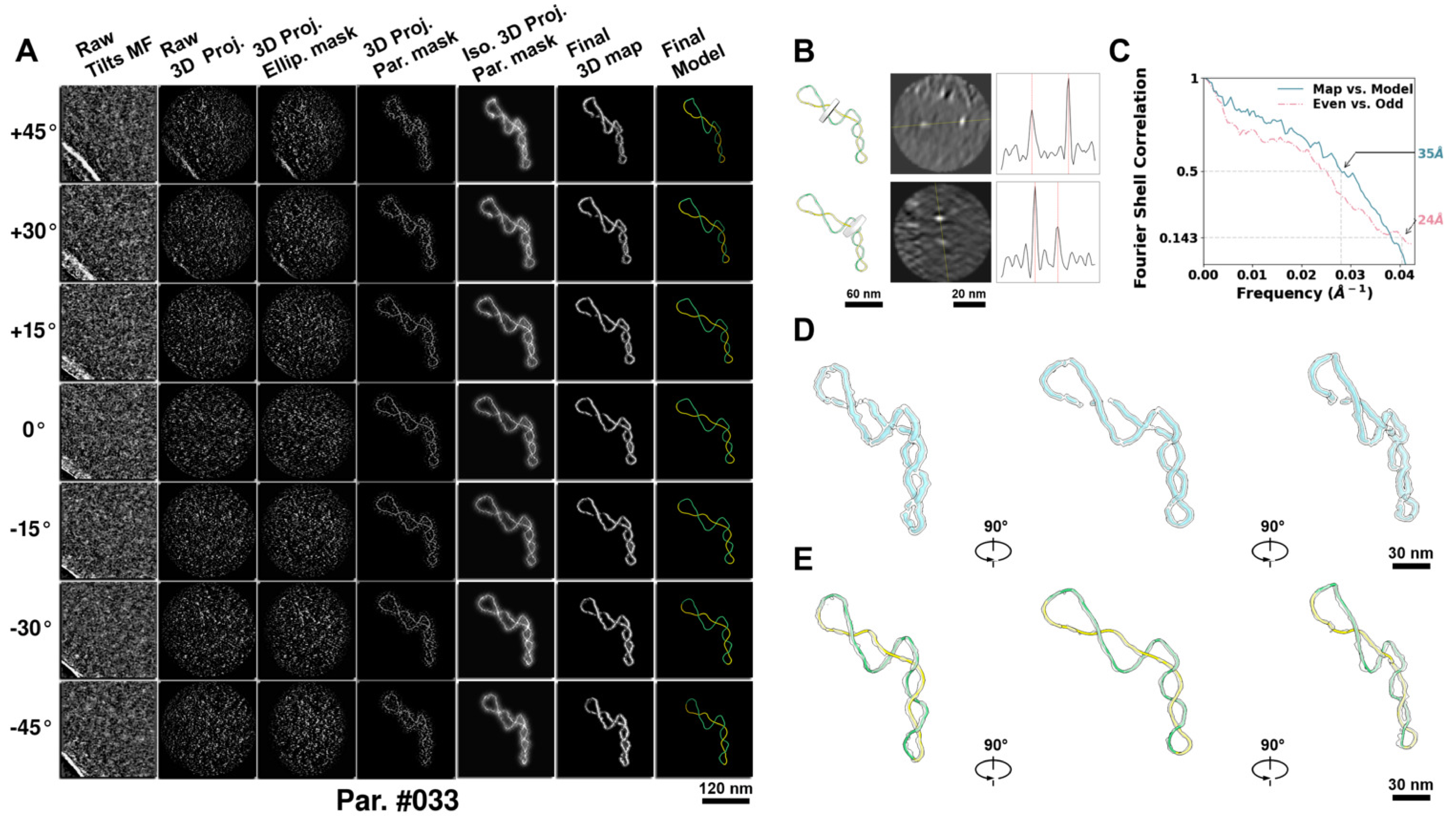

Supplementary Figure 33. Cryo-electron tomography (cryo-ET) 3D reconstruction of an individual plasmid particle in 5 mM K⁺. (A) 3D reconstruction of the plasmid particle (index no. 33). The first column shows seven representative tilt images from –45° to +45° in step of 15°. The second, third, and fourth columns show 3D projections of the particle with spherical, ellipsoidal (thinner along the z-dimension), and particle-shaped masks, respectively. The fifth column displays the 3D projections of the enhanced and IsoNet missing-wedge-corrected particle. The sixth and seventh columns present the final 3D map and the flexibly fitted model, respectively. (B) Two random cross-sectional views (12 nm thickness) of the plasmid density map along its plectoneme axis are shown in the left-middle panel. The intensity profile along the line crossing the two high-density DNA spots is displayed in the right panel. (C) Resolution assessment of the final 3D map using Fourier shell correlation (FSC). Two criteria are shown: FSC between two half-maps reconstructed from even and odd frames (evaluated at 0.143) and FSC between the final 3D map and the fitted model (evaluated at 0.5). (D) Zoomed-in views of the final 3D density map from panel A, displayed at two contour levels. (E) Superimposition of the high-contour level map from panel D onto its flexibly fitted model.

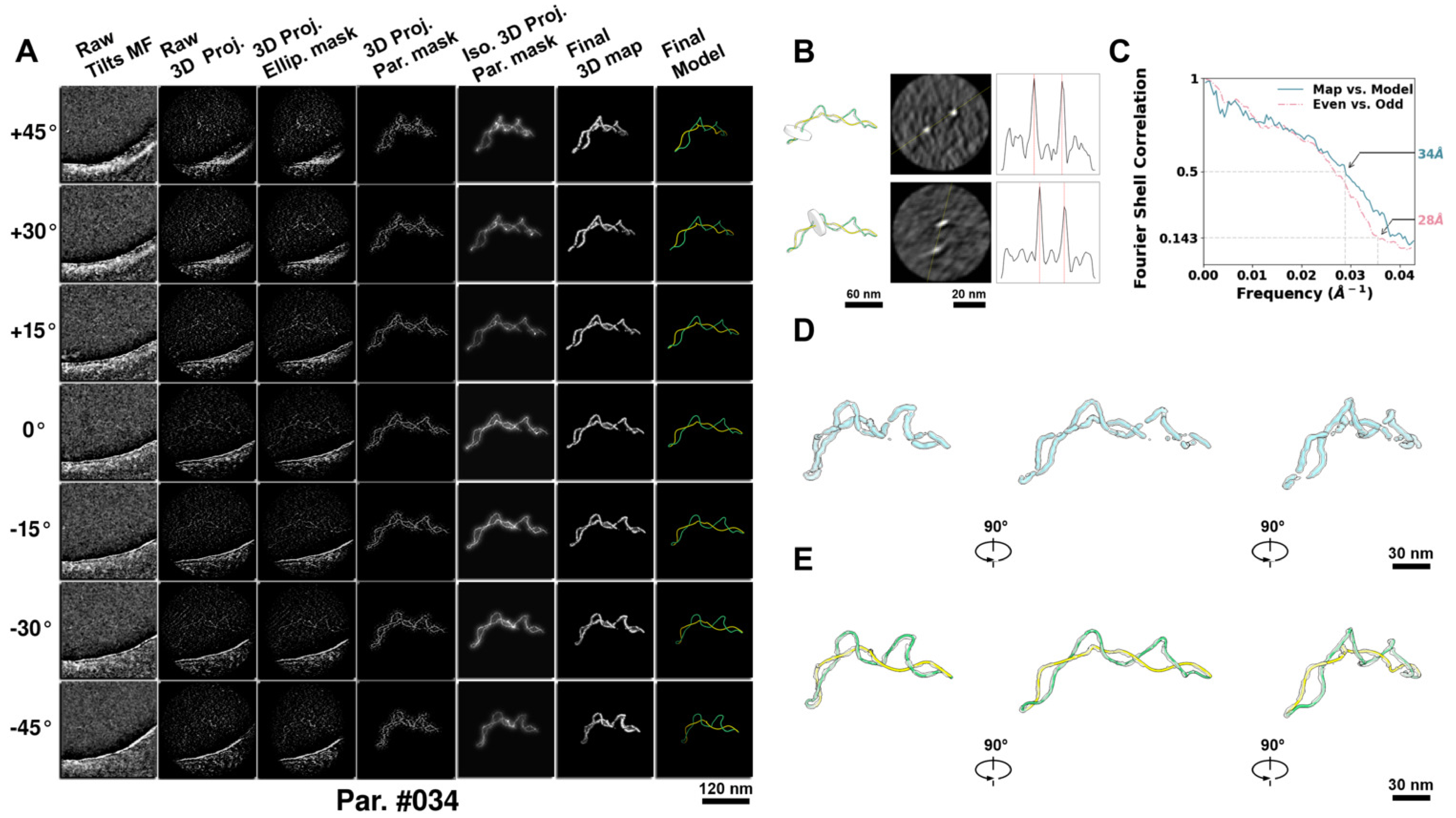

Supplementary Figure 34. Cryo-electron tomography (cryo-ET) 3D reconstruction of an individual plasmid particle in 5 mM K⁺. (A) 3D reconstruction of the plasmid particle (index no. 34). The first column shows seven representative tilt images from –45° to +45° in step of 15°. The second, third, and fourth columns show 3D projections of the particle with spherical, ellipsoidal (thinner along the z-dimension), and particle-shaped masks, respectively. The fifth column displays the 3D projections of the enhanced and IsoNet missing-wedge-corrected particle. The sixth and seventh columns present the final 3D map and the flexibly fitted model, respectively. (B) Two random cross-sectional views (12 nm thickness) of the plasmid density map along its plectoneme axis are shown in the left-middle panel. The intensity profile along the line crossing the two high-density DNA spots is displayed in the right panel. (C) Resolution assessment of the final 3D map using Fourier shell correlation (FSC). Two criteria are shown: FSC between two half-maps reconstructed from even and odd frames (evaluated at 0.143) and FSC between the final 3D map and the fitted model (evaluated at 0.5). (D) Zoomed-in views of the final 3D density map from panel A, displayed at two contour levels. (E) Superimposition of the high-contour level map from panel D onto its flexibly fitted model.

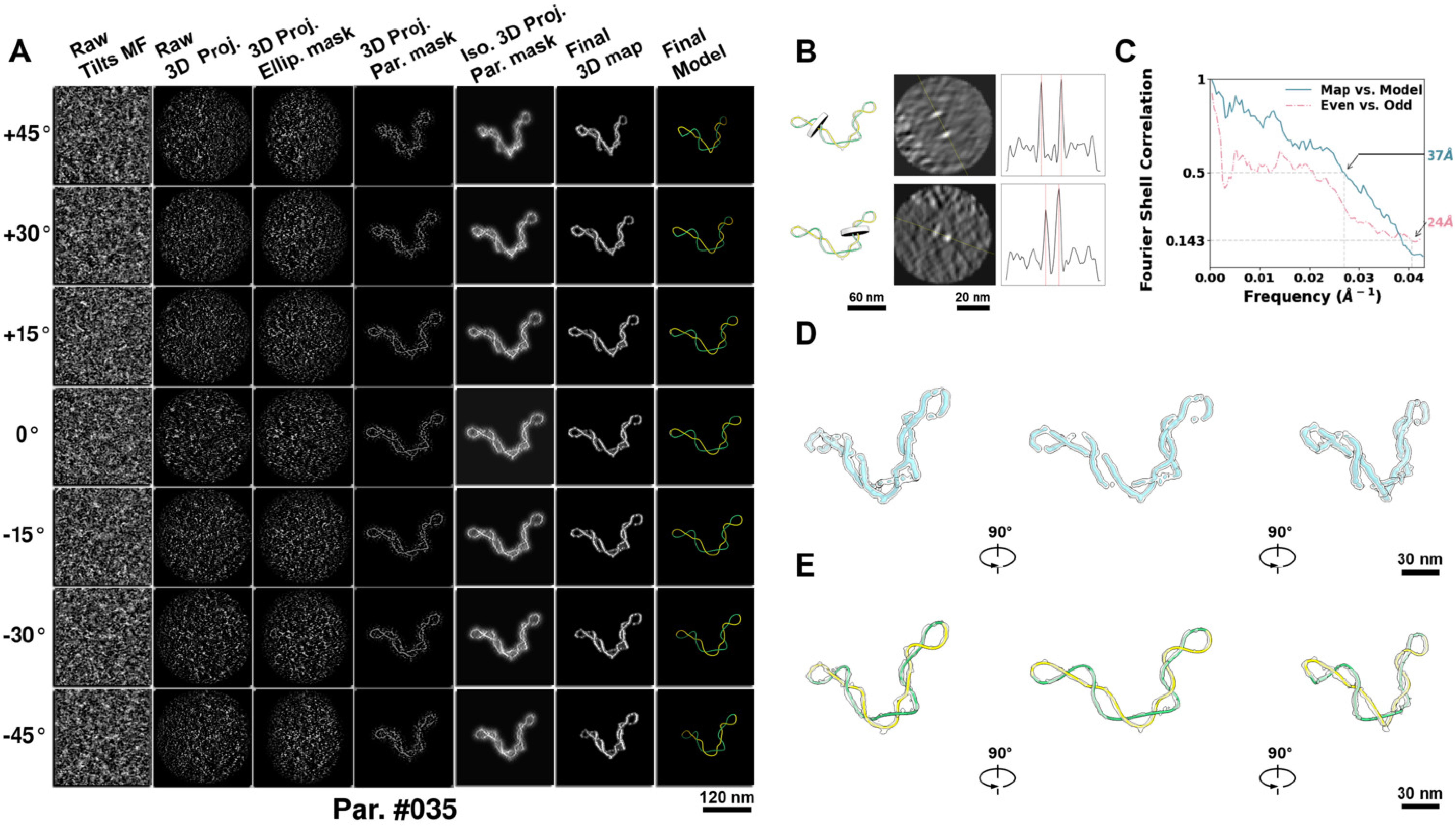

Supplementary Figure 35. Cryo-electron tomography (cryo-ET) 3D reconstruction of an individual plasmid particle in 5 mM K^{+}.
(A) 3D reconstruction of the plasmid particle (index no. 35). The first column shows seven representative tilt images from –45° to +45° in step of 15°. The second, third, and fourth columns show 3D projections of the particle with spherical, ellipsoidal (thinner along the z-dimension), and particle-shaped masks, respectively. The fifth column displays the 3D projections of the enhanced and IsoNet missing-wedge-corrected particle. The sixth and seventh columns present the final 3D map and the flexibly fitted model, respectively. (B) Two random cross-sectional views (12 nm thickness) of the plasmid density map along its plectoneme axis are shown in the left-middle panel. The intensity profile along the line crossing the two high-density DNA spots is displayed in the right panel. (C) Resolution assessment of the final 3D map using Fourier shell correlation (FSC). Two criteria are shown: FSC between two half-maps reconstructed from even and odd frames (evaluated at 0.143) and FSC between the final 3D map and the fitted model (evaluated at 0.5). (D) Zoomed-in views of the final 3D density map from panel A, displayed at two contour levels. (E) Superimposition of the high-contour level map from panel D onto its flexibly fitted model.

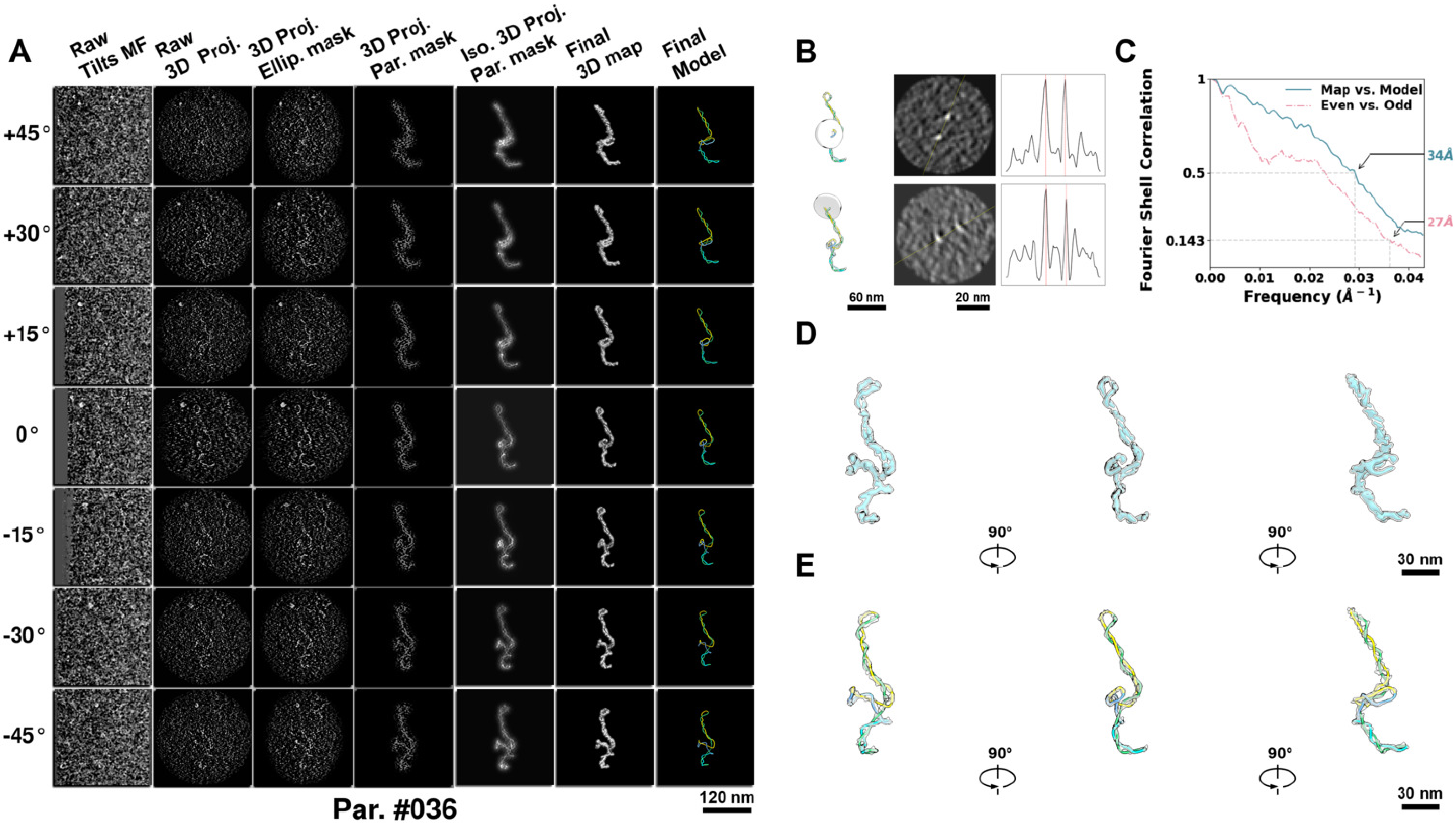

Supplementary Figure 36. Cryo-electron tomography (cryo-ET) 3D reconstruction of an individual plasmid particle in 40 mM K^{+}, 5 mM Mg^{2+}.
(A) 3D reconstruction of the plasmid particle (index no. 36). The first column shows seven representative tilt images from –45° to +45° in step of 15°. The second, third, and fourth columns show 3D projections of the particle with spherical, ellipsoidal (thinner along the z-dimension), and particle-shaped masks, respectively. The fifth column displays the 3D projections of the enhanced and IsoNet missing-wedge-corrected particle. The sixth and seventh columns present the final 3D map and the flexibly fitted model, respectively. (B) Two random cross-sectional views (12 nm thickness) of the plasmid density map along its plectoneme axis are shown in the left-middle panel. The intensity profile along the line crossing the two high-density DNA spots is displayed in the right panel. (C) Resolution assessment of the final 3D map using Fourier shell correlation (FSC). Two criteria are shown: FSC between two half-maps reconstructed from even and odd frames (evaluated at 0.143) and FSC between the final 3D map and the fitted model (evaluated at 0.5). (D) Zoomed-in views of the final 3D density map from panel A, displayed at two contour levels. (E) Superimposition of the high-contour level map from panel D onto its flexibly fitted model.

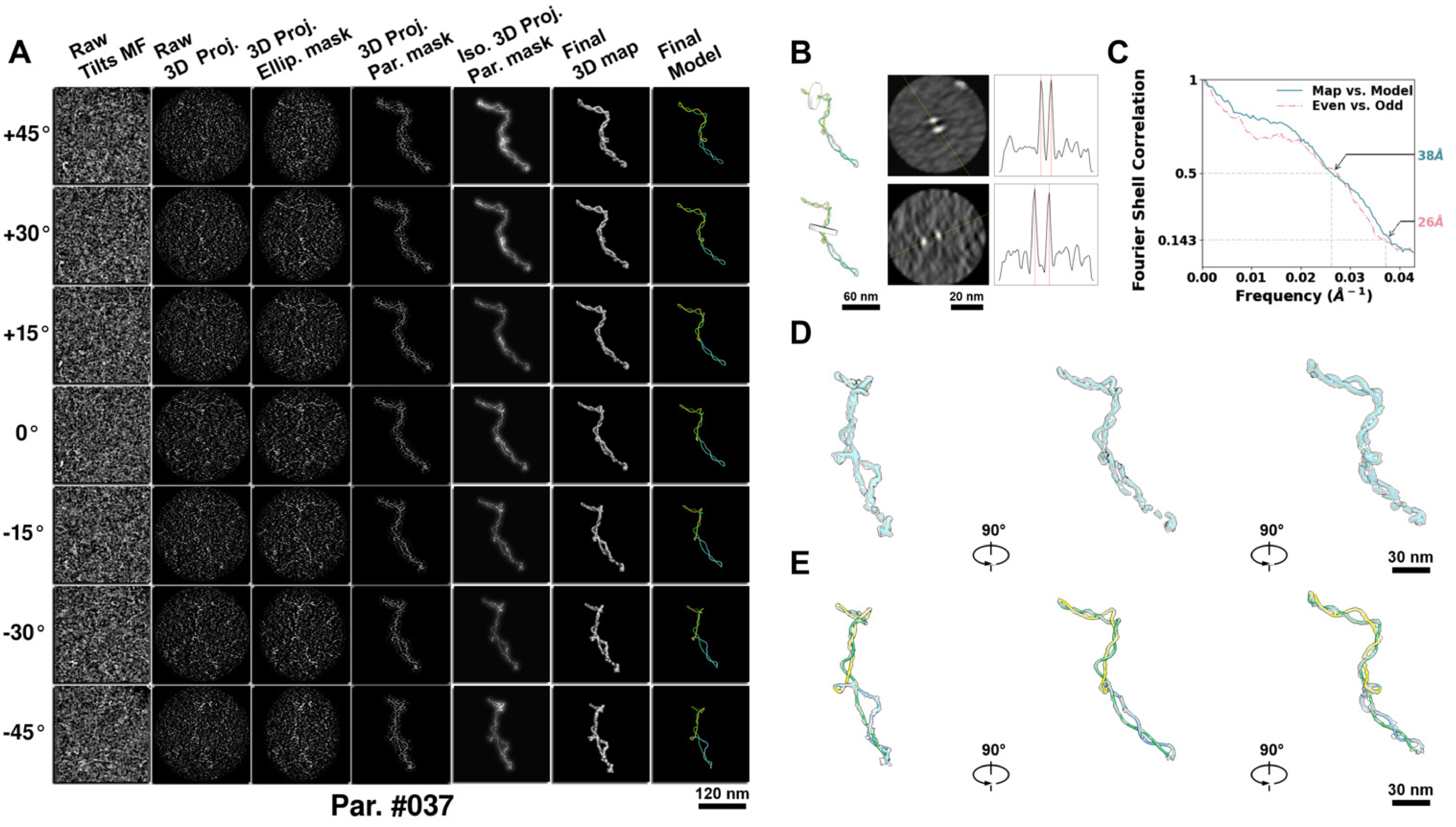

Supplementary Figure 37. Cryo-electron tomography (cryo-ET) 3D reconstruction of an individual plasmid particle in 40 mM K^+, 5 mM Mg^{2+}.
(A) 3D reconstruction of the plasmid particle (index no. 37). The first column shows seven representative tilt images from –45° to +45° in step of 15°. The second, third, and fourth columns show 3D projections of the particle with spherical, ellipsoidal (thinner along the z-dimension), and particle-shaped masks, respectively. The fifth column displays the 3D projections of the enhanced and IsoNet missing-wedge-corrected particle. The sixth and seventh columns present the final 3D map and the flexibly fitted model, respectively. (B) Two random cross-sectional views (12 nm thickness) of the plasmid density map along its plectoneme axis are shown in the left-middle panel. The intensity profile along the line crossing the two high-density DNA spots is displayed in the right panel. (C) Resolution assessment of the final 3D map using Fourier shell correlation (FSC). Two criteria are shown: FSC between two half-maps reconstructed from even and odd frames (evaluated at 0.143) and FSC between the final 3D map and the fitted model (evaluated at 0.5). (D) Zoomed-in views of the final 3D density map from panel A, displayed at two contour levels. (E) Superimposition of the high-contour level map from panel D onto its flexibly fitted model.

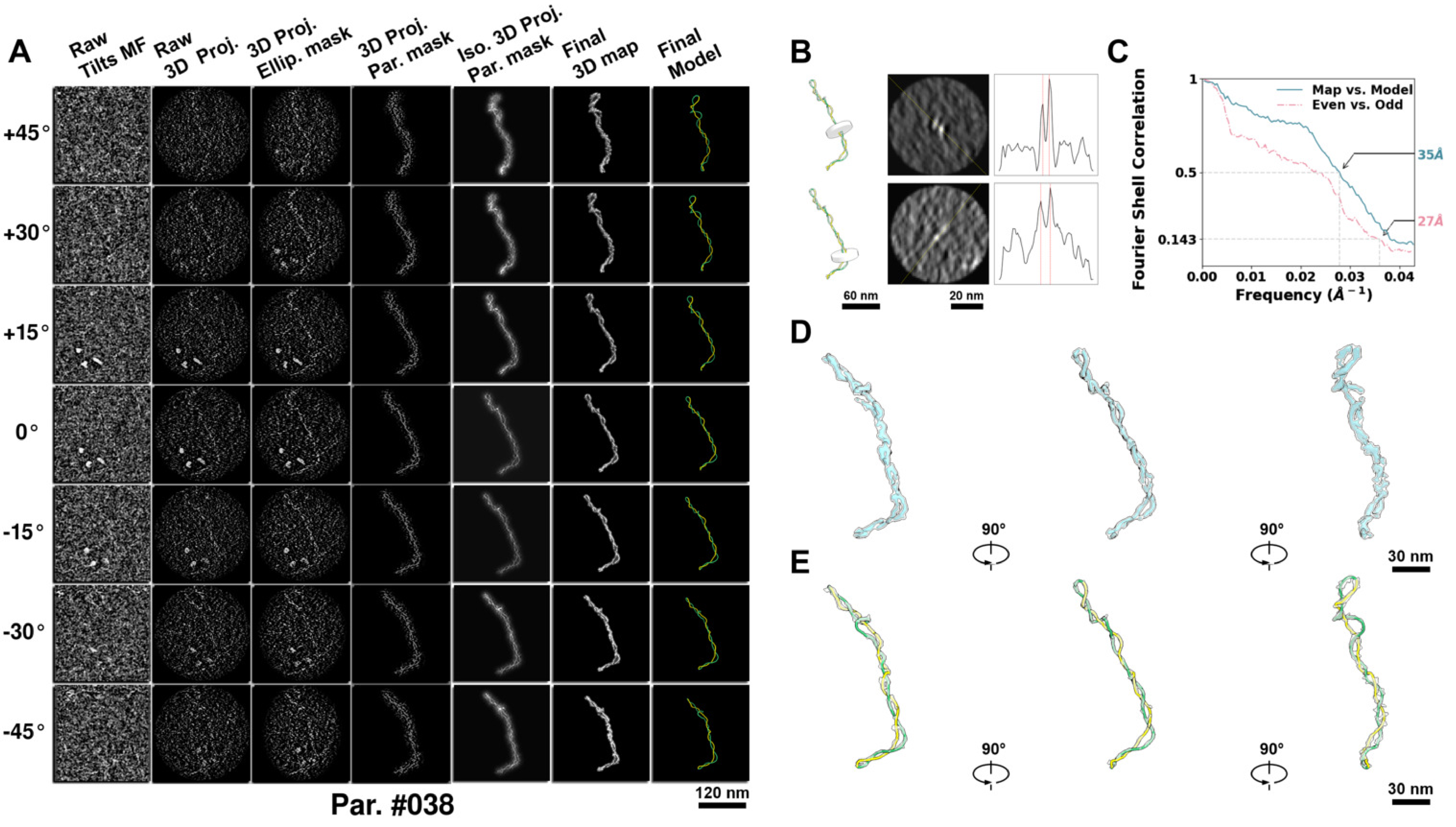

Supplementary Figure 38. Cryo-electron tomography (cryo-ET) 3D reconstruction of an individual plasmid particle in 40 mM K^+, 5 mM Mg^{2+}.
(A) 3D reconstruction of the plasmid particle (index no. 38). The first column shows seven representative tilt images from –45° to +45° in step of 15°. The second, third, and fourth columns show 3D projections of the particle with spherical, ellipsoidal (thinner along the z-dimension), and particle-shaped masks, respectively. The fifth column displays the 3D projections of the enhanced and IsoNet missing-wedge-corrected particle. The sixth and seventh columns present the final 3D map and the flexibly fitted model, respectively. (B) Two random cross-sectional views (12 nm thickness) of the plasmid density map along its plectoneme axis are shown in the left-middle panel. The intensity profile along the line crossing the two high-density DNA spots is displayed in the right panel. (C) Resolution assessment of the final 3D map using Fourier shell correlation (FSC). Two criteria are shown: FSC between two half-maps reconstructed from even and odd frames (evaluated at 0.143) and FSC between the final 3D map and the fitted model (evaluated at 0.5). (D) Zoomed-in views of the final 3D density map from panel A, displayed at two contour levels. (E) Superimposition of the high-contour level map from panel D onto its flexibly fitted model.

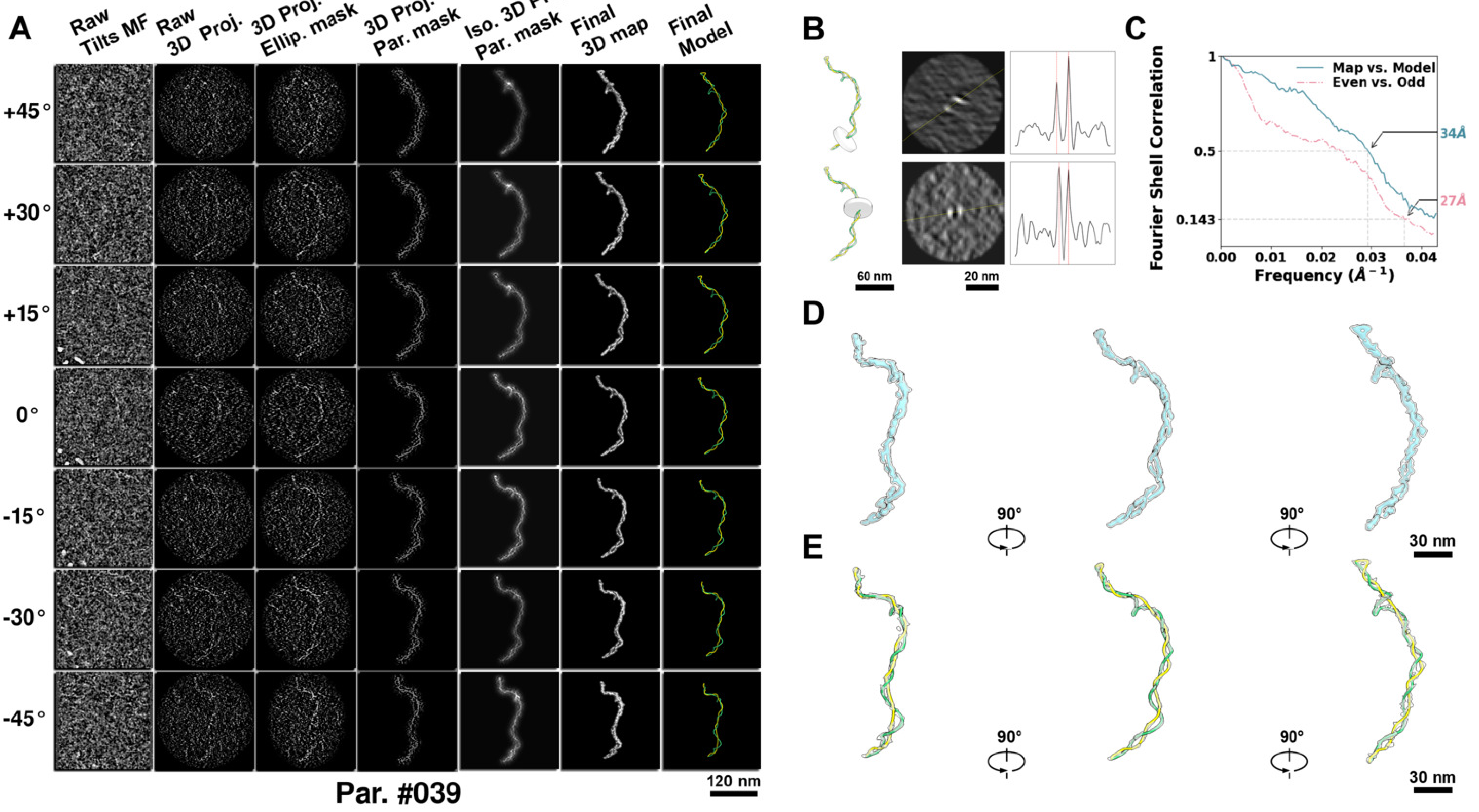

Supplementary Figure 39. Cryo-electron tomography (cryo-ET) 3D reconstruction of an individual plasmid particle in 40 mM K^+, 5 mM Mg^{2+}.
(A) 3D reconstruction of the plasmid particle (index no. 39). The first column shows seven representative tilt images from –45° to +45° in step of 15°. The second, third, and fourth columns show 3D projections of the particle with spherical, ellipsoidal (thinner along the z-dimension), and particle-shaped masks, respectively. The fifth column displays the 3D projections of the enhanced and IsoNet missing-wedge-corrected particle. The sixth and seventh columns present the final 3D map and the flexibly fitted model, respectively. (B) Two random cross-sectional views (12 nm thickness) of the plasmid density map along its plectoneme axis are shown in the left-middle panel. The intensity profile along the line crossing the two high-density DNA spots is displayed in the right panel. (C) Resolution assessment of the final 3D map using Fourier shell correlation (FSC). Two criteria are shown: FSC between two half-maps reconstructed from even and odd frames (evaluated at 0.143) and FSC between the final 3D map and the fitted model (evaluated at 0.5). (D) Zoomed-in views of the final 3D density map from panel A, displayed at two contour levels. (E) Superimposition of the high-contour level map from panel D onto its flexibly fitted model.

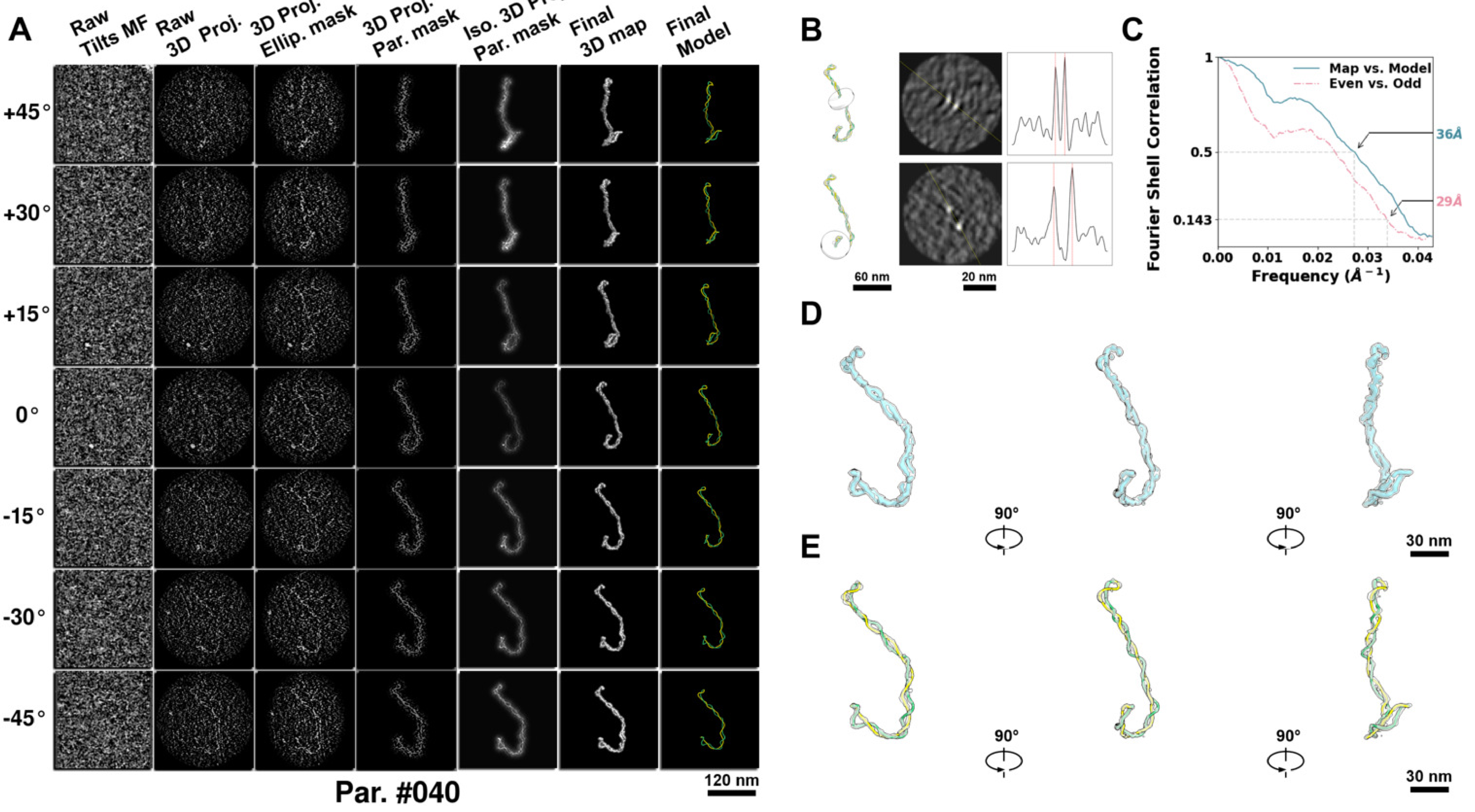

Supplementary Figure 40. Cryo-electron tomography (cryo-ET) 3D reconstruction of an individual plasmid particle in 40 mM K^+, 5 mM Mg^{2+}.
(A) 3D reconstruction of the plasmid particle (index no. 40). The first column shows seven representative tilt images from –45° to +45° in step of 15°. The second, third, and fourth columns show 3D projections of the particle with spherical, ellipsoidal (thinner along the z-dimension), and particle-shaped masks, respectively. The fifth column displays the 3D projections of the enhanced and IsoNet missing-wedge-corrected particle. The sixth and seventh columns present the final 3D map and the flexibly fitted model, respectively. (B) Two random cross-sectional views (12 nm thickness) of the plasmid density map along its plectoneme axis are shown in the left-middle panel. The intensity profile along the line crossing the two high-density DNA spots is displayed in the right panel. (C) Resolution assessment of the final 3D map using Fourier shell correlation (FSC). Two criteria are shown: FSC between two half-maps reconstructed from even and odd frames (evaluated at 0.143) and FSC between the final 3D map and the fitted model (evaluated at 0.5). (D) Zoomed-in views of the final 3D density map from panel A, displayed at two contour levels. (E) Superimposition of the high-contour level map from panel D onto its flexibly fitted model.

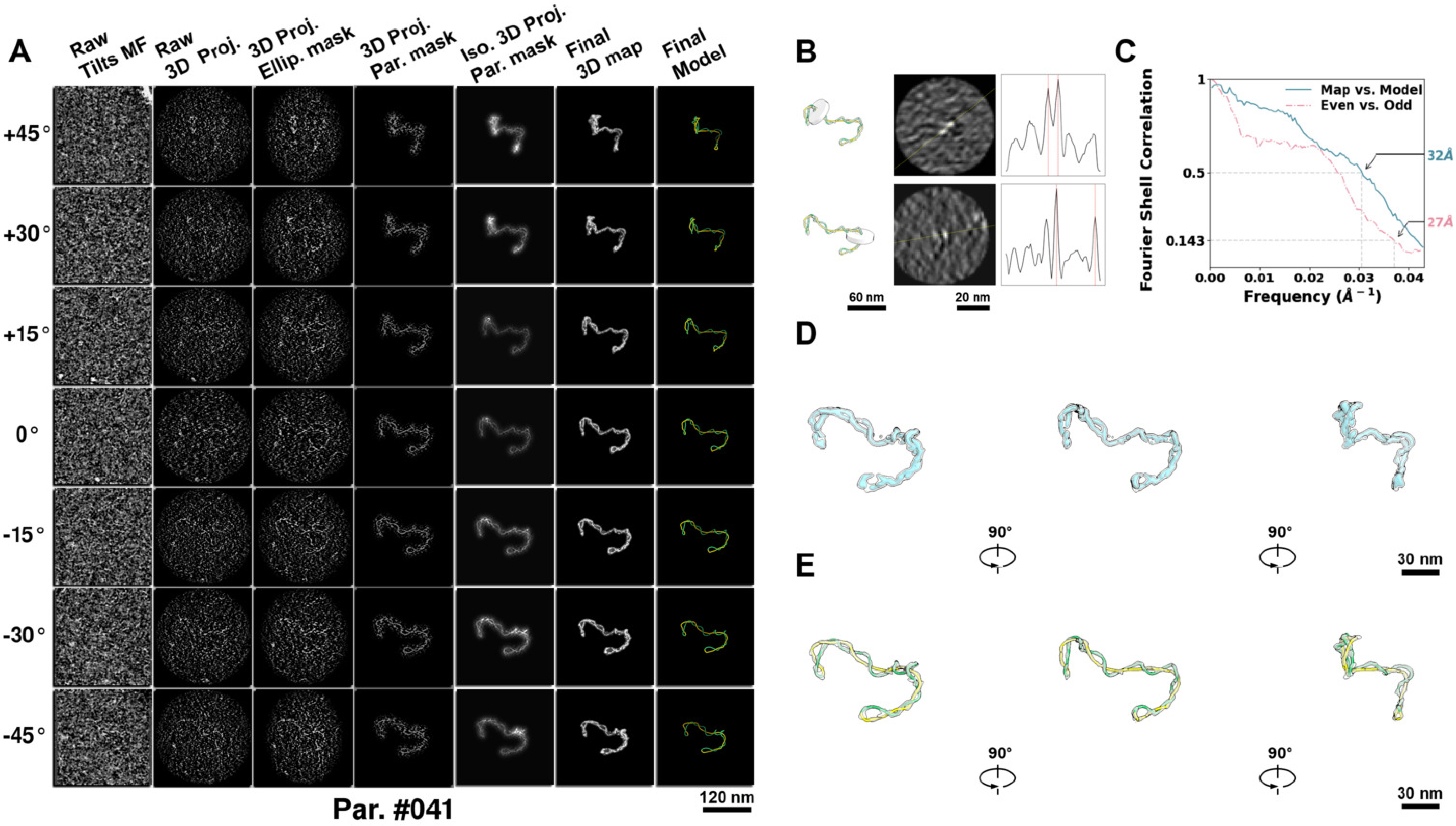

Supplementary Figure 41. Cryo-electron tomography (cryo-ET) 3D reconstruction of an individual plasmid particle in 40 mM K^+, 5 mM Mg^{2+}.
(A) 3D reconstruction of the plasmid particle (index no. 41). The first column shows seven representative tilt images from –45° to +45° in step of 15°. The second, third, and fourth columns show 3D projections of the particle with spherical, ellipsoidal (thinner along the z-dimension), and particle-shaped masks, respectively. The fifth column displays the 3D projections of the enhanced and IsoNet missing-wedge-corrected particle. The sixth and seventh columns present the final 3D map and the flexibly fitted model, respectively. (B) Two random cross-sectional views (12 nm thickness) of the plasmid density map along its plectoneme axis are shown in the left-middle panel. The intensity profile along the line crossing the two high-density DNA spots is displayed in the right panel. (C) Resolution assessment of the final 3D map using Fourier shell correlation (FSC). Two criteria are shown: FSC between two half-maps reconstructed from even and odd frames (evaluated at 0.143) and FSC between the final 3D map and the fitted model (evaluated at 0.5). (D) Zoomed-in views of the final 3D density map from panel A, displayed at two contour levels. (E) Superimposition of the high-contour level map from panel D onto its flexibly fitted model.

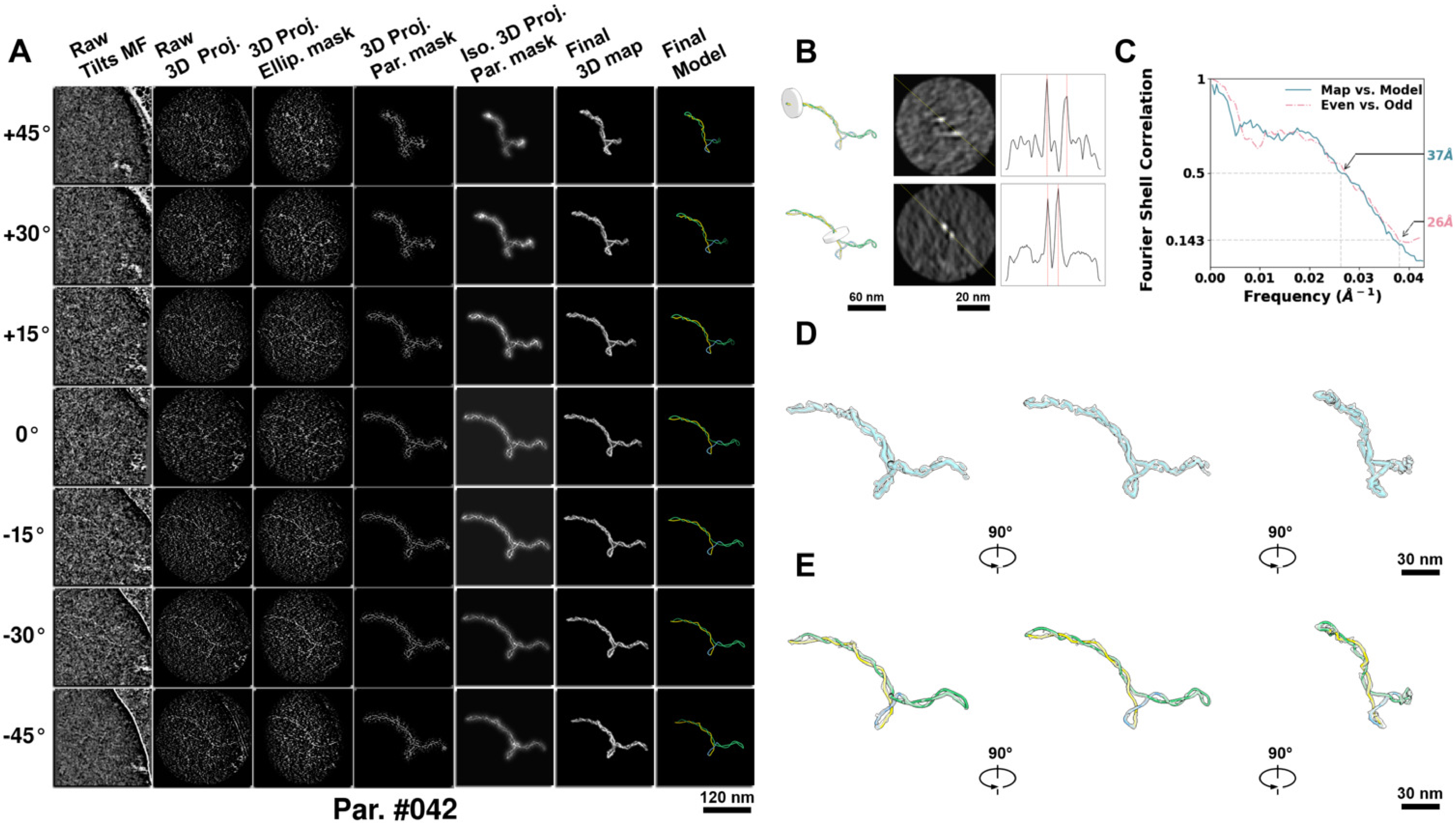

Supplementary Figure 42. Cryo-electron tomography (cryo-ET) 3D reconstruction of an individual plasmid particle in 40 mM K^+, 5 mM Mg^{2+}.
(A) 3D reconstruction of the plasmid particle (index no. 42). The first column shows seven representative tilt images from –45° to +45° in step of 15°. The second, third, and fourth columns show 3D projections of the particle with spherical, ellipsoidal (thinner along the z-dimension), and particle-shaped masks, respectively. The fifth column displays the 3D projections of the enhanced and IsoNet missing-wedge-corrected particle. The sixth and seventh columns present the final 3D map and the flexibly fitted model, respectively. (B) Two random cross-sectional views (12 nm thickness) of the plasmid density map along its plectoneme axis are shown in the left-middle panel. The intensity profile along the line crossing the two high-density DNA spots is displayed in the right panel. (C) Resolution assessment of the final 3D map using Fourier shell correlation (FSC). Two criteria are shown: FSC between two half-maps reconstructed from even and odd frames (evaluated at 0.143) and FSC between the final 3D map and the fitted model (evaluated at 0.5). (D) Zoomed-in views of the final 3D density map from panel A, displayed at two contour levels. (E) Superimposition of the high-contour level map from panel D onto its flexibly fitted model.

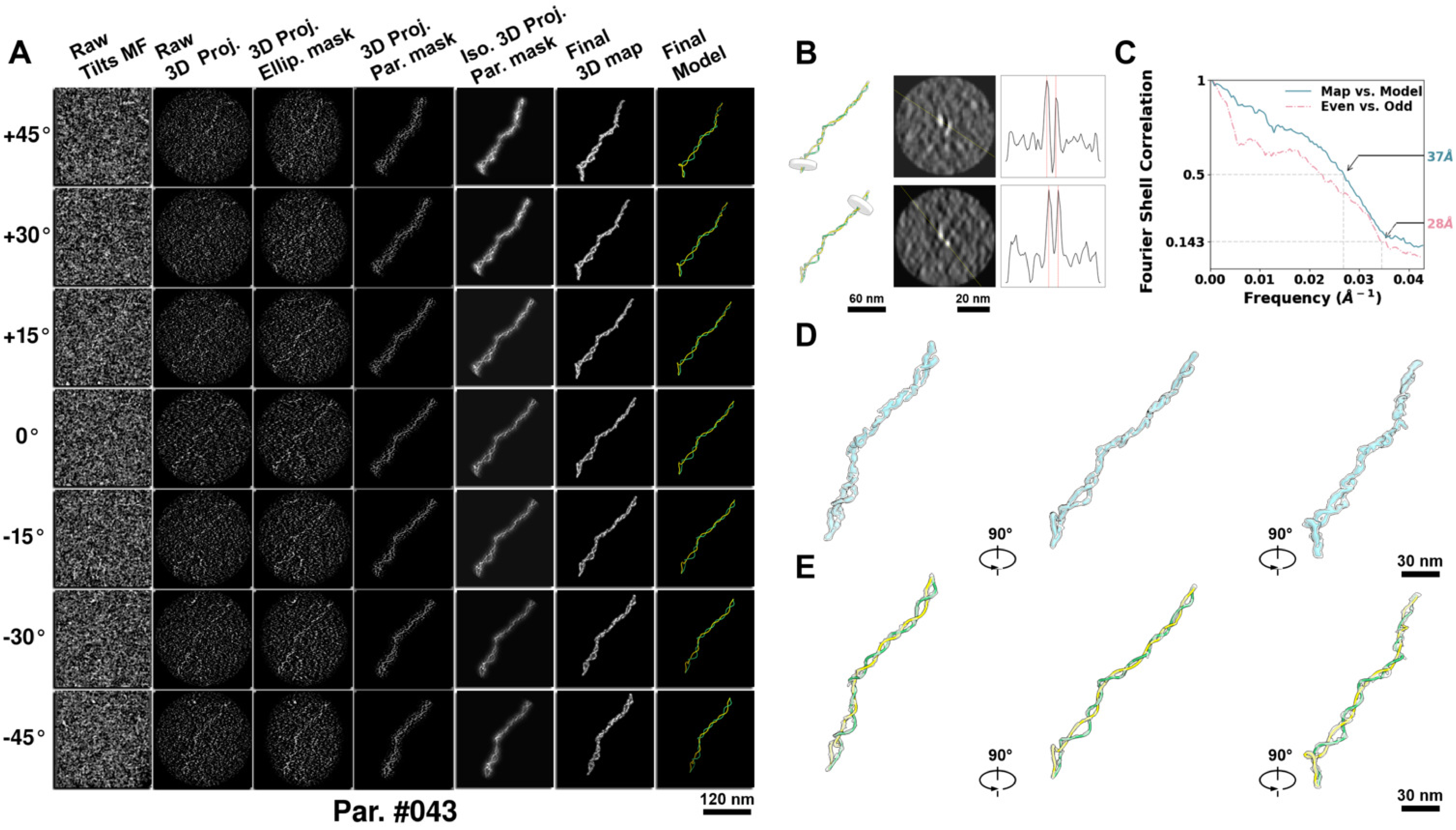

Supplementary Figure 43. Cryo-electron tomography (cryo-ET) 3D reconstruction of an individual plasmid particle in 40 mM K^+, 5 mM Mg^{2+}.
(A) 3D reconstruction of the plasmid particle (index no. 43). The first column shows seven representative tilt images from –45° to +45° in step of 15°. The second, third, and fourth columns show 3D projections of the particle with spherical, ellipsoidal (thinner along the z-dimension), and particle-shaped masks, respectively. The fifth column displays the 3D projections of the enhanced and IsoNet missing-wedge-corrected particle. The sixth and seventh columns present the final 3D map and the flexibly fitted model, respectively. (B) Two random cross-sectional views (12 nm thickness) of the plasmid density map along its plectoneme axis are shown in the left-middle panel. The intensity profile along the line crossing the two high-density DNA spots is displayed in the right panel. (C) Resolution assessment of the final 3D map using Fourier shell correlation (FSC). Two criteria are shown: FSC between two half-maps reconstructed from even and odd frames (evaluated at 0.143) and FSC between the final 3D map and the fitted model (evaluated at 0.5). (D) Zoomed-in views of the final 3D density map from panel A, displayed at two contour levels. (E) Superimposition of the high-contour level map from panel D onto its flexibly fitted model.

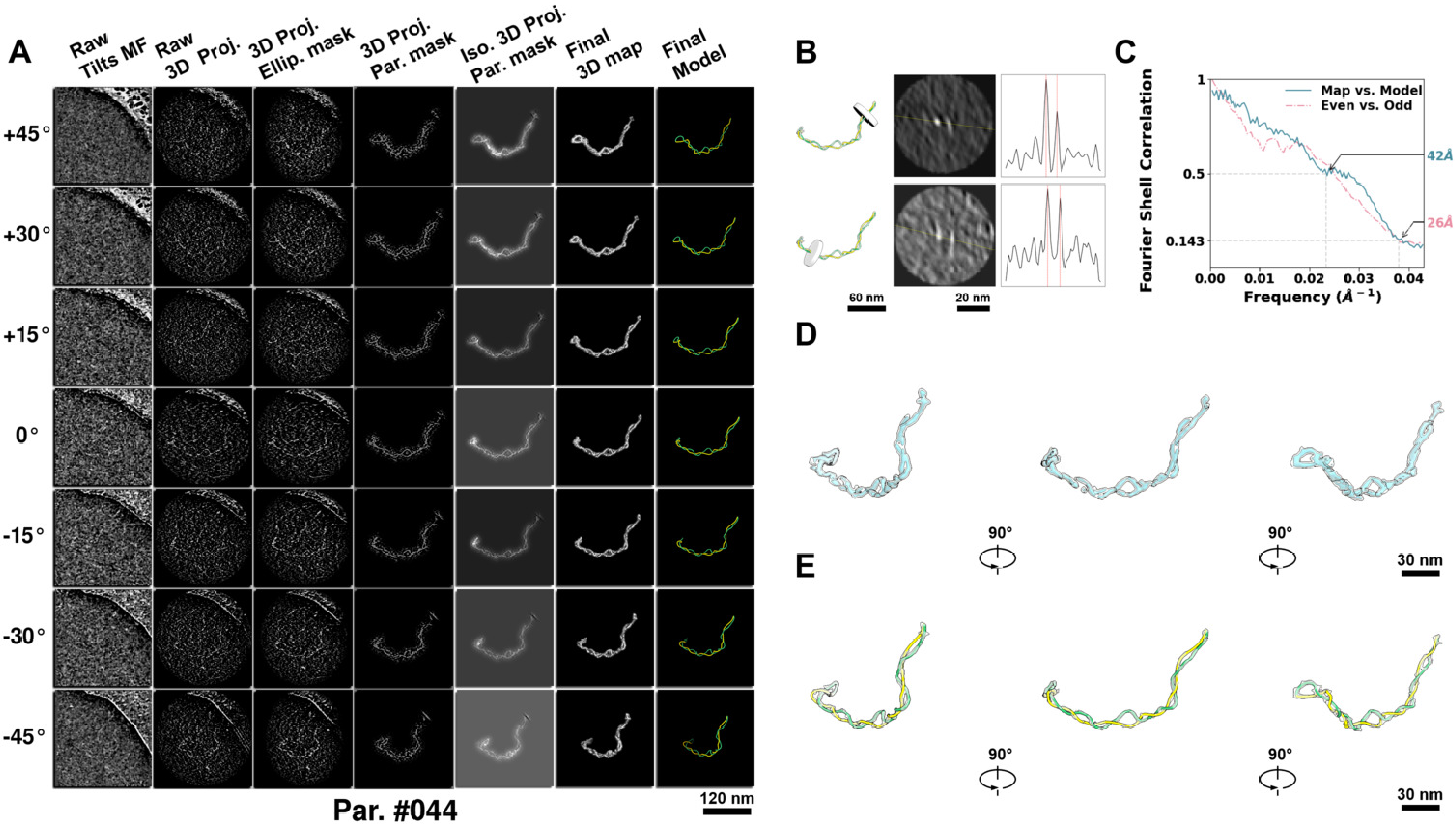

Supplementary Figure 44. Cryo-electron tomography (cryo-ET) 3D reconstruction of an individual plasmid particle in 40 mM K^+, 5 mM Mg^{2+}.
(A) 3D reconstruction of the plasmid particle (index no. 44). The first column shows seven representative tilt images from –45° to +45° in step of 15°. The second, third, and fourth columns show 3D projections of the particle with spherical, ellipsoidal (thinner along the z-dimension), and particle-shaped masks, respectively. The fifth column displays the 3D projections of the enhanced and IsoNet missing-wedge-corrected particle. The sixth and seventh columns present the final 3D map and the flexibly fitted model, respectively. (B) Two random cross-sectional views (12 nm thickness) of the plasmid density map along its plectoneme axis are shown in the left-middle panel. The intensity profile along the line crossing the two high-density DNA spots is displayed in the right panel. (C) Resolution assessment of the final 3D map using Fourier shell correlation (FSC). Two criteria are shown: FSC between two half-maps reconstructed from even and odd frames (evaluated at 0.143) and FSC between the final 3D map and the fitted model (evaluated at 0.5). (D) Zoomed-in views of the final 3D density map from panel A, displayed at two contour levels. (E) Superimposition of the high-contour level map from panel D onto its flexibly fitted model.

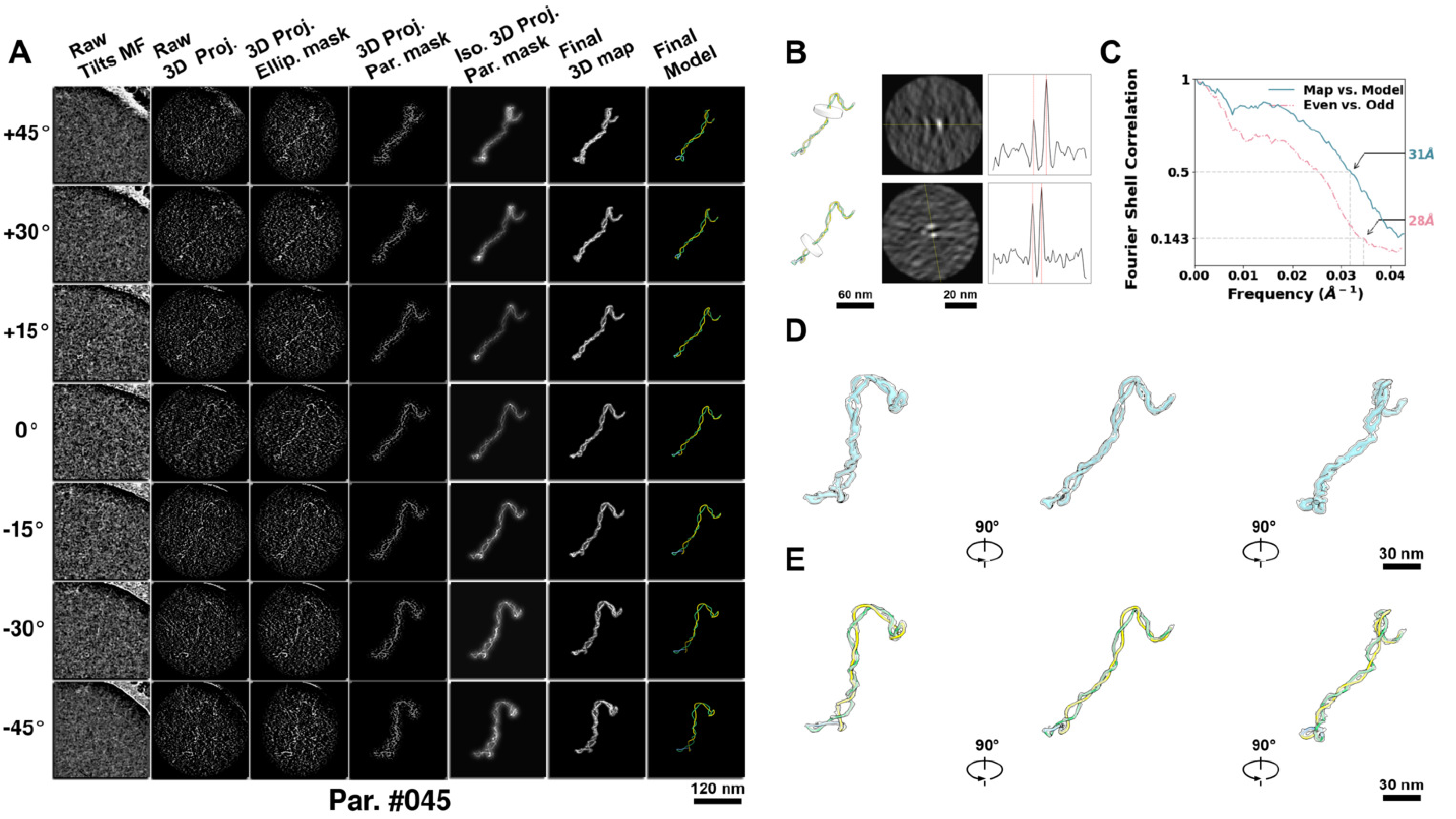

Supplementary Figure 45. Cryo-electron tomography (cryo-ET) 3D reconstruction of an individual plasmid particle in 40 mM K^{+}, 5 mM Mg^{2+}.
(A) 3D reconstruction of the plasmid particle (index no. 45). The first column shows seven representative tilt images from –45° to +45° in step of 15°. The second, third, and fourth columns show 3D projections of the particle with spherical, ellipsoidal (thinner along the z-dimension), and particle-shaped masks, respectively. The fifth column displays the 3D projections of the enhanced and IsoNet missing-wedge-corrected particle. The sixth and seventh columns present the final 3D map and the flexibly fitted model, respectively. (B) Two random cross-sectional views (12 nm thickness) of the plasmid density map along its plectoneme axis are shown in the left-middle panel. The intensity profile along the line crossing the two high-density DNA spots is displayed in the right panel. (C) Resolution assessment of the final 3D map using Fourier shell correlation (FSC). Two criteria are shown: FSC between two half-maps reconstructed from even and odd frames (evaluated at 0.143) and FSC between the final 3D map and the fitted model (evaluated at 0.5). (D) Zoomed-in views of the final 3D density map from panel A, displayed at two contour levels. (E) Superimposition of the high-contour level map from panel D onto its flexibly fitted model.

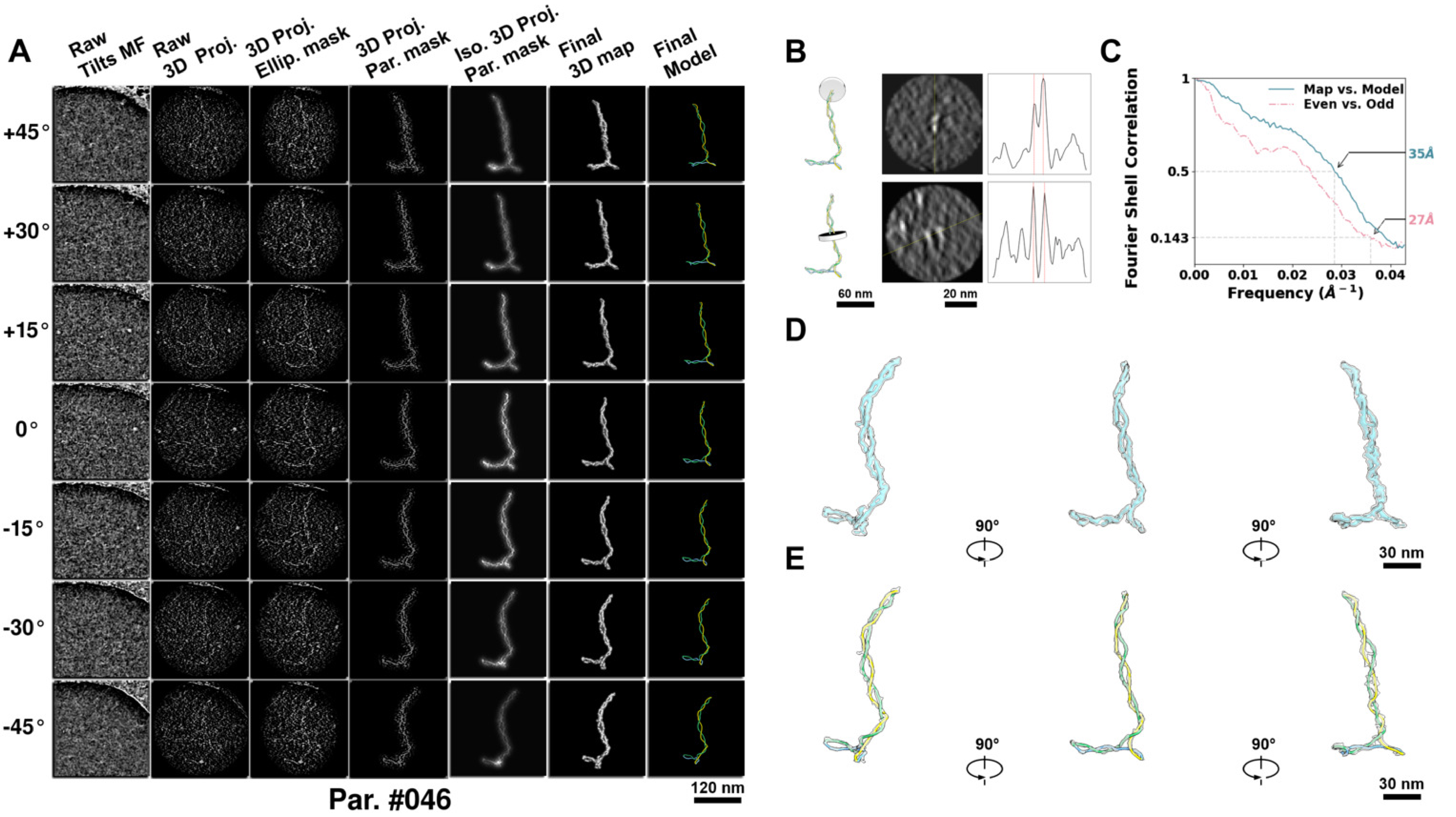

Supplementary Figure 46. Cryo-electron tomography (cryo-ET) 3D reconstruction of an individual plasmid particle in 40 mM K^{+}, 5 mM Mg^{2+}.
(A) 3D reconstruction of the plasmid particle (index no. 46). The first column shows seven representative tilt images from –45° to +45° in step of 15°. The second, third, and fourth columns show 3D projections of the particle with spherical, ellipsoidal (thinner along the z-dimension), and particle-shaped masks, respectively. The fifth column displays the 3D projections of the enhanced and IsoNet missing-wedge-corrected particle. The sixth and seventh columns present the final 3D map and the flexibly fitted model, respectively. (B) Two random cross-sectional views (12 nm thickness) of the plasmid density map along its plectoneme axis are shown in the left-middle panel. The intensity profile along the line crossing the two high-density DNA spots is displayed in the right panel. (C) Resolution assessment of the final 3D map using Fourier shell correlation (FSC). Two criteria are shown: FSC between two half-maps reconstructed from even and odd frames (evaluated at 0.143) and FSC between the final 3D map and the fitted model (evaluated at 0.5). (D) Zoomed-in views of the final 3D density map from panel A, displayed at two contour levels. (E) Superimposition of the high-contour level map from panel D onto its flexibly fitted model.

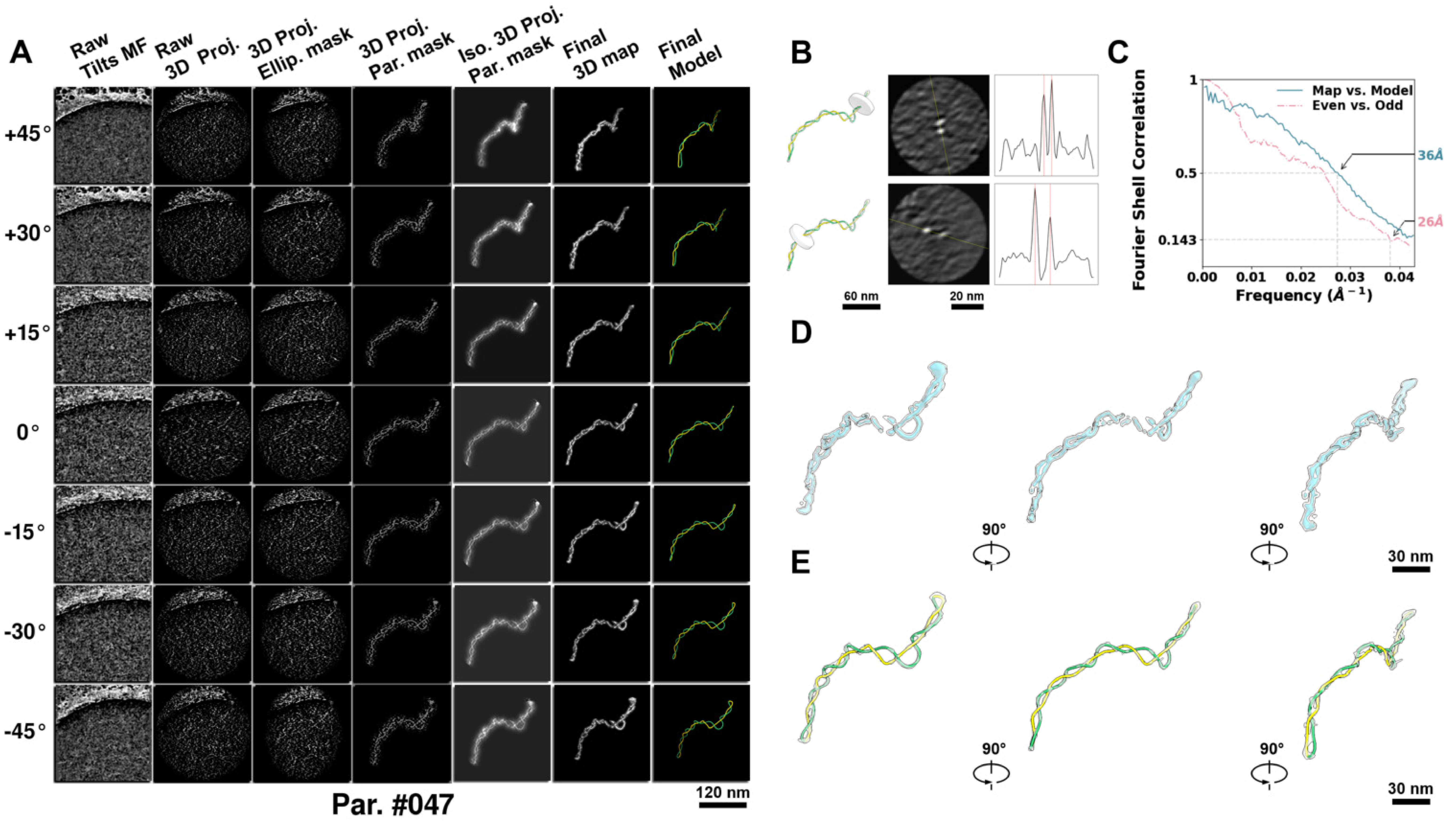

Supplementary Figure 47. Cryo-electron tomography (cryo-ET) 3D reconstruction of an individual plasmid particle in 40 mM K^+, 5 mM Mg^{2+}.
(A) 3D reconstruction of the plasmid particle (index no. 47). The first column shows seven representative tilt images from –45° to +45° in step of 15°. The second, third, and fourth columns show 3D projections of the particle with spherical, ellipsoidal (thinner along the z-dimension), and particle-shaped masks, respectively. The fifth column displays the 3D projections of the enhanced and IsoNet missing-wedge-corrected particle. The sixth and seventh columns present the final 3D map and the flexibly fitted model, respectively. (B) Two random cross-sectional views (12 nm thickness) of the plasmid density map along its plectoneme axis are shown in the left-middle panel. The intensity profile along the line crossing the two high-density DNA spots is displayed in the right panel. (C) Resolution assessment of the final 3D map using Fourier shell correlation (FSC). Two criteria are shown: FSC between two half-maps reconstructed from even and odd frames (evaluated at 0.143) and FSC between the final 3D map and the fitted model (evaluated at 0.5). (D) Zoomed-in views of the final 3D density map from panel A, displayed at two contour levels. (E) Superimposition of the high-contour level map from panel D onto its flexibly fitted model.

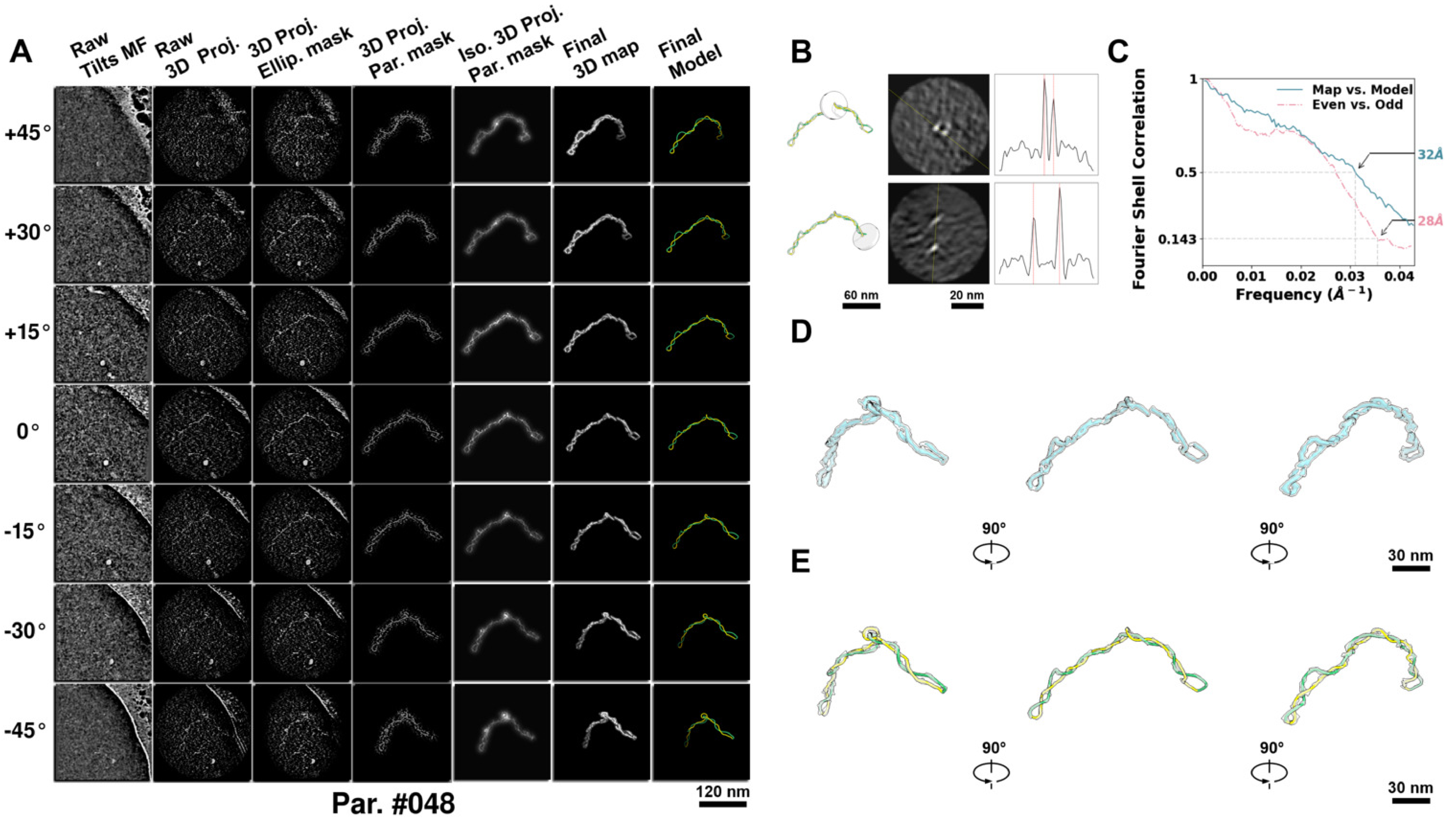

Supplementary Figure 48. Cryo-electron tomography (cryo-ET) 3D reconstruction of an individual plasmid particle in 40 mM K^+, 5 mM Mg^{2+}.
(A) 3D reconstruction of the plasmid particle (index no. 48). The first column shows seven representative tilt images from –45° to +45° in step of 15°. The second, third, and fourth columns show 3D projections of the particle with spherical, ellipsoidal (thinner along the z-dimension), and particle-shaped masks, respectively. The fifth column displays the 3D projections of the enhanced and IsoNet missing-wedge-corrected particle. The sixth and seventh columns present the final 3D map and the flexibly fitted model, respectively. (B) Two random cross-sectional views (12 nm thickness) of the plasmid density map along its plectoneme axis are shown in the left-middle panel. The intensity profile along the line crossing the two high-density DNA spots is displayed in the right panel. (C) Resolution assessment of the final 3D map using Fourier shell correlation (FSC). Two criteria are shown: FSC between two half-maps reconstructed from even and odd frames (evaluated at 0.143) and FSC between the final 3D map and the fitted model (evaluated at 0.5). (D) Zoomed-in views of the final 3D density map from panel A, displayed at two contour levels. (E) Superimposition of the high-contour level map from panel D onto its flexibly fitted model.

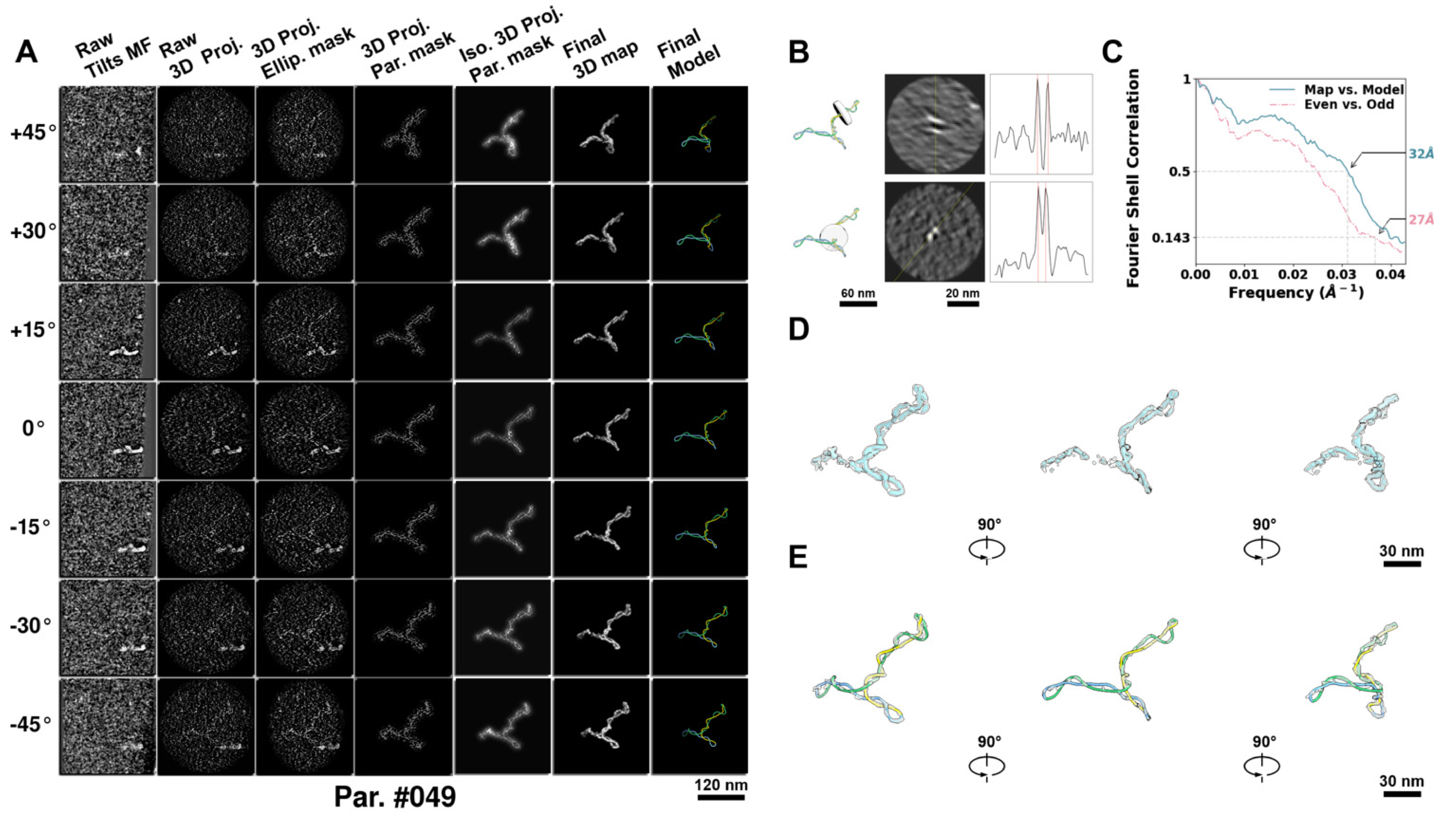

Supplementary Figure 49. Cryo-electron tomography (cryo-ET) 3D reconstruction of an individual plasmid particle in 40 mM K^+, 5 mM Mg^{2+}.
(A) 3D reconstruction of the plasmid particle (index no. 49). The first column shows seven representative tilt images from –45° to +45° in step of 15°. The second, third, and fourth columns show 3D projections of the particle with spherical, ellipsoidal (thinner along the z-dimension), and particle-shaped masks, respectively. The fifth column displays the 3D projections of the enhanced and IsoNet missing-wedge-corrected particle. The sixth and seventh columns present the final 3D map and the flexibly fitted model, respectively. (B) Two random cross-sectional views (12 nm thickness) of the plasmid density map along its plectoneme axis are shown in the left-middle panel. The intensity profile along the line crossing the two high-density DNA spots is displayed in the right panel. (C) Resolution assessment of the final 3D map using Fourier shell correlation (FSC). Two criteria are shown: FSC between two half-maps reconstructed from even and odd frames (evaluated at 0.143) and FSC between the final 3D map and the fitted model (evaluated at 0.5). (D) Zoomed-in views of the final 3D density map from panel A, displayed at two contour levels. (E) Superimposition of the high-contour level map from panel D onto its flexibly fitted model.

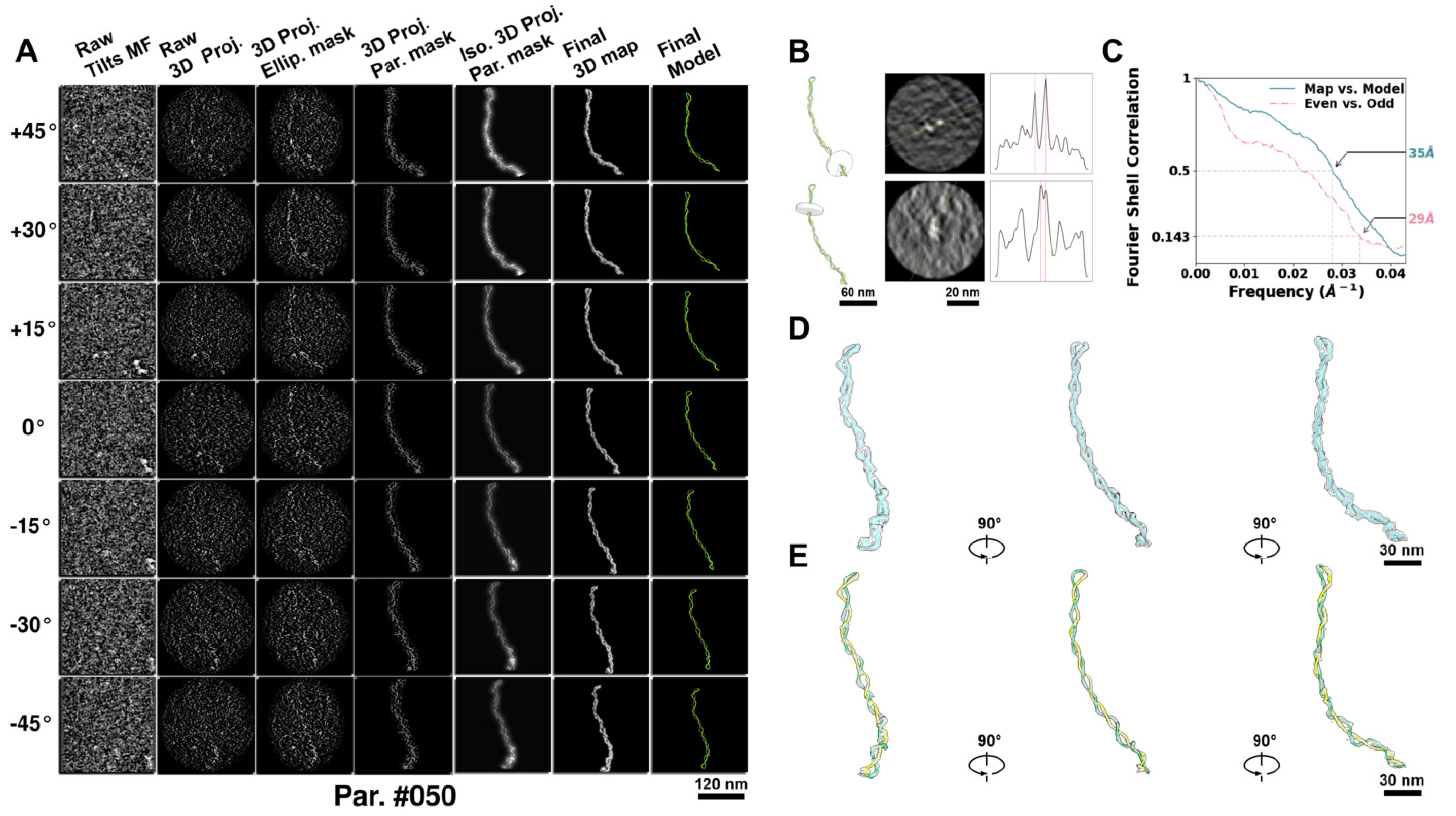

Supplementary Figure 50. Cryo-electron tomography (cryo-ET) 3D reconstruction of an individual plasmid particle in 40 mM K^+, 5 mM Mg^{2+}.
(A) 3D reconstruction of the plasmid particle (index no. 50). The first column shows seven representative tilt images from –45° to +45° in step of 15°. The second, third, and fourth columns show 3D projections of the particle with spherical, ellipsoidal (thinner along the z-dimension), and particle-shaped masks, respectively. The fifth column displays the 3D projections of the enhanced and IsoNet missing-wedge-corrected particle. The sixth and seventh columns present the final 3D map and the flexibly fitted model, respectively. (B) Two random cross-sectional views (12 nm thickness) of the plasmid density map along its plectoneme axis are shown in the left-middle panel. The intensity profile along the line crossing the two high-density DNA spots is displayed in the right panel. (C) Resolution assessment of the final 3D map using Fourier shell correlation (FSC). Two criteria are shown: FSC between two half-maps reconstructed from even and odd frames (evaluated at 0.143) and FSC between the final 3D map and the fitted model (evaluated at 0.5). (D) Zoomed-in views of the final 3D density map from panel A, displayed at two contour levels. (E) Superimposition of the high-contour level map from panel D onto its flexibly fitted model.

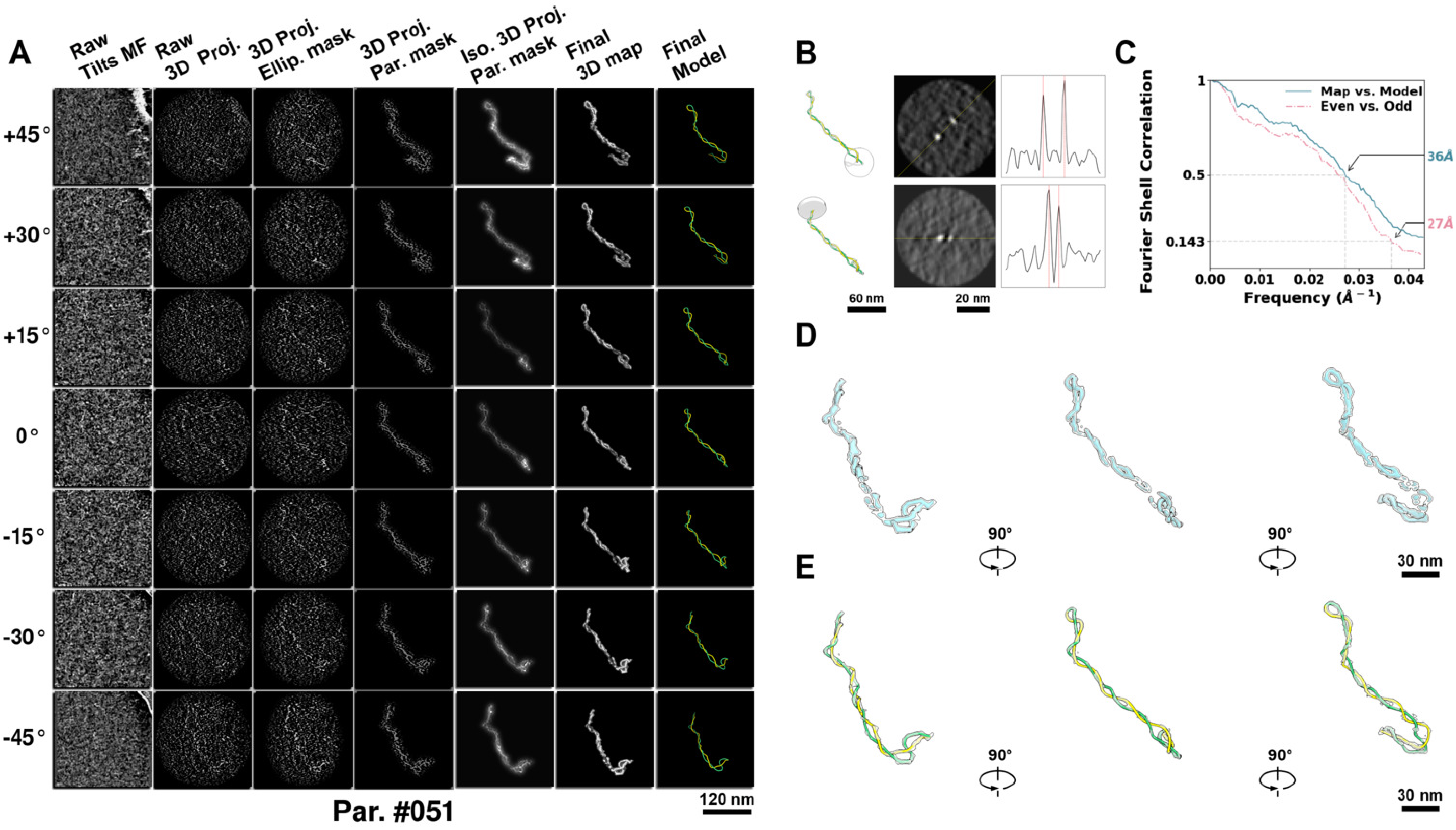

Supplementary Figure 51. Cryo-electron tomography (cryo-ET) 3D reconstruction of an individual plasmid particle in 40 mM K⁺, 5 mM Mg²⁺.
(A) 3D reconstruction of the plasmid particle (index no. 51). The first column shows seven representative tilt images from –45° to +45° in step of 15°. The second, third, and fourth columns show 3D projections of the particle with spherical, ellipsoidal (thinner along the z-dimension), and particle-shaped masks, respectively. The fifth column displays the 3D projections of the enhanced and IsoNet missing-wedge-corrected particle. The sixth and seventh columns present the final 3D map and the flexibly fitted model, respectively. (B) Two random cross-sectional views (12 nm thickness) of the plasmid density map along its plectoneme axis are shown in the left-middle panel. The intensity profile along the line crossing the two high-density DNA spots is displayed in the right panel. (C) Resolution assessment of the final 3D map using Fourier shell correlation (FSC). Two criteria are shown: FSC between two half-maps reconstructed from even and odd frames (evaluated at 0.143) and FSC between the final 3D map and the fitted model (evaluated at 0.5). (D) Zoomed-in views of the final 3D density map from panel A, displayed at two contour levels. (E) Superimposition of the high-contour level map from panel D onto its flexibly fitted model.

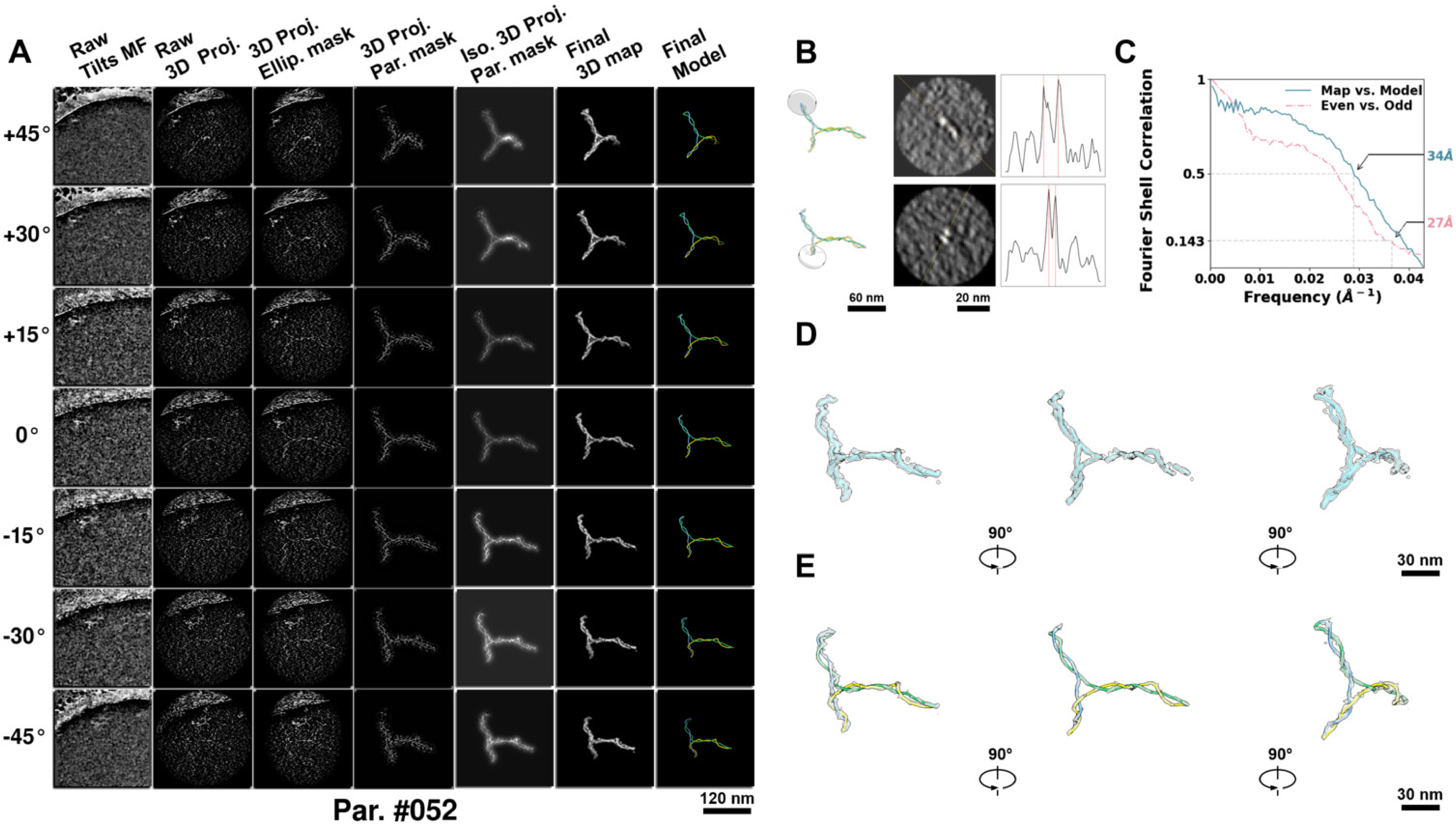

Supplementary Figure 52. Cryo-electron tomography (cryo-ET) 3D reconstruction of an individual plasmid particle in 40 mM K⁺, 5 mM Mg²⁺.
(A) 3D reconstruction of the plasmid particle (index no. 52). The first column shows seven representative tilt images from –45° to +45° in step of 15°. The second, third, and fourth columns show 3D projections of the particle with spherical, ellipsoidal (thinner along the z-dimension), and particle-shaped masks, respectively. The fifth column displays the 3D projections of the enhanced and IsoNet missing-wedge-corrected particle. The sixth and seventh columns present the final 3D map and the flexibly fitted model, respectively. (B) Two random cross-sectional views (12 nm thickness) of the plasmid density map along its plectoneme axis are shown in the left-middle panel. The intensity profile along the line crossing the two high-density DNA spots is displayed in the right panel. (C) Resolution assessment of the final 3D map using Fourier shell correlation (FSC). Two criteria are shown: FSC between two half-maps reconstructed from even and odd frames (evaluated at 0.143) and FSC between the final 3D map and the fitted model (evaluated at 0.5). (D) Zoomed-in views of the final 3D density map from panel A, displayed at two contour levels. (E) Superimposition of the high-contour level map from panel D onto its flexibly fitted model.

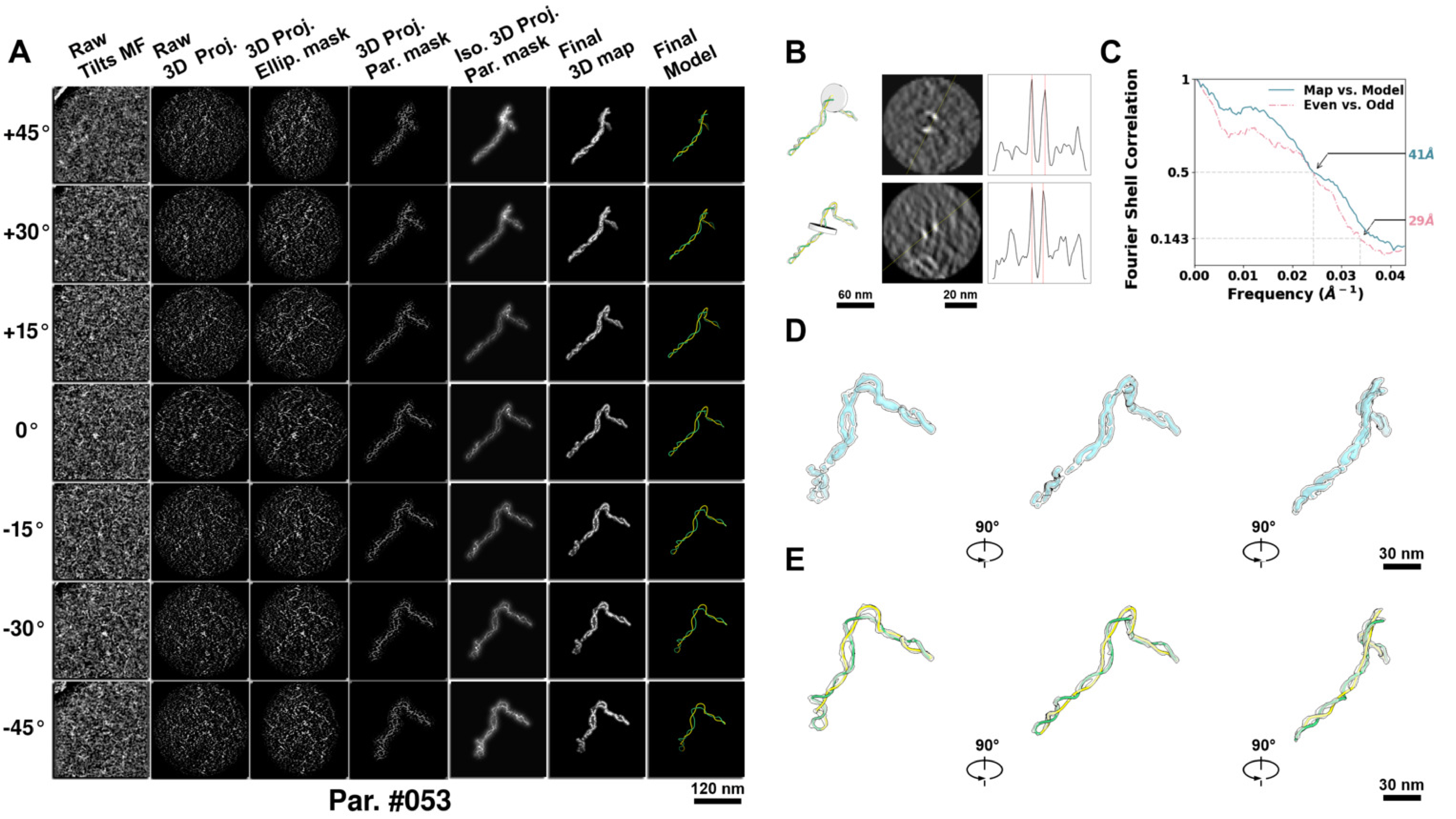

Supplementary Figure 53. Cryo-electron tomography (cryo-ET) 3D reconstruction of an individual plasmid particle in 40 mM K$^+$, 5 mM Mg^{2+}.
(A) 3D reconstruction of the plasmid particle (index no. 53). The first column shows seven representative tilt images from –45° to +45° in step of 15°. The second, third, and fourth columns show 3D projections of the particle with spherical, ellipsoidal (thinner along the z-dimension), and particle-shaped masks, respectively. The fifth column displays the 3D projections of the enhanced and IsoNet missing-wedge-corrected particle. The sixth and seventh columns present the final 3D map and the flexibly fitted model, respectively. (B) Two random cross-sectional views (12 nm thickness) of the plasmid density map along its plectoneme axis are shown in the left-middle panel. The intensity profile along the line crossing the two high-density DNA spots is displayed in the right panel. (C) Resolution assessment of the final 3D map using Fourier shell correlation (FSC). Two criteria are shown: FSC between two half-maps reconstructed from even and odd frames (evaluated at 0.143) and FSC between the final 3D map and the fitted model (evaluated at 0.5). (D) Zoomed-in views of the final 3D density map from panel A, displayed at two contour levels. (E) Superimposition of the high-contour level map from panel D onto its flexibly fitted model.

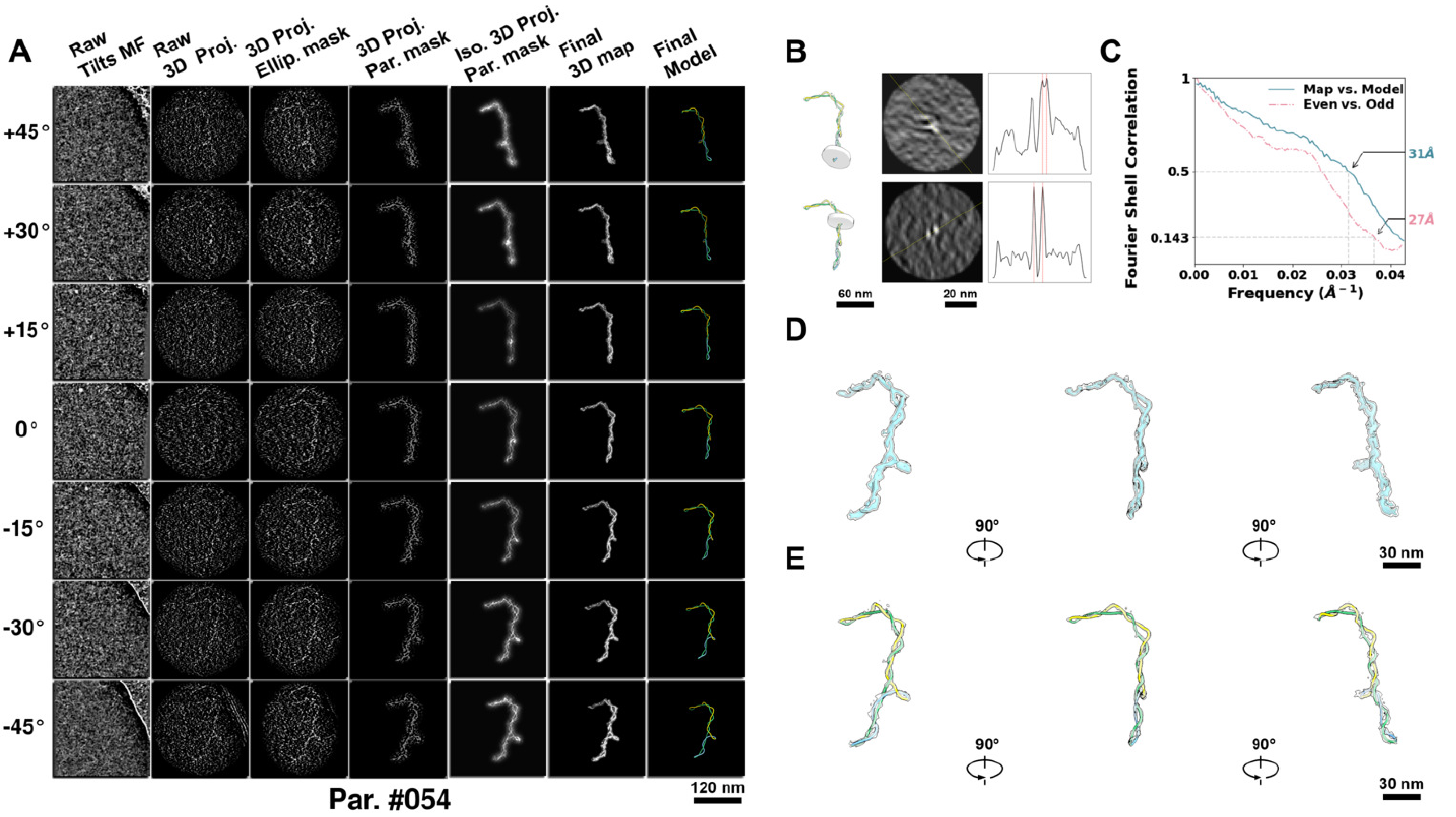

Supplementary Figure 54. Cryo-electron tomography (cryo-ET) 3D reconstruction of an individual plasmid particle in 40 mM K$^+$, 5 mM Mg^{2+}.
(A) 3D reconstruction of the plasmid particle (index no. 54). The first column shows seven representative tilt images from –45° to +45° in step of 15°. The second, third, and fourth columns show 3D projections of the particle with spherical, ellipsoidal (thinner along the z-dimension), and particle-shaped masks, respectively. The fifth column displays the 3D projections of the enhanced and IsoNet missing-wedge-corrected particle. The sixth and seventh columns present the final 3D map and the flexibly fitted model, respectively. (B) Two random cross-sectional views (12 nm thickness) of the plasmid density map along its plectoneme axis are shown in the left-middle panel. The intensity profile along the line crossing the two high-density DNA spots is displayed in the right panel. (C) Resolution assessment of the final 3D map using Fourier shell correlation (FSC). Two criteria are shown: FSC between two half-maps reconstructed from even and odd frames (evaluated at 0.143) and FSC between the final 3D map and the fitted model (evaluated at 0.5). (D) Zoomed-in views of the final 3D density map from panel A, displayed at two contour levels. (E) Superimposition of the high-contour level map from panel D onto its flexibly fitted model.

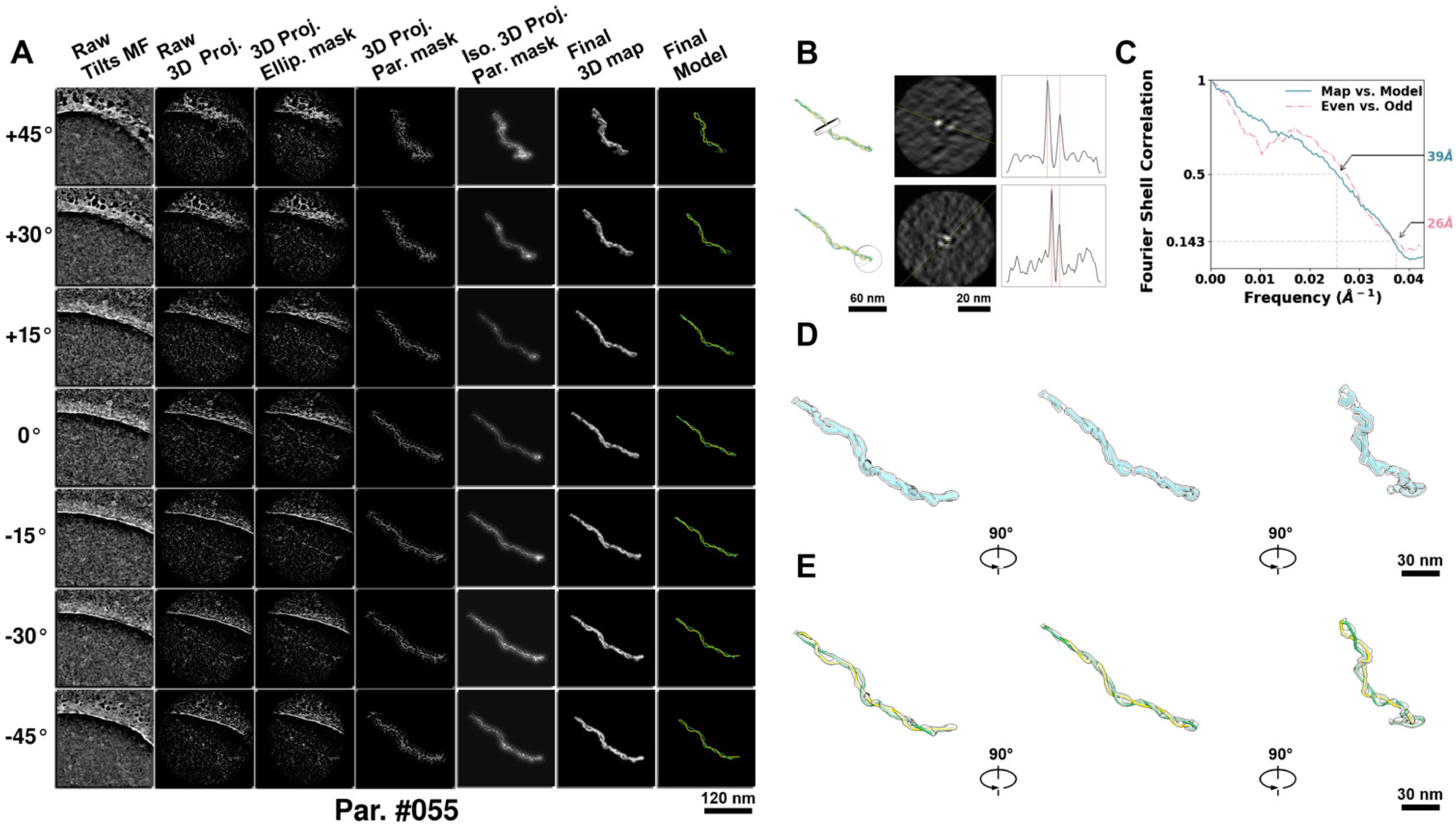

Supplementary Figure 55. Cryo-electron tomography (cryo-ET) 3D reconstruction of an individual plasmid particle in 40 mM K^+, 5 mM Mg^{2+}.
(A) 3D reconstruction of the plasmid particle (index no. 55). The first column shows seven representative tilt images from –45° to +45° in step of 15°. The second, third, and fourth columns show 3D projections of the particle with spherical, ellipsoidal (thinner along the z-dimension), and particle-shaped masks, respectively. The fifth column displays the 3D projections of the enhanced and IsoNet missing-wedge-corrected particle. The sixth and seventh columns present the final 3D map and the flexibly fitted model, respectively. (B) Two random cross-sectional views (12 nm thickness) of the plasmid density map along its plectoneme axis are shown in the left-middle panel. The intensity profile along the line crossing the two high-density DNA spots is displayed in the right panel. (C) Resolution assessment of the final 3D map using Fourier shell correlation (FSC). Two criteria are shown: FSC between two half-maps reconstructed from even and odd frames (evaluated at 0.143) and FSC between the final 3D map and the fitted model (evaluated at 0.5). (D) Zoomed-in views of the final 3D density map from panel A, displayed at two contour levels. (E) Superimposition of the high-contour level map from panel D onto its flexibly fitted model.

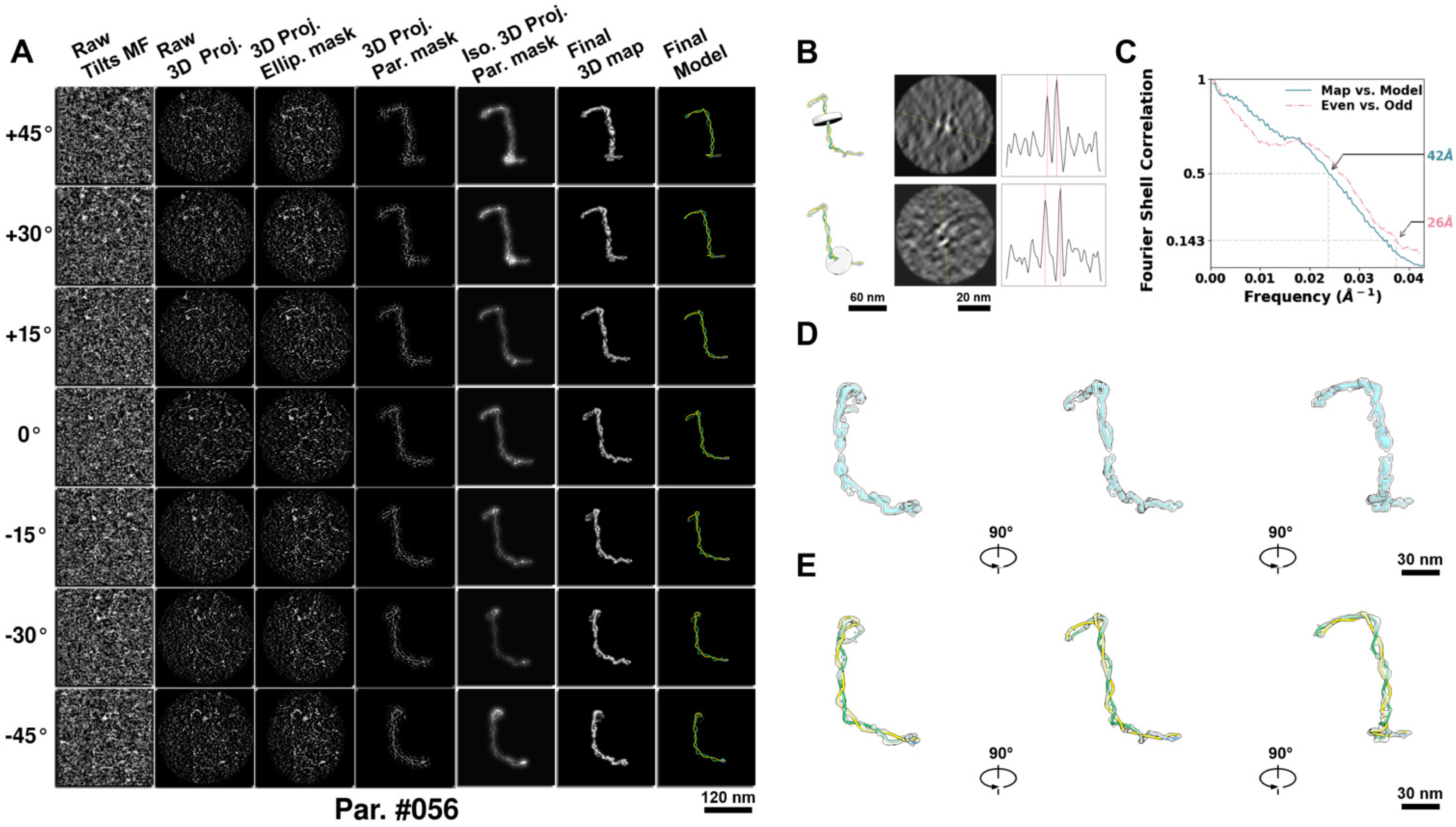

Supplementary Figure 56. Cryo-electron tomography (cryo-ET) 3D reconstruction of an individual plasmid particle in 40 mM K^+, 5 mM Mg^{2+}.
(A) 3D reconstruction of the plasmid particle (index no. 56). The first column shows seven representative tilt images from –45° to +45° in step of 15°. The second, third, and fourth columns show 3D projections of the particle with spherical, ellipsoidal (thinner along the z-dimension), and particle-shaped masks, respectively. The fifth column displays the 3D projections of the enhanced and IsoNet missing-wedge-corrected particle. The sixth and seventh columns present the final 3D map and the flexibly fitted model, respectively. (B) Two random cross-sectional views (12 nm thickness) of the plasmid density map along its plectoneme axis are shown in the left-middle panel. The intensity profile along the line crossing the two high-density DNA spots is displayed in the right panel. (C) Resolution assessment of the final 3D map using Fourier shell correlation (FSC). Two criteria are shown: FSC between two half-maps reconstructed from even and odd frames (evaluated at 0.143) and FSC between the final 3D map and the fitted model (evaluated at 0.5). (D) Zoomed-in views of the final 3D density map from panel A, displayed at two contour levels. (E) Superimposition of the high-contour level map from panel D onto its flexibly fitted model.

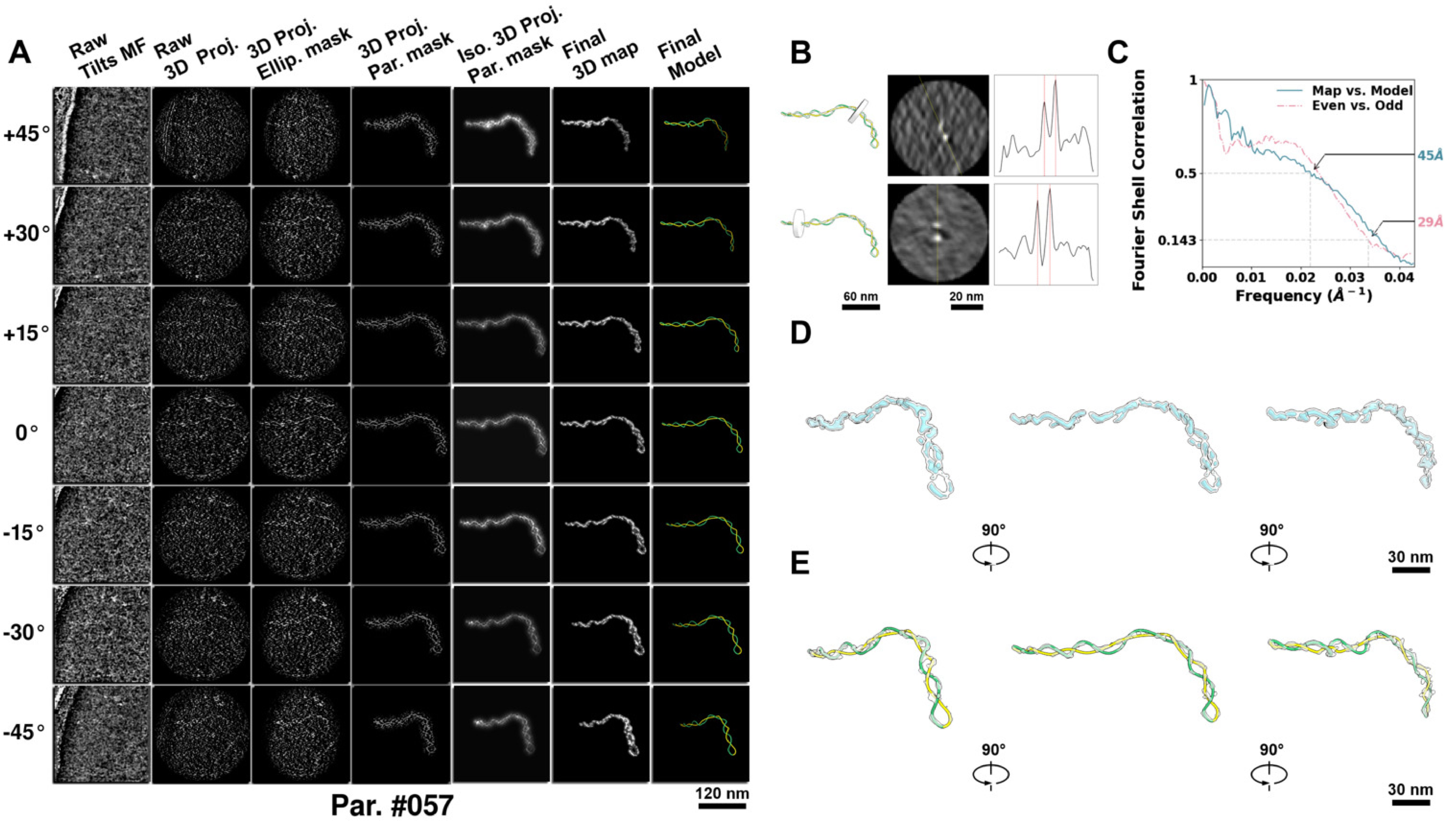

Supplementary Figure 57. Cryo-electron tomography (cryo-ET) 3D reconstruction of an individual plasmid particle in 40 mM K^+, 5 mM Mg^{2+}.
(A) 3D reconstruction of the plasmid particle (index no. 57). The first column shows seven representative tilt images from –45° to +45° in step of 15°. The second, third, and fourth columns show 3D projections of the particle with spherical, ellipsoidal (thinner along the z-dimension), and particle-shaped masks, respectively. The fifth column displays the 3D projections of the enhanced and IsoNet missing-wedge-corrected particle. The sixth and seventh columns present the final 3D map and the flexibly fitted model, respectively. (B) Two random cross-sectional views (12 nm thickness) of the plasmid density map along its plectoneme axis are shown in the left-middle panel. The intensity profile along the line crossing the two high-density DNA spots is displayed in the right panel. (C) Resolution assessment of the final 3D map using Fourier shell correlation (FSC). Two criteria are shown: FSC between two half-maps reconstructed from even and odd frames (evaluated at 0.143) and FSC between the final 3D map and the fitted model (evaluated at 0.5). (D) Zoomed-in views of the final 3D density map from panel A, displayed at two contour levels. (E) Superimposition of the high-contour level map from panel D onto its flexibly fitted model.

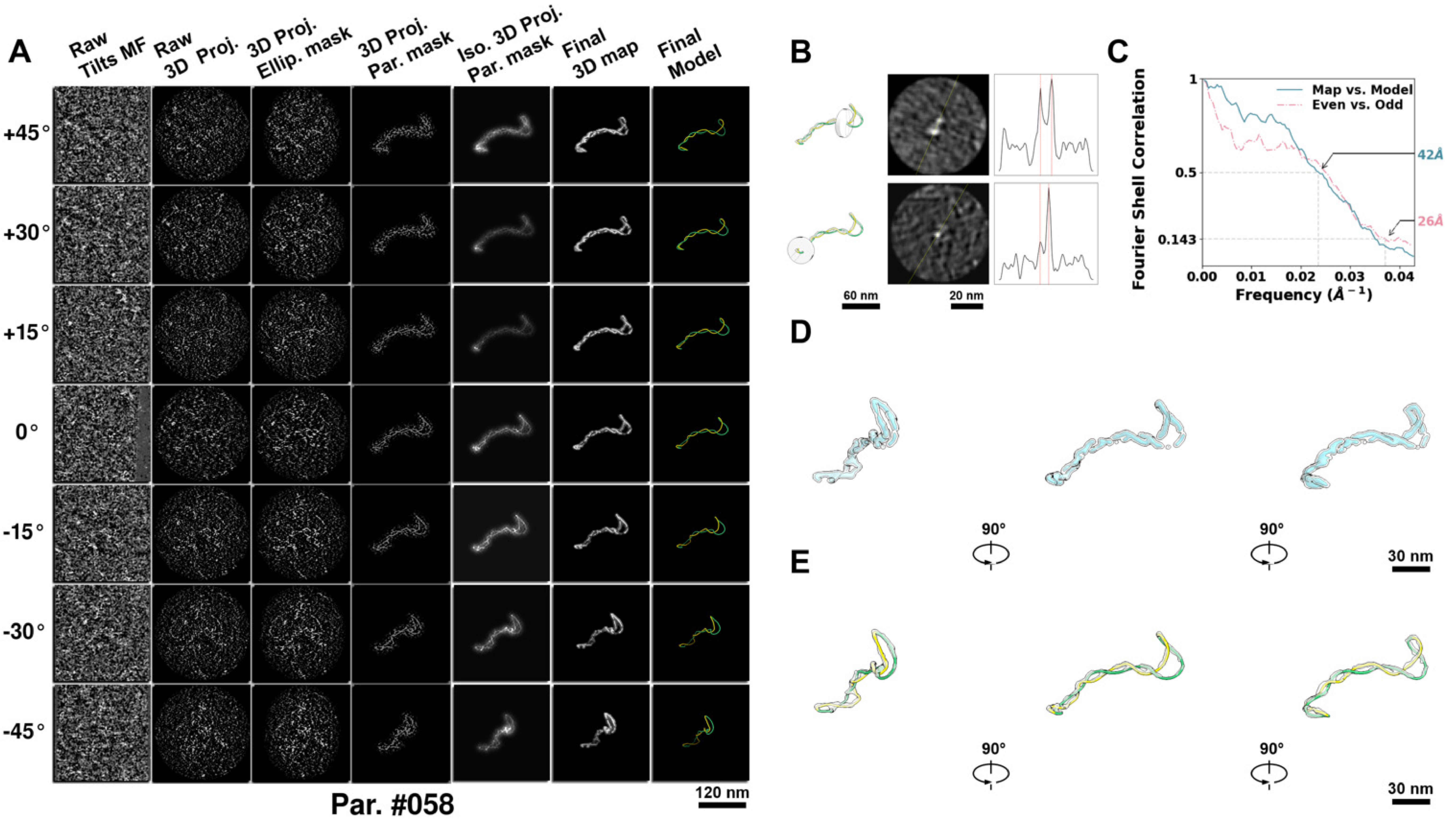

Supplementary Figure 58. Cryo-electron tomography (cryo-ET) 3D reconstruction of an individual plasmid particle in 40 mM K^+, 5 mM Mg^{2+}.
(A) 3D reconstruction of the plasmid particle (index no. 58). The first column shows seven representative tilt images from –45° to +45° in step of 15°. The second, third, and fourth columns show 3D projections of the particle with spherical, ellipsoidal (thinner along the z-dimension), and particle-shaped masks, respectively. The fifth column displays the 3D projections of the enhanced and IsoNet missing-wedge-corrected particle. The sixth and seventh columns present the final 3D map and the flexibly fitted model, respectively. (B) Two random cross-sectional views (12 nm thickness) of the plasmid density map along its plectoneme axis are shown in the left-middle panel. The intensity profile along the line crossing the two high-density DNA spots is displayed in the right panel. (C) Resolution assessment of the final 3D map using Fourier shell correlation (FSC). Two criteria are shown: FSC between two half-maps reconstructed from even and odd frames (evaluated at 0.143) and FSC between the final 3D map and the fitted model (evaluated at 0.5). (D) Zoomed-in views of the final 3D density map from panel A, displayed at two contour levels. (E) Superimposition of the high-contour level map from panel D onto its flexibly fitted model.

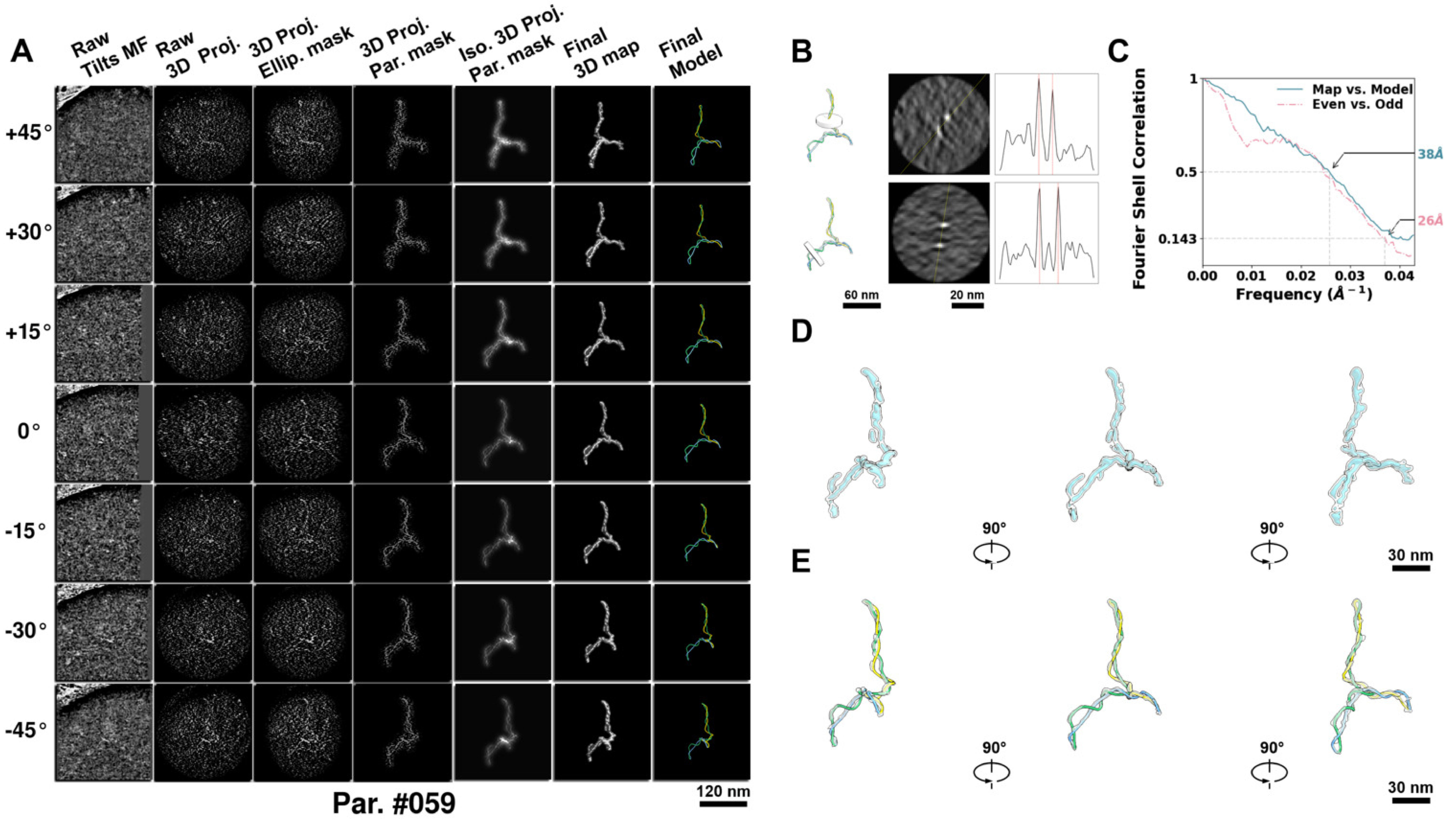

Supplementary Figure 59. Cryo-electron tomography (cryo-ET) 3D reconstruction of an individual plasmid particle in 40 mM K^+, 5 mM Mg^{2+}.
(A) 3D reconstruction of the plasmid particle (index no. 59). The first column shows seven representative tilt images from –45° to +45° in step of 15°. The second, third, and fourth columns show 3D projections of the particle with spherical, ellipsoidal (thinner along the z-dimension), and particle-shaped masks, respectively. The fifth column displays the 3D projections of the enhanced and IsoNet missing-wedge-corrected particle. The sixth and seventh columns present the final 3D map and the flexibly fitted model, respectively. (B) Two random cross-sectional views (12 nm thickness) of the plasmid density map along its plectoneme axis are shown in the left-middle panel. The intensity profile along the line crossing the two high-density DNA spots is displayed in the right panel. (C) Resolution assessment of the final 3D map using Fourier shell correlation (FSC). Two criteria are shown: FSC between two half-maps reconstructed from even and odd frames (evaluated at 0.143) and FSC between the final 3D map and the fitted model (evaluated at 0.5). (D) Zoomed-in views of the final 3D density map from panel A, displayed at two contour levels. (E) Superimposition of the high-contour level map from panel D onto its flexibly fitted model.

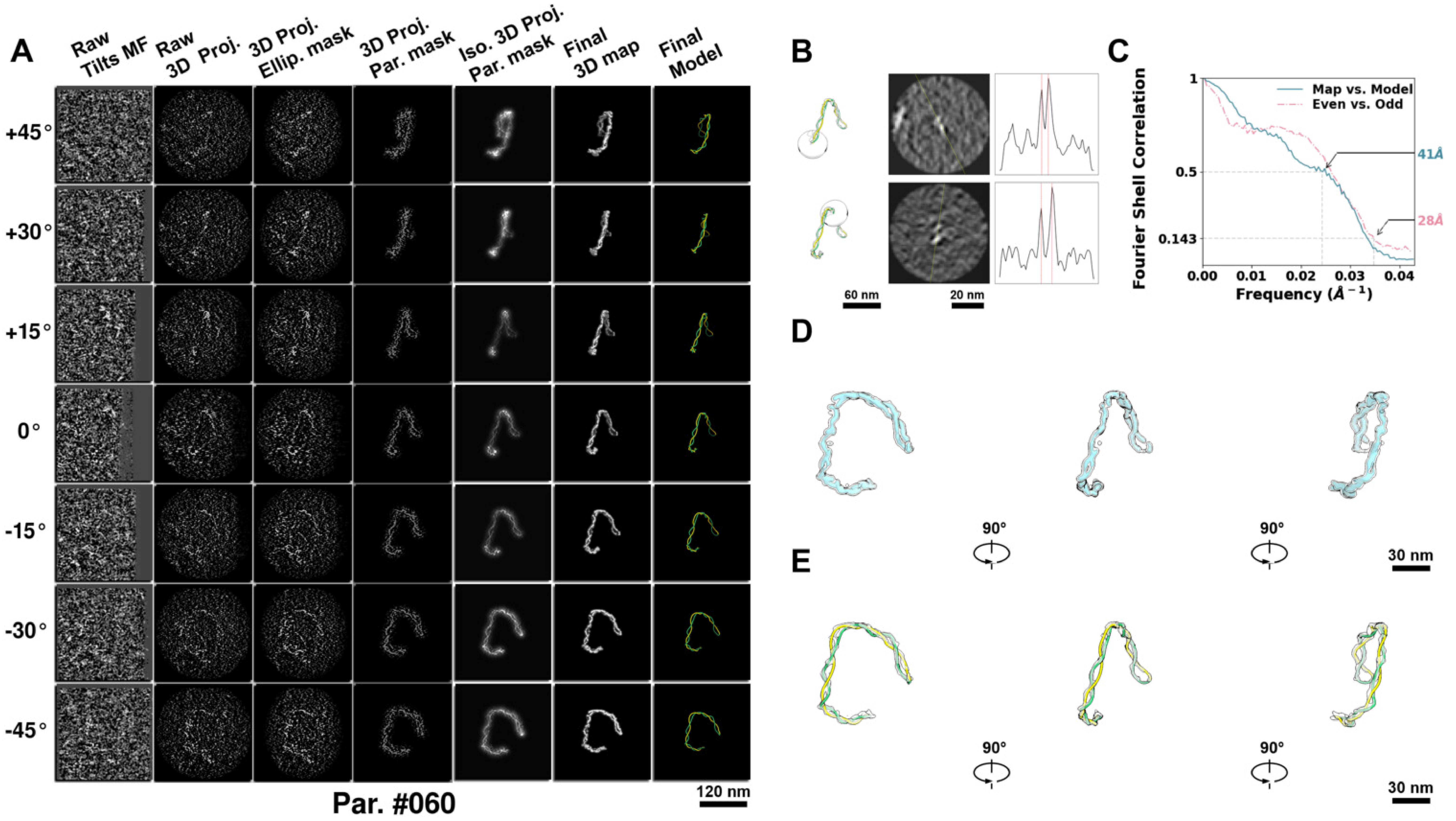

Supplementary Figure 60. Cryo-electron tomography (cryo-ET) 3D reconstruction of an individual plasmid particle in 40 mM K^+, 5 mM Mg^{2+}.
(A) 3D reconstruction of the plasmid particle (index no. 60). The first column shows seven representative tilt images from –45° to +45° in step of 15°. The second, third, and fourth columns show 3D projections of the particle with spherical, ellipsoidal (thinner along the z-dimension), and particle-shaped masks, respectively. The fifth column displays the 3D projections of the enhanced and IsoNet missing-wedge-corrected particle. The sixth and seventh columns present the final 3D map and the flexibly fitted model, respectively. (B) Two random cross-sectional views (12 nm thickness) of the plasmid density map along its plectoneme axis are shown in the left-middle panel. The intensity profile along the line crossing the two high-density DNA spots is displayed in the right panel. (C) Resolution assessment of the final 3D map using Fourier shell correlation (FSC). Two criteria are shown: FSC between two half-maps reconstructed from even and odd frames (evaluated at 0.143) and FSC between the final 3D map and the fitted model (evaluated at 0.5). (D) Zoomed-in views of the final 3D density map from panel A, displayed at two contour levels. (E) Superimposition of the high-contour level map from panel D onto its flexibly fitted model.

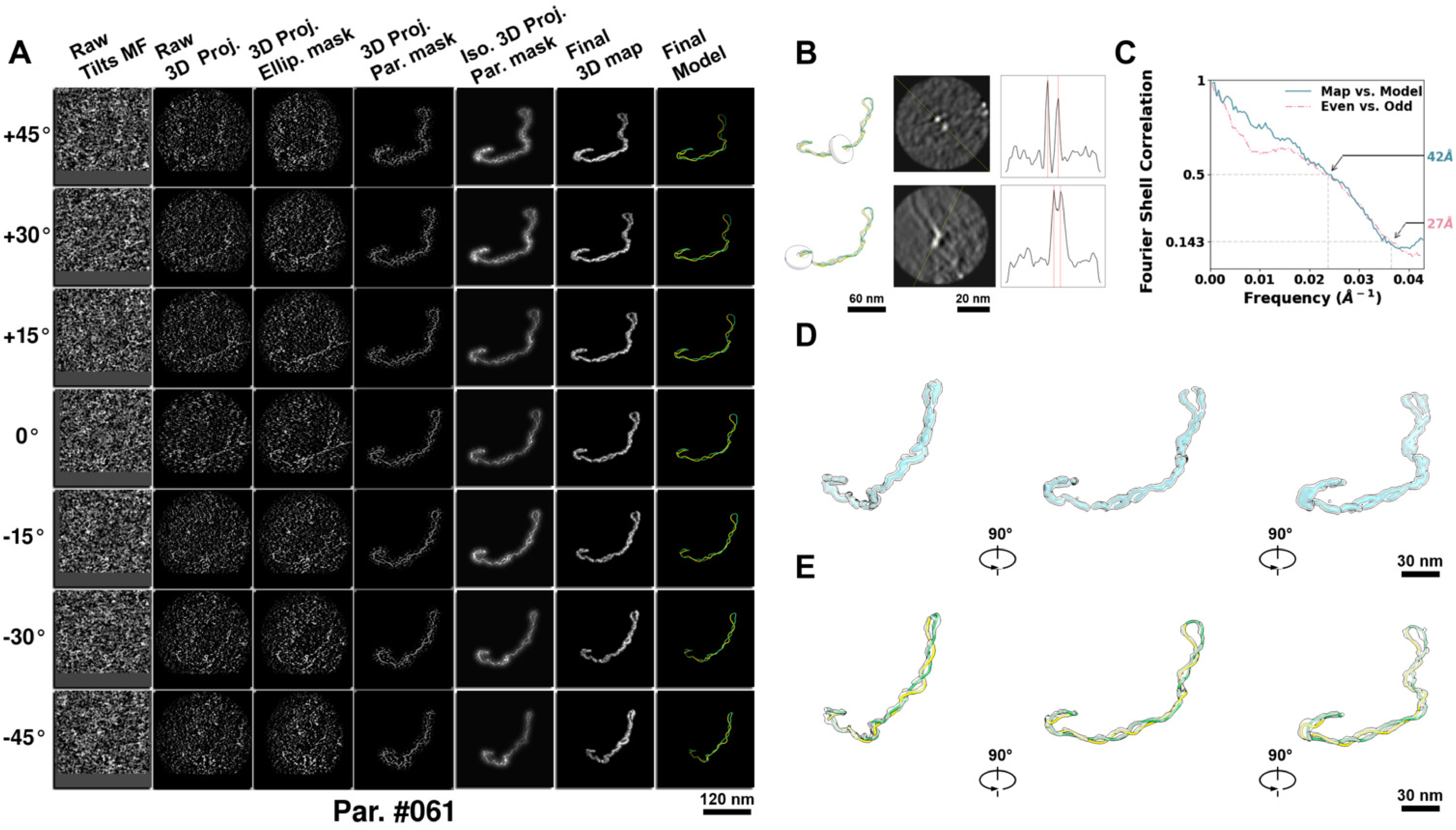

Supplementary Figure 61. Cryo-electron tomography (cryo-ET) 3D reconstruction of an individual plasmid particle in 40 mM K^+, 5 mM Mg^{2+}.
(A) 3D reconstruction of the plasmid particle (index no. 61). The first column shows seven representative tilt images from –45° to +45° in step of 15°. The second, third, and fourth columns show 3D projections of the particle with spherical, ellipsoidal (thinner along the z-dimension), and particle-shaped masks, respectively. The fifth column displays the 3D projections of the enhanced and IsoNet missing-wedge-corrected particle. The sixth and seventh columns present the final 3D map and the flexibly fitted model, respectively. (B) Two random cross-sectional views (12 nm thickness) of the plasmid density map along its plectoneme axis are shown in the left-middle panel. The intensity profile along the line crossing the two high-density DNA spots is displayed in the right panel. (C) Resolution assessment of the final 3D map using Fourier shell correlation (FSC). Two criteria are shown: FSC between two half-maps reconstructed from even and odd frames (evaluated at 0.143) and FSC between the final 3D map and the fitted model (evaluated at 0.5). (D) Zoomed-in views of the final 3D density map from panel A, displayed at two contour levels. (E) Superimposition of the high-contour level map from panel D onto its flexibly fitted model.

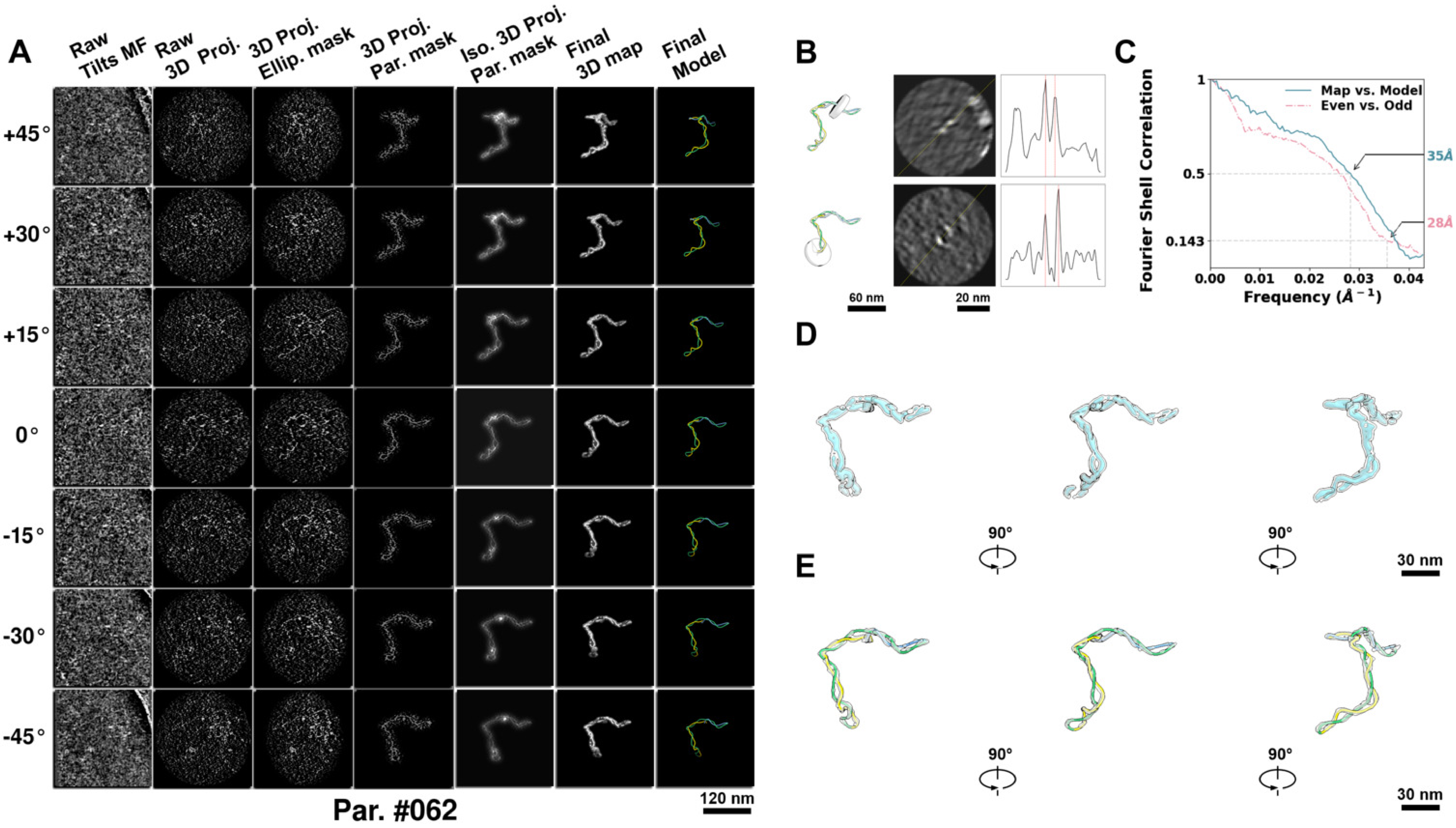

Supplementary Figure 62. Cryo-electron tomography (cryo-ET) 3D reconstruction of an individual plasmid particle in 40 mM K^+, 5 mM Mg^{2+}.
(A) 3D reconstruction of the plasmid particle (index no. 62). The first column shows seven representative tilt images from –45° to +45° in step of 15°. The second, third, and fourth columns show 3D projections of the particle with spherical, ellipsoidal (thinner along the z-dimension), and particle-shaped masks, respectively. The fifth column displays the 3D projections of the enhanced and IsoNet missing-wedge-corrected particle. The sixth and seventh columns present the final 3D map and the flexibly fitted model, respectively. (B) Two random cross-sectional views (12 nm thickness) of the plasmid density map along its plectoneme axis are shown in the left-middle panel. The intensity profile along the line crossing the two high-density DNA spots is displayed in the right panel. (C) Resolution assessment of the final 3D map using Fourier shell correlation (FSC). Two criteria are shown: FSC between two half-maps reconstructed from even and odd frames (evaluated at 0.143) and FSC between the final 3D map and the fitted model (evaluated at 0.5). (D) Zoomed-in views of the final 3D density map from panel A, displayed at two contour levels. (E) Superimposition of the high-contour level map from panel D onto its flexibly fitted model.

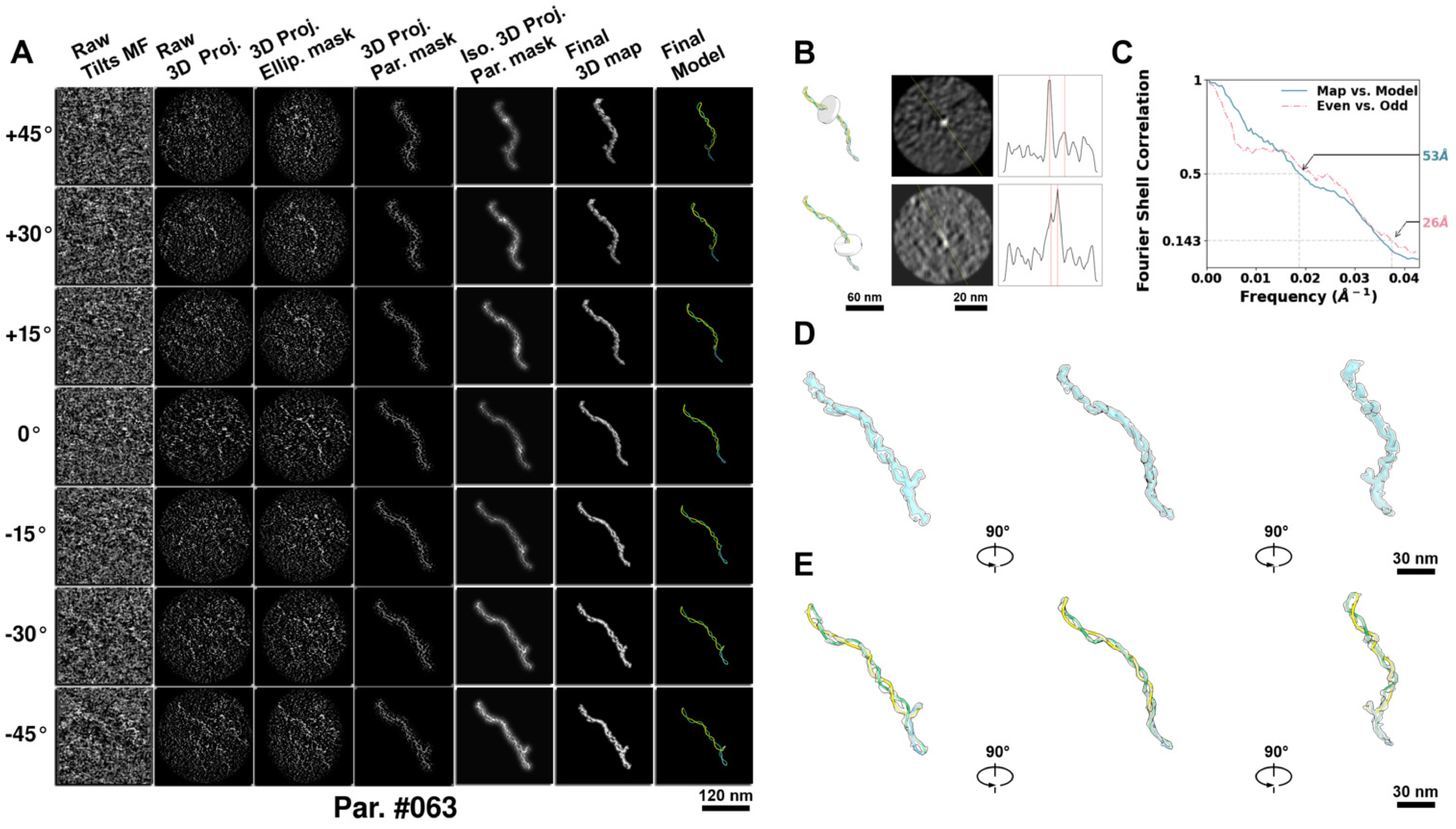

Supplementary Figure 63. Cryo-electron tomography (cryo-ET) 3D reconstruction of an individual plasmid particle in 40 mM K^+, 5 mM Mg^{2+}.
(A) 3D reconstruction of the plasmid particle (index no. 63). The first column shows seven representative tilt images from –45° to +45° in step of 15°. The second, third, and fourth columns show 3D projections of the particle with spherical, ellipsoidal (thinner along the z-dimension), and particle-shaped masks, respectively. The fifth column displays the 3D projections of the enhanced and IsoNet missing-wedge-corrected particle. The sixth and seventh columns present the final 3D map and the flexibly fitted model, respectively. (B) Two random cross-sectional views (12 nm thickness) of the plasmid density map along its plectoneme axis are shown in the left-middle panel. The intensity profile along the line crossing the two high-density DNA spots is displayed in the right panel. (C) Resolution assessment of the final 3D map using Fourier shell correlation (FSC). Two criteria are shown: FSC between two half-maps reconstructed from even and odd frames (evaluated at 0.143) and FSC between the final 3D map and the fitted model (evaluated at 0.5). (D) Zoomed-in views of the final 3D density map from panel A, displayed at two contour levels. (E) Superimposition of the high-contour level map from panel D onto its flexibly fitted model.

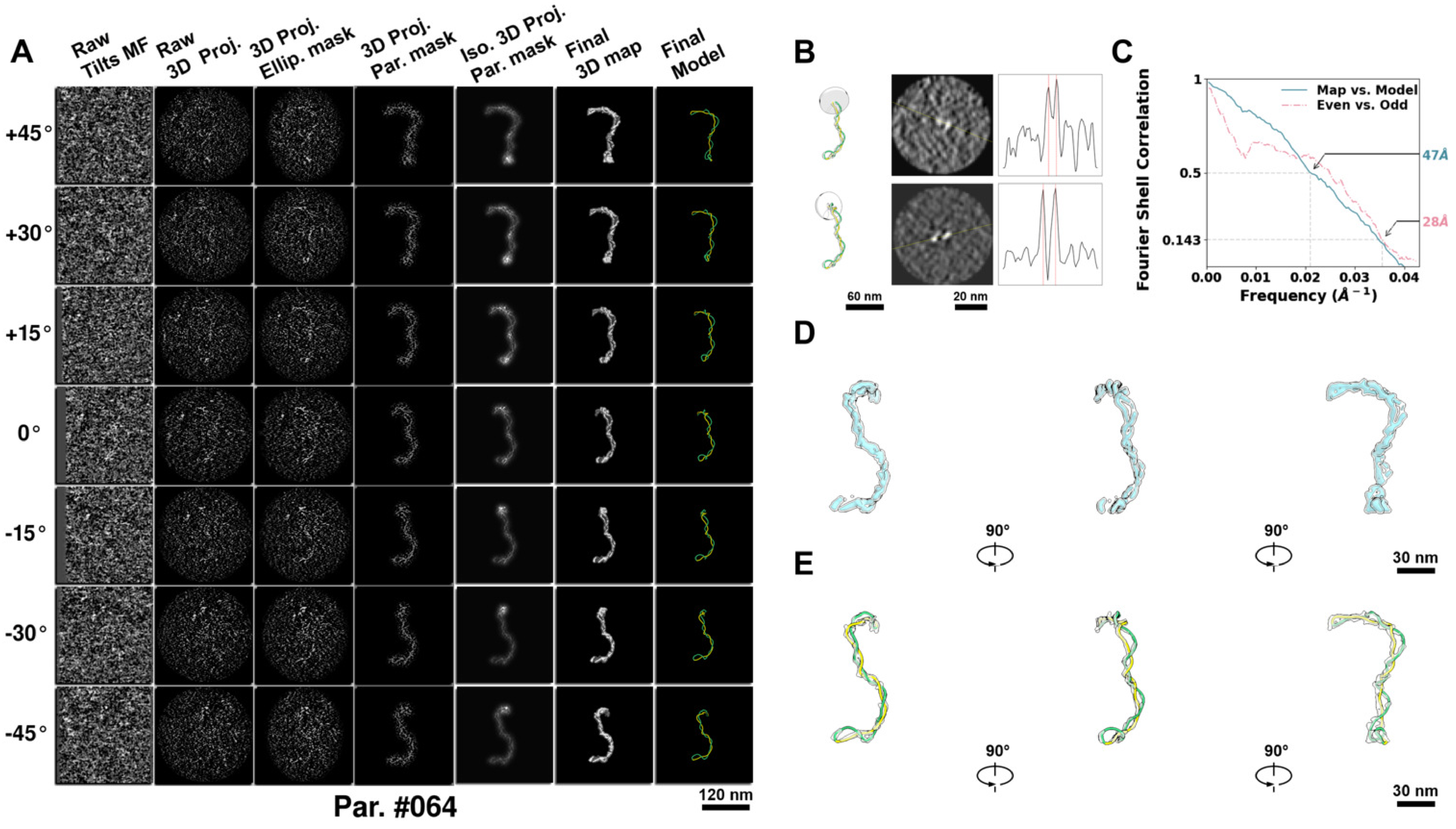

Supplementary Figure 64. Cryo-electron tomography (cryo-ET) 3D reconstruction of an individual plasmid particle in 40 mM K^+, 5 mM Mg^{2+}.
(A) 3D reconstruction of the plasmid particle (index no. 64). The first column shows seven representative tilt images from –45° to +45° in step of 15°. The second, third, and fourth columns show 3D projections of the particle with spherical, ellipsoidal (thinner along the z-dimension), and particle-shaped masks, respectively. The fifth column displays the 3D projections of the enhanced and IsoNet missing-wedge-corrected particle. The sixth and seventh columns present the final 3D map and the flexibly fitted model, respectively. (B) Two random cross-sectional views (12 nm thickness) of the plasmid density map along its plectoneme axis are shown in the left-middle panel. The intensity profile along the line crossing the two high-density DNA spots is displayed in the right panel. (C) Resolution assessment of the final 3D map using Fourier shell correlation (FSC). Two criteria are shown: FSC between two half-maps reconstructed from even and odd frames (evaluated at 0.143) and FSC between the final 3D map and the fitted model (evaluated at 0.5). (D) Zoomed-in views of the final 3D density map from panel A, displayed at two contour levels. (E) Superimposition of the high-contour level map from panel D onto its flexibly fitted model.

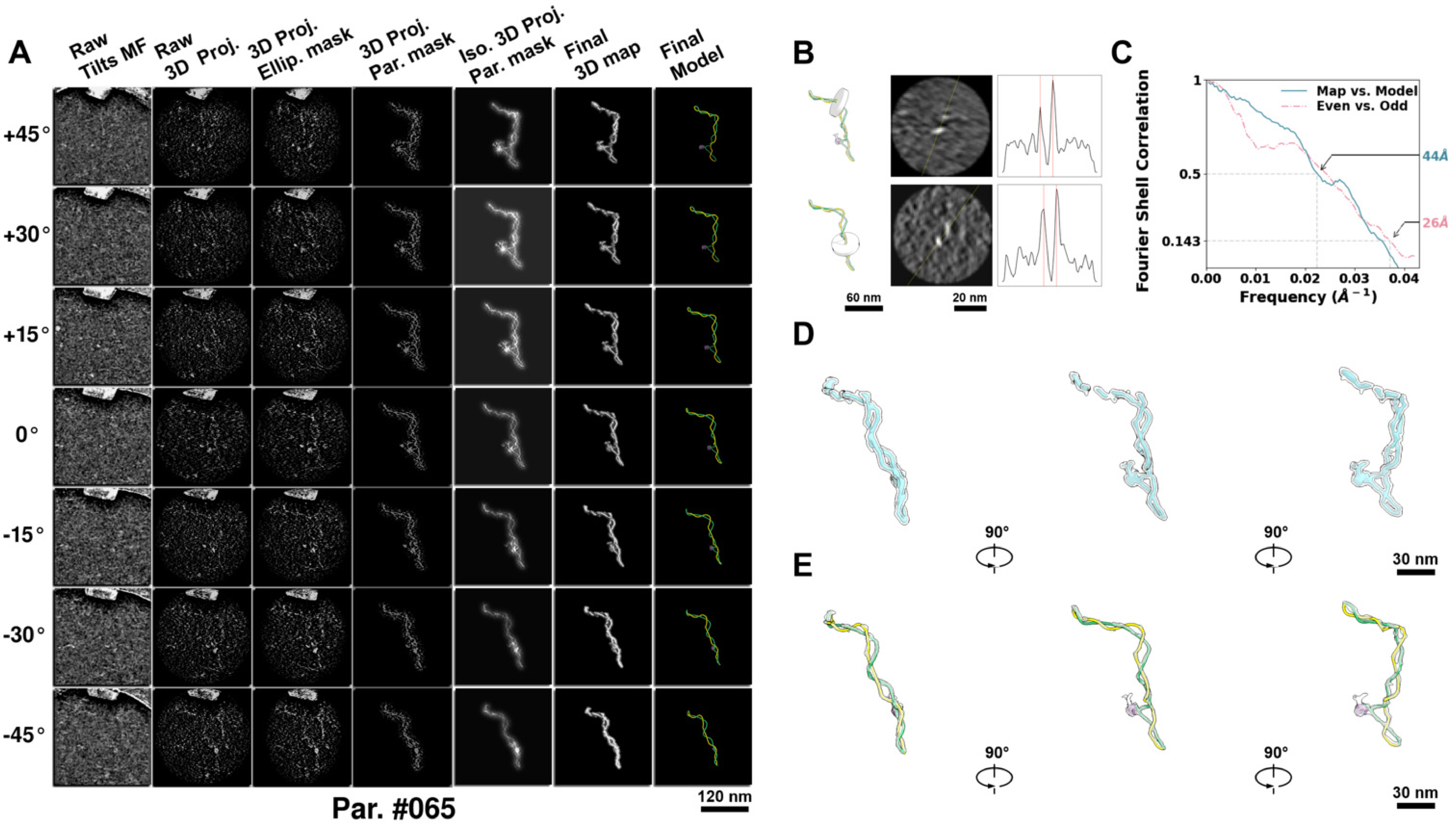

Supplementary Figure 65. Cryo-electron tomography (cryo-ET) 3D reconstruction of an individual plasmid particle in 40 mM K^+, 5 mM Mg^{2+} in the presence of stalled RNAP.
(A) 3D reconstruction of the plasmid particle (index no. 65). The first column shows seven representative tilt images from –45° to +45° in step of 15°. The second, third, and fourth columns show 3D projections of the particle with spherical, ellipsoidal (thinner along the z-dimension), and particle-shaped masks, respectively. The fifth column displays the 3D projections of the enhanced and IsoNet missing-wedge-corrected particle. The sixth and seventh columns present the final 3D map and the flexibly fitted model, respectively. (B) Two random cross-sectional views (12 nm thickness) of the plasmid density map along its plectoneme axis are shown in the left-middle panel. The intensity profile along the line crossing the two high-density DNA spots is displayed in the right panel. (C) Resolution assessment of the final 3D map using Fourier shell correlation (FSC). Two criteria are shown: FSC between two half-maps reconstructed from even and odd frames (evaluated at 0.143) and FSC between the final 3D map and the fitted model (evaluated at 0.5). (D) Zoomed-in views of the final 3D density map from panel A, displayed at two contour levels. (E) Superimposition of the high-contour level map from panel D onto its flexibly fitted model.

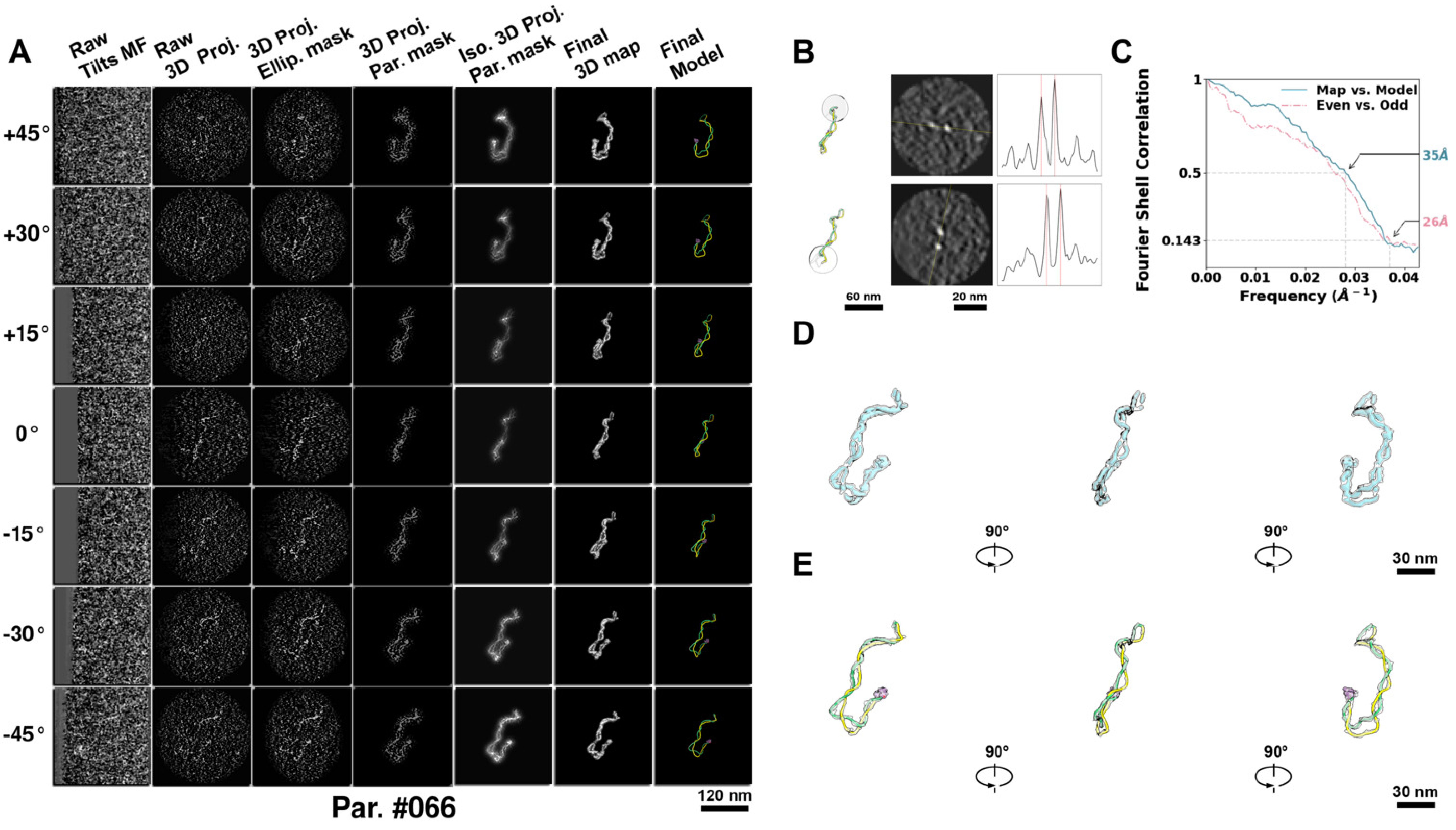

Supplementary Figure 66. Cryo-electron tomography (cryo-ET) 3D reconstruction of an individual plasmid particle in 40 mM K^+, 5 mM Mg^{2+} in the presence of stalled RNAP.
(A) 3D reconstruction of the plasmid particle (index no. 66). The first column shows seven representative tilt images from –45° to +45° in step of 15°. The second, third, and fourth columns show 3D projections of the particle with spherical, ellipsoidal (thinner along the z-dimension), and particle-shaped masks, respectively. The fifth column displays the 3D projections of the enhanced and IsoNet missing-wedge-corrected particle. The sixth and seventh columns present the final 3D map and the flexibly fitted model, respectively. (B) Two random cross-sectional views (12 nm thickness) of the plasmid density map along its plectoneme axis are shown in the left-middle panel. The intensity profile along the line crossing the two high-density DNA spots is displayed in the right panel. (C) Resolution assessment of the final 3D map using Fourier shell correlation (FSC). Two criteria are shown: FSC between two half-maps reconstructed from even and odd frames (evaluated at 0.143) and FSC between the final 3D map and the fitted model (evaluated at 0.5). (D) Zoomed-in views of the final 3D density map from panel A, displayed at two contour levels. (E) Superimposition of the high-contour level map from panel D onto its flexibly fitted model.

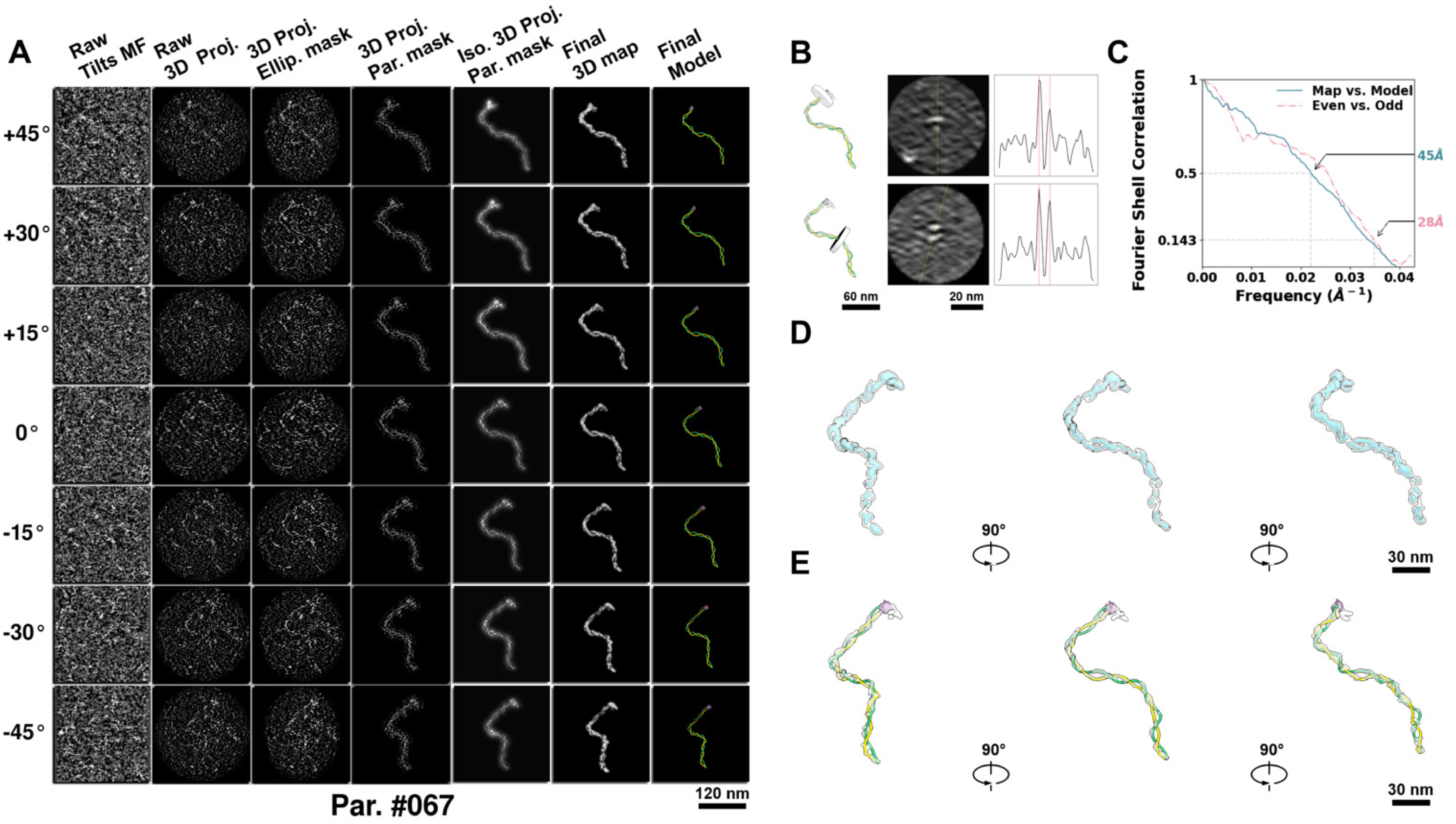

Supplementary Figure 67. Cryo-electron tomography (cryo-ET) 3D reconstruction of an individual plasmid particle in 40 mM K^+, 5 mM Mg^{2+} in the presence of stalled RNAP.
(A) 3D reconstruction of the plasmid particle (index no. 67). The first column shows seven representative tilt images from –45° to +45° in step of 15°. The second, third, and fourth columns show 3D projections of the particle with spherical, ellipsoidal (thinner along the z-dimension), and particle-shaped masks, respectively. The fifth column displays the 3D projections of the enhanced and IsoNet missing-wedge-corrected particle. The sixth and seventh columns present the final 3D map and the flexibly fitted model, respectively. (B) Two random cross-sectional views (12 nm thickness) of the plasmid density map along its plectoneme axis are shown in the left-middle panel. The intensity profile along the line crossing the two high-density DNA spots is displayed in the right panel. (C) Resolution assessment of the final 3D map using Fourier shell correlation (FSC). Two criteria are shown: FSC between two half-maps reconstructed from even and odd frames (evaluated at 0.143) and FSC between the final 3D map and the fitted model (evaluated at 0.5). (D) Zoomed-in views of the final 3D density map from panel A, displayed at two contour levels. (E) Superimposition of the high-contour level map from panel D onto its flexibly fitted model.

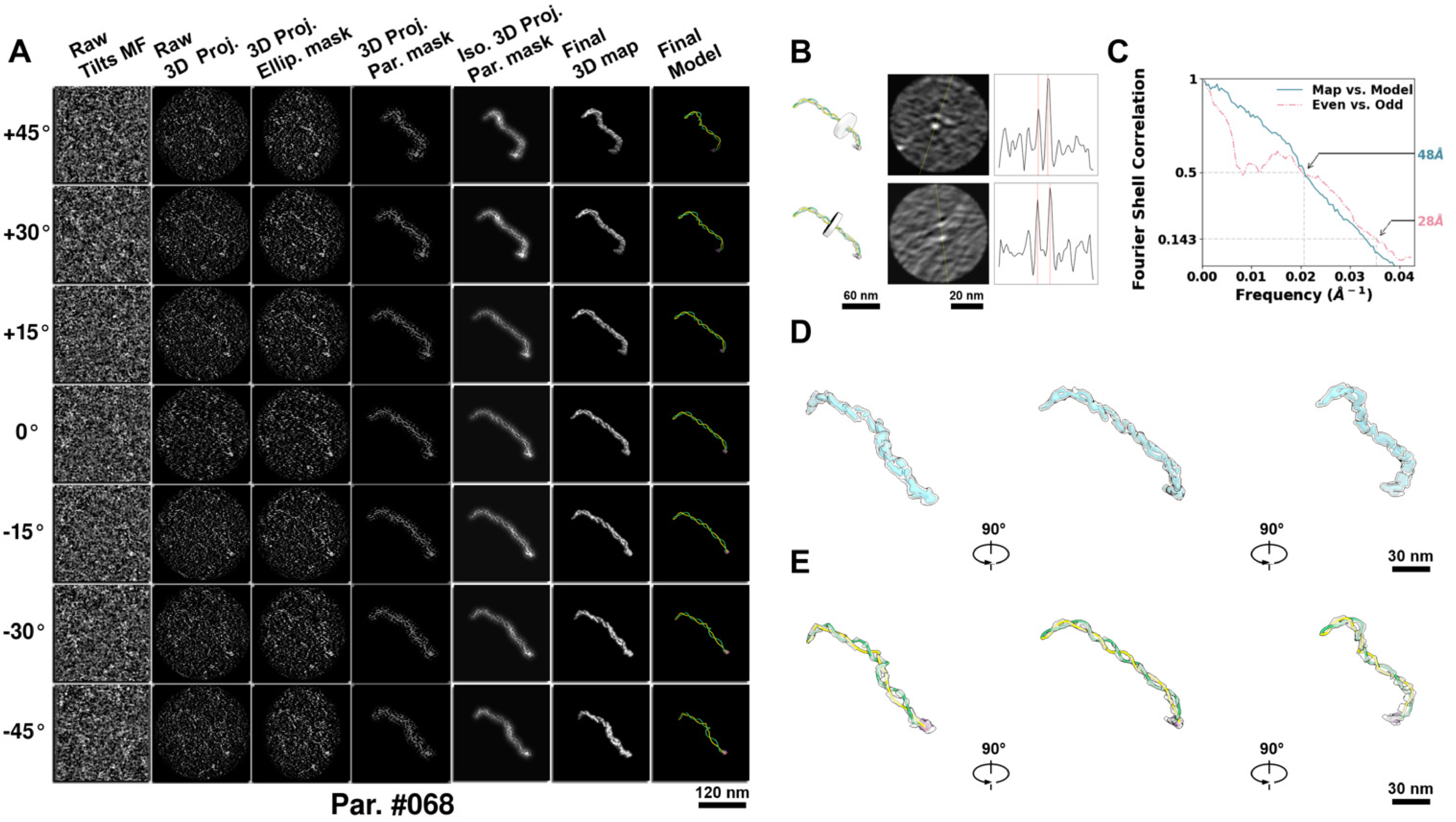

Supplementary Figure 68. Cryo-electron tomography (cryo-ET) 3D reconstruction of an individual plasmid particle in 40 mM K^+, 5 mM Mg^{2+} in the presence of stalled RNAP.
(A) 3D reconstruction of the plasmid particle (index no. 68). The first column shows seven representative tilt images from –45° to +45° in step of 15°. The second, third, and fourth columns show 3D projections of the particle with spherical, ellipsoidal (thinner along the z-dimension), and particle-shaped masks, respectively. The fifth column displays the 3D projections of the enhanced and IsoNet missing-wedge-corrected particle. The sixth and seventh columns present the final 3D map and the flexibly fitted model, respectively. (B) Two random cross-sectional views (12 nm thickness) of the plasmid density map along its plectoneme axis are shown in the left-middle panel. The intensity profile along the line crossing the two high-density DNA spots is displayed in the right panel. (C) Resolution assessment of the final 3D map using Fourier shell correlation (FSC). Two criteria are shown: FSC between two half-maps reconstructed from even and odd frames (evaluated at 0.143) and FSC between the final 3D map and the fitted model (evaluated at 0.5). (D) Zoomed-in views of the final 3D density map from panel A, displayed at two contour levels. (E) Superimposition of the high-contour level map from panel D onto its flexibly fitted model.

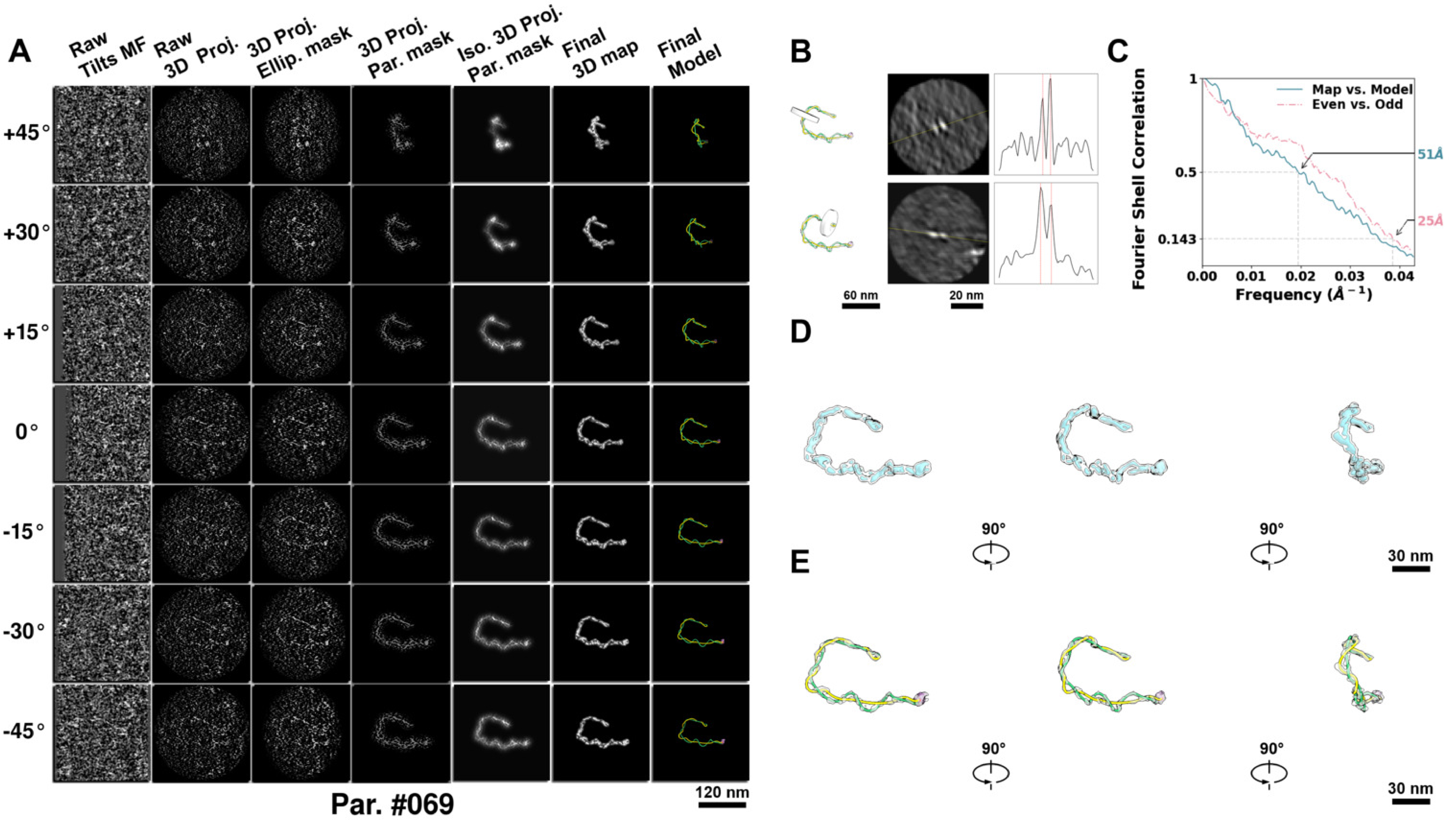

Supplementary Figure 69. Cryo-electron tomography (cryo-ET) 3D reconstruction of an individual plasmid particle in 40 mM K$^+$, 5 mM Mg^{2+} in the presence of stalled RNAP.
(A) 3D reconstruction of the plasmid particle (index no. 69). The first column shows seven representative tilt images from –45° to +45° in step of 15°. The second, third, and fourth columns show 3D projections of the particle with spherical, ellipsoidal (thinner along the z-dimension), and particle-shaped masks, respectively. The fifth column displays the 3D projections of the enhanced and IsoNet missing-wedge-corrected particle. The sixth and seventh columns present the final 3D map and the flexibly fitted model, respectively. (B) Two random cross-sectional views (12 nm thickness) of the plasmid density map along its plectoneme axis are shown in the left-middle panel. The intensity profile along the line crossing the two high-density DNA spots is displayed in the right panel. (C) Resolution assessment of the final 3D map using Fourier shell correlation (FSC). Two criteria are shown: FSC between two half-maps reconstructed from even and odd frames (evaluated at 0.143) and FSC between the final 3D map and the fitted model (evaluated at 0.5). (D) Zoomed-in views of the final 3D density map from panel A, displayed at two contour levels. (E) Superimposition of the high-contour level map from panel D onto its flexibly fitted model.

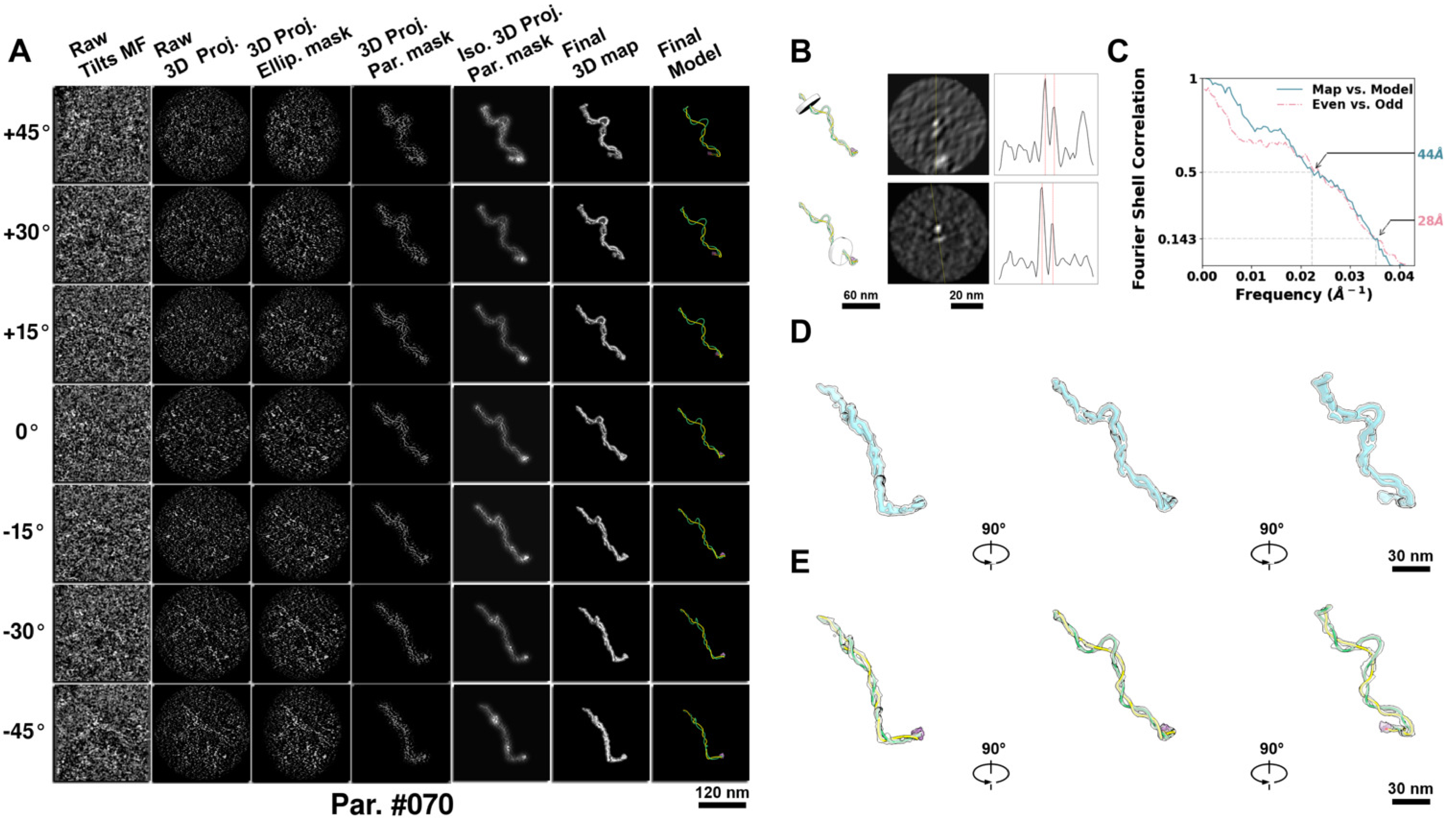

Supplementary Figure 70. Cryo-electron tomography (cryo-ET) 3D reconstruction of an individual plasmid particle in 40 mM K$^+$, 5 mM Mg^{2+} in the presence of stalled RNAP.
(A) 3D reconstruction of the plasmid particle (index no. 70). The first column shows seven representative tilt images from –45° to +45° in step of 15°. The second, third, and fourth columns show 3D projections of the particle with spherical, ellipsoidal (thinner along the z-dimension), and particle-shaped masks, respectively. The fifth column displays the 3D projections of the enhanced and IsoNet missing-wedge-corrected particle. The sixth and seventh columns present the final 3D map and the flexibly fitted model, respectively. (B) Two random cross-sectional views (12 nm thickness) of the plasmid density map along its plectoneme axis are shown in the left-middle panel. The intensity profile along the line crossing the two high-density DNA spots is displayed in the right panel. (C) Resolution assessment of the final 3D map using Fourier shell correlation (FSC). Two criteria are shown: FSC between two half-maps reconstructed from even and odd frames (evaluated at 0.143) and FSC between the final 3D map and the fitted model (evaluated at 0.5). (D) Zoomed-in views of the final 3D density map from panel A, displayed at two contour levels. (E) Superimposition of the high-contour level map from panel D onto its flexibly fitted model.

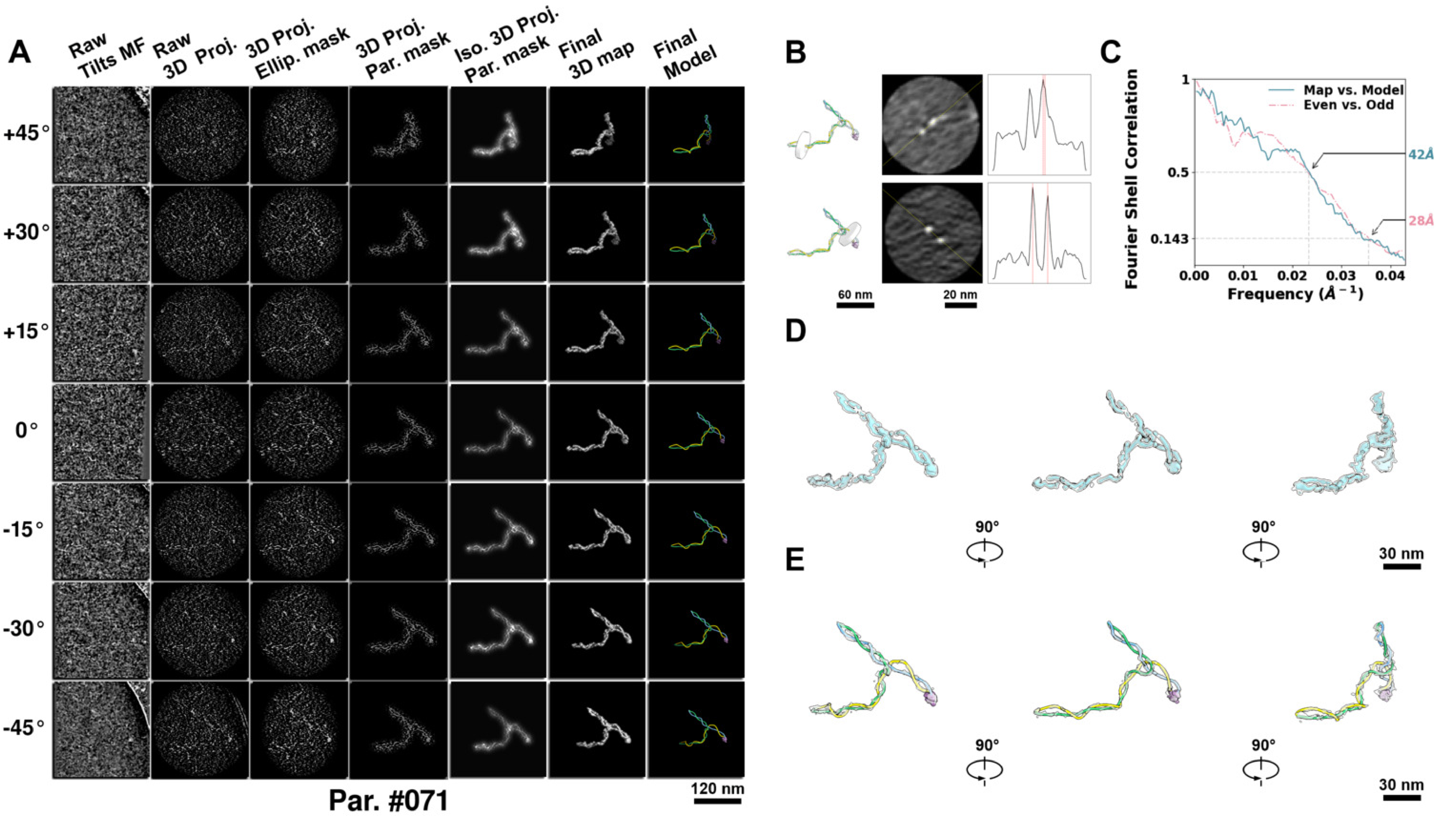

Supplementary Figure 71. Cryo-electron tomography (cryo-ET) 3D reconstruction of an individual plasmid particle in 40 mM K^{+}, 5 mM Mg^{2+} in the presence of stalled RNAP.
(A) 3D reconstruction of the plasmid particle (index no. 71). The first column shows seven representative tilt images from –45° to +45° in step of 15°. The second, third, and fourth columns show 3D projections of the particle with spherical, ellipsoidal (thinner along the z-dimension), and particle-shaped masks, respectively. The fifth column displays the 3D projections of the enhanced and IsoNet missing-wedge-corrected particle. The sixth and seventh columns present the final 3D map and the flexibly fitted model, respectively. (B) Two random cross-sectional views (12 nm thickness) of the plasmid density map along its plectoneme axis are shown in the left-middle panel. The intensity profile along the line crossing the two high-density DNA spots is displayed in the right panel. (C) Resolution assessment of the final 3D map using Fourier shell correlation (FSC). Two criteria are shown: FSC between two half-maps reconstructed from even and odd frames (evaluated at 0.143) and FSC between the final 3D map and the fitted model (evaluated at 0.5). (D) Zoomed-in views of the final 3D density map from panel A, displayed at two contour levels. (E) Superimposition of the high-contour level map from panel D onto its flexibly fitted model.

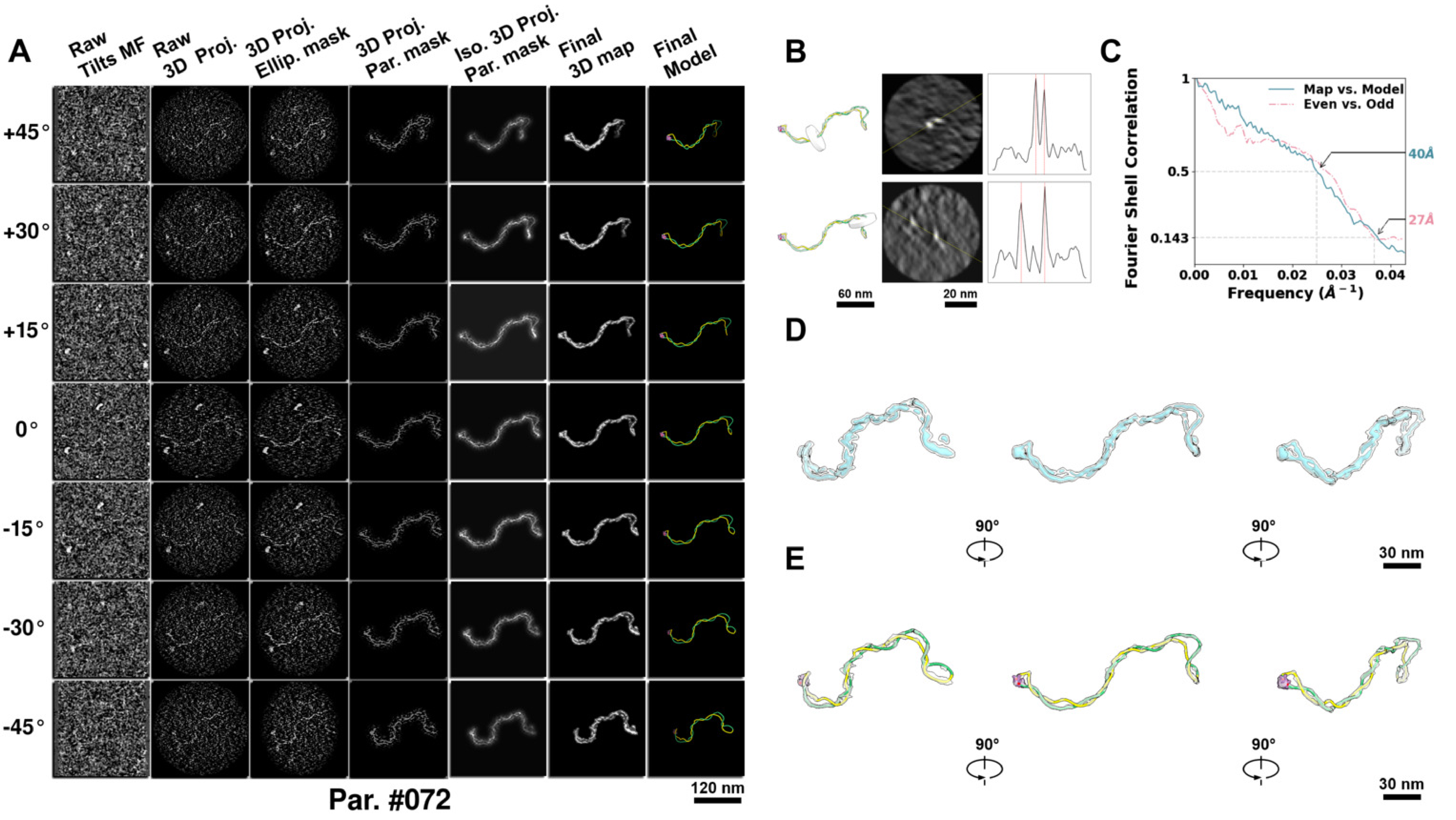

Supplementary Figure 72. Cryo-electron tomography (cryo-ET) 3D reconstruction of an individual plasmid particle in 40 mM K^{+}, 5 mM Mg^{2+} in the presence of stalled RNAP.
(A) 3D reconstruction of the plasmid particle (index no. 72). The first column shows seven representative tilt images from –45° to +45° in step of 15°. The second, third, and fourth columns show 3D projections of the particle with spherical, ellipsoidal (thinner along the z-dimension), and particle-shaped masks, respectively. The fifth column displays the 3D projections of the enhanced and IsoNet missing-wedge-corrected particle. The sixth and seventh columns present the final 3D map and the flexibly fitted model, respectively. (B) Two random cross-sectional views (12 nm thickness) of the plasmid density map along its plectoneme axis are shown in the left-middle panel. The intensity profile along the line crossing the two high-density DNA spots is displayed in the right panel. (C) Resolution assessment of the final 3D map using Fourier shell correlation (FSC). Two criteria are shown: FSC between two half-maps reconstructed from even and odd frames (evaluated at 0.143) and FSC between the final 3D map and the fitted model (evaluated at 0.5). (D) Zoomed-in views of the final 3D density map from panel A, displayed at two contour levels. (E) Superimposition of the high-contour level map from panel D onto its flexibly fitted model.

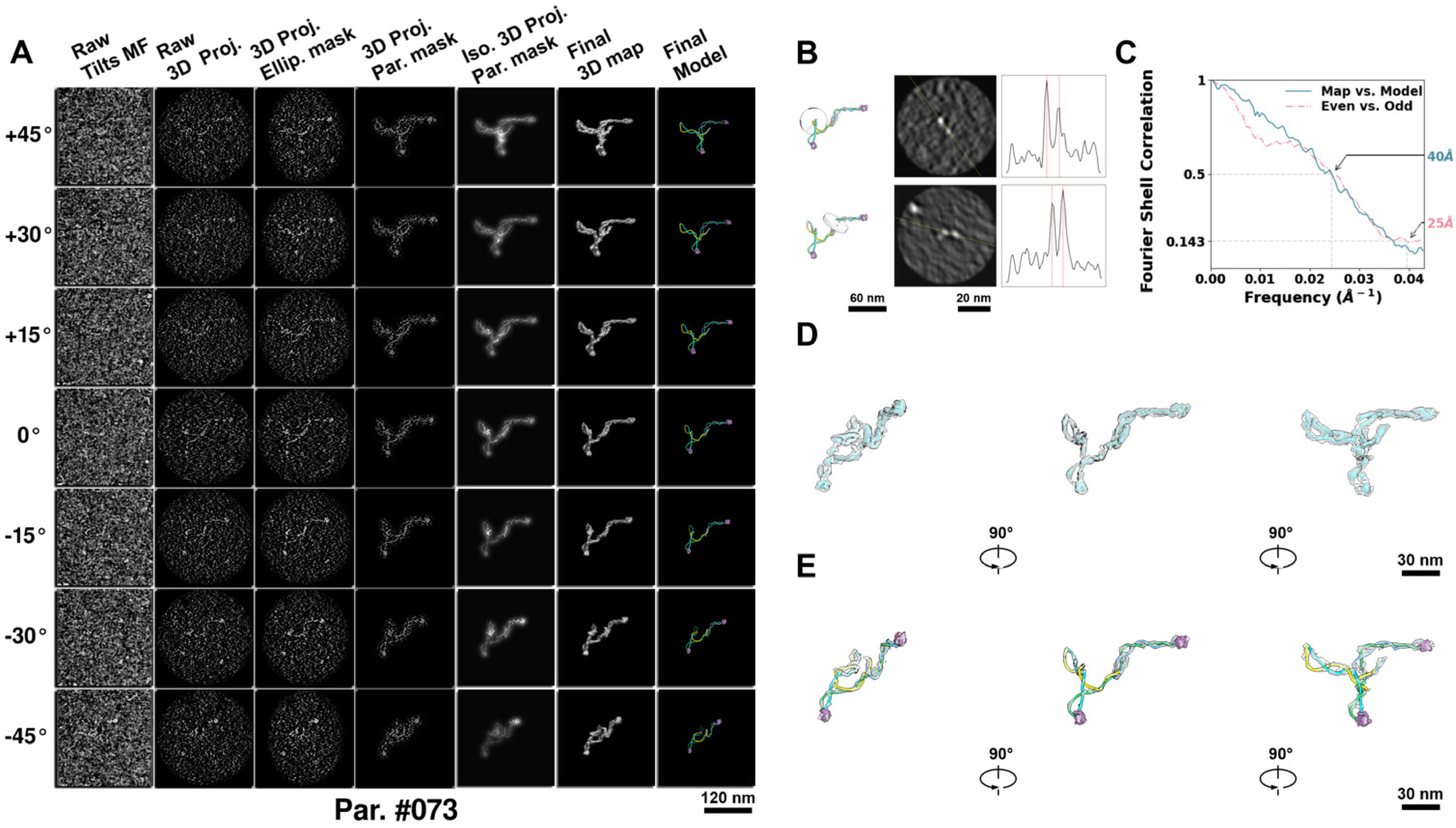

Supplementary Figure 73. Cryo-electron tomography (cryo-ET) 3D reconstruction of an individual plasmid particle in 40 mM K⁺, 5 mM Mg²⁺ in the presence of stalled RNAP.
(A) 3D reconstruction of the plasmid particle (index no. 73). The first column shows seven representative tilt images from –45° to +45° in step of 15°. The second, third, and fourth columns show 3D projections of the particle with spherical, ellipsoidal (thinner along the z-dimension), and particle-shaped masks, respectively. The fifth column displays the 3D projections of the enhanced and IsoNet missing-wedge-corrected particle. The sixth and seventh columns present the final 3D map and the flexibly fitted model, respectively. (B) Two random cross-sectional views (12 nm thickness) of the plasmid density map along its plectoneme axis are shown in the left-middle panel. The intensity profile along the line crossing the two high-density DNA spots is displayed in the right panel. (C) Resolution assessment of the final 3D map using Fourier shell correlation (FSC). Two criteria are shown: FSC between two half-maps reconstructed from even and odd frames (evaluated at 0.143) and FSC between the final 3D map and the fitted model (evaluated at 0.5). (D) Zoomed-in views of the final 3D density map from panel A, displayed at two contour levels. (E) Superimposition of the high-contour level map from panel D onto its flexibly fitted model.

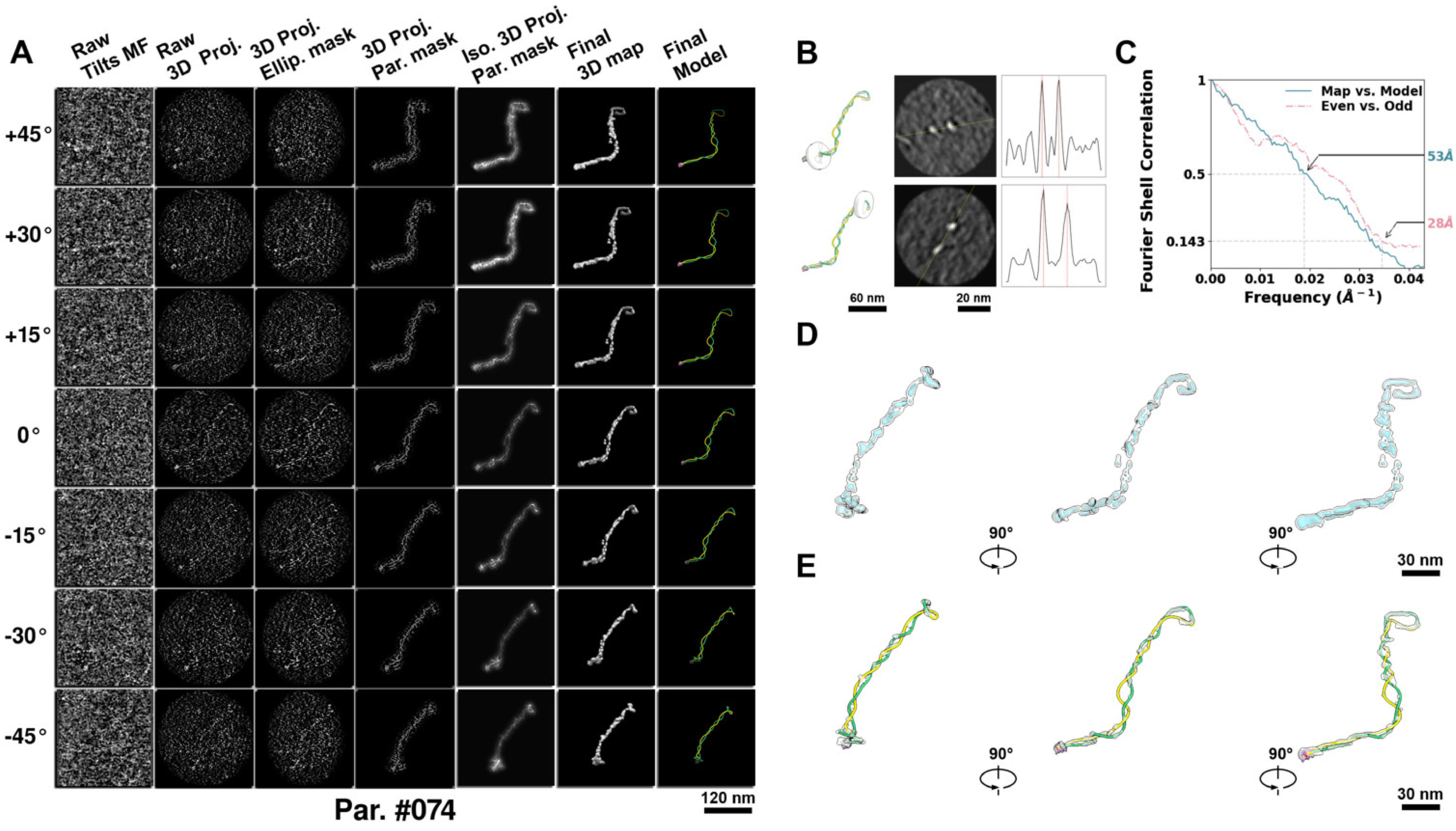

Supplementary Figure 74. Cryo-electron tomography (cryo-ET) 3D reconstruction of an individual plasmid particle in 40 mM K⁺, 5 mM Mg²⁺ in the presence of stalled RNAP.
(A) 3D reconstruction of the plasmid particle (index no. 74). The first column shows seven representative tilt images from –45° to +45° in step of 15°. The second, third, and fourth columns show 3D projections of the particle with spherical, ellipsoidal (thinner along the z-dimension), and particle-shaped masks, respectively. The fifth column displays the 3D projections of the enhanced and IsoNet missing-wedge-corrected particle. The sixth and seventh columns present the final 3D map and the flexibly fitted model, respectively. (B) Two random cross-sectional views (12 nm thickness) of the plasmid density map along its plectoneme axis are shown in the left-middle panel. The intensity profile along the line crossing the two high-density DNA spots is displayed in the right panel. (C) Resolution assessment of the final 3D map using Fourier shell correlation (FSC). Two criteria are shown: FSC between two half-maps reconstructed from even and odd frames (evaluated at 0.143) and FSC between the final 3D map and the fitted model (evaluated at 0.5). (D) Zoomed-in views of the final 3D density map from panel A, displayed at two contour levels. (E) Superimposition of the high-contour level map from panel D onto its flexibly fitted model.

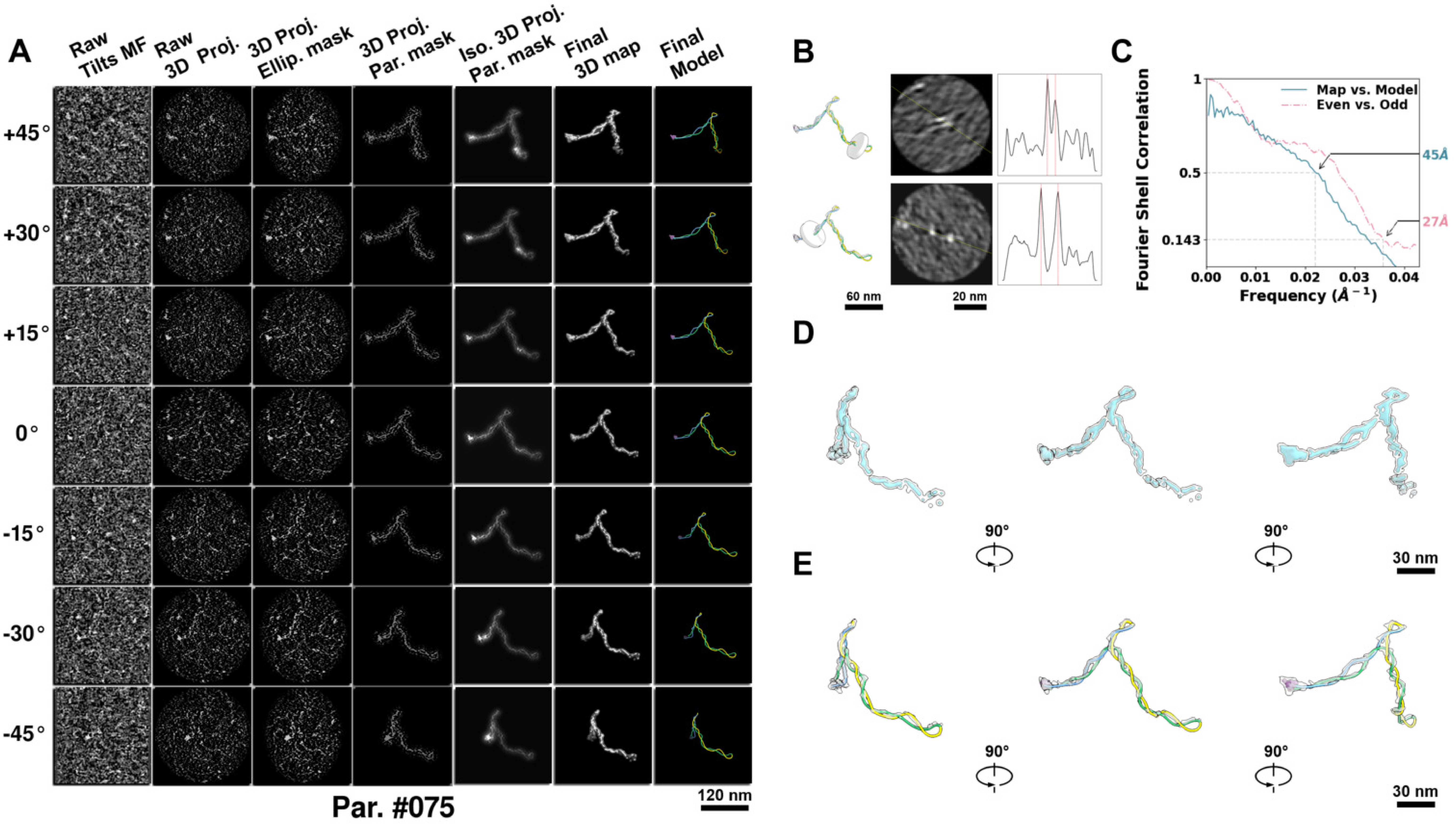

Supplementary Figure 75. Cryo-electron tomography (cryo-ET) 3D reconstruction of an individual plasmid particle in 40 mM K^+, 5 mM Mg^{2+} in the presence of stalled RNAP.
(A) 3D reconstruction of the plasmid particle (index no. 75). The first column shows seven representative tilt images from –45° to +45° in step of 15°. The second, third, and fourth columns show 3D projections of the particle with spherical, ellipsoidal (thinner along the z-dimension), and particle-shaped masks, respectively. The fifth column displays the 3D projections of the enhanced and IsoNet missing-wedge-corrected particle. The sixth and seventh columns present the final 3D map and the flexibly fitted model, respectively. (B) Two random cross-sectional views (12 nm thickness) of the plasmid density map along its plectoneme axis are shown in the left-middle panel. The intensity profile along the line crossing the two high-density DNA spots is displayed in the right panel. (C) Resolution assessment of the final 3D map using Fourier shell correlation (FSC). Two criteria are shown: FSC between two half-maps reconstructed from even and odd frames (evaluated at 0.143) and FSC between the final 3D map and the fitted model (evaluated at 0.5). (D) Zoomed-in views of the final 3D density map from panel A, displayed at two contour levels. (E) Superimposition of the high-contour level map from panel D onto its flexibly fitted model.

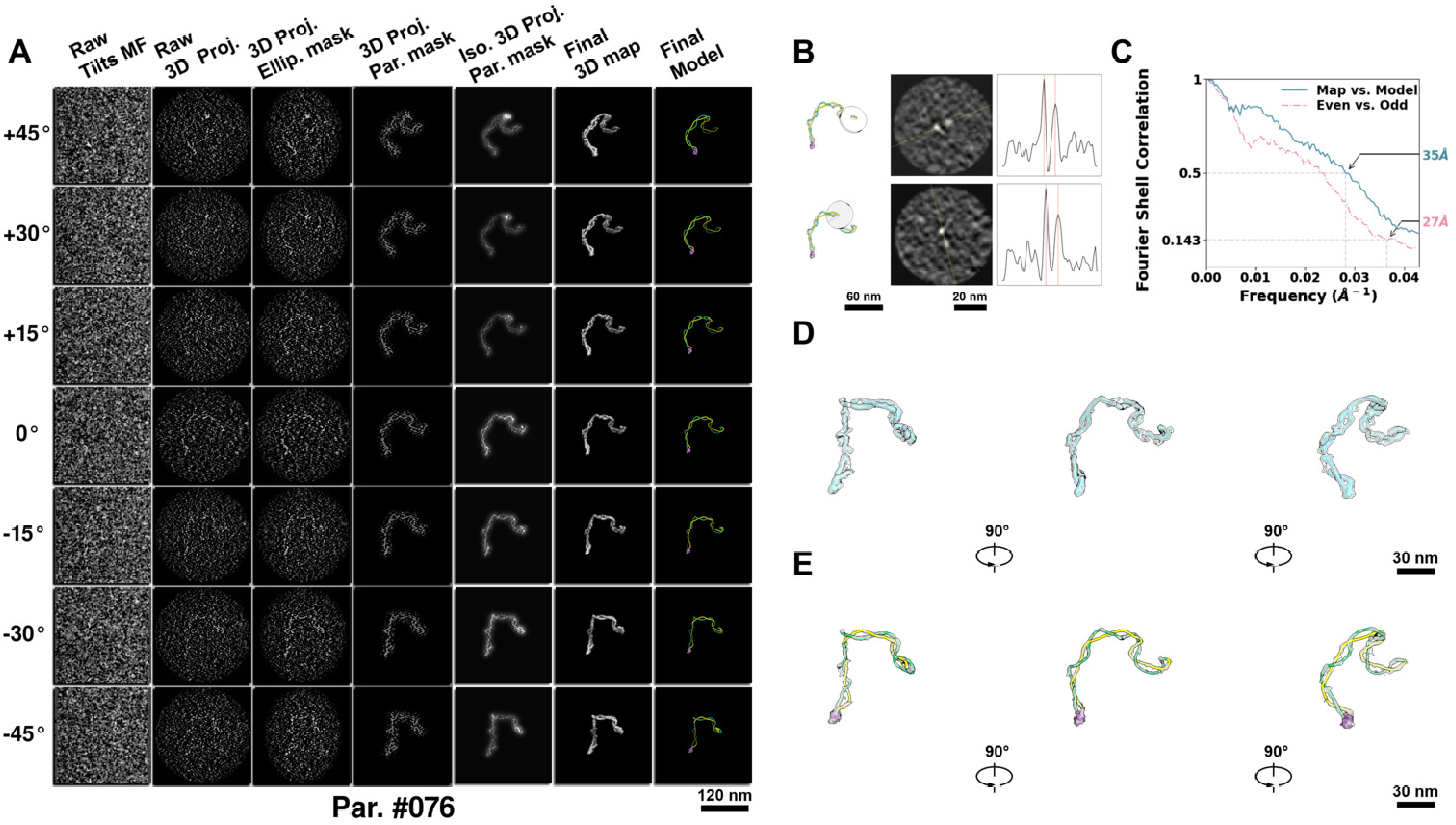

Supplementary Figure 76. Cryo-electron tomography (cryo-ET) 3D reconstruction of an individual plasmid particle in 40 mM K^+, 5 mM Mg^{2+} in the presence of stalled RNAP.
(A) 3D reconstruction of the plasmid particle (index no. 76). The first column shows seven representative tilt images from –45° to +45° in step of 15°. The second, third, and fourth columns show 3D projections of the particle with spherical, ellipsoidal (thinner along the z-dimension), and particle-shaped masks, respectively. The fifth column displays the 3D projections of the enhanced and IsoNet missing-wedge-corrected particle. The sixth and seventh columns present the final 3D map and the flexibly fitted model, respectively. (B) Two random cross-sectional views (12 nm thickness) of the plasmid density map along its plectoneme axis are shown in the left-middle panel. The intensity profile along the line crossing the two high-density DNA spots is displayed in the right panel. (C) Resolution assessment of the final 3D map using Fourier shell correlation (FSC). Two criteria are shown: FSC between two half-maps reconstructed from even and odd frames (evaluated at 0.143) and FSC between the final 3D map and the fitted model (evaluated at 0.5). (D) Zoomed-in views of the final 3D density map from panel A, displayed at two contour levels. (E) Superimposition of the high-contour level map from panel D onto its flexibly fitted model.

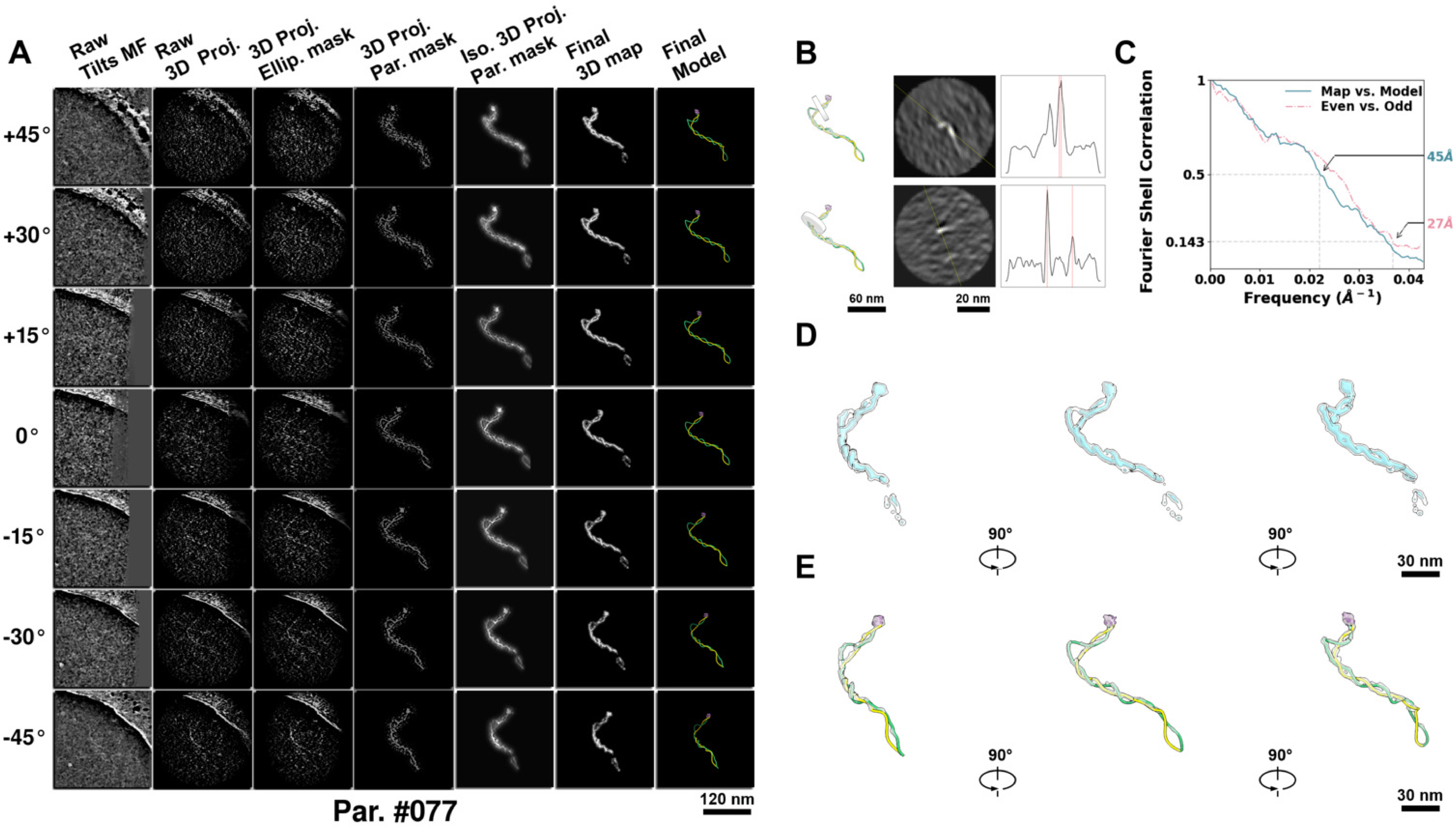

Supplementary Figure 77. Cryo-electron tomography (cryo-ET) 3D reconstruction of an individual plasmid particle in 40 mM K$^+$, 5 mM Mg^{2+} in the presence of stalled RNAP.
(A) 3D reconstruction of the plasmid particle (index no. 77). The first column shows seven representative tilt images from –45° to +45° in step of 15°. The second, third, and fourth columns show 3D projections of the particle with spherical, ellipsoidal (thinner along the z-dimension), and particle-shaped masks, respectively. The fifth column displays the 3D projections of the enhanced and IsoNet missing-wedge-corrected particle. The sixth and seventh columns present the final 3D map and the flexibly fitted model, respectively. (B) Two random cross-sectional views (12 nm thickness) of the plasmid density map along its plectoneme axis are shown in the left-middle panel. The intensity profile along the line crossing the two high-density DNA spots is displayed in the right panel. (C) Resolution assessment of the final 3D map using Fourier shell correlation (FSC). Two criteria are shown: FSC between two half-maps reconstructed from even and odd frames (evaluated at 0.143) and FSC between the final 3D map and the fitted model (evaluated at 0.5). (D) Zoomed-in views of the final 3D density map from panel A, displayed at two contour levels. (E) Superimposition of the high-contour level map from panel D onto its flexibly fitted model.

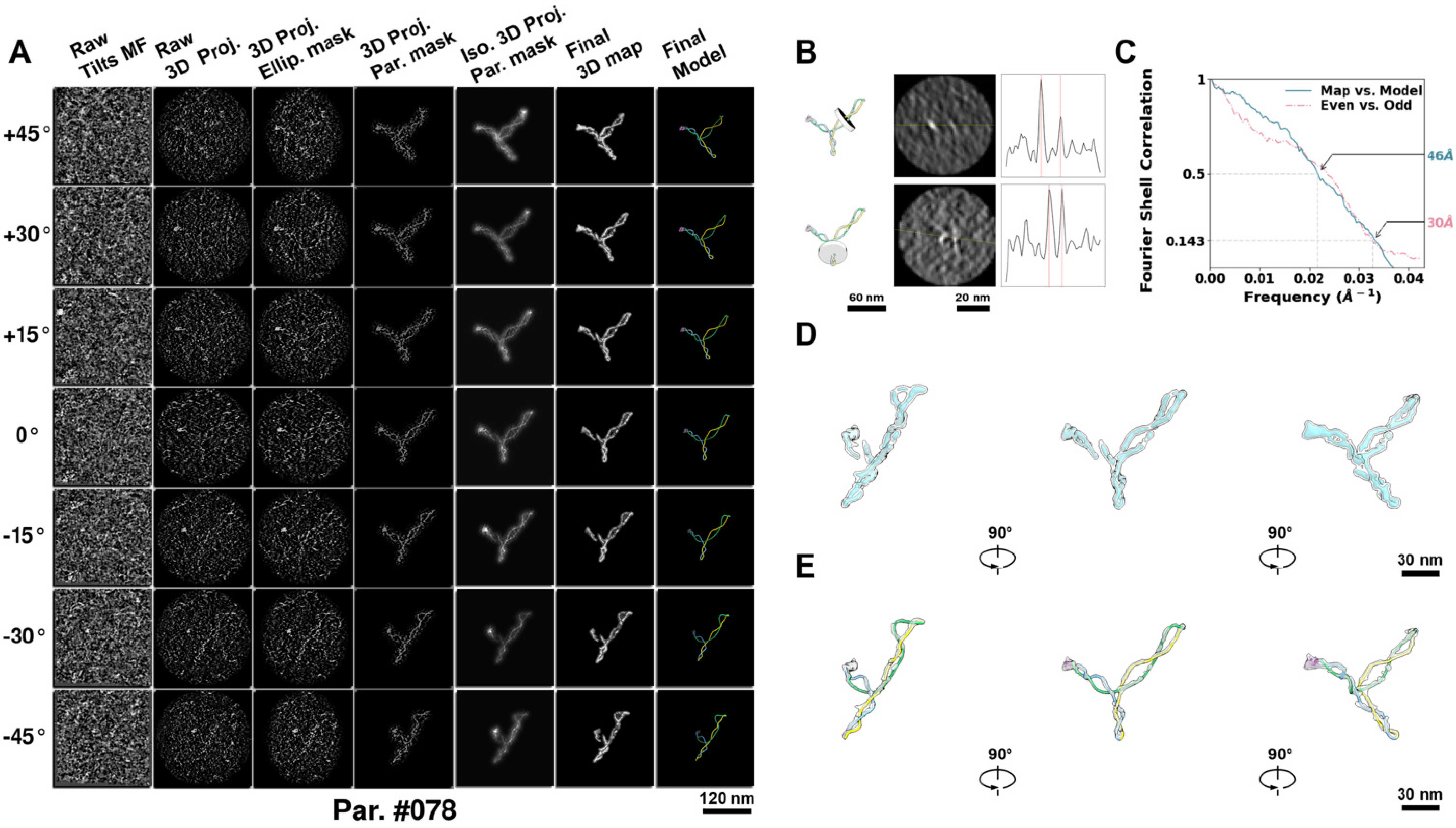

Supplementary Figure 78. Cryo-electron tomography (cryo-ET) 3D reconstruction of an individual plasmid particle in 40 mM K$^+$, 5 mM Mg^{2+} in the presence of stalled RNAP.
(A) 3D reconstruction of the plasmid particle (index no. 78). The first column shows seven representative tilt images from –45° to +45° in step of 15°. The second, third, and fourth columns show 3D projections of the particle with spherical, ellipsoidal (thinner along the z-dimension), and particle-shaped masks, respectively. The fifth column displays the 3D projections of the enhanced and IsoNet missing-wedge-corrected particle. The sixth and seventh columns present the final 3D map and the flexibly fitted model, respectively. (B) Two random cross-sectional views (12 nm thickness) of the plasmid density map along its plectoneme axis are shown in the left-middle panel. The intensity profile along the line crossing the two high-density DNA spots is displayed in the right panel. (C) Resolution assessment of the final 3D map using Fourier shell correlation (FSC). Two criteria are shown: FSC between two half-maps reconstructed from even and odd frames (evaluated at 0.143) and FSC between the final 3D map and the fitted model (evaluated at 0.5). (D) Zoomed-in views of the final 3D density map from panel A, displayed at two contour levels. (E) Superimposition of the high-contour level map from panel D onto its flexibly fitted model.

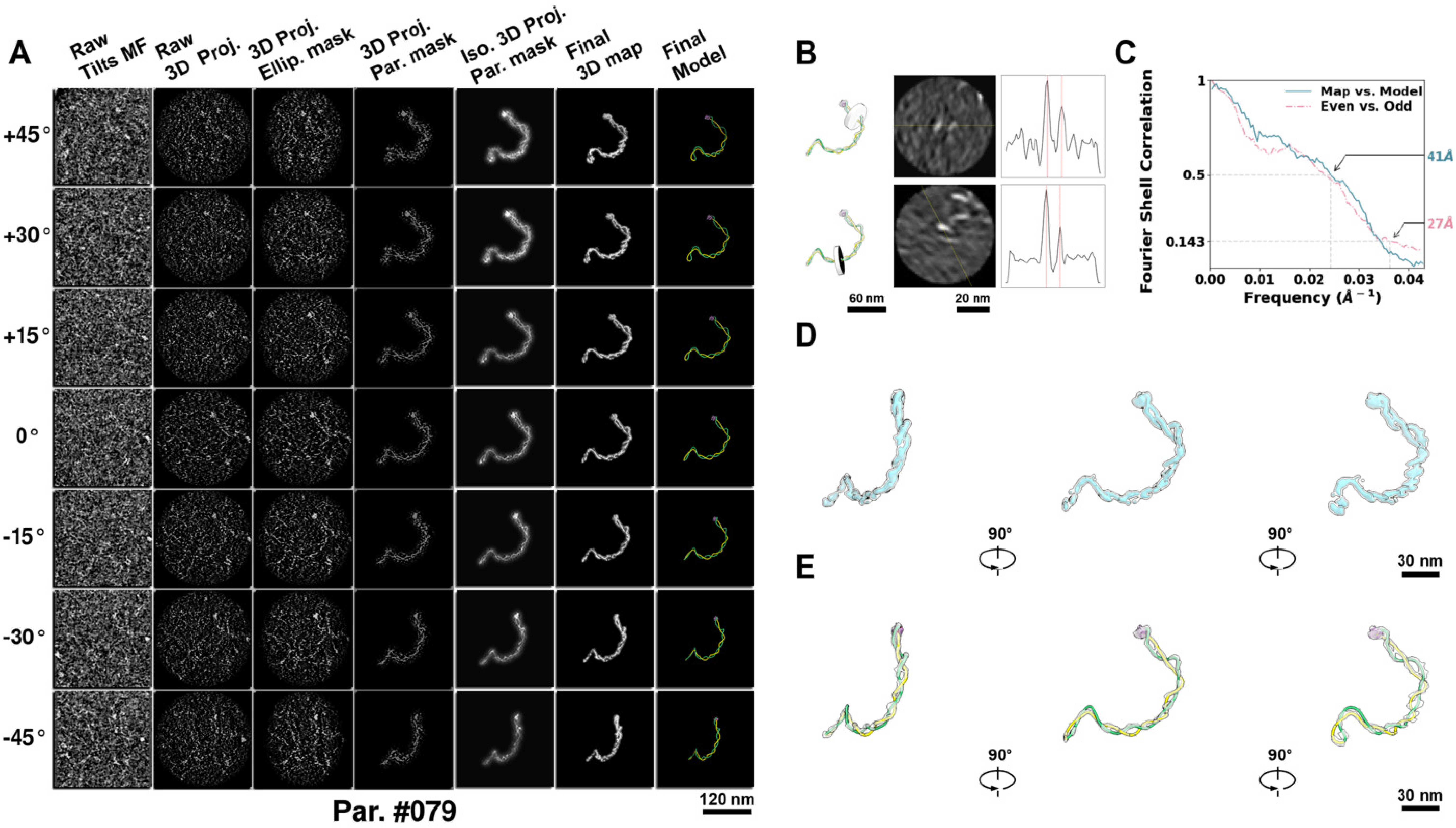

Supplementary Figure 79. Cryo-electron tomography (cryo-ET) 3D reconstruction of an individual plasmid particle in 40 mM K⁺, 5 mM Mg^{2+} in the presence of stalled RNAP.
(A) 3D reconstruction of the plasmid particle (index no. 79). The first column shows seven representative tilt images from –45° to +45° in step of 15°. The second, third, and fourth columns show 3D projections of the particle with spherical, ellipsoidal (thinner along the z-dimension), and particle-shaped masks, respectively. The fifth column displays the 3D projections of the enhanced and IsoNet missing-wedge-corrected particle. The sixth and seventh columns present the final 3D map and the flexibly fitted model, respectively. (B) Two random cross-sectional views (12 nm thickness) of the plasmid density map along its plectoneme axis are shown in the left-middle panel. The intensity profile along the line crossing the two high-density DNA spots is displayed in the right panel. (C) Resolution assessment of the final 3D map using Fourier shell correlation (FSC). Two criteria are shown: FSC between two half-maps reconstructed from even and odd frames (evaluated at 0.143) and FSC between the final 3D map and the fitted model (evaluated at 0.5). (D) Zoomed-in views of the final 3D density map from panel A, displayed at two contour levels. (E) Superimposition of the high-contour level map from panel D onto its flexibly fitted model.

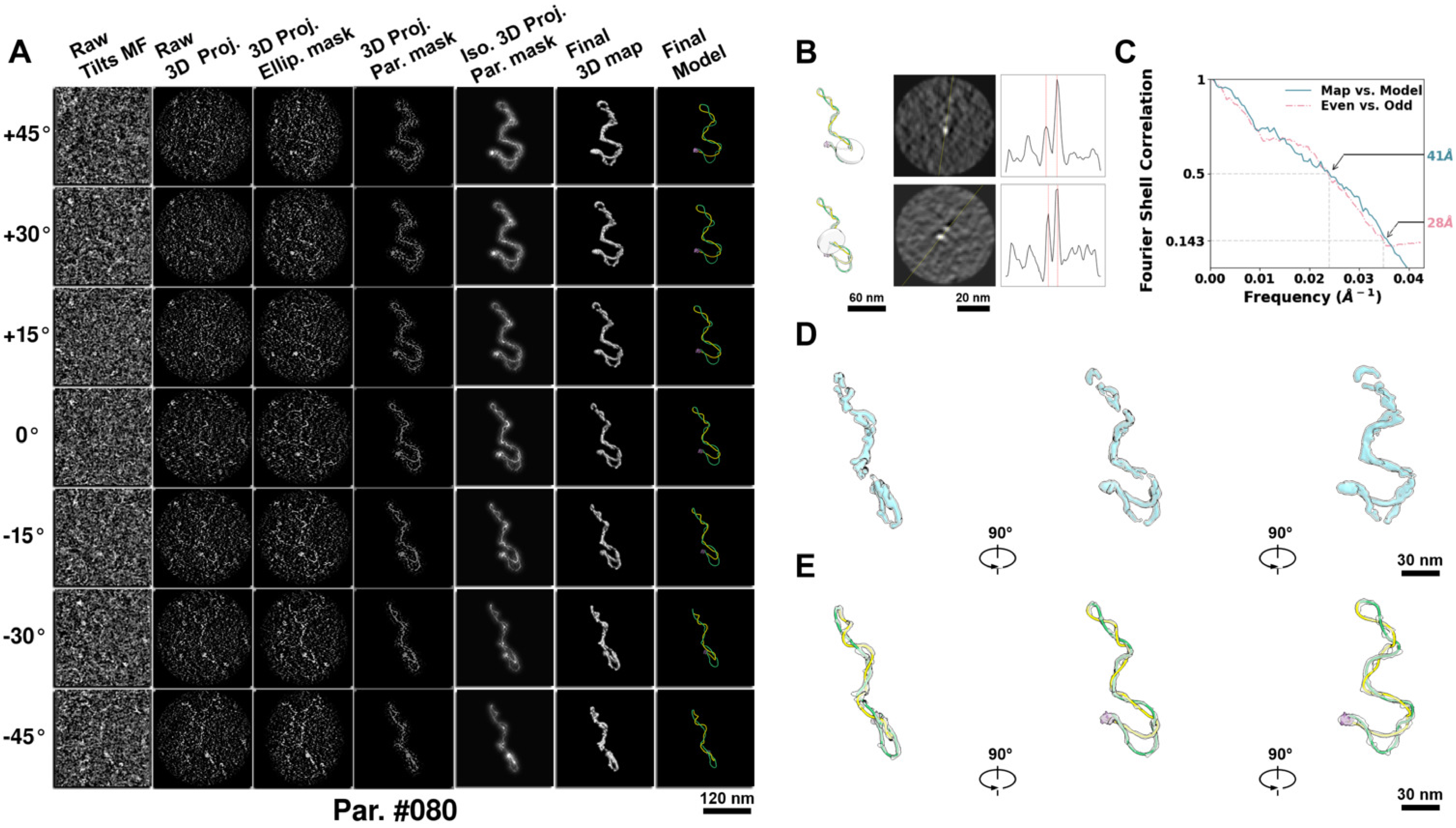

Supplementary Figure 80. Cryo-electron tomography (cryo-ET) 3D reconstruction of an individual plasmid particle in 40 mM K⁺, 5 mM Mg^{2+} in the presence of stalled RNAP.
(A) 3D reconstruction of the plasmid particle (index no. 80). The first column shows seven representative tilt images from –45° to +45° in step of 15°. The second, third, and fourth columns show 3D projections of the particle with spherical, ellipsoidal (thinner along the z-dimension), and particle-shaped masks, respectively. The fifth column displays the 3D projections of the enhanced and IsoNet missing-wedge-corrected particle. The sixth and seventh columns present the final 3D map and the flexibly fitted model, respectively. (B) Two random cross-sectional views (12 nm thickness) of the plasmid density map along its plectoneme axis are shown in the left-middle panel. The intensity profile along the line crossing the two high-density DNA spots is displayed in the right panel. (C) Resolution assessment of the final 3D map using Fourier shell correlation (FSC). Two criteria are shown: FSC between two half-maps reconstructed from even and odd frames (evaluated at 0.143) and FSC between the final 3D map and the fitted model (evaluated at 0.5). (D) Zoomed-in views of the final 3D density map from panel A, displayed at two contour levels. (E) Superimposition of the high-contour level map from panel D onto its flexibly fitted model.

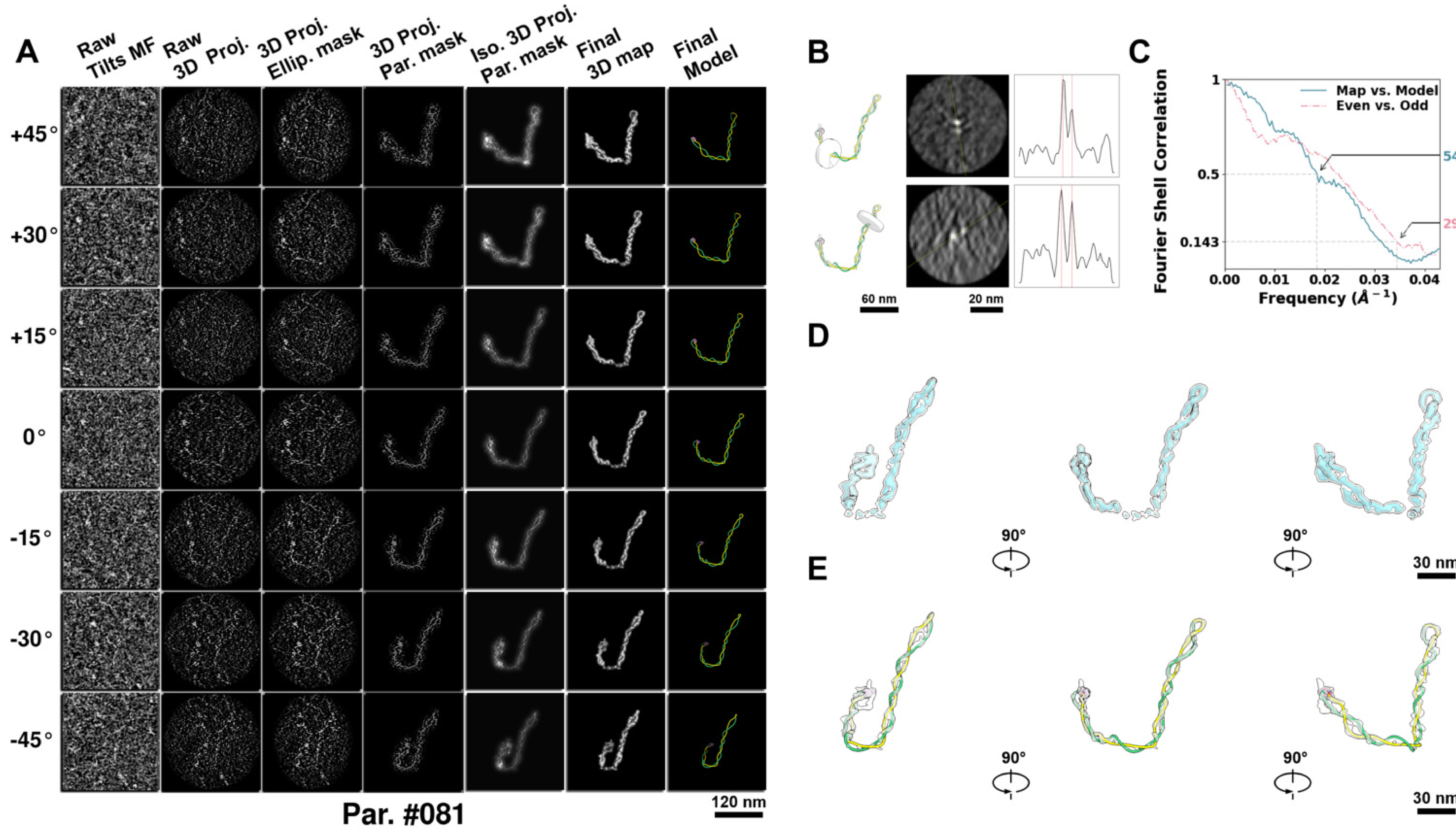

Supplementary Figure 81. Cryo-electron tomography (cryo-ET) 3D reconstruction of an individual plasmid particle in 40 mM K^+, 5 mM Mg^{2+} in the presence of stalled RNAP.
(A) 3D reconstruction of the plasmid particle (index no. 81). The first column shows seven representative tilt images from –45° to +45° in step of 15°. The second, third, and fourth columns show 3D projections of the particle with spherical, ellipsoidal (thinner along the z-dimension), and particle-shaped masks, respectively. The fifth column displays the 3D projections of the enhanced and IsoNet missing-wedge-corrected particle. The sixth and seventh columns present the final 3D map and the flexibly fitted model, respectively. (B) Two random cross-sectional views (12 nm thickness) of the plasmid density map along its plectoneme axis are shown in the left-middle panel. The intensity profile along the line crossing the two high-density DNA spots is displayed in the right panel. (C) Resolution assessment of the final 3D map using Fourier shell correlation (FSC). Two criteria are shown: FSC between two half-maps reconstructed from even and odd frames (evaluated at 0.143) and FSC between the final 3D map and the fitted model (evaluated at 0.5). (D) Zoomed-in views of the final 3D density map from panel A, displayed at two contour levels. (E) Superimposition of the high-contour level map from panel D onto its flexibly fitted model.

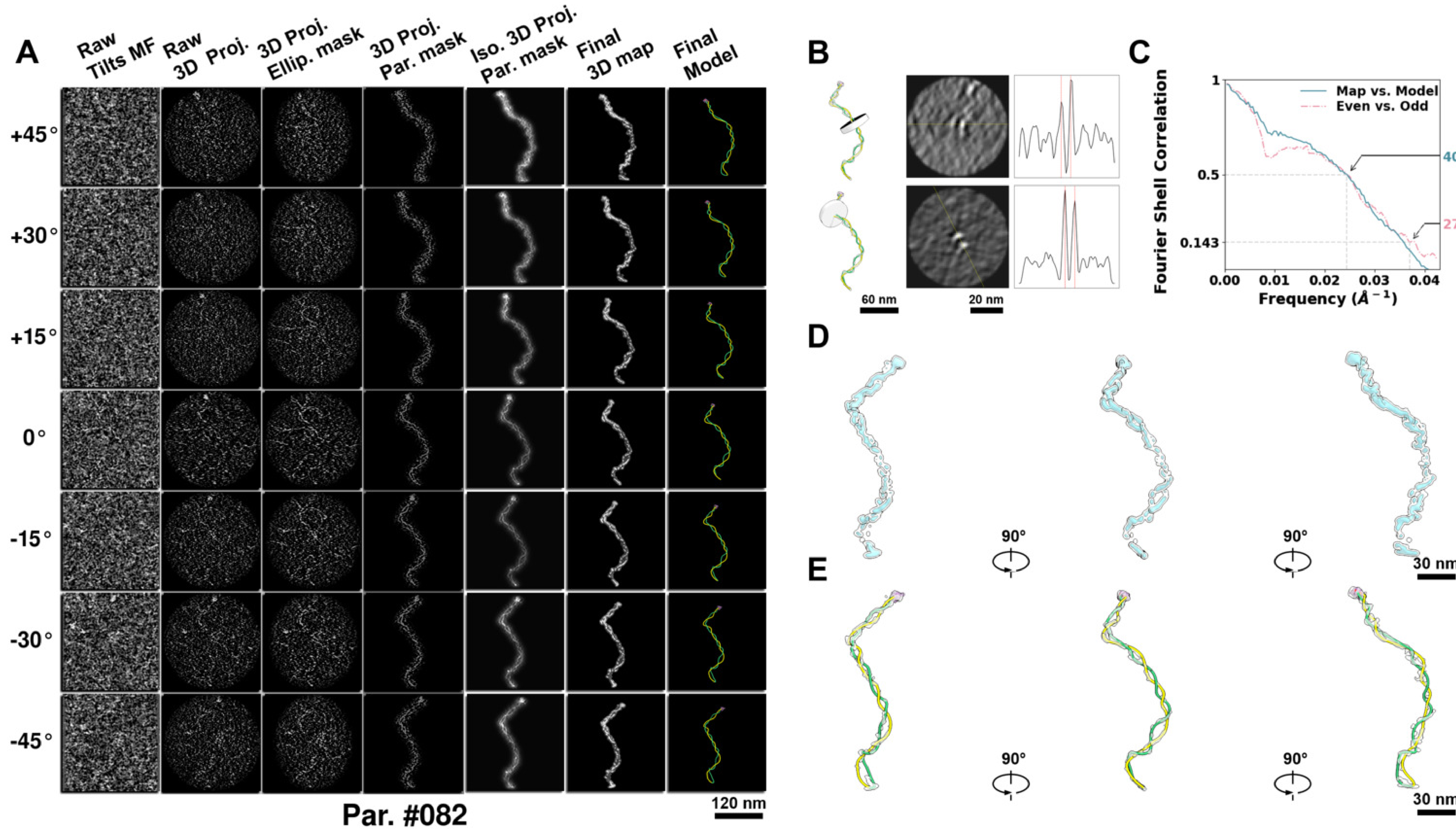

Supplementary Figure 82. Cryo-electron tomography (cryo-ET) 3D reconstruction of an individual plasmid particle in 40 mM K^+, 5 mM Mg^{2+} in the presence of stalled RNAP.
(A) 3D reconstruction of the plasmid particle (index no. 82). The first column shows seven representative tilt images from –45° to +45° in step of 15°. The second, third, and fourth columns show 3D projections of the particle with spherical, ellipsoidal (thinner along the z-dimension), and particle-shaped masks, respectively. The fifth column displays the 3D projections of the enhanced and IsoNet missing-wedge-corrected particle. The sixth and seventh columns present the final 3D map and the flexibly fitted model, respectively. (B) Two random cross-sectional views (12 nm thickness) of the plasmid density map along its plectoneme axis are shown in the left-middle panel. The intensity profile along the line crossing the two high-density DNA spots is displayed in the right panel. (C) Resolution assessment of the final 3D map using Fourier shell correlation (FSC). Two criteria are shown: FSC between two half-maps reconstructed from even and odd frames (evaluated at 0.143) and FSC between the final 3D map and the fitted model (evaluated at 0.5). (D) Zoomed-in views of the final 3D density map from panel A, displayed at two contour levels. (E) Superimposition of the high-contour level map from panel D onto its flexibly fitted model.

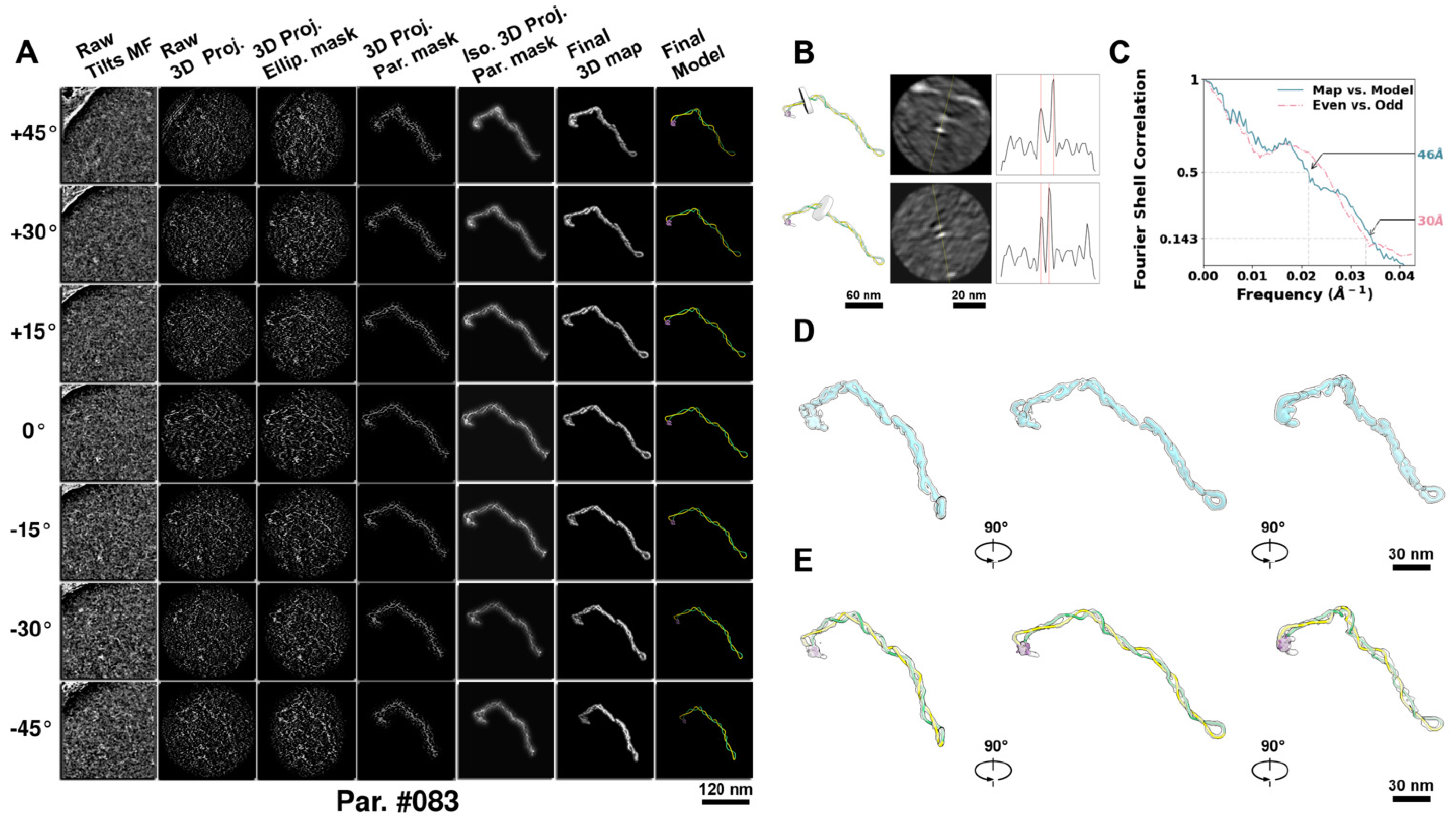

Supplementary Figure 83. Cryo-electron tomography (cryo-ET) 3D reconstruction of an individual plasmid particle in 40 mM K^+, 5 mM Mg^{2+} in the presence of stalled RNAP.
(A) 3D reconstruction of the plasmid particle (index no. 83). The first column shows seven representative tilt images from –45° to +45° in step of 15°. The second, third, and fourth columns show 3D projections of the particle with spherical, ellipsoidal (thinner along the z-dimension), and particle-shaped masks, respectively. The fifth column displays the 3D projections of the enhanced and IsoNet missing-wedge-corrected particle. The sixth and seventh columns present the final 3D map and the flexibly fitted model, respectively. (B) Two random cross-sectional views (12 nm thickness) of the plasmid density map along its plectoneme axis are shown in the left-middle panel. The intensity profile along the line crossing the two high-density DNA spots is displayed in the right panel. (C) Resolution assessment of the final 3D map using Fourier shell correlation (FSC). Two criteria are shown: FSC between two half-maps reconstructed from even and odd frames (evaluated at 0.143) and FSC between the final 3D map and the fitted model (evaluated at 0.5). (D) Zoomed-in views of the final 3D density map from panel A, displayed at two contour levels. (E) Superimposition of the high-contour level map from panel D onto its flexibly fitted model.

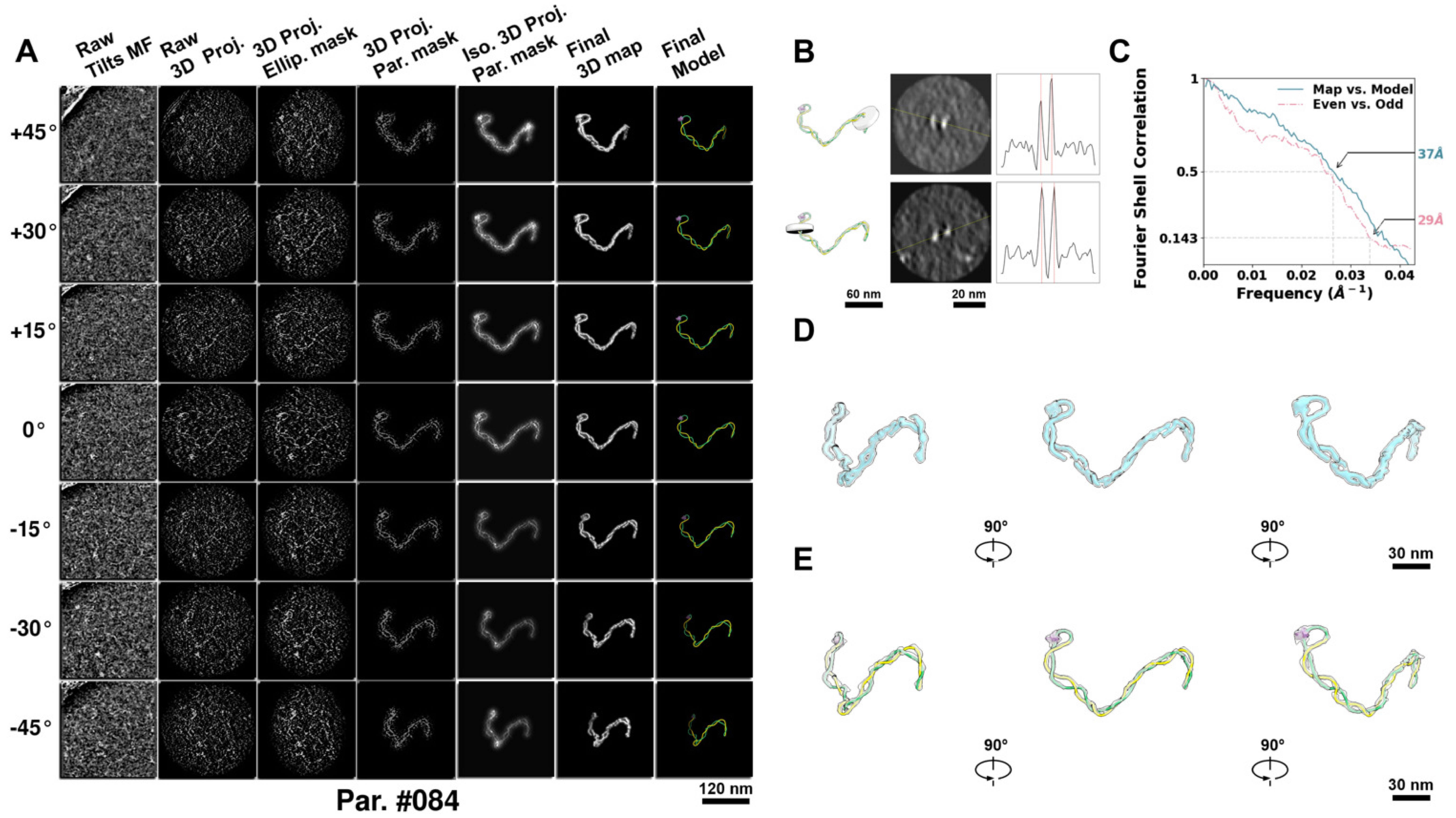

Supplementary Figure 84. Cryo-electron tomography (cryo-ET) 3D reconstruction of an individual plasmid particle in 40 mM K^+, 5 mM Mg^{2+} in the presence of stalled RNAP.
(A) 3D reconstruction of the plasmid particle (index no. 84). The first column shows seven representative tilt images from –45° to +45° in step of 15°. The second, third, and fourth columns show 3D projections of the particle with spherical, ellipsoidal (thinner along the z-dimension), and particle-shaped masks, respectively. The fifth column displays the 3D projections of the enhanced and IsoNet missing-wedge-corrected particle. The sixth and seventh columns present the final 3D map and the flexibly fitted model, respectively. (B) Two random cross-sectional views (12 nm thickness) of the plasmid density map along its plectoneme axis are shown in the left-middle panel. The intensity profile along the line crossing the two high-density DNA spots is displayed in the right panel. (C) Resolution assessment of the final 3D map using Fourier shell correlation (FSC). Two criteria are shown: FSC between two half-maps reconstructed from even and odd frames (evaluated at 0.143) and FSC between the final 3D map and the fitted model (evaluated at 0.5). (D) Zoomed-in views of the final 3D density map from panel A, displayed at two contour levels. (E) Superimposition of the high-contour level map from panel D onto its flexibly fitted model.

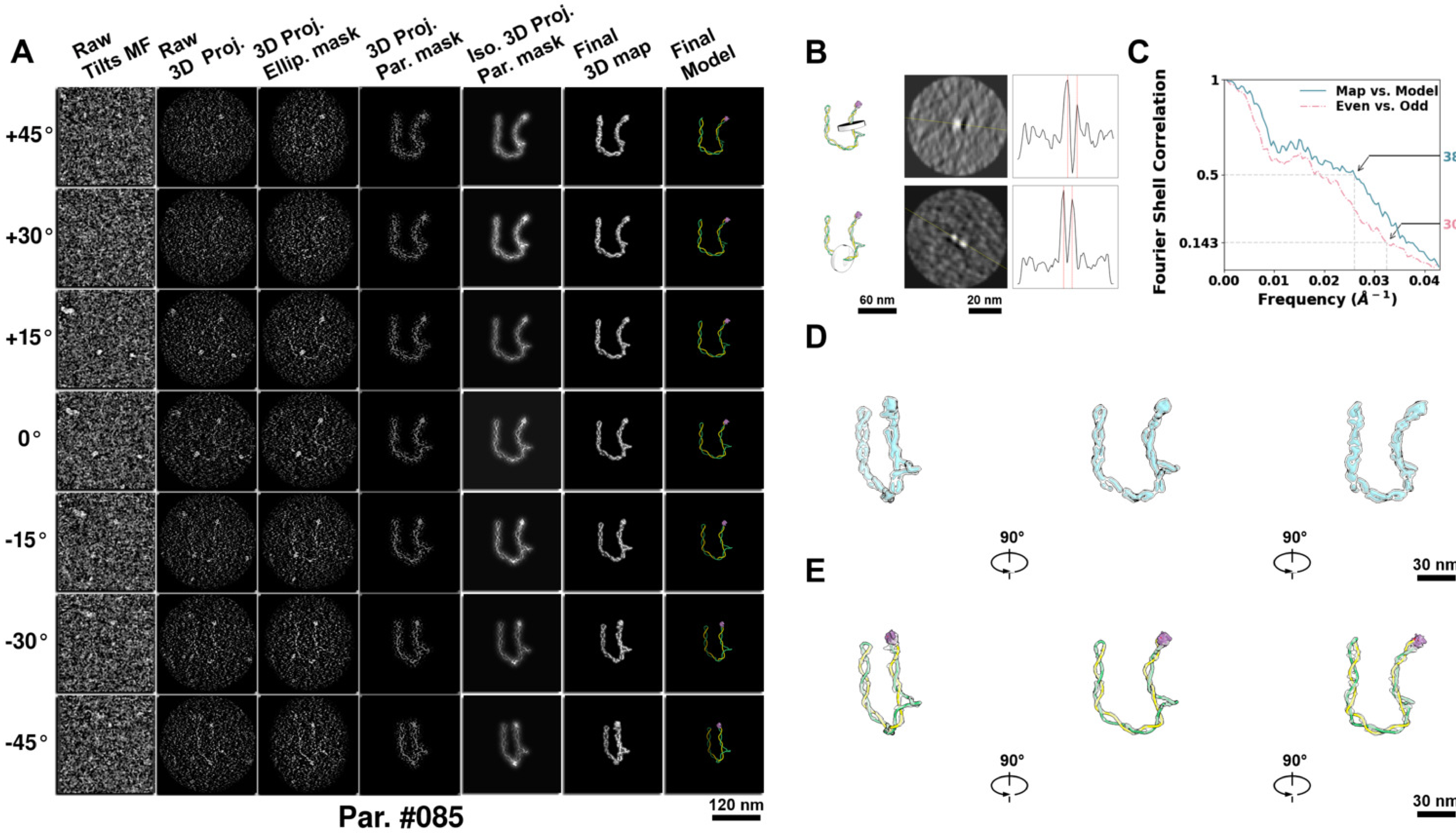

Supplementary Figure 85. Cryo-electron tomography (cryo-ET) 3D reconstruction of an individual plasmid particle in 40 mM K^+, 5 mM Mg^{2+} in the presence of stalled RNAP.
(A) 3D reconstruction of the plasmid particle (index no. 85). The first column shows seven representative tilt images from –45° to +45° in step of 15°. The second, third, and fourth columns show 3D projections of the particle with spherical, ellipsoidal (thinner along the z-dimension), and particle-shaped masks, respectively. The fifth column displays the 3D projections of the enhanced and IsoNet missing-wedge-corrected particle. The sixth and seventh columns present the final 3D map and the flexibly fitted model, respectively. (B) Two random cross-sectional views (12 nm thickness) of the plasmid density map along its plectoneme axis are shown in the left-middle panel. The intensity profile along the line crossing the two high-density DNA spots is displayed in the right panel. (C) Resolution assessment of the final 3D map using Fourier shell correlation (FSC). Two criteria are shown: FSC between two half-maps reconstructed from even and odd frames (evaluated at 0.143) and FSC between the final 3D map and the fitted model (evaluated at 0.5). (D) Zoomed-in views of the final 3D density map from panel A, displayed at two contour levels. (E) Superimposition of the high-contour level map from panel D onto its flexibly fitted model.

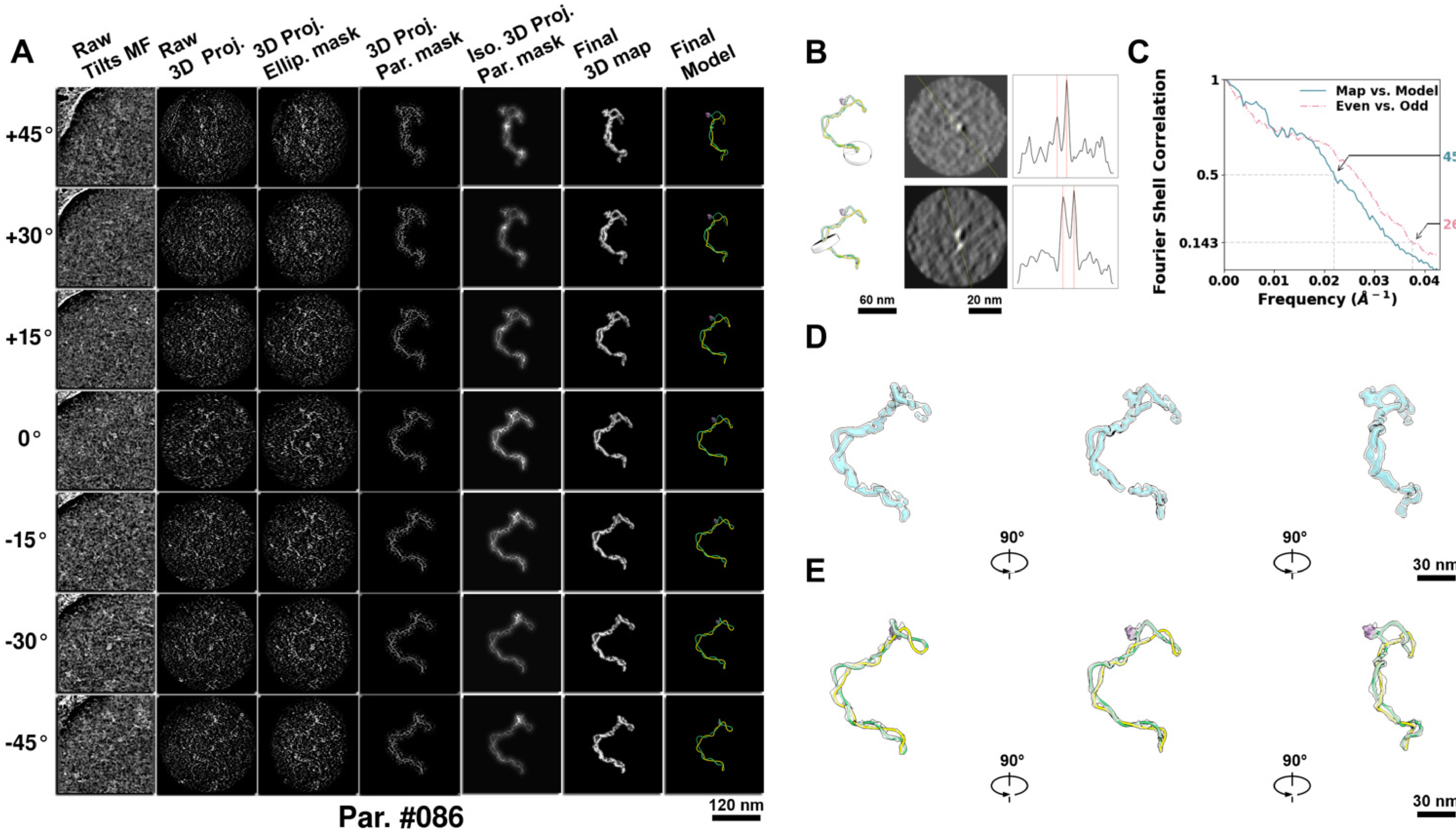

Supplementary Figure 86. Cryo-electron tomography (cryo-ET) 3D reconstruction of an individual plasmid particle in 40 mM K^+, 5 mM Mg^{2+} in the presence of stalled RNAP.
(A) 3D reconstruction of the plasmid particle (index no. 86). The first column shows seven representative tilt images from –45° to +45° in step of 15°. The second, third, and fourth columns show 3D projections of the particle with spherical, ellipsoidal (thinner along the z-dimension), and particle-shaped masks, respectively. The fifth column displays the 3D projections of the enhanced and IsoNet missing-wedge-corrected particle. The sixth and seventh columns present the final 3D map and the flexibly fitted model, respectively. (B) Two random cross-sectional views (12 nm thickness) of the plasmid density map along its plectoneme axis are shown in the left-middle panel. The intensity profile along the line crossing the two high-density DNA spots is displayed in the right panel. (C) Resolution assessment of the final 3D map using Fourier shell correlation (FSC). Two criteria are shown: FSC between two half-maps reconstructed from even and odd frames (evaluated at 0.143) and FSC between the final 3D map and the fitted model (evaluated at 0.5). (D) Zoomed-in views of the final 3D density map from panel A, displayed at two contour levels. (E) Superimposition of the high-contour level map from panel D onto its flexibly fitted model.

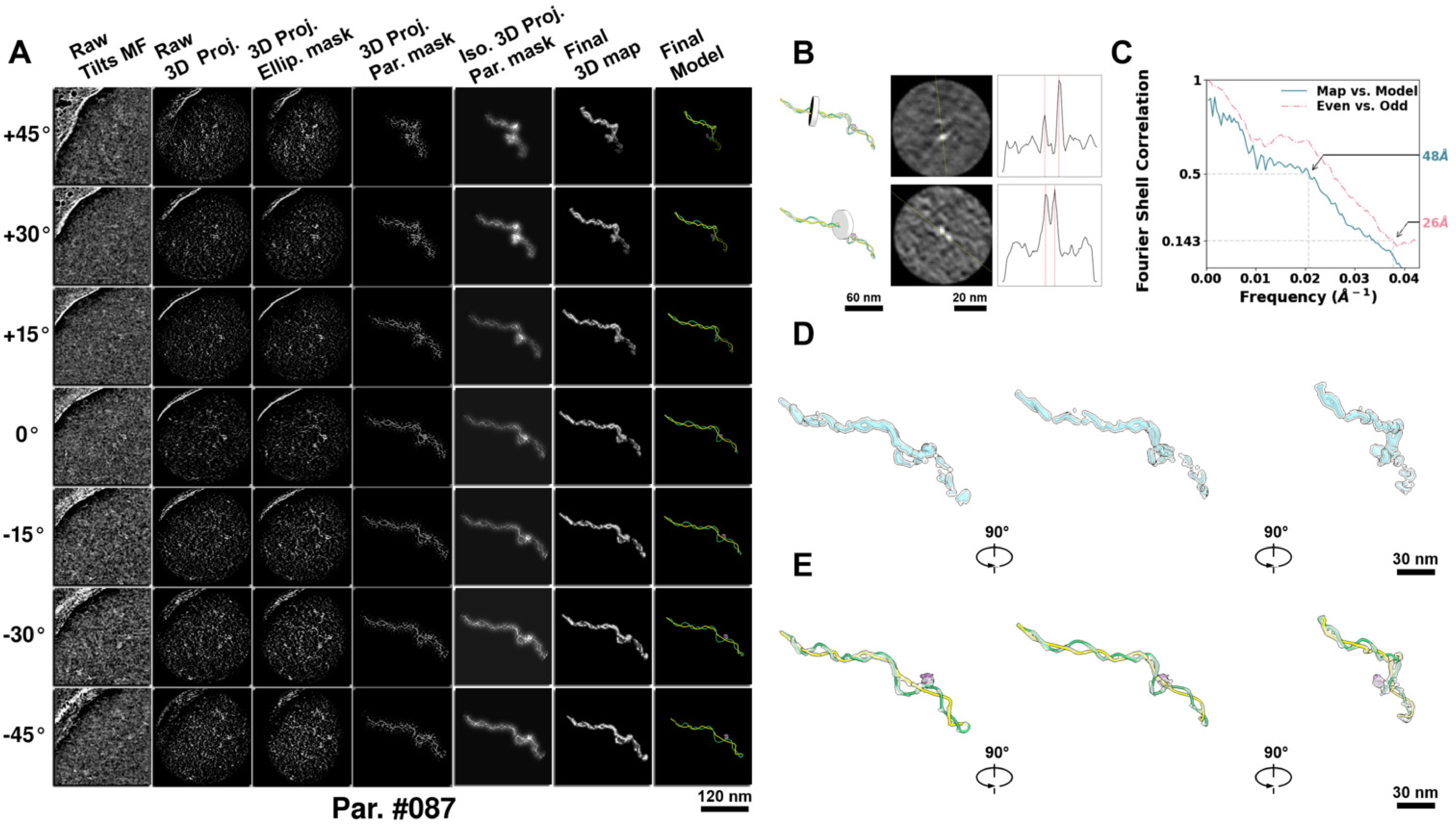

Supplementary Figure 87. Cryo-electron tomography (cryo-ET) 3D reconstruction of an individual plasmid particle in 40 mM K$^+$, 5 mM Mg^{2+} in the presence of stalled RNAP.
(A) 3D reconstruction of the plasmid particle (index no. 87). The first column shows seven representative tilt images from –45° to +45° in step of 15°. The second, third, and fourth columns show 3D projections of the particle with spherical, ellipsoidal (thinner along the z-dimension), and particle-shaped masks, respectively. The fifth column displays the 3D projections of the enhanced and IsoNet missing-wedge-corrected particle. The sixth and seventh columns present the final 3D map and the flexibly fitted model, respectively. (B) Two random cross-sectional views (12 nm thickness) of the plasmid density map along its plectoneme axis are shown in the left-middle panel. The intensity profile along the line crossing the two high-density DNA spots is displayed in the right panel. (C) Resolution assessment of the final 3D map using Fourier shell correlation (FSC). Two criteria are shown: FSC between two half-maps reconstructed from even and odd frames (evaluated at 0.143) and FSC between the final 3D map and the fitted model (evaluated at 0.5). (D) Zoomed-in views of the final 3D density map from panel A, displayed at two contour levels. (E) Superimposition of the high-contour level map from panel D onto its flexibly fitted model.

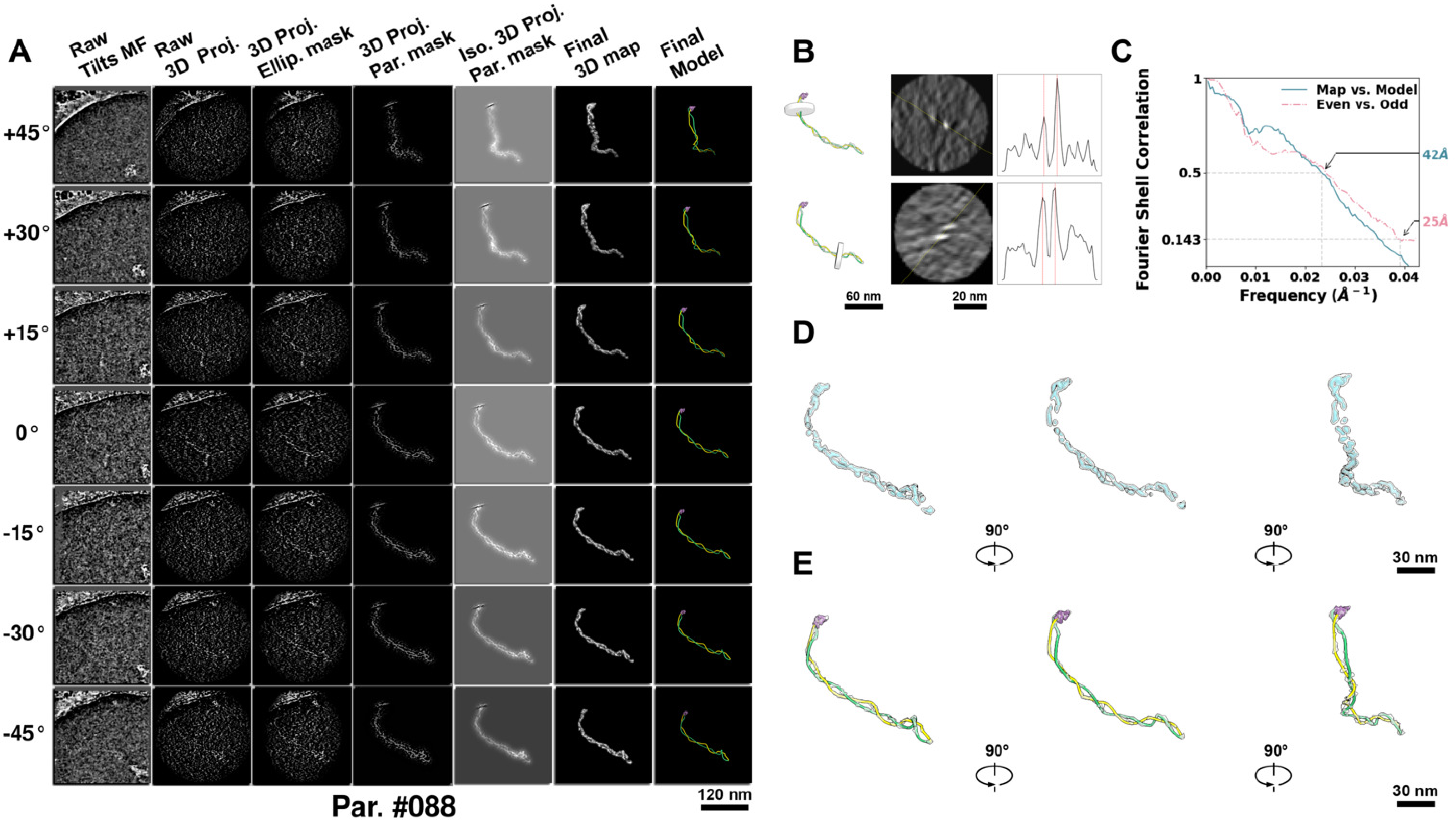

Supplementary Figure 88. Cryo-electron tomography (cryo-ET) 3D reconstruction of an individual plasmid particle in 40 mM K$^+$, 5 mM Mg^{2+} in the presence of stalled RNAP.
(A) 3D reconstruction of the plasmid particle (index no. 88). The first column shows seven representative tilt images from –45° to +45° in step of 15°. The second, third, and fourth columns show 3D projections of the particle with spherical, ellipsoidal (thinner along the z-dimension), and particle-shaped masks, respectively. The fifth column displays the 3D projections of the enhanced and IsoNet missing-wedge-corrected particle. The sixth and seventh columns present the final 3D map and the flexibly fitted model, respectively. (B) Two random cross-sectional views (12 nm thickness) of the plasmid density map along its plectoneme axis are shown in the left-middle panel. The intensity profile along the line crossing the two high-density DNA spots is displayed in the right panel. (C) Resolution assessment of the final 3D map using Fourier shell correlation (FSC). Two criteria are shown: FSC between two half-maps reconstructed from even and odd frames (evaluated at 0.143) and FSC between the final 3D map and the fitted model (evaluated at 0.5). (D) Zoomed-in views of the final 3D density map from panel A, displayed at two contour levels. (E) Superimposition of the high-contour level map from panel D onto its flexibly fitted model.

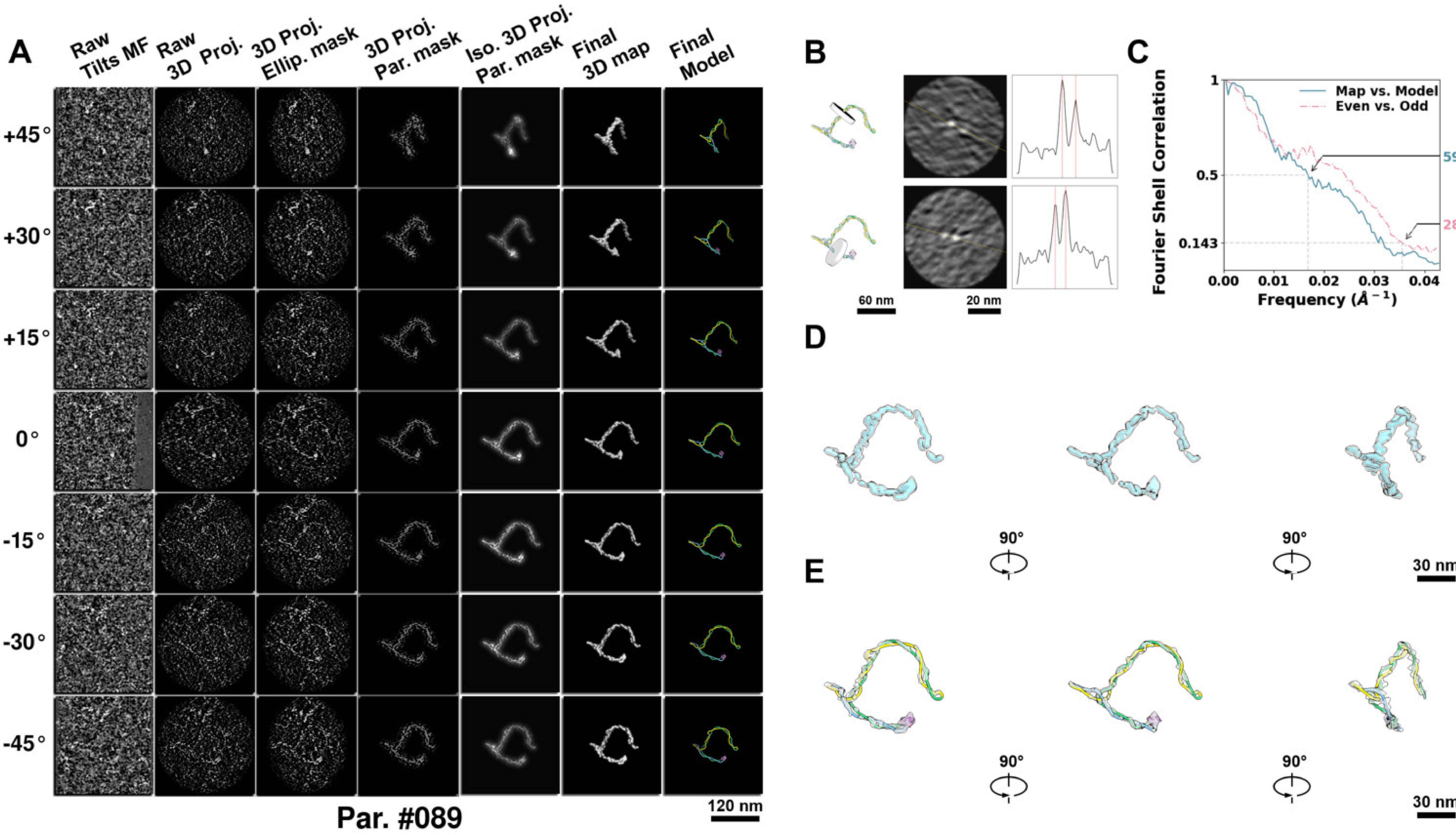

Supplementary Figure 89. Cryo-electron tomography (cryo-ET) 3D reconstruction of an individual plasmid particle in 40 mM K^+, 5 mM Mg^{2+} in the presence of stalled RNAP.
(A) 3D reconstruction of the plasmid particle (index no. 89). The first column shows seven representative tilt images from –45° to +45° in step of 15°. The second, third, and fourth columns show 3D projections of the particle with spherical, ellipsoidal (thinner along the z-dimension), and particle-shaped masks, respectively. The fifth column displays the 3D projections of the enhanced and IsoNet missing-wedge-corrected particle. The sixth and seventh columns present the final 3D map and the flexibly fitted model, respectively. (B) Two random cross-sectional views (12 nm thickness) of the plasmid density map along its plectoneme axis are shown in the left-middle panel. The intensity profile along the line crossing the two high-density DNA spots is displayed in the right panel. (C) Resolution assessment of the final 3D map using Fourier shell correlation (FSC). Two criteria are shown: FSC between two half-maps reconstructed from even and odd frames (evaluated at 0.143) and FSC between the final 3D map and the fitted model (evaluated at 0.5). (D) Zoomed-in views of the final 3D density map from panel A, displayed at two contour levels. (E) Superimposition of the high-contour level map from panel D onto its flexibly fitted model.

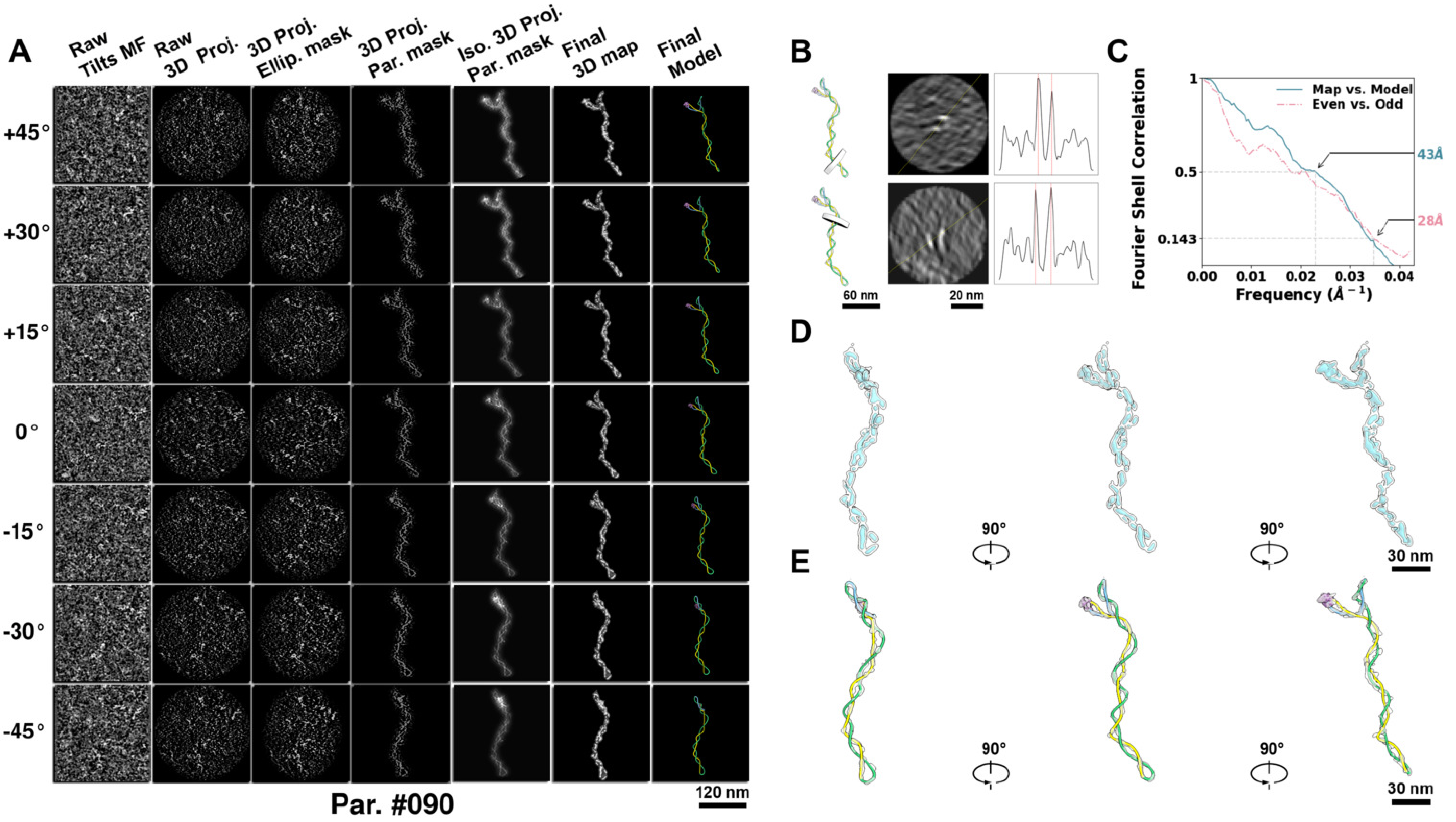

Supplementary Figure 90. Cryo-electron tomography (cryo-ET) 3D reconstruction of an individual plasmid particle in 40 mM K^+, 5 mM Mg^{2+} in the presence of stalled RNAP.
(A) 3D reconstruction of the plasmid particle (index no. 90). The first column shows seven representative tilt images from –45° to +45° in step of 15°. The second, third, and fourth columns show 3D projections of the particle with spherical, ellipsoidal (thinner along the z-dimension), and particle-shaped masks, respectively. The fifth column displays the 3D projections of the enhanced and IsoNet missing-wedge-corrected particle. The sixth and seventh columns present the final 3D map and the flexibly fitted model, respectively. (B) Two random cross-sectional views (12 nm thickness) of the plasmid density map along its plectoneme axis are shown in the left-middle panel. The intensity profile along the line crossing the two high-density DNA spots is displayed in the right panel. (C) Resolution assessment of the final 3D map using Fourier shell correlation (FSC). Two criteria are shown: FSC between two half-maps reconstructed from even and odd frames (evaluated at 0.143) and FSC between the final 3D map and the fitted model (evaluated at 0.5). (D) Zoomed-in views of the final 3D density map from panel A, displayed at two contour levels. (E) Superimposition of the high-contour level map from panel D onto its flexibly fitted model.

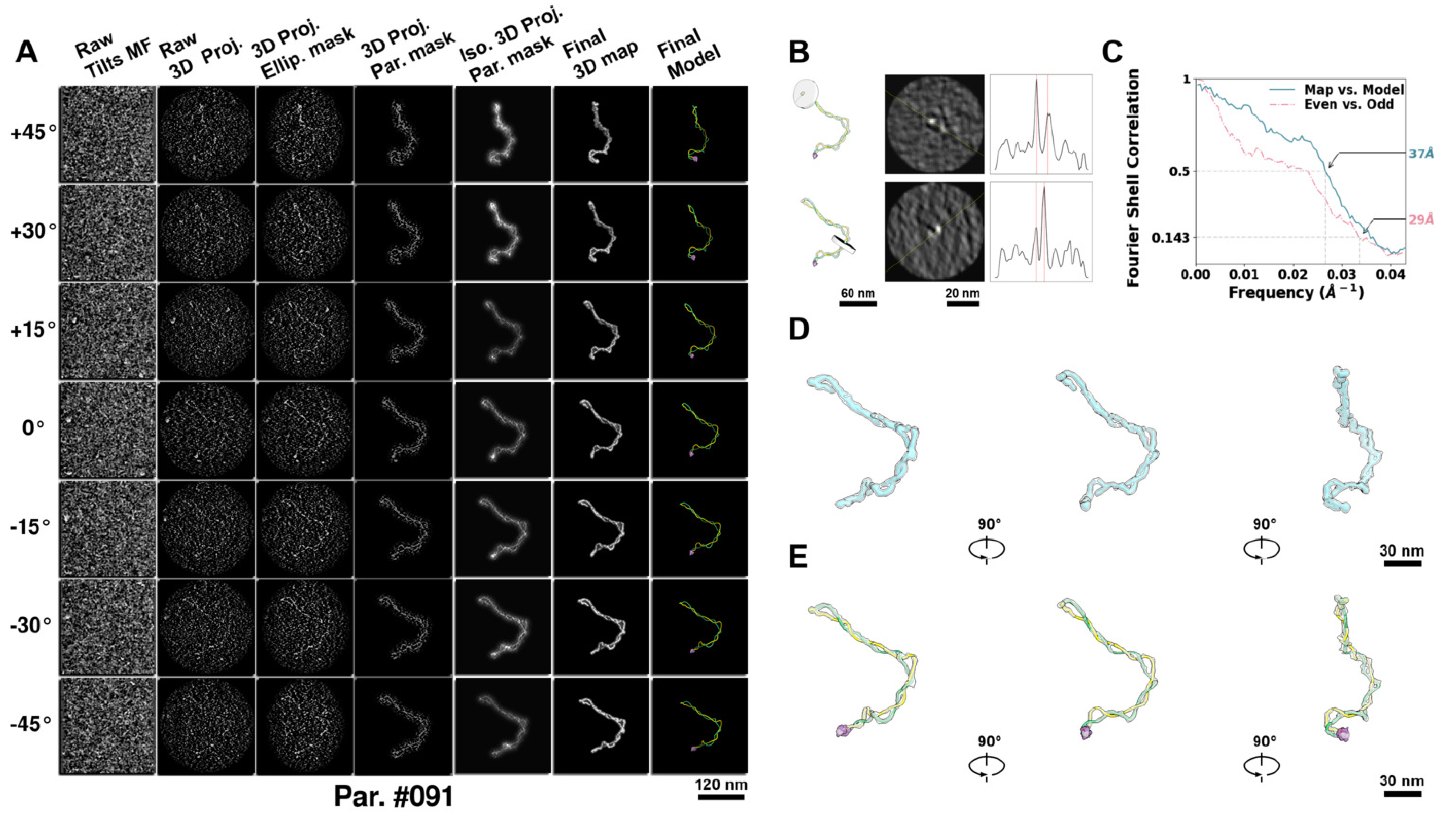

Supplementary Figure 91. Cryo-electron tomography (cryo-ET) 3D reconstruction of an individual plasmid particle in 40 mM K^+, 5 mM Mg^{2+} in the presence of stalled RNAP.
(A) 3D reconstruction of the plasmid particle (index no. 91). The first column shows seven representative tilt images from –45° to +45° in step of 15°. The second, third, and fourth columns show 3D projections of the particle with spherical, ellipsoidal (thinner along the z-dimension), and particle-shaped masks, respectively. The fifth column displays the 3D projections of the enhanced and IsoNet missing-wedge-corrected particle. The sixth and seventh columns present the final 3D map and the flexibly fitted model, respectively. (B) Two random cross-sectional views (12 nm thickness) of the plasmid density map along its plectoneme axis are shown in the left-middle panel. The intensity profile along the line crossing the two high-density DNA spots is displayed in the right panel. (C) Resolution assessment of the final 3D map using Fourier shell correlation (FSC). Two criteria are shown: FSC between two half-maps reconstructed from even and odd frames (evaluated at 0.143) and FSC between the final 3D map and the fitted model (evaluated at 0.5). (D) Zoomed-in views of the final 3D density map from panel A, displayed at two contour levels. (E) Superimposition of the high-contour level map from panel D onto its flexibly fitted model.

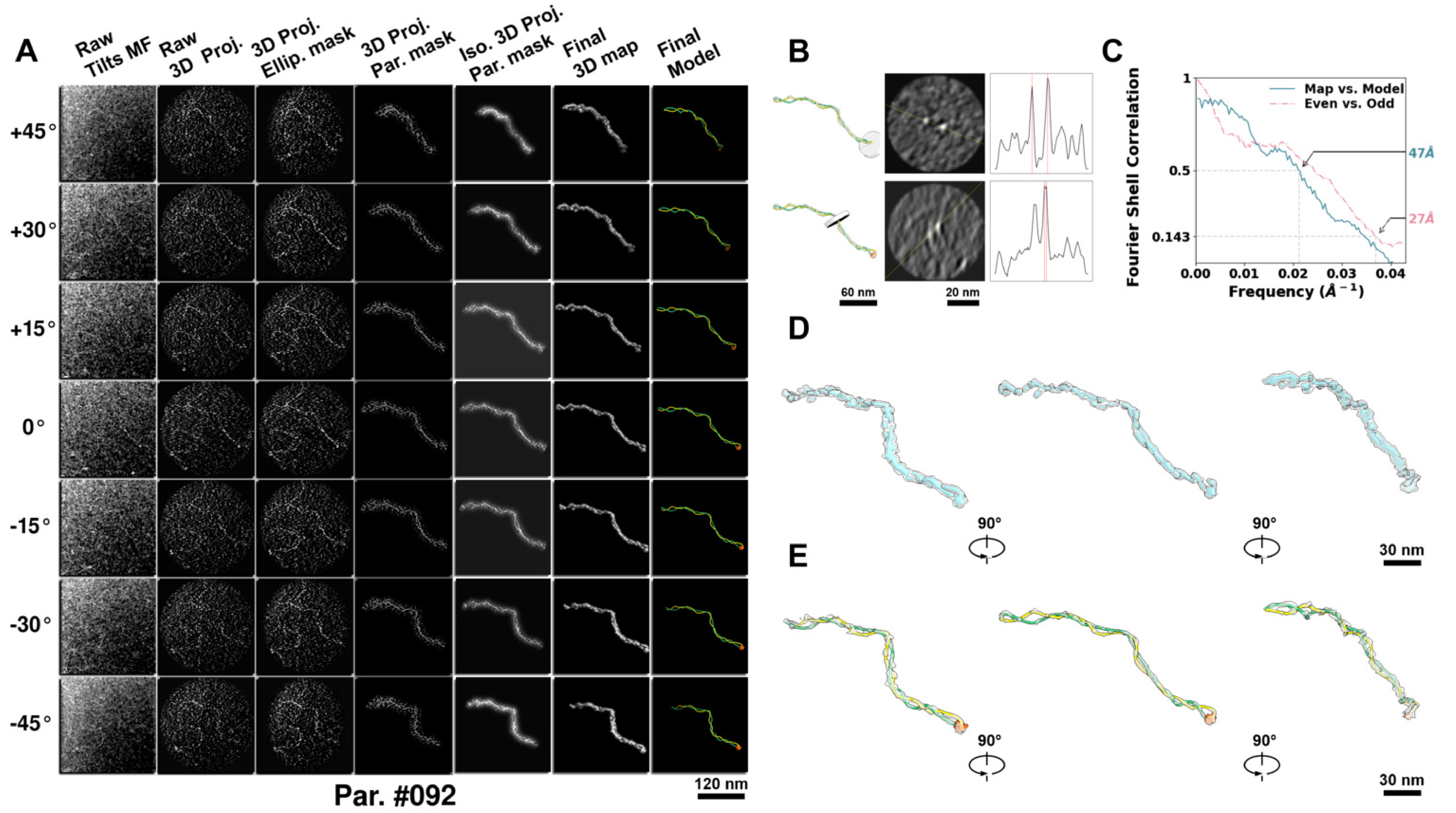

Supplementary Figure 92. Cryo-electron tomography (cryo-ET) 3D reconstruction of an individual plasmid particle in 40 mM K^+, 5 mM Mg^{2+} in the presence of dCas9.
(A) 3D reconstruction of the plasmid particle (index no. 92). The first column shows seven representative tilt images from –45° to +45° in step of 15°. The second, third, and fourth columns show 3D projections of the particle with spherical, ellipsoidal (thinner along the z-dimension), and particle-shaped masks, respectively. The fifth column displays the 3D projections of the enhanced and IsoNet missing-wedge-corrected particle. The sixth and seventh columns present the final 3D map and the flexibly fitted model, respectively. (B) Two random cross-sectional views (12 nm thickness) of the plasmid density map along its plectoneme axis are shown in the left-middle panel. The intensity profile along the line crossing the two high-density DNA spots is displayed in the right panel. (C) Resolution assessment of the final 3D map using Fourier shell correlation (FSC). Two criteria are shown: FSC between two half-maps reconstructed from even and odd frames (evaluated at 0.143) and FSC between the final 3D map and the fitted model (evaluated at 0.5). (D) Zoomed-in views of the final 3D density map from panel A, displayed at two contour levels. (E) Superimposition of the high-contour level map from panel D onto its flexibly fitted model.

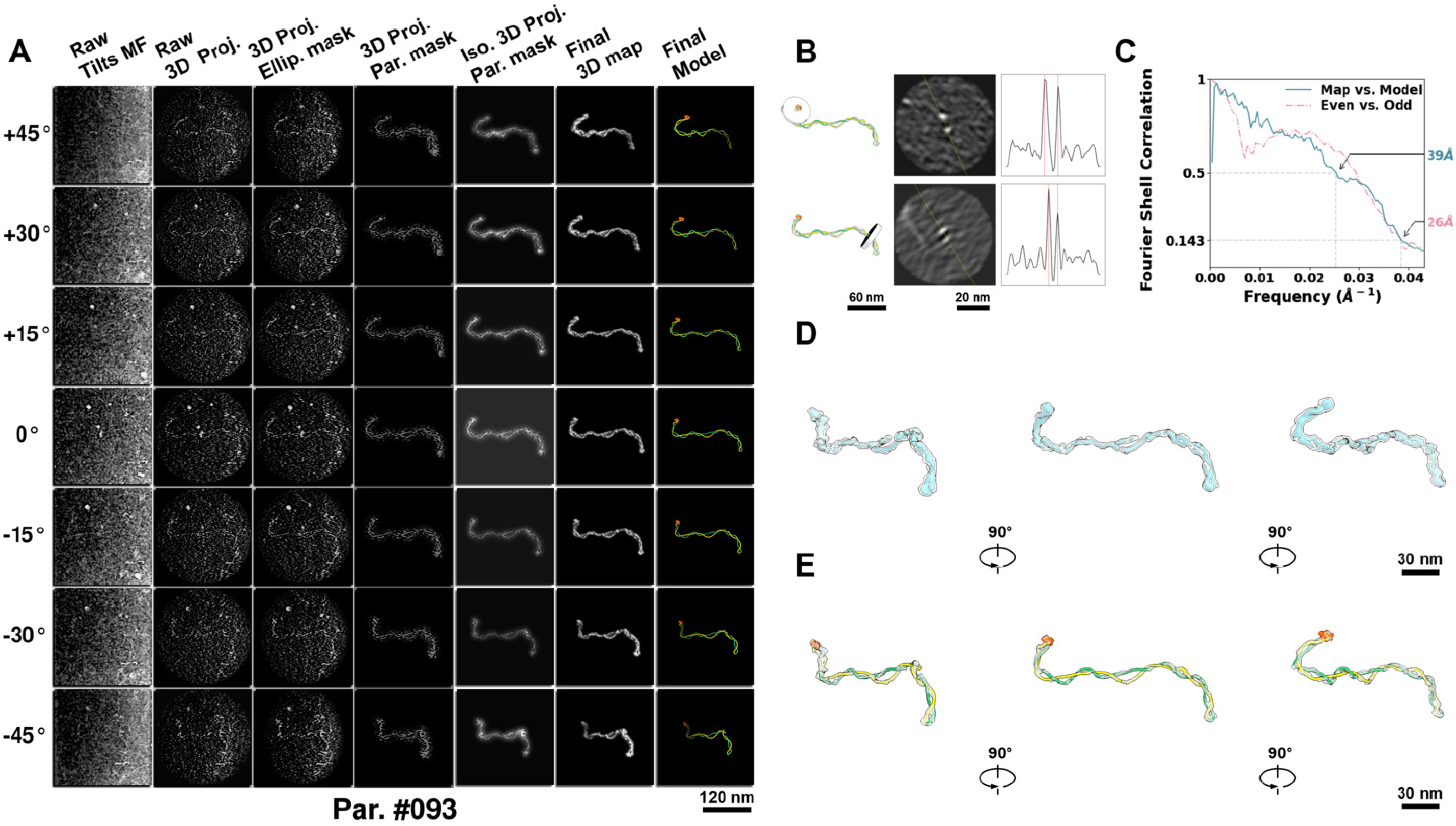

Supplementary Figure 93. Cryo-electron tomography (cryo-ET) 3D reconstruction of an individual plasmid particle in 40 mM K^+, 5 mM Mg^{2+} in the presence of dCas9.
(A) 3D reconstruction of the plasmid particle (index no. 93). The first column shows seven representative tilt images from –45° to +45° in step of 15°. The second, third, and fourth columns show 3D projections of the particle with spherical, ellipsoidal (thinner along the z-dimension), and particle-shaped masks, respectively. The fifth column displays the 3D projections of the enhanced and IsoNet missing-wedge-corrected particle. The sixth and seventh columns present the final 3D map and the flexibly fitted model, respectively. (B) Two random cross-sectional views (12 nm thickness) of the plasmid density map along its plectoneme axis are shown in the left-middle panel. The intensity profile along the line crossing the two high-density DNA spots is displayed in the right panel. (C) Resolution assessment of the final 3D map using Fourier shell correlation (FSC). Two criteria are shown: FSC between two half-maps reconstructed from even and odd frames (evaluated at 0.143) and FSC between the final 3D map and the fitted model (evaluated at 0.5). (D) Zoomed-in views of the final 3D density map from panel A, displayed at two contour levels. (E) Superimposition of the high-contour level map from panel D onto its flexibly fitted model.

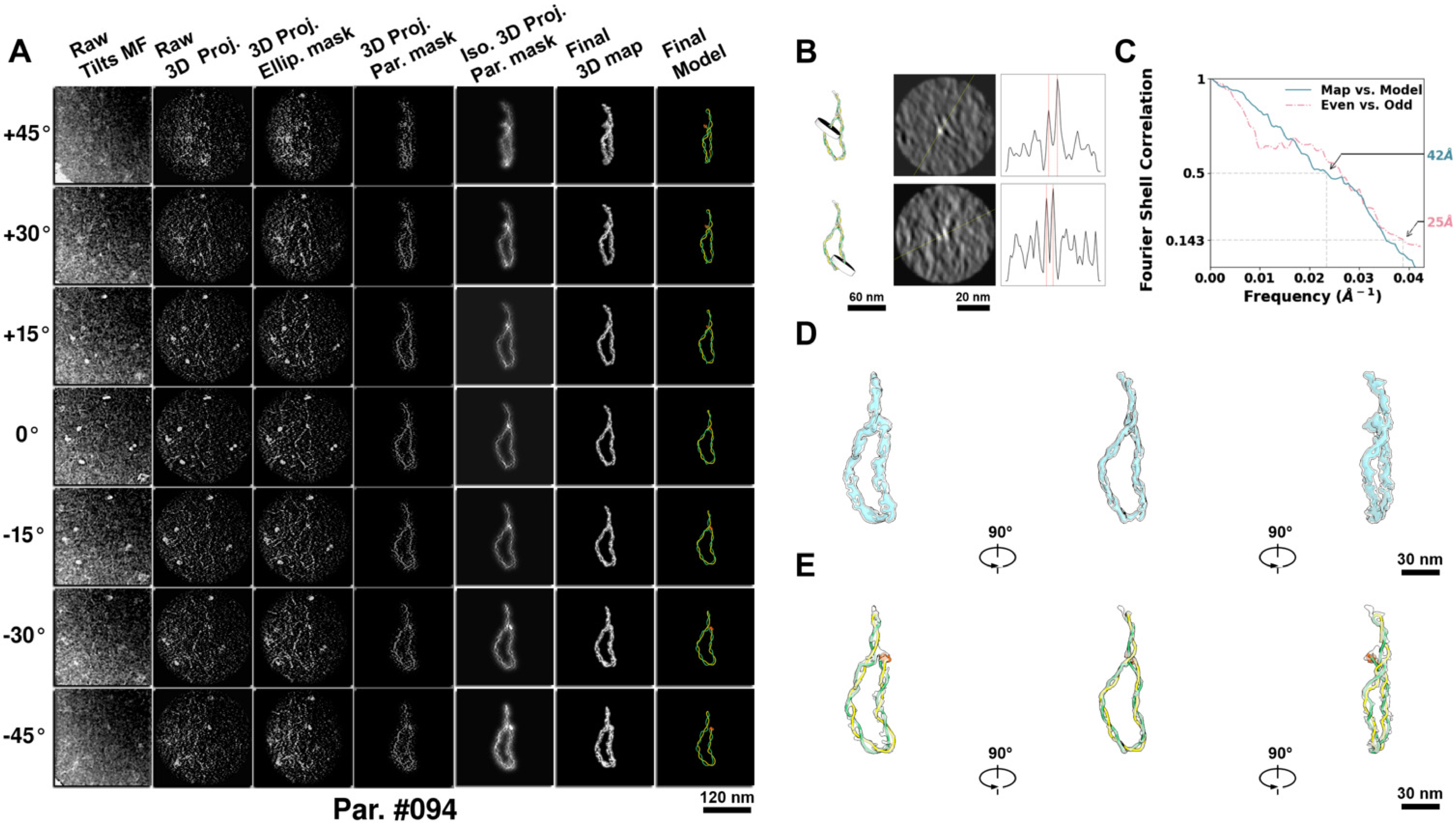

Supplementary Figure 94. Cryo-electron tomography (cryo-ET) 3D reconstruction of an individual plasmid particle in 40 mM K^+, 5 mM Mg^{2+} in the presence of dCas9.
(A) 3D reconstruction of the plasmid particle (index no. 94). The first column shows seven representative tilt images from –45° to +45° in step of 15°. The second, third, and fourth columns show 3D projections of the particle with spherical, ellipsoidal (thinner along the z-dimension), and particle-shaped masks, respectively. The fifth column displays the 3D projections of the enhanced and IsoNet missing-wedge-corrected particle. The sixth and seventh columns present the final 3D map and the flexibly fitted model, respectively. (B) Two random cross-sectional views (12 nm thickness) of the plasmid density map along its plectoneme axis are shown in the left-middle panel. The intensity profile along the line crossing the two high-density DNA spots is displayed in the right panel. (C) Resolution assessment of the final 3D map using Fourier shell correlation (FSC). Two criteria are shown: FSC between two half-maps reconstructed from even and odd frames (evaluated at 0.143) and FSC between the final 3D map and the fitted model (evaluated at 0.5). (D) Zoomed-in views of the final 3D density map from panel A, displayed at two contour levels. (E) Superimposition of the high-contour level map from panel D onto its flexibly fitted model.

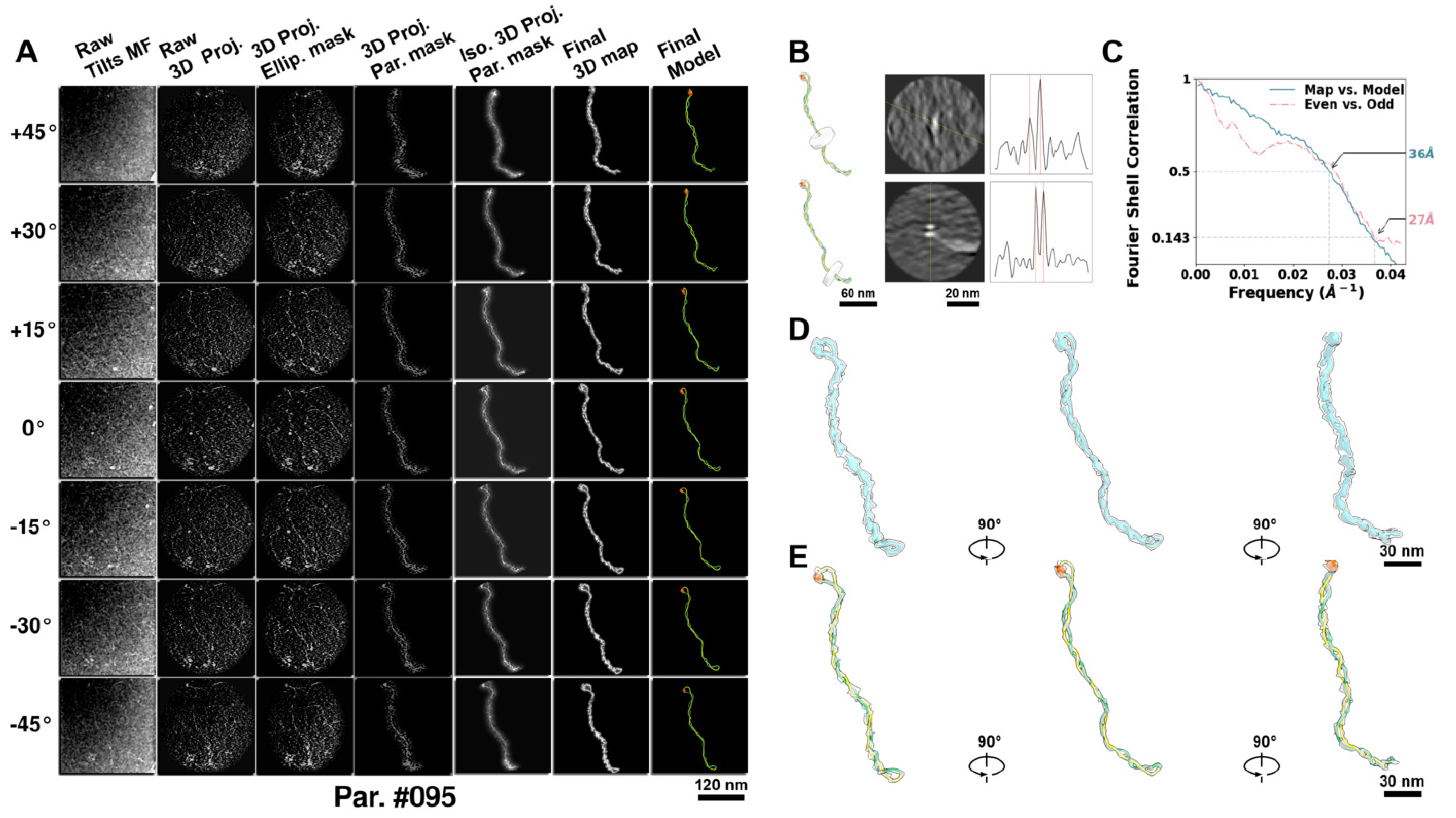

Supplementary Figure 95. Cryo-electron tomography (cryo-ET) 3D reconstruction of an individual plasmid particle in 40 mM K⁺, 5 mM Mg²⁺.
(A) 3D reconstruction of the plasmid particle (index no. 95). The first column shows seven representative tilt images from –45° to +45° in step of 15°. The second, third, and fourth columns show 3D projections of the particle with spherical, ellipsoidal (thinner along the z-dimension), and particle-shaped masks, respectively. The fifth column displays the 3D projections of the enhanced and IsoNet missing-wedge-corrected particle. The sixth and seventh columns present the final 3D map and the flexibly fitted model, respectively. (B) Two random cross-sectional views (12 nm thickness) of the plasmid density map along its plectoneme axis are shown in the left-middle panel. The intensity profile along the line crossing the two high-density DNA spots is displayed in the right panel. (C) Resolution assessment of the final 3D map using Fourier shell correlation (FSC). Two criteria are shown: FSC between two half-maps reconstructed from even and odd frames (evaluated at 0.143) and FSC between the final 3D map and the fitted model (evaluated at 0.5). (D) Zoomed-in views of the final 3D density map from panel A, displayed at two contour levels. (E) Superimposition of the high-contour level map from panel D onto its flexibly fitted model.

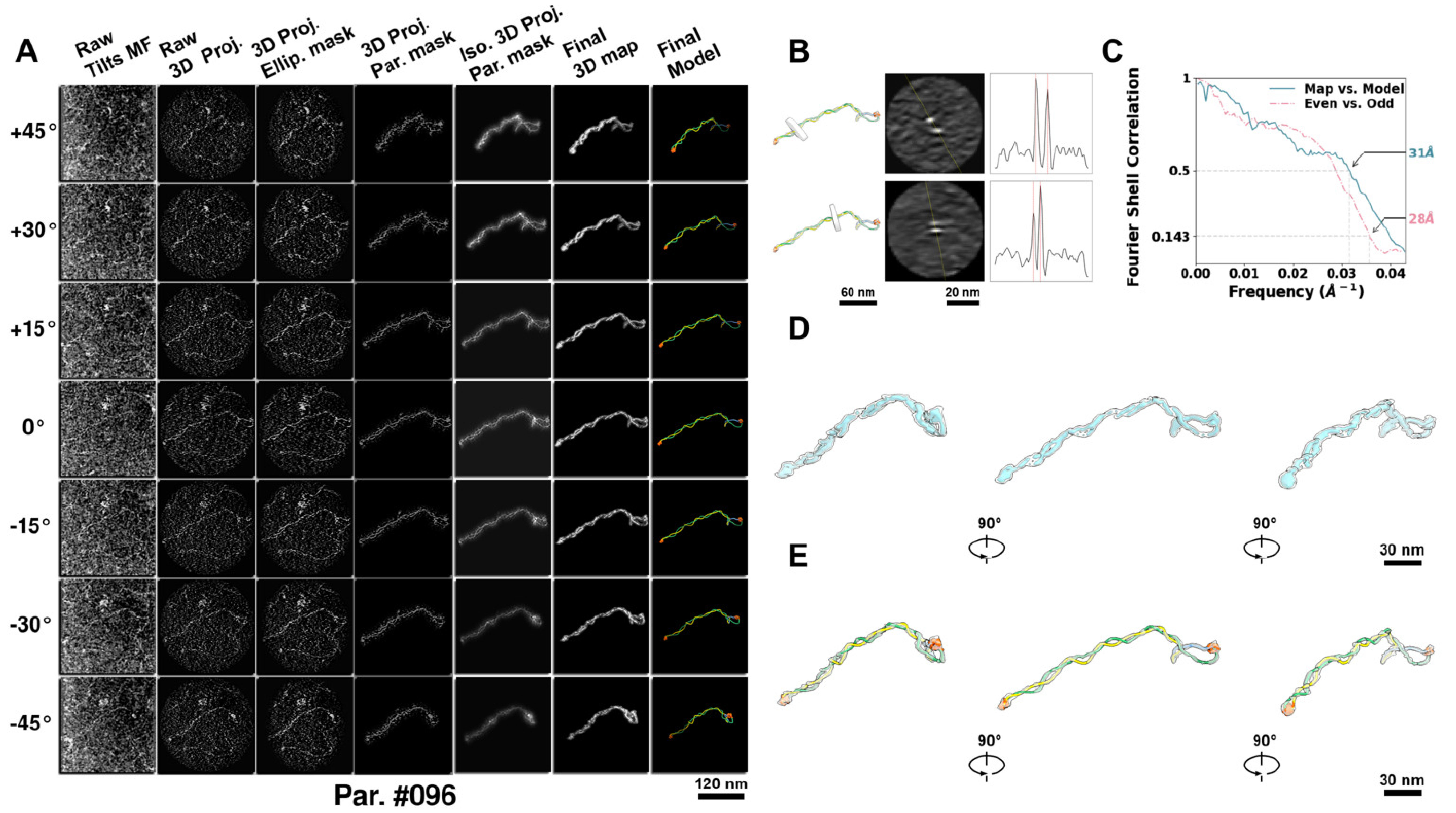

Supplementary Figure 96. Cryo-electron tomography (cryo-ET) 3D reconstruction of an individual plasmid particle in 40 mM K⁺, 5 mM Mg²⁺ in the presence of dCas9.
(A) 3D reconstruction of the plasmid particle (index no. 96). The first column shows seven representative tilt images from –45° to +45° in step of 15°. The second, third, and fourth columns show 3D projections of the particle with spherical, ellipsoidal (thinner along the z-dimension), and particle-shaped masks, respectively. The fifth column displays the 3D projections of the enhanced and IsoNet missing-wedge-corrected particle. The sixth and seventh columns present the final 3D map and the flexibly fitted model, respectively. (B) Two random cross-sectional views (12 nm thickness) of the plasmid density map along its plectoneme axis are shown in the left-middle panel. The intensity profile along the line crossing the two high-density DNA spots is displayed in the right panel. (C) Resolution assessment of the final 3D map using Fourier shell correlation (FSC). Two criteria are shown: FSC between two half-maps reconstructed from even and odd frames (evaluated at 0.143) and FSC between the final 3D map and the fitted model (evaluated at 0.5). (D) Zoomed-in views of the final 3D density map from panel A, displayed at two contour levels. (E) Superimposition of the high-contour level map from panel D onto its flexibly fitted model.

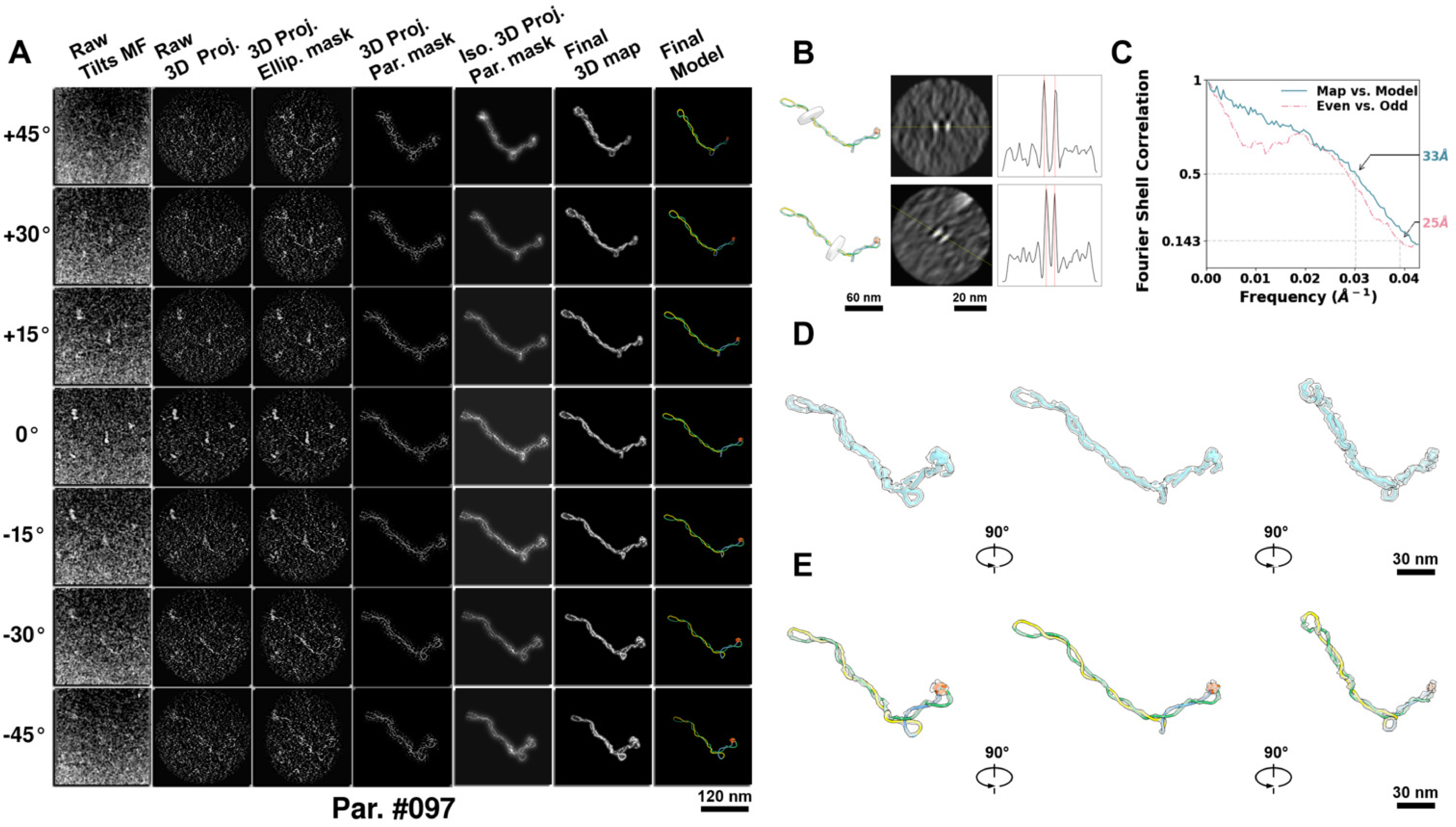

Supplementary Figure 97. Cryo-electron tomography (cryo-ET) 3D reconstruction of an individual plasmid particle in 40 mM K^{+}, 5 mM Mg^{2+} in the presence of dCas9.
(A) 3D reconstruction of the plasmid particle (index no. 97). The first column shows seven representative tilt images from –45° to +45° in step of 15°. The second, third, and fourth columns show 3D projections of the particle with spherical, ellipsoidal (thinner along the z-dimension), and particle-shaped masks, respectively. The fifth column displays the 3D projections of the enhanced and IsoNet missing-wedge-corrected particle. The sixth and seventh columns present the final 3D map and the flexibly fitted model, respectively. (B) Two random cross-sectional views (12 nm thickness) of the plasmid density map along its plectoneme axis are shown in the left-middle panel. The intensity profile along the line crossing the two high-density DNA spots is displayed in the right panel. (C) Resolution assessment of the final 3D map using Fourier shell correlation (FSC). Two criteria are shown: FSC between two half-maps reconstructed from even and odd frames (evaluated at 0.143) and FSC between the final 3D map and the fitted model (evaluated at 0.5). (D) Zoomed-in views of the final 3D density map from panel A, displayed at two contour levels. (E) Superimposition of the high-contour level map from panel D onto its flexibly fitted model.

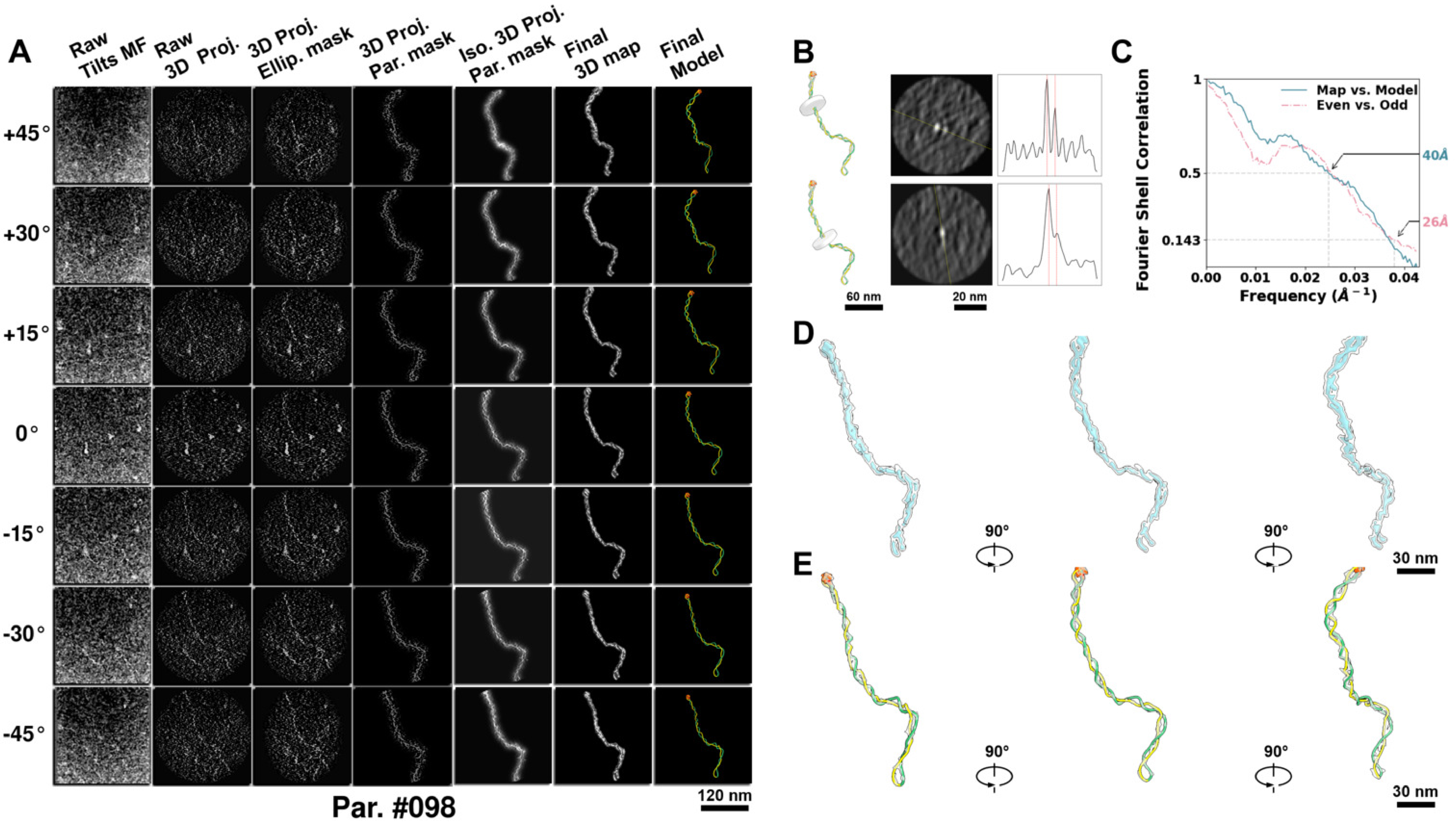

Supplementary Figure 98. Cryo-electron tomography (cryo-ET) 3D reconstruction of an individual plasmid particle in 40 mM K^{+}, 5 mM Mg^{2+} in the presence of dCas9.
(A) 3D reconstruction of the plasmid particle (index no. 98). The first column shows seven representative tilt images from –45° to +45° in step of 15°. The second, third, and fourth columns show 3D projections of the particle with spherical, ellipsoidal (thinner along the z-dimension), and particle-shaped masks, respectively. The fifth column displays the 3D projections of the enhanced and IsoNet missing-wedge-corrected particle. The sixth and seventh columns present the final 3D map and the flexibly fitted model, respectively. (B) Two random cross-sectional views (12 nm thickness) of the plasmid density map along its plectoneme axis are shown in the left-middle panel. The intensity profile along the line crossing the two high-density DNA spots is displayed in the right panel. (C) Resolution assessment of the final 3D map using Fourier shell correlation (FSC). Two criteria are shown: FSC between two half-maps reconstructed from even and odd frames (evaluated at 0.143) and FSC between the final 3D map and the fitted model (evaluated at 0.5). (D) Zoomed-in views of the final 3D density map from panel A, displayed at two contour levels. (E) Superimposition of the high-contour level map from panel D onto its flexibly fitted model.

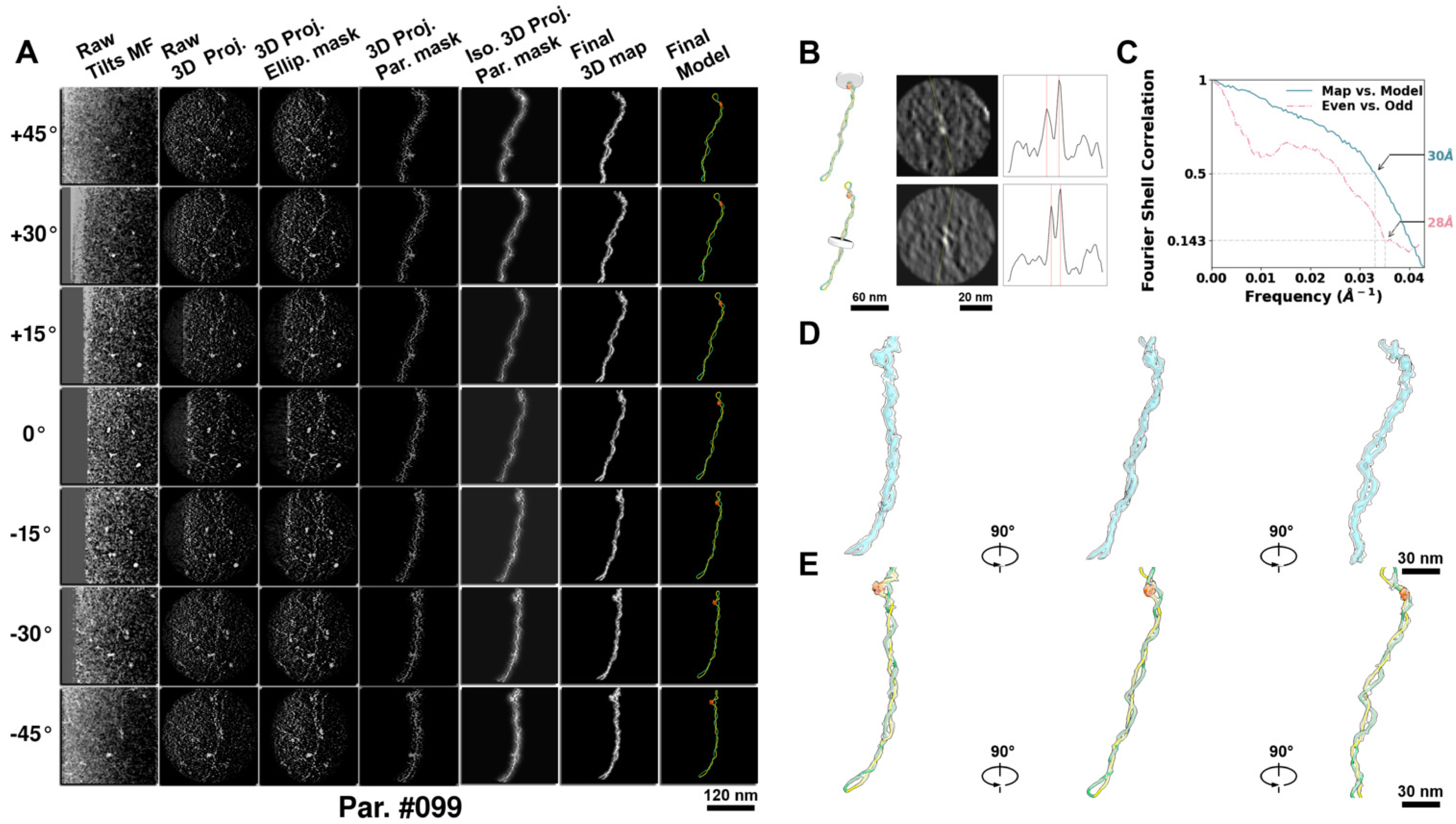

Supplementary Figure 99. Cryo-electron tomography (cryo-ET) 3D reconstruction of an individual plasmid particle in 40 mM K^+, 5 mM Mg^{2+} in the presence of dCas9.
(A) 3D reconstruction of the plasmid particle (index no. 99). The first column shows seven representative tilt images from –45° to +45° in step of 15°. The second, third, and fourth columns show 3D projections of the particle with spherical, ellipsoidal (thinner along the z-dimension), and particle-shaped masks, respectively. The fifth column displays the 3D projections of the enhanced and IsoNet missing-wedge-corrected particle. The sixth and seventh columns present the final 3D map and the flexibly fitted model, respectively. (B) Two random cross-sectional views (12 nm thickness) of the plasmid density map along its plectoneme axis are shown in the left-middle panel. The intensity profile along the line crossing the two high-density DNA spots is displayed in the right panel. (C) Resolution assessment of the final 3D map using Fourier shell correlation (FSC). Two criteria are shown: FSC between two half-maps reconstructed from even and odd frames (evaluated at 0.143) and FSC between the final 3D map and the fitted model (evaluated at 0.5). (D) Zoomed-in views of the final 3D density map from panel A, displayed at two contour levels. (E) Superimposition of the high-contour level map from panel D onto its flexibly fitted model.

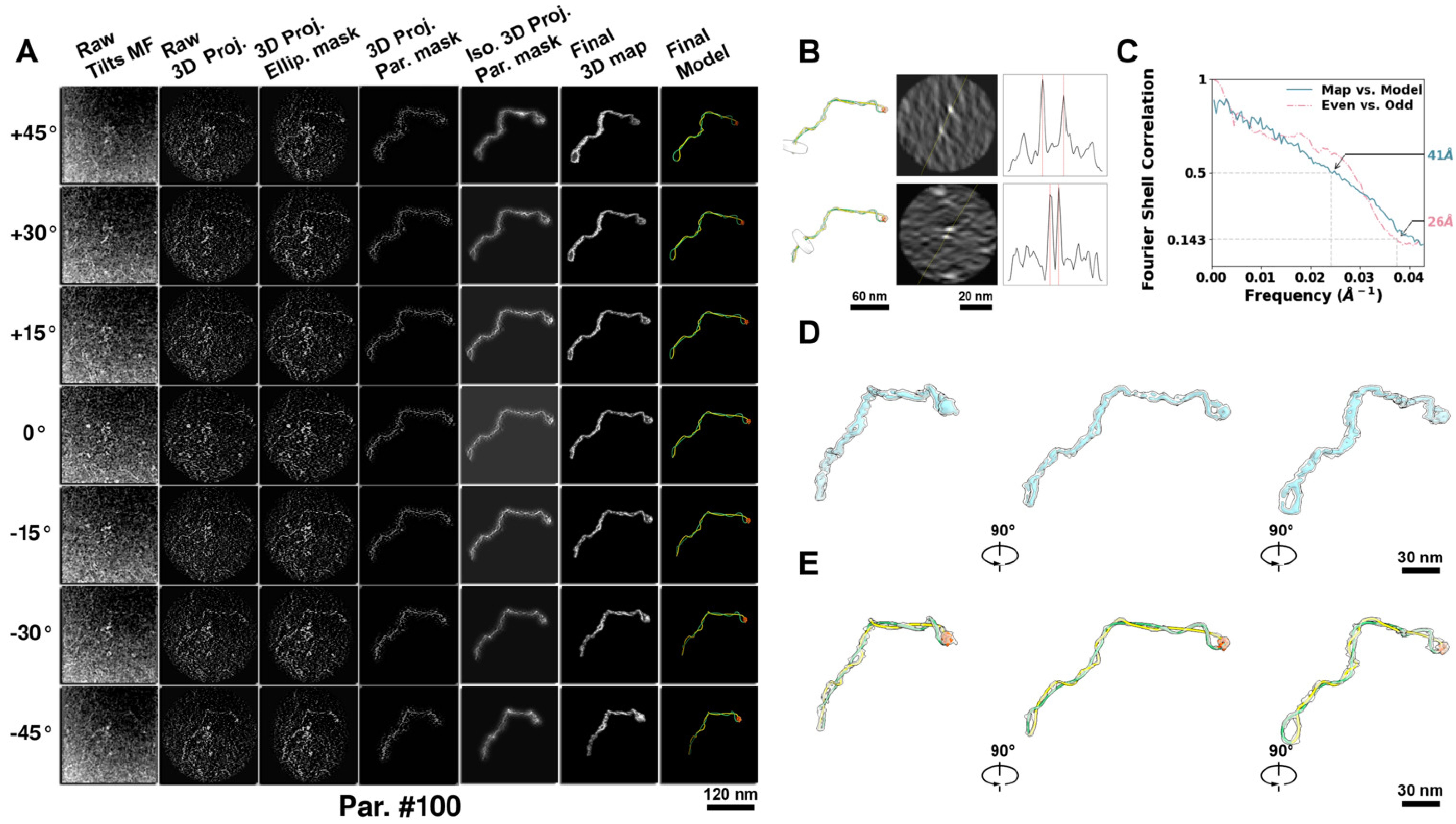

Supplementary Figure 100. Cryo-electron tomography (cryo-ET) 3D reconstruction of an individual plasmid particle in 40 mM K^+, 5 mM Mg^{2+} in the presence of dCas9.
(A) 3D reconstruction of the plasmid particle (index no. 100). The first column shows seven representative tilt images from –45° to +45° in step of 15°. The second, third, and fourth columns show 3D projections of the particle with spherical, ellipsoidal (thinner along the z-dimension), and particle-shaped masks, respectively. The fifth column displays the 3D projections of the enhanced and IsoNet missing-wedge-corrected particle. The sixth and seventh columns present the final 3D map and the flexibly fitted model, respectively. (B) Two random cross-sectional views (12 nm thickness) of the plasmid density map along its plectoneme axis are shown in the left-middle panel. The intensity profile along the line crossing the two high-density DNA spots is displayed in the right panel. (C) Resolution assessment of the final 3D map using Fourier shell correlation (FSC). Two criteria are shown: FSC between two half-maps reconstructed from even and odd frames (evaluated at 0.143) and FSC between the final 3D map and the fitted model (evaluated at 0.5). (D) Zoomed-in views of the final 3D density map from panel A, displayed at two contour levels. (E) Superimposition of the high-contour level map from panel D onto its flexibly fitted model.

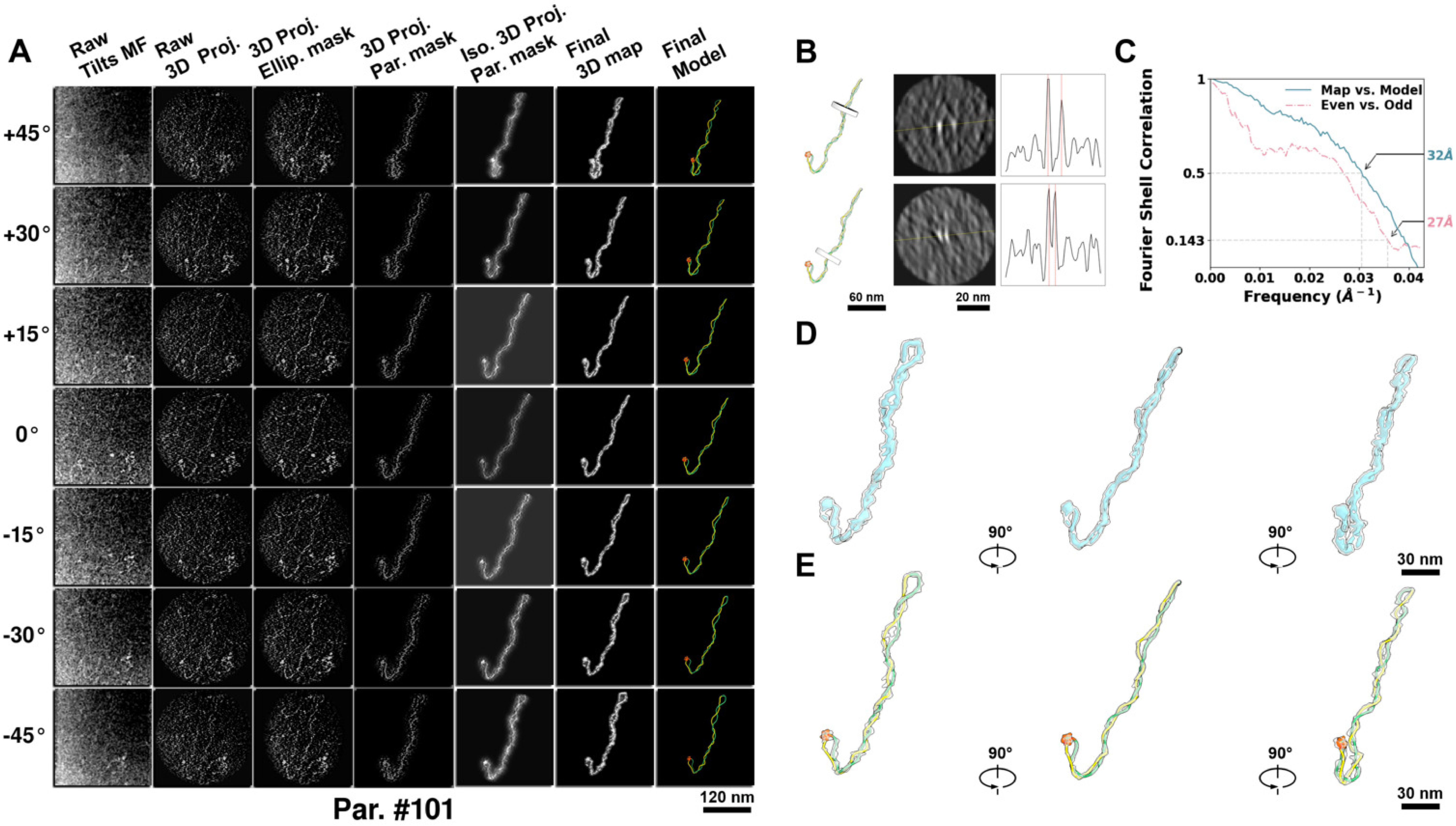

Supplementary Figure 101. Cryo-electron tomography (cryo-ET) 3D reconstruction of an individual plasmid particle in 40 mM K⁺, 5 mM Mg²⁺ in the presence of dCas9.
(A) 3D reconstruction of the plasmid particle (index no. 101). The first column shows seven representative tilt images from –45° to +45° in step of 15°. The second, third, and fourth columns show 3D projections of the particle with spherical, ellipsoidal (thinner along the z-dimension), and particle-shaped masks, respectively. The fifth column displays the 3D projections of the enhanced and IsoNet missing-wedge-corrected particle. The sixth and seventh columns present the final 3D map and the flexibly fitted model, respectively. (B) Two random cross-sectional views (12 nm thickness) of the plasmid density map along its plectoneme axis are shown in the left-middle panel. The intensity profile along the line crossing the two high-density DNA spots is displayed in the right panel. (C) Resolution assessment of the final 3D map using Fourier shell correlation (FSC). Two criteria are shown: FSC between two half-maps reconstructed from even and odd frames (evaluated at 0.143) and FSC between the final 3D map and the fitted model (evaluated at 0.5). (D) Zoomed-in views of the final 3D density map from panel A, displayed at two contour levels. (E) Superimposition of the high-contour level map from panel D onto its flexibly fitted model.

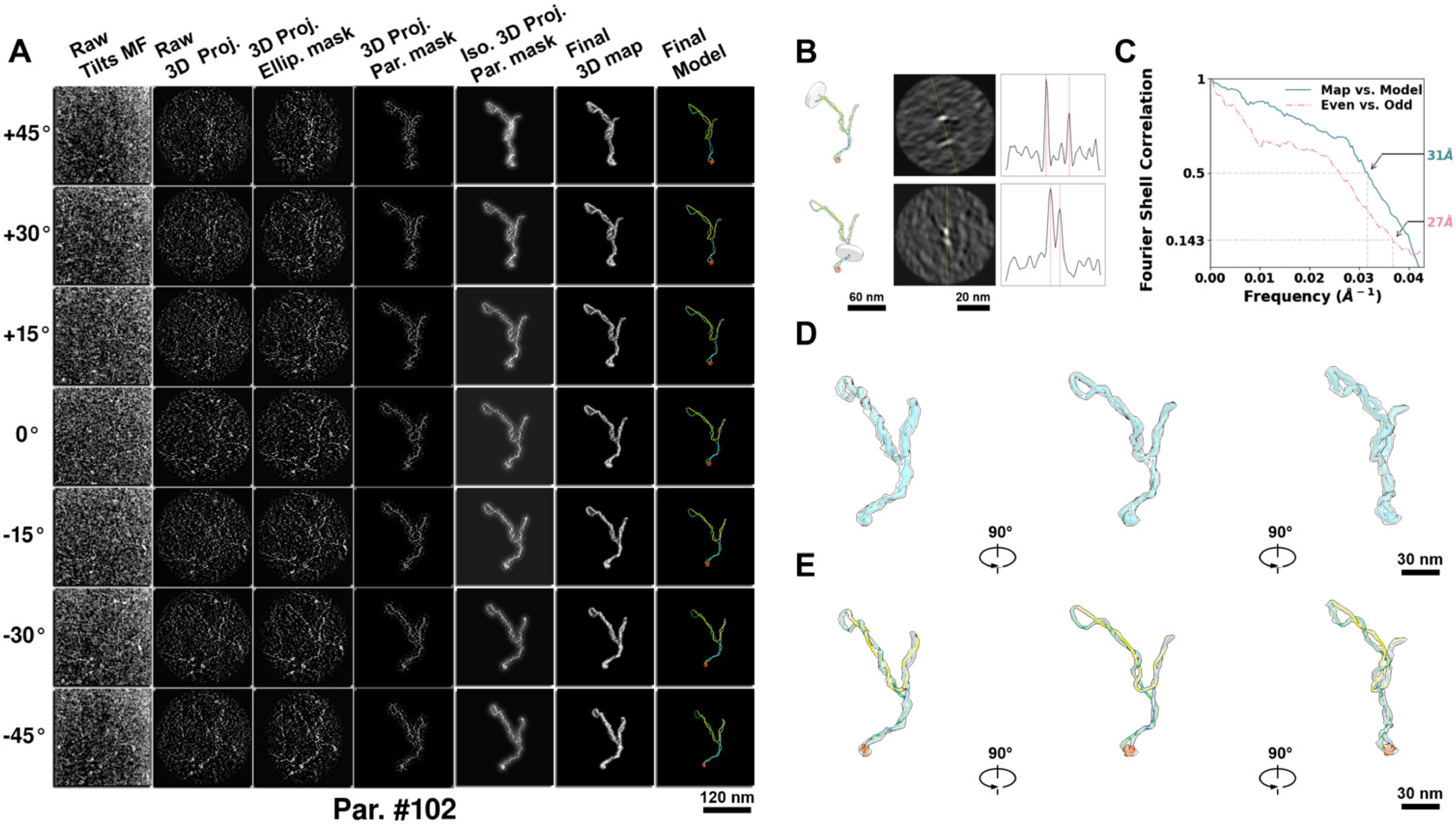

Supplementary Figure 102. Cryo-electron tomography (cryo-ET) 3D reconstruction of an individual plasmid particle in 40 mM K⁺, 5 mM Mg²⁺ in the presence of dCas9.
(A) 3D reconstruction of the plasmid particle (index no. 102). The first column shows seven representative tilt images from –45° to +45° in step of 15°. The second, third, and fourth columns show 3D projections of the particle with spherical, ellipsoidal (thinner along the z-dimension), and particle-shaped masks, respectively. The fifth column displays the 3D projections of the enhanced and IsoNet missing-wedge-corrected particle. The sixth and seventh columns present the final 3D map and the flexibly fitted model, respectively. (B) Two random cross-sectional views (12 nm thickness) of the plasmid density map along its plectoneme axis are shown in the left-middle panel. The intensity profile along the line crossing the two high-density DNA spots is displayed in the right panel. (C) Resolution assessment of the final 3D map using Fourier shell correlation (FSC). Two criteria are shown: FSC between two half-maps reconstructed from even and odd frames (evaluated at 0.143) and FSC between the final 3D map and the fitted model (evaluated at 0.5). (D) Zoomed-in views of the final 3D density map from panel A, displayed at two contour levels. (E) Superimposition of the high-contour level map from panel D onto its flexibly fitted model.

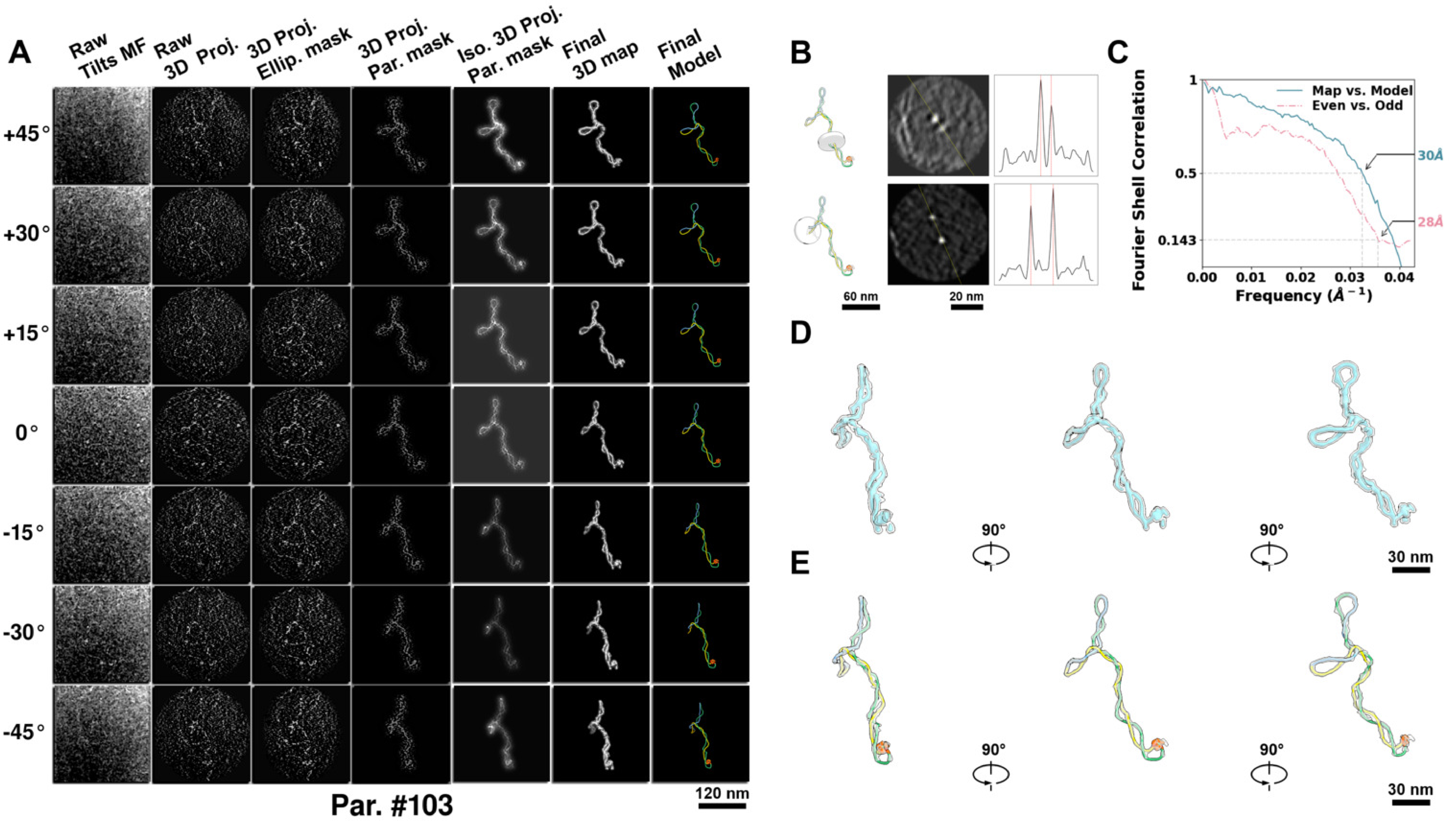

Supplementary Figure 103. Cryo-electron tomography (cryo-ET) 3D reconstruction of an individual plasmid particle in 40 mM K$^+$, 5 mM Mg^{2+} in the presence of dCas9.
(A) 3D reconstruction of the plasmid particle (index no. 103). The first column shows seven representative tilt images from –45° to +45° in step of 15°. The second, third, and fourth columns show 3D projections of the particle with spherical, ellipsoidal (thinner along the z-dimension), and particle-shaped masks, respectively. The fifth column displays the 3D projections of the enhanced and IsoNet missing-wedge-corrected particle. The sixth and seventh columns present the final 3D map and the flexibly fitted model, respectively. (B) Two random cross-sectional views (12 nm thickness) of the plasmid density map along its plectoneme axis are shown in the left-middle panel. The intensity profile along the line crossing the two high-density DNA spots is displayed in the right panel. (C) Resolution assessment of the final 3D map using Fourier shell correlation (FSC). Two criteria are shown: FSC between two half-maps reconstructed from even and odd frames (evaluated at 0.143) and FSC between the final 3D map and the fitted model (evaluated at 0.5). (D) Zoomed-in views of the final 3D density map from panel A, displayed at two contour levels. (E) Superimposition of the high-contour level map from panel D onto its flexibly fitted model.

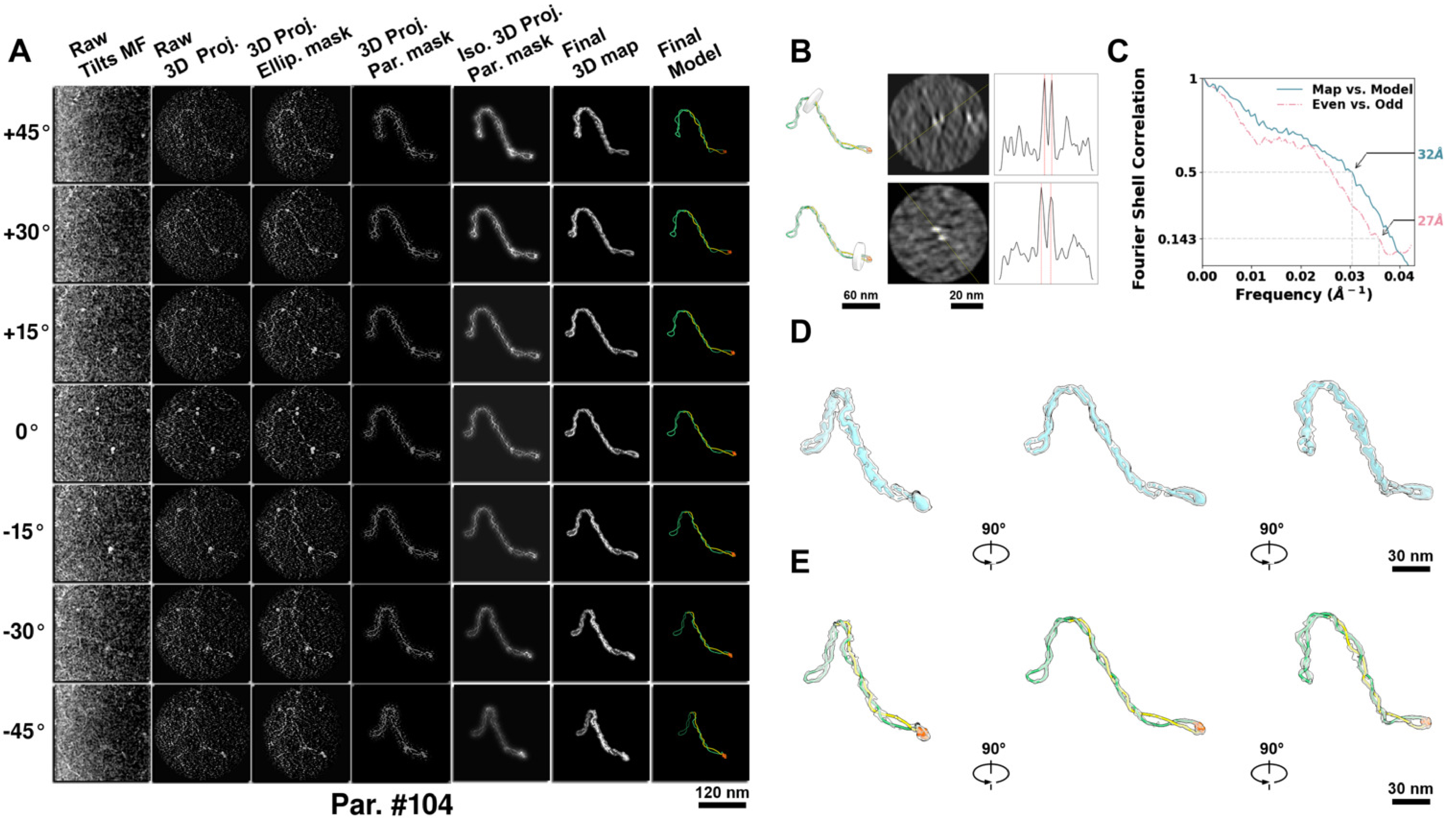

Supplementary Figure 104. Cryo-electron tomography (cryo-ET) 3D reconstruction of an individual plasmid particle in 40 mM K$^+$, 5 mM Mg^{2+} in the presence of dCas9.
(A) 3D reconstruction of the plasmid particle (index no. 104). The first column shows seven representative tilt images from –45° to +45° in step of 15°. The second, third, and fourth columns show 3D projections of the particle with spherical, ellipsoidal (thinner along the z-dimension), and particle-shaped masks, respectively. The fifth column displays the 3D projections of the enhanced and IsoNet missing-wedge-corrected particle. The sixth and seventh columns present the final 3D map and the flexibly fitted model, respectively. (B) Two random cross-sectional views (12 nm thickness) of the plasmid density map along its plectoneme axis are shown in the left-middle panel. The intensity profile along the line crossing the two high-density DNA spots is displayed in the right panel. (C) Resolution assessment of the final 3D map using Fourier shell correlation (FSC). Two criteria are shown: FSC between two half-maps reconstructed from even and odd frames (evaluated at 0.143) and FSC between the final 3D map and the fitted model (evaluated at 0.5). (D) Zoomed-in views of the final 3D density map from panel A, displayed at two contour levels. (E) Superimposition of the high-contour level map from panel D onto its flexibly fitted model.

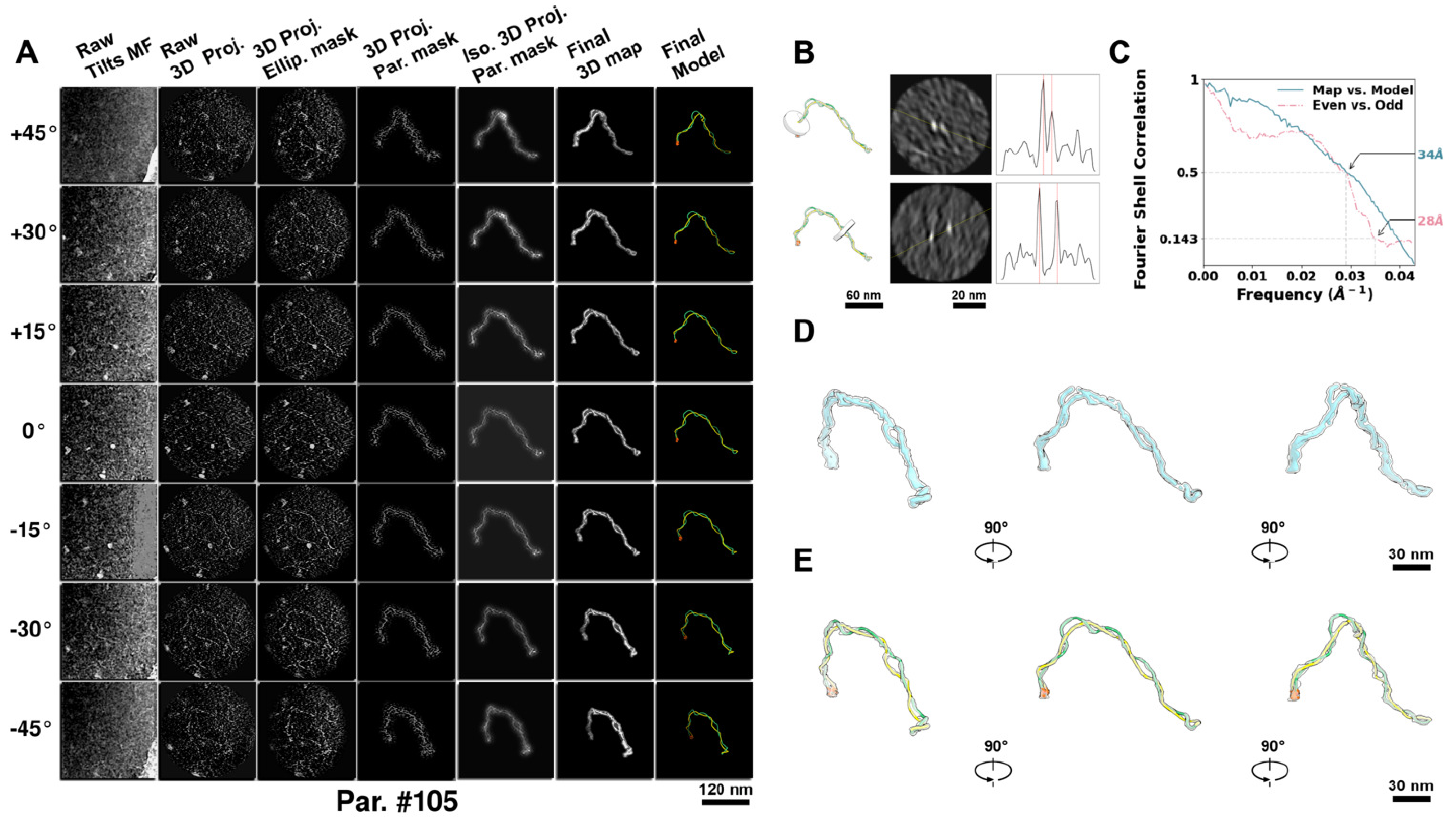

Supplementary Figure 105. Cryo-electron tomography (cryo-ET) 3D reconstruction of an individual plasmid particle in 40 mM K^+, 5 mM Mg^{2+} in the presence of dCas9.
(A) 3D reconstruction of the plasmid particle (index no. 105). The first column shows seven representative tilt images from –45° to +45° in step of 15°. The second, third, and fourth columns show 3D projections of the particle with spherical, ellipsoidal (thinner along the z-dimension), and particle-shaped masks, respectively. The fifth column displays the 3D projections of the enhanced and IsoNet missing-wedge-corrected particle. The sixth and seventh columns present the final 3D map and the flexibly fitted model, respectively. (B) Two random cross-sectional views (12 nm thickness) of the plasmid density map along its plectoneme axis are shown in the left-middle panel. The intensity profile along the line crossing the two high-density DNA spots is displayed in the right panel. (C) Resolution assessment of the final 3D map using Fourier shell correlation (FSC). Two criteria are shown: FSC between two half-maps reconstructed from even and odd frames (evaluated at 0.143) and FSC between the final 3D map and the fitted model (evaluated at 0.5). (D) Zoomed-in views of the final 3D density map from panel A, displayed at two contour levels. (E) Superimposition of the high-contour level map from panel D onto its flexibly fitted model.

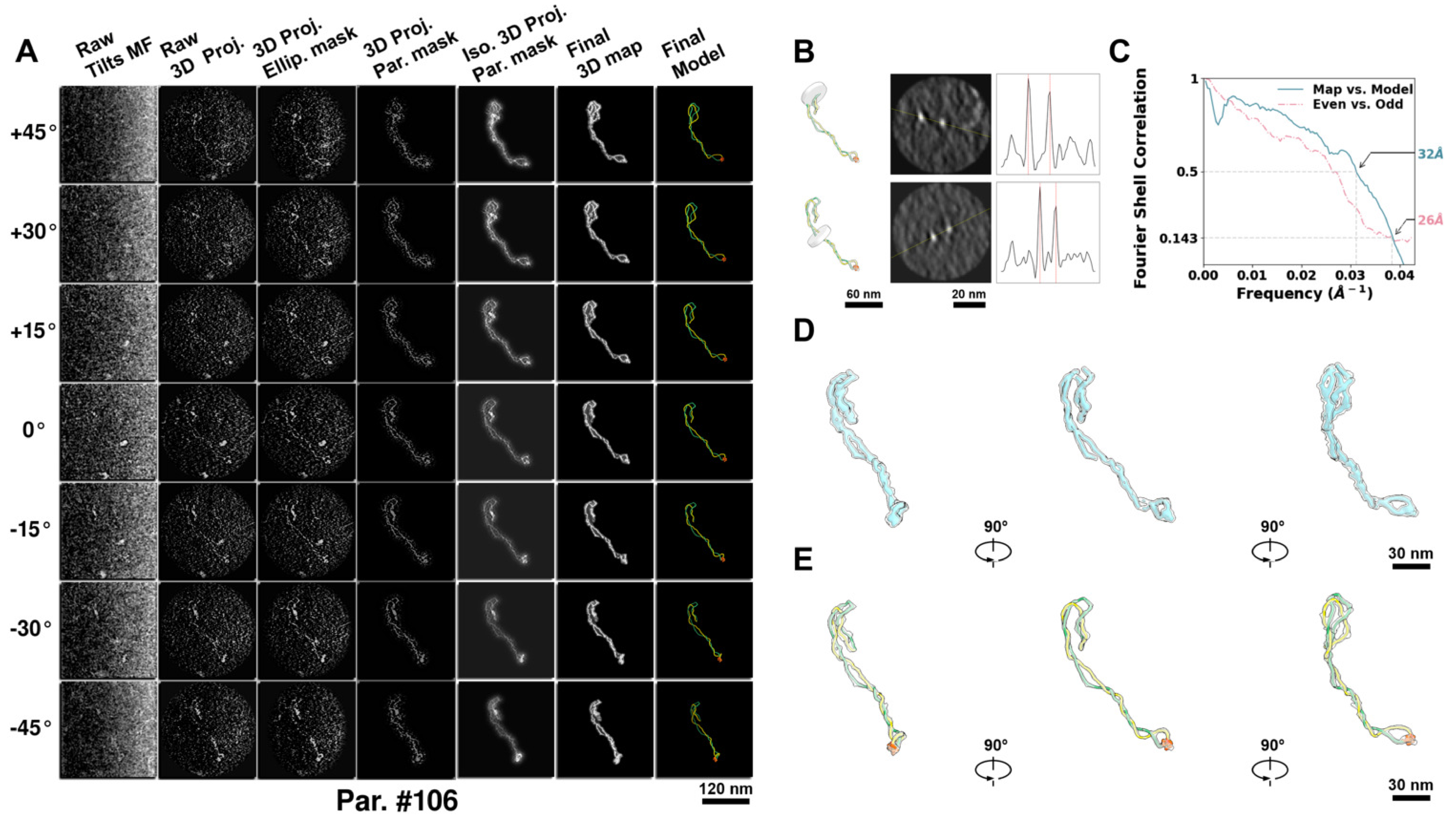

Supplementary Figure 106. Cryo-electron tomography (cryo-ET) 3D reconstruction of an individual plasmid particle in 40 mM K^+, 5 mM Mg^{2+} in the presence of dCas9.
(A) 3D reconstruction of the plasmid particle (index no. 106). The first column shows seven representative tilt images from –45° to +45° in step of 15°. The second, third, and fourth columns show 3D projections of the particle with spherical, ellipsoidal (thinner along the z-dimension), and particle-shaped masks, respectively. The fifth column displays the 3D projections of the enhanced and IsoNet missing-wedge-corrected particle. The sixth and seventh columns present the final 3D map and the flexibly fitted model, respectively. (B) Two random cross-sectional views (12 nm thickness) of the plasmid density map along its plectoneme axis are shown in the left-middle panel. The intensity profile along the line crossing the two high-density DNA spots is displayed in the right panel. (C) Resolution assessment of the final 3D map using Fourier shell correlation (FSC). Two criteria are shown: FSC between two half-maps reconstructed from even and odd frames (evaluated at 0.143) and FSC between the final 3D map and the fitted model (evaluated at 0.5). (D) Zoomed-in views of the final 3D density map from panel A, displayed at two contour levels. (E) Superimposition of the high-contour level map from panel D onto its flexibly fitted model.

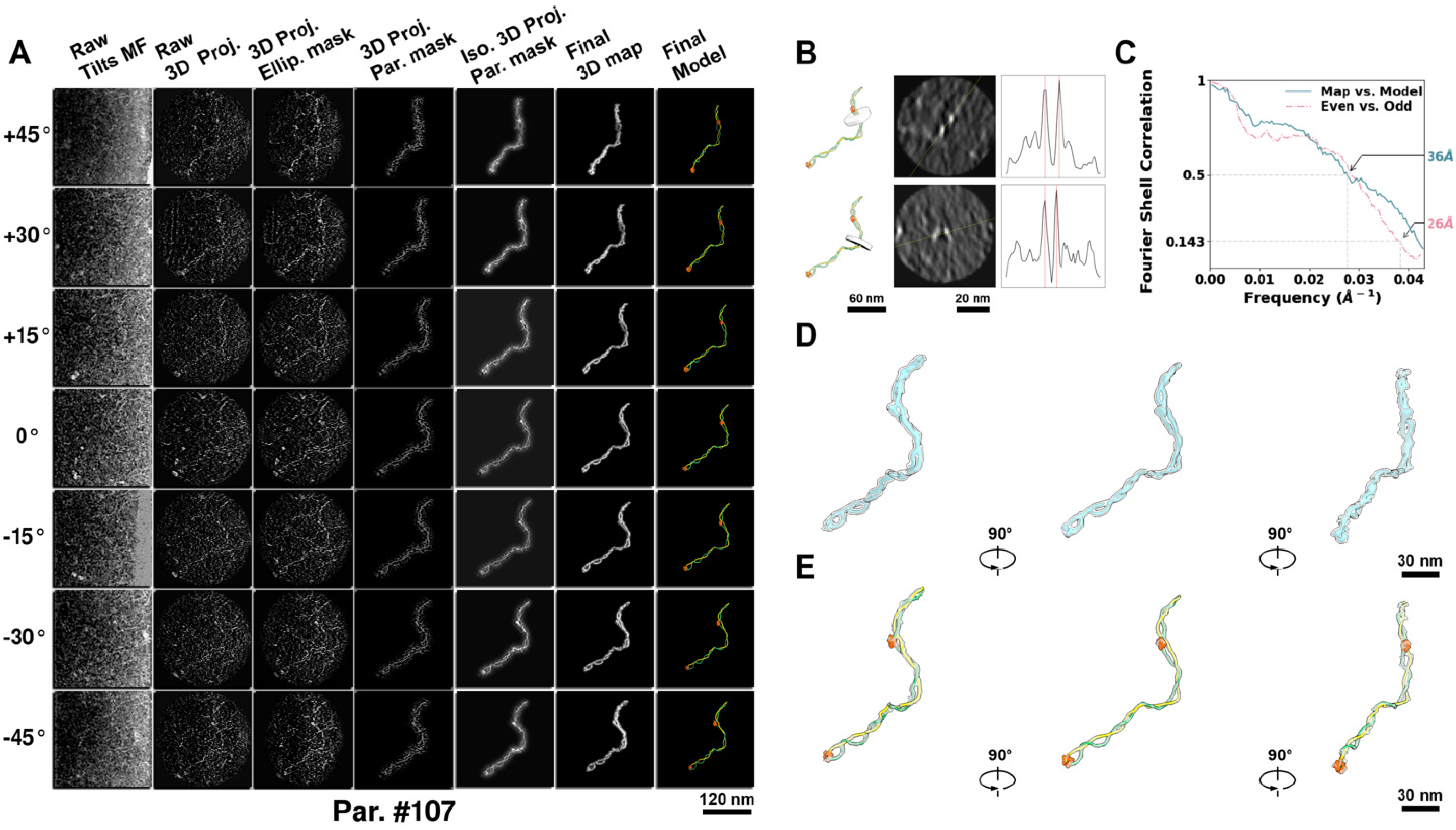

Supplementary Figure 107. Cryo-electron tomography (cryo-ET) 3D reconstruction of an individual plasmid particle in 40 mM K^{+}, 5 mM Mg^{2+} in the presence of dCas9.
(A) 3D reconstruction of the plasmid particle (index no. 107). The first column shows seven representative tilt images from –45° to +45° in step of 15°. The second, third, and fourth columns show 3D projections of the particle with spherical, ellipsoidal (thinner along the z-dimension), and particle-shaped masks, respectively. The fifth column displays the 3D projections of the enhanced and IsoNet missing-wedge-corrected particle. The sixth and seventh columns present the final 3D map and the flexibly fitted model, respectively. (B) Two random cross-sectional views (12 nm thickness) of the plasmid density map along its plectoneme axis are shown in the left-middle panel. The intensity profile along the line crossing the two high-density DNA spots is displayed in the right panel. (C) Resolution assessment of the final 3D map using Fourier shell correlation (FSC). Two criteria are shown: FSC between two half-maps reconstructed from even and odd frames (evaluated at 0.143) and FSC between the final 3D map and the fitted model (evaluated at 0.5). (D) Zoomed-in views of the final 3D density map from panel A, displayed at two contour levels. (E) Superimposition of the high-contour level map from panel D onto its flexibly fitted model.

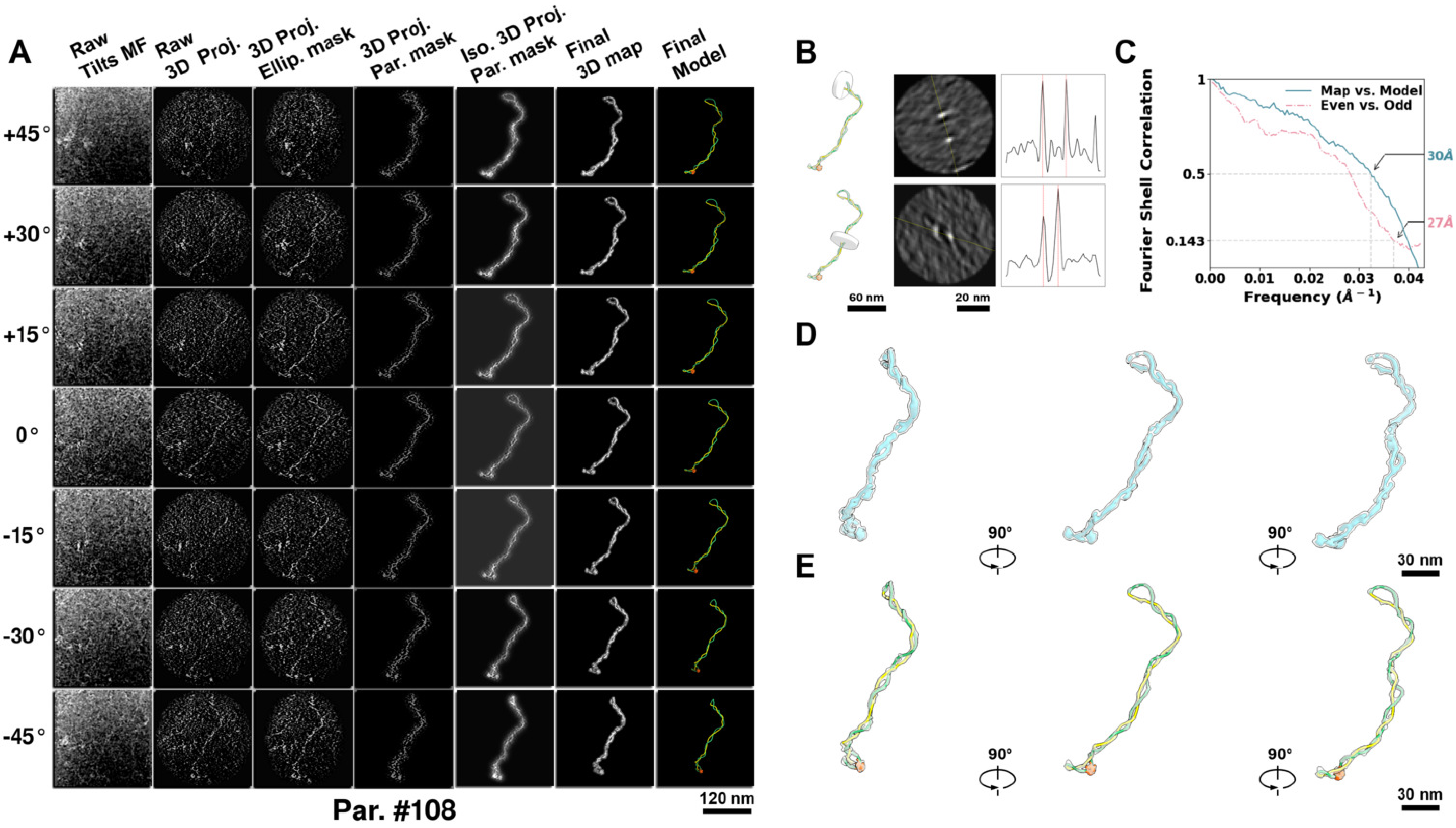

Supplementary Figure 108. Cryo-electron tomography (cryo-ET) 3D reconstruction of an individual plasmid particle in 40 mM K^{+}, 5 mM Mg^{2+} in the presence of dCas9.
(A) 3D reconstruction of the plasmid particle (index no. 108). The first column shows seven representative tilt images from –45° to +45° in step of 15°. The second, third, and fourth columns show 3D projections of the particle with spherical, ellipsoidal (thinner along the z-dimension), and particle-shaped masks, respectively. The fifth column displays the 3D projections of the enhanced and IsoNet missing-wedge-corrected particle. The sixth and seventh columns present the final 3D map and the flexibly fitted model, respectively. (B) Two random cross-sectional views (12 nm thickness) of the plasmid density map along its plectoneme axis are shown in the left-middle panel. The intensity profile along the line crossing the two high-density DNA spots is displayed in the right panel. (C) Resolution assessment of the final 3D map using Fourier shell correlation (FSC). Two criteria are shown: FSC between two half-maps reconstructed from even and odd frames (evaluated at 0.143) and FSC between the final 3D map and the fitted model (evaluated at 0.5). (D) Zoomed-in views of the final 3D density map from panel A, displayed at two contour levels. (E) Superimposition of the high-contour level map from panel D onto its flexibly fitted model.

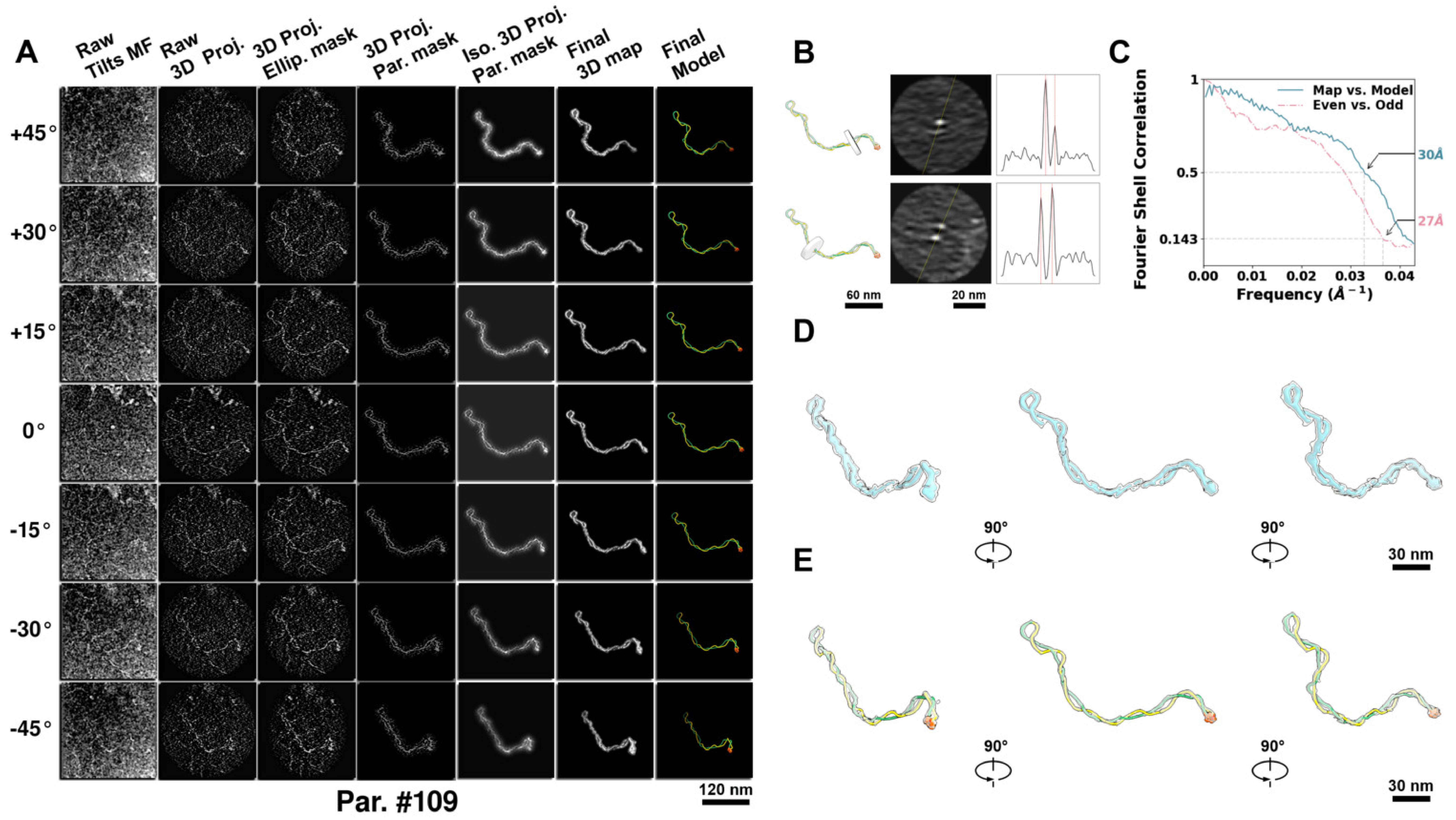

Supplementary Figure 109. Cryo-electron tomography (cryo-ET) 3D reconstruction of an individual plasmid particle in 40 mM K⁺, 5 mM Mg^{2+} in the presence of dCas9.
(A) 3D reconstruction of the plasmid particle (index no. 109). The first column shows seven representative tilt images from –45° to +45° in step of 15°. The second, third, and fourth columns show 3D projections of the particle with spherical, ellipsoidal (thinner along the z-dimension), and particle-shaped masks, respectively. The fifth column displays the 3D projections of the enhanced and IsoNet missing-wedge-corrected particle. The sixth and seventh columns present the final 3D map and the flexibly fitted model, respectively. (B) Two random cross-sectional views (12 nm thickness) of the plasmid density map along its plectoneme axis are shown in the left-middle panel. The intensity profile along the line crossing the two high-density DNA spots is displayed in the right panel. (C) Resolution assessment of the final 3D map using Fourier shell correlation (FSC). Two criteria are shown: FSC between two half-maps reconstructed from even and odd frames (evaluated at 0.143) and FSC between the final 3D map and the fitted model (evaluated at 0.5). (D) Zoomed-in views of the final 3D density map from panel A, displayed at two contour levels. (E) Superimposition of the high-contour level map from panel D onto its flexibly fitted model.

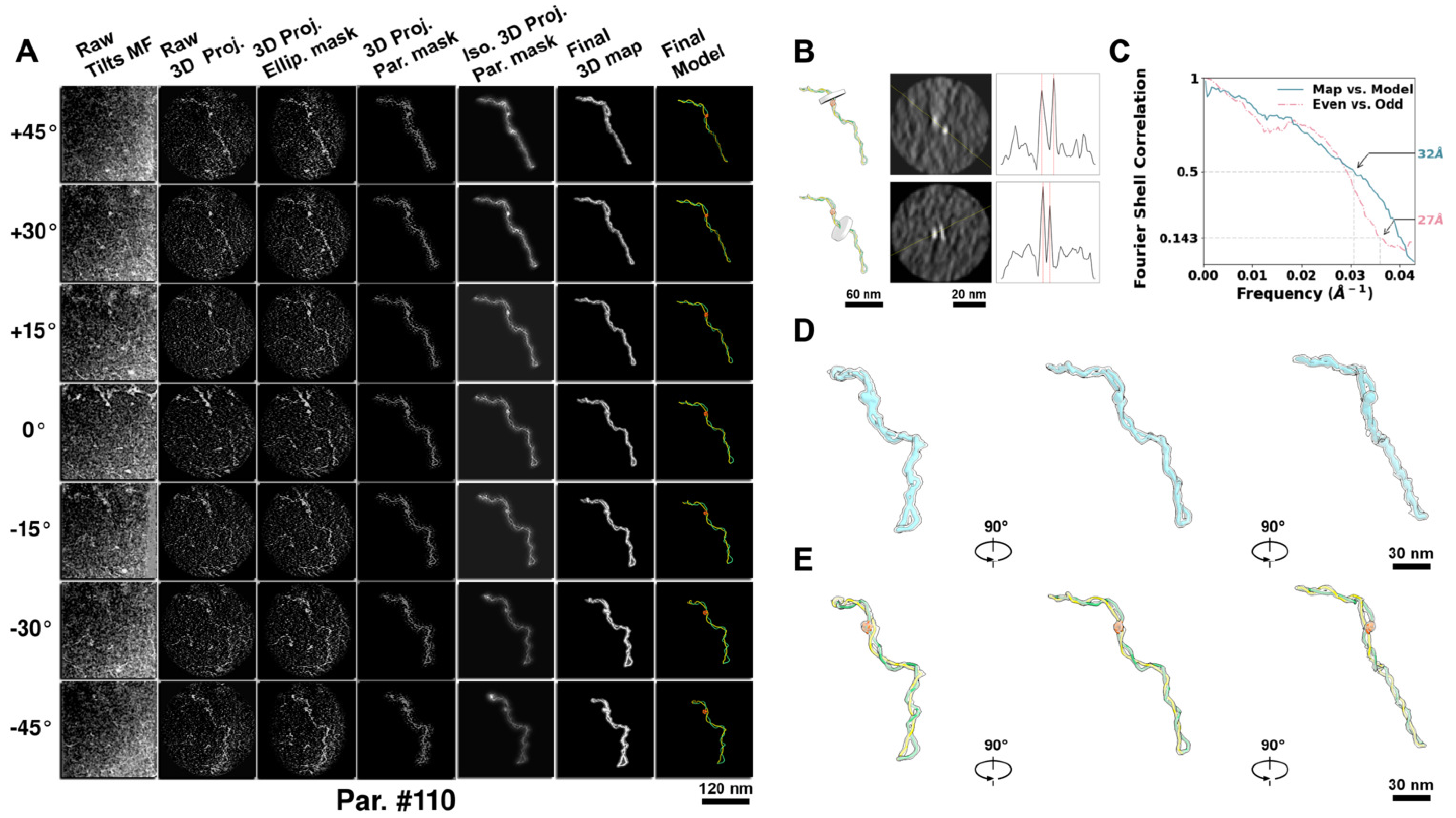

Supplementary Figure 110. Cryo-electron tomography (cryo-ET) 3D reconstruction of an individual plasmid particle in 40 mM K⁺, 5 mM Mg^{2+} in the presence of dCas9.
(A) 3D reconstruction of the plasmid particle (index no. 110). The first column shows seven representative tilt images from –45° to +45° in step of 15°. The second, third, and fourth columns show 3D projections of the particle with spherical, ellipsoidal (thinner along the z-dimension), and particle-shaped masks, respectively. The fifth column displays the 3D projections of the enhanced and IsoNet missing-wedge-corrected particle. The sixth and seventh columns present the final 3D map and the flexibly fitted model, respectively. (B) Two random cross-sectional views (12 nm thickness) of the plasmid density map along its plectoneme axis are shown in the left-middle panel. The intensity profile along the line crossing the two high-density DNA spots is displayed in the right panel. (C) Resolution assessment of the final 3D map using Fourier shell correlation (FSC). Two criteria are shown: FSC between two half-maps reconstructed from even and odd frames (evaluated at 0.143) and FSC between the final 3D map and the fitted model (evaluated at 0.5). (D) Zoomed-in views of the final 3D density map from panel A, displayed at two contour levels. (E) Superimposition of the high-contour level map from panel D onto its flexibly fitted model.

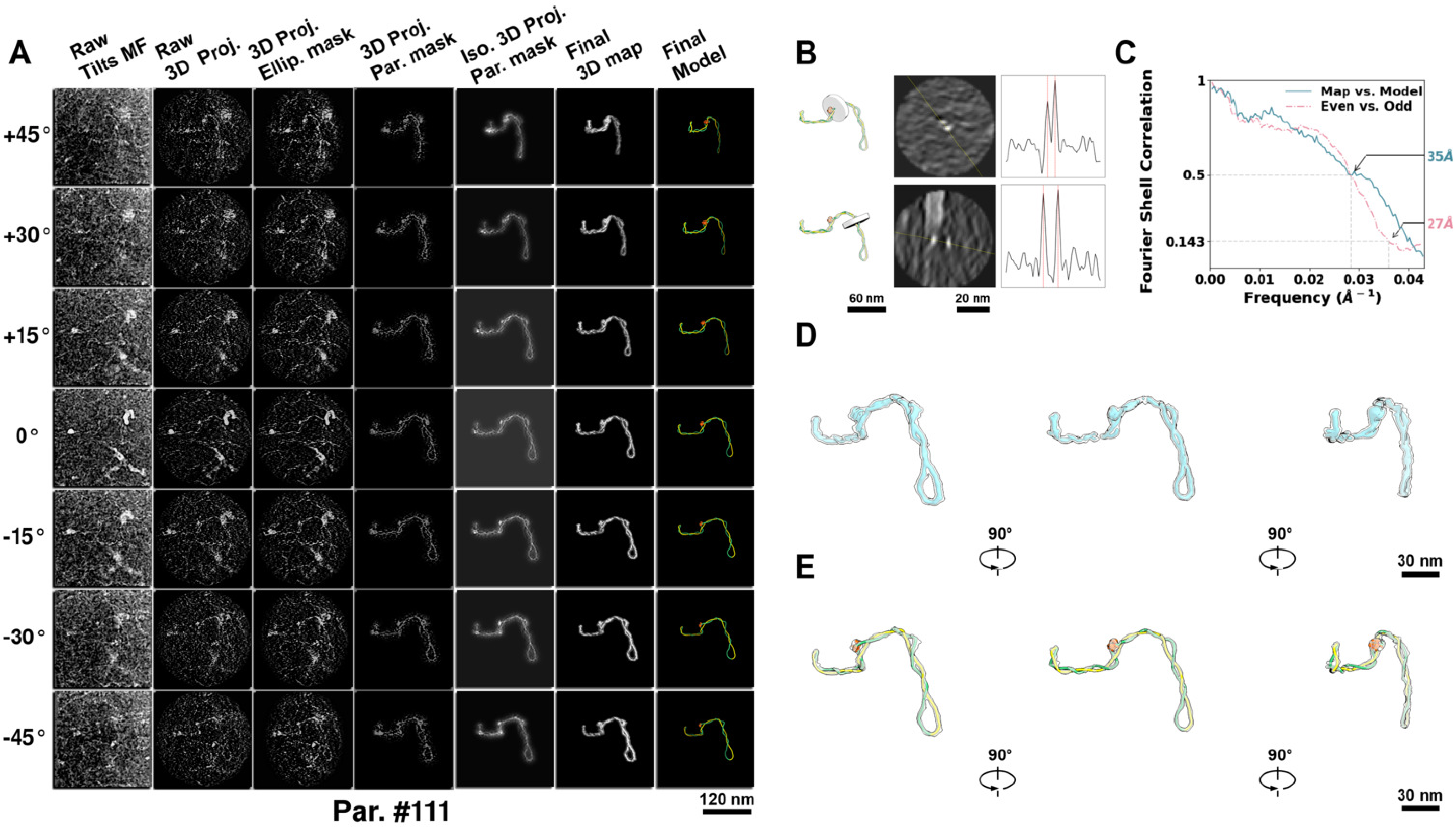

Supplementary Figure 111. Cryo-electron tomography (cryo-ET) 3D reconstruction of an individual plasmid particle in 40 mM K⁺, 5 mM Mg²⁺ in the presence of dCas9.
(A) 3D reconstruction of the plasmid particle (index no. 111). The first column shows seven representative tilt images from –45° to +45° in step of 15°. The second, third, and fourth columns show 3D projections of the particle with spherical, ellipsoidal (thinner along the z-dimension), and particle-shaped masks, respectively. The fifth column displays the 3D projections of the enhanced and IsoNet missing-wedge-corrected particle. The sixth and seventh columns present the final 3D map and the flexibly fitted model, respectively. (B) Two random cross-sectional views (12 nm thickness) of the plasmid density map along its plectoneme axis are shown in the left-middle panel. The intensity profile along the line crossing the two high-density DNA spots is displayed in the right panel. (C) Resolution assessment of the final 3D map using Fourier shell correlation (FSC). Two criteria are shown: FSC between two half-maps reconstructed from even and odd frames (evaluated at 0.143) and FSC between the final 3D map and the fitted model (evaluated at 0.5). (D) Zoomed-in views of the final 3D density map from panel A, displayed at two contour levels. (E) Superimposition of the high-contour level map from panel D onto its flexibly fitted model.

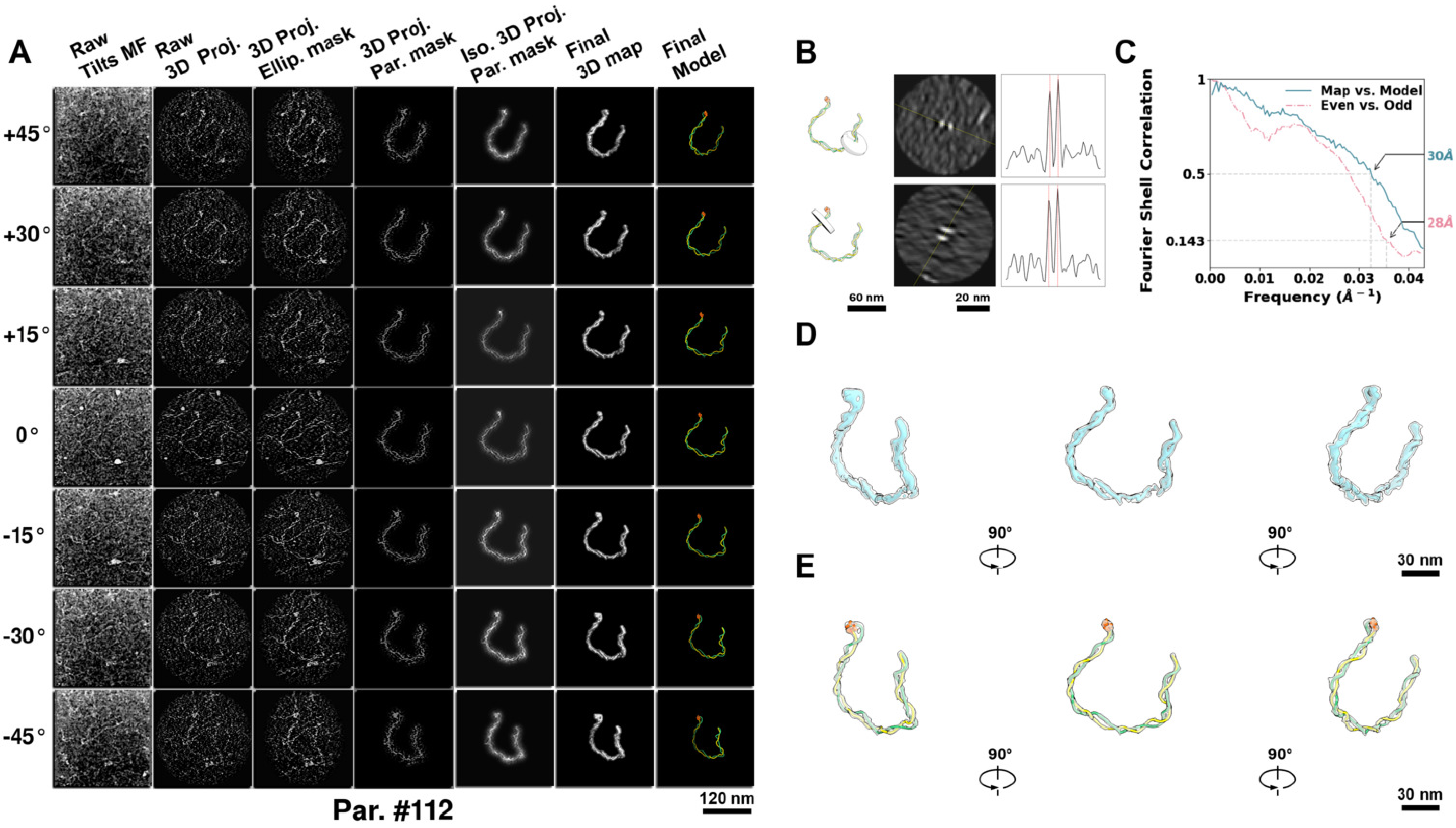

Supplementary Figure 112. Cryo-electron tomography (cryo-ET) 3D reconstruction of an individual plasmid particle in 40 mM K⁺, 5 mM Mg²⁺ in the presence of dCas9.
(A) 3D reconstruction of the plasmid particle (index no. 112). The first column shows seven representative tilt images from –45° to +45° in step of 15°. The second, third, and fourth columns show 3D projections of the particle with spherical, ellipsoidal (thinner along the z-dimension), and particle-shaped masks, respectively. The fifth column displays the 3D projections of the enhanced and IsoNet missing-wedge-corrected particle. The sixth and seventh columns present the final 3D map and the flexibly fitted model, respectively. (B) Two random cross-sectional views (12 nm thickness) of the plasmid density map along its plectoneme axis are shown in the left-middle panel. The intensity profile along the line crossing the two high-density DNA spots is displayed in the right panel. (C) Resolution assessment of the final 3D map using Fourier shell correlation (FSC). Two criteria are shown: FSC between two half-maps reconstructed from even and odd frames (evaluated at 0.143) and FSC between the final 3D map and the fitted model (evaluated at 0.5). (D) Zoomed-in views of the final 3D density map from panel A, displayed at two contour levels. (E) Superimposition of the high-contour level map from panel D onto its flexibly fitted model.

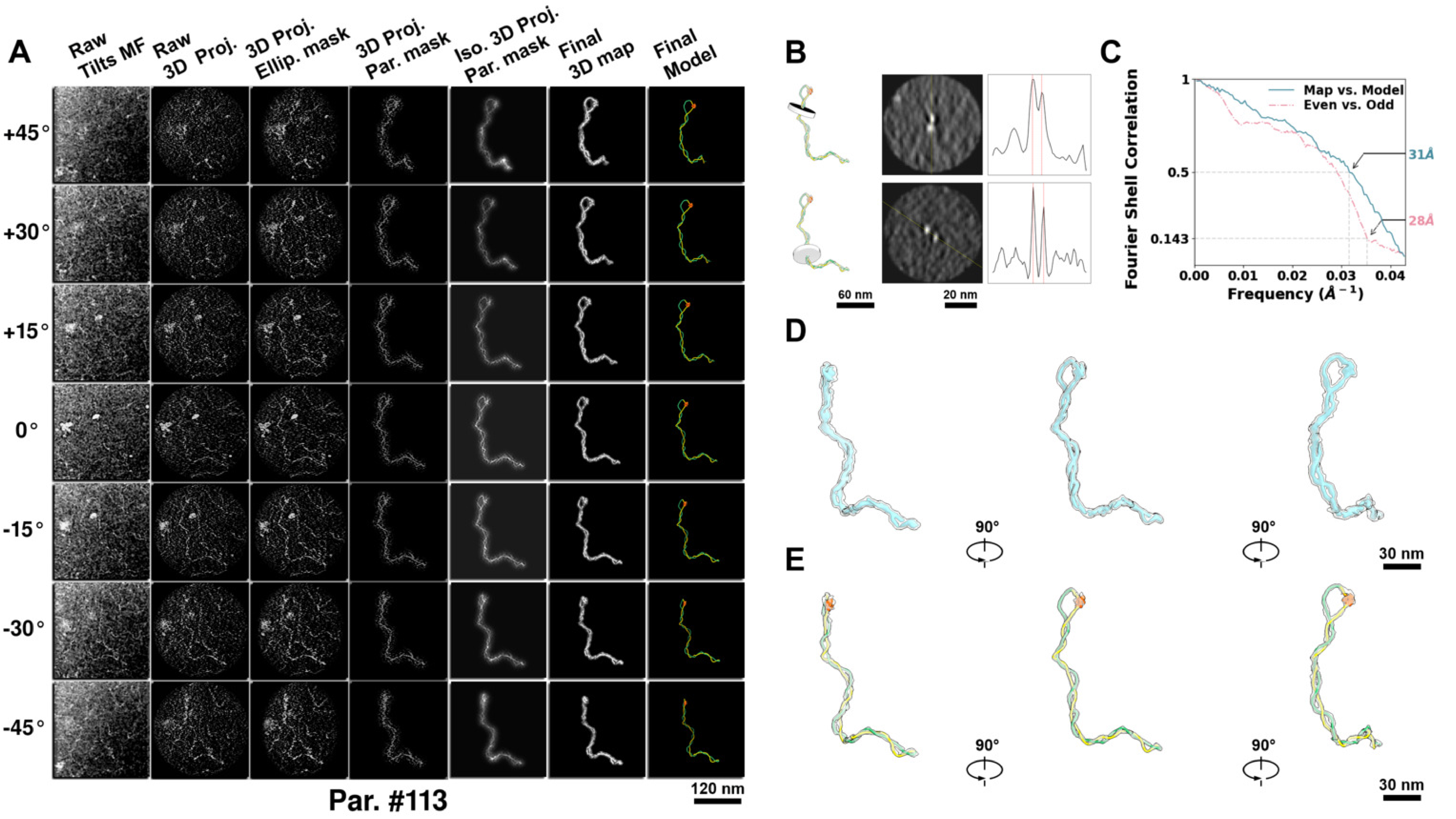

Supplementary Figure 113. Cryo-electron tomography (cryo-ET) 3D reconstruction of an individual plasmid particle in 40 mM K⁺, 5 mM Mg^{2+} in the presence of dCas9.
(A) 3D reconstruction of the plasmid particle (index no. 113). The first column shows seven representative tilt images from –45° to +45° in step of 15°. The second, third, and fourth columns show 3D projections of the particle with spherical, ellipsoidal (thinner along the z-dimension), and particle-shaped masks, respectively. The fifth column displays the 3D projections of the enhanced and IsoNet missing-wedge-corrected particle. The sixth and seventh columns present the final 3D map and the flexibly fitted model, respectively. (B) Two random cross-sectional views (12 nm thickness) of the plasmid density map along its plectoneme axis are shown in the left-middle panel. The intensity profile along the line crossing the two high-density DNA spots is displayed in the right panel. (C) Resolution assessment of the final 3D map using Fourier shell correlation (FSC). Two criteria are shown: FSC between two half-maps reconstructed from even and odd frames (evaluated at 0.143) and FSC between the final 3D map and the fitted model (evaluated at 0.5). (D) Zoomed-in views of the final 3D density map from panel A, displayed at two contour levels. (E) Superimposition of the high-contour level map from panel D onto its flexibly fitted model.

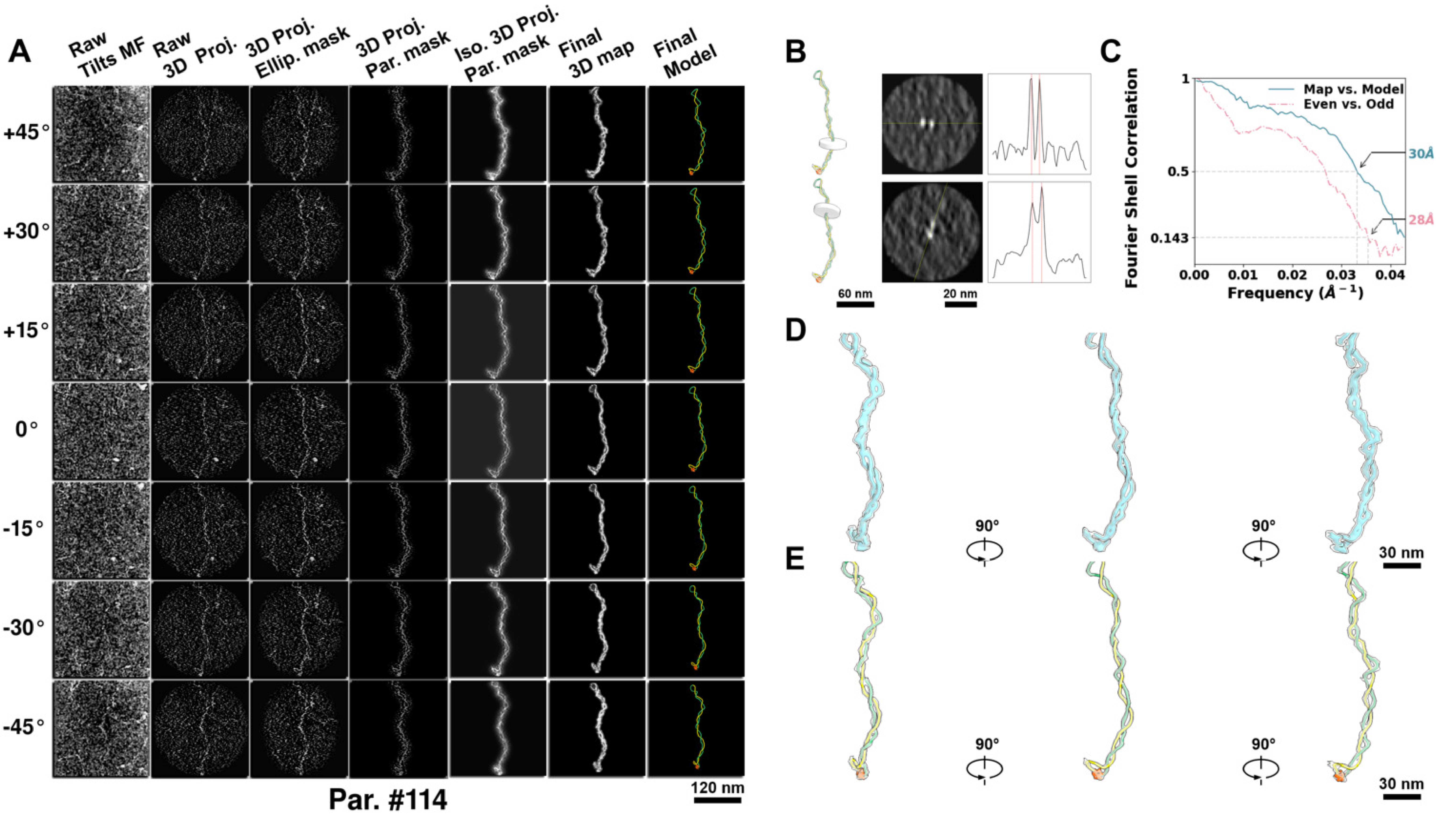

Supplementary Figure 114. Cryo-electron tomography (cryo-ET) 3D reconstruction of an individual plasmid particle in 40 mM K⁺, 5 mM Mg^{2+} in the presence of dCas9.
(A) 3D reconstruction of the plasmid particle (index no. 114). The first column shows seven representative tilt images from –45° to +45° in step of 15°. The second, third, and fourth columns show 3D projections of the particle with spherical, ellipsoidal (thinner along the z-dimension), and particle-shaped masks, respectively. The fifth column displays the 3D projections of the enhanced and IsoNet missing-wedge-corrected particle. The sixth and seventh columns present the final 3D map and the flexibly fitted model, respectively. (B) Two random cross-sectional views (12 nm thickness) of the plasmid density map along its plectoneme axis are shown in the left-middle panel. The intensity profile along the line crossing the two high-density DNA spots is displayed in the right panel. (C) Resolution assessment of the final 3D map using Fourier shell correlation (FSC). Two criteria are shown: FSC between two half-maps reconstructed from even and odd frames (evaluated at 0.143) and FSC between the final 3D map and the fitted model (evaluated at 0.5). (D) Zoomed-in views of the final 3D density map from panel A, displayed at two contour levels. (E) Superimposition of the high-contour level map from panel D onto its flexibly fitted model.

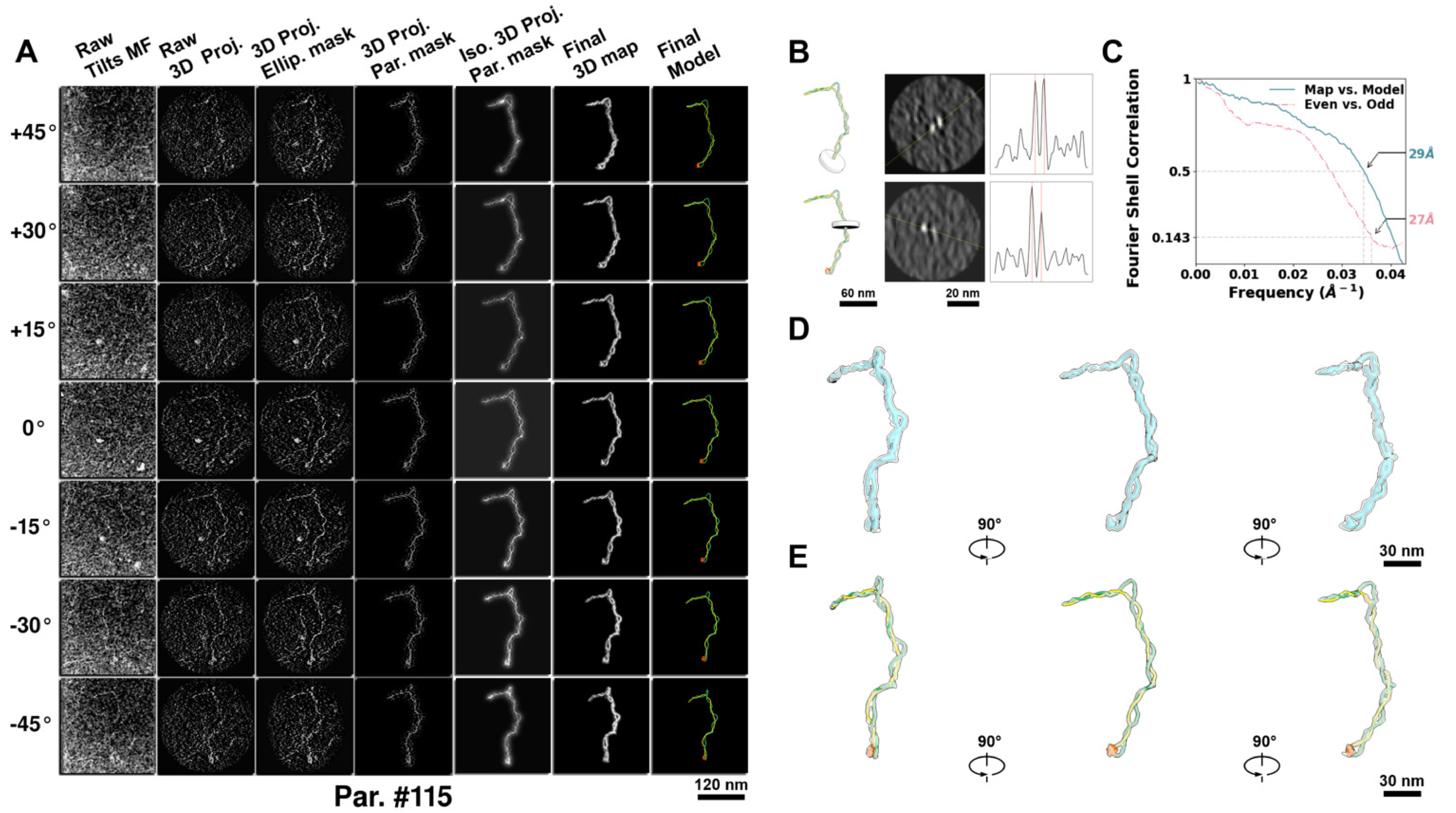

Supplementary Figure 115. Cryo-electron tomography (cryo-ET) 3D reconstruction of an individual plasmid particle in 40 mM K⁺, 5 mM Mg^{2+} in the presence of dCas9.
(A) 3D reconstruction of the plasmid particle (index no. 115). The first column shows seven representative tilt images from –45° to +45° in step of 15°. The second, third, and fourth columns show 3D projections of the particle with spherical, ellipsoidal (thinner along the z-dimension), and particle-shaped masks, respectively. The fifth column displays the 3D projections of the enhanced and IsoNet missing-wedge-corrected particle. The sixth and seventh columns present the final 3D map and the flexibly fitted model, respectively. (B) Two random cross-sectional views (12 nm thickness) of the plasmid density map along its plectoneme axis are shown in the left-middle panel. The intensity profile along the line crossing the two high-density DNA spots is displayed in the right panel. (C) Resolution assessment of the final 3D map using Fourier shell correlation (FSC). Two criteria are shown: FSC between two half-maps reconstructed from even and odd frames (evaluated at 0.143) and FSC between the final 3D map and the fitted model (evaluated at 0.5). (D) Zoomed-in views of the final 3D density map from panel A, displayed at two contour levels. (E) Superimposition of the high-contour level map from panel D onto its flexibly fitted model.

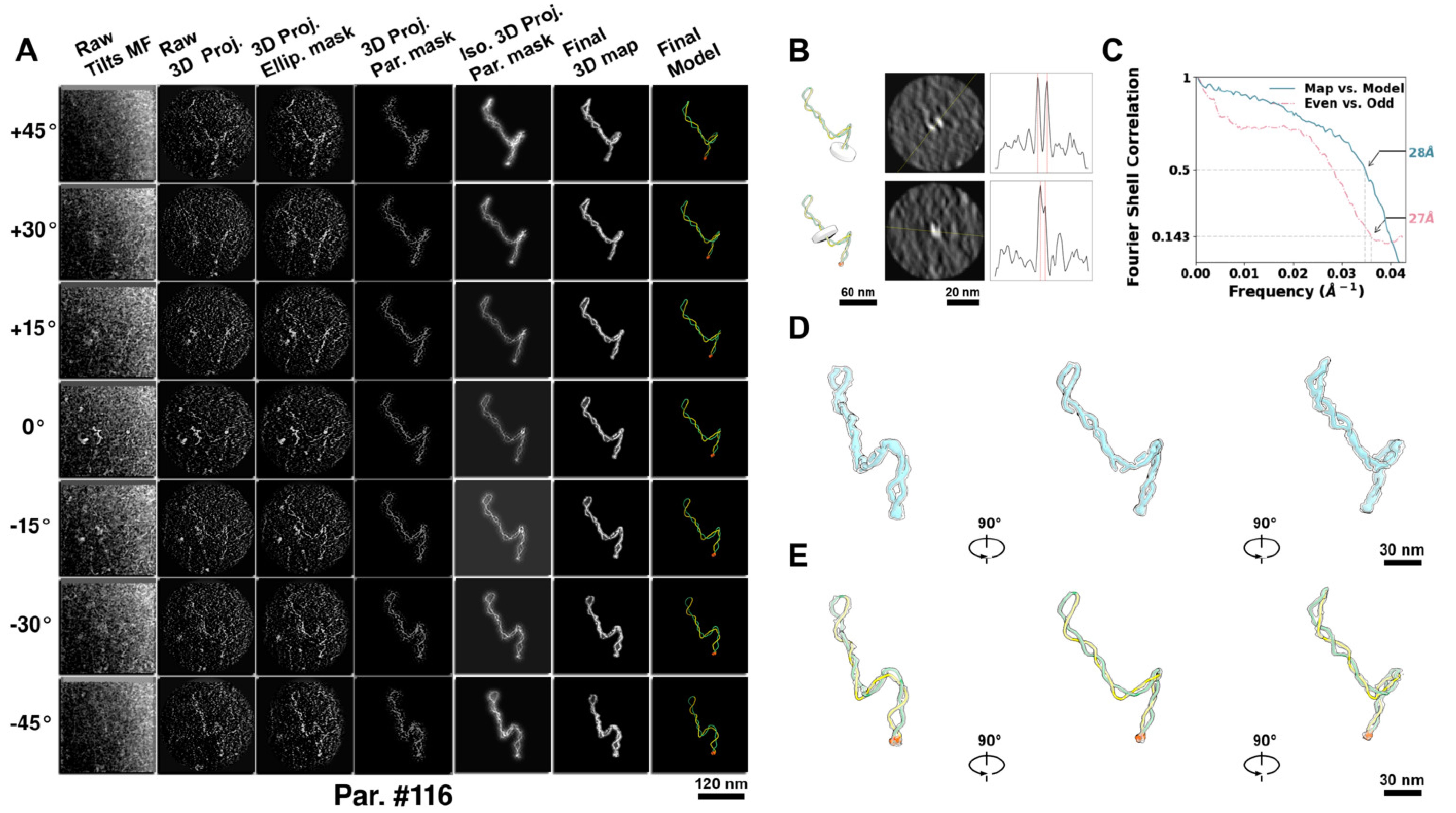

Supplementary Figure 116. Cryo-electron tomography (cryo-ET) 3D reconstruction of an individual plasmid particle in 40 mM K⁺, 5 mM Mg^{2+} in the presence of dCas9.
(A) 3D reconstruction of the plasmid particle (index no. 116). The first column shows seven representative tilt images from –45° to +45° in step of 15°. The second, third, and fourth columns show 3D projections of the particle with spherical, ellipsoidal (thinner along the z-dimension), and particle-shaped masks, respectively. The fifth column displays the 3D projections of the enhanced and IsoNet missing-wedge-corrected particle. The sixth and seventh columns present the final 3D map and the flexibly fitted model, respectively. (B) Two random cross-sectional views (12 nm thickness) of the plasmid density map along its plectoneme axis are shown in the left-middle panel. The intensity profile along the line crossing the two high-density DNA spots is displayed in the right panel. (C) Resolution assessment of the final 3D map using Fourier shell correlation (FSC). Two criteria are shown: FSC between two half-maps reconstructed from even and odd frames (evaluated at 0.143) and FSC between the final 3D map and the fitted model (evaluated at 0.5). (D) Zoomed-in views of the final 3D density map from panel A, displayed at two contour levels. (E) Superimposition of the high-contour level map from panel D onto its flexibly fitted model.

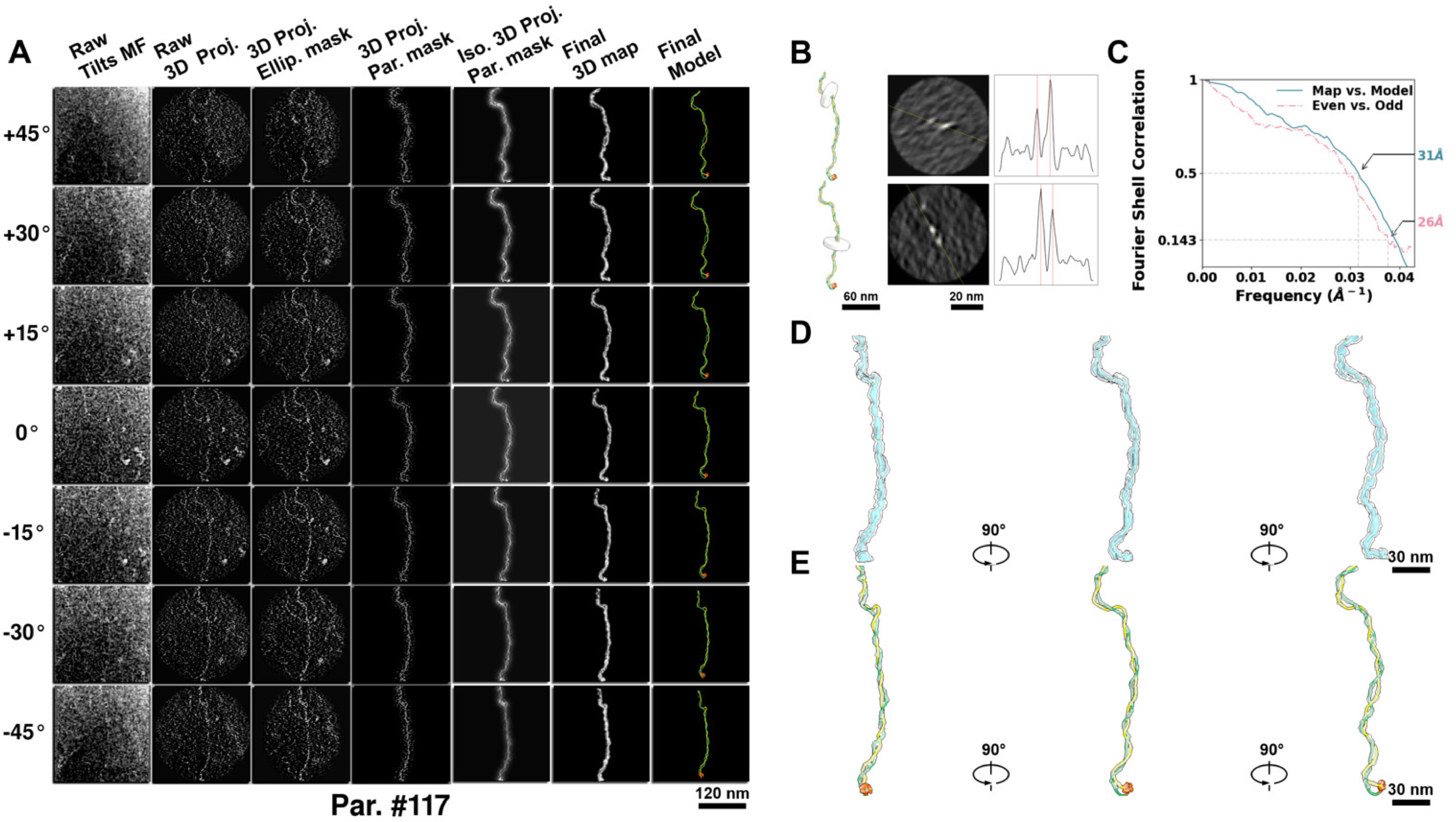

Supplementary Figure 117. Cryo-electron tomography (cryo-ET) 3D reconstruction of an individual plasmid particle in 40 mM K^+, 5 mM Mg^{2+} in the presence of dCas9.
(A) 3D reconstruction of the plasmid particle (index no. 117). The first column shows seven representative tilt images from –45° to +45° in step of 15°. The second, third, and fourth columns show 3D projections of the particle with spherical, ellipsoidal (thinner along the z-dimension), and particle-shaped masks, respectively. The fifth column displays the 3D projections of the enhanced and IsoNet missing-wedge-corrected particle. The sixth and seventh columns present the final 3D map and the flexibly fitted model, respectively. (B) Two random cross-sectional views (12 nm thickness) of the plasmid density map along its plectoneme axis are shown in the left-middle panel. The intensity profile along the line crossing the two high-density DNA spots is displayed in the right panel. (C) Resolution assessment of the final 3D map using Fourier shell correlation (FSC). Two criteria are shown: FSC between two half-maps reconstructed from even and odd frames (evaluated at 0.143) and FSC between the final 3D map and the fitted model (evaluated at 0.5). (D) Zoomed-in views of the final 3D density map from panel A, displayed at two contour levels. (E) Superimposition of the high-contour level map from panel D onto its flexibly fitted model.

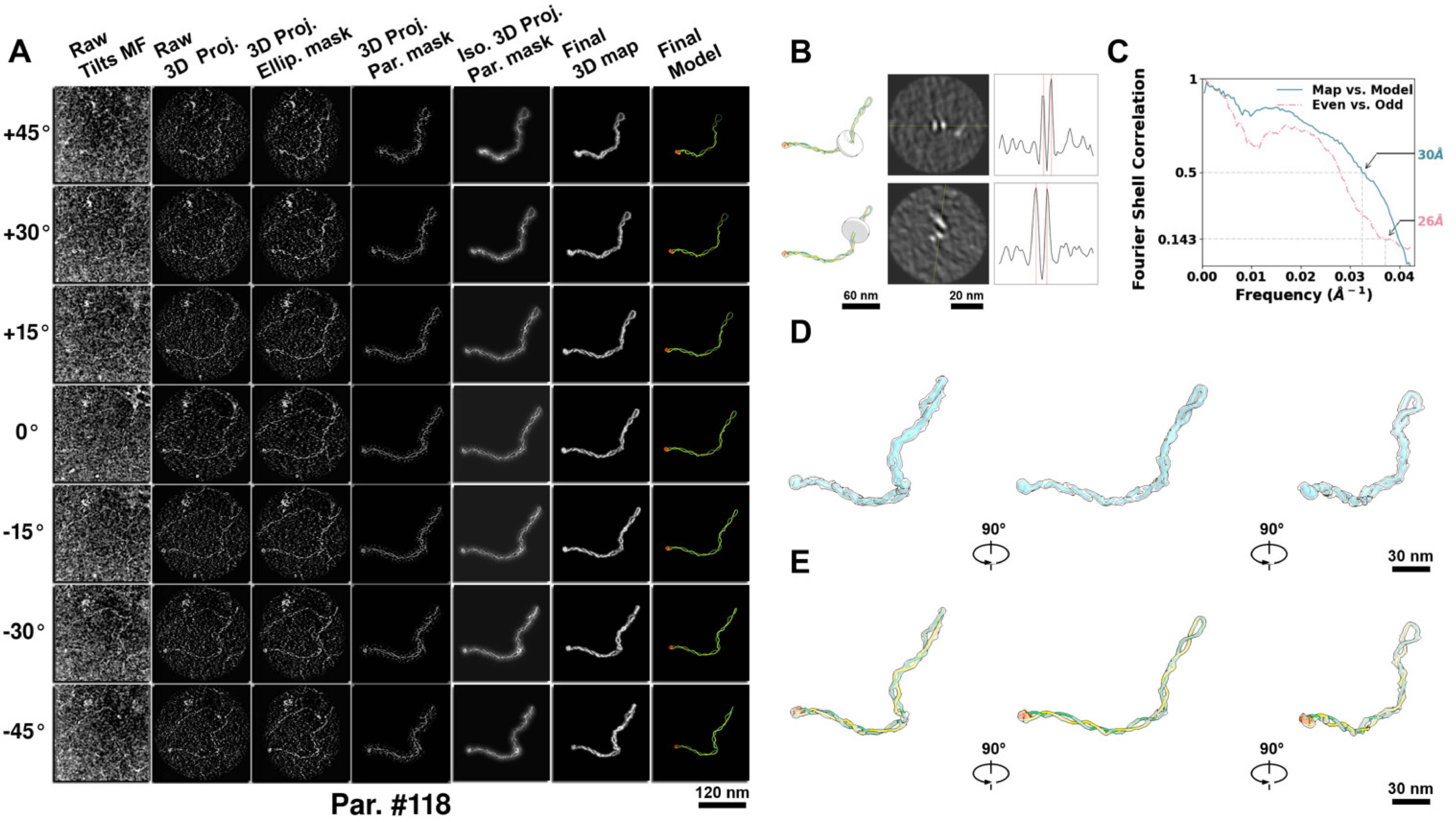

Supplementary Figure 118. Cryo-electron tomography (cryo-ET) 3D reconstruction of an individual plasmid particle in 40 mM K^+, 5 mM Mg^{2+} in the presence of dCas9.
(A) 3D reconstruction of the plasmid particle (index no. 118). The first column shows seven representative tilt images from –45° to +45° in step of 15°. The second, third, and fourth columns show 3D projections of the particle with spherical, ellipsoidal (thinner along the z-dimension), and particle-shaped masks, respectively. The fifth column displays the 3D projections of the enhanced and IsoNet missing-wedge-corrected particle. The sixth and seventh columns present the final 3D map and the flexibly fitted model, respectively. (B) Two random cross-sectional views (12 nm thickness) of the plasmid density map along its plectoneme axis are shown in the left-middle panel. The intensity profile along the line crossing the two high-density DNA spots is displayed in the right panel. (C) Resolution assessment of the final 3D map using Fourier shell correlation (FSC). Two criteria are shown: FSC between two half-maps reconstructed from even and odd frames (evaluated at 0.143) and FSC between the final 3D map and the fitted model (evaluated at 0.5). (D) Zoomed-in views of the final 3D density map from panel A, displayed at two contour levels. (E) Superimposition of the high-contour level map from panel D onto its flexibly fitted model.

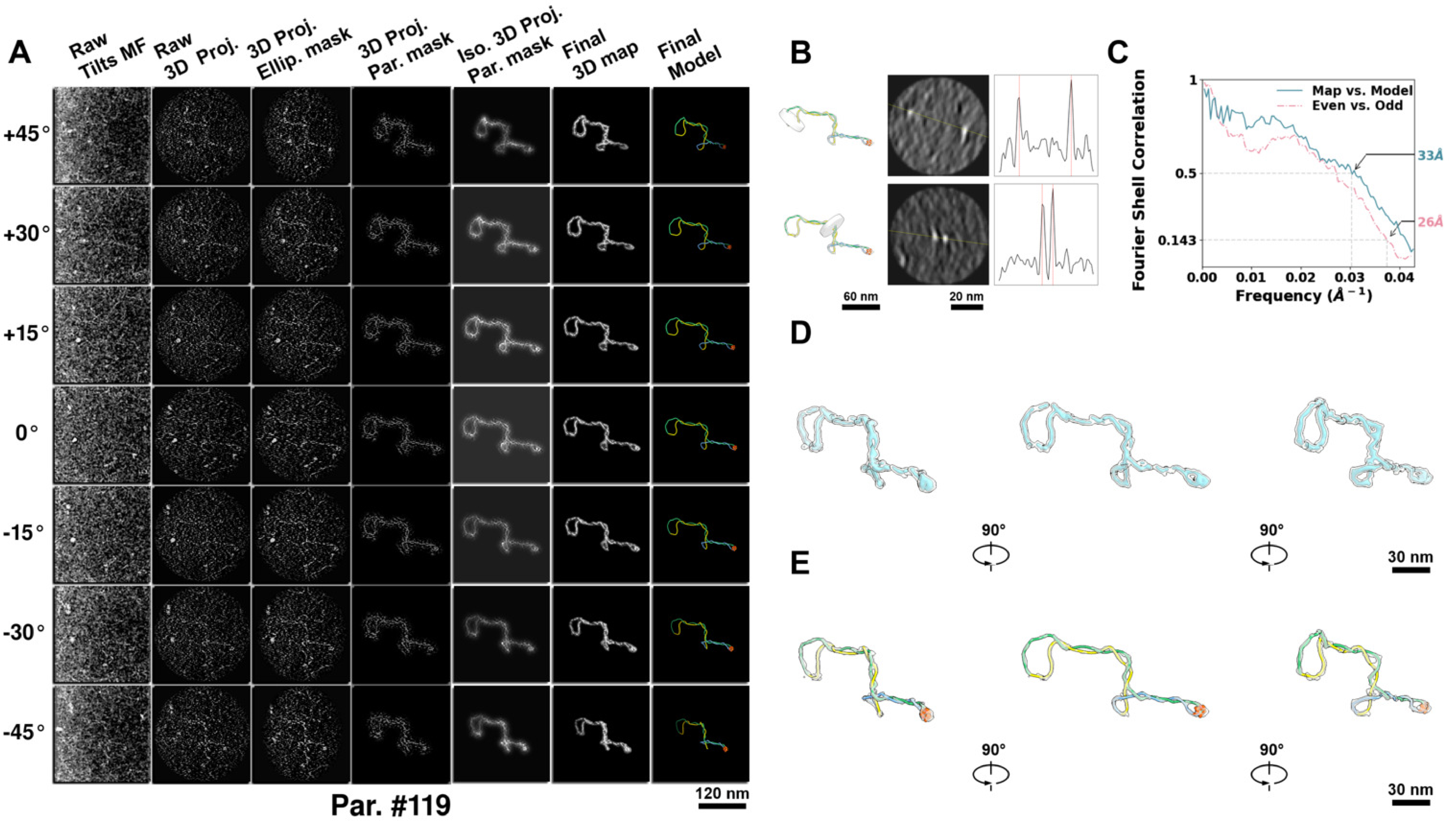

Supplementary Figure 119. Cryo-electron tomography (cryo-ET) 3D reconstruction of an individual plasmid particle in 40 mM K^{+}, 5 mM Mg^{2+} in the presence of dCas9.
(A) 3D reconstruction of the plasmid particle (index no. 119). The first column shows seven representative tilt images from –45° to +45° in step of 15°. The second, third, and fourth columns show 3D projections of the particle with spherical, ellipsoidal (thinner along the z-dimension), and particle-shaped masks, respectively. The fifth column displays the 3D projections of the enhanced and IsoNet missing-wedge-corrected particle. The sixth and seventh columns present the final 3D map and the flexibly fitted model, respectively. (B) Two random cross-sectional views (12 nm thickness) of the plasmid density map along its plectoneme axis are shown in the left-middle panel. The intensity profile along the line crossing the two high-density DNA spots is displayed in the right panel. (C) Resolution assessment of the final 3D map using Fourier shell correlation (FSC). Two criteria are shown: FSC between two half-maps reconstructed from even and odd frames (evaluated at 0.143) and FSC between the final 3D map and the fitted model (evaluated at 0.5). (D) Zoomed-in views of the final 3D density map from panel A, displayed at two contour levels. (E) Superimposition of the high-contour level map from panel D onto its flexibly fitted model.

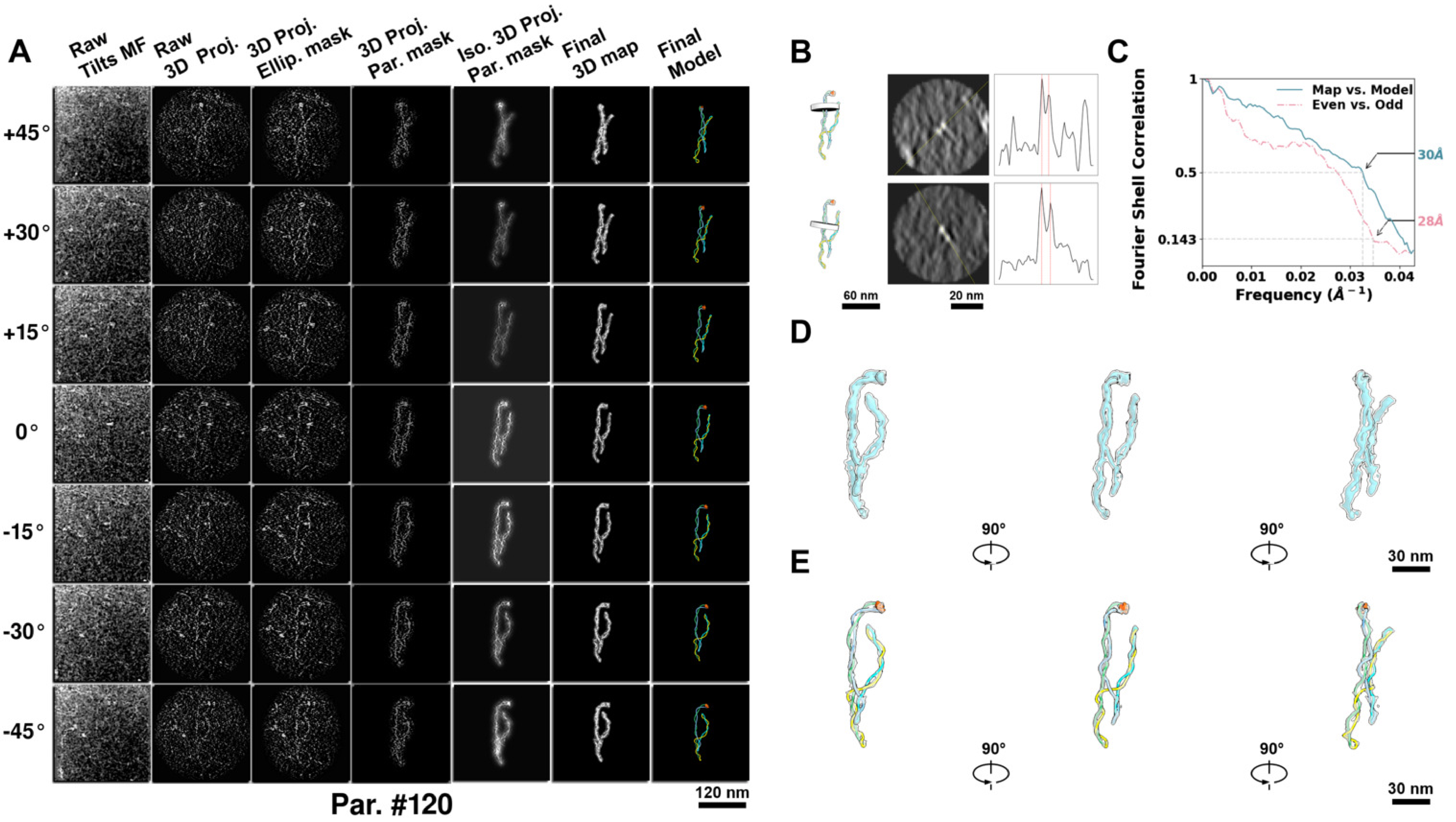

Supplementary Figure 120. Cryo-electron tomography (cryo-ET) 3D reconstruction of an individual plasmid particle in 40 mM K^{+}, 5 mM Mg^{2+} in the presence of dCas9.
(A) 3D reconstruction of the plasmid particle (index no. 120). The first column shows seven representative tilt images from –45° to +45° in step of 15°. The second, third, and fourth columns show 3D projections of the particle with spherical, ellipsoidal (thinner along the z-dimension), and particle-shaped masks, respectively. The fifth column displays the 3D projections of the enhanced and IsoNet missing-wedge-corrected particle. The sixth and seventh columns present the final 3D map and the flexibly fitted model, respectively. (B) Two random cross-sectional views (12 nm thickness) of the plasmid density map along its plectoneme axis are shown in the left-middle panel. The intensity profile along the line crossing the two high-density DNA spots is displayed in the right panel. (C) Resolution assessment of the final 3D map using Fourier shell correlation (FSC). Two criteria are shown: FSC between two half-maps reconstructed from even and odd frames (evaluated at 0.143) and FSC between the final 3D map and the fitted model (evaluated at 0.5). (D) Zoomed-in views of the final 3D density map from panel A, displayed at two contour levels. (E) Superimposition of the high-contour level map from panel D onto its flexibly fitted model.

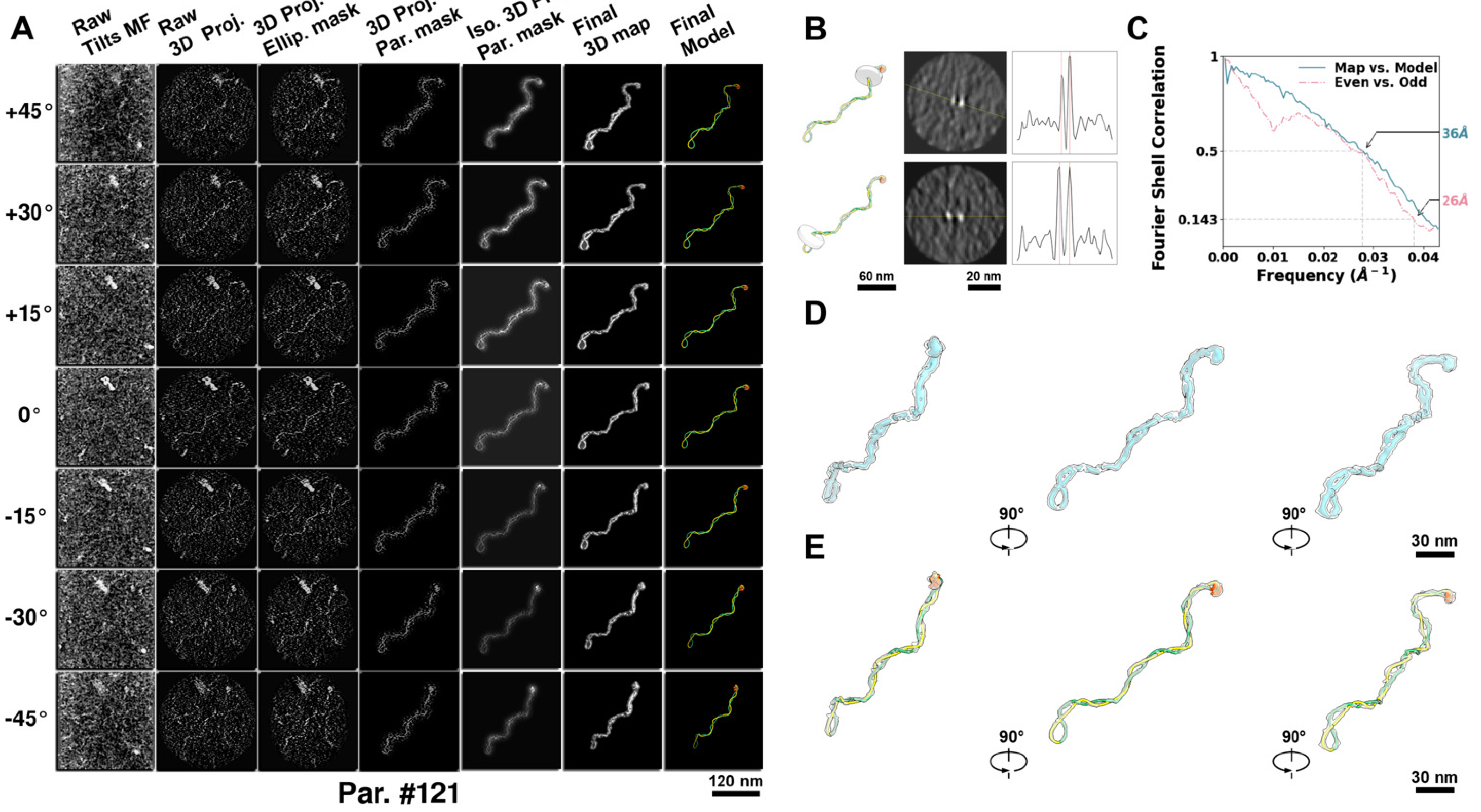

Supplementary Figure 121. Cryo-electron tomography (cryo-ET) 3D reconstruction of an individual plasmid particle in 40 mM K^+, 5 mM Mg^{2+} in the presence of dCas9.
(A) 3D reconstruction of the plasmid particle (index no. 121). The first column shows seven representative tilt images from –45° to +45° in step of 15°. The second, third, and fourth columns show 3D projections of the particle with spherical, ellipsoidal (thinner along the z-dimension), and particle-shaped masks, respectively. The fifth column displays the 3D projections of the enhanced and IsoNet missing-wedge-corrected particle. The sixth and seventh columns present the final 3D map and the flexibly fitted model, respectively. (B) Two random cross-sectional views (12 nm thickness) of the plasmid density map along its plectoneme axis are shown in the left-middle panel. The intensity profile along the line crossing the two high-density DNA spots is displayed in the right panel. (C) Resolution assessment of the final 3D map using Fourier shell correlation (FSC). Two criteria are shown: FSC between two half-maps reconstructed from even and odd frames (evaluated at 0.143) and FSC between the final 3D map and the fitted model (evaluated at 0.5). (D) Zoomed-in views of the final 3D density map from panel A, displayed at two contour levels. (E) Superimposition of the high-contour level map from panel D onto its flexibly fitted model.

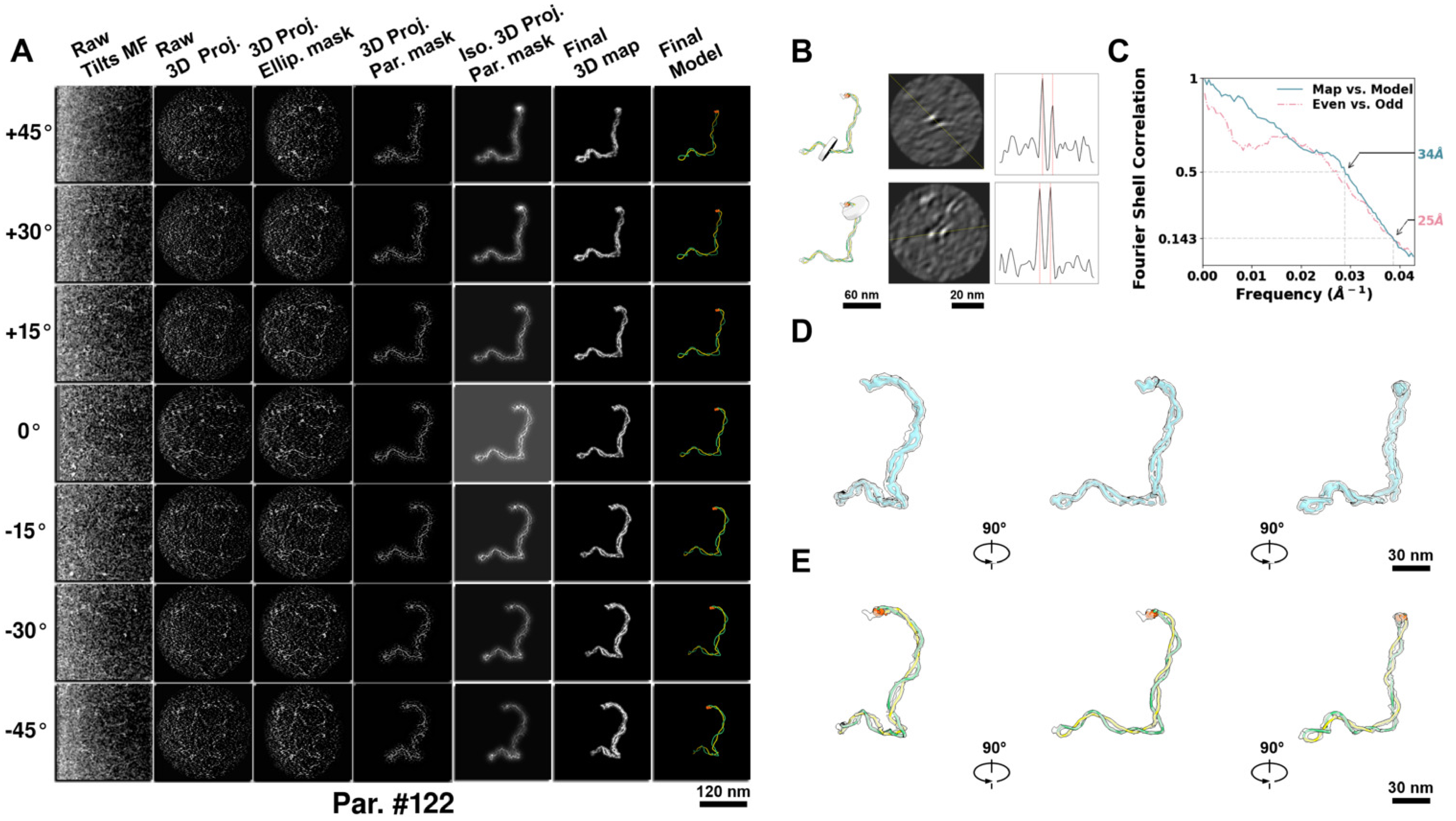

Supplementary Figure 122. Cryo-electron tomography (cryo-ET) 3D reconstruction of an individual plasmid particle in 40 mM K^+, 5 mM Mg^{2+} in the presence of dCas9.
(A) 3D reconstruction of the plasmid particle (index no. 122). The first column shows seven representative tilt images from –45° to +45° in step of 15°. The second, third, and fourth columns show 3D projections of the particle with spherical, ellipsoidal (thinner along the z-dimension), and particle-shaped masks, respectively. The fifth column displays the 3D projections of the enhanced and IsoNet missing-wedge-corrected particle. The sixth and seventh columns present the final 3D map and the flexibly fitted model, respectively. (B) Two random cross-sectional views (12 nm thickness) of the plasmid density map along its plectoneme axis are shown in the left-middle panel. The intensity profile along the line crossing the two high-density DNA spots is displayed in the right panel. (C) Resolution assessment of the final 3D map using Fourier shell correlation (FSC). Two criteria are shown: FSC between two half-maps reconstructed from even and odd frames (evaluated at 0.143) and FSC between the final 3D map and the fitted model (evaluated at 0.5). (D) Zoomed-in views of the final 3D density map from panel A, displayed at two contour levels. (E) Superimposition of the high-contour level map from panel D onto its flexibly fitted model.

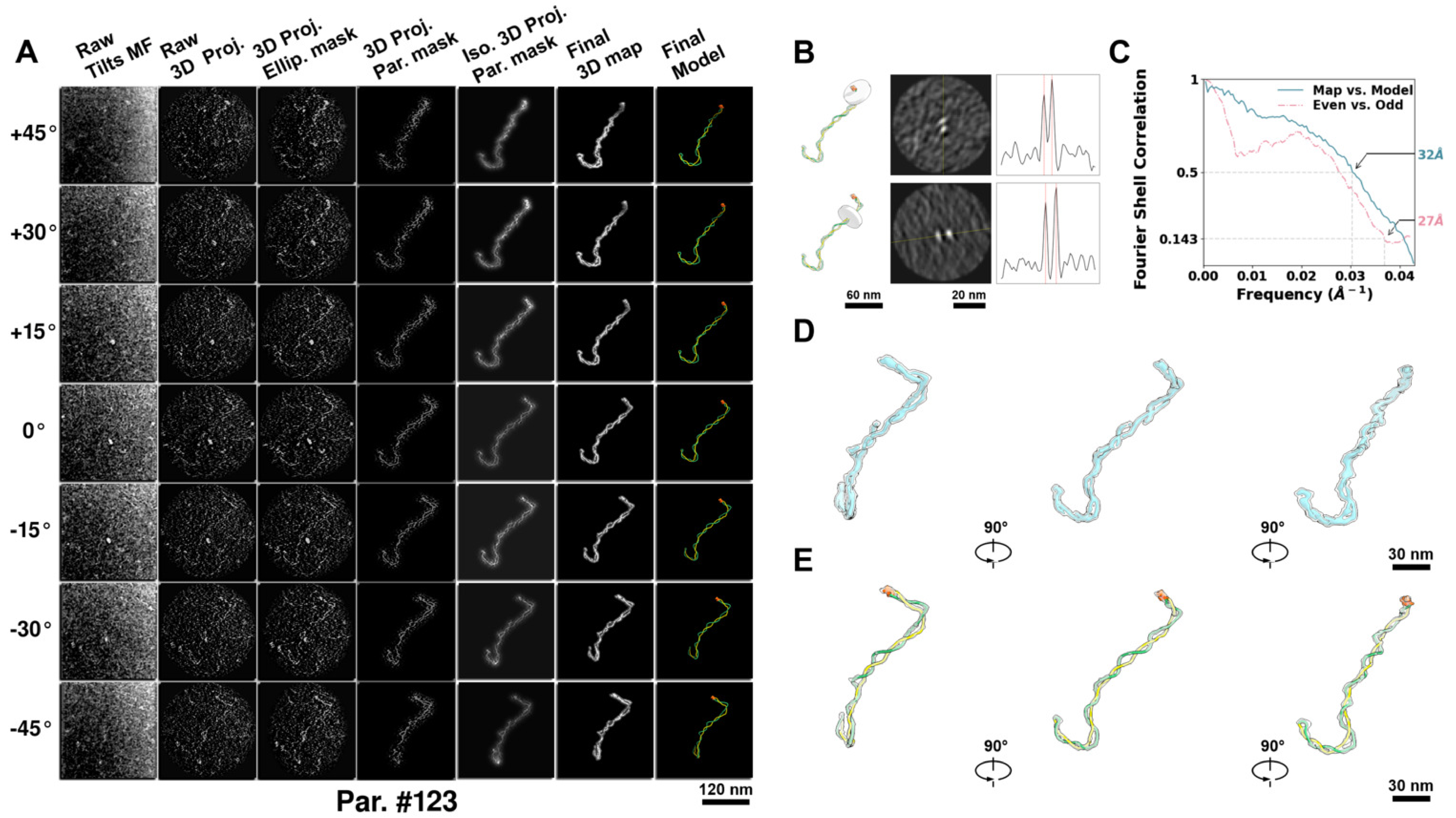

Supplementary Figure 123. Cryo-electron tomography (cryo-ET) 3D reconstruction of an individual plasmid particle in 40 mM K$^+$, 5 mM Mg^{2+} in the presence of dCas9.
(A) 3D reconstruction of the plasmid particle (index no. 123). The first column shows seven representative tilt images from –45° to +45° in step of 15°. The second, third, and fourth columns show 3D projections of the particle with spherical, ellipsoidal (thinner along the z-dimension), and particle-shaped masks, respectively. The fifth column displays the 3D projections of the enhanced and IsoNet missing-wedge-corrected particle. The sixth and seventh columns present the final 3D map and the flexibly fitted model, respectively. (B) Two random cross-sectional views (12 nm thickness) of the plasmid density map along its plectoneme axis are shown in the left-middle panel. The intensity profile along the line crossing the two high-density DNA spots is displayed in the right panel. (C) Resolution assessment of the final 3D map using Fourier shell correlation (FSC). Two criteria are shown: FSC between two half-maps reconstructed from even and odd frames (evaluated at 0.143) and FSC between the final 3D map and the fitted model (evaluated at 0.5). (D) Zoomed-in views of the final 3D density map from panel A, displayed at two contour levels. (E) Superimposition of the high-contour level map from panel D onto its flexibly fitted model.

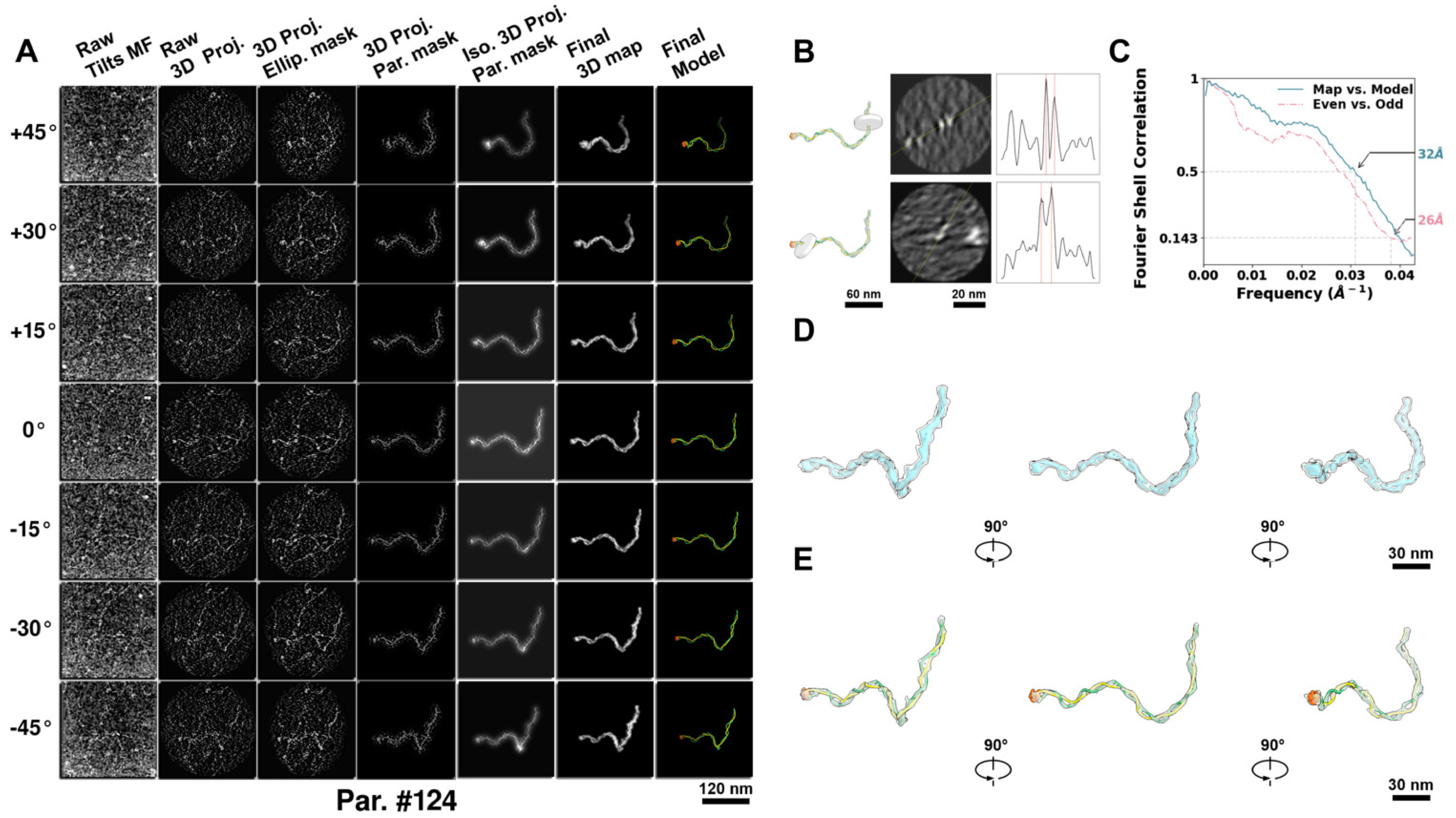

Supplementary Figure 124. Cryo-electron tomography (cryo-ET) 3D reconstruction of an individual plasmid particle in 40 mM K$^+$, 5 mM Mg^{2+} in the presence of dCas9.
(A) 3D reconstruction of the plasmid particle (index no. 124). The first column shows seven representative tilt images from –45° to +45° in step of 15°. The second, third, and fourth columns show 3D projections of the particle with spherical, ellipsoidal (thinner along the z-dimension), and particle-shaped masks, respectively. The fifth column displays the 3D projections of the enhanced and IsoNet missing-wedge-corrected particle. The sixth and seventh columns present the final 3D map and the flexibly fitted model, respectively. (B) Two random cross-sectional views (12 nm thickness) of the plasmid density map along its plectoneme axis are shown in the left-middle panel. The intensity profile along the line crossing the two high-density DNA spots is displayed in the right panel. (C) Resolution assessment of the final 3D map using Fourier shell correlation (FSC). Two criteria are shown: FSC between two half-maps reconstructed from even and odd frames (evaluated at 0.143) and FSC between the final 3D map and the fitted model (evaluated at 0.5). (D) Zoomed-in views of the final 3D density map from panel A, displayed at two contour levels. (E) Superimposition of the high-contour level map from panel D onto its flexibly fitted model.

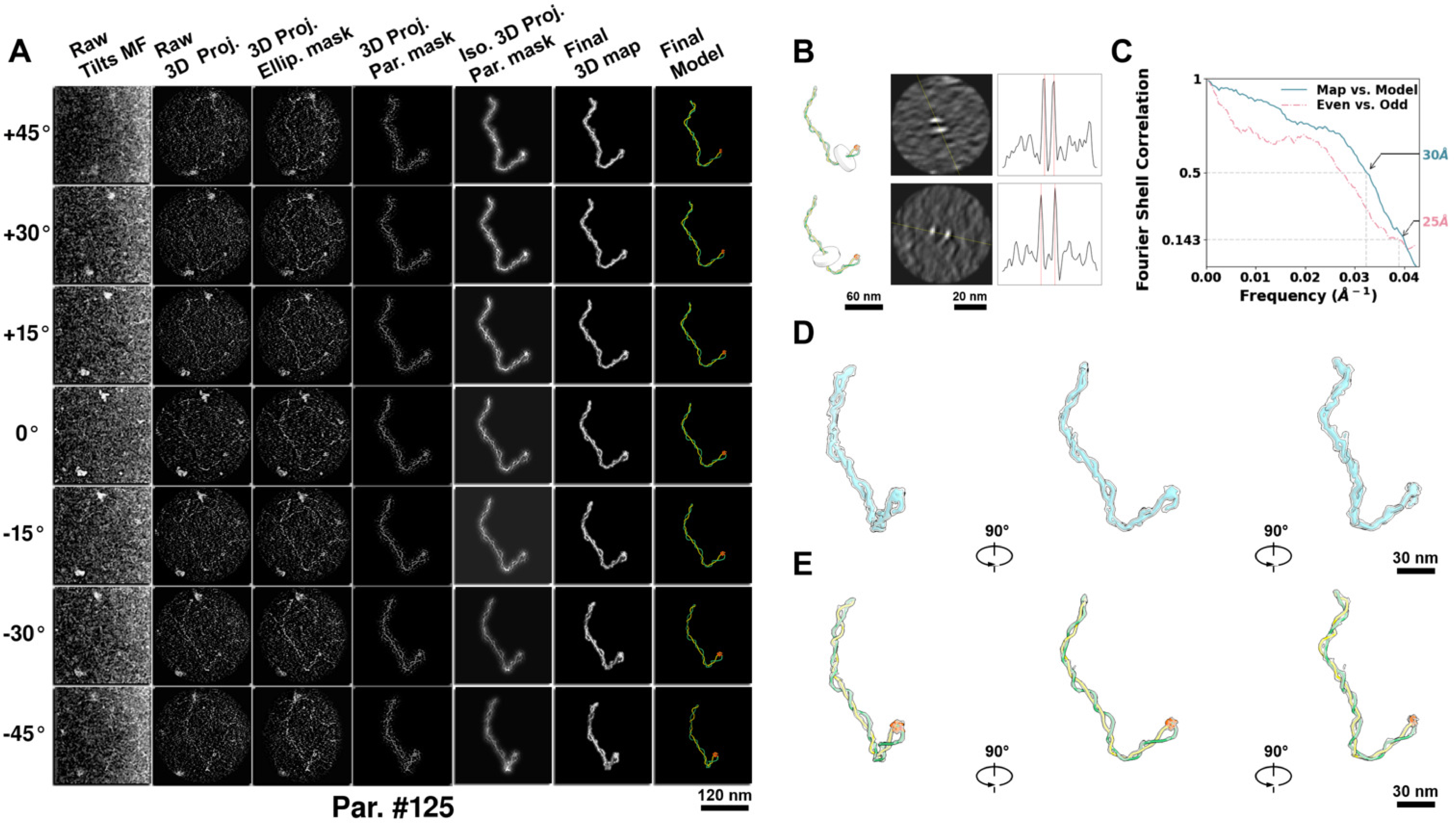

Supplementary Figure 125. Cryo-electron tomography (cryo-ET) 3D reconstruction of an individual plasmid particle in 40 mM K⁺, 5 mM Mg²⁺ in the presence of dCas9.
(A) 3D reconstruction of the plasmid particle (index no. 125). The first column shows seven representative tilt images from –45° to +45° in step of 15°. The second, third, and fourth columns show 3D projections of the particle with spherical, ellipsoidal (thinner along the z-dimension), and particle-shaped masks, respectively. The fifth column displays the 3D projections of the enhanced and IsoNet missing-wedge-corrected particle. The sixth and seventh columns present the final 3D map and the flexibly fitted model, respectively. (B) Two random cross-sectional views (12 nm thickness) of the plasmid density map along its plectoneme axis are shown in the left-middle panel. The intensity profile along the line crossing the two high-density DNA spots is displayed in the right panel. (C) Resolution assessment of the final 3D map using Fourier shell correlation (FSC). Two criteria are shown: FSC between two half-maps reconstructed from even and odd frames (evaluated at 0.143) and FSC between the final 3D map and the fitted model (evaluated at 0.5). (D) Zoomed-in views of the final 3D density map from panel A, displayed at two contour levels. (E) Superimposition of the high-contour level map from panel D onto its flexibly fitted model.

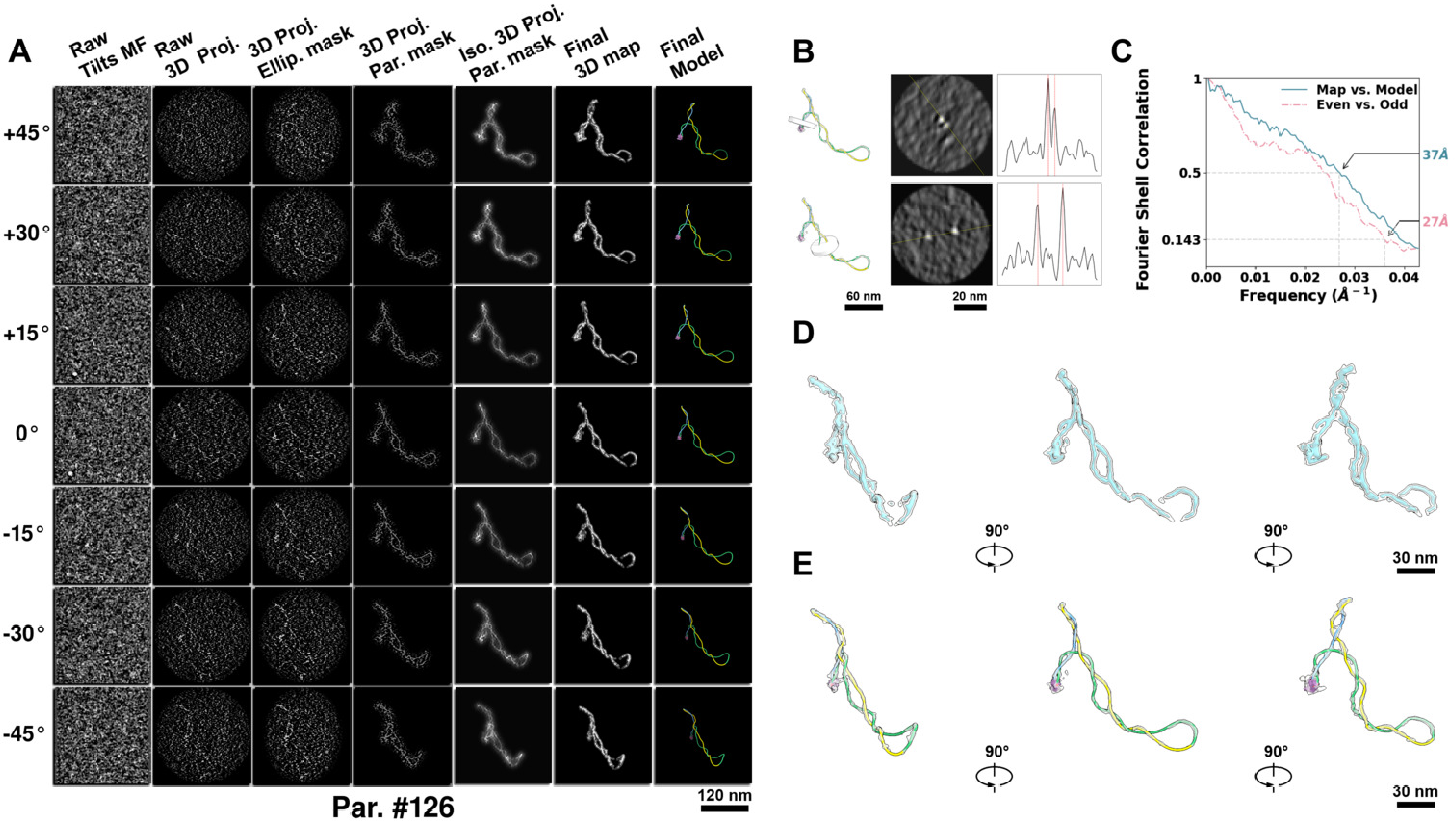

Supplementary Figure 126. Cryo-electron tomography (cryo-ET) 3D reconstruction of an individual plasmid particle in 40 mM K⁺, 5 mM Mg²⁺ in the presence of transcribed RNAP.
(A) 3D reconstruction of the plasmid particle (index no. 126). The first column shows seven representative tilt images from –45° to +45° in step of 15°. The second, third, and fourth columns show 3D projections of the particle with spherical, ellipsoidal (thinner along the z-dimension), and particle-shaped masks, respectively. The fifth column displays the 3D projections of the enhanced and IsoNet missing-wedge-corrected particle. The sixth and seventh columns present the final 3D map and the flexibly fitted model, respectively. (B) Two random cross-sectional views (12 nm thickness) of the plasmid density map along its plectoneme axis are shown in the left-middle panel. The intensity profile along the line crossing the two high-density DNA spots is displayed in the right panel. (C) Resolution assessment of the final 3D map using Fourier shell correlation (FSC). Two criteria are shown: FSC between two half-maps reconstructed from even and odd frames (evaluated at 0.143) and FSC between the final 3D map and the fitted model (evaluated at 0.5). (D) Zoomed-in views of the final 3D density map from panel A, displayed at two contour levels. (E) Superimposition of the high-contour level map from panel D onto its flexibly fitted model.

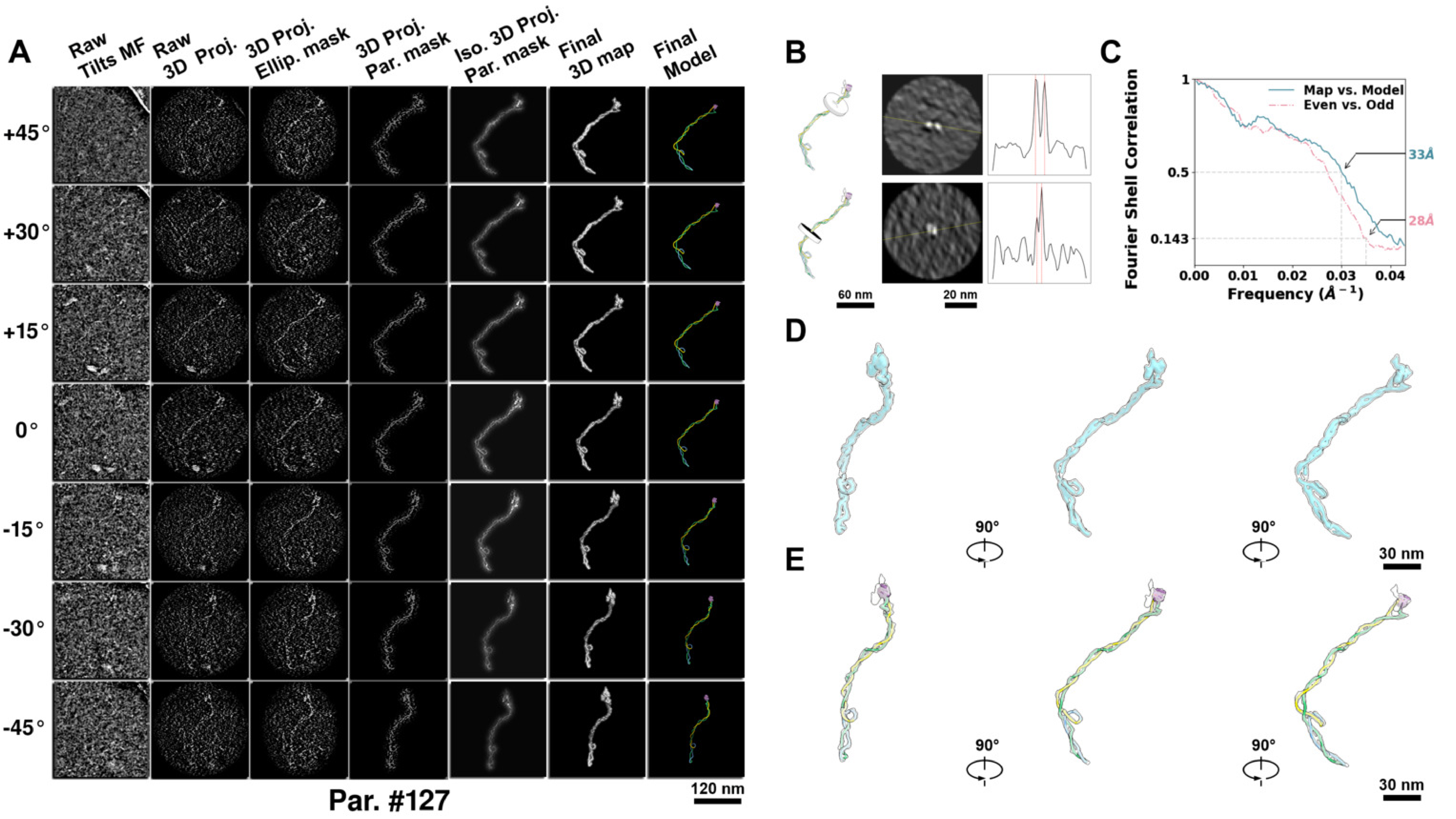

Supplementary Figure 127. Cryo-electron tomography (cryo-ET) 3D reconstruction of an individual plasmid particle in 40 mM K^+, 5 mM Mg^{2+} in the presence of transcribed RNAP.
(A) 3D reconstruction of the plasmid particle (index no. 127). The first column shows seven representative tilt images from –45° to +45° in step of 15°. The second, third, and fourth columns show 3D projections of the particle with spherical, ellipsoidal (thinner along the z-dimension), and particle-shaped masks, respectively. The fifth column displays the 3D projections of the enhanced and IsoNet missing-wedge-corrected particle. The sixth and seventh columns present the final 3D map and the flexibly fitted model, respectively. (B) Two random cross-sectional views (12 nm thickness) of the plasmid density map along its plectoneme axis are shown in the left-middle panel. The intensity profile along the line crossing the two high-density DNA spots is displayed in the right panel. (C) Resolution assessment of the final 3D map using Fourier shell correlation (FSC). Two criteria are shown: FSC between two half-maps reconstructed from even and odd frames (evaluated at 0.143) and FSC between the final 3D map and the fitted model (evaluated at 0.5). (D) Zoomed-in views of the final 3D density map from panel A, displayed at two contour levels. (E) Superimposition of the high-contour level map from panel D onto its flexibly fitted model.

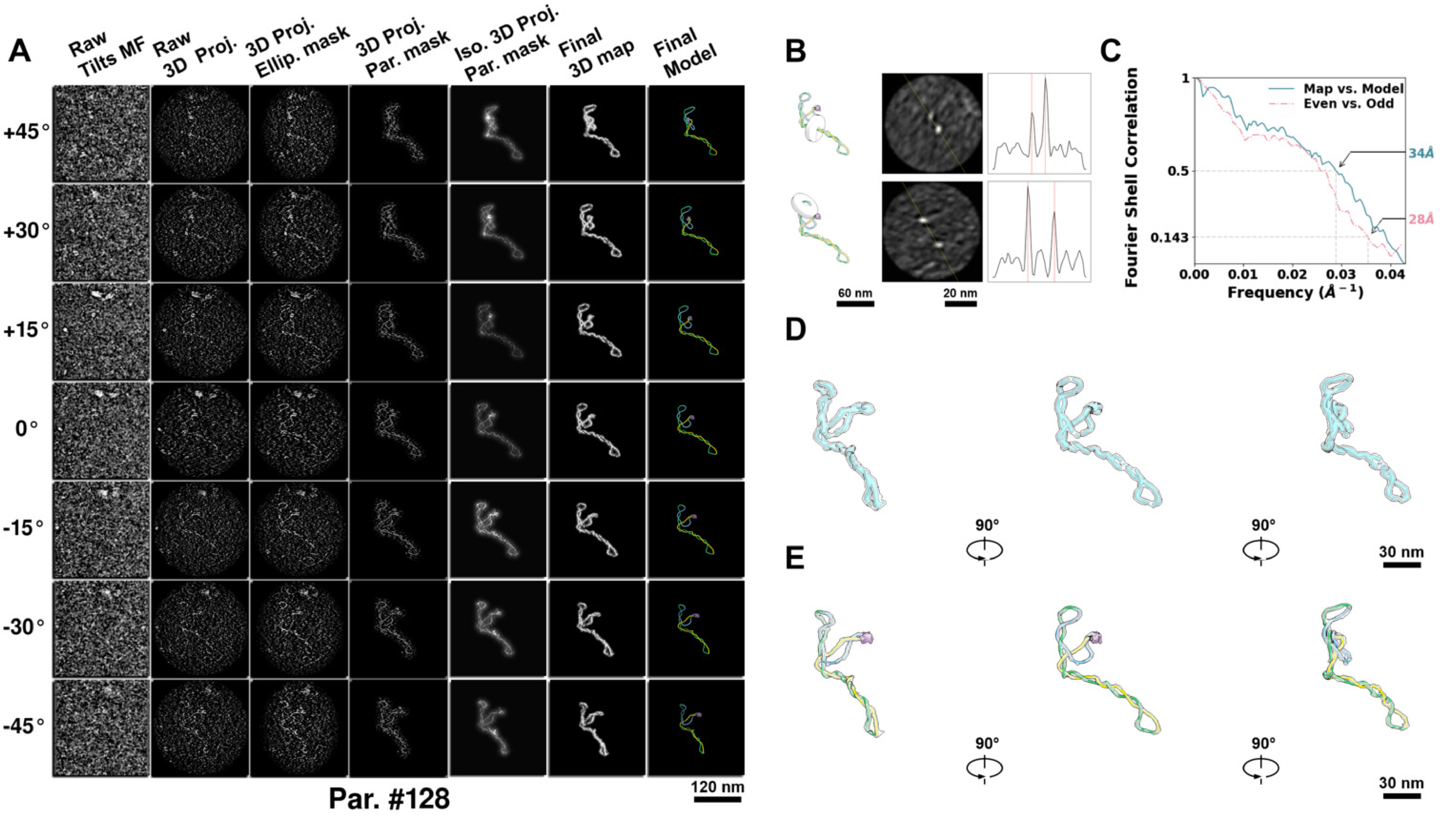

Supplementary Figure 128. Cryo-electron tomography (cryo-ET) 3D reconstruction of an individual plasmid particle in 40 mM K^+, 5 mM Mg^{2+} in the presence of transcribed RNAP.
(A) 3D reconstruction of the plasmid particle (index no. 128). The first column shows seven representative tilt images from –45° to +45° in step of 15°. The second, third, and fourth columns show 3D projections of the particle with spherical, ellipsoidal (thinner along the z-dimension), and particle-shaped masks, respectively. The fifth column displays the 3D projections of the enhanced and IsoNet missing-wedge-corrected particle. The sixth and seventh columns present the final 3D map and the flexibly fitted model, respectively. (B) Two random cross-sectional views (12 nm thickness) of the plasmid density map along its plectoneme axis are shown in the left-middle panel. The intensity profile along the line crossing the two high-density DNA spots is displayed in the right panel. (C) Resolution assessment of the final 3D map using Fourier shell correlation (FSC). Two criteria are shown: FSC between two half-maps reconstructed from even and odd frames (evaluated at 0.143) and FSC between the final 3D map and the fitted model (evaluated at 0.5). (D) Zoomed-in views of the final 3D density map from panel A, displayed at two contour levels. (E) Superimposition of the high-contour level map from panel D onto its flexibly fitted model.

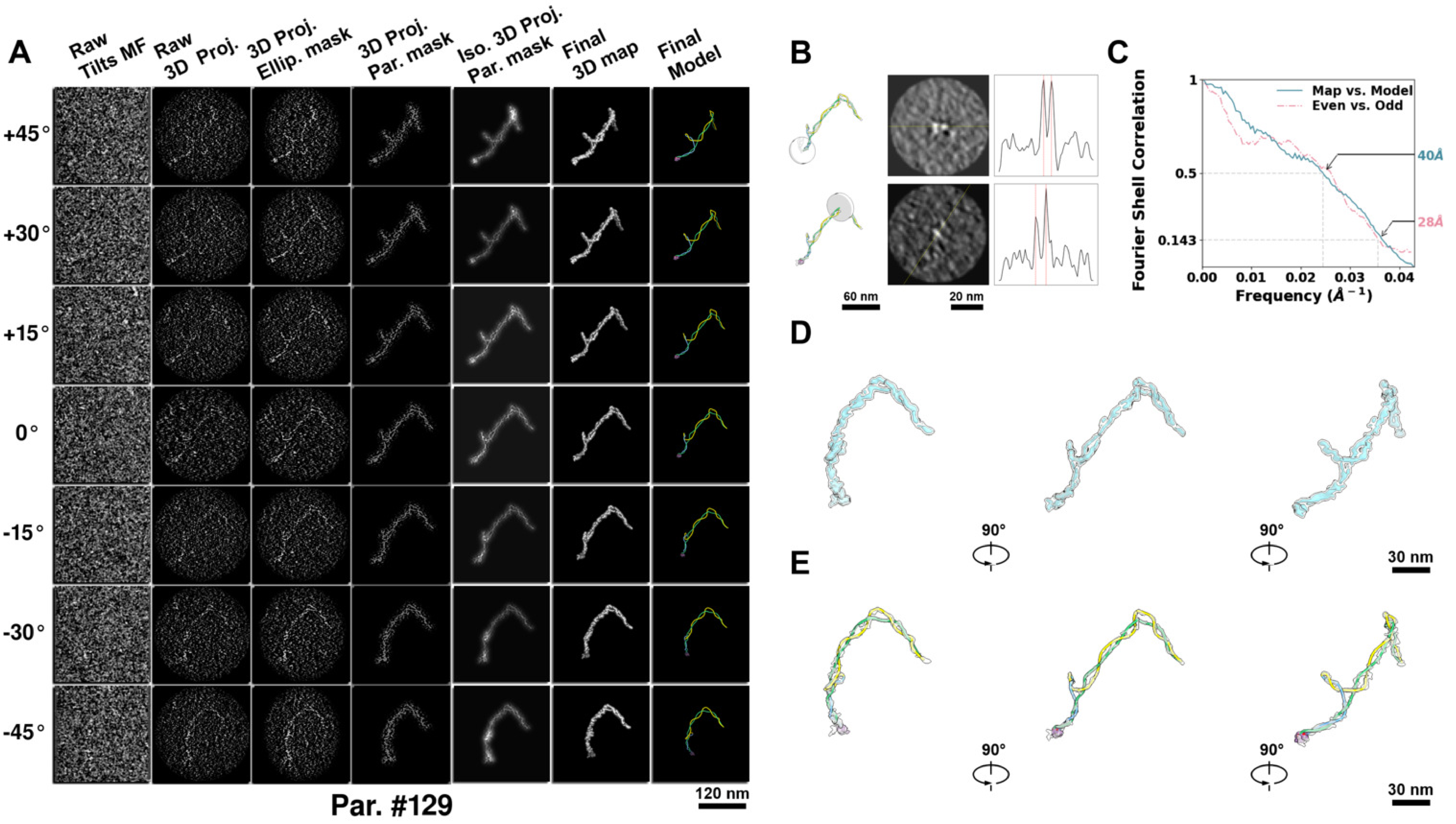

Supplementary Figure 129. Cryo-electron tomography (cryo-ET) 3D reconstruction of an individual plasmid particle in 40 mM K^+, 5 mM Mg^{2+} in the presence of transcribed RNAP.
(A) 3D reconstruction of the plasmid particle (index no. 129). The first column shows seven representative tilt images from –45° to +45° in step of 15°. The second, third, and fourth columns show 3D projections of the particle with spherical, ellipsoidal (thinner along the z-dimension), and particle-shaped masks, respectively. The fifth column displays the 3D projections of the enhanced and IsoNet missing-wedge-corrected particle. The sixth and seventh columns present the final 3D map and the flexibly fitted model, respectively. (B) Two random cross-sectional views (12 nm thickness) of the plasmid density map along its plectoneme axis are shown in the left-middle panel. The intensity profile along the line crossing the two high-density DNA spots is displayed in the right panel. (C) Resolution assessment of the final 3D map using Fourier shell correlation (FSC). Two criteria are shown: FSC between two half-maps reconstructed from even and odd frames (evaluated at 0.143) and FSC between the final 3D map and the fitted model (evaluated at 0.5). (D) Zoomed-in views of the final 3D density map from panel A, displayed at two contour levels. (E) Superimposition of the high-contour level map from panel D onto its flexibly fitted model.

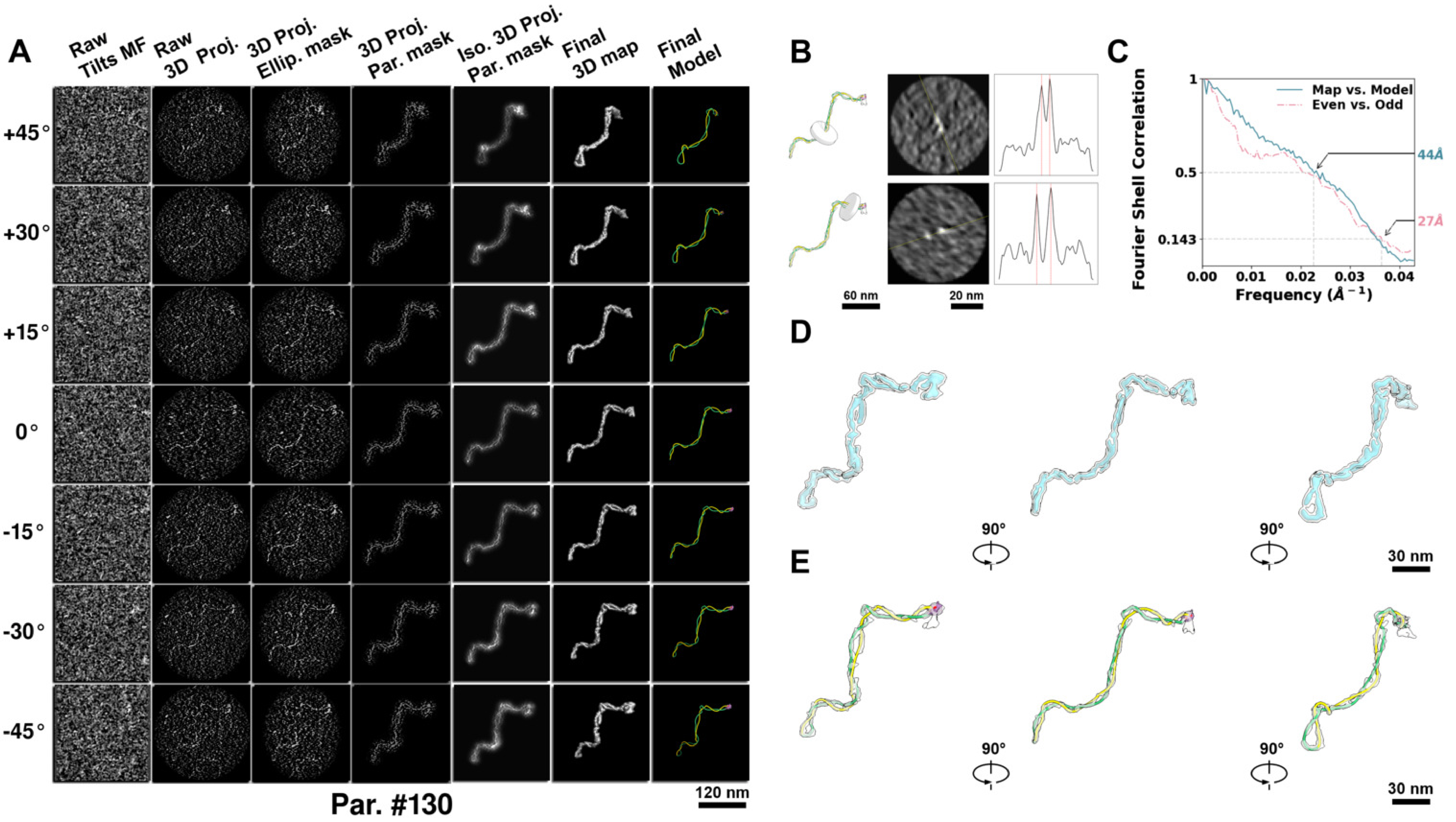

Supplementary Figure 130. Cryo-electron tomography (cryo-ET) 3D reconstruction of an individual plasmid particle in 40 mM K^+, 5 mM Mg^{2+} in the presence of transcribed RNAP.
(A) 3D reconstruction of the plasmid particle (index no. 130). The first column shows seven representative tilt images from –45° to +45° in step of 15°. The second, third, and fourth columns show 3D projections of the particle with spherical, ellipsoidal (thinner along the z-dimension), and particle-shaped masks, respectively. The fifth column displays the 3D projections of the enhanced and IsoNet missing-wedge-corrected particle. The sixth and seventh columns present the final 3D map and the flexibly fitted model, respectively. (B) Two random cross-sectional views (12 nm thickness) of the plasmid density map along its plectoneme axis are shown in the left-middle panel. The intensity profile along the line crossing the two high-density DNA spots is displayed in the right panel. (C) Resolution assessment of the final 3D map using Fourier shell correlation (FSC). Two criteria are shown: FSC between two half-maps reconstructed from even and odd frames (evaluated at 0.143) and FSC between the final 3D map and the fitted model (evaluated at 0.5). (D) Zoomed-in views of the final 3D density map from panel A, displayed at two contour levels. (E) Superimposition of the high-contour level map from panel D onto its flexibly fitted model.

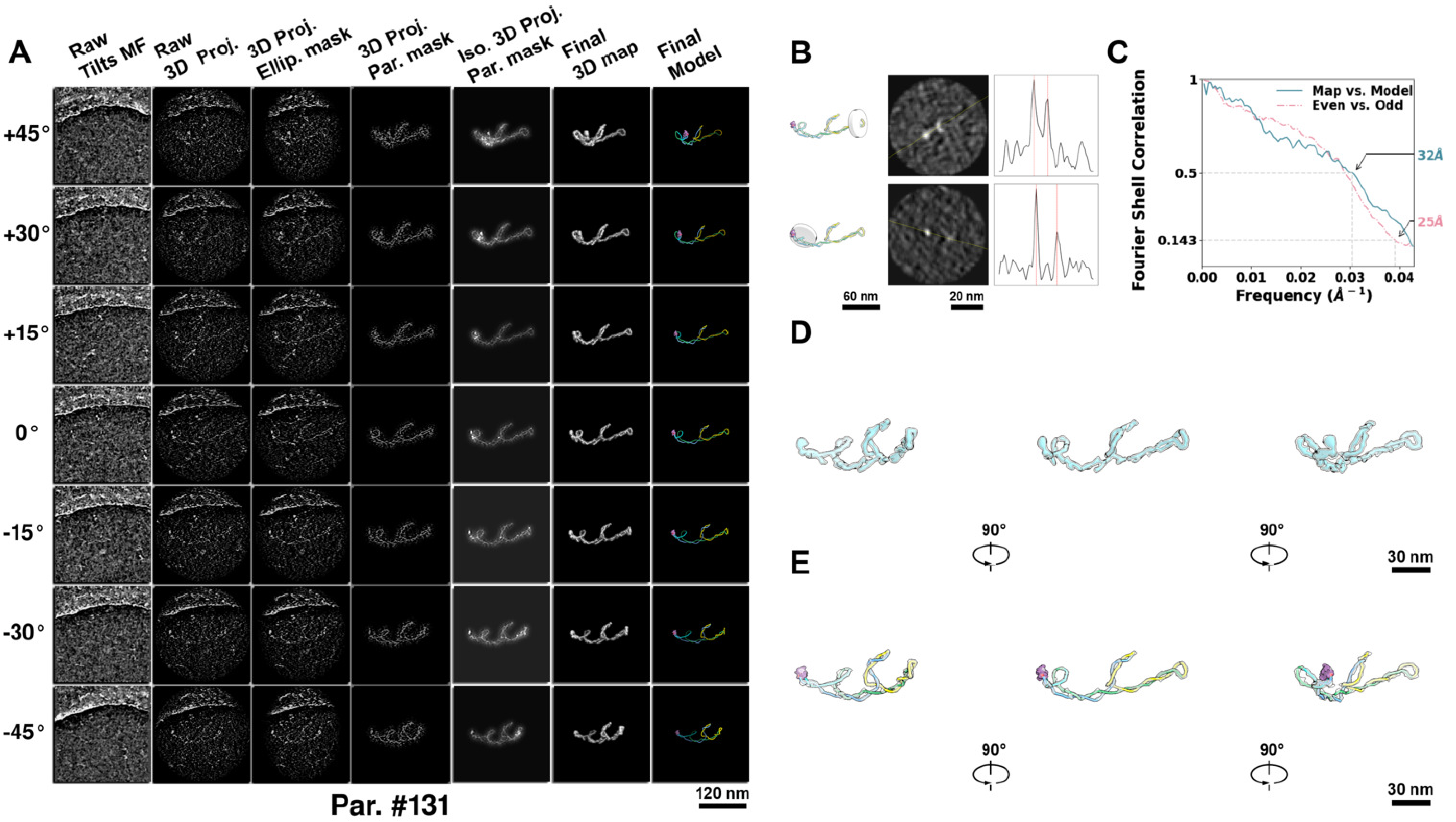

Supplementary Figure 131. Cryo-electron tomography (cryo-ET) 3D reconstruction of an individual plasmid particle in 40 mM K^+, 5 mM Mg^{2+} in the presence of transcribed RNAP.
(A) 3D reconstruction of the plasmid particle (index no. 131). The first column shows seven representative tilt images from –45° to +45° in step of 15°. The second, third, and fourth columns show 3D projections of the particle with spherical, ellipsoidal (thinner along the z-dimension), and particle-shaped masks, respectively. The fifth column displays the 3D projections of the enhanced and IsoNet missing-wedge-corrected particle. The sixth and seventh columns present the final 3D map and the flexibly fitted model, respectively. (B) Two random cross-sectional views (12 nm thickness) of the plasmid density map along its plectoneme axis are shown in the left-middle panel. The intensity profile along the line crossing the two high-density DNA spots is displayed in the right panel. (C) Resolution assessment of the final 3D map using Fourier shell correlation (FSC). Two criteria are shown: FSC between two half-maps reconstructed from even and odd frames (evaluated at 0.143) and FSC between the final 3D map and the fitted model (evaluated at 0.5). (D) Zoomed-in views of the final 3D density map from panel A, displayed at two contour levels. (E) Superimposition of the high-contour level map from panel D onto its flexibly fitted model.

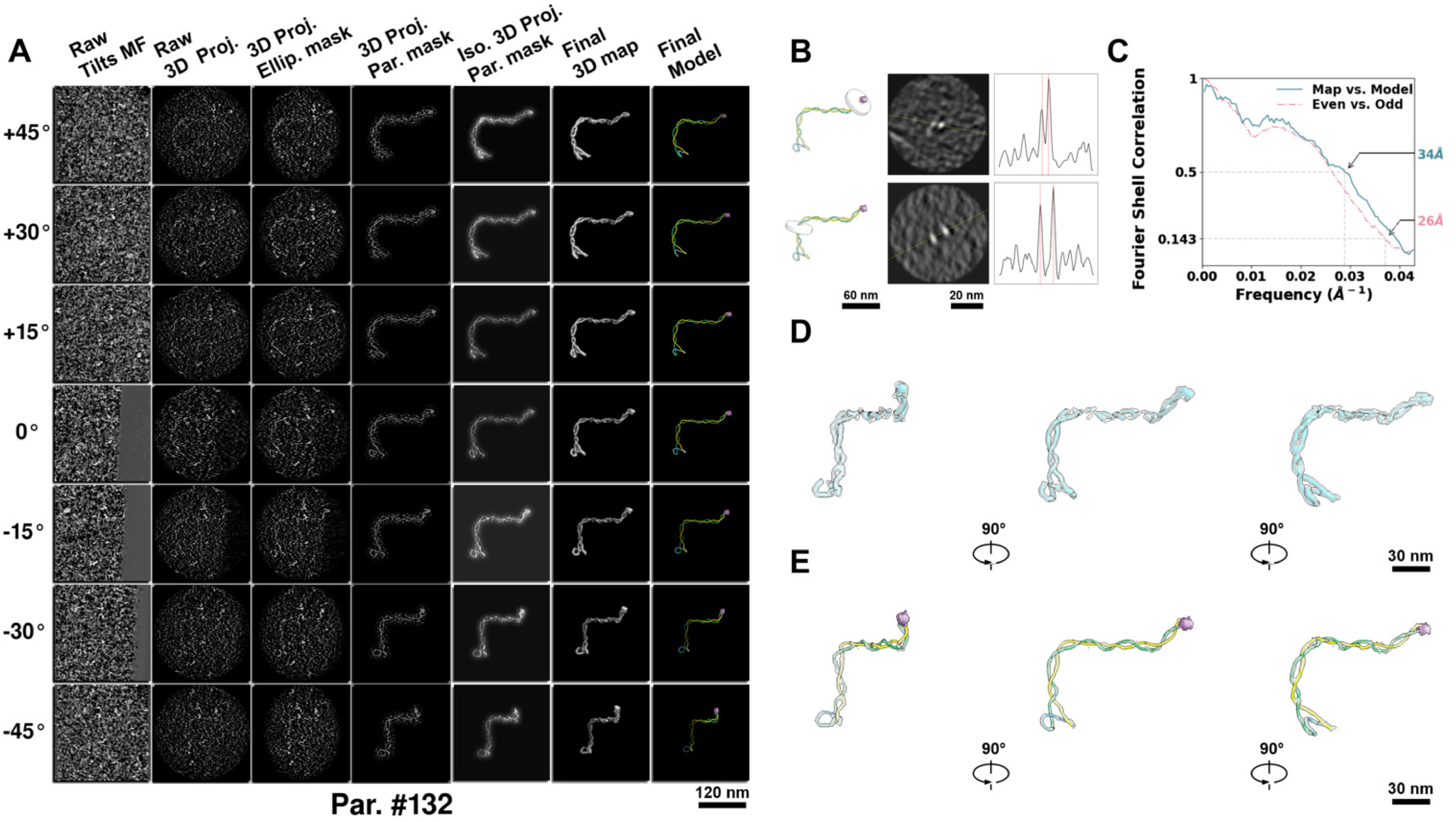

Supplementary Figure 132. Cryo-electron tomography (cryo-ET) 3D reconstruction of an individual plasmid particle in 40 mM K^+, 5 mM Mg^{2+} in the presence of transcribed RNAP.
(A) 3D reconstruction of the plasmid particle (index no. 132). The first column shows seven representative tilt images from –45° to +45° in step of 15°. The second, third, and fourth columns show 3D projections of the particle with spherical, ellipsoidal (thinner along the z-dimension), and particle-shaped masks, respectively. The fifth column displays the 3D projections of the enhanced and IsoNet missing-wedge-corrected particle. The sixth and seventh columns present the final 3D map and the flexibly fitted model, respectively. (B) Two random cross-sectional views (12 nm thickness) of the plasmid density map along its plectoneme axis are shown in the left-middle panel. The intensity profile along the line crossing the two high-density DNA spots is displayed in the right panel. (C) Resolution assessment of the final 3D map using Fourier shell correlation (FSC). Two criteria are shown: FSC between two half-maps reconstructed from even and odd frames (evaluated at 0.143) and FSC between the final 3D map and the fitted model (evaluated at 0.5). (D) Zoomed-in views of the final 3D density map from panel A, displayed at two contour levels. (E) Superimposition of the high-contour level map from panel D onto its flexibly fitted model.

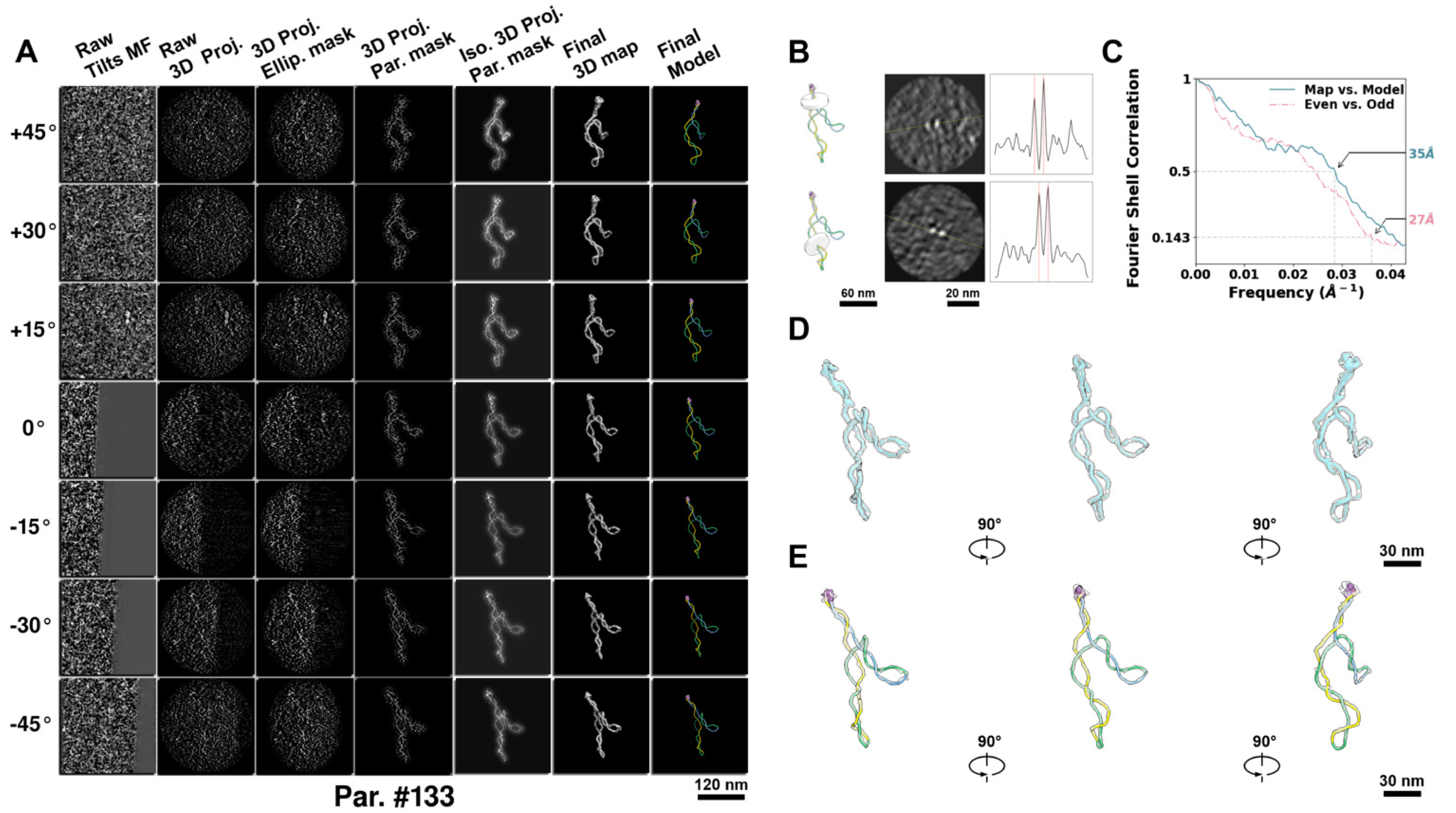

Supplementary Figure 133. Cryo-electron tomography (cryo-ET) 3D reconstruction of an individual plasmid particle in 40 mM K^{+}, 5 mM Mg^{2+} in the presence of transcribed RNAP.
(A) 3D reconstruction of the plasmid particle (index no. 133). The first column shows seven representative tilt images from –45° to +45° in step of 15°. The second, third, and fourth columns show 3D projections of the particle with spherical, ellipsoidal (thinner along the z-dimension), and particle-shaped masks, respectively. The fifth column displays the 3D projections of the enhanced and IsoNet missing-wedge-corrected particle. The sixth and seventh columns present the final 3D map and the flexibly fitted model, respectively. (B) Two random cross-sectional views (12 nm thickness) of the plasmid density map along its plectoneme axis are shown in the left-middle panel. The intensity profile along the line crossing the two high-density DNA spots is displayed in the right panel. (C) Resolution assessment of the final 3D map using Fourier shell correlation (FSC). Two criteria are shown: FSC between two half-maps reconstructed from even and odd frames (evaluated at 0.143) and FSC between the final 3D map and the fitted model (evaluated at 0.5). (D) Zoomed-in views of the final 3D density map from panel A, displayed at two contour levels. (E) Superimposition of the high-contour level map from panel D onto its flexibly fitted model.

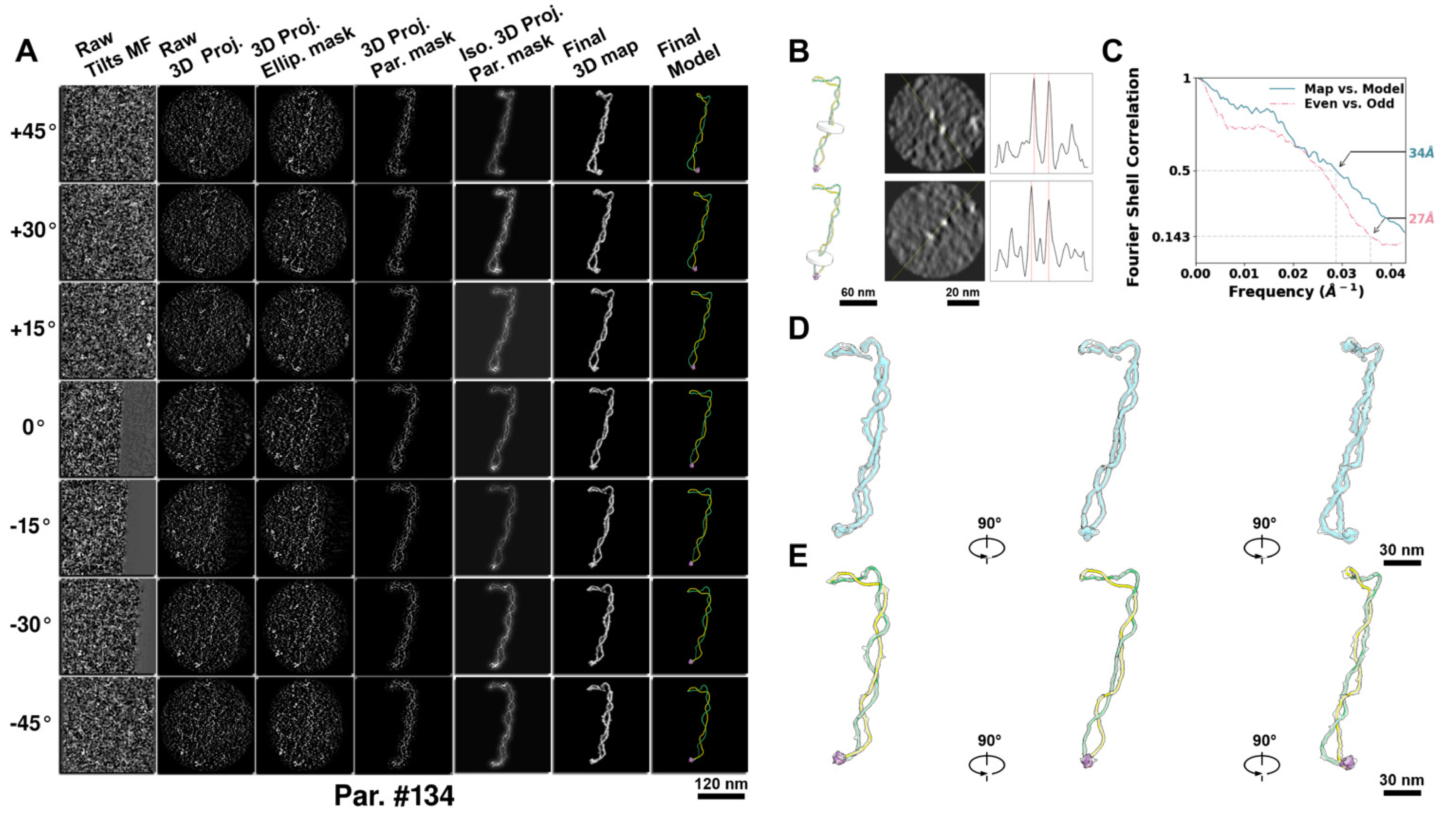

Supplementary Figure 134. Cryo-electron tomography (cryo-ET) 3D reconstruction of an individual plasmid particle in 40 mM K^{+}, 5 mM Mg^{2+} in the presence of transcribed RNAP.
(A) 3D reconstruction of the plasmid particle (index no. 134). The first column shows seven representative tilt images from –45° to +45° in step of 15°. The second, third, and fourth columns show 3D projections of the particle with spherical, ellipsoidal (thinner along the z-dimension), and particle-shaped masks, respectively. The fifth column displays the 3D projections of the enhanced and IsoNet missing-wedge-corrected particle. The sixth and seventh columns present the final 3D map and the flexibly fitted model, respectively. (B) Two random cross-sectional views (12 nm thickness) of the plasmid density map along its plectoneme axis are shown in the left-middle panel. The intensity profile along the line crossing the two high-density DNA spots is displayed in the right panel. (C) Resolution assessment of the final 3D map using Fourier shell correlation (FSC). Two criteria are shown: FSC between two half-maps reconstructed from even and odd frames (evaluated at 0.143) and FSC between the final 3D map and the fitted model (evaluated at 0.5). (D) Zoomed-in views of the final 3D density map from panel A, displayed at two contour levels. (E) Superimposition of the high-contour level map from panel D onto its flexibly fitted model.

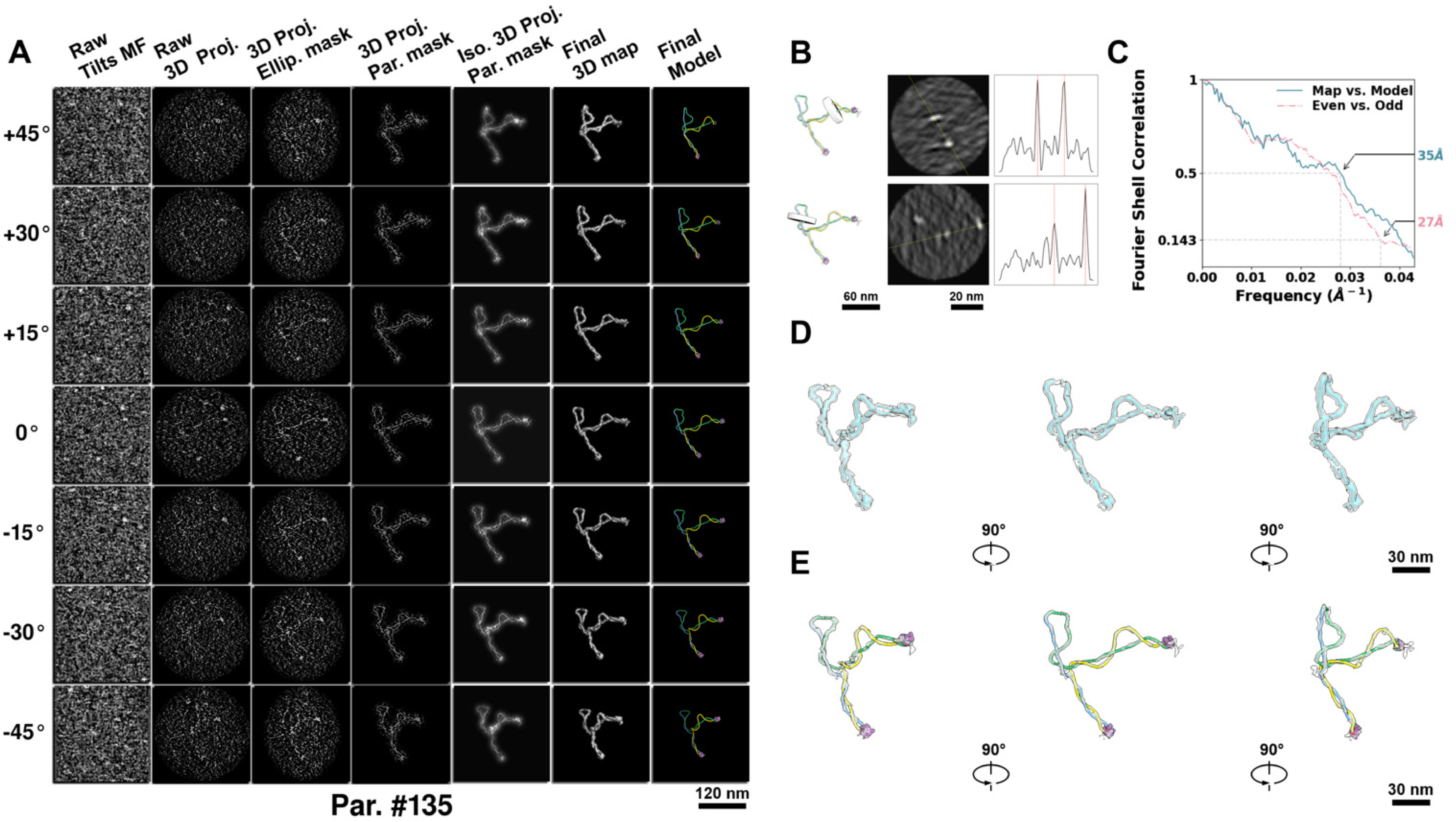

Supplementary Figure 135. Cryo-electron tomography (cryo-ET) 3D reconstruction of an individual plasmid particle in 40 mM K+, 5 mM Mg^{2+} in the presence of transcribed RNAP.
(A) 3D reconstruction of the plasmid particle (index no. 135). The first column shows seven representative tilt images from –45° to +45° in step of 15°. The second, third, and fourth columns show 3D projections of the particle with spherical, ellipsoidal (thinner along the z-dimension), and particle-shaped masks, respectively. The fifth column displays the 3D projections of the enhanced and IsoNet missing-wedge-corrected particle. The sixth and seventh columns present the final 3D map and the flexibly fitted model, respectively. (B) Two random cross-sectional views (12 nm thickness) of the plasmid density map along its plectoneme axis are shown in the left-middle panel. The intensity profile along the line crossing the two high-density DNA spots is displayed in the right panel. (C) Resolution assessment of the final 3D map using Fourier shell correlation (FSC). Two criteria are shown: FSC between two half-maps reconstructed from even and odd frames (evaluated at 0.143) and FSC between the final 3D map and the fitted model (evaluated at 0.5). (D) Zoomed-in views of the final 3D density map from panel A, displayed at two contour levels. (E) Superimposition of the high-contour level map from panel D onto its flexibly fitted model.

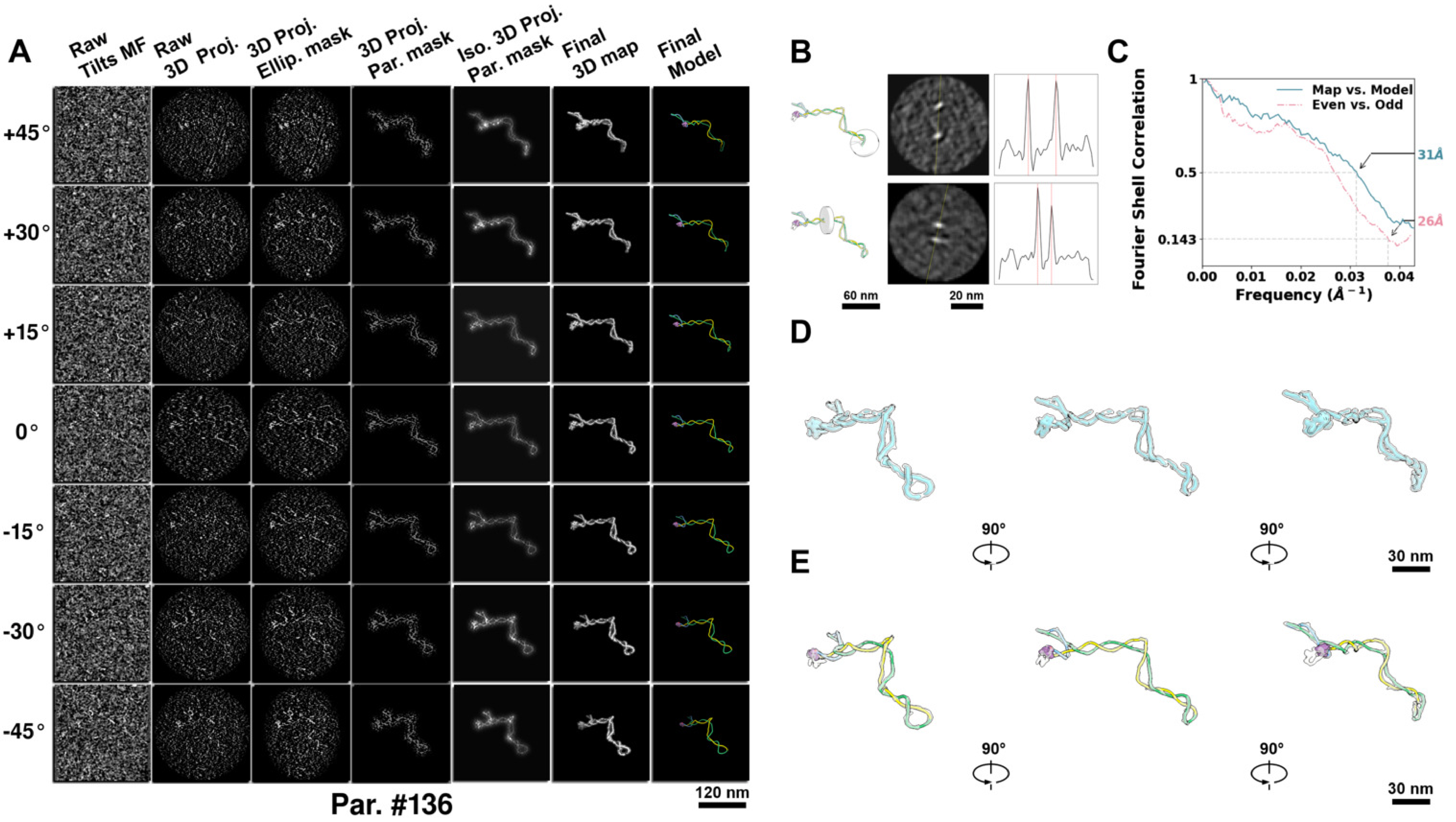

Supplementary Figure 136. Cryo-electron tomography (cryo-ET) 3D reconstruction of an individual plasmid particle in 40 mM K+, 5 mM Mg^{2+} in the presence of transcribed RNAP.
(A) 3D reconstruction of the plasmid particle (index no. 136). The first column shows seven representative tilt images from –45° to +45° in step of 15°. The second, third, and fourth columns show 3D projections of the particle with spherical, ellipsoidal (thinner along the z-dimension), and particle-shaped masks, respectively. The fifth column displays the 3D projections of the enhanced and IsoNet missing-wedge-corrected particle. The sixth and seventh columns present the final 3D map and the flexibly fitted model, respectively. (B) Two random cross-sectional views (12 nm thickness) of the plasmid density map along its plectoneme axis are shown in the left-middle panel. The intensity profile along the line crossing the two high-density DNA spots is displayed in the right panel. (C) Resolution assessment of the final 3D map using Fourier shell correlation (FSC). Two criteria are shown: FSC between two half-maps reconstructed from even and odd frames (evaluated at 0.143) and FSC between the final 3D map and the fitted model (evaluated at 0.5). (D) Zoomed-in views of the final 3D density map from panel A, displayed at two contour levels. (E) Superimposition of the high-contour level map from panel D onto its flexibly fitted model.

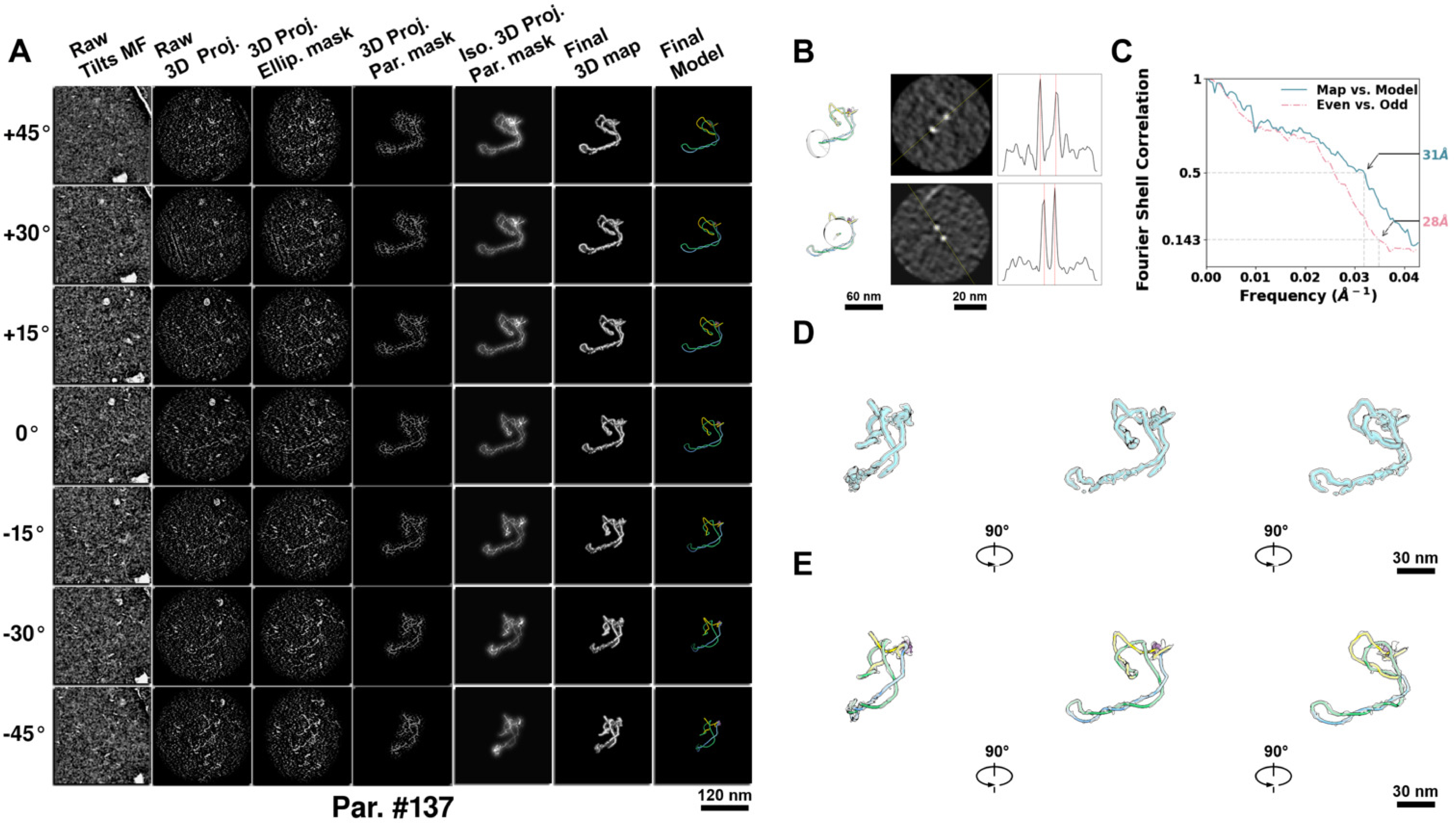

Supplementary Figure 137. Cryo-electron tomography (cryo-ET) 3D reconstruction of an individual plasmid particle in 40 mM K^+, 5 mM Mg^{2+} in the presence of transcribed RNAP.
(A) 3D reconstruction of the plasmid particle (index no. 137). The first column shows seven representative tilt images from –45° to +45° in step of 15°. The second, third, and fourth columns show 3D projections of the particle with spherical, ellipsoidal (thinner along the z-dimension), and particle-shaped masks, respectively. The fifth column displays the 3D projections of the enhanced and IsoNet missing-wedge-corrected particle. The sixth and seventh columns present the final 3D map and the flexibly fitted model, respectively. (B) Two random cross-sectional views (12 nm thickness) of the plasmid density map along its plectoneme axis are shown in the left-middle panel. The intensity profile along the line crossing the two high-density DNA spots is displayed in the right panel. (C) Resolution assessment of the final 3D map using Fourier shell correlation (FSC). Two criteria are shown: FSC between two half-maps reconstructed from even and odd frames (evaluated at 0.143) and FSC between the final 3D map and the fitted model (evaluated at 0.5). (D) Zoomed-in views of the final 3D density map from panel A, displayed at two contour levels. (E) Superimposition of the high-contour level map from panel D onto its flexibly fitted model.

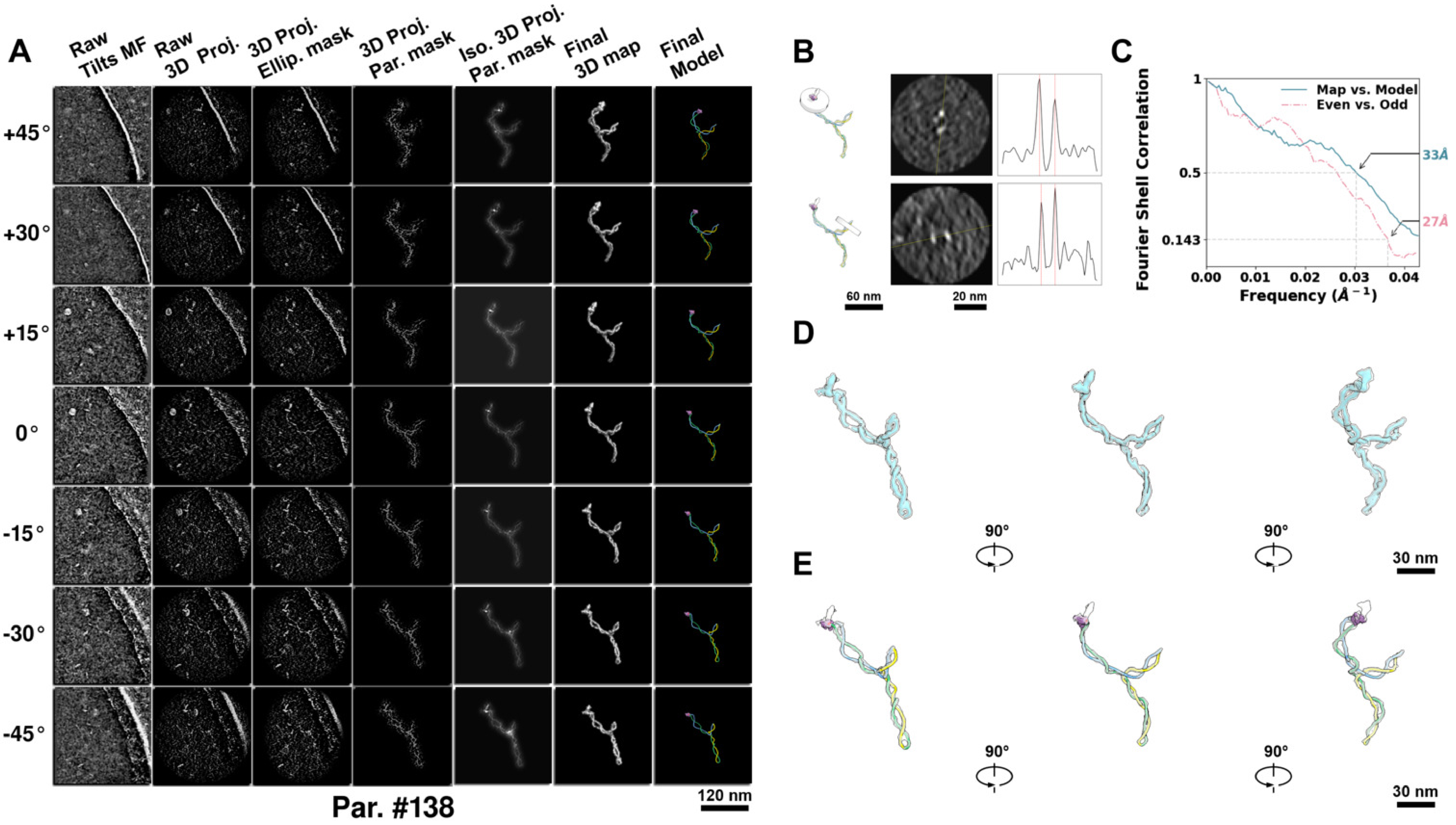

Supplementary Figure 138. Cryo-electron tomography (cryo-ET) 3D reconstruction of an individual plasmid particle in 40 mM K^+, 5 mM Mg^{2+} in the presence of transcribed RNAP.
(A) 3D reconstruction of the plasmid particle (index no. 138). The first column shows seven representative tilt images from –45° to +45° in step of 15°. The second, third, and fourth columns show 3D projections of the particle with spherical, ellipsoidal (thinner along the z-dimension), and particle-shaped masks, respectively. The fifth column displays the 3D projections of the enhanced and IsoNet missing-wedge-corrected particle. The sixth and seventh columns present the final 3D map and the flexibly fitted model, respectively. (B) Two random cross-sectional views (12 nm thickness) of the plasmid density map along its plectoneme axis are shown in the left-middle panel. The intensity profile along the line crossing the two high-density DNA spots is displayed in the right panel. (C) Resolution assessment of the final 3D map using Fourier shell correlation (FSC). Two criteria are shown: FSC between two half-maps reconstructed from even and odd frames (evaluated at 0.143) and FSC between the final 3D map and the fitted model (evaluated at 0.5). (D) Zoomed-in views of the final 3D density map from panel A, displayed at two contour levels. (E) Superimposition of the high-contour level map from panel D onto its flexibly fitted model.

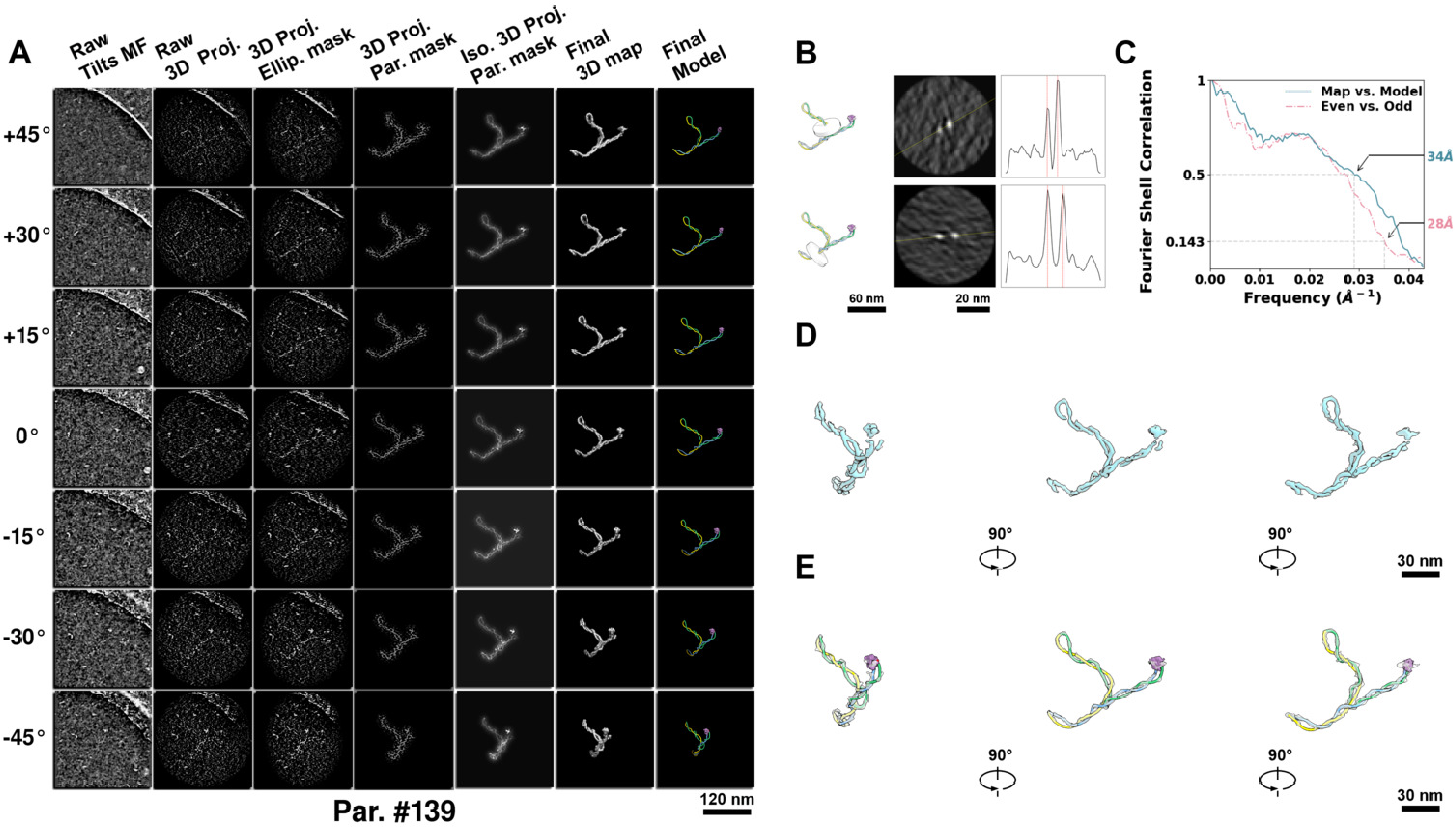

Supplementary Figure 139. Cryo-electron tomography (cryo-ET) 3D reconstruction of an individual plasmid particle in 40 mM K⁺, 5 mM Mg²⁺ in the presence of transcribed RNAP.
(A) 3D reconstruction of the plasmid particle (index no. 139). The first column shows seven representative tilt images from –45° to +45° in step of 15°. The second, third, and fourth columns show 3D projections of the particle with spherical, ellipsoidal (thinner along the z-dimension), and particle-shaped masks, respectively. The fifth column displays the 3D projections of the enhanced and IsoNet missing-wedge-corrected particle. The sixth and seventh columns present the final 3D map and the flexibly fitted model, respectively. (B) Two random cross-sectional views (12 nm thickness) of the plasmid density map along its plectoneme axis are shown in the left-middle panel. The intensity profile along the line crossing the two high-density DNA spots is displayed in the right panel. (C) Resolution assessment of the final 3D map using Fourier shell correlation (FSC). Two criteria are shown: FSC between two half-maps reconstructed from even and odd frames (evaluated at 0.143) and FSC between the final 3D map and the fitted model (evaluated at 0.5). (D) Zoomed-in views of the final 3D density map from panel A, displayed at two contour levels. (E) Superimposition of the high-contour level map from panel D onto its flexibly fitted model.

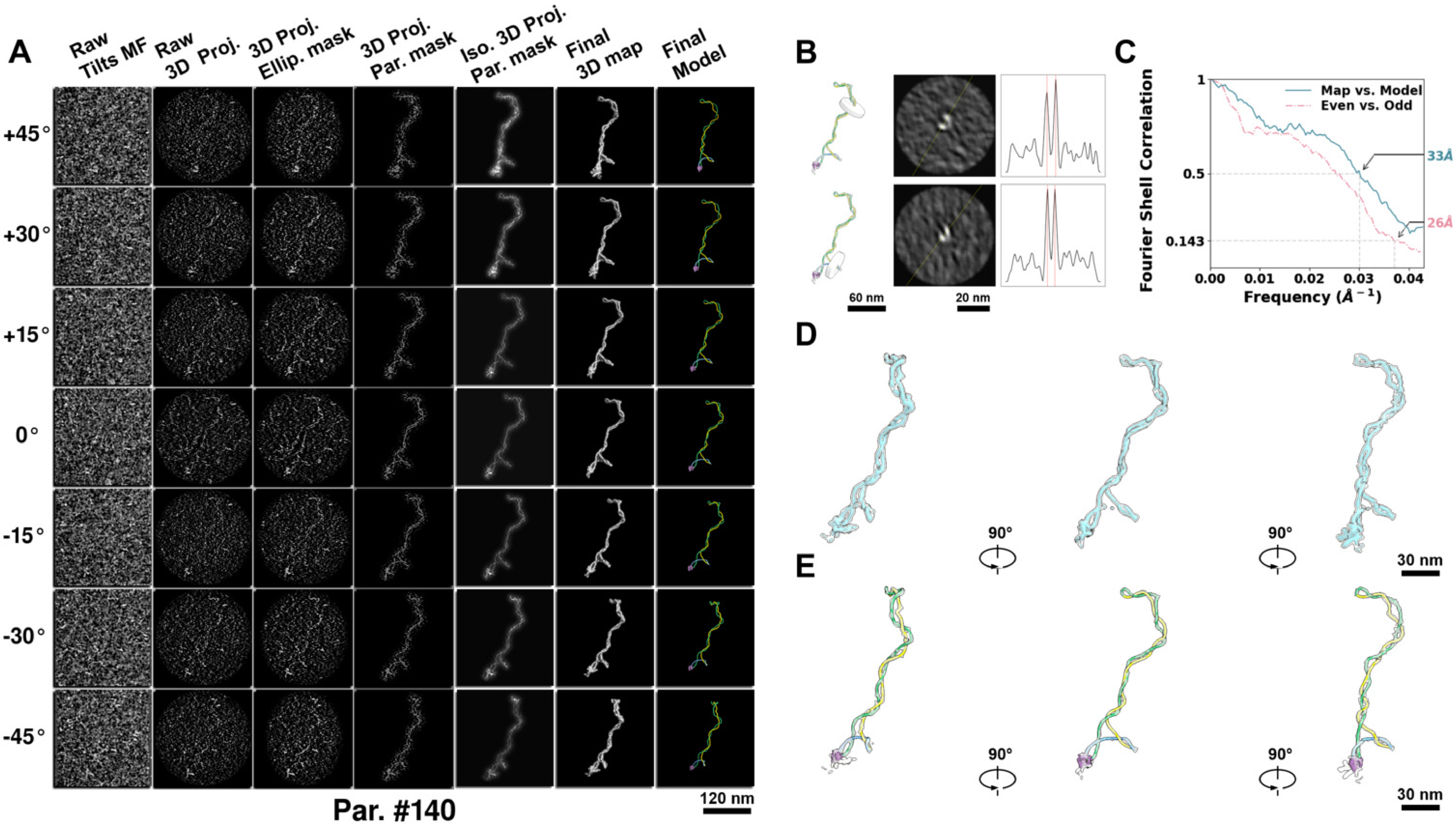

Supplementary Figure 140. Cryo-electron tomography (cryo-ET) 3D reconstruction of an individual plasmid particle in 40 mM K⁺, 5 mM Mg²⁺ in the presence of transcribed RNAP.
(A) 3D reconstruction of the plasmid particle (index no. 140). The first column shows seven representative tilt images from –45° to +45° in step of 15°. The second, third, and fourth columns show 3D projections of the particle with spherical, ellipsoidal (thinner along the z-dimension), and particle-shaped masks, respectively. The fifth column displays the 3D projections of the enhanced and IsoNet missing-wedge-corrected particle. The sixth and seventh columns present the final 3D map and the flexibly fitted model, respectively. (B) Two random cross-sectional views (12 nm thickness) of the plasmid density map along its plectoneme axis are shown in the left-middle panel. The intensity profile along the line crossing the two high-density DNA spots is displayed in the right panel. (C) Resolution assessment of the final 3D map using Fourier shell correlation (FSC). Two criteria are shown: FSC between two half-maps reconstructed from even and odd frames (evaluated at 0.143) and FSC between the final 3D map and the fitted model (evaluated at 0.5). (D) Zoomed-in views of the final 3D density map from panel A, displayed at two contour levels. (E) Superimposition of the high-contour level map from panel D onto its flexibly fitted model.

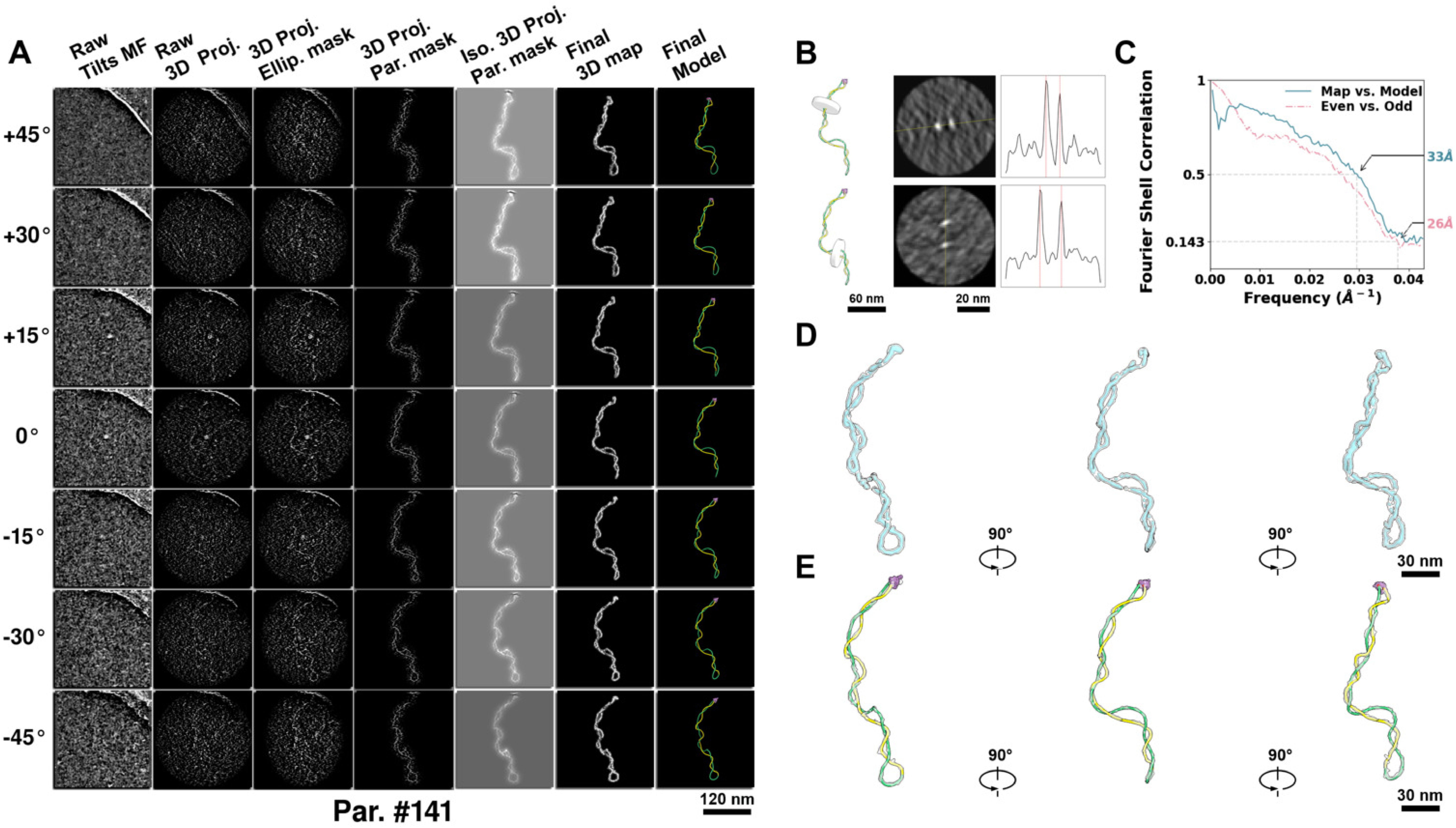

Supplementary Figure 141. Cryo-electron tomography (cryo-ET) 3D reconstruction of an individual plasmid particle in 40 mM K⁺, 5 mM Mg²⁺ in the presence of transcribed RNAP.
(A) 3D reconstruction of the plasmid particle (index no. 141). The first column shows seven representative tilt images from –45° to +45° in step of 15°. The second, third, and fourth columns show 3D projections of the particle with spherical, ellipsoidal (thinner along the z-dimension), and particle-shaped masks, respectively. The fifth column displays the 3D projections of the enhanced and IsoNet missing-wedge-corrected particle. The sixth and seventh columns present the final 3D map and the flexibly fitted model, respectively. (B) Two random cross-sectional views (12 nm thickness) of the plasmid density map along its plectoneme axis are shown in the left-middle panel. The intensity profile along the line crossing the two high-density DNA spots is displayed in the right panel. (C) Resolution assessment of the final 3D map using Fourier shell correlation (FSC). Two criteria are shown: FSC between two half-maps reconstructed from even and odd frames (evaluated at 0.143) and FSC between the final 3D map and the fitted model (evaluated at 0.5). (D) Zoomed-in views of the final 3D density map from panel A, displayed at two contour levels. (E) Superimposition of the high-contour level map from panel D onto its flexibly fitted model.

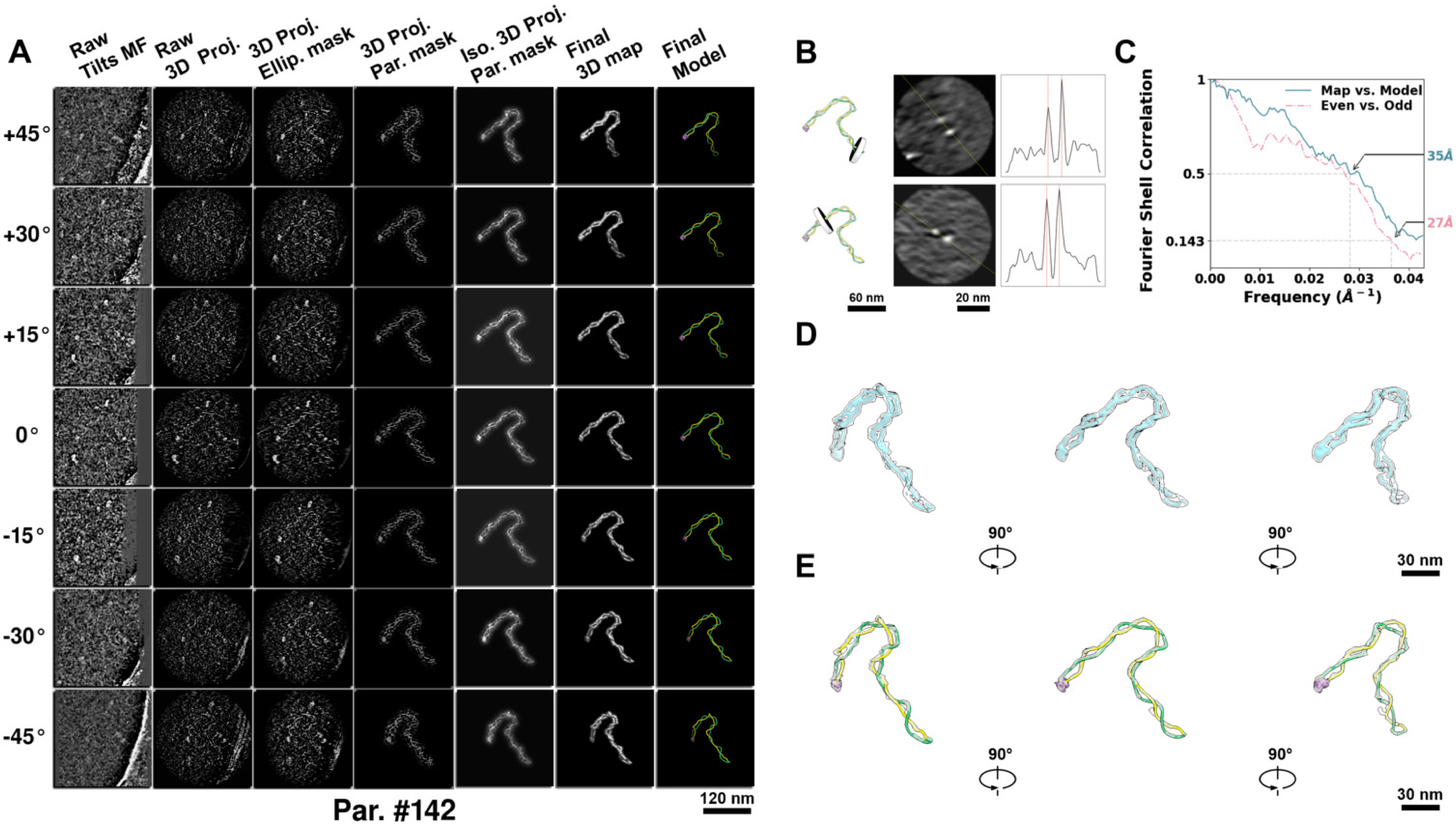

Supplementary Figure 142. Cryo-electron tomography (cryo-ET) 3D reconstruction of an individual plasmid particle in 40 mM K⁺, 5 mM Mg²⁺ in the presence of transcribed RNAP.
(A) 3D reconstruction of the plasmid particle (index no. 142). The first column shows seven representative tilt images from –45° to +45° in step of 15°. The second, third, and fourth columns show 3D projections of the particle with spherical, ellipsoidal (thinner along the z-dimension), and particle-shaped masks, respectively. The fifth column displays the 3D projections of the enhanced and IsoNet missing-wedge-corrected particle. The sixth and seventh columns present the final 3D map and the flexibly fitted model, respectively. (B) Two random cross-sectional views (12 nm thickness) of the plasmid density map along its plectoneme axis are shown in the left-middle panel. The intensity profile along the line crossing the two high-density DNA spots is displayed in the right panel. (C) Resolution assessment of the final 3D map using Fourier shell correlation (FSC). Two criteria are shown: FSC between two half-maps reconstructed from even and odd frames (evaluated at 0.143) and FSC between the final 3D map and the fitted model (evaluated at 0.5). (D) Zoomed-in views of the final 3D density map from panel A, displayed at two contour levels. (E) Superimposition of the high-contour level map from panel D onto its flexibly fitted model.

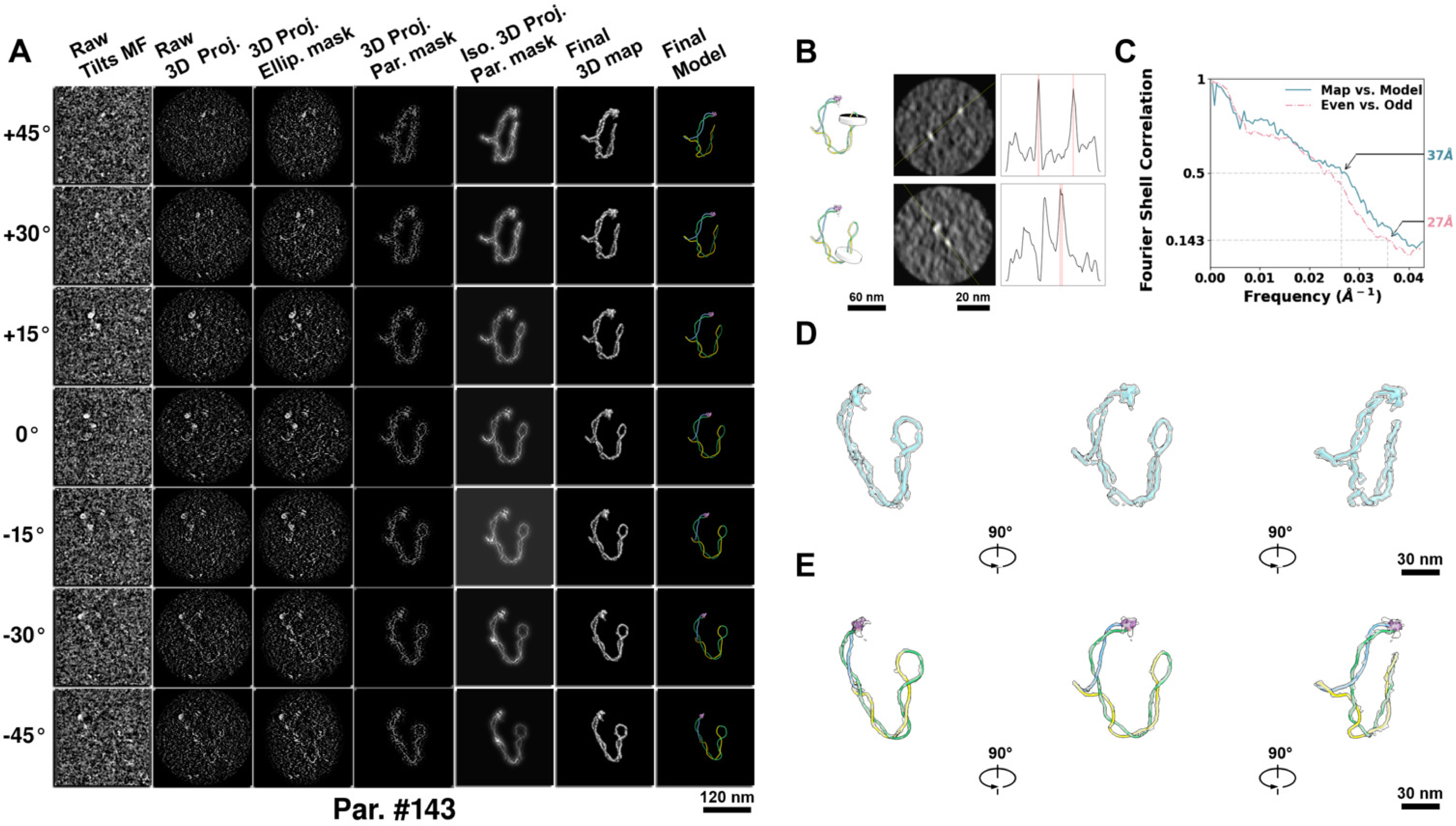

Supplementary Figure 143. Cryo-electron tomography (cryo-ET) 3D reconstruction of an individual plasmid particle in 40 mM K^+, 5 mM Mg^{2+} in the presence of transcribed RNAP.
(A) 3D reconstruction of the plasmid particle (index no. 143). The first column shows seven representative tilt images from –45° to +45° in step of 15°. The second, third, and fourth columns show 3D projections of the particle with spherical, ellipsoidal (thinner along the z-dimension), and particle-shaped masks, respectively. The fifth column displays the 3D projections of the enhanced and IsoNet missing-wedge-corrected particle. The sixth and seventh columns present the final 3D map and the flexibly fitted model, respectively. (B) Two random cross-sectional views (12 nm thickness) of the plasmid density map along its plectoneme axis are shown in the left-middle panel. The intensity profile along the line crossing the two high-density DNA spots is displayed in the right panel. (C) Resolution assessment of the final 3D map using Fourier shell correlation (FSC). Two criteria are shown: FSC between two half-maps reconstructed from even and odd frames (evaluated at 0.143) and FSC between the final 3D map and the fitted model (evaluated at 0.5). (D) Zoomed-in views of the final 3D density map from panel A, displayed at two contour levels. (E) Superimposition of the high-contour level map from panel D onto its flexibly fitted model.

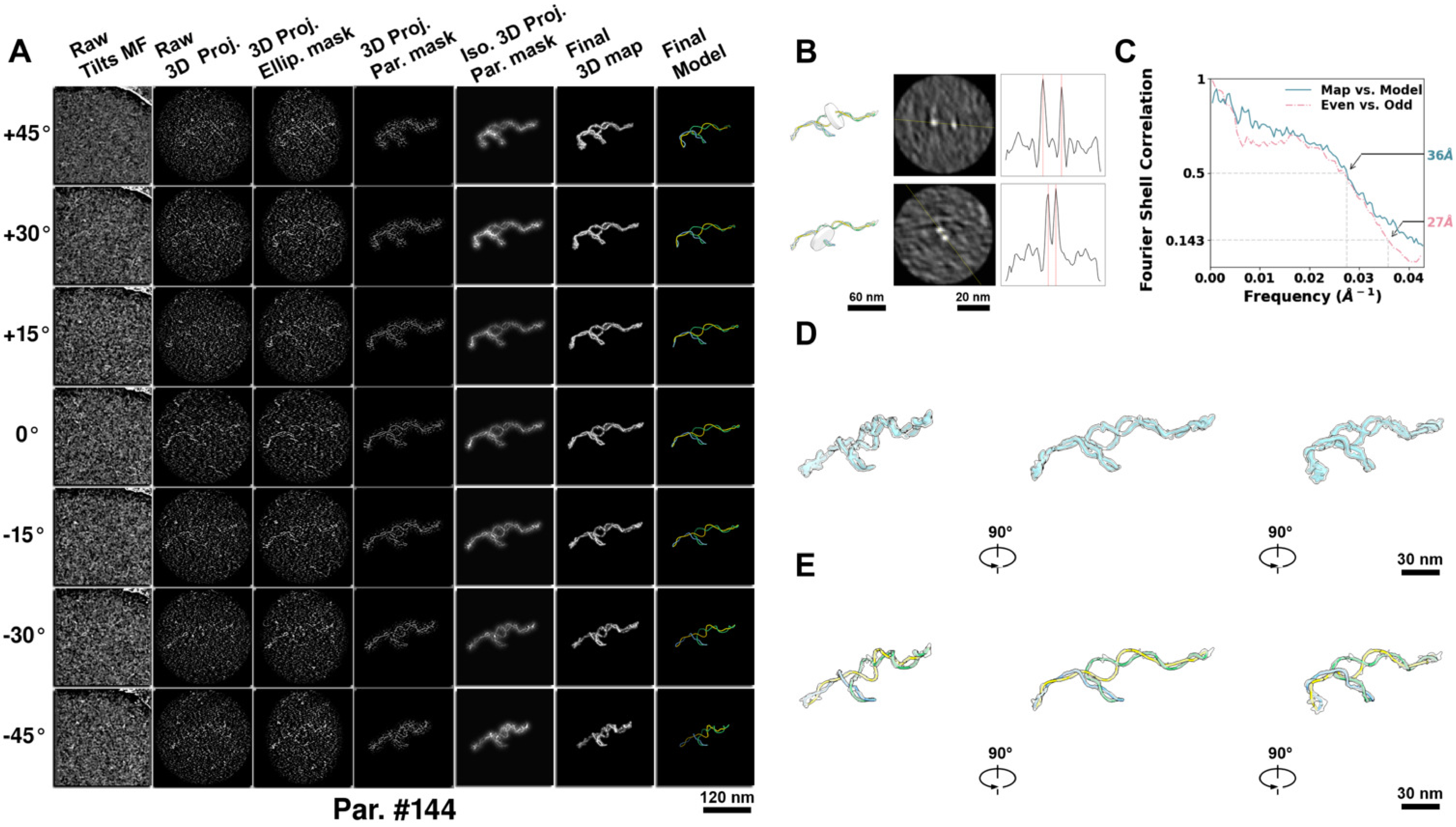

Supplementary Figure 144. Cryo-electron tomography (cryo-ET) 3D reconstruction of an individual plasmid particle in 40 mM K^+, 5 mM Mg^{2+} in the presence of transcribed RNAP.
(A) 3D reconstruction of the plasmid particle (index no. 144). The first column shows seven representative tilt images from –45° to +45° in step of 15°. The second, third, and fourth columns show 3D projections of the particle with spherical, ellipsoidal (thinner along the z-dimension), and particle-shaped masks, respectively. The fifth column displays the 3D projections of the enhanced and IsoNet missing-wedge-corrected particle. The sixth and seventh columns present the final 3D map and the flexibly fitted model, respectively. (B) Two random cross-sectional views (12 nm thickness) of the plasmid density map along its plectoneme axis are shown in the left-middle panel. The intensity profile along the line crossing the two high-density DNA spots is displayed in the right panel. (C) Resolution assessment of the final 3D map using Fourier shell correlation (FSC). Two criteria are shown: FSC between two half-maps reconstructed from even and odd frames (evaluated at 0.143) and FSC between the final 3D map and the fitted model (evaluated at 0.5). (D) Zoomed-in views of the final 3D density map from panel A, displayed at two contour levels. (E) Superimposition of the high-contour level map from panel D onto its flexibly fitted model.

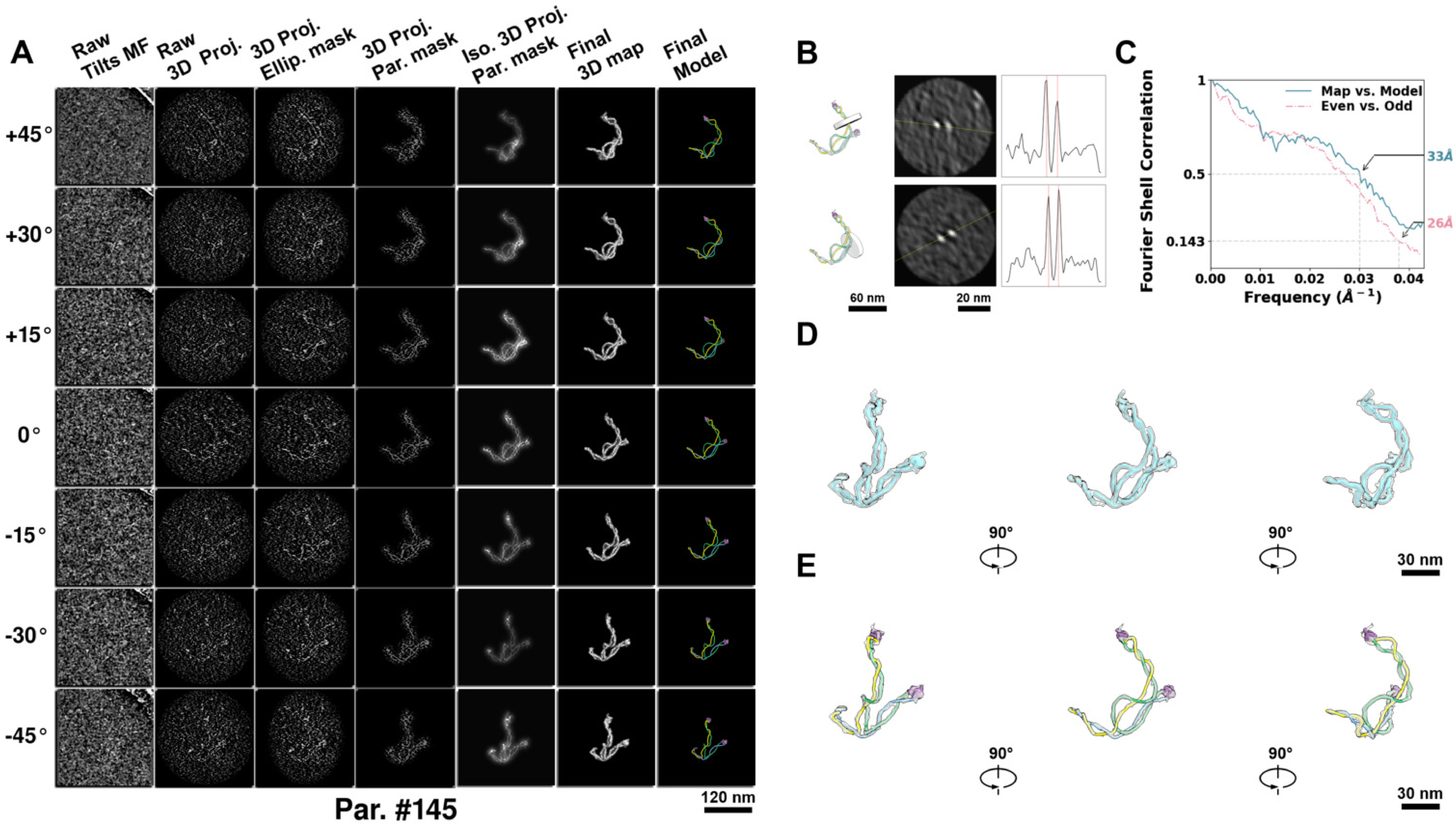

Supplementary Figure 145. Cryo-electron tomography (cryo-ET) 3D reconstruction of an individual plasmid particle in 40 mM K⁺, 5 mM Mg²⁺ in the presence of transcribed RNAP.
(A) 3D reconstruction of the plasmid particle (index no. 145). The first column shows seven representative tilt images from –45° to +45° in step of 15°. The second, third, and fourth columns show 3D projections of the particle with spherical, ellipsoidal (thinner along the z-dimension), and particle-shaped masks, respectively. The fifth column displays the 3D projections of the enhanced and IsoNet missing-wedge-corrected particle. The sixth and seventh columns present the final 3D map and the flexibly fitted model, respectively. (B) Two random cross-sectional views (12 nm thickness) of the plasmid density map along its plectoneme axis are shown in the left-middle panel. The intensity profile along the line crossing the two high-density DNA spots is displayed in the right panel. (C) Resolution assessment of the final 3D map using Fourier shell correlation (FSC). Two criteria are shown: FSC between two half-maps reconstructed from even and odd frames (evaluated at 0.143) and FSC between the final 3D map and the fitted model (evaluated at 0.5). (D) Zoomed-in views of the final 3D density map from panel A, displayed at two contour levels. (E) Superimposition of the high-contour level map from panel D onto its flexibly fitted model.

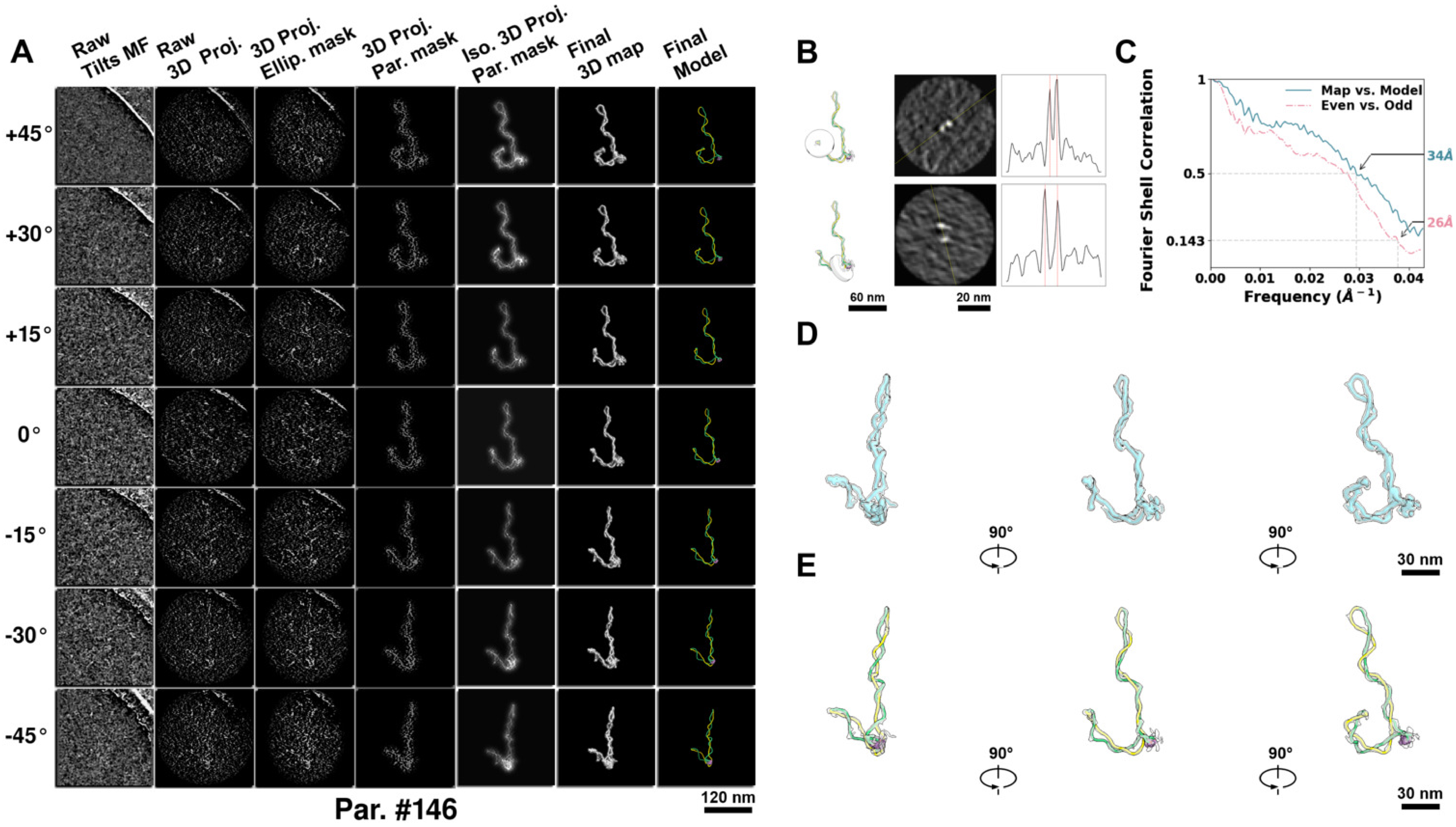

Supplementary Figure 146. Cryo-electron tomography (cryo-ET) 3D reconstruction of an individual plasmid particle in 40 mM K⁺, 5 mM Mg²⁺ in the presence of transcribed RNAP.
(A) 3D reconstruction of the plasmid particle (index no. 146). The first column shows seven representative tilt images from –45° to +45° in step of 15°. The second, third, and fourth columns show 3D projections of the particle with spherical, ellipsoidal (thinner along the z-dimension), and particle-shaped masks, respectively. The fifth column displays the 3D projections of the enhanced and IsoNet missing-wedge-corrected particle. The sixth and seventh columns present the final 3D map and the flexibly fitted model, respectively. (B) Two random cross-sectional views (12 nm thickness) of the plasmid density map along its plectoneme axis are shown in the left-middle panel. The intensity profile along the line crossing the two high-density DNA spots is displayed in the right panel. (C) Resolution assessment of the final 3D map using Fourier shell correlation (FSC). Two criteria are shown: FSC between two half-maps reconstructed from even and odd frames (evaluated at 0.143) and FSC between the final 3D map and the fitted model (evaluated at 0.5). (D) Zoomed-in views of the final 3D density map from panel A, displayed at two contour levels. (E) Superimposition of the high-contour level map from panel D onto its flexibly fitted model.

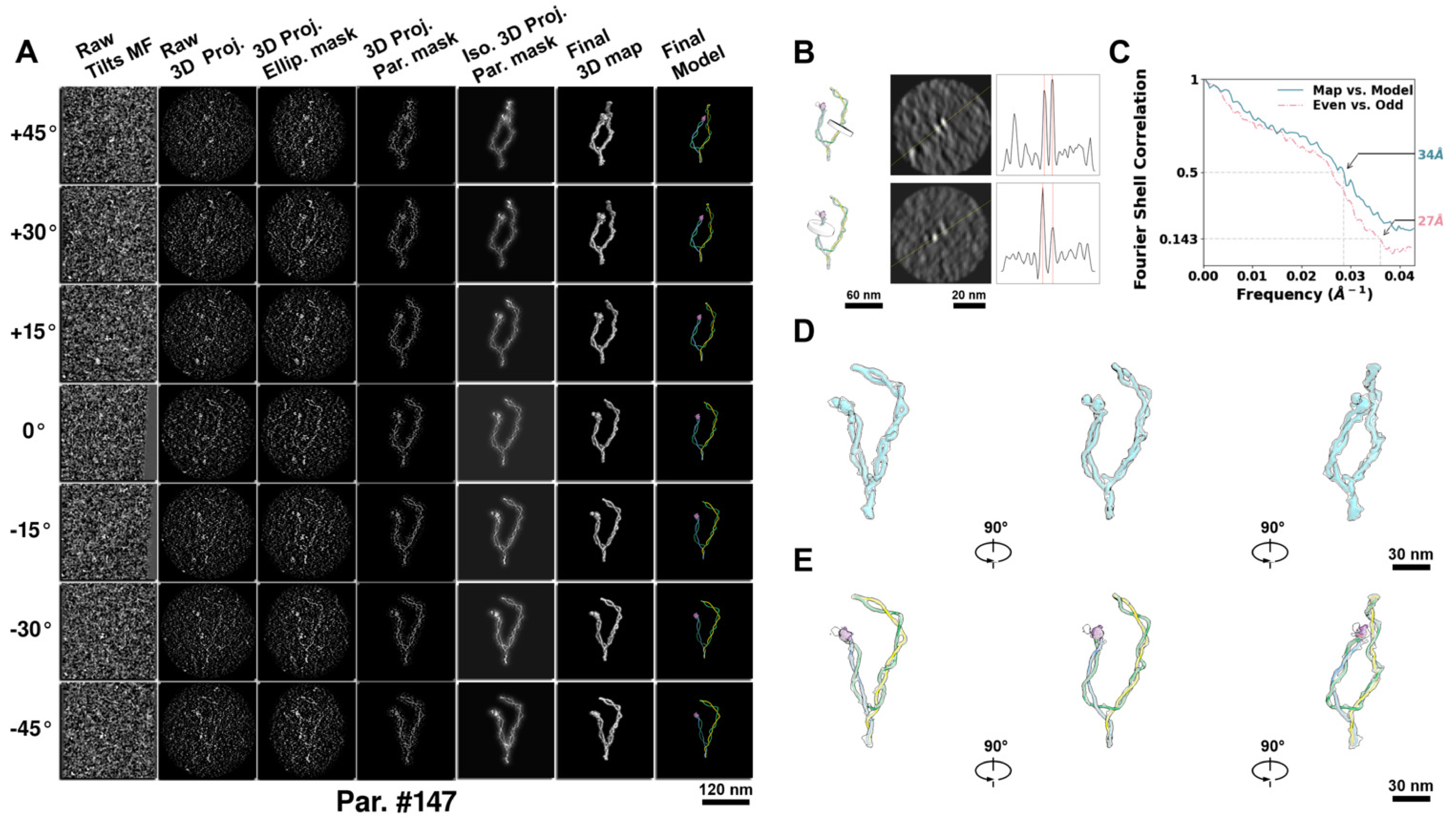

Supplementary Figure 147. Cryo-electron tomography (cryo-ET) 3D reconstruction of an individual plasmid particle in 40 mM K^{+}, 5 mM Mg^{2+} in the presence of transcribed RNAP.
(A) 3D reconstruction of the plasmid particle (index no. 147). The first column shows seven representative tilt images from –45° to +45° in step of 15°. The second, third, and fourth columns show 3D projections of the particle with spherical, ellipsoidal (thinner along the z-dimension), and particle-shaped masks, respectively. The fifth column displays the 3D projections of the enhanced and IsoNet missing-wedge-corrected particle. The sixth and seventh columns present the final 3D map and the flexibly fitted model, respectively. (B) Two random cross-sectional views (12 nm thickness) of the plasmid density map along its plectoneme axis are shown in the left-middle panel. The intensity profile along the line crossing the two high-density DNA spots is displayed in the right panel. (C) Resolution assessment of the final 3D map using Fourier shell correlation (FSC). Two criteria are shown: FSC between two half-maps reconstructed from even and odd frames (evaluated at 0.143) and FSC between the final 3D map and the fitted model (evaluated at 0.5). (D) Zoomed-in views of the final 3D density map from panel A, displayed at two contour levels. (E) Superimposition of the high-contour level map from panel D onto its flexibly fitted model.

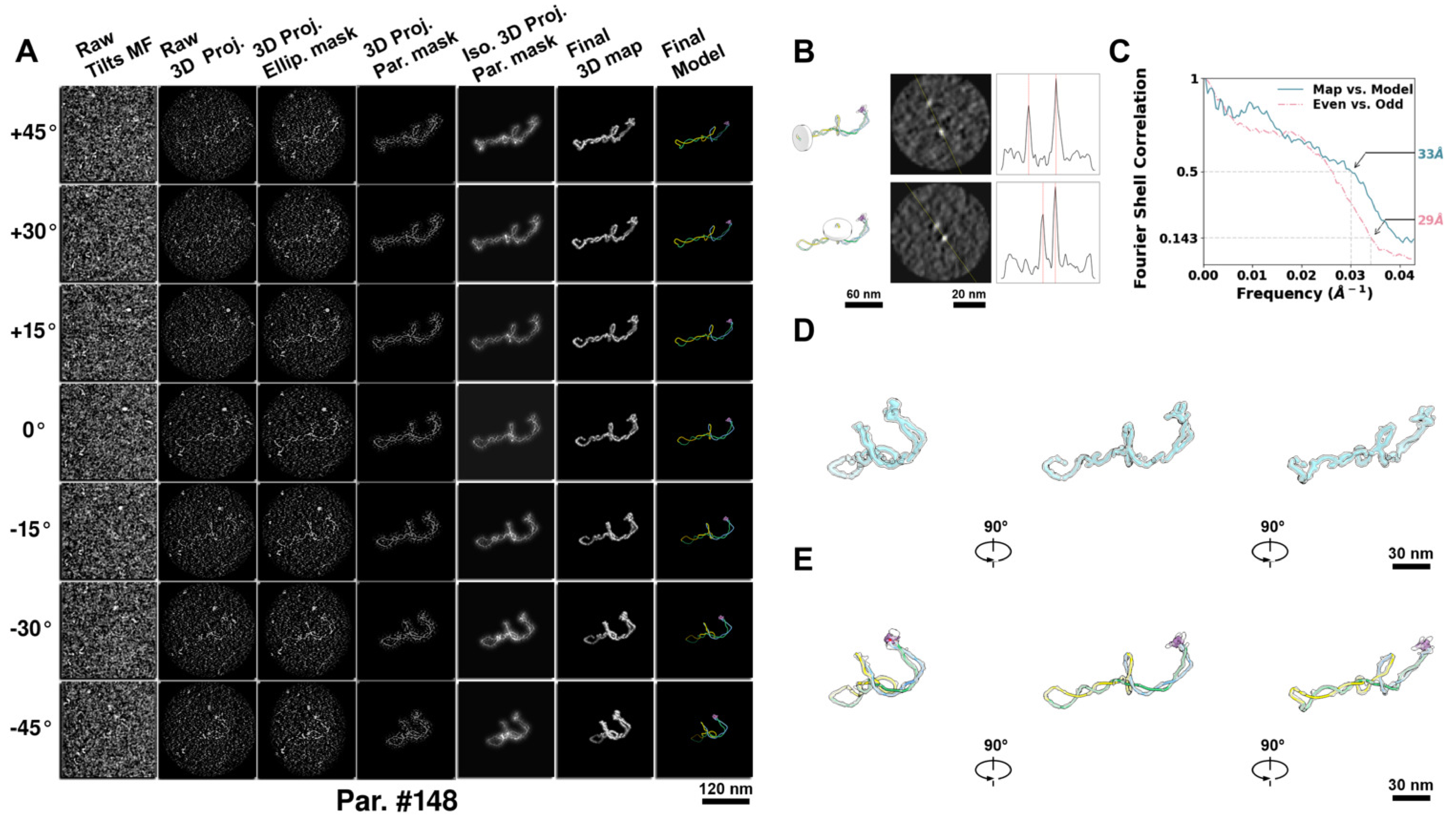

Supplementary Figure 148. Cryo-electron tomography (cryo-ET) 3D reconstruction of an individual plasmid particle in 40 mM K^{+}, 5 mM Mg^{2+} in the presence of transcribed RNAP.
(A) 3D reconstruction of the plasmid particle (index no. 148). The first column shows seven representative tilt images from –45° to +45° in step of 15°. The second, third, and fourth columns show 3D projections of the particle with spherical, ellipsoidal (thinner along the z-dimension), and particle-shaped masks, respectively. The fifth column displays the 3D projections of the enhanced and IsoNet missing-wedge-corrected particle. The sixth and seventh columns present the final 3D map and the flexibly fitted model, respectively. (B) Two random cross-sectional views (12 nm thickness) of the plasmid density map along its plectoneme axis are shown in the left-middle panel. The intensity profile along the line crossing the two high-density DNA spots is displayed in the right panel. (C) Resolution assessment of the final 3D map using Fourier shell correlation (FSC). Two criteria are shown: FSC between two half-maps reconstructed from even and odd frames (evaluated at 0.143) and FSC between the final 3D map and the fitted model (evaluated at 0.5). (D) Zoomed-in views of the final 3D density map from panel A, displayed at two contour levels. (E) Superimposition of the high-contour level map from panel D onto its flexibly fitted model.

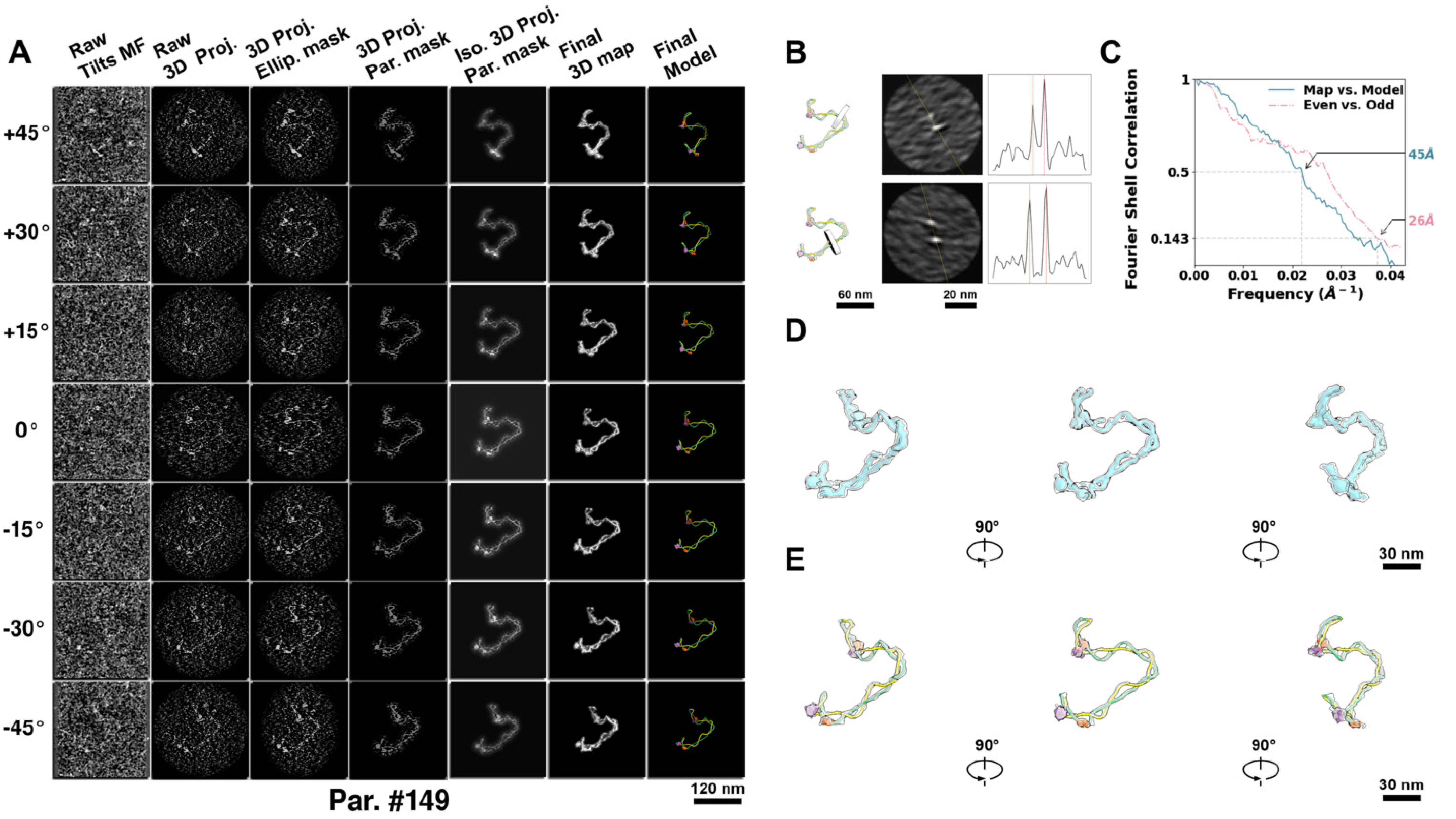

Supplementary Figure 149. Cryo-electron tomography (cryo-ET) 3D reconstruction of an individual plasmid particle in 40 mM K^+, 5 mM Mg^{2+} in the presence of stalled RNAP and dCas9.
(A) 3D reconstruction of the plasmid particle (index no. 149). The first column shows seven representative tilt images from –45° to +45° in step of 15°. The second, third, and fourth columns show 3D projections of the particle with spherical, ellipsoidal (thinner along the z-dimension), and particle-shaped masks, respectively. The fifth column displays the 3D projections of the enhanced and IsoNet missing-wedge-corrected particle. The sixth and seventh columns present the final 3D map and the flexibly fitted model, respectively. (B) Two random cross-sectional views (12 nm thickness) of the plasmid density map along its plectoneme axis are shown in the left-middle panel. The intensity profile along the line crossing the two high-density DNA spots is displayed in the right panel. (C) Resolution assessment of the final 3D map using Fourier shell correlation (FSC). Two criteria are shown: FSC between two half-maps reconstructed from even and odd frames (evaluated at 0.143) and FSC between the final 3D map and the fitted model (evaluated at 0.5). (D) Zoomed-in views of the final 3D density map from panel A, displayed at two contour levels. (E) Superimposition of the high-contour level map from panel D onto its flexibly fitted model.

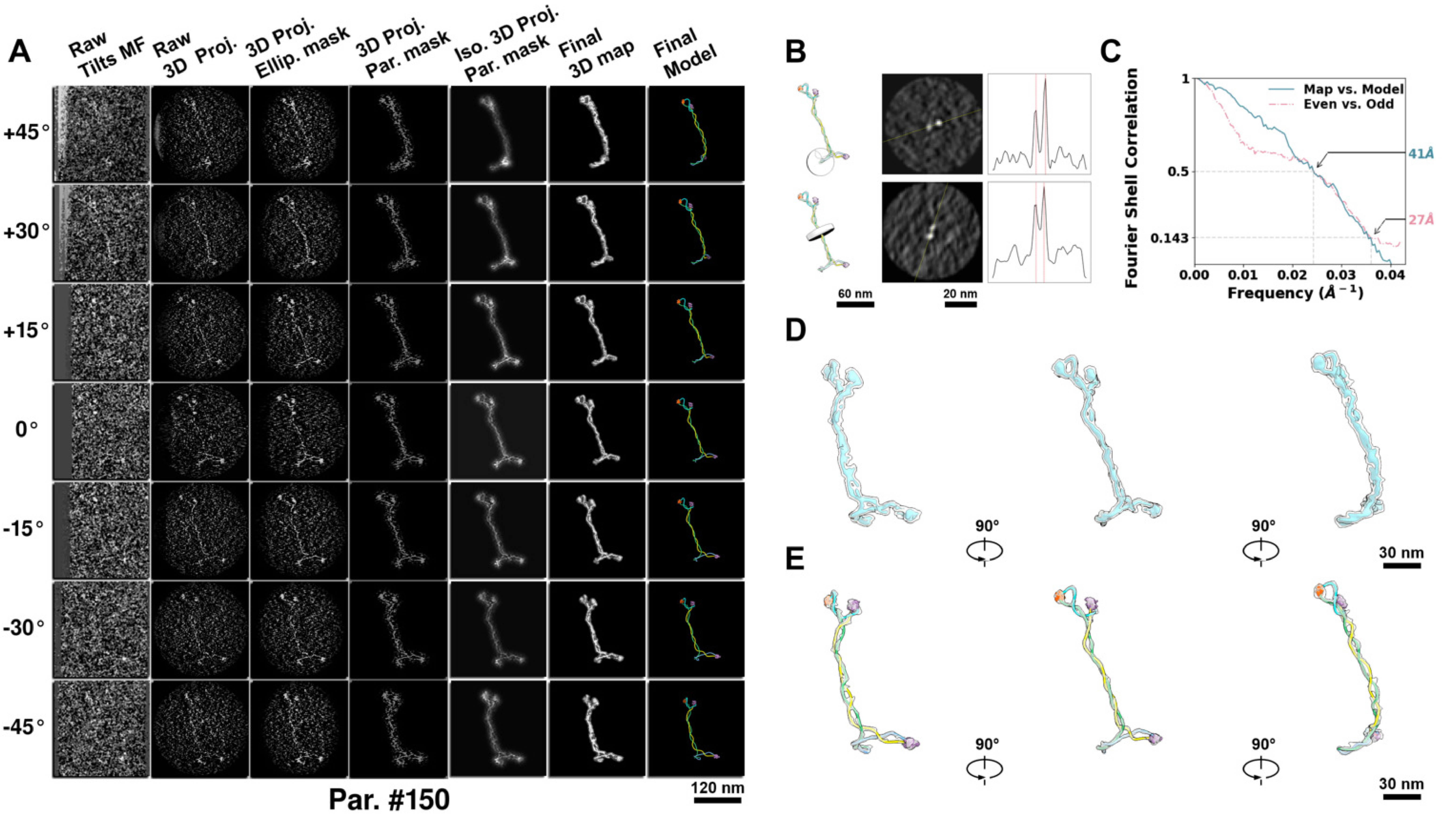

Supplementary Figure 150. Cryo-electron tomography (cryo-ET) 3D reconstruction of an individual plasmid particle in 40 mM K^+, 5 mM Mg^{2+} in the presence of stalled RNAP and dCas9.
(A) 3D reconstruction of the plasmid particle (index no. 150). The first column shows seven representative tilt images from –45° to +45° in step of 15°. The second, third, and fourth columns show 3D projections of the particle with spherical, ellipsoidal (thinner along the z-dimension), and particle-shaped masks, respectively. The fifth column displays the 3D projections of the enhanced and IsoNet missing-wedge-corrected particle. The sixth and seventh columns present the final 3D map and the flexibly fitted model, respectively. (B) Two random cross-sectional views (12 nm thickness) of the plasmid density map along its plectoneme axis are shown in the left-middle panel. The intensity profile along the line crossing the two high-density DNA spots is displayed in the right panel. (C) Resolution assessment of the final 3D map using Fourier shell correlation (FSC). Two criteria are shown: FSC between two half-maps reconstructed from even and odd frames (evaluated at 0.143) and FSC between the final 3D map and the fitted model (evaluated at 0.5). (D) Zoomed-in views of the final 3D density map from panel A, displayed at two contour levels. (E) Superimposition of the high-contour level map from panel D onto its flexibly fitted model.

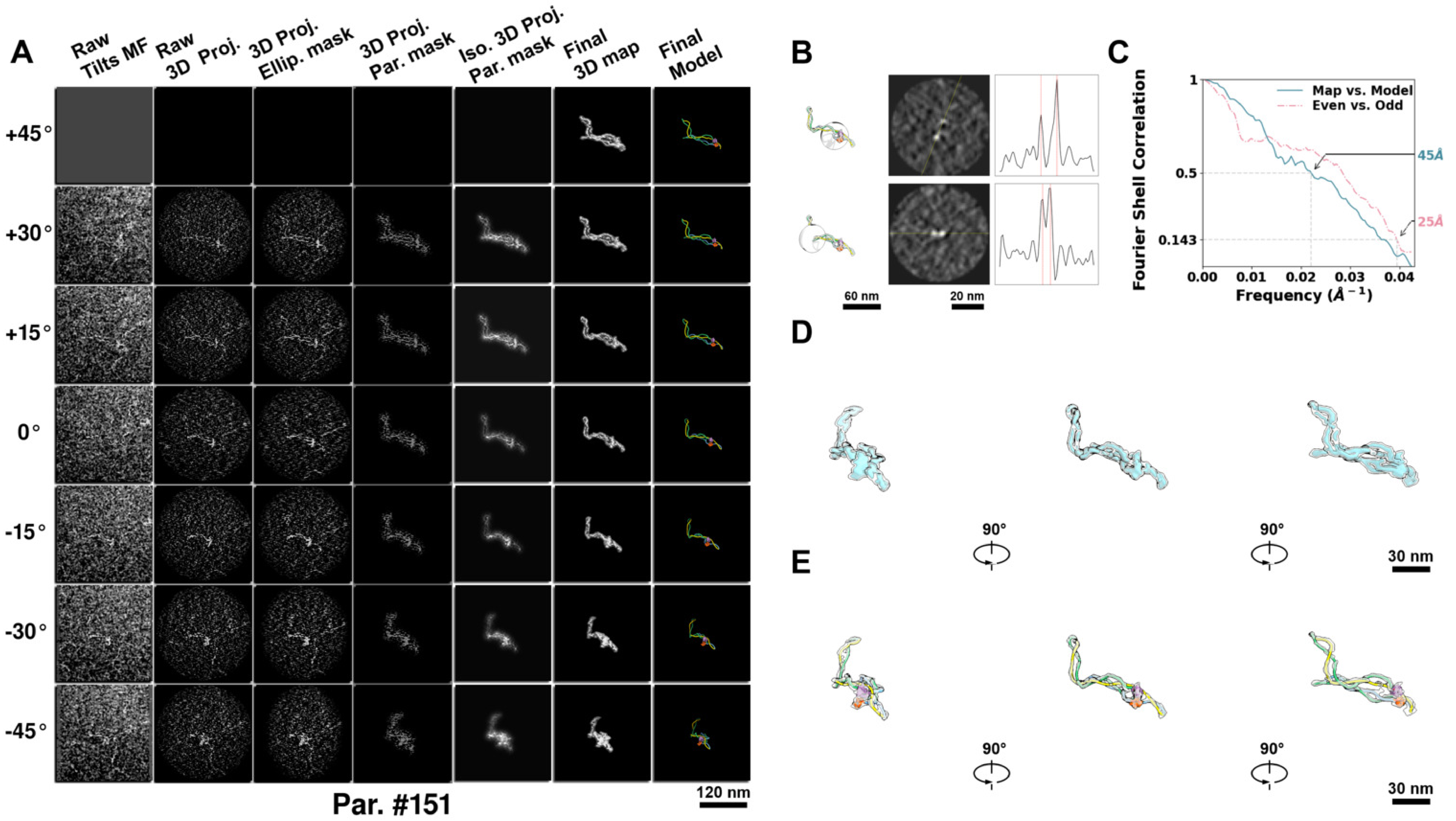

Supplementary Figure 151. Cryo-electron tomography (cryo-ET) 3D reconstruction of an individual plasmid particle in 40 mM K⁺, 5 mM Mg²⁺ in the presence of stalled RNAP and dCas9.
(A) 3D reconstruction of the plasmid particle (index no. 151). The first column shows seven representative tilt images from –45° to +45° in step of 15°. The second, third, and fourth columns show 3D projections of the particle with spherical, ellipsoidal (thinner along the z-dimension), and particle-shaped masks, respectively. The fifth column displays the 3D projections of the enhanced and IsoNet missing-wedge-corrected particle. The sixth and seventh columns present the final 3D map and the flexibly fitted model, respectively. (B) Two random cross-sectional views (12 nm thickness) of the plasmid density map along its plectoneme axis are shown in the left-middle panel. The intensity profile along the line crossing the two high-density DNA spots is displayed in the right panel. (C) Resolution assessment of the final 3D map using Fourier shell correlation (FSC). Two criteria are shown: FSC between two half-maps reconstructed from even and odd frames (evaluated at 0.143) and FSC between the final 3D map and the fitted model (evaluated at 0.5). (D) Zoomed-in views of the final 3D density map from panel A, displayed at two contour levels. (E) Superimposition of the high-contour level map from panel D onto its flexibly fitted model.

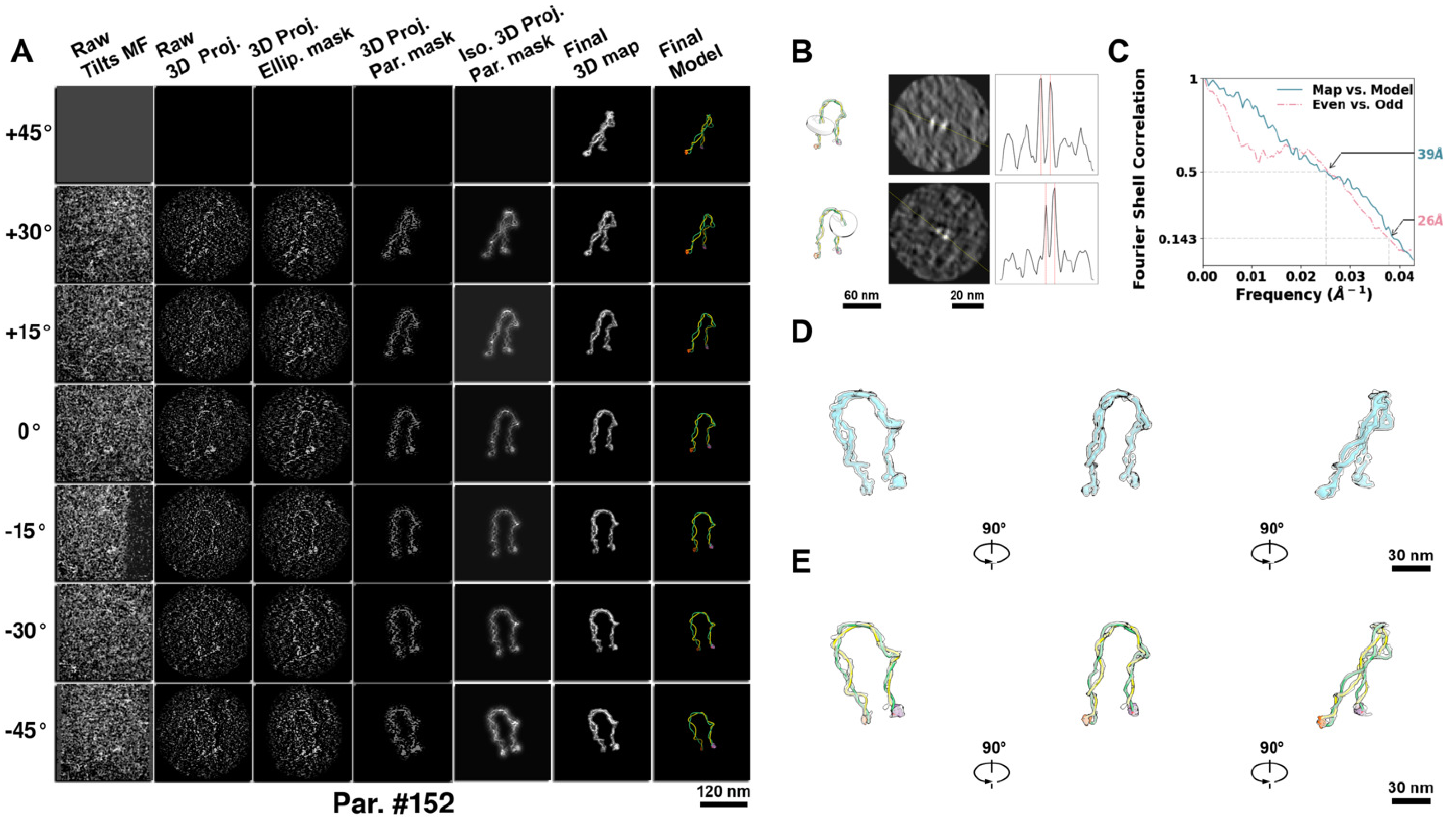

Supplementary Figure 152. Cryo-electron tomography (cryo-ET) 3D reconstruction of an individual plasmid particle in 40 mM K⁺, 5 mM Mg²⁺ in the presence of stalled RNAP and dCas9.
(A) 3D reconstruction of the plasmid particle (index no. 152). The first column shows seven representative tilt images from –45° to +45° in step of 15°. The second, third, and fourth columns show 3D projections of the particle with spherical, ellipsoidal (thinner along the z-dimension), and particle-shaped masks, respectively. The fifth column displays the 3D projections of the enhanced and IsoNet missing-wedge-corrected particle. The sixth and seventh columns present the final 3D map and the flexibly fitted model, respectively. (B) Two random cross-sectional views (12 nm thickness) of the plasmid density map along its plectoneme axis are shown in the left-middle panel. The intensity profile along the line crossing the two high-density DNA spots is displayed in the right panel. (C) Resolution assessment of the final 3D map using Fourier shell correlation (FSC). Two criteria are shown: FSC between two half-maps reconstructed from even and odd frames (evaluated at 0.143) and FSC between the final 3D map and the fitted model (evaluated at 0.5). (D) Zoomed-in views of the final 3D density map from panel A, displayed at two contour levels. (E) Superimposition of the high-contour level map from panel D onto its flexibly fitted model.

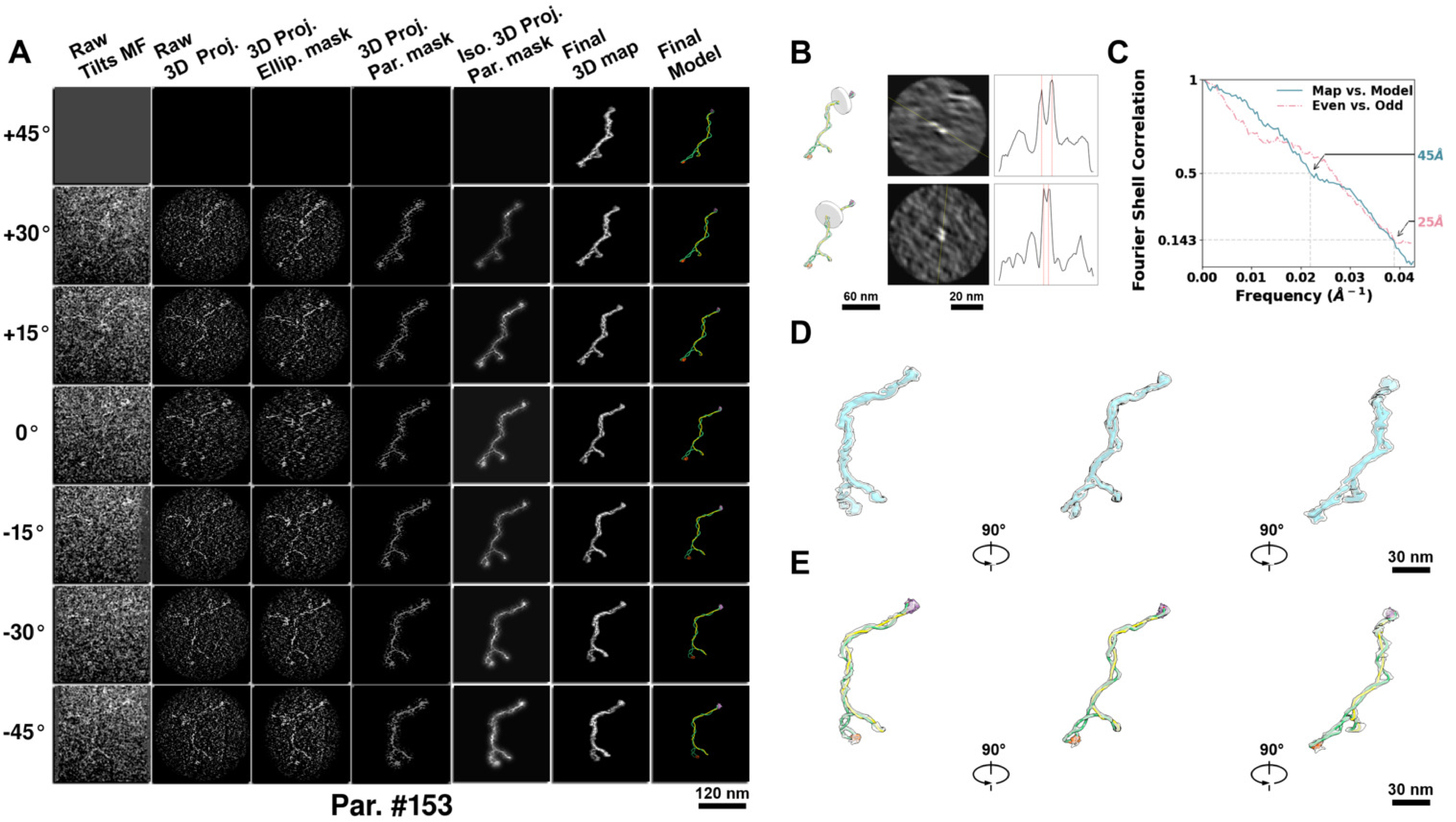

Supplementary Figure 153. Cryo-electron tomography (cryo-ET) 3D reconstruction of an individual plasmid particle in 40 mM K^+, 5 mM Mg^{2+} in the presence of stalled RNAP and dCas9.
(A) 3D reconstruction of the plasmid particle (index no. 153). The first column shows seven representative tilt images from –45° to +45° in step of 15°. The second, third, and fourth columns show 3D projections of the particle with spherical, ellipsoidal (thinner along the z-dimension), and particle-shaped masks, respectively. The fifth column displays the 3D projections of the enhanced and IsoNet missing-wedge-corrected particle. The sixth and seventh columns present the final 3D map and the flexibly fitted model, respectively. (B) Two random cross-sectional views (12 nm thickness) of the plasmid density map along its plectoneme axis are shown in the left-middle panel. The intensity profile along the line crossing the two high-density DNA spots is displayed in the right panel. (C) Resolution assessment of the final 3D map using Fourier shell correlation (FSC). Two criteria are shown: FSC between two half-maps reconstructed from even and odd frames (evaluated at 0.143) and FSC between the final 3D map and the fitted model (evaluated at 0.5). (D) Zoomed-in views of the final 3D density map from panel A, displayed at two contour levels. (E) Superimposition of the high-contour level map from panel D onto its flexibly fitted model.

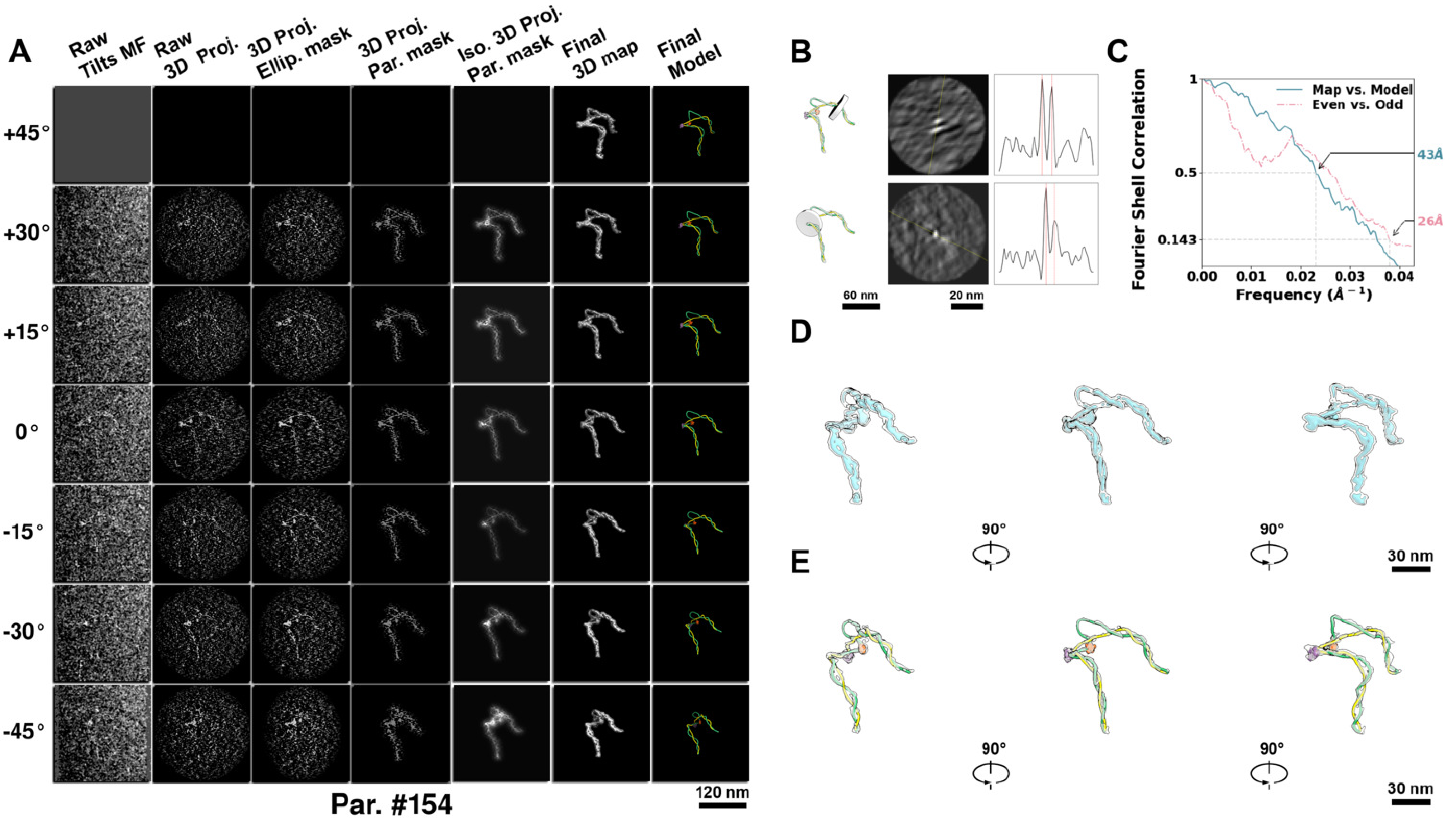

Supplementary Figure 154. Cryo-electron tomography (cryo-ET) 3D reconstruction of an individual plasmid particle in 40 mM K^+, 5 mM Mg^{2+} in the presence of stalled RNAP and dCas9.
(A) 3D reconstruction of the plasmid particle (index no. 154). The first column shows seven representative tilt images from –45° to +45° in step of 15°. The second, third, and fourth columns show 3D projections of the particle with spherical, ellipsoidal (thinner along the z-dimension), and particle-shaped masks, respectively. The fifth column displays the 3D projections of the enhanced and IsoNet missing-wedge-corrected particle. The sixth and seventh columns present the final 3D map and the flexibly fitted model, respectively. (B) Two random cross-sectional views (12 nm thickness) of the plasmid density map along its plectoneme axis are shown in the left-middle panel. The intensity profile along the line crossing the two high-density DNA spots is displayed in the right panel. (C) Resolution assessment of the final 3D map using Fourier shell correlation (FSC). Two criteria are shown: FSC between two half-maps reconstructed from even and odd frames (evaluated at 0.143) and FSC between the final 3D map and the fitted model (evaluated at 0.5). (D) Zoomed-in views of the final 3D density map from panel A, displayed at two contour levels. (E) Superimposition of the high-contour level map from panel D onto its flexibly fitted model.

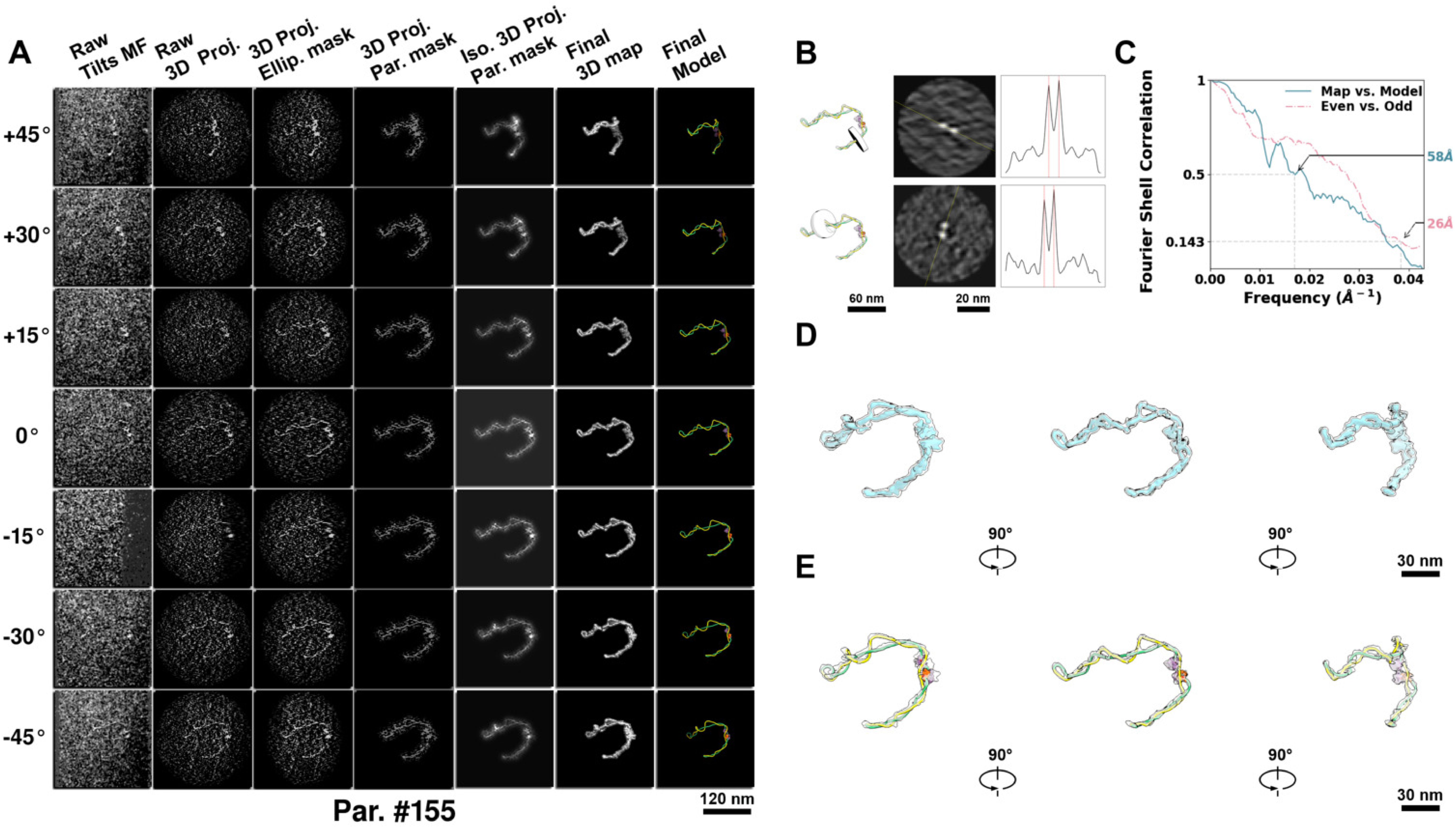

Supplementary Figure 155. Cryo-electron tomography (cryo-ET) 3D reconstruction of an individual plasmid particle in 40 mM K⁺, 5 mM Mg²⁺ in the presence of stalled RNAP and dCas9.
(A) 3D reconstruction of the plasmid particle (index no. 155). The first column shows seven representative tilt images from –45° to +45° in step of 15°. The second, third, and fourth columns show 3D projections of the particle with spherical, ellipsoidal (thinner along the z-dimension), and particle-shaped masks, respectively. The fifth column displays the 3D projections of the enhanced and IsoNet missing-wedge-corrected particle. The sixth and seventh columns present the final 3D map and the flexibly fitted model, respectively. (B) Two random cross-sectional views (12 nm thickness) of the plasmid density map along its plectoneme axis are shown in the left-middle panel. The intensity profile along the line crossing the two high-density DNA spots is displayed in the right panel. (C) Resolution assessment of the final 3D map using Fourier shell correlation (FSC). Two criteria are shown: FSC between two half-maps reconstructed from even and odd frames (evaluated at 0.143) and FSC between the final 3D map and the fitted model (evaluated at 0.5). (D) Zoomed-in views of the final 3D density map from panel A, displayed at two contour levels. (E) Superimposition of the high-contour level map from panel D onto its flexibly fitted model.

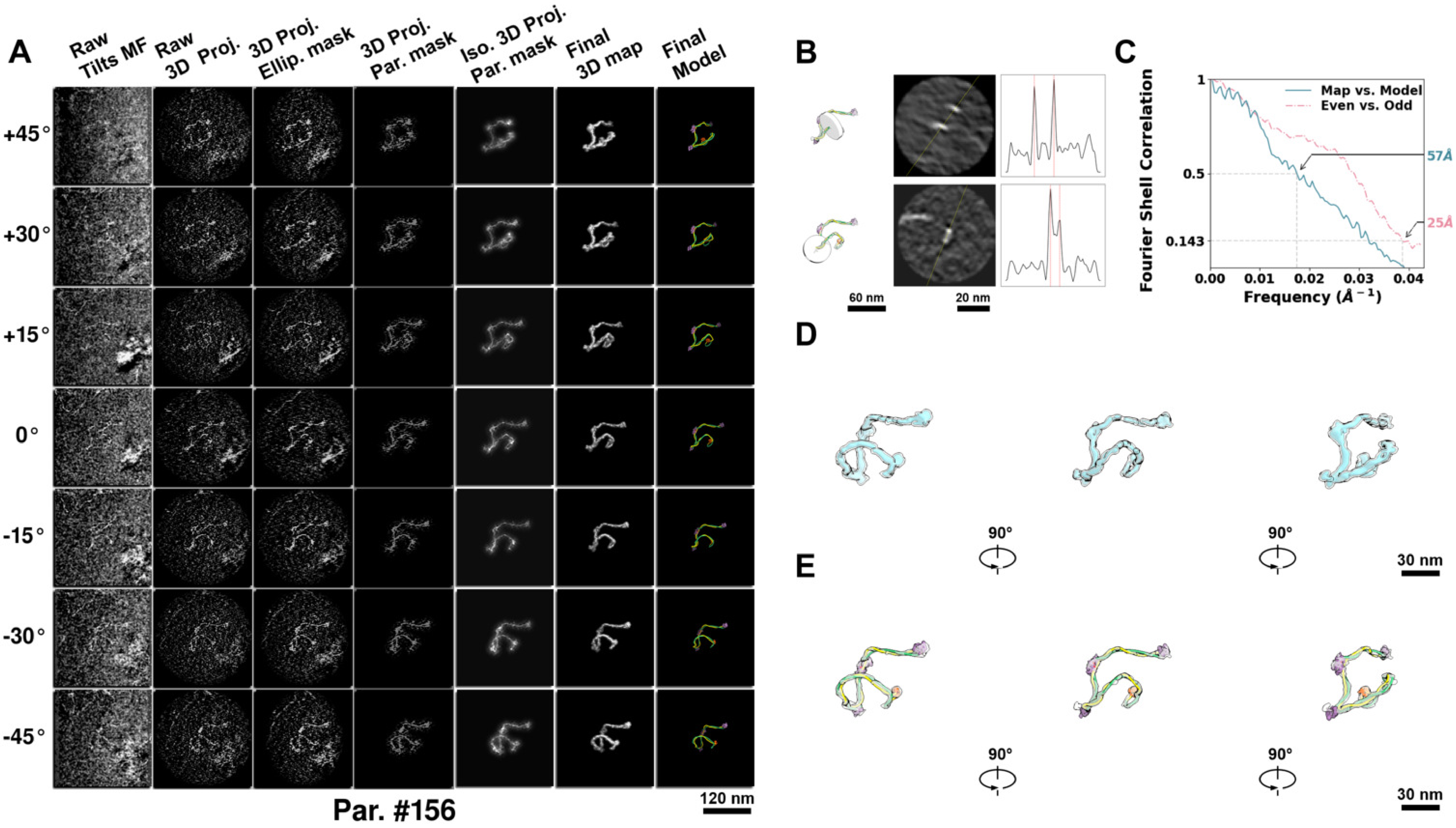

Supplementary Figure 156. Cryo-electron tomography (cryo-ET) 3D reconstruction of an individual plasmid particle in 40 mM K⁺, 5 mM Mg²⁺ in the presence of stalled RNAP and dCas9.
(A) 3D reconstruction of the plasmid particle (index no. 156). The first column shows seven representative tilt images from –45° to +45° in step of 15°. The second, third, and fourth columns show 3D projections of the particle with spherical, ellipsoidal (thinner along the z-dimension), and particle-shaped masks, respectively. The fifth column displays the 3D projections of the enhanced and IsoNet missing-wedge-corrected particle. The sixth and seventh columns present the final 3D map and the flexibly fitted model, respectively. (B) Two random cross-sectional views (12 nm thickness) of the plasmid density map along its plectoneme axis are shown in the left-middle panel. The intensity profile along the line crossing the two high-density DNA spots is displayed in the right panel. (C) Resolution assessment of the final 3D map using Fourier shell correlation (FSC). Two criteria are shown: FSC between two half-maps reconstructed from even and odd frames (evaluated at 0.143) and FSC between the final 3D map and the fitted model (evaluated at 0.5). (D) Zoomed-in views of the final 3D density map from panel A, displayed at two contour levels. (E) Superimposition of the high-contour level map from panel D onto its flexibly fitted model.

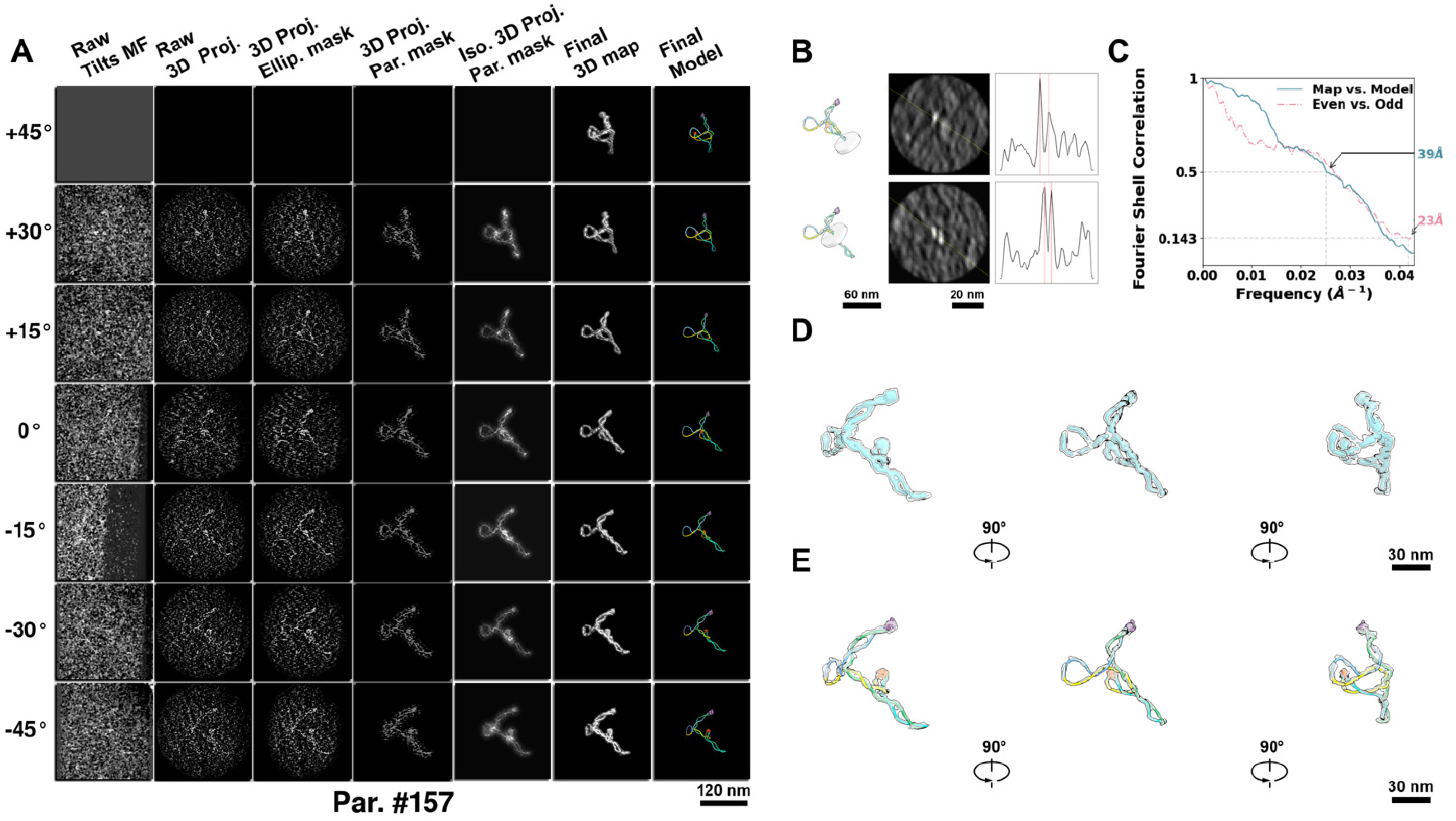

Supplementary Figure 157. Cryo-electron tomography (cryo-ET) 3D reconstruction of an individual plasmid particle in 40 mM K^+, 5 mM Mg^{2+} in the presence of stalled RNAP and dCas9.
(A) 3D reconstruction of the plasmid particle (index no. 157). The first column shows seven representative tilt images from –45° to +45° in step of 15°. The second, third, and fourth columns show 3D projections of the particle with spherical, ellipsoidal (thinner along the z-dimension), and particle-shaped masks, respectively. The fifth column displays the 3D projections of the enhanced and IsoNet missing-wedge-corrected particle. The sixth and seventh columns present the final 3D map and the flexibly fitted model, respectively. (B) Two random cross-sectional views (12 nm thickness) of the plasmid density map along its plectoneme axis are shown in the left-middle panel. The intensity profile along the line crossing the two high-density DNA spots is displayed in the right panel. (C) Resolution assessment of the final 3D map using Fourier shell correlation (FSC). Two criteria are shown: FSC between two half-maps reconstructed from even and odd frames (evaluated at 0.143) and FSC between the final 3D map and the fitted model (evaluated at 0.5). (D) Zoomed-in views of the final 3D density map from panel A, displayed at two contour levels. (E) Superimposition of the high-contour level map from panel D onto its flexibly fitted model.

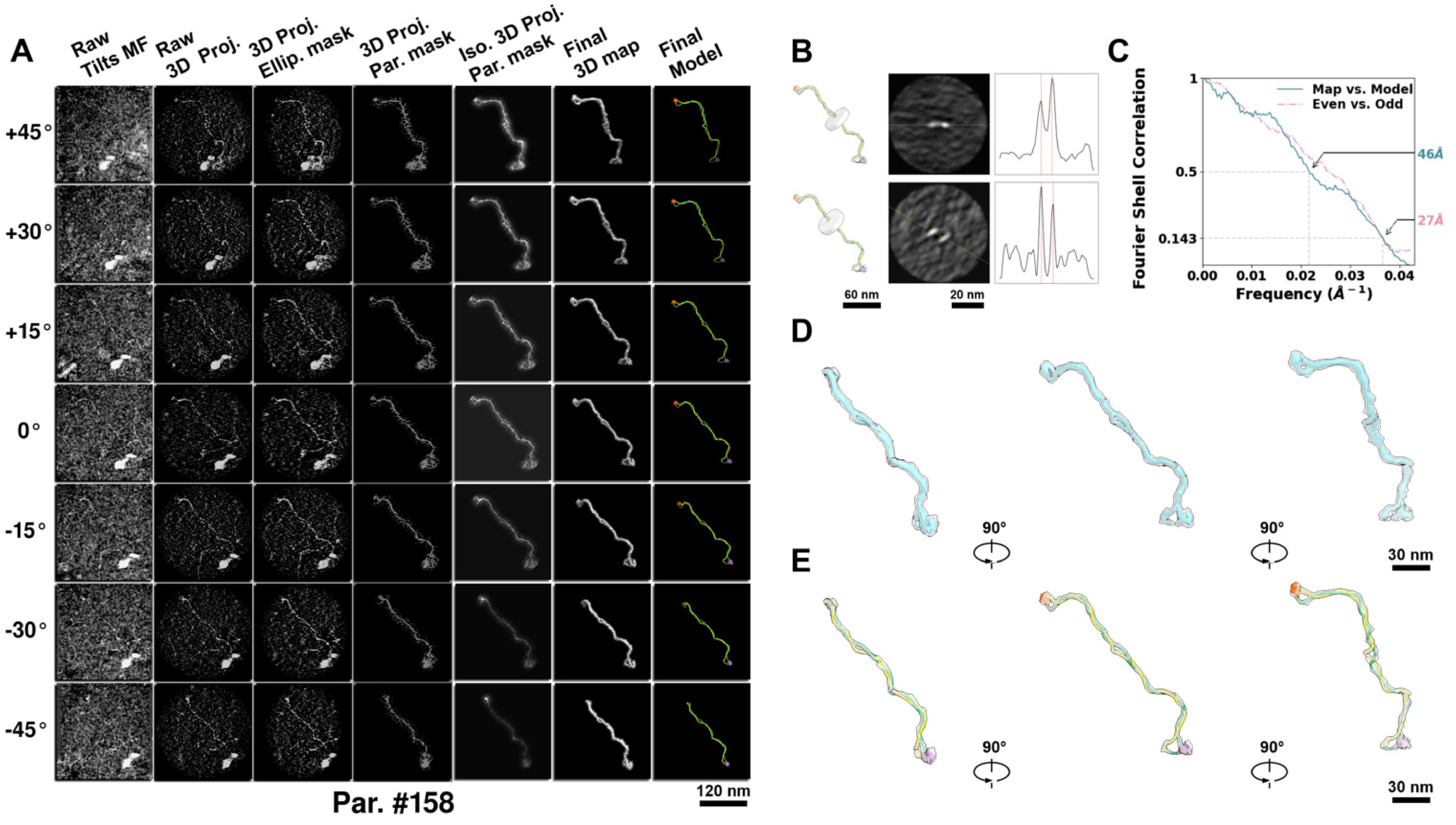

Supplementary Figure 158. Cryo-electron tomography (cryo-ET) 3D reconstruction of an individual plasmid particle in 40 mM K^+, 5 mM Mg^{2+} in the presence of stalled RNAP and dCas9.
(A) 3D reconstruction of the plasmid particle (index no. 158). The first column shows seven representative tilt images from –45° to +45° in step of 15°. The second, third, and fourth columns show 3D projections of the particle with spherical, ellipsoidal (thinner along the z-dimension), and particle-shaped masks, respectively. The fifth column displays the 3D projections of the enhanced and IsoNet missing-wedge-corrected particle. The sixth and seventh columns present the final 3D map and the flexibly fitted model, respectively. (B) Two random cross-sectional views (12 nm thickness) of the plasmid density map along its plectoneme axis are shown in the left-middle panel. The intensity profile along the line crossing the two high-density DNA spots is displayed in the right panel. (C) Resolution assessment of the final 3D map using Fourier shell correlation (FSC). Two criteria are shown: FSC between two half-maps reconstructed from even and odd frames (evaluated at 0.143) and FSC between the final 3D map and the fitted model (evaluated at 0.5). (D) Zoomed-in views of the final 3D density map from panel A, displayed at two contour levels. (E) Superimposition of the high-contour level map from panel D onto its flexibly fitted model.

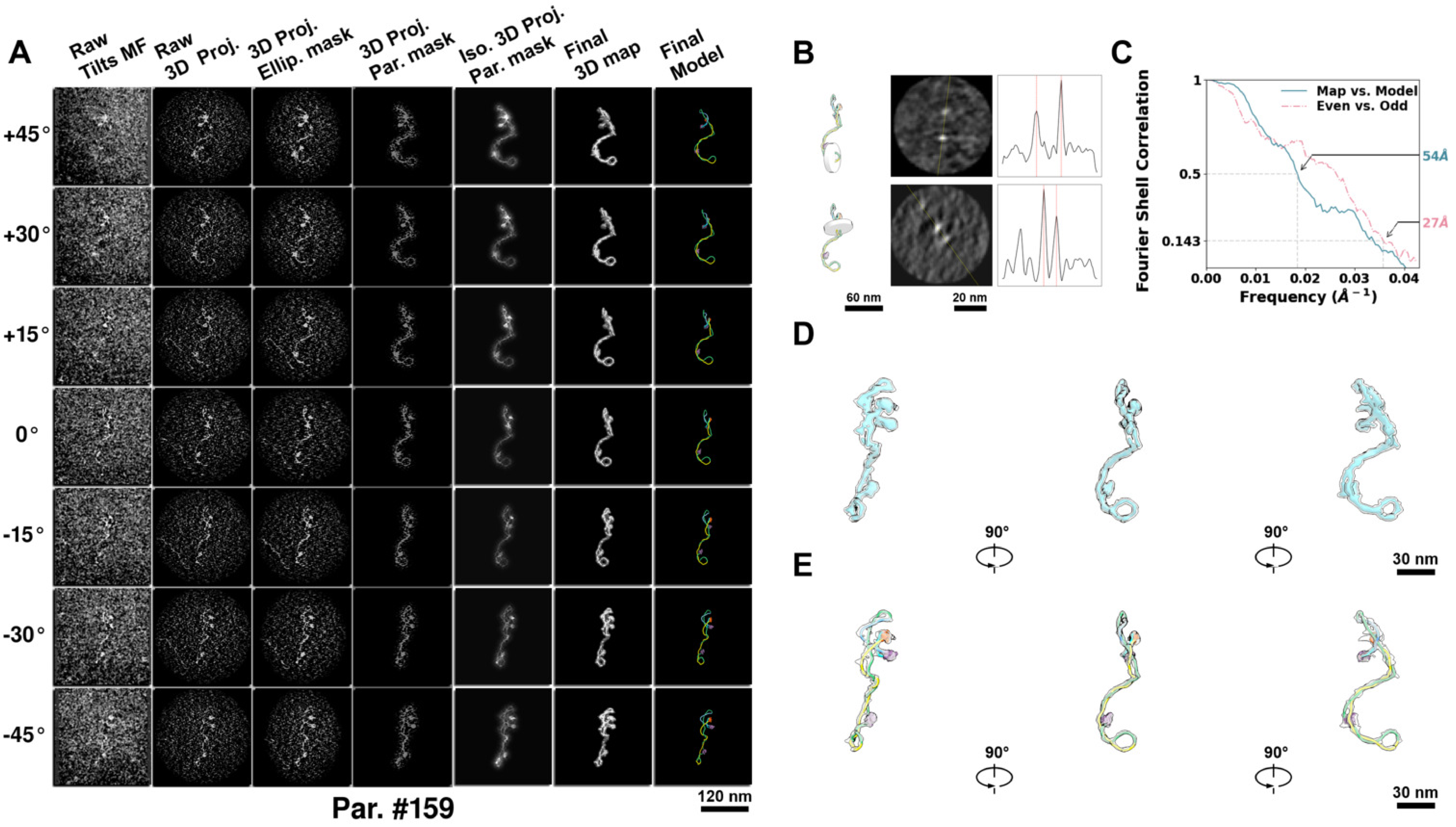

Supplementary Figure 159. Cryo-electron tomography (cryo-ET) 3D reconstruction of an individual plasmid particle in 40 mM K^+, 5 mM Mg^{2+} in the presence of stalled RNAP and dCas9.
(A) 3D reconstruction of the plasmid particle (index no. 159). The first column shows seven representative tilt images from –45° to +45° in step of 15°. The second, third, and fourth columns show 3D projections of the particle with spherical, ellipsoidal (thinner along the z-dimension), and particle-shaped masks, respectively. The fifth column displays the 3D projections of the enhanced and IsoNet missing-wedge-corrected particle. The sixth and seventh columns present the final 3D map and the flexibly fitted model, respectively. (B) Two random cross-sectional views (12 nm thickness) of the plasmid density map along its plectoneme axis are shown in the left-middle panel. The intensity profile along the line crossing the two high-density DNA spots is displayed in the right panel. (C) Resolution assessment of the final 3D map using Fourier shell correlation (FSC). Two criteria are shown: FSC between two half-maps reconstructed from even and odd frames (evaluated at 0.143) and FSC between the final 3D map and the fitted model (evaluated at 0.5). (D) Zoomed-in views of the final 3D density map from panel A, displayed at two contour levels. (E) Superimposition of the high-contour level map from panel D onto its flexibly fitted model.

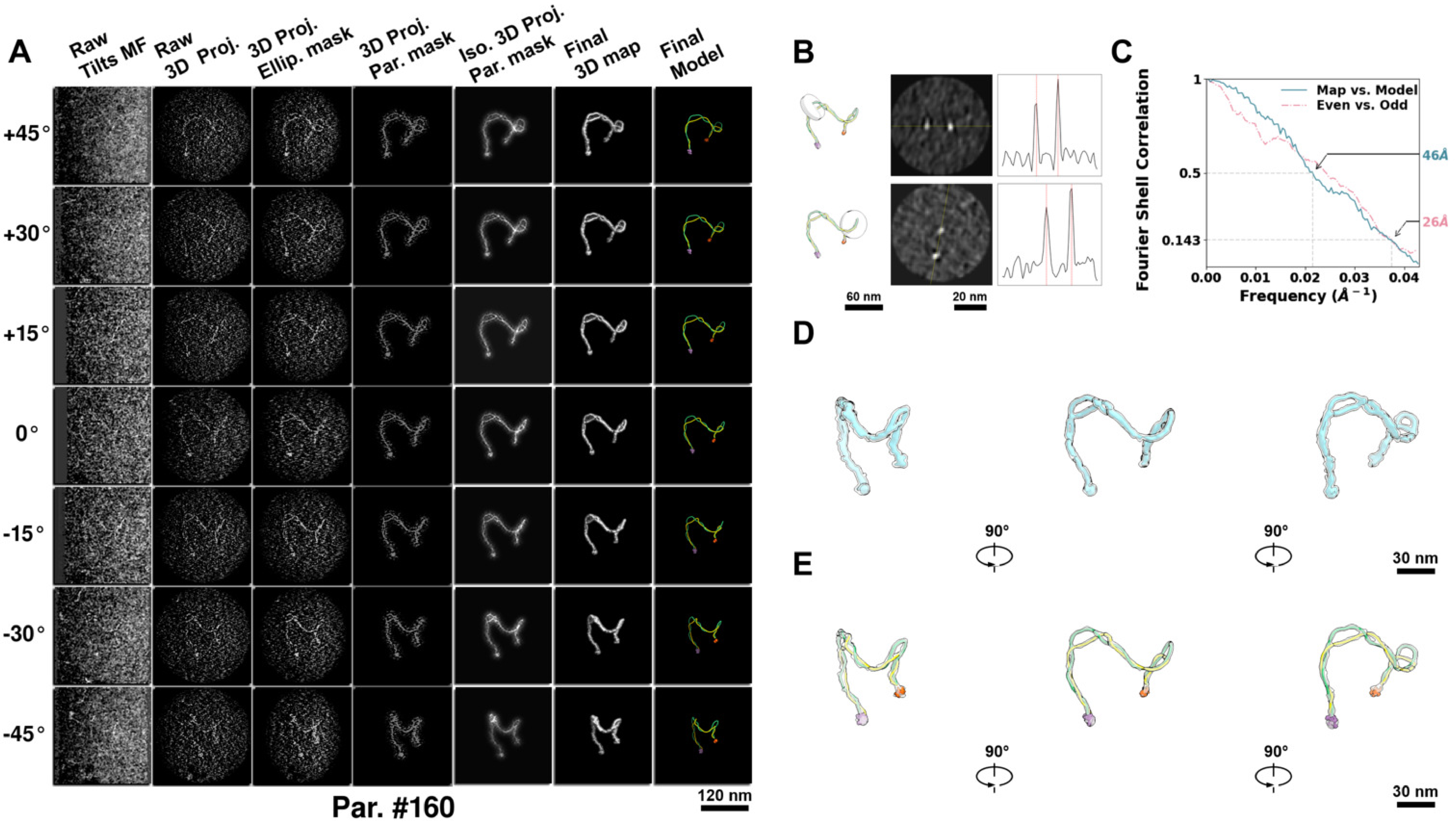

Supplementary Figure 160. Cryo-electron tomography (cryo-ET) 3D reconstruction of an individual plasmid particle in 40 mM K^+, 5 mM Mg^{2+} in the presence of stalled RNAP and dCas9.
(A) 3D reconstruction of the plasmid particle (index no. 160). The first column shows seven representative tilt images from –45° to +45° in step of 15°. The second, third, and fourth columns show 3D projections of the particle with spherical, ellipsoidal (thinner along the z-dimension), and particle-shaped masks, respectively. The fifth column displays the 3D projections of the enhanced and IsoNet missing-wedge-corrected particle. The sixth and seventh columns present the final 3D map and the flexibly fitted model, respectively. (B) Two random cross-sectional views (12 nm thickness) of the plasmid density map along its plectoneme axis are shown in the left-middle panel. The intensity profile along the line crossing the two high-density DNA spots is displayed in the right panel. (C) Resolution assessment of the final 3D map using Fourier shell correlation (FSC). Two criteria are shown: FSC between two half-maps reconstructed from even and odd frames (evaluated at 0.143) and FSC between the final 3D map and the fitted model (evaluated at 0.5). (D) Zoomed-in views of the final 3D density map from panel A, displayed at two contour levels. (E) Superimposition of the high-contour level map from panel D onto its flexibly fitted model.

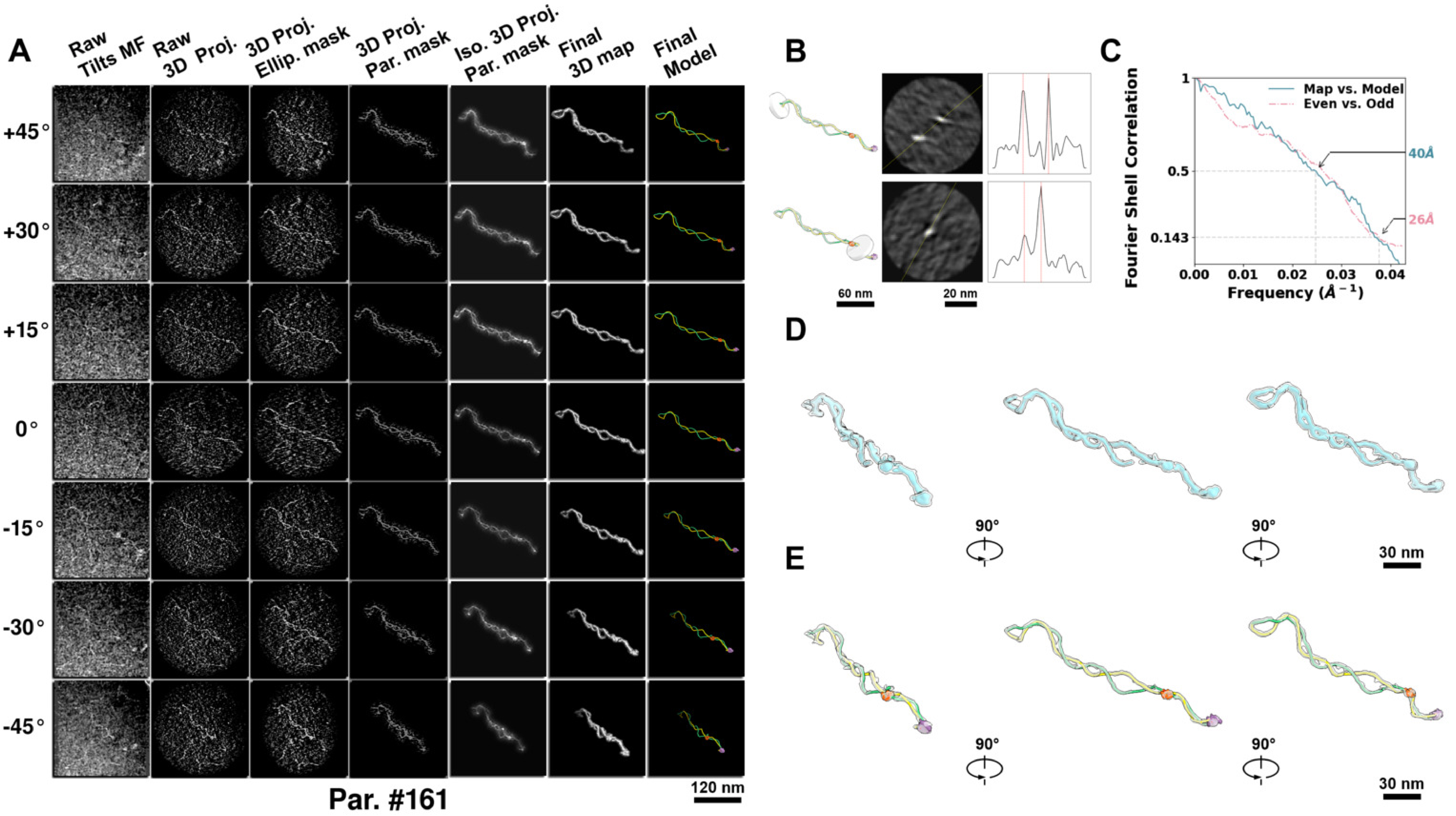

Supplementary Figure 161. Cryo-electron tomography (cryo-ET) 3D reconstruction of an individual plasmid particle in 40 mM K⁺, 5 mM Mg²⁺ in the presence of stalled RNAP and dCas9.
(A) 3D reconstruction of the plasmid particle (index no. 161). The first column shows seven representative tilt images from –45° to +45° in step of 15°. The second, third, and fourth columns show 3D projections of the particle with spherical, ellipsoidal (thinner along the z-dimension), and particle-shaped masks, respectively. The fifth column displays the 3D projections of the enhanced and IsoNet missing-wedge-corrected particle. The sixth and seventh columns present the final 3D map and the flexibly fitted model, respectively. (B) Two random cross-sectional views (12 nm thickness) of the plasmid density map along its plectoneme axis are shown in the left-middle panel. The intensity profile along the line crossing the two high-density DNA spots is displayed in the right panel. (C) Resolution assessment of the final 3D map using Fourier shell correlation (FSC). Two criteria are shown: FSC between two half-maps reconstructed from even and odd frames (evaluated at 0.143) and FSC between the final 3D map and the fitted model (evaluated at 0.5). (D) Zoomed-in views of the final 3D density map from panel A, displayed at two contour levels. (E) Superimposition of the high-contour level map from panel D onto its flexibly fitted model.

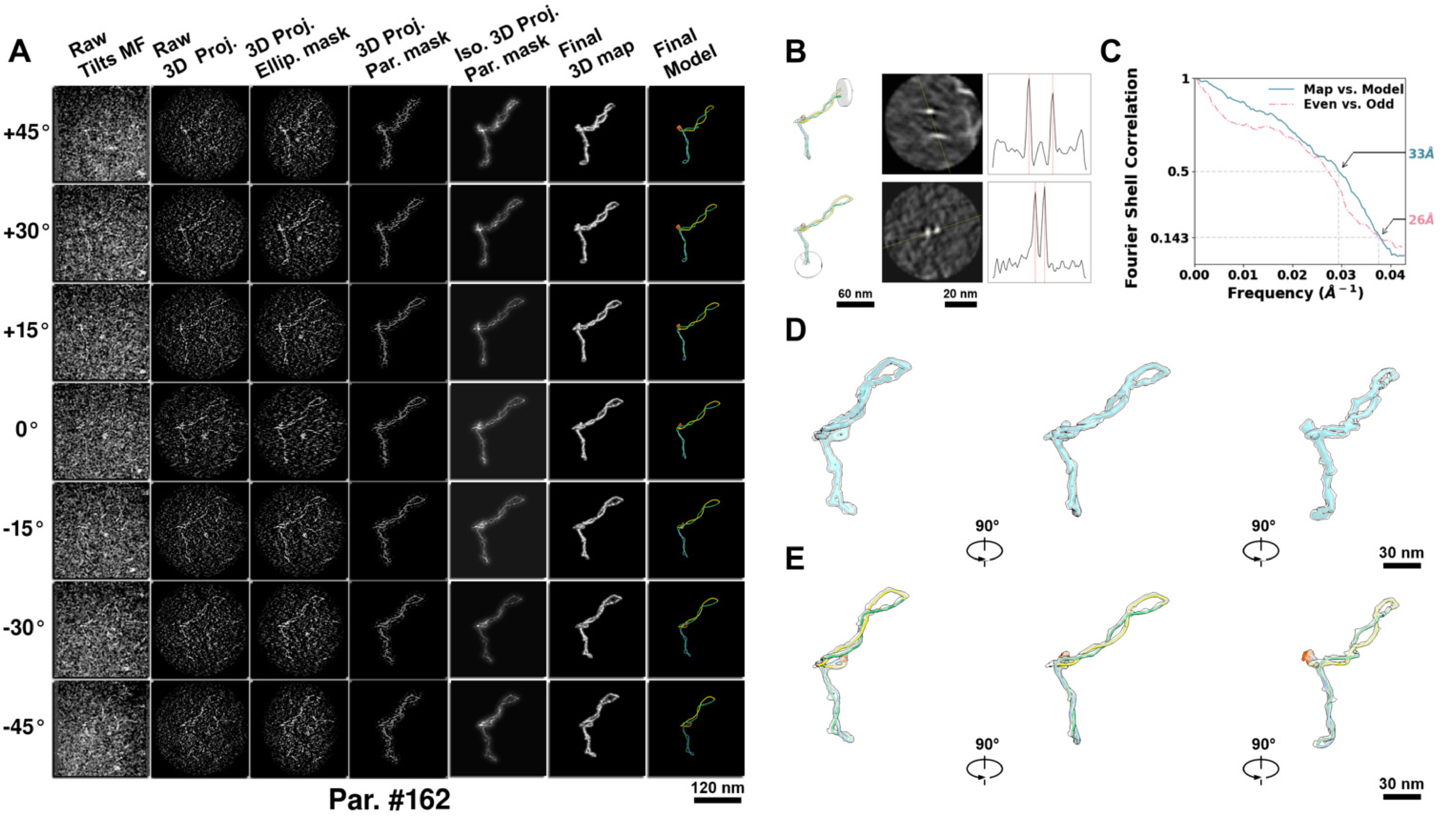

Supplementary Figure 162. Cryo-electron tomography (cryo-ET) 3D reconstruction of an individual plasmid particle in 40 mM K⁺, 5 mM Mg²⁺ in the presence of stalled RNAP and dCas9.
(A) 3D reconstruction of the plasmid particle (index no. 162). The first column shows seven representative tilt images from –45° to +45° in step of 15°. The second, third, and fourth columns show 3D projections of the particle with spherical, ellipsoidal (thinner along the z-dimension), and particle-shaped masks, respectively. The fifth column displays the 3D projections of the enhanced and IsoNet missing-wedge-corrected particle. The sixth and seventh columns present the final 3D map and the flexibly fitted model, respectively. (B) Two random cross-sectional views (12 nm thickness) of the plasmid density map along its plectoneme axis are shown in the left-middle panel. The intensity profile along the line crossing the two high-density DNA spots is displayed in the right panel. (C) Resolution assessment of the final 3D map using Fourier shell correlation (FSC). Two criteria are shown: FSC between two half-maps reconstructed from even and odd frames (evaluated at 0.143) and FSC between the final 3D map and the fitted model (evaluated at 0.5). (D) Zoomed-in views of the final 3D density map from panel A, displayed at two contour levels. (E) Superimposition of the high-contour level map from panel D onto its flexibly fitted model.

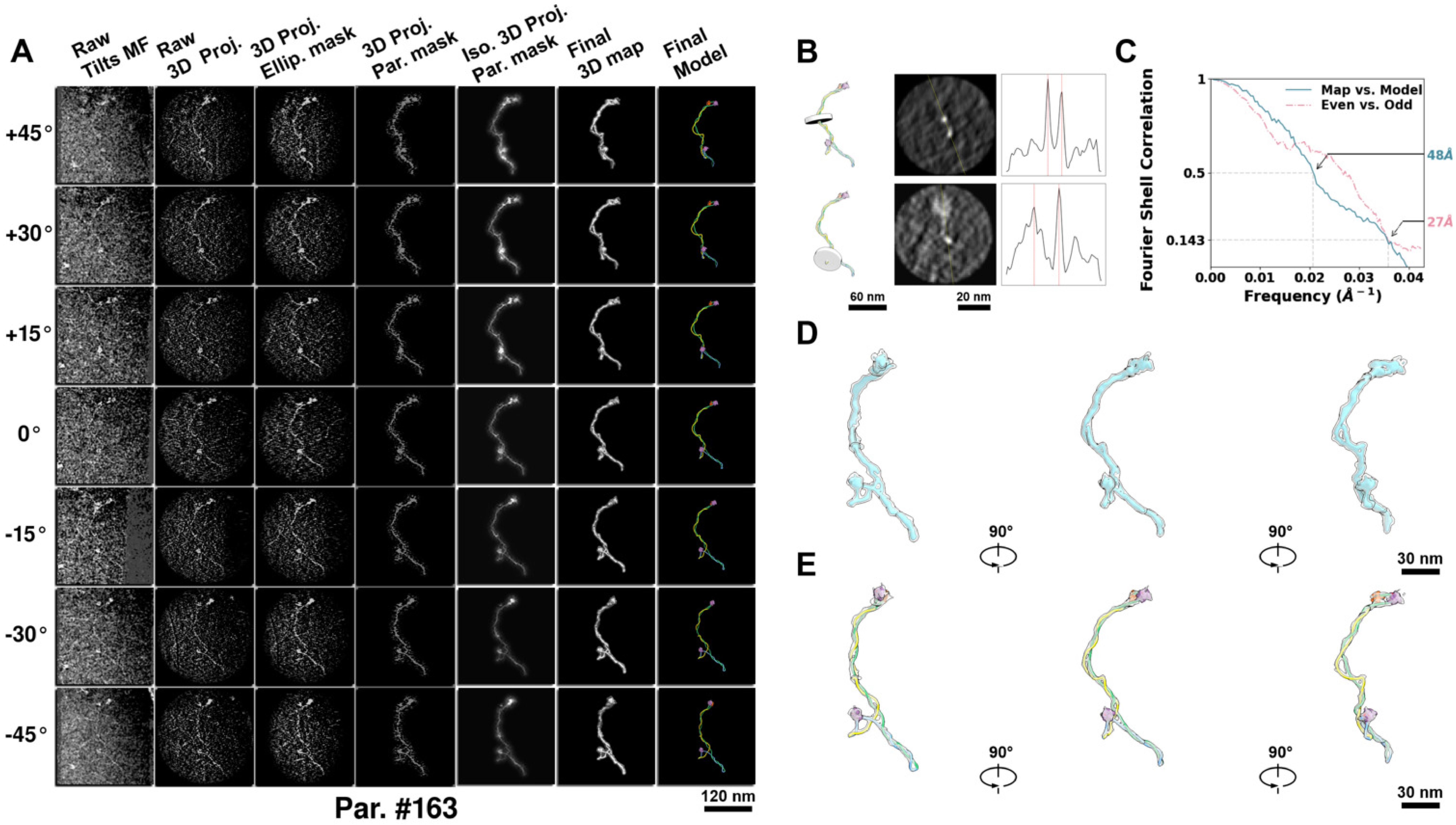

Supplementary Figure 163. Cryo-electron tomography (cryo-ET) 3D reconstruction of an individual plasmid particle in 40 mM K^+, 5 mM Mg^{2+} in the presence of stalled RNAP and dCas9.
(A) 3D reconstruction of the plasmid particle (index no. 163). The first column shows seven representative tilt images from –45° to +45° in step of 15°. The second, third, and fourth columns show 3D projections of the particle with spherical, ellipsoidal (thinner along the z-dimension), and particle-shaped masks, respectively. The fifth column displays the 3D projections of the enhanced and IsoNet missing-wedge-corrected particle. The sixth and seventh columns present the final 3D map and the flexibly fitted model, respectively. (B) Two random cross-sectional views (12 nm thickness) of the plasmid density map along its plectoneme axis are shown in the left-middle panel. The intensity profile along the line crossing the two high-density DNA spots is displayed in the right panel. (C) Resolution assessment of the final 3D map using Fourier shell correlation (FSC). Two criteria are shown: FSC between two half-maps reconstructed from even and odd frames (evaluated at 0.143) and FSC between the final 3D map and the fitted model (evaluated at 0.5). (D) Zoomed-in views of the final 3D density map from panel A, displayed at two contour levels. (E) Superimposition of the high-contour level map from panel D onto its flexibly fitted model.

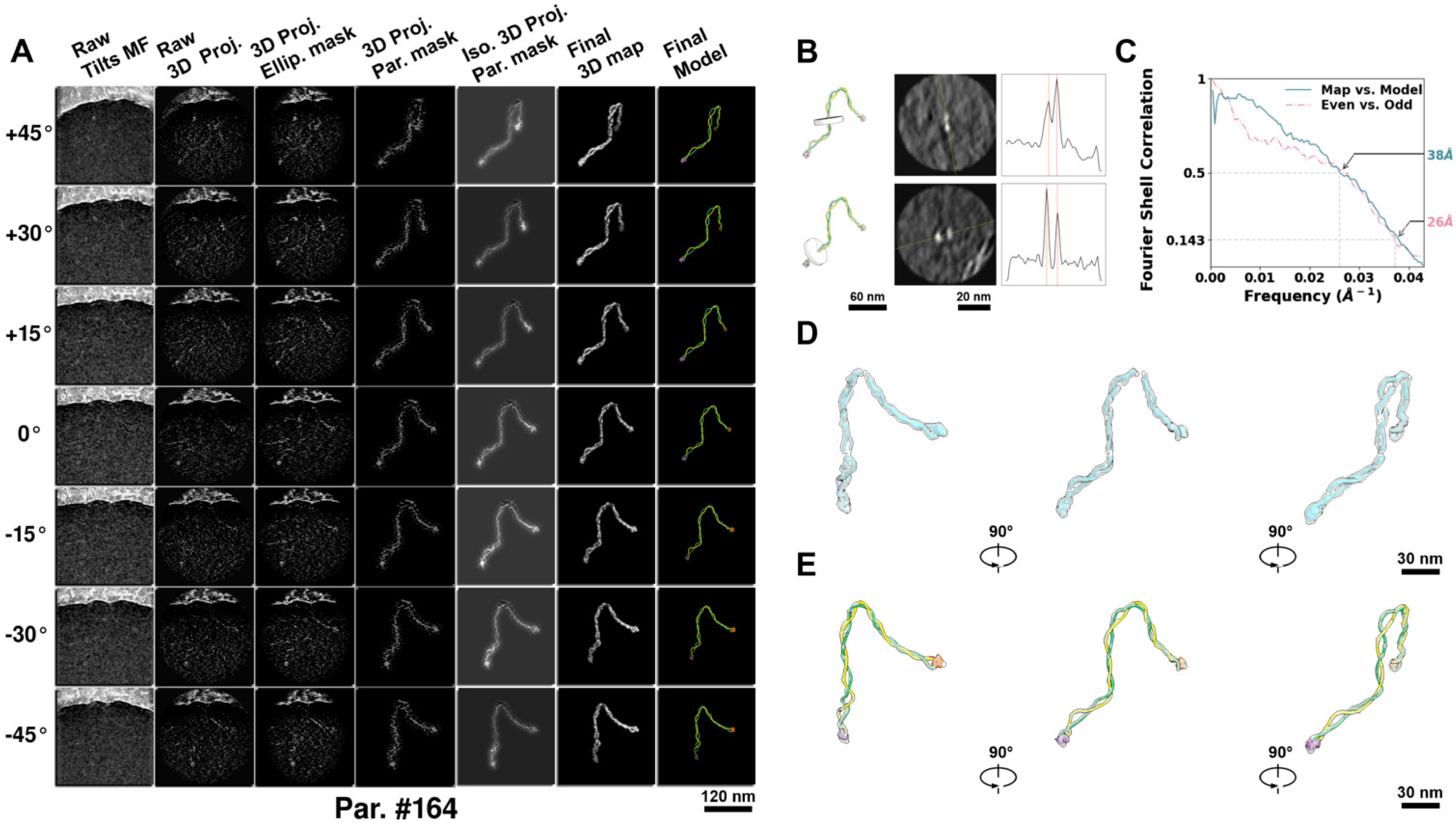

Supplementary Figure 164. Cryo-electron tomography (cryo-ET) 3D reconstruction of an individual plasmid particle in 40 mM K^+, 5 mM Mg^{2+} in the presence of stalled RNAP and dCas9.
(A) 3D reconstruction of the plasmid particle (index no. 164). The first column shows seven representative tilt images from –45° to +45° in step of 15°. The second, third, and fourth columns show 3D projections of the particle with spherical, ellipsoidal (thinner along the z-dimension), and particle-shaped masks, respectively. The fifth column displays the 3D projections of the enhanced and IsoNet missing-wedge-corrected particle. The sixth and seventh columns present the final 3D map and the flexibly fitted model, respectively. (B) Two random cross-sectional views (12 nm thickness) of the plasmid density map along its plectoneme axis are shown in the left-middle panel. The intensity profile along the line crossing the two high-density DNA spots is displayed in the right panel. (C) Resolution assessment of the final 3D map using Fourier shell correlation (FSC). Two criteria are shown: FSC between two half-maps reconstructed from even and odd frames (evaluated at 0.143) and FSC between the final 3D map and the fitted model (evaluated at 0.5). (D) Zoomed-in views of the final 3D density map from panel A, displayed at two contour levels. (E) Superimposition of the high-contour level map from panel D onto its flexibly fitted model.

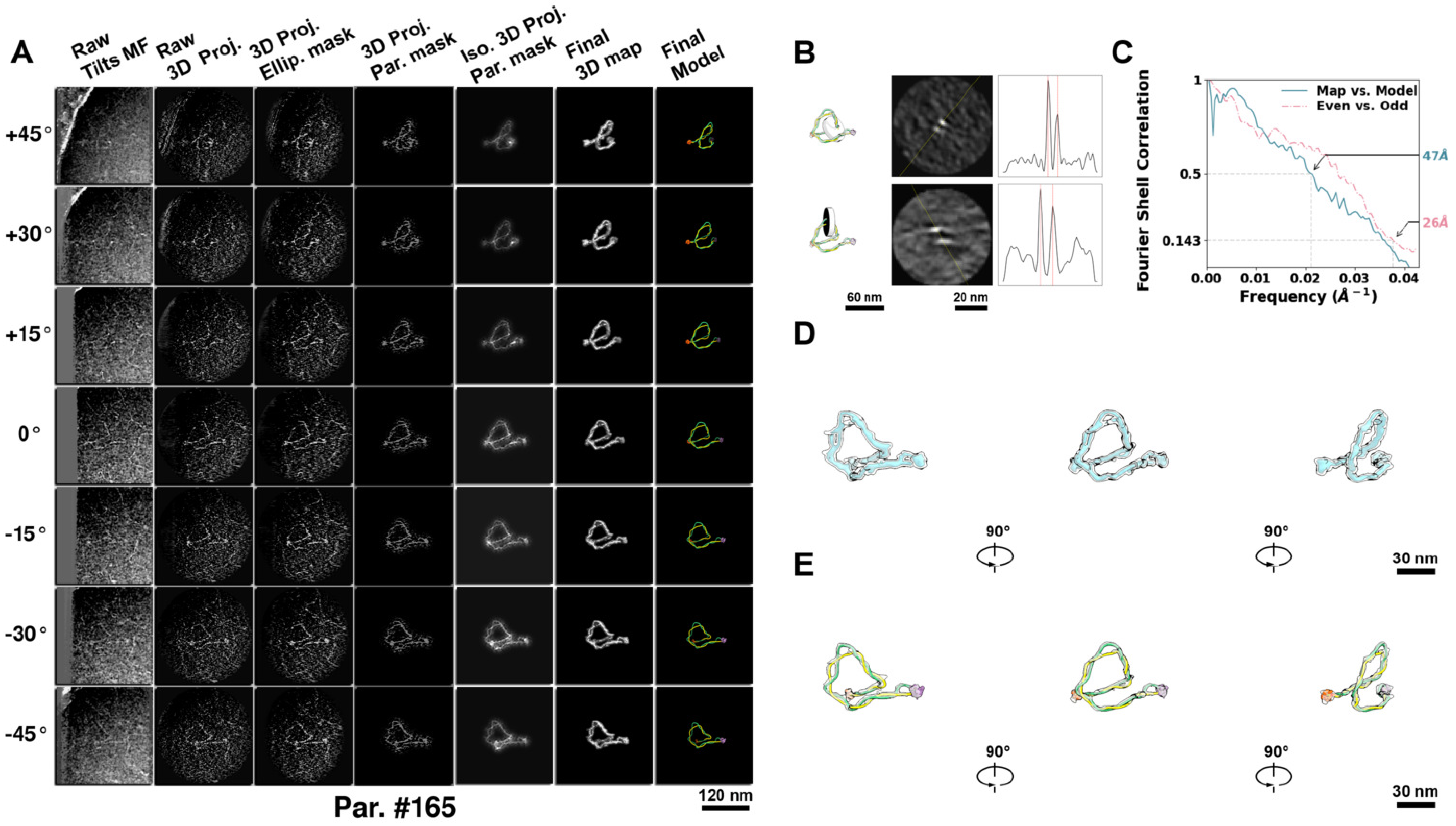

Supplementary Figure 165. Cryo-electron tomography (cryo-ET) 3D reconstruction of an individual plasmid particle in 40 mM K^+, 5 mM Mg^{2+} in the presence of stalled RNAP and dCas9.
(A) 3D reconstruction of the plasmid particle (index no. 165). The first column shows seven representative tilt images from –45° to +45° in step of 15°. The second, third, and fourth columns show 3D projections of the particle with spherical, ellipsoidal (thinner along the z-dimension), and particle-shaped masks, respectively. The fifth column displays the 3D projections of the enhanced and IsoNet missing-wedge-corrected particle. The sixth and seventh columns present the final 3D map and the flexibly fitted model, respectively. (B) Two random cross-sectional views (12 nm thickness) of the plasmid density map along its plectoneme axis are shown in the left-middle panel. The intensity profile along the line crossing the two high-density DNA spots is displayed in the right panel. (C) Resolution assessment of the final 3D map using Fourier shell correlation (FSC). Two criteria are shown: FSC between two half-maps reconstructed from even and odd frames (evaluated at 0.143) and FSC between the final 3D map and the fitted model (evaluated at 0.5). (D) Zoomed-in views of the final 3D density map from panel A, displayed at two contour levels. (E) Superimposition of the high-contour level map from panel D onto its flexibly fitted model.

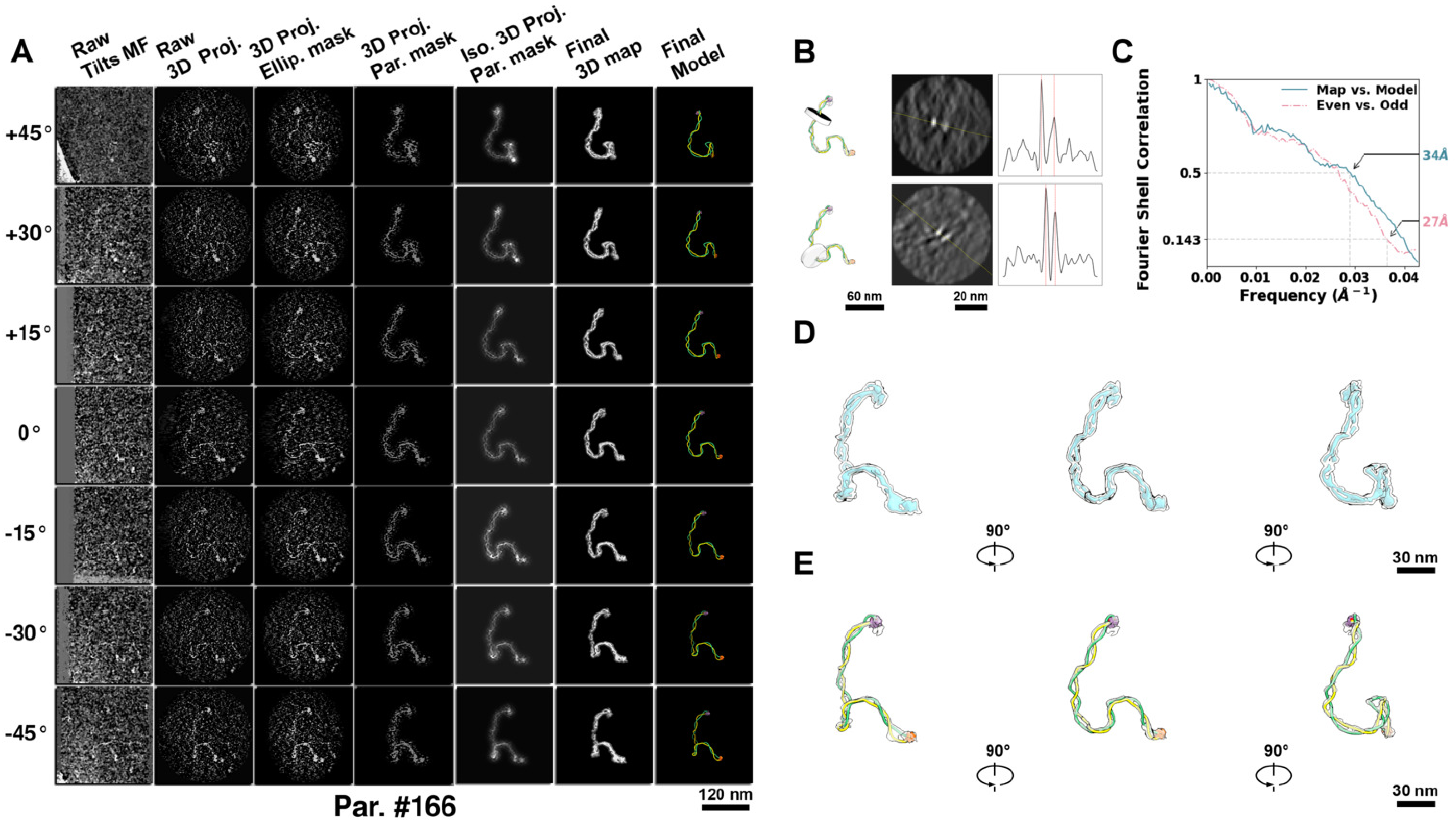

Supplementary Figure 166. Cryo-electron tomography (cryo-ET) 3D reconstruction of an individual plasmid particle in 40 mM K^+, 5 mM Mg^{2+} in the presence of stalled RNAP and dCas9.
(A) 3D reconstruction of the plasmid particle (index no. 166). The first column shows seven representative tilt images from –45° to +45° in step of 15°. The second, third, and fourth columns show 3D projections of the particle with spherical, ellipsoidal (thinner along the z-dimension), and particle-shaped masks, respectively. The fifth column displays the 3D projections of the enhanced and IsoNet missing-wedge-corrected particle. The sixth and seventh columns present the final 3D map and the flexibly fitted model, respectively. (B) Two random cross-sectional views (12 nm thickness) of the plasmid density map along its plectoneme axis are shown in the left-middle panel. The intensity profile along the line crossing the two high-density DNA spots is displayed in the right panel. (C) Resolution assessment of the final 3D map using Fourier shell correlation (FSC). Two criteria are shown: FSC between two half-maps reconstructed from even and odd frames (evaluated at 0.143) and FSC between the final 3D map and the fitted model (evaluated at 0.5). (D) Zoomed-in views of the final 3D density map from panel A, displayed at two contour levels. (E) Superimposition of the high-contour level map from panel D onto its flexibly fitted model.

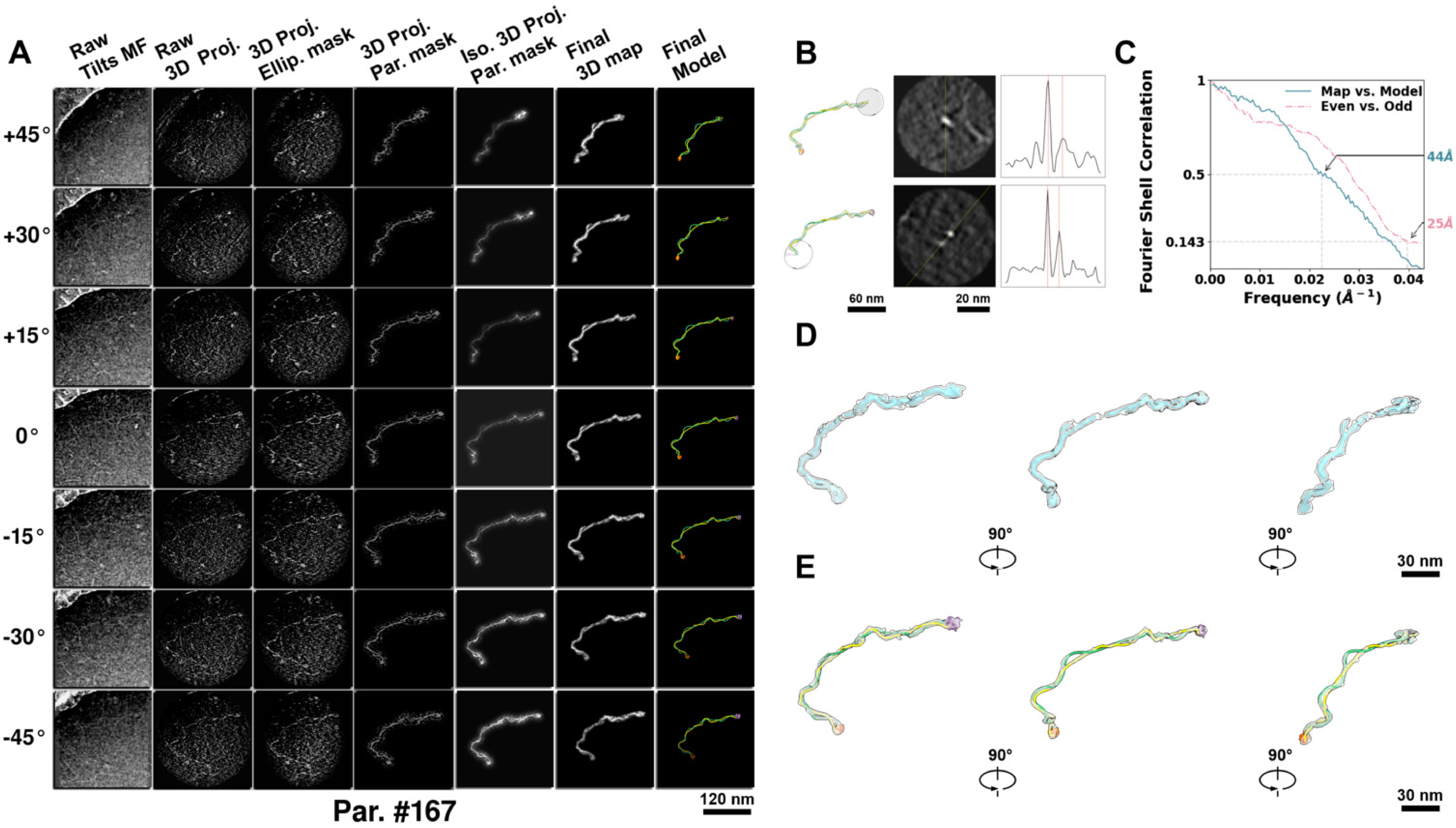

Supplementary Figure 167. Cryo-electron tomography (cryo-ET) 3D reconstruction of an individual plasmid particle in 40 mM K+, 5 mM Mg2+ in the presence of stalled RNAP and dCas9.
(A) 3D reconstruction of the plasmid particle (index no. 167). The first column shows seven representative tilt images from –45° to +45° in step of 15°. The second, third, and fourth columns show 3D projections of the particle with spherical, ellipsoidal (thinner along the z-dimension), and particle-shaped masks, respectively. The fifth column displays the 3D projections of the enhanced and IsoNet missing-wedge-corrected particle. The sixth and seventh columns present the final 3D map and the flexibly fitted model, respectively. (B) Two random cross-sectional views (12 nm thickness) of the plasmid density map along its plectoneme axis are shown in the left-middle panel. The intensity profile along the line crossing the two high-density DNA spots is displayed in the right panel. (C) Resolution assessment of the final 3D map using Fourier shell correlation (FSC). Two criteria are shown: FSC between two half-maps reconstructed from even and odd frames (evaluated at 0.143) and FSC between the final 3D map and the fitted model (evaluated at 0.5). (D) Zoomed-in views of the final 3D density map from panel A, displayed at two contour levels. (E) Superimposition of the high-contour level map from panel D onto its flexibly fitted model.

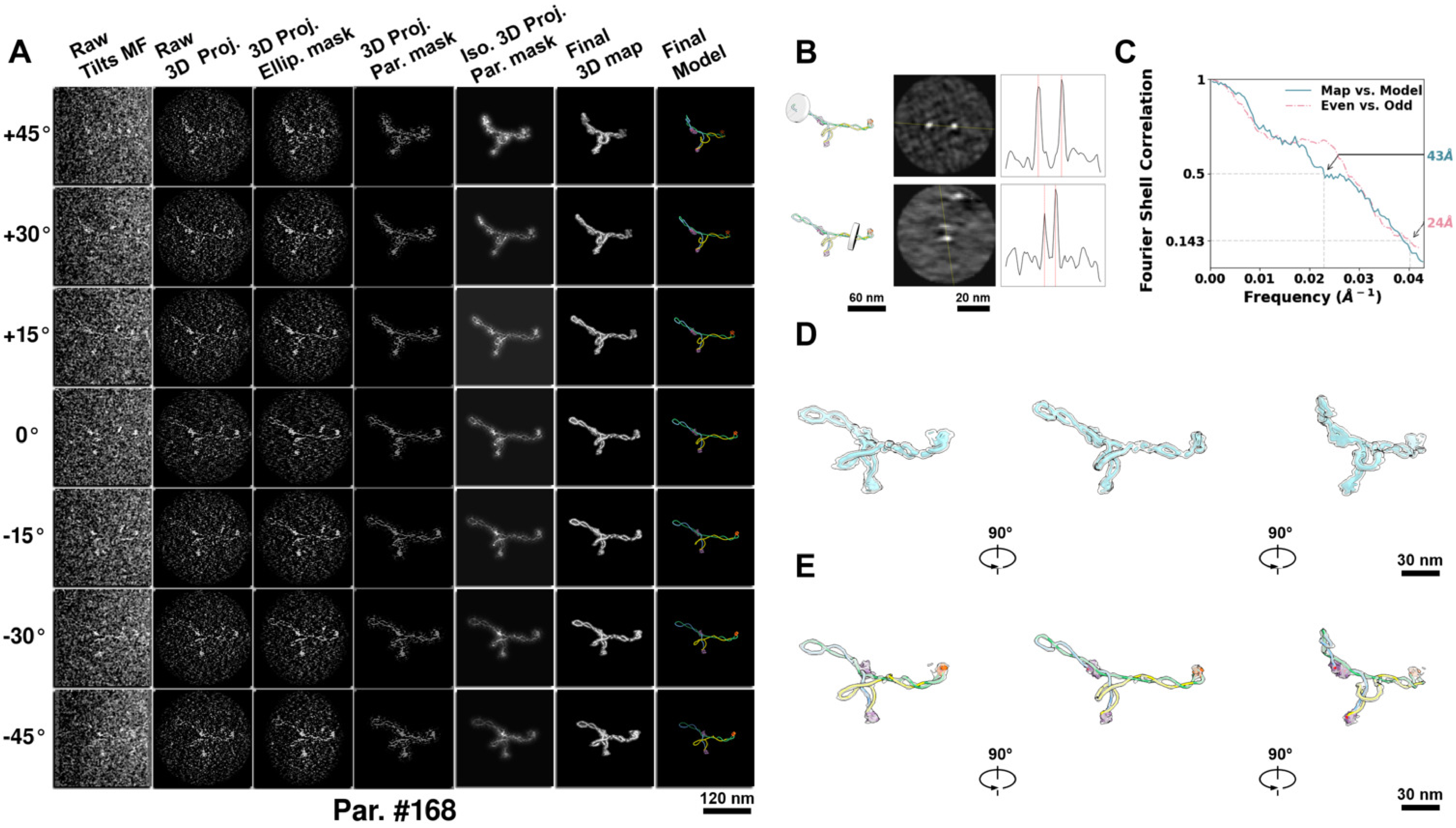

Supplementary Figure 168. Cryo-electron tomography (cryo-ET) 3D reconstruction of an individual plasmid particle in 40 mM K+, 5 mM Mg2+ in the presence of stalled RNAP and dCas9.
(A) 3D reconstruction of the plasmid particle (index no. 168). The first column shows seven representative tilt images from –45° to +45° in step of 15°. The second, third, and fourth columns show 3D projections of the particle with spherical, ellipsoidal (thinner along the z-dimension), and particle-shaped masks, respectively. The fifth column displays the 3D projections of the enhanced and IsoNet missing-wedge-corrected particle. The sixth and seventh columns present the final 3D map and the flexibly fitted model, respectively. (B) Two random cross-sectional views (12 nm thickness) of the plasmid density map along its plectoneme axis are shown in the left-middle panel. The intensity profile along the line crossing the two high-density DNA spots is displayed in the right panel. (C) Resolution assessment of the final 3D map using Fourier shell correlation (FSC). Two criteria are shown: FSC between two half-maps reconstructed from even and odd frames (evaluated at 0.143) and FSC between the final 3D map and the fitted model (evaluated at 0.5). (D) Zoomed-in views of the final 3D density map from panel A, displayed at two contour levels. (E) Superimposition of the high-contour level map from panel D onto its flexibly fitted model.

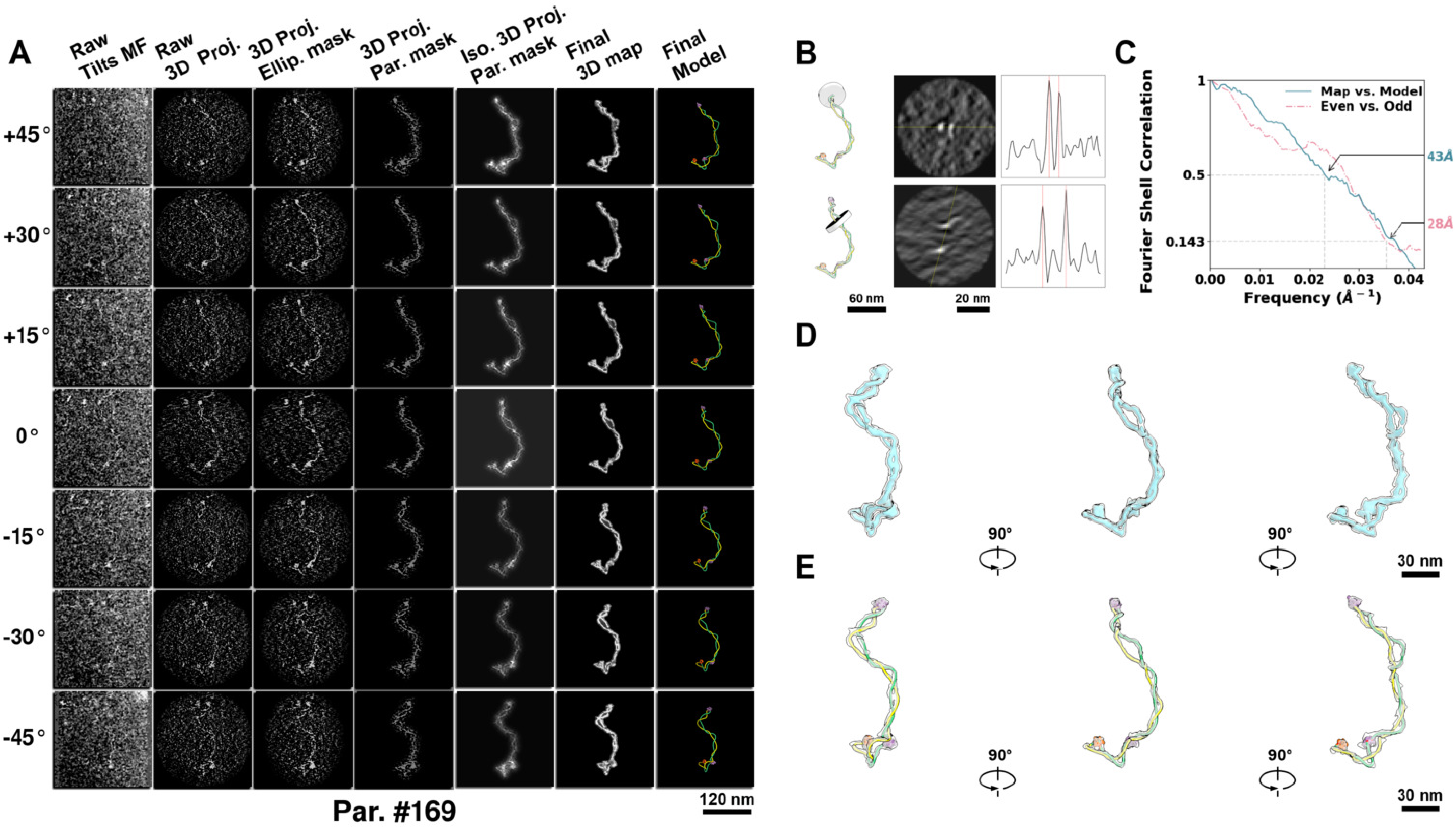

Supplementary Figure 169. Cryo-electron tomography (cryo-ET) 3D reconstruction of an individual plasmid particle in 40 mM K$^+$, 5 mM Mg^{2+} in the presence of stalled RNAP and dCas9.
(A) 3D reconstruction of the plasmid particle (index no. 169). The first column shows seven representative tilt images from –45° to +45° in step of 15°. The second, third, and fourth columns show 3D projections of the particle with spherical, ellipsoidal (thinner along the z-dimension), and particle-shaped masks, respectively. The fifth column displays the 3D projections of the enhanced and IsoNet missing-wedge-corrected particle. The sixth and seventh columns present the final 3D map and the flexibly fitted model, respectively. (B) Two random cross-sectional views (12 nm thickness) of the plasmid density map along its plectoneme axis are shown in the left-middle panel. The intensity profile along the line crossing the two high-density DNA spots is displayed in the right panel. (C) Resolution assessment of the final 3D map using Fourier shell correlation (FSC). Two criteria are shown: FSC between two half-maps reconstructed from even and odd frames (evaluated at 0.143) and FSC between the final 3D map and the fitted model (evaluated at 0.5). (D) Zoomed-in views of the final 3D density map from panel A, displayed at two contour levels. (E) Superimposition of the high-contour level map from panel D onto its flexibly fitted model.

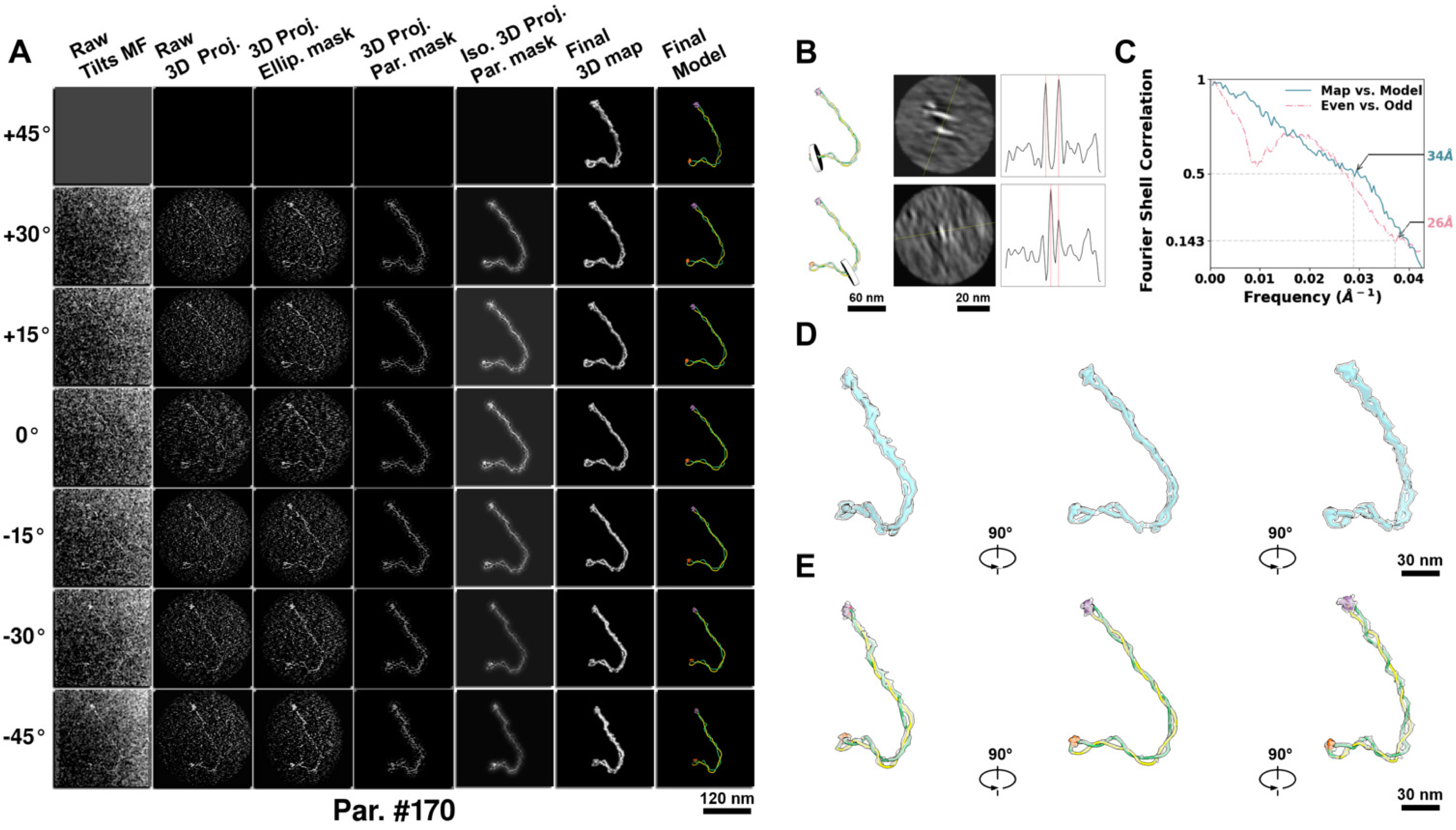

Supplementary Figure 170. Cryo-electron tomography (cryo-ET) 3D reconstruction of an individual plasmid particle in 40 mM K$^+$, 5 mM Mg^{2+} in the presence of stalled RNAP and dCas9.
(A) 3D reconstruction of the plasmid particle (index no. 170). The first column shows seven representative tilt images from –45° to +45° in step of 15°. The second, third, and fourth columns show 3D projections of the particle with spherical, ellipsoidal (thinner along the z-dimension), and particle-shaped masks, respectively. The fifth column displays the 3D projections of the enhanced and IsoNet missing-wedge-corrected particle. The sixth and seventh columns present the final 3D map and the flexibly fitted model, respectively. (B) Two random cross-sectional views (12 nm thickness) of the plasmid density map along its plectoneme axis are shown in the left-middle panel. The intensity profile along the line crossing the two high-density DNA spots is displayed in the right panel. (C) Resolution assessment of the final 3D map using Fourier shell correlation (FSC). Two criteria are shown: FSC between two half-maps reconstructed from even and odd frames (evaluated at 0.143) and FSC between the final 3D map and the fitted model (evaluated at 0.5). (D) Zoomed-in views of the final 3D density map from panel A, displayed at two contour levels. (E) Superimposition of the high-contour level map from panel D onto its flexibly fitted model.

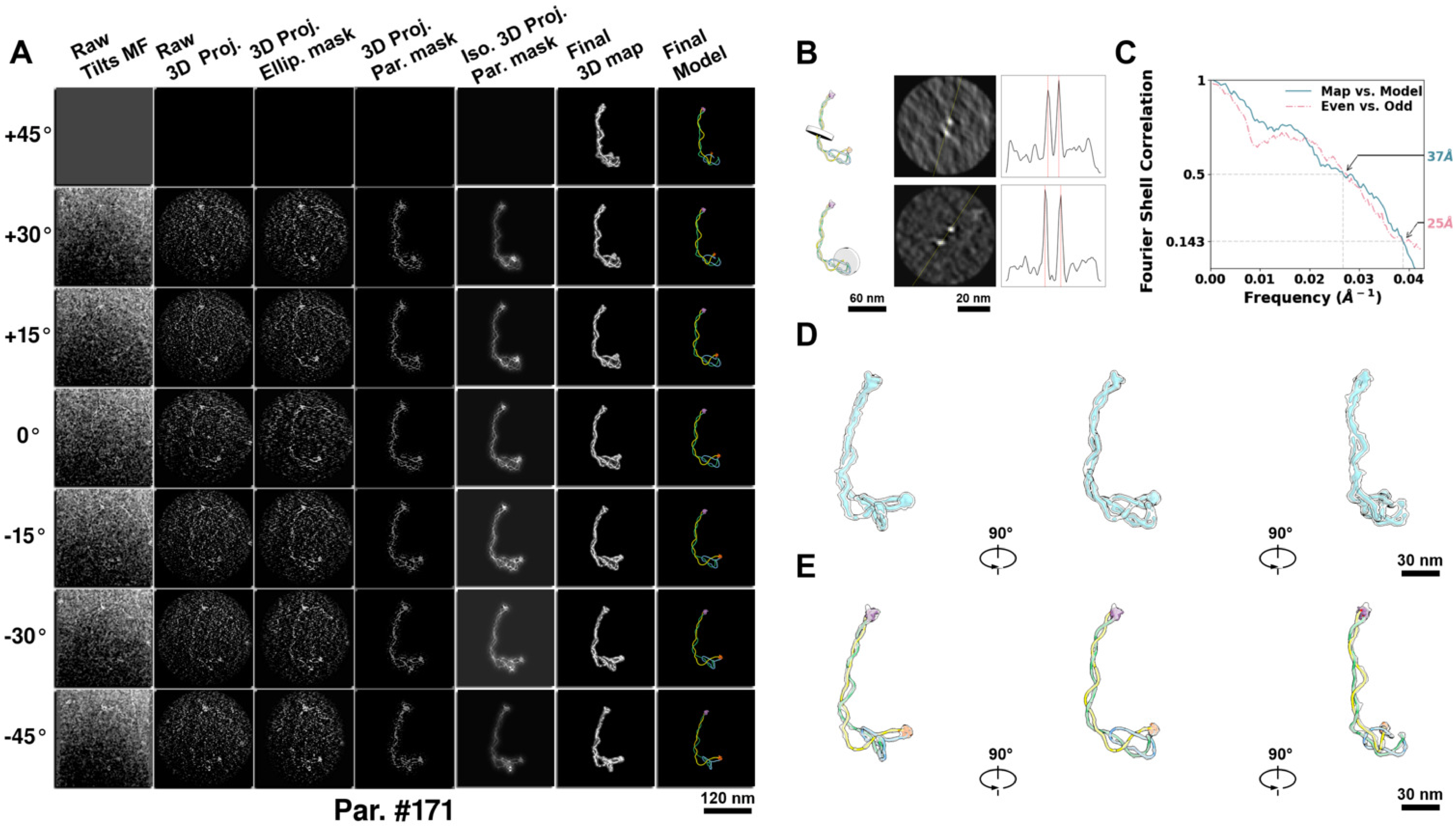

Supplementary Figure 171. Cryo-electron tomography (cryo-ET) 3D reconstruction of an individual plasmid particle in 40 mM K+, 5 mM Mg2+ in the presence of stalled RNAP and dCas9.
(A) 3D reconstruction of the plasmid particle (index no. 171). The first column shows seven representative tilt images from –45° to +45° in step of 15°. The second, third, and fourth columns show 3D projections of the particle with spherical, ellipsoidal (thinner along the z-dimension), and particle-shaped masks, respectively. The fifth column displays the 3D projections of the enhanced and IsoNet missing-wedge-corrected particle. The sixth and seventh columns present the final 3D map and the flexibly fitted model, respectively. (B) Two random cross-sectional views (12 nm thickness) of the plasmid density map along its plectoneme axis are shown in the left-middle panel. The intensity profile along the line crossing the two high-density DNA spots is displayed in the right panel. (C) Resolution assessment of the final 3D map using Fourier shell correlation (FSC). Two criteria are shown: FSC between two half-maps reconstructed from even and odd frames (evaluated at 0.143) and FSC between the final 3D map and the fitted model (evaluated at 0.5). (D) Zoomed-in views of the final 3D density map from panel A, displayed at two contour levels. (E) Superimposition of the high-contour level map from panel D onto its flexibly fitted model.

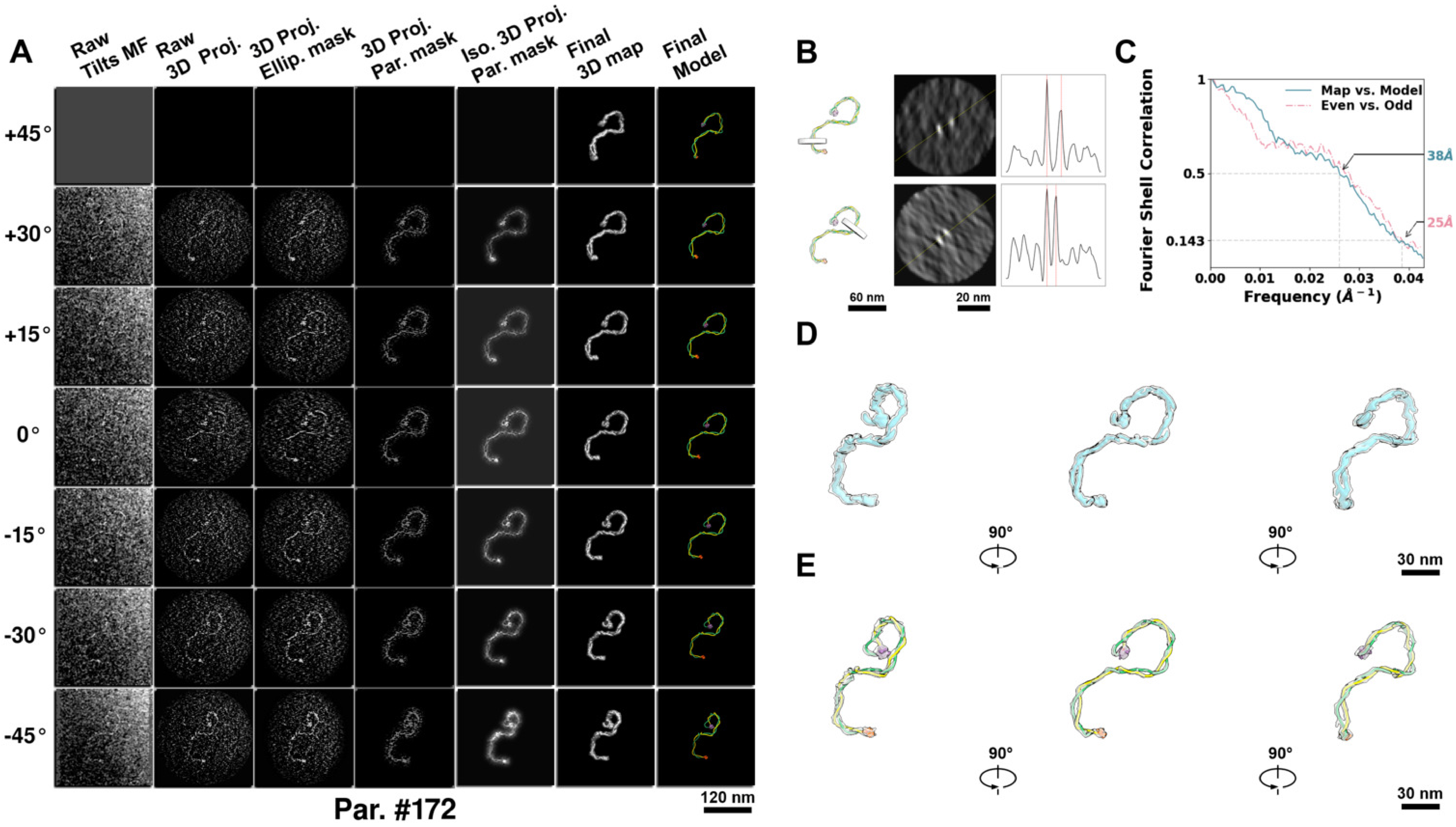

Supplementary Figure 172. Cryo-electron tomography (cryo-ET) 3D reconstruction of an individual plasmid particle in 40 mM K+, 5 mM Mg2+ in the presence of stalled RNAP and dCas9.
(A) 3D reconstruction of the plasmid particle (index no. 172). The first column shows seven representative tilt images from –45° to +45° in step of 15°. The second, third, and fourth columns show 3D projections of the particle with spherical, ellipsoidal (thinner along the z-dimension), and particle-shaped masks, respectively. The fifth column displays the 3D projections of the enhanced and IsoNet missing-wedge-corrected particle. The sixth and seventh columns present the final 3D map and the flexibly fitted model, respectively. (B) Two random cross-sectional views (12 nm thickness) of the plasmid density map along its plectoneme axis are shown in the left-middle panel. The intensity profile along the line crossing the two high-density DNA spots is displayed in the right panel. (C) Resolution assessment of the final 3D map using Fourier shell correlation (FSC). Two criteria are shown: FSC between two half-maps reconstructed from even and odd frames (evaluated at 0.143) and FSC between the final 3D map and the fitted model (evaluated at 0.5). (D) Zoomed-in views of the final 3D density map from panel A, displayed at two contour levels. (E) Superimposition of the high-contour level map from panel D onto its flexibly fitted model.

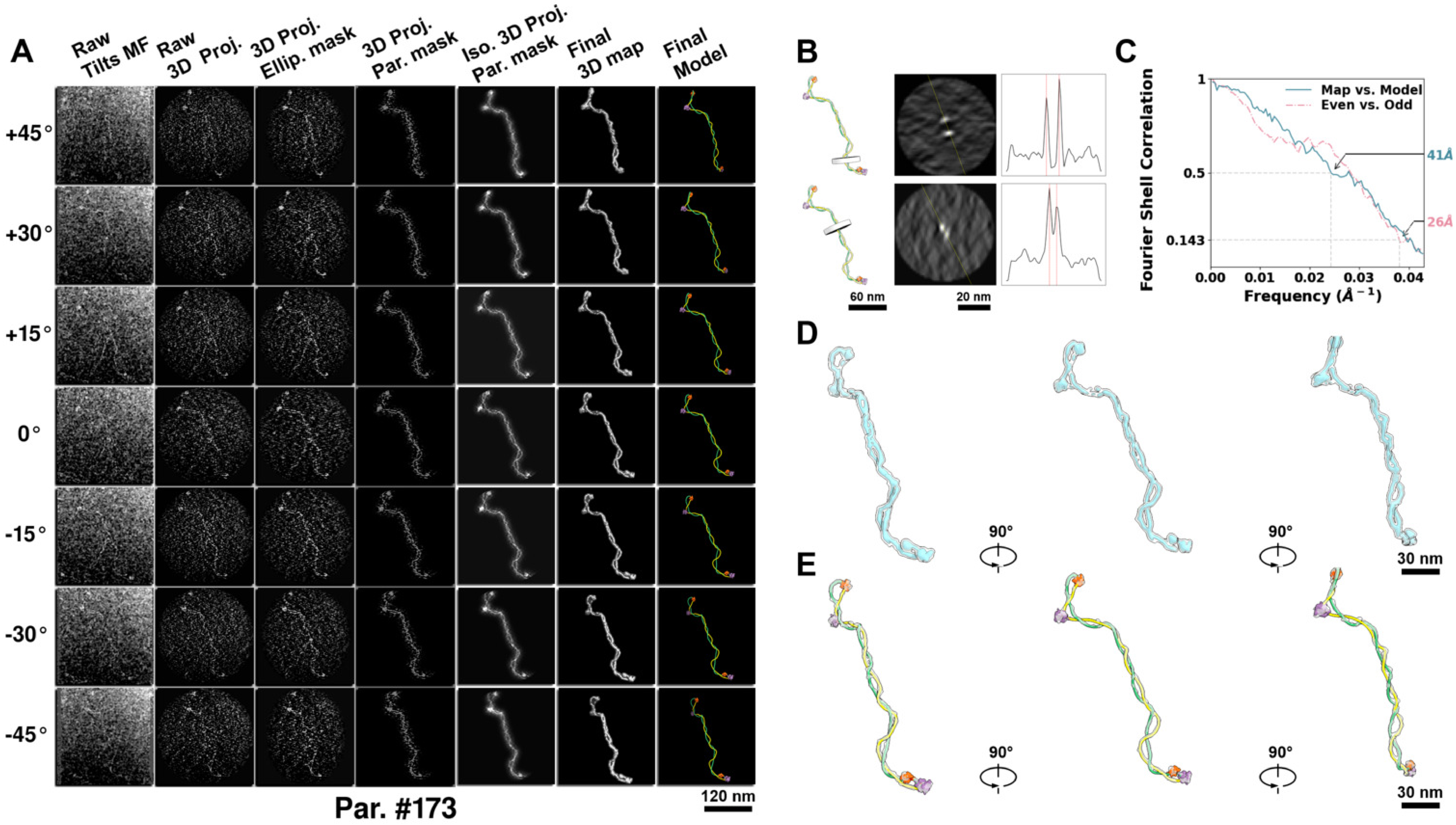

Supplementary Figure 173. Cryo-electron tomography (cryo-ET) 3D reconstruction of an individual plasmid particle in 40 mM K⁺, 5 mM Mg²⁺ in the presence of stalled RNAP and dCas9.
(A) 3D reconstruction of the plasmid particle (index no. 173). The first column shows seven representative tilt images from –45° to +45° in step of 15°. The second, third, and fourth columns show 3D projections of the particle with spherical, ellipsoidal (thinner along the z-dimension), and particle-shaped masks, respectively. The fifth column displays the 3D projections of the enhanced and IsoNet missing-wedge-corrected particle. The sixth and seventh columns present the final 3D map and the flexibly fitted model, respectively. (B) Two random cross-sectional views (12 nm thickness) of the plasmid density map along its plectoneme axis are shown in the left-middle panel. The intensity profile along the line crossing the two high-density DNA spots is displayed in the right panel. (C) Resolution assessment of the final 3D map using Fourier shell correlation (FSC). Two criteria are shown: FSC between two half-maps reconstructed from even and odd frames (evaluated at 0.143) and FSC between the final 3D map and the fitted model (evaluated at 0.5). (D) Zoomed-in views of the final 3D density map from panel A, displayed at two contour levels. (E) Superimposition of the high-contour level map from panel D onto its flexibly fitted model.

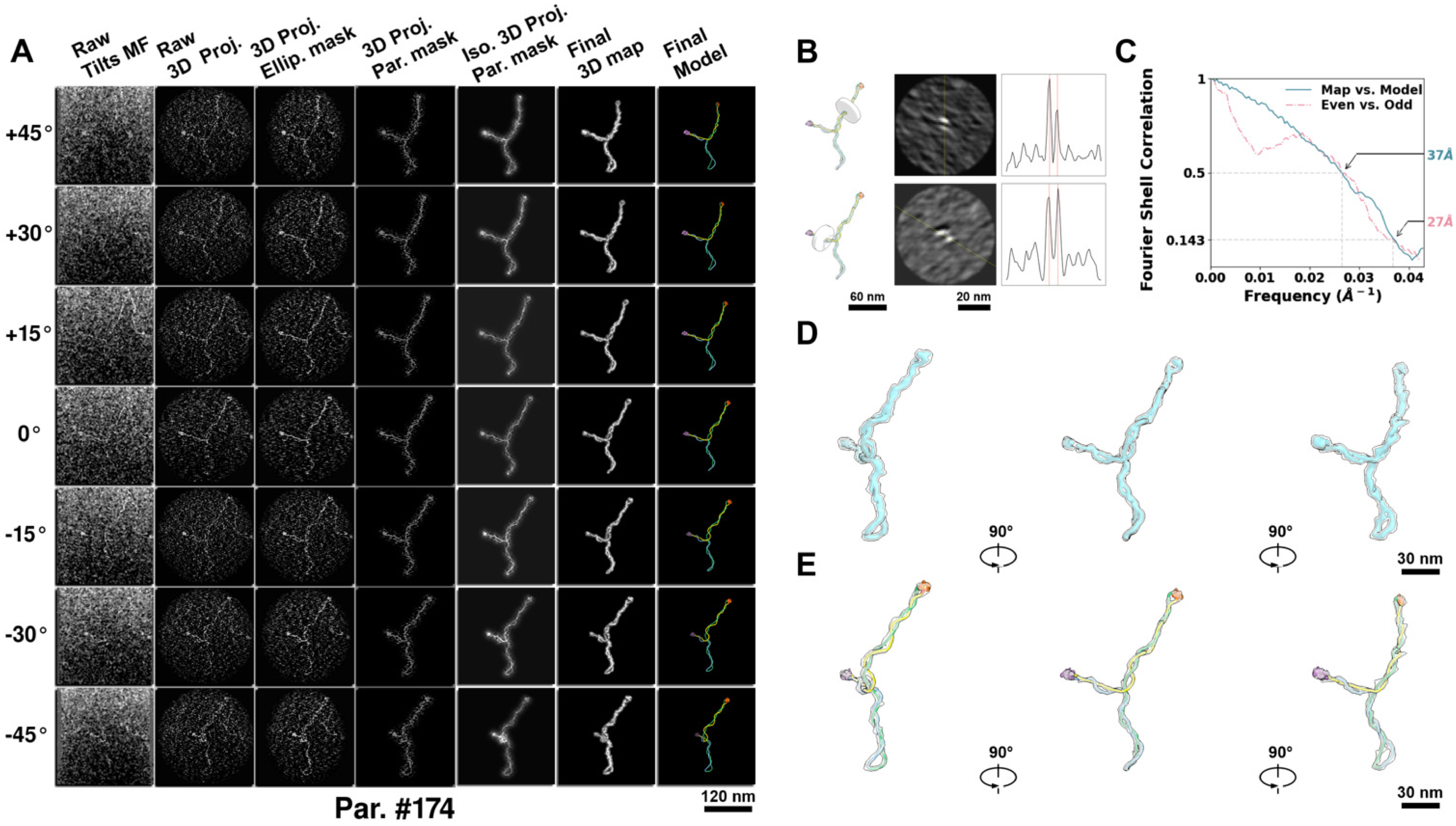

Supplementary Figure 174. Cryo-electron tomography (cryo-ET) 3D reconstruction of an individual plasmid particle in 40 mM K⁺, 5 mM Mg²⁺ in the presence of stalled RNAP and dCas9.
(A) 3D reconstruction of the plasmid particle (index no. 174). The first column shows seven representative tilt images from –45° to +45° in step of 15°. The second, third, and fourth columns show 3D projections of the particle with spherical, ellipsoidal (thinner along the z-dimension), and particle-shaped masks, respectively. The fifth column displays the 3D projections of the enhanced and IsoNet missing-wedge-corrected particle. The sixth and seventh columns present the final 3D map and the flexibly fitted model, respectively. (B) Two random cross-sectional views (12 nm thickness) of the plasmid density map along its plectoneme axis are shown in the left-middle panel. The intensity profile along the line crossing the two high-density DNA spots is displayed in the right panel. (C) Resolution assessment of the final 3D map using Fourier shell correlation (FSC). Two criteria are shown: FSC between two half-maps reconstructed from even and odd frames (evaluated at 0.143) and FSC between the final 3D map and the fitted model (evaluated at 0.5). (D) Zoomed-in views of the final 3D density map from panel A, displayed at two contour levels. (E) Superimposition of the high-contour level map from panel D onto its flexibly fitted model.

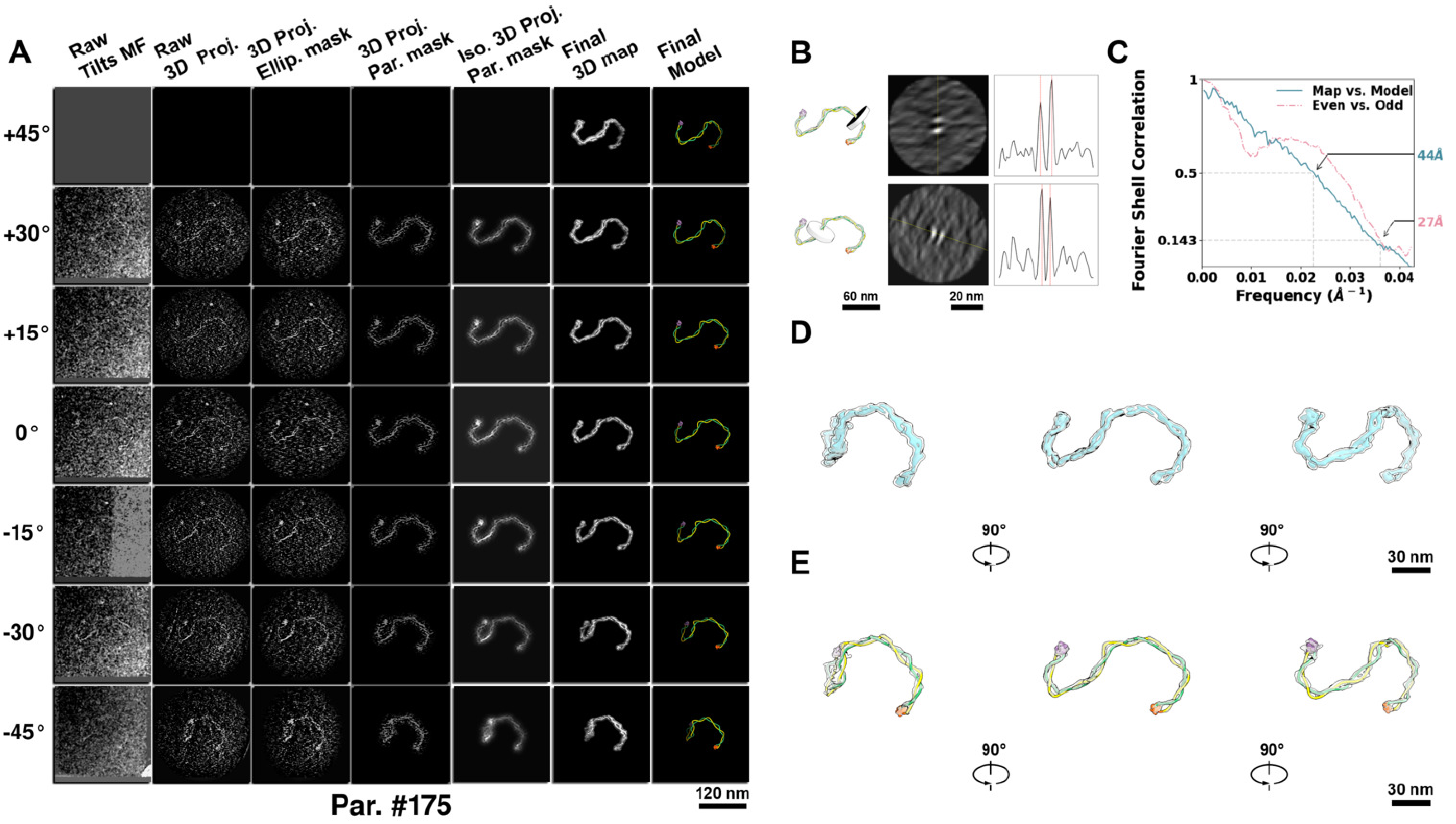

Supplementary Figure 175. Cryo-electron tomography (cryo-ET) 3D reconstruction of an individual plasmid particle in 40 mM K⁺, 5 mM Mg²⁺ in the presence of stalled RNAP and dCas9.
(A) 3D reconstruction of the plasmid particle (index no. 175). The first column shows seven representative tilt images from –45° to +45° in step of 15°. The second, third, and fourth columns show 3D projections of the particle with spherical, ellipsoidal (thinner along the z-dimension), and particle-shaped masks, respectively. The fifth column displays the 3D projections of the enhanced and IsoNet missing-wedge-corrected particle. The sixth and seventh columns present the final 3D map and the flexibly fitted model, respectively. (B) Two random cross-sectional views (12 nm thickness) of the plasmid density map along its plectoneme axis are shown in the left-middle panel. The intensity profile along the line crossing the two high-density DNA spots is displayed in the right panel. (C) Resolution assessment of the final 3D map using Fourier shell correlation (FSC). Two criteria are shown: FSC between two half-maps reconstructed from even and odd frames (evaluated at 0.143) and FSC between the final 3D map and the fitted model (evaluated at 0.5). (D) Zoomed-in views of the final 3D density map from panel A, displayed at two contour levels. (E) Superimposition of the high-contour level map from panel D onto its flexibly fitted model.

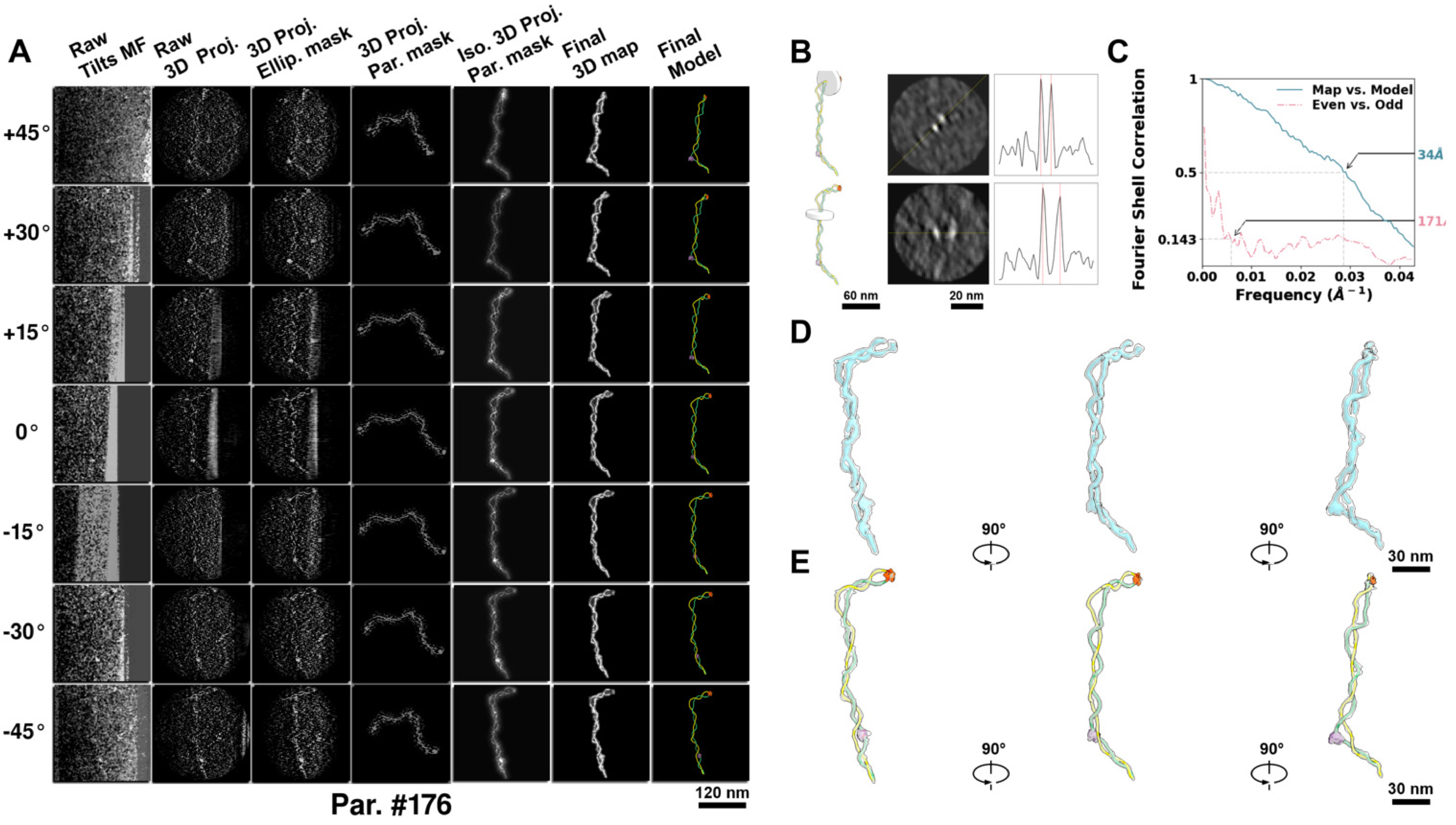

Supplementary Figure 176. Cryo-electron tomography (cryo-ET) 3D reconstruction of an individual plasmid particle in 40 mM K⁺, 5 mM Mg²⁺ in the presence of stalled RNAP and dCas9.
(A) 3D reconstruction of the plasmid particle (index no. 176). The first column shows seven representative tilt images from –45° to +45° in step of 15°. The second, third, and fourth columns show 3D projections of the particle with spherical, ellipsoidal (thinner along the z-dimension), and particle-shaped masks, respectively. The fifth column displays the 3D projections of the enhanced and IsoNet missing-wedge-corrected particle. The sixth and seventh columns present the final 3D map and the flexibly fitted model, respectively. (B) Two random cross-sectional views (12 nm thickness) of the plasmid density map along its plectoneme axis are shown in the left-middle panel. The intensity profile along the line crossing the two high-density DNA spots is displayed in the right panel. (C) Resolution assessment of the final 3D map using Fourier shell correlation (FSC). Two criteria are shown: FSC between two half-maps reconstructed from even and odd frames (evaluated at 0.143) and FSC between the final 3D map and the fitted model (evaluated at 0.5). (D) Zoomed-in views of the final 3D density map from panel A, displayed at two contour levels. (E) Superimposition of the high-contour level map from panel D onto its flexibly fitted model.

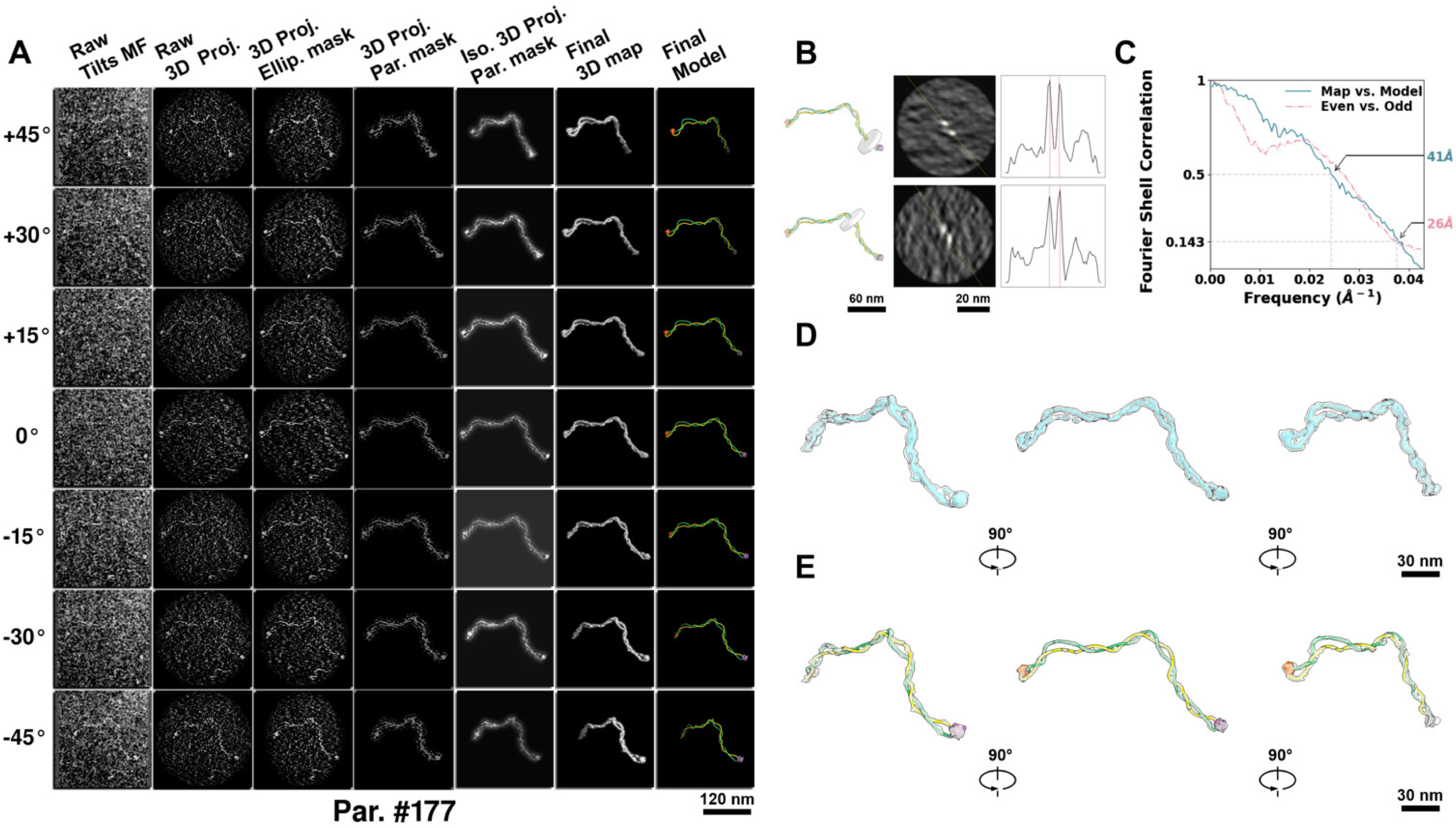

Supplementary Figure 177. Cryo-electron tomography (cryo-ET) 3D reconstruction of an individual plasmid particle in 40 mM K⁺, 5 mM Mg²⁺ in the presence of stalled RNAP and dCas9.
(A) 3D reconstruction of the plasmid particle (index no. 177). The first column shows seven representative tilt images from –45° to +45° in step of 15°. The second, third, and fourth columns show 3D projections of the particle with spherical, ellipsoidal (thinner along the z-dimension), and particle-shaped masks, respectively. The fifth column displays the 3D projections of the enhanced and IsoNet missing-wedge-corrected particle. The sixth and seventh columns present the final 3D map and the flexibly fitted model, respectively. (B) Two random cross-sectional views (12 nm thickness) of the plasmid density map along its plectoneme axis are shown in the left-middle panel. The intensity profile along the line crossing the two high-density DNA spots is displayed in the right panel. (C) Resolution assessment of the final 3D map using Fourier shell correlation (FSC). Two criteria are shown: FSC between two half-maps reconstructed from even and odd frames (evaluated at 0.143) and FSC between the final 3D map and the fitted model (evaluated at 0.5). (D) Zoomed-in views of the final 3D density map from panel A, displayed at two contour levels. (E) Superimposition of the high-contour level map from panel D onto its flexibly fitted model.

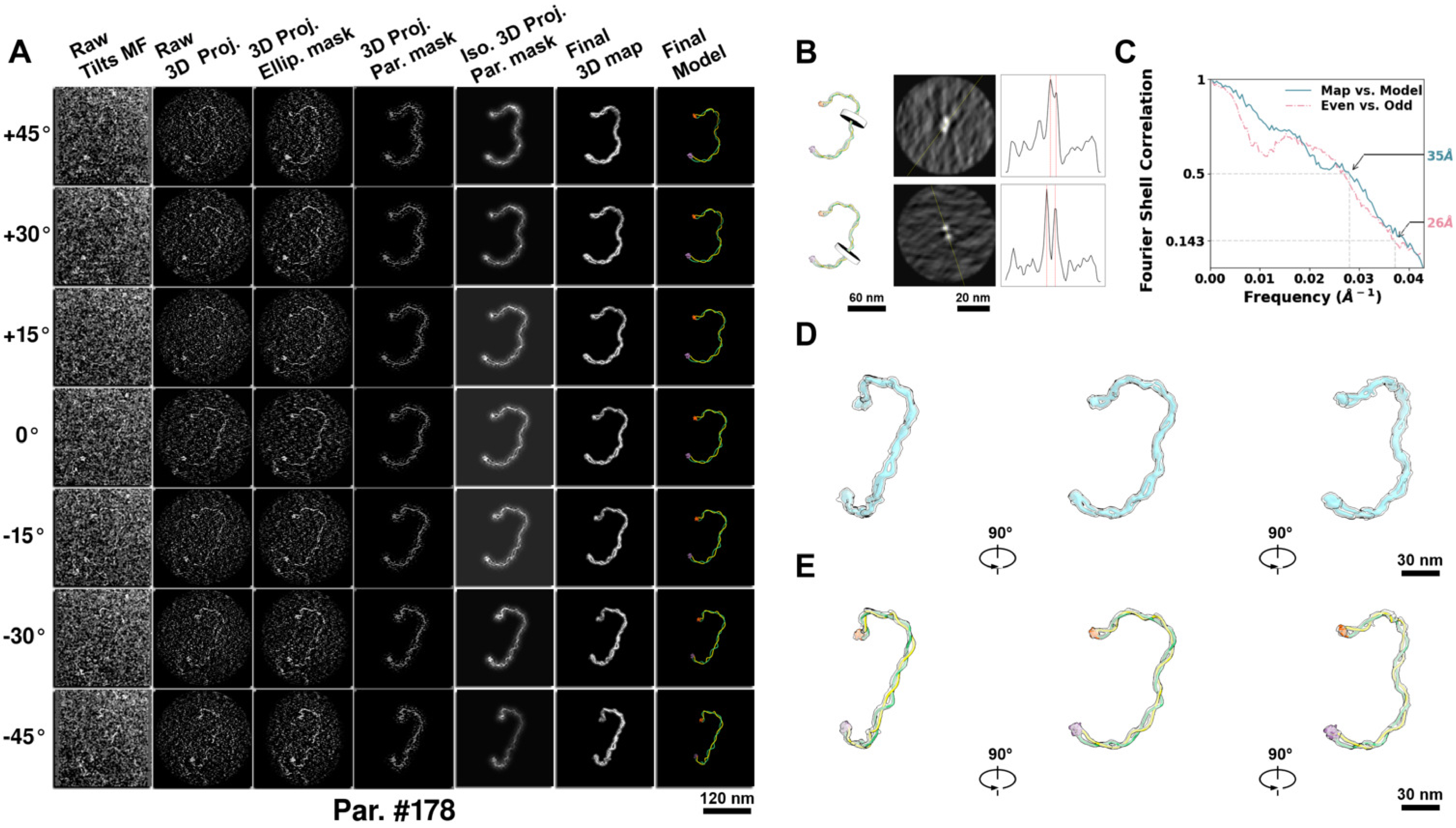

Supplementary Figure 178. Cryo-electron tomography (cryo-ET) 3D reconstruction of an individual plasmid particle in 40 mM K⁺, 5 mM Mg²⁺ in the presence of stalled RNAP and dCas9.
(A) 3D reconstruction of the plasmid particle (index no. 178). The first column shows seven representative tilt images from –45° to +45° in step of 15°. The second, third, and fourth columns show 3D projections of the particle with spherical, ellipsoidal (thinner along the z-dimension), and particle-shaped masks, respectively. The fifth column displays the 3D projections of the enhanced and IsoNet missing-wedge-corrected particle. The sixth and seventh columns present the final 3D map and the flexibly fitted model, respectively. (B) Two random cross-sectional views (12 nm thickness) of the plasmid density map along its plectoneme axis are shown in the left-middle panel. The intensity profile along the line crossing the two high-density DNA spots is displayed in the right panel. (C) Resolution assessment of the final 3D map using Fourier shell correlation (FSC). Two criteria are shown: FSC between two half-maps reconstructed from even and odd frames (evaluated at 0.143) and FSC between the final 3D map and the fitted model (evaluated at 0.5). (D) Zoomed-in views of the final 3D density map from panel A, displayed at two contour levels. (E) Superimposition of the high-contour level map from panel D onto its flexibly fitted model.

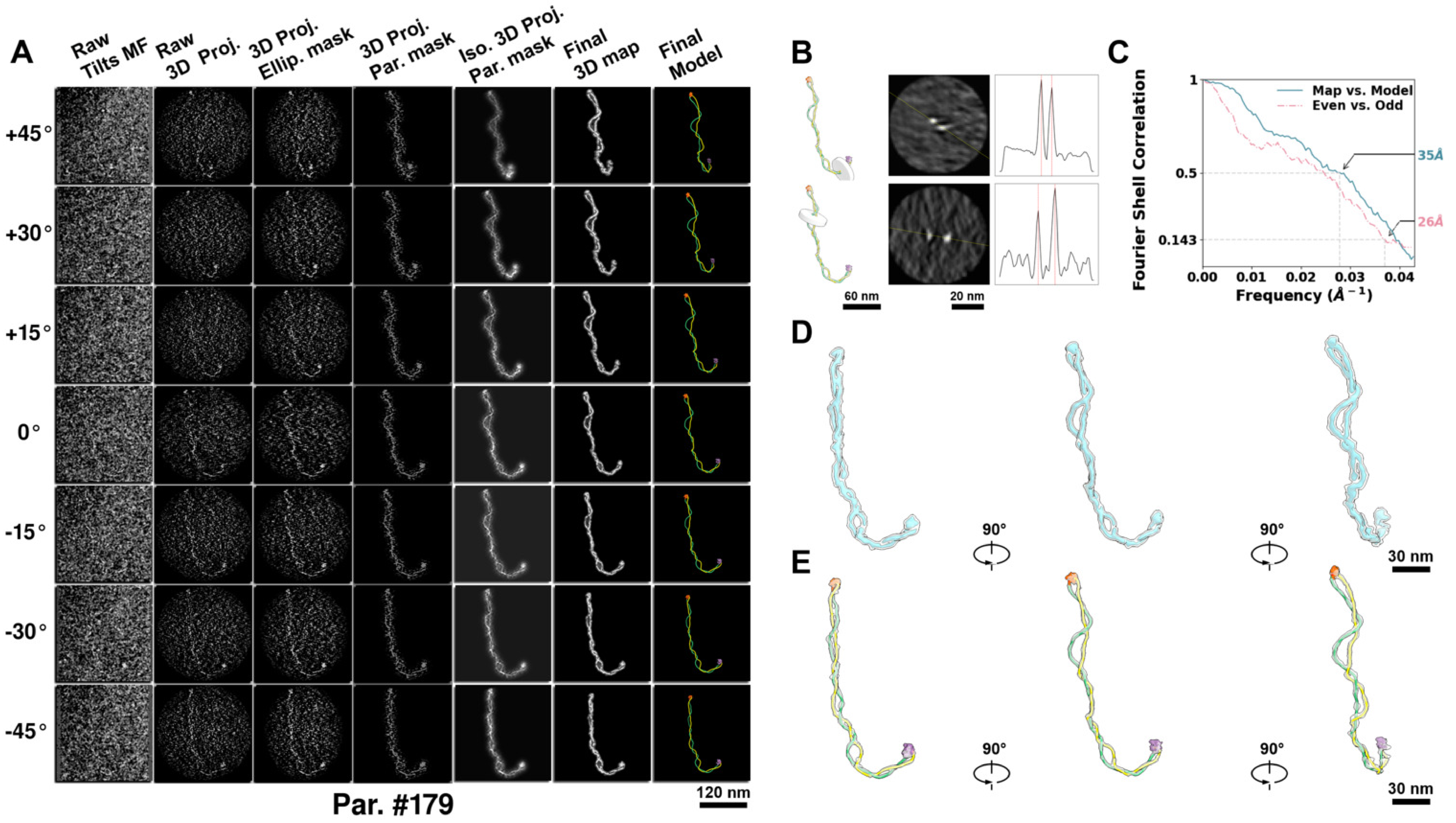

Supplementary Figure 179. Cryo-electron tomography (cryo-ET) 3D reconstruction of an individual plasmid particle in 40 mM K^{+}, 5 mM Mg^{2+} in the presence of stalled RNAP and dCas9.
(A) 3D reconstruction of the plasmid particle (index no. 179). The first column shows seven representative tilt images from –45° to +45° in step of 15°. The second, third, and fourth columns show 3D projections of the particle with spherical, ellipsoidal (thinner along the z-dimension), and particle-shaped masks, respectively. The fifth column displays the 3D projections of the enhanced and IsoNet missing-wedge-corrected particle. The sixth and seventh columns present the final 3D map and the flexibly fitted model, respectively. (B) Two random cross-sectional views (12 nm thickness) of the plasmid density map along its plectoneme axis are shown in the left-middle panel. The intensity profile along the line crossing the two high-density DNA spots is displayed in the right panel. (C) Resolution assessment of the final 3D map using Fourier shell correlation (FSC). Two criteria are shown: FSC between two half-maps reconstructed from even and odd frames (evaluated at 0.143) and FSC between the final 3D map and the fitted model (evaluated at 0.5). (D) Zoomed-in views of the final 3D density map from panel A, displayed at two contour levels. (E) Superimposition of the high-contour level map from panel D onto its flexibly fitted model.

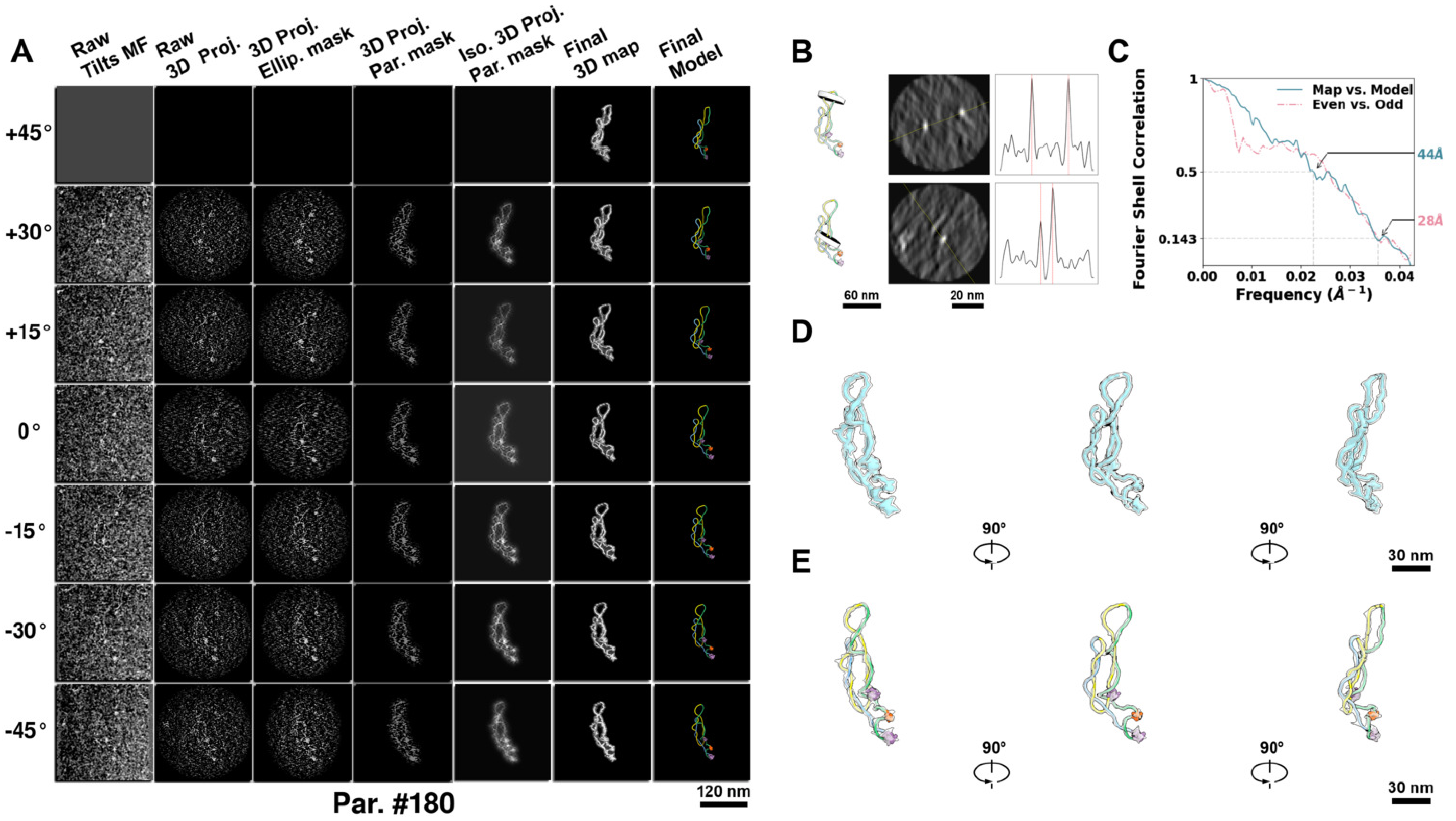

Supplementary Figure 180. Cryo-electron tomography (cryo-ET) 3D reconstruction of an individual plasmid particle in 40 mM K^{+}, 5 mM Mg^{2+} in the presence of stalled RNAP and dCas9.
(A) 3D reconstruction of the plasmid particle (index no. 180). The first column shows seven representative tilt images from –45° to +45° in step of 15°. The second, third, and fourth columns show 3D projections of the particle with spherical, ellipsoidal (thinner along the z-dimension), and particle-shaped masks, respectively. The fifth column displays the 3D projections of the enhanced and IsoNet missing-wedge-corrected particle. The sixth and seventh columns present the final 3D map and the flexibly fitted model, respectively. (B) Two random cross-sectional views (12 nm thickness) of the plasmid density map along its plectoneme axis are shown in the left-middle panel. The intensity profile along the line crossing the two high-density DNA spots is displayed in the right panel. (C) Resolution assessment of the final 3D map using Fourier shell correlation (FSC). Two criteria are shown: FSC between two half-maps reconstructed from even and odd frames (evaluated at 0.143) and FSC between the final 3D map and the fitted model (evaluated at 0.5). (D) Zoomed-in views of the final 3D density map from panel A, displayed at two contour levels. (E) Superimposition of the high-contour level map from panel D onto its flexibly fitted model.

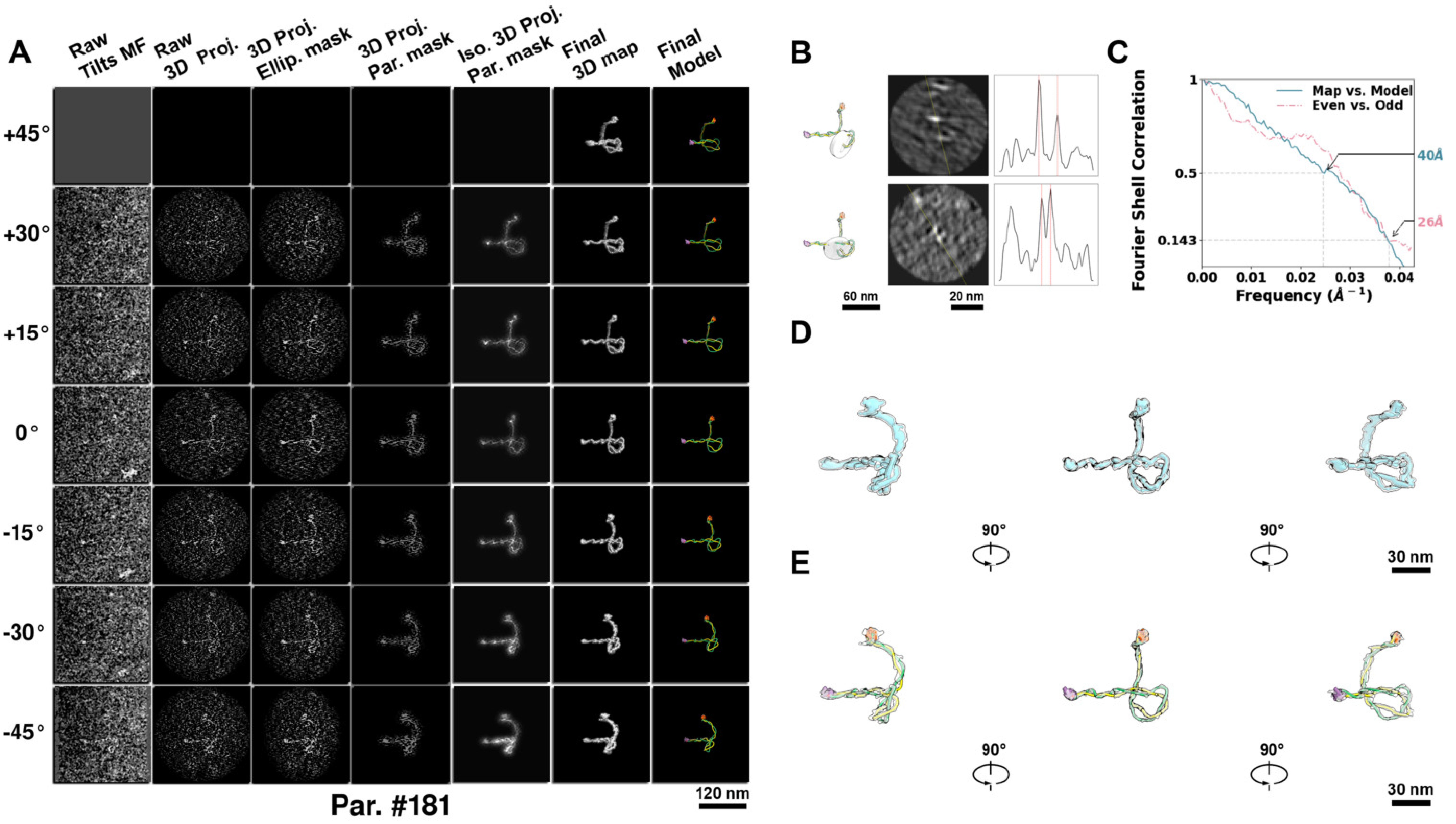

Supplementary Figure 181. Cryo-electron tomography (cryo-ET) 3D reconstruction of an individual plasmid particle in 40 mM K^+, 5 mM Mg^{2+} in the presence of stalled RNAP and dCas9.
(A) 3D reconstruction of the plasmid particle (index no. 181). The first column shows seven representative tilt images from –45° to +45° in step of 15°. The second, third, and fourth columns show 3D projections of the particle with spherical, ellipsoidal (thinner along the z-dimension), and particle-shaped masks, respectively. The fifth column displays the 3D projections of the enhanced and IsoNet missing-wedge-corrected particle. The sixth and seventh columns present the final 3D map and the flexibly fitted model, respectively. (B) Two random cross-sectional views (12 nm thickness) of the plasmid density map along its plectoneme axis are shown in the left-middle panel. The intensity profile along the line crossing the two high-density DNA spots is displayed in the right panel. (C) Resolution assessment of the final 3D map using Fourier shell correlation (FSC). Two criteria are shown: FSC between two half-maps reconstructed from even and odd frames (evaluated at 0.143) and FSC between the final 3D map and the fitted model (evaluated at 0.5). (D) Zoomed-in views of the final 3D density map from panel A, displayed at two contour levels. (E) Superimposition of the high-contour level map from panel D onto its flexibly fitted model.

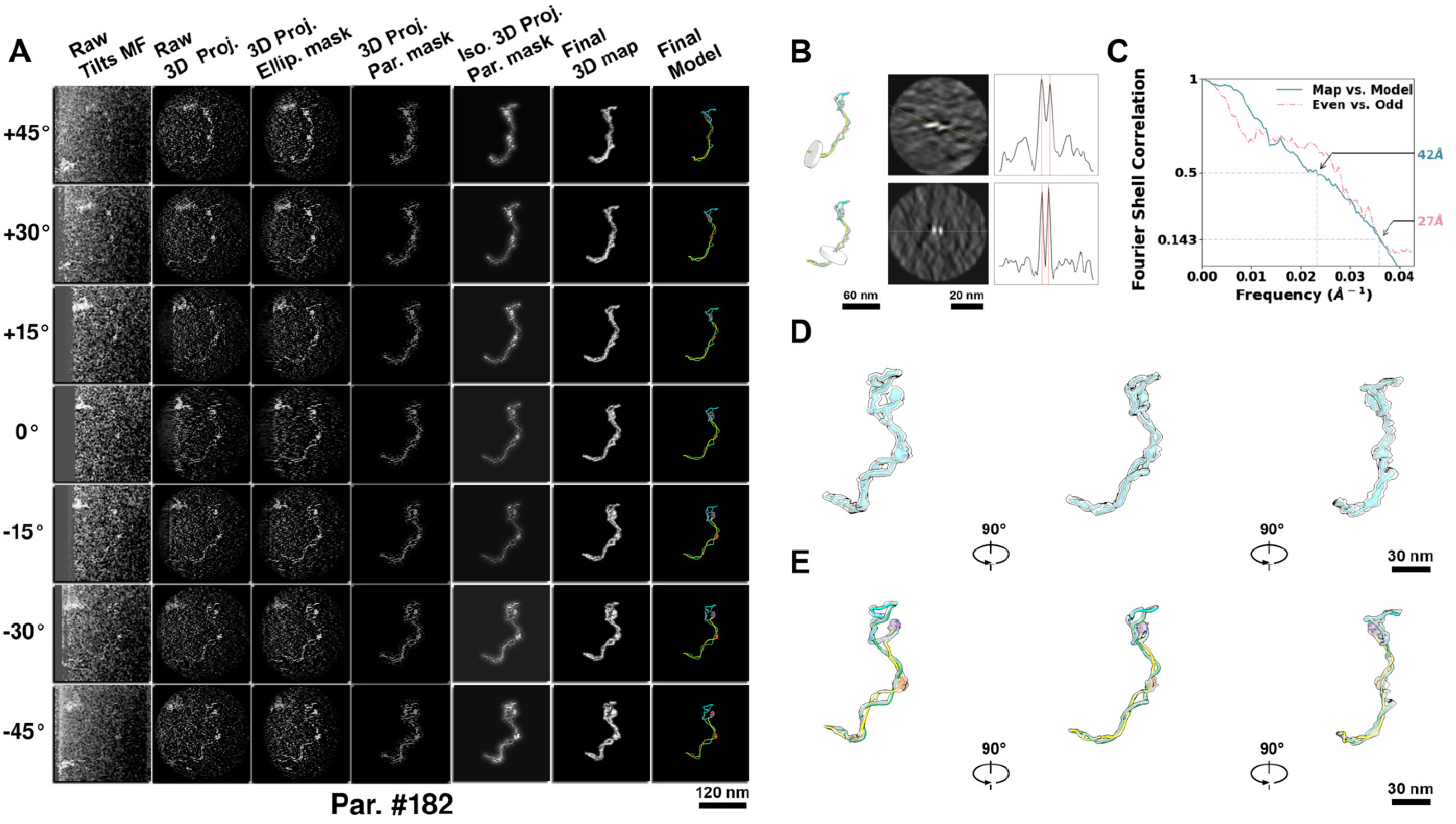

Supplementary Figure 182. Cryo-electron tomography (cryo-ET) 3D reconstruction of an individual plasmid particle in 40 mM K^+, 5 mM Mg^{2+} in the presence of stalled RNAP and dCas9.
(A) 3D reconstruction of the plasmid particle (index no. 182). The first column shows seven representative tilt images from –45° to +45° in step of 15°. The second, third, and fourth columns show 3D projections of the particle with spherical, ellipsoidal (thinner along the z-dimension), and particle-shaped masks, respectively. The fifth column displays the 3D projections of the enhanced and IsoNet missing-wedge-corrected particle. The sixth and seventh columns present the final 3D map and the flexibly fitted model, respectively. (B) Two random cross-sectional views (12 nm thickness) of the plasmid density map along its plectoneme axis are shown in the left-middle panel. The intensity profile along the line crossing the two high-density DNA spots is displayed in the right panel. (C) Resolution assessment of the final 3D map using Fourier shell correlation (FSC). Two criteria are shown: FSC between two half-maps reconstructed from even and odd frames (evaluated at 0.143) and FSC between the final 3D map and the fitted model (evaluated at 0.5). (D) Zoomed-in views of the final 3D density map from panel A, displayed at two contour levels. (E) Superimposition of the high-contour level map from panel D onto its flexibly fitted model.

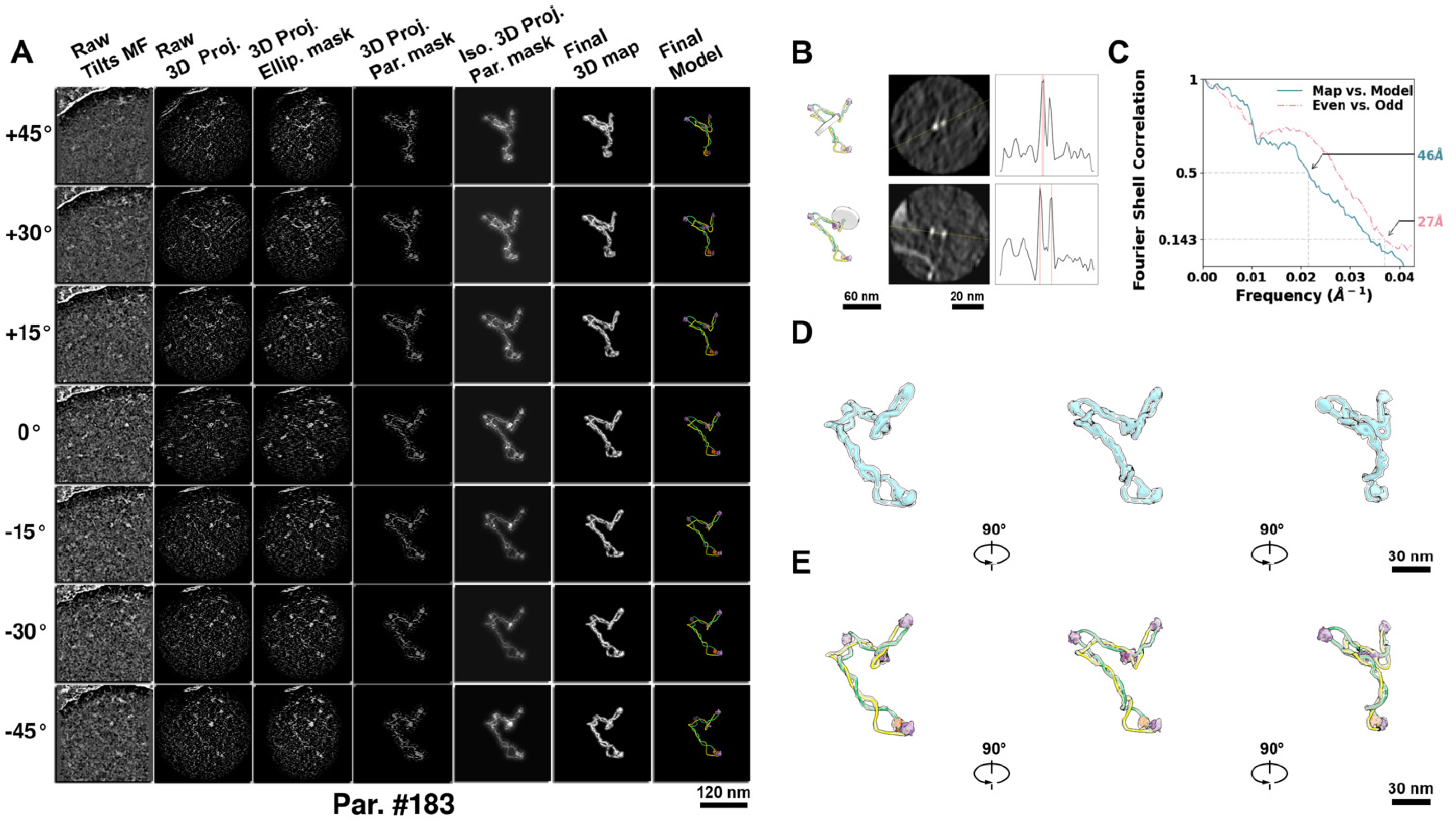

Supplementary Figure 183. Cryo-electron tomography (cryo-ET) 3D reconstruction of an individual plasmid particle in 40 mM K^+, 5 mM Mg^{2+} in the presence of transcribed RNAP and dCas9.
(A) 3D reconstruction of the plasmid particle (index no. 183). The first column shows seven representative tilt images from –45° to +45° in step of 15°. The second, third, and fourth columns show 3D projections of the particle with spherical, ellipsoidal (thinner along the z-dimension), and particle-shaped masks, respectively. The fifth column displays the 3D projections of the enhanced and IsoNet missing-wedge-corrected particle. The sixth and seventh columns present the final 3D map and the flexibly fitted model, respectively. (B) Two random cross-sectional views (12 nm thickness) of the plasmid density map along its plectoneme axis are shown in the left-middle panel. The intensity profile along the line crossing the two high-density DNA spots is displayed in the right panel. (C) Resolution assessment of the final 3D map using Fourier shell correlation (FSC). Two criteria are shown: FSC between two half-maps reconstructed from even and odd frames (evaluated at 0.143) and FSC between the final 3D map and the fitted model (evaluated at 0.5). (D) Zoomed-in views of the final 3D density map from panel A, displayed at two contour levels. (E) Superimposition of the high-contour level map from panel D onto its flexibly fitted model.

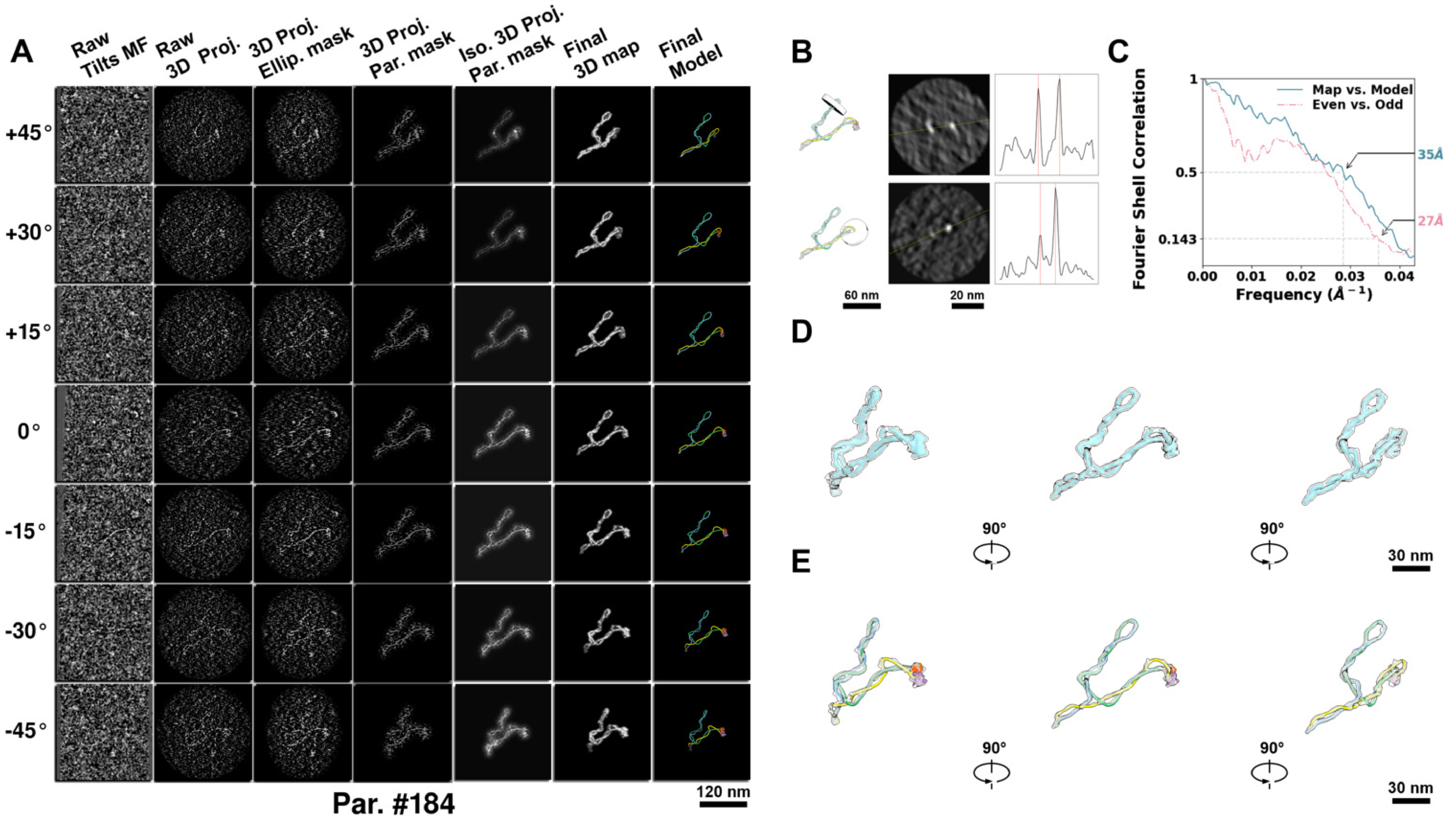

Supplementary Figure 184. Cryo-electron tomography (cryo-ET) 3D reconstruction of an individual plasmid particle in 40 mM K^+, 5 mM Mg^{2+} in the presence of transcribed RNAP and dCas9.
(A) 3D reconstruction of the plasmid particle (index no. 184). The first column shows seven representative tilt images from –45° to +45° in step of 15°. The second, third, and fourth columns show 3D projections of the particle with spherical, ellipsoidal (thinner along the z-dimension), and particle-shaped masks, respectively. The fifth column displays the 3D projections of the enhanced and IsoNet missing-wedge-corrected particle. The sixth and seventh columns present the final 3D map and the flexibly fitted model, respectively. (B) Two random cross-sectional views (12 nm thickness) of the plasmid density map along its plectoneme axis are shown in the left-middle panel. The intensity profile along the line crossing the two high-density DNA spots is displayed in the right panel. (C) Resolution assessment of the final 3D map using Fourier shell correlation (FSC). Two criteria are shown: FSC between two half-maps reconstructed from even and odd frames (evaluated at 0.143) and FSC between the final 3D map and the fitted model (evaluated at 0.5). (D) Zoomed-in views of the final 3D density map from panel A, displayed at two contour levels. (E) Superimposition of the high-contour level map from panel D onto its flexibly fitted model.

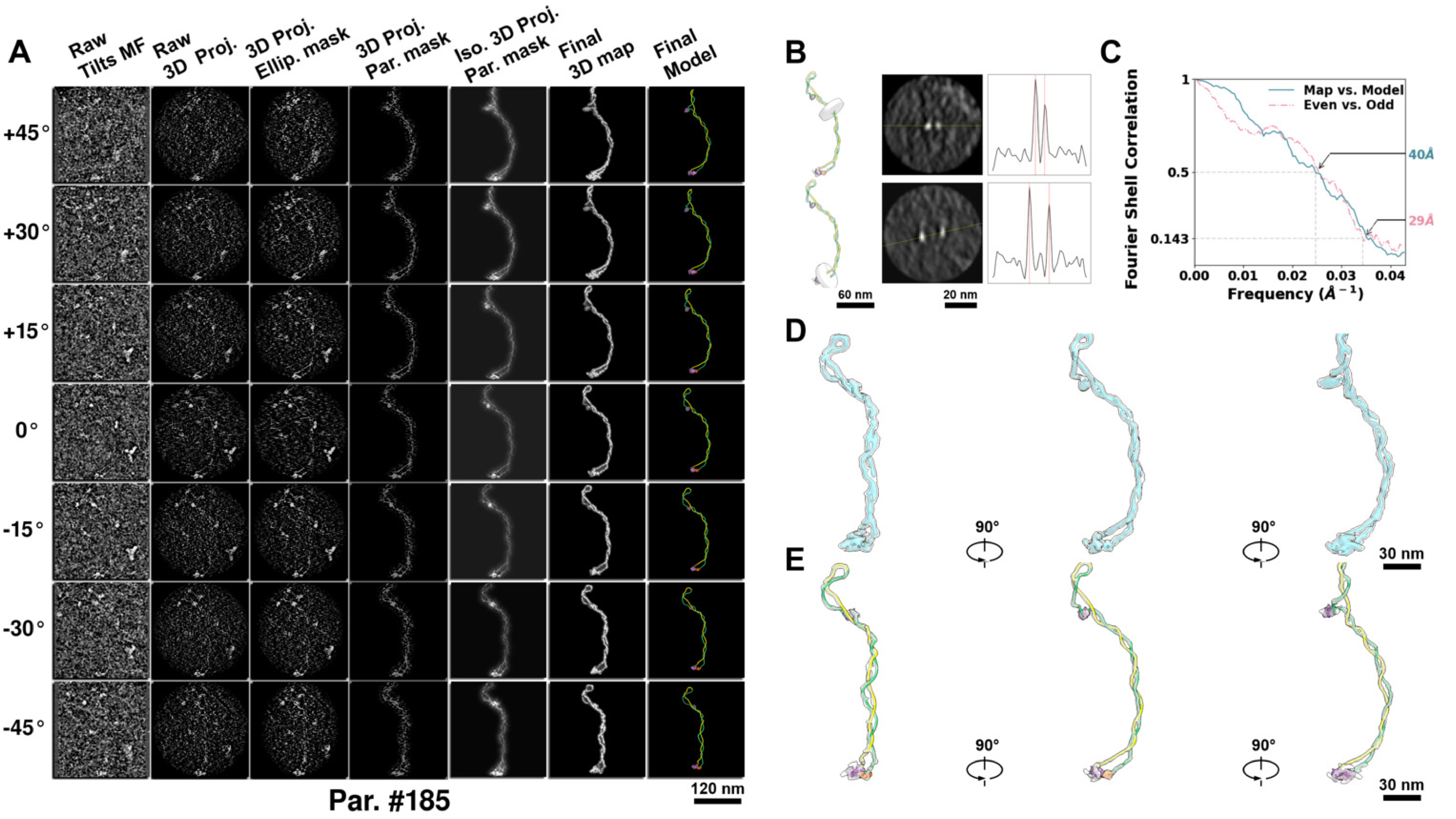

Supplementary Figure 185. Cryo-electron tomography (cryo-ET) 3D reconstruction of an individual plasmid particle in 40 mM K⁺, 5 mM Mg²⁺ in the presence of transcribed RNAP and dCas9.
(A) 3D reconstruction of the plasmid particle (index no. 185). The first column shows seven representative tilt images from –45° to +45° in step of 15°. The second, third, and fourth columns show 3D projections of the particle with spherical, ellipsoidal (thinner along the z-dimension), and particle-shaped masks, respectively. The fifth column displays the 3D projections of the enhanced and IsoNet missing-wedge-corrected particle. The sixth and seventh columns present the final 3D map and the flexibly fitted model, respectively. (B) Two random cross-sectional views (12 nm thickness) of the plasmid density map along its plectoneme axis are shown in the left-middle panel. The intensity profile along the line crossing the two high-density DNA spots is displayed in the right panel. (C) Resolution assessment of the final 3D map using Fourier shell correlation (FSC). Two criteria are shown: FSC between two half-maps reconstructed from even and odd frames (evaluated at 0.143) and FSC between the final 3D map and the fitted model (evaluated at 0.5). (D) Zoomed-in views of the final 3D density map from panel A, displayed at two contour levels. (E) Superimposition of the high-contour level map from panel D onto its flexibly fitted model.

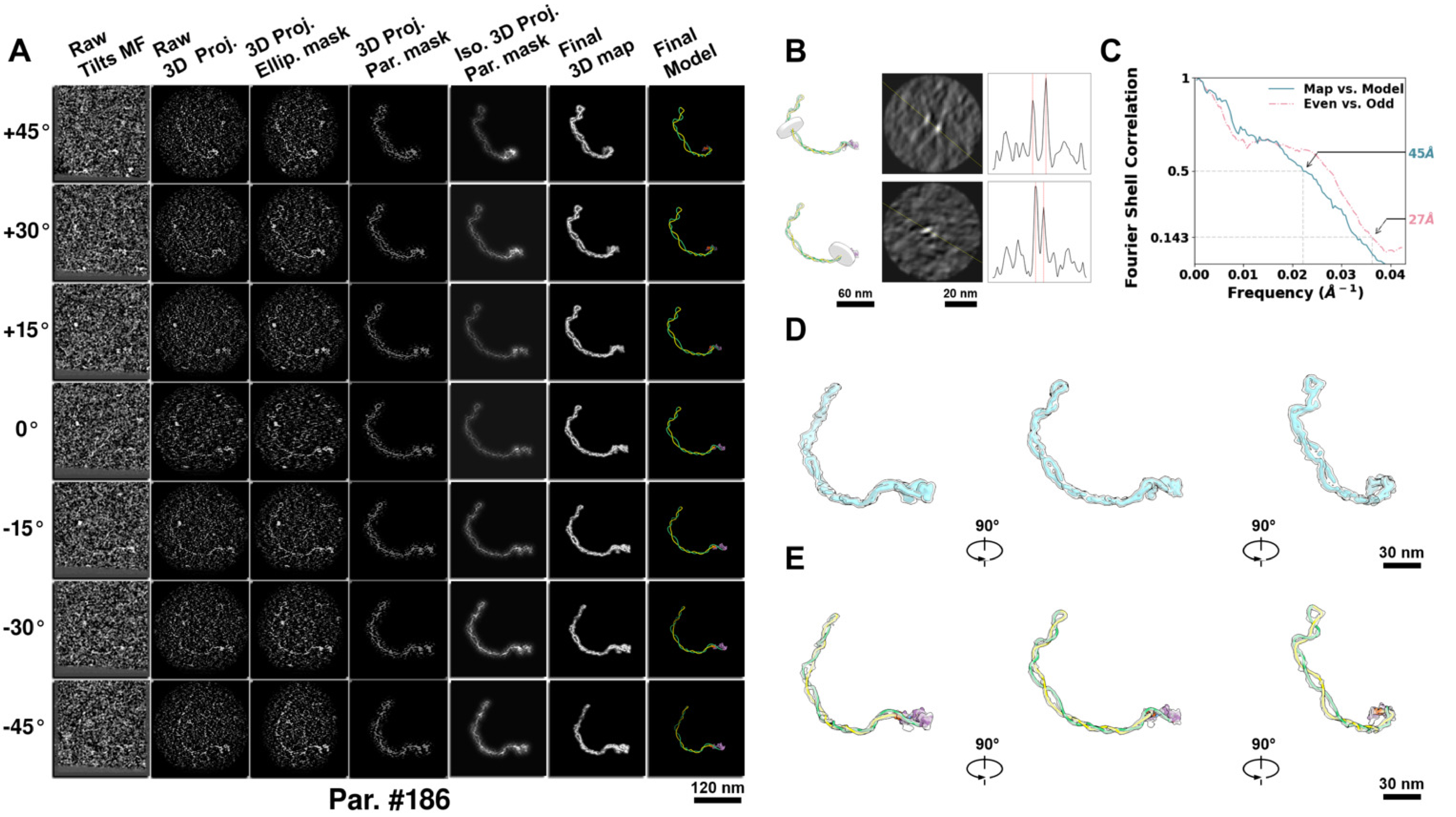

Supplementary Figure 186. Cryo-electron tomography (cryo-ET) 3D reconstruction of an individual plasmid particle in 40 mM K⁺, 5 mM Mg²⁺ in the presence of transcribed RNAP and dCas9.
(A) 3D reconstruction of the plasmid particle (index no. 186). The first column shows seven representative tilt images from –45° to +45° in step of 15°. The second, third, and fourth columns show 3D projections of the particle with spherical, ellipsoidal (thinner along the z-dimension), and particle-shaped masks, respectively. The fifth column displays the 3D projections of the enhanced and IsoNet missing-wedge-corrected particle. The sixth and seventh columns present the final 3D map and the flexibly fitted model, respectively. (B) Two random cross-sectional views (12 nm thickness) of the plasmid density map along its plectoneme axis are shown in the left-middle panel. The intensity profile along the line crossing the two high-density DNA spots is displayed in the right panel. (C) Resolution assessment of the final 3D map using Fourier shell correlation (FSC). Two criteria are shown: FSC between two half-maps reconstructed from even and odd frames (evaluated at 0.143) and FSC between the final 3D map and the fitted model (evaluated at 0.5). (D) Zoomed-in views of the final 3D density map from panel A, displayed at two contour levels. (E) Superimposition of the high-contour level map from panel D onto its flexibly fitted model.

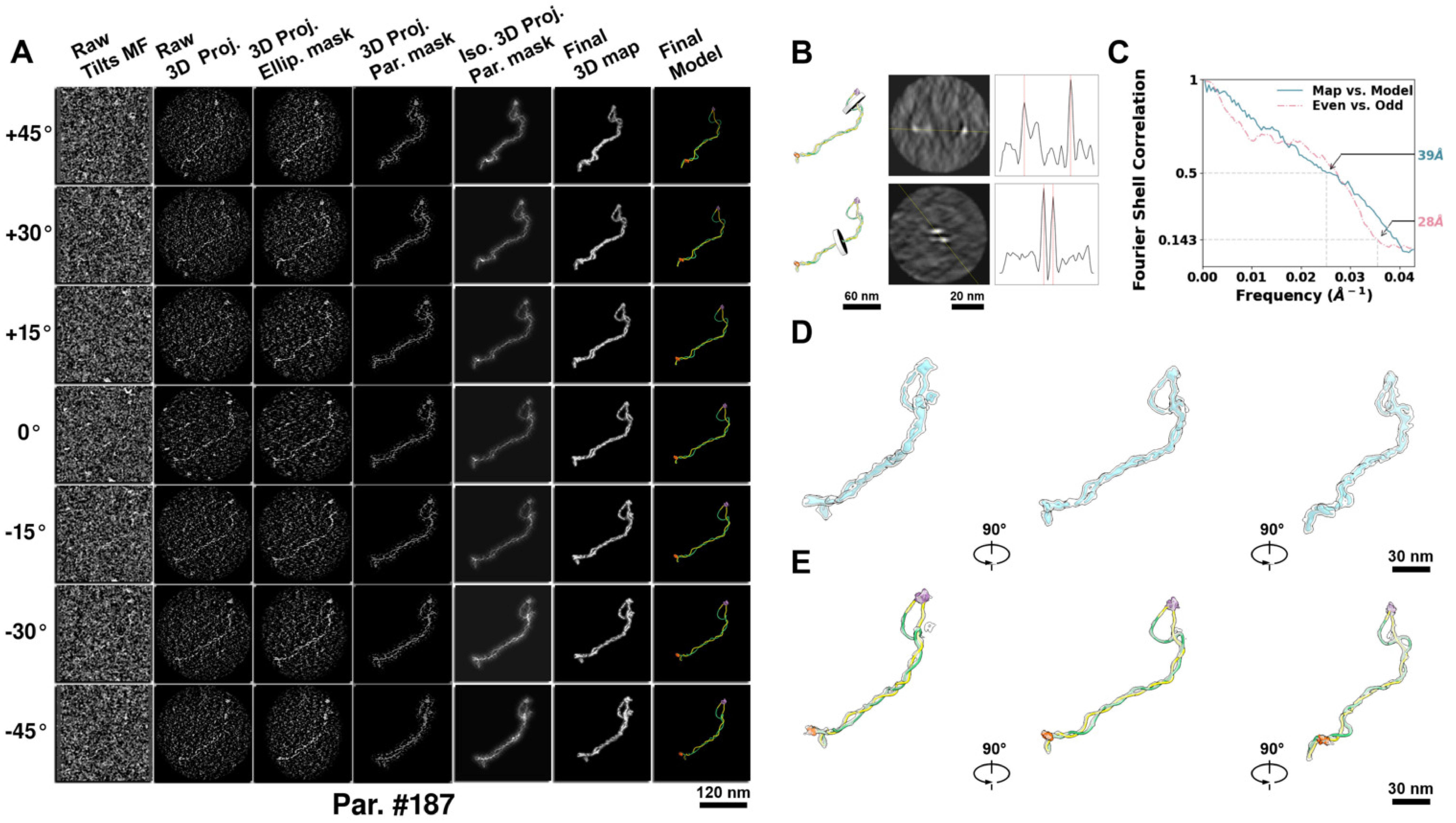

Supplementary Figure 187. Cryo-electron tomography (cryo-ET) 3D reconstruction of an individual plasmid particle in 40 mM K^+, 5 mM Mg^{2+} in the presence of transcribed RNAP and dCas9.
(A) 3D reconstruction of the plasmid particle (index no. 187). The first column shows seven representative tilt images from –45° to +45° in step of 15°. The second, third, and fourth columns show 3D projections of the particle with spherical, ellipsoidal (thinner along the z-dimension), and particle-shaped masks, respectively. The fifth column displays the 3D projections of the enhanced and IsoNet missing-wedge-corrected particle. The sixth and seventh columns present the final 3D map and the flexibly fitted model, respectively. (B) Two random cross-sectional views (12 nm thickness) of the plasmid density map along its plectoneme axis are shown in the left-middle panel. The intensity profile along the line crossing the two high-density DNA spots is displayed in the right panel. (C) Resolution assessment of the final 3D map using Fourier shell correlation (FSC). Two criteria are shown: FSC between two half-maps reconstructed from even and odd frames (evaluated at 0.143) and FSC between the final 3D map and the fitted model (evaluated at 0.5). (D) Zoomed-in views of the final 3D density map from panel A, displayed at two contour levels. (E) Superimposition of the high-contour level map from panel D onto its flexibly fitted model.

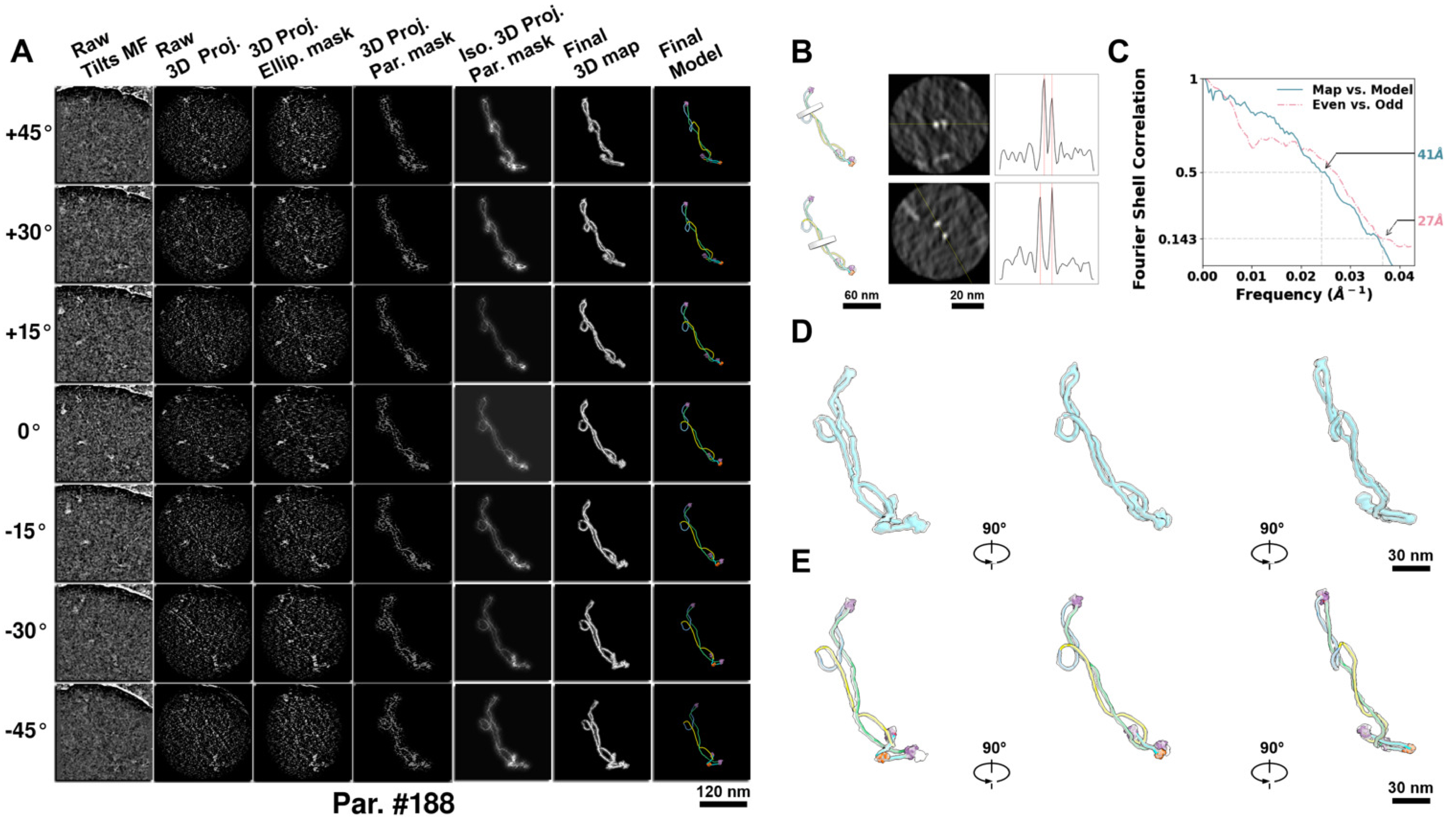

Supplementary Figure 188. Cryo-electron tomography (cryo-ET) 3D reconstruction of an individual plasmid particle in 40 mM K^+, 5 mM Mg^{2+} in the presence of transcribed RNAP and dCas9.
(A) 3D reconstruction of the plasmid particle (index no. 188). The first column shows seven representative tilt images from –45° to +45° in step of 15°. The second, third, and fourth columns show 3D projections of the particle with spherical, ellipsoidal (thinner along the z-dimension), and particle-shaped masks, respectively. The fifth column displays the 3D projections of the enhanced and IsoNet missing-wedge-corrected particle. The sixth and seventh columns present the final 3D map and the flexibly fitted model, respectively. (B) Two random cross-sectional views (12 nm thickness) of the plasmid density map along its plectoneme axis are shown in the left-middle panel. The intensity profile along the line crossing the two high-density DNA spots is displayed in the right panel. (C) Resolution assessment of the final 3D map using Fourier shell correlation (FSC). Two criteria are shown: FSC between two half-maps reconstructed from even and odd frames (evaluated at 0.143) and FSC between the final 3D map and the fitted model (evaluated at 0.5). (D) Zoomed-in views of the final 3D density map from panel A, displayed at two contour levels. (E) Superimposition of the high-contour level map from panel D onto its flexibly fitted model.

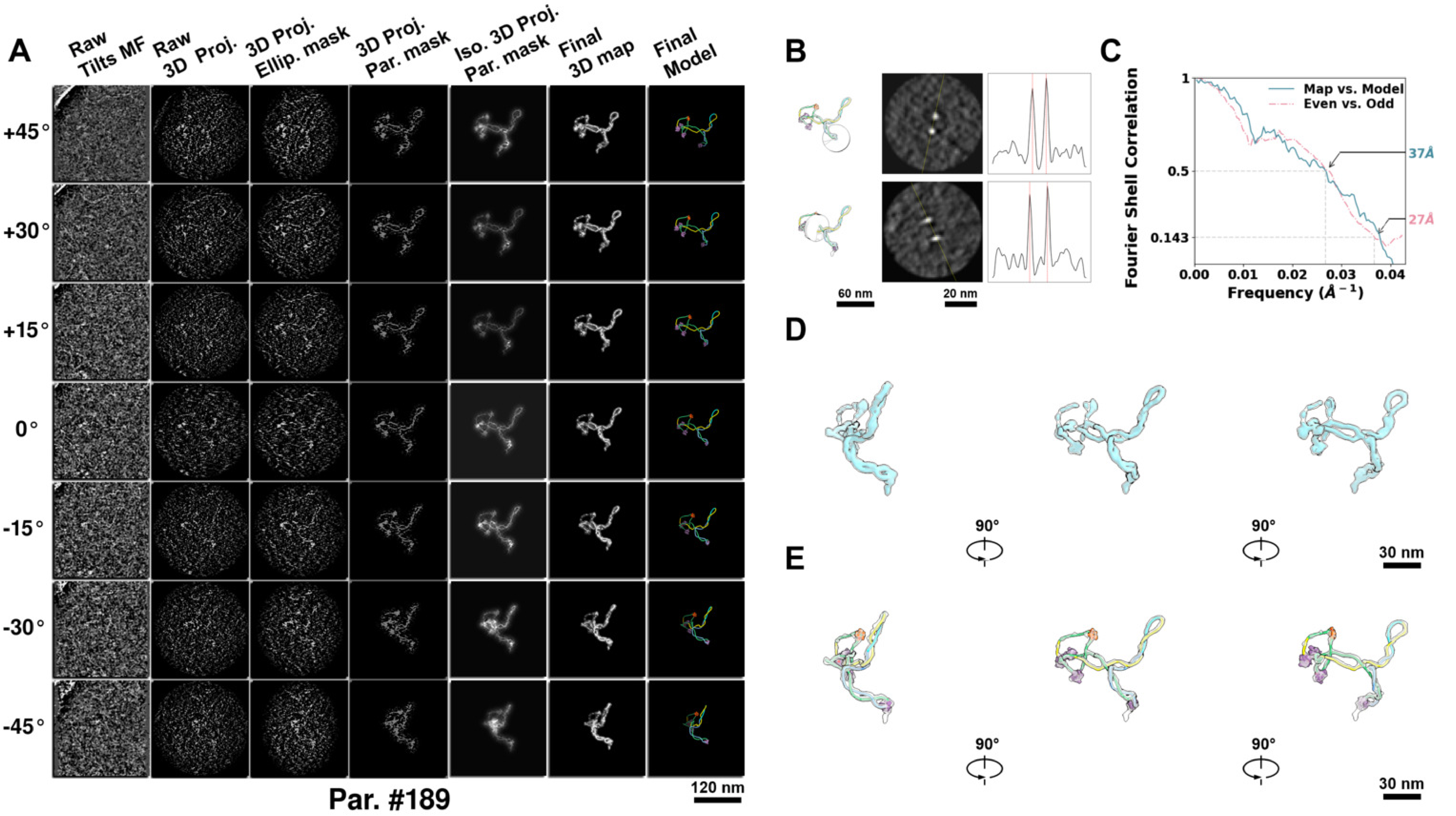

Supplementary Figure 189. Cryo-electron tomography (cryo-ET) 3D reconstruction of an individual plasmid particle in 40 mM K^+, 5 mM Mg^{2+} in the presence of transcribed RNAP and dCas9.
(A) 3D reconstruction of the plasmid particle (index no. 189). The first column shows seven representative tilt images from –45° to +45° in step of 15°. The second, third, and fourth columns show 3D projections of the particle with spherical, ellipsoidal (thinner along the z-dimension), and particle-shaped masks, respectively. The fifth column displays the 3D projections of the enhanced and IsoNet missing-wedge-corrected particle. The sixth and seventh columns present the final 3D map and the flexibly fitted model, respectively. (B) Two random cross-sectional views (12 nm thickness) of the plasmid density map along its plectoneme axis are shown in the left-middle panel. The intensity profile along the line crossing the two high-density DNA spots is displayed in the right panel. (C) Resolution assessment of the final 3D map using Fourier shell correlation (FSC). Two criteria are shown: FSC between two half-maps reconstructed from even and odd frames (evaluated at 0.143) and FSC between the final 3D map and the fitted model (evaluated at 0.5). (D) Zoomed-in views of the final 3D density map from panel A, displayed at two contour levels. (E) Superimposition of the high-contour level map from panel D onto its flexibly fitted model.

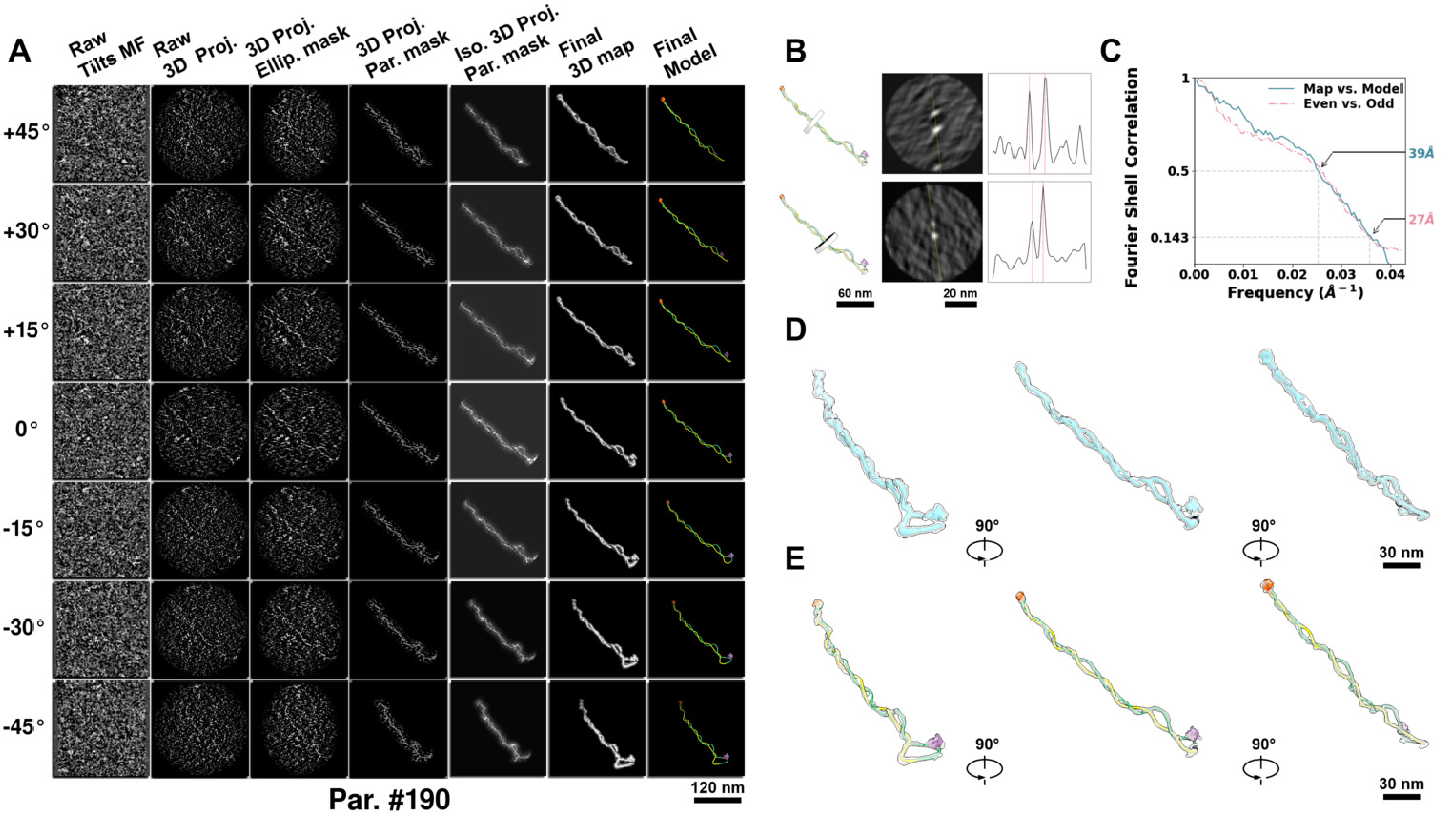

Supplementary Figure 190. Cryo-electron tomography (cryo-ET) 3D reconstruction of an individual plasmid particle in 40 mM K^+, 5 mM Mg^{2+} in the presence of transcribed RNAP and dCas9.
(A) 3D reconstruction of the plasmid particle (index no. 190). The first column shows seven representative tilt images from –45° to +45° in step of 15°. The second, third, and fourth columns show 3D projections of the particle with spherical, ellipsoidal (thinner along the z-dimension), and particle-shaped masks, respectively. The fifth column displays the 3D projections of the enhanced and IsoNet missing-wedge-corrected particle. The sixth and seventh columns present the final 3D map and the flexibly fitted model, respectively. (B) Two random cross-sectional views (12 nm thickness) of the plasmid density map along its plectoneme axis are shown in the left-middle panel. The intensity profile along the line crossing the two high-density DNA spots is displayed in the right panel. (C) Resolution assessment of the final 3D map using Fourier shell correlation (FSC). Two criteria are shown: FSC between two half-maps reconstructed from even and odd frames (evaluated at 0.143) and FSC between the final 3D map and the fitted model (evaluated at 0.5). (D) Zoomed-in views of the final 3D density map from panel A, displayed at two contour levels. (E) Superimposition of the high-contour level map from panel D onto its flexibly fitted model.

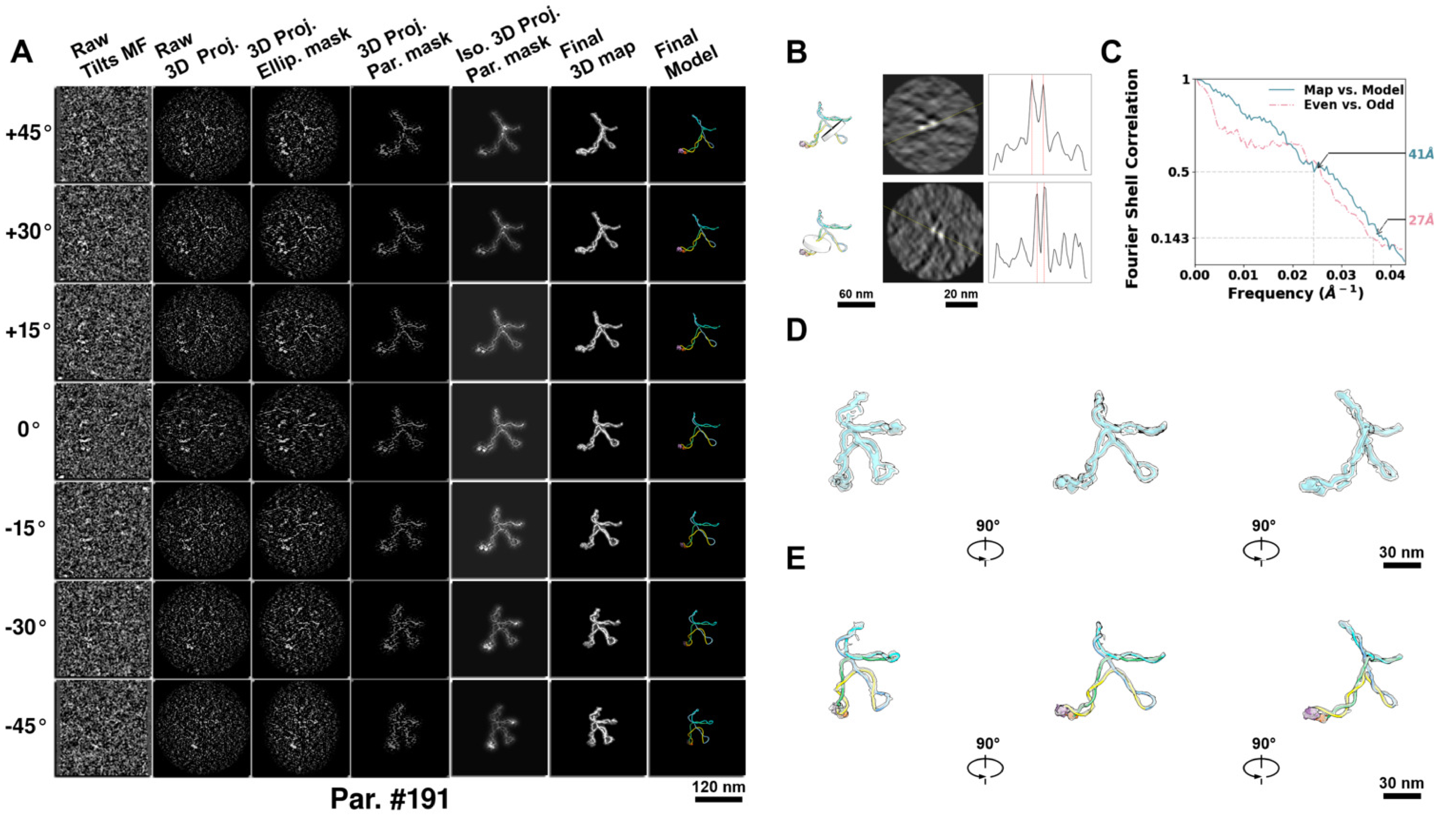

Supplementary Figure 191. Cryo-electron tomography (cryo-ET) 3D reconstruction of an individual plasmid particle in 40 mM K⁺, 5 mM Mg²⁺ in the presence of transcribed RNAP and dCas9.
(A) 3D reconstruction of the plasmid particle (index no. 191). The first column shows seven representative tilt images from –45° to +45° in step of 15°. The second, third, and fourth columns show 3D projections of the particle with spherical, ellipsoidal (thinner along the z-dimension), and particle-shaped masks, respectively. The fifth column displays the 3D projections of the enhanced and IsoNet missing-wedge-corrected particle. The sixth and seventh columns present the final 3D map and the flexibly fitted model, respectively. (B) Two random cross-sectional views (12 nm thickness) of the plasmid density map along its plectoneme axis are shown in the left-middle panel. The intensity profile along the line crossing the two high-density DNA spots is displayed in the right panel. (C) Resolution assessment of the final 3D map using Fourier shell correlation (FSC). Two criteria are shown: FSC between two half-maps reconstructed from even and odd frames (evaluated at 0.143) and FSC between the final 3D map and the fitted model (evaluated at 0.5). (D) Zoomed-in views of the final 3D density map from panel A, displayed at two contour levels. (E) Superimposition of the high-contour level map from panel D onto its flexibly fitted model.

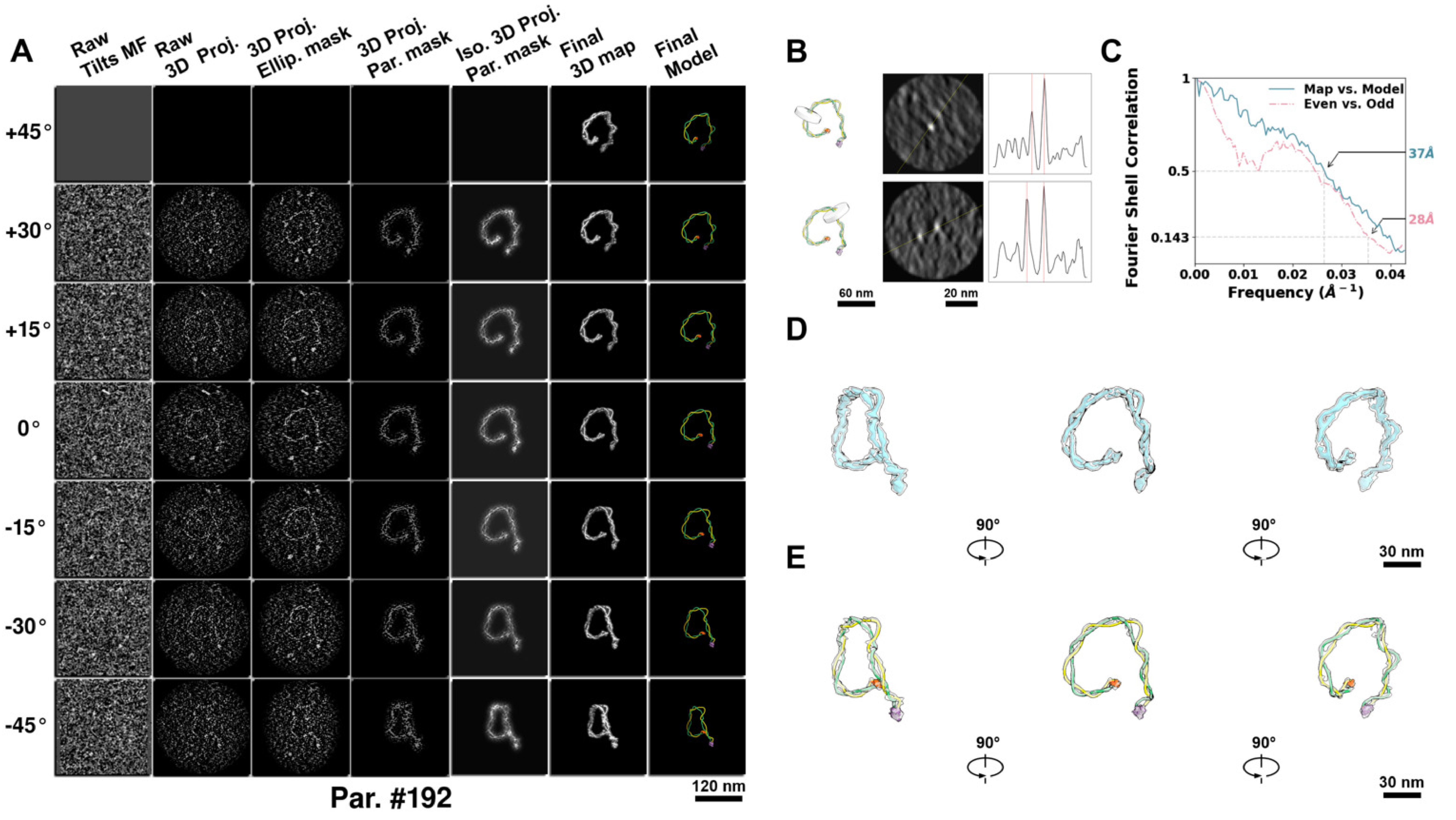

Supplementary Figure 192. Cryo-electron tomography (cryo-ET) 3D reconstruction of an individual plasmid particle in 40 mM K⁺, 5 mM Mg²⁺ in the presence of transcribed RNAP and dCas9.
(A) 3D reconstruction of the plasmid particle (index no. 192). The first column shows seven representative tilt images from –45° to +45° in step of 15°. The second, third, and fourth columns show 3D projections of the particle with spherical, ellipsoidal (thinner along the z-dimension), and particle-shaped masks, respectively. The fifth column displays the 3D projections of the enhanced and IsoNet missing-wedge-corrected particle. The sixth and seventh columns present the final 3D map and the flexibly fitted model, respectively. (B) Two random cross-sectional views (12 nm thickness) of the plasmid density map along its plectoneme axis are shown in the left-middle panel. The intensity profile along the line crossing the two high-density DNA spots is displayed in the right panel. (C) Resolution assessment of the final 3D map using Fourier shell correlation (FSC). Two criteria are shown: FSC between two half-maps reconstructed from even and odd frames (evaluated at 0.143) and FSC between the final 3D map and the fitted model (evaluated at 0.5). (D) Zoomed-in views of the final 3D density map from panel A, displayed at two contour levels. (E) Superimposition of the high-contour level map from panel D onto its flexibly fitted model.

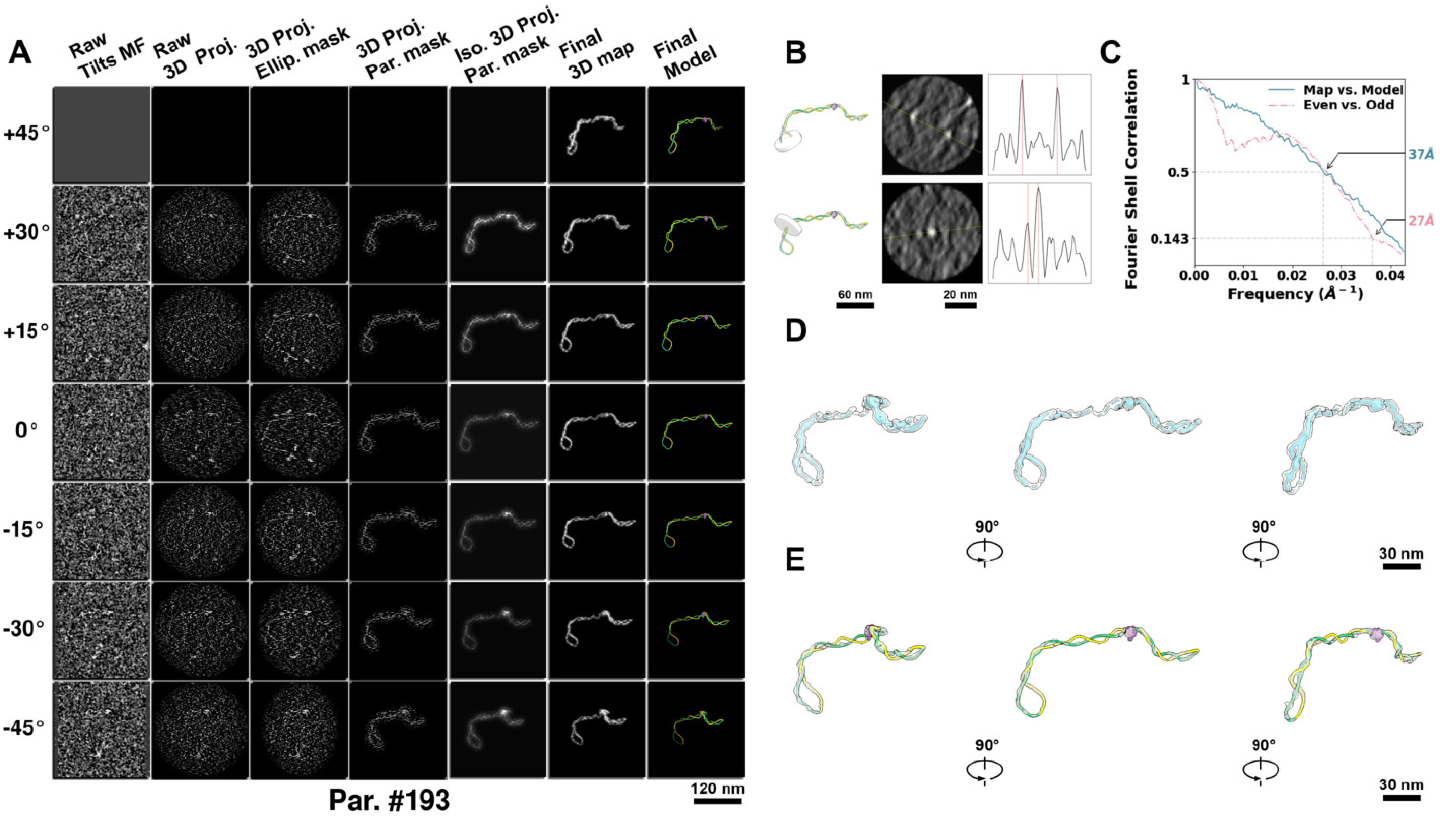

Supplementary Figure 193. Cryo-electron tomography (cryo-ET) 3D reconstruction of an individual plasmid particle in 40 mM K⁺, 5 mM Mg²⁺ in the presence of transcribed RNAP and dCas9.
(A) 3D reconstruction of the plasmid particle (index no. 193). The first column shows seven representative tilt images from –45° to +45° in step of 15°. The second, third, and fourth columns show 3D projections of the particle with spherical, ellipsoidal (thinner along the z-dimension), and particle-shaped masks, respectively. The fifth column displays the 3D projections of the enhanced and IsoNet missing-wedge-corrected particle. The sixth and seventh columns present the final 3D map and the flexibly fitted model, respectively. (B) Two random cross-sectional views (12 nm thickness) of the plasmid density map along its plectoneme axis are shown in the left-middle panel. The intensity profile along the line crossing the two high-density DNA spots is displayed in the right panel. (C) Resolution assessment of the final 3D map using Fourier shell correlation (FSC). Two criteria are shown: FSC between two half-maps reconstructed from even and odd frames (evaluated at 0.143) and FSC between the final 3D map and the fitted model (evaluated at 0.5). (D) Zoomed-in views of the final 3D density map from panel A, displayed at two contour levels. (E) Superimposition of the high-contour level map from panel D onto its flexibly fitted model.

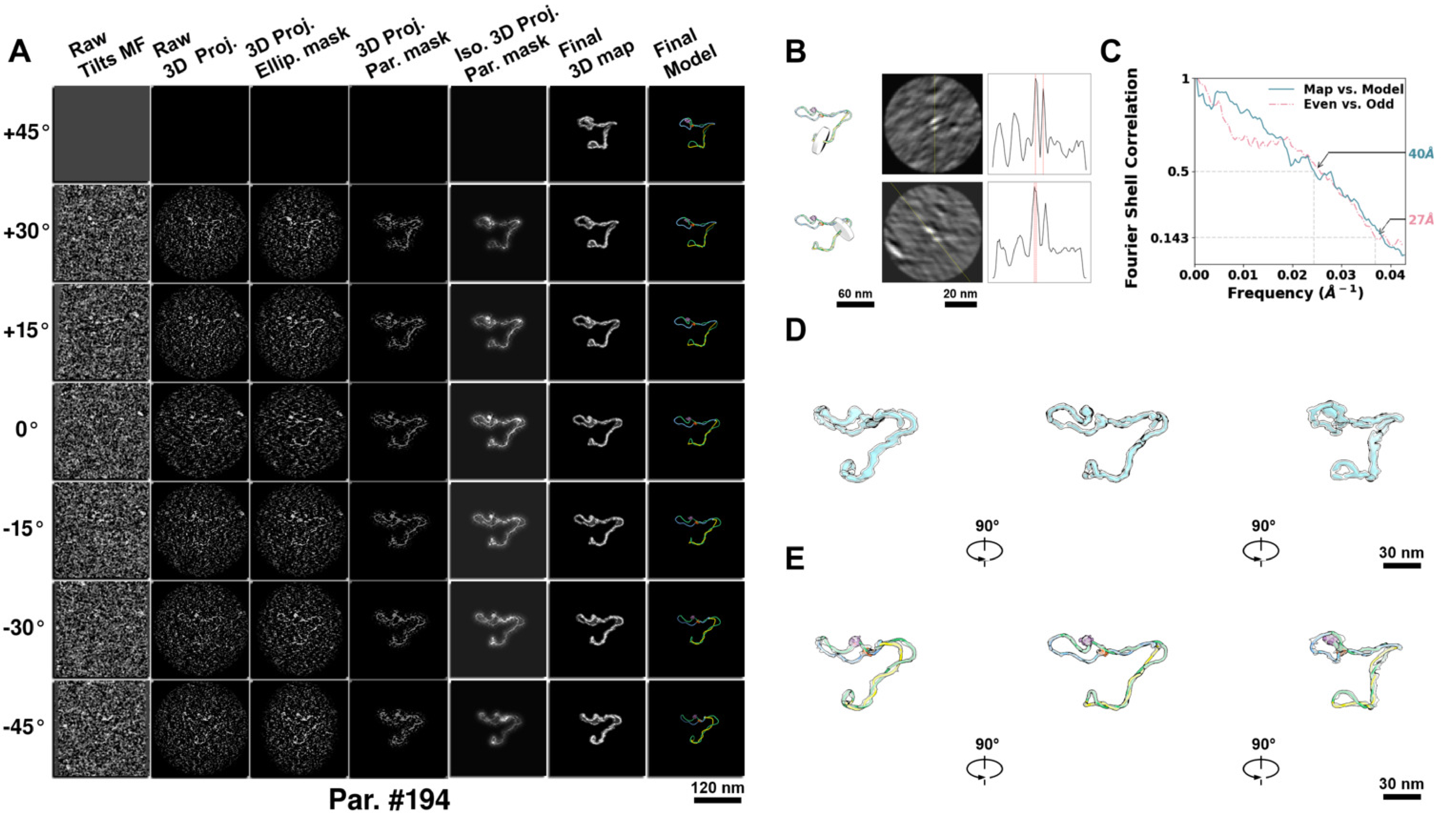

Supplementary Figure 194. Cryo-electron tomography (cryo-ET) 3D reconstruction of an individual plasmid particle in 40 mM K⁺, 5 mM Mg²⁺ in the presence of transcribed RNAP and dCas9.
(A) 3D reconstruction of the plasmid particle (index no. 194). The first column shows seven representative tilt images from –45° to +45° in step of 15°. The second, third, and fourth columns show 3D projections of the particle with spherical, ellipsoidal (thinner along the z-dimension), and particle-shaped masks, respectively. The fifth column displays the 3D projections of the enhanced and IsoNet missing-wedge-corrected particle. The sixth and seventh columns present the final 3D map and the flexibly fitted model, respectively. (B) Two random cross-sectional views (12 nm thickness) of the plasmid density map along its plectoneme axis are shown in the left-middle panel. The intensity profile along the line crossing the two high-density DNA spots is displayed in the right panel. (C) Resolution assessment of the final 3D map using Fourier shell correlation (FSC). Two criteria are shown: FSC between two half-maps reconstructed from even and odd frames (evaluated at 0.143) and FSC between the final 3D map and the fitted model (evaluated at 0.5). (D) Zoomed-in views of the final 3D density map from panel A, displayed at two contour levels. (E) Superimposition of the high-contour level map from panel D onto its flexibly fitted model.

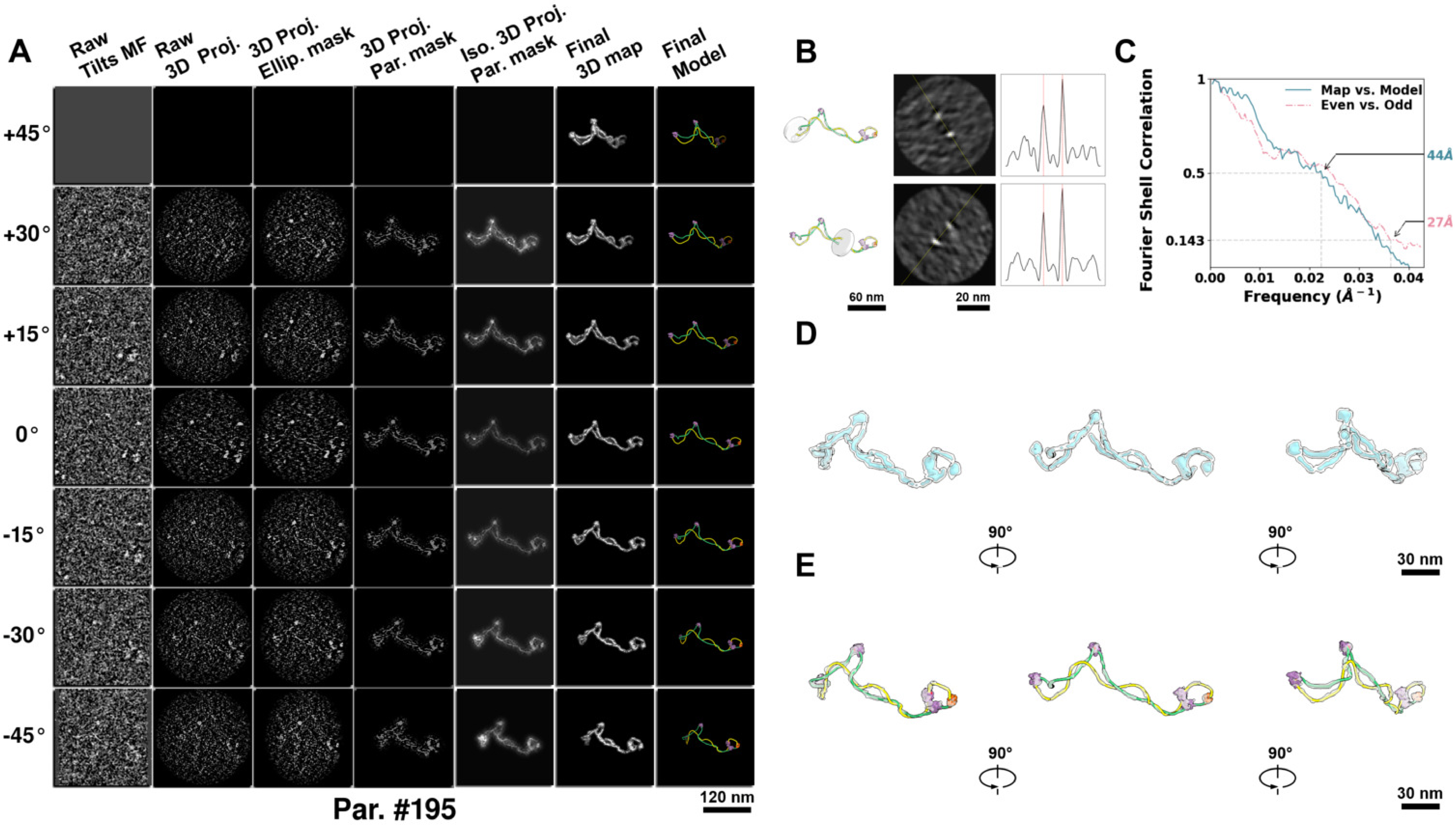

Supplementary Figure 195. Cryo-electron tomography (cryo-ET) 3D reconstruction of an individual plasmid particle in 40 mM K$^+$, 5 mM Mg^{2+} in the presence of transcribed RNAP and dCas9.
(A) 3D reconstruction of the plasmid particle (index no. 195). The first column shows seven representative tilt images from –45° to +45° in step of 15°. The second, third, and fourth columns show 3D projections of the particle with spherical, ellipsoidal (thinner along the z-dimension), and particle-shaped masks, respectively. The fifth column displays the 3D projections of the enhanced and IsoNet missing-wedge-corrected particle. The sixth and seventh columns present the final 3D map and the flexibly fitted model, respectively. (B) Two random cross-sectional views (12 nm thickness) of the plasmid density map along its plectoneme axis are shown in the left-middle panel. The intensity profile along the line crossing the two high-density DNA spots is displayed in the right panel. (C) Resolution assessment of the final 3D map using Fourier shell correlation (FSC). Two criteria are shown: FSC between two half-maps reconstructed from even and odd frames (evaluated at 0.143) and FSC between the final 3D map and the fitted model (evaluated at 0.5). (D) Zoomed-in views of the final 3D density map from panel A, displayed at two contour levels. (E) Superimposition of the high-contour level map from panel D onto its flexibly fitted model.

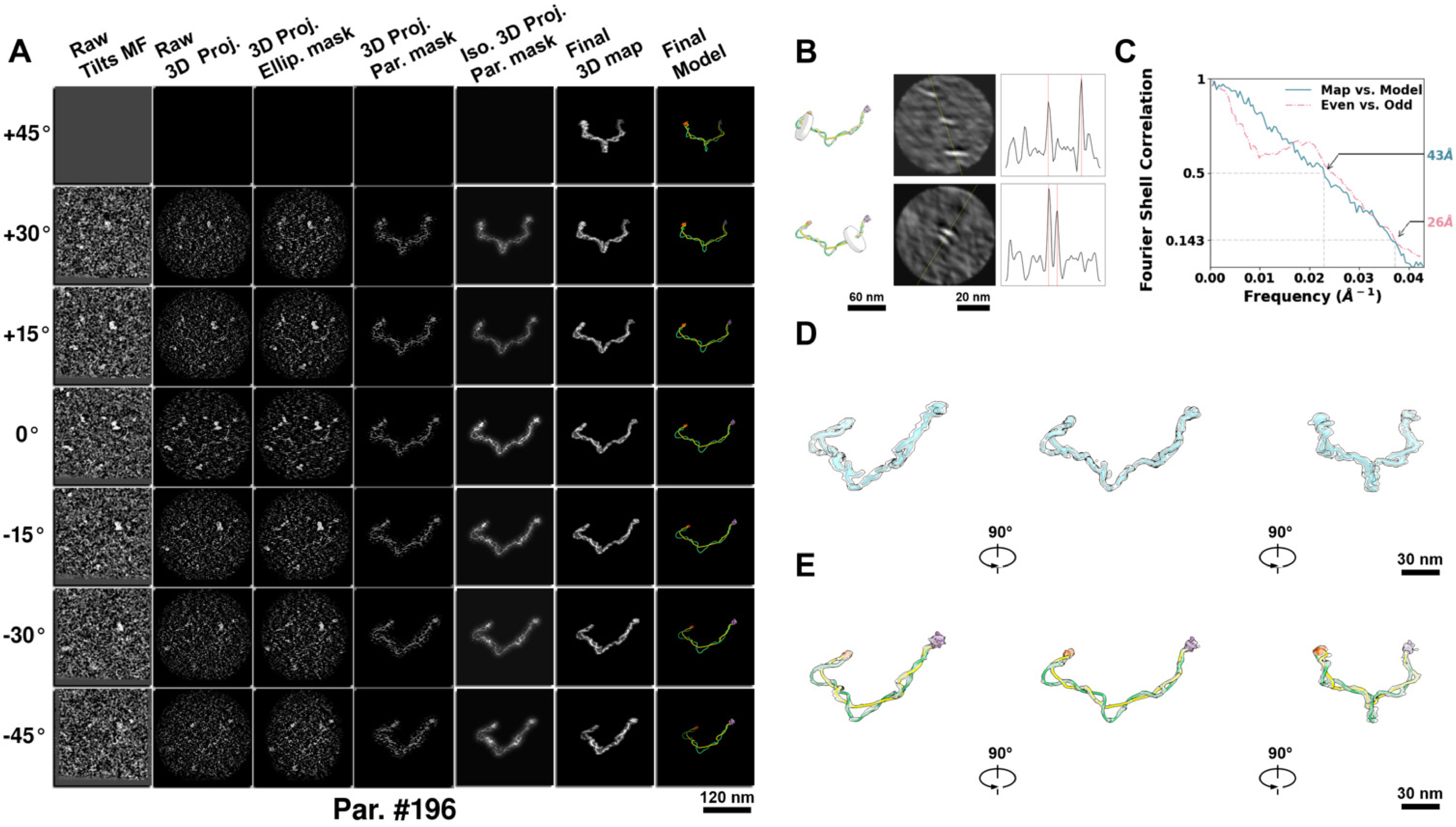

Supplementary Figure 196. Cryo-electron tomography (cryo-ET) 3D reconstruction of an individual plasmid particle in 40 mM K$^+$, 5 mM Mg^{2+} in the presence of transcribed RNAP and dCas9.
(A) 3D reconstruction of the plasmid particle (index no. 196). The first column shows seven representative tilt images from –45° to +45° in step of 15°. The second, third, and fourth columns show 3D projections of the particle with spherical, ellipsoidal (thinner along the z-dimension), and particle-shaped masks, respectively. The fifth column displays the 3D projections of the enhanced and IsoNet missing-wedge-corrected particle. The sixth and seventh columns present the final 3D map and the flexibly fitted model, respectively. (B) Two random cross-sectional views (12 nm thickness) of the plasmid density map along its plectoneme axis are shown in the left-middle panel. The intensity profile along the line crossing the two high-density DNA spots is displayed in the right panel. (C) Resolution assessment of the final 3D map using Fourier shell correlation (FSC). Two criteria are shown: FSC between two half-maps reconstructed from even and odd frames (evaluated at 0.143) and FSC between the final 3D map and the fitted model (evaluated at 0.5). (D) Zoomed-in views of the final 3D density map from panel A, displayed at two contour levels. (E) Superimposition of the high-contour level map from panel D onto its flexibly fitted model.

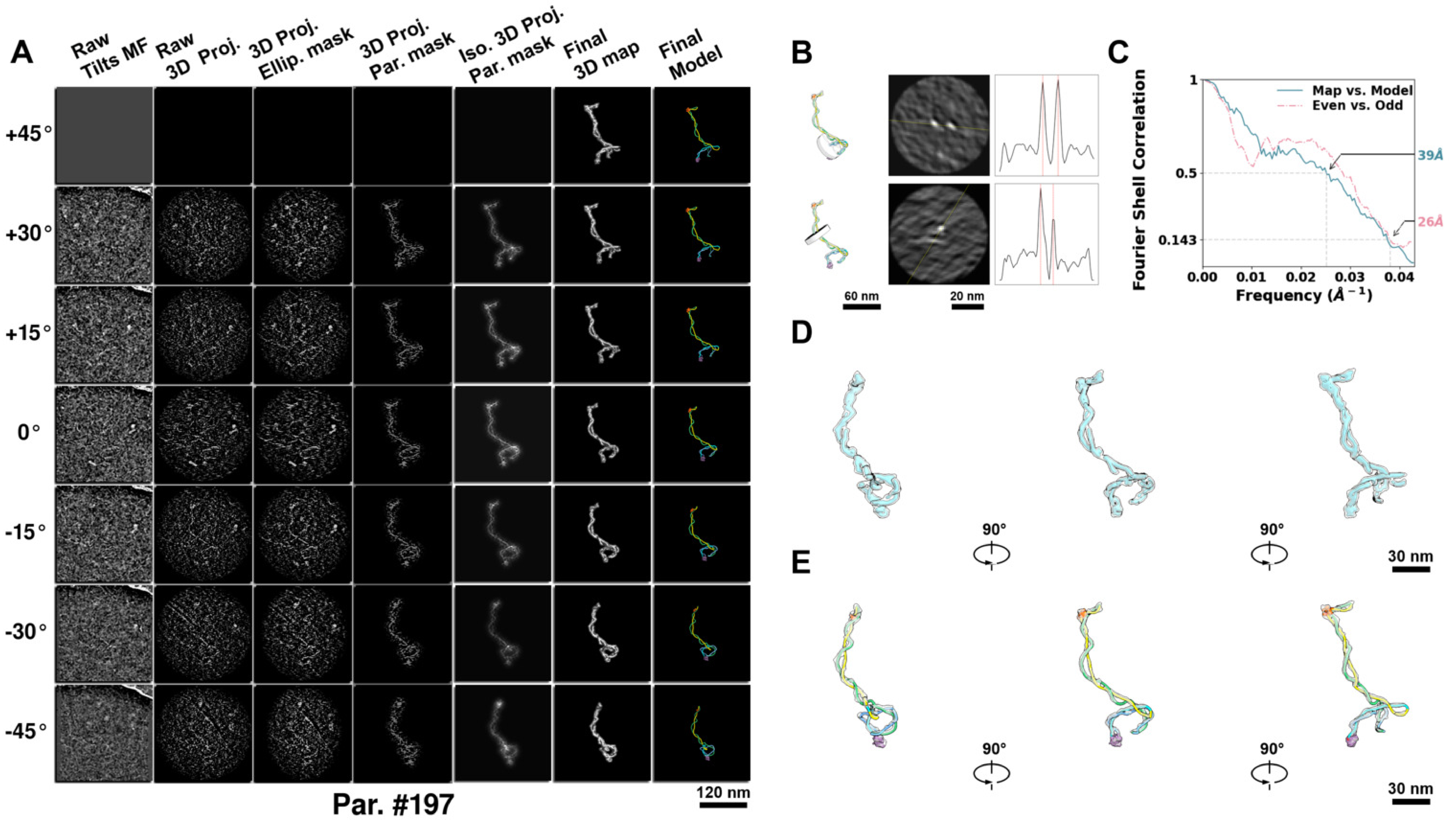

Supplementary Figure 197. Cryo-electron tomography (cryo-ET) 3D reconstruction of an individual plasmid particle in 40 mM K^+, 5 mM Mg^{2+} in the presence of transcribed RNAP and dCas9.
(A) 3D reconstruction of the plasmid particle (index no. 197). The first column shows seven representative tilt images from –45° to +45° in step of 15°. The second, third, and fourth columns show 3D projections of the particle with spherical, ellipsoidal (thinner along the z-dimension), and particle-shaped masks, respectively. The fifth column displays the 3D projections of the enhanced and IsoNet missing-wedge-corrected particle. The sixth and seventh columns present the final 3D map and the flexibly fitted model, respectively. (B) Two random cross-sectional views (12 nm thickness) of the plasmid density map along its plectoneme axis are shown in the left-middle panel. The intensity profile along the line crossing the two high-density DNA spots is displayed in the right panel. (C) Resolution assessment of the final 3D map using Fourier shell correlation (FSC). Two criteria are shown: FSC between two half-maps reconstructed from even and odd frames (evaluated at 0.143) and FSC between the final 3D map and the fitted model (evaluated at 0.5). (D) Zoomed-in views of the final 3D density map from panel A, displayed at two contour levels. (E) Superimposition of the high-contour level map from panel D onto its flexibly fitted model.

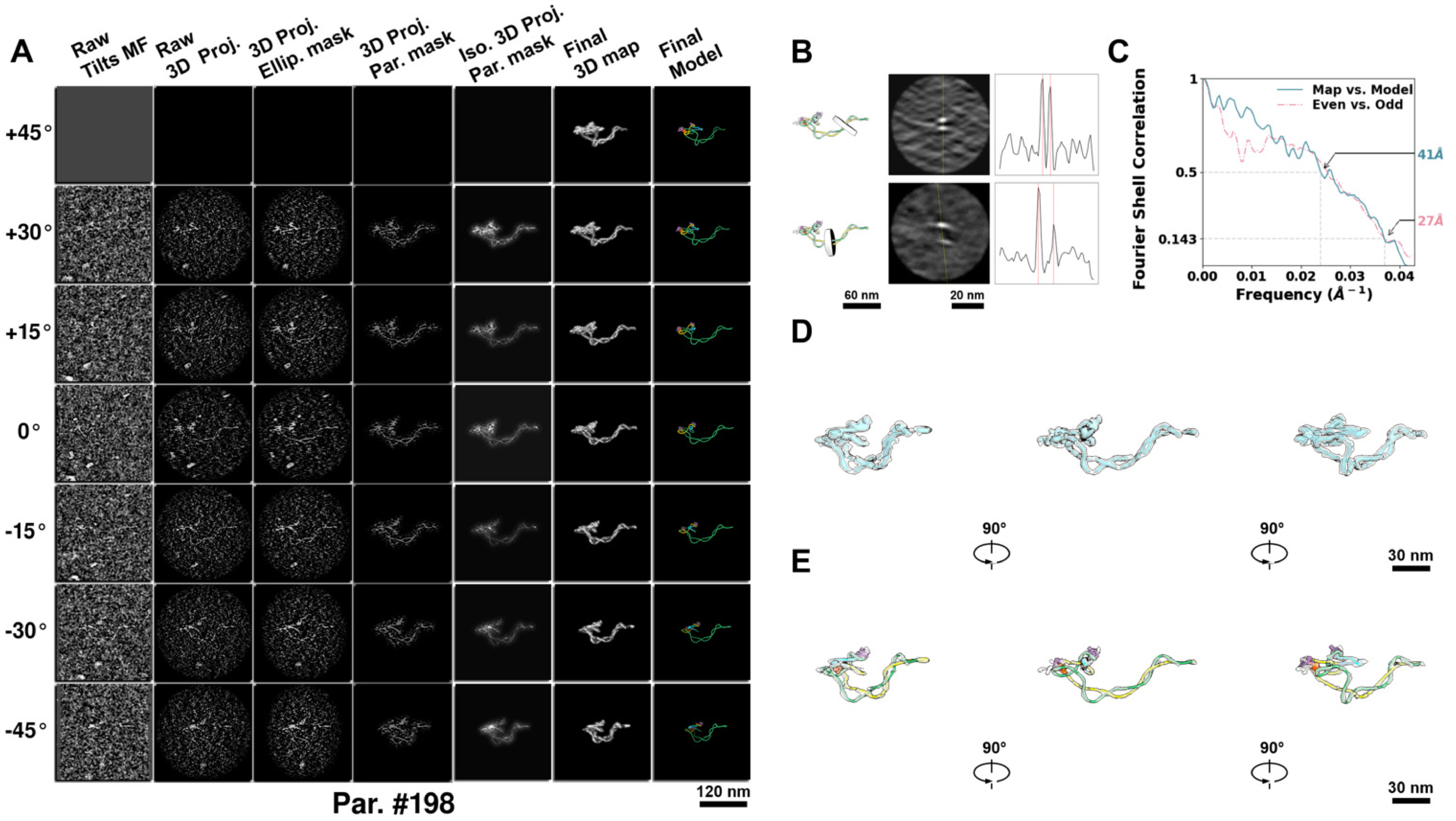

Supplementary Figure 198. Cryo-electron tomography (cryo-ET) 3D reconstruction of an individual plasmid particle in 40 mM K^+, 5 mM Mg^{2+} in the presence of transcribed RNAP and dCas9.
(A) 3D reconstruction of the plasmid particle (index no. 198). The first column shows seven representative tilt images from –45° to +45° in step of 15°. The second, third, and fourth columns show 3D projections of the particle with spherical, ellipsoidal (thinner along the z-dimension), and particle-shaped masks, respectively. The fifth column displays the 3D projections of the enhanced and IsoNet missing-wedge-corrected particle. The sixth and seventh columns present the final 3D map and the flexibly fitted model, respectively. (B) Two random cross-sectional views (12 nm thickness) of the plasmid density map along its plectoneme axis are shown in the left-middle panel. The intensity profile along the line crossing the two high-density DNA spots is displayed in the right panel. (C) Resolution assessment of the final 3D map using Fourier shell correlation (FSC). Two criteria are shown: FSC between two half-maps reconstructed from even and odd frames (evaluated at 0.143) and FSC between the final 3D map and the fitted model (evaluated at 0.5). (D) Zoomed-in views of the final 3D density map from panel A, displayed at two contour levels. (E) Superimposition of the high-contour level map from panel D onto its flexibly fitted model.

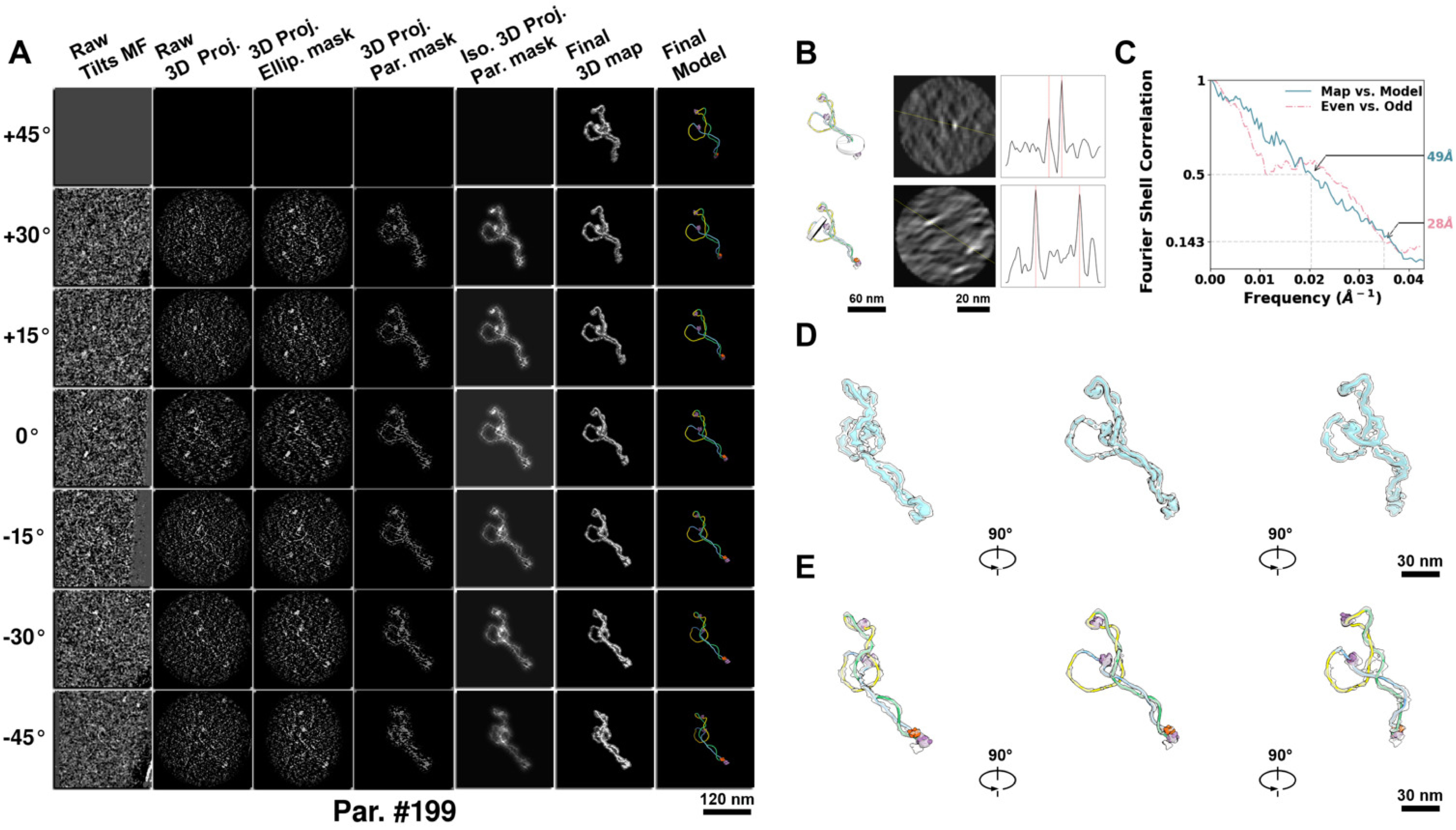

Supplementary Figure 199. Cryo-electron tomography (cryo-ET) 3D reconstruction of an individual plasmid particle in 40 mM K^+, 5 mM Mg^{2+} in the presence of transcribed RNAP and dCas9.
(A) 3D reconstruction of the plasmid particle (index no. 199). The first column shows seven representative tilt images from –45° to +45° in step of 15°. The second, third, and fourth columns show 3D projections of the particle with spherical, ellipsoidal (thinner along the z-dimension), and particle-shaped masks, respectively. The fifth column displays the 3D projections of the enhanced and IsoNet missing-wedge-corrected particle. The sixth and seventh columns present the final 3D map and the flexibly fitted model, respectively. (B) Two random cross-sectional views (12 nm thickness) of the plasmid density map along its plectoneme axis are shown in the left-middle panel. The intensity profile along the line crossing the two high-density DNA spots is displayed in the right panel. (C) Resolution assessment of the final 3D map using Fourier shell correlation (FSC). Two criteria are shown: FSC between two half-maps reconstructed from even and odd frames (evaluated at 0.143) and FSC between the final 3D map and the fitted model (evaluated at 0.5). (D) Zoomed-in views of the final 3D density map from panel A, displayed at two contour levels. (E) Superimposition of the high-contour level map from panel D onto its flexibly fitted model.

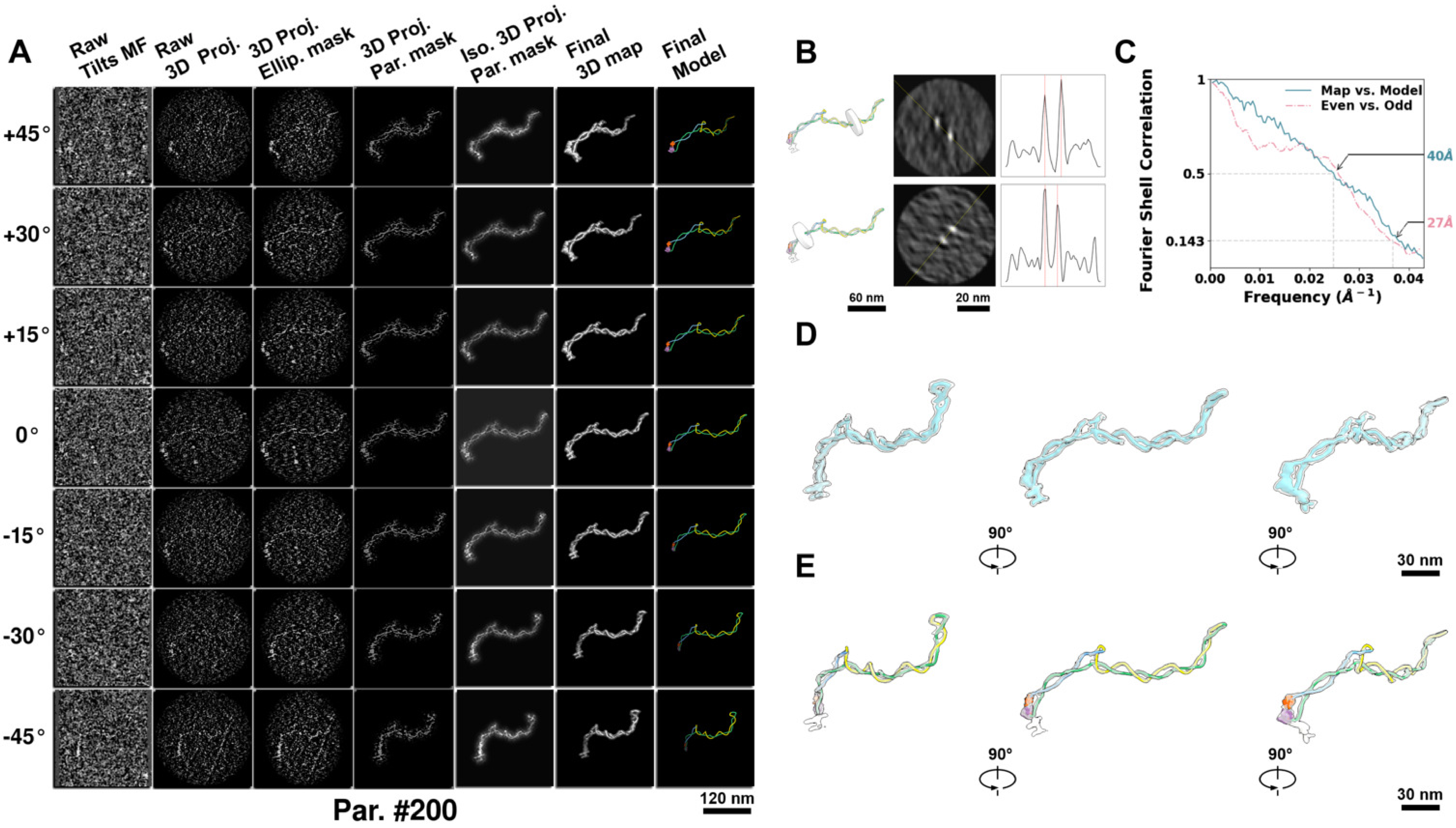

Supplementary Figure 200. Cryo-electron tomography (cryo-ET) 3D reconstruction of an individual plasmid particle in 40 mM K^+, 5 mM Mg^{2+} in the presence of transcribed RNAP and dCas9.
(A) 3D reconstruction of the plasmid particle (index no. 200). The first column shows seven representative tilt images from –45° to +45° in step of 15°. The second, third, and fourth columns show 3D projections of the particle with spherical, ellipsoidal (thinner along the z-dimension), and particle-shaped masks, respectively. The fifth column displays the 3D projections of the enhanced and IsoNet missing-wedge-corrected particle. The sixth and seventh columns present the final 3D map and the flexibly fitted model, respectively. (B) Two random cross-sectional views (12 nm thickness) of the plasmid density map along its plectoneme axis are shown in the left-middle panel. The intensity profile along the line crossing the two high-density DNA spots is displayed in the right panel. (C) Resolution assessment of the final 3D map using Fourier shell correlation (FSC). Two criteria are shown: FSC between two half-maps reconstructed from even and odd frames (evaluated at 0.143) and FSC between the final 3D map and the fitted model (evaluated at 0.5). (D) Zoomed-in views of the final 3D density map from panel A, displayed at two contour levels. (E) Superimposition of the high-contour level map from panel D onto its flexibly fitted model.

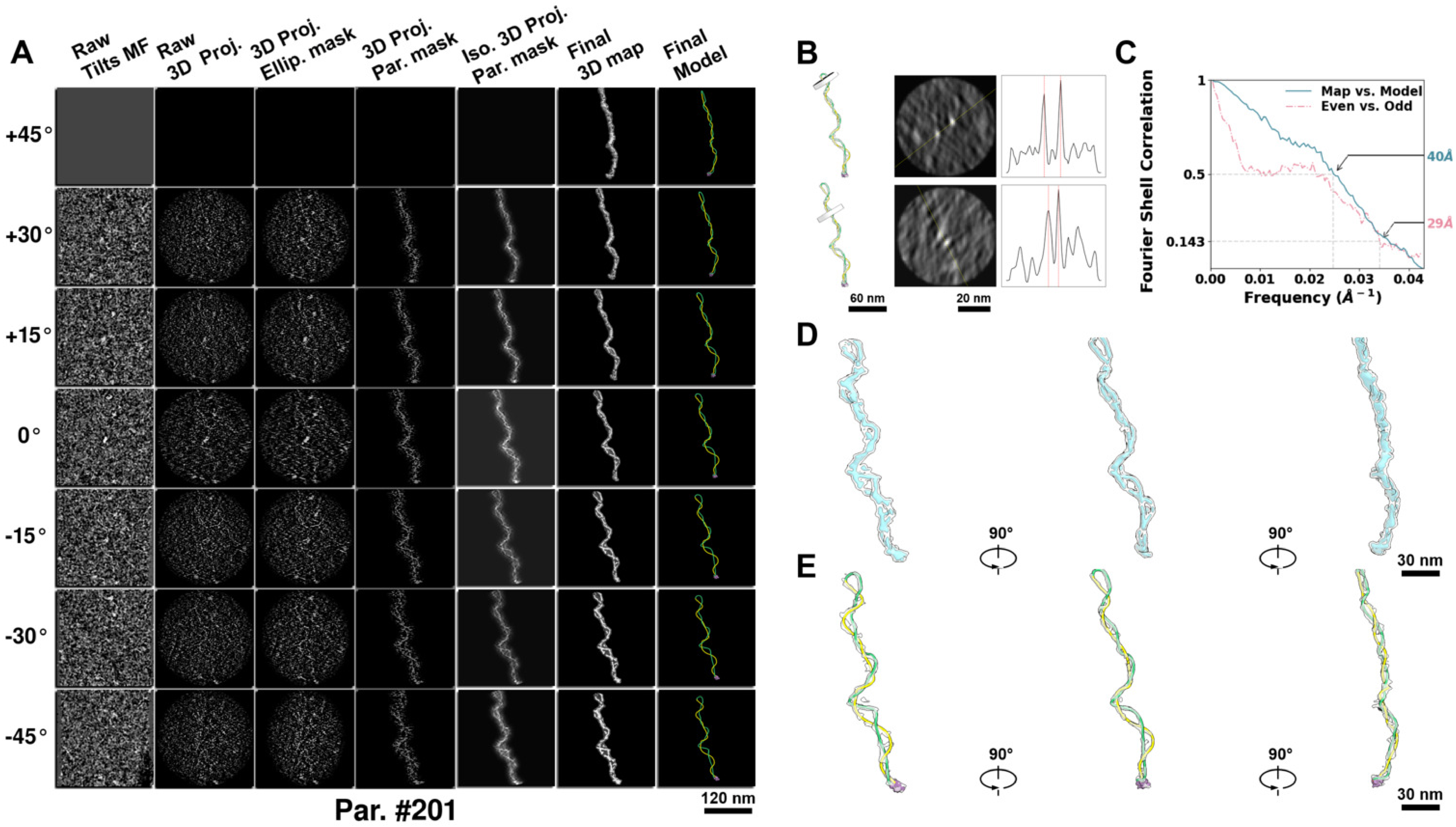

Supplementary Figure 201. Cryo-electron tomography (cryo-ET) 3D reconstruction of an individual plasmid particle in 40 mM K$^+$, 5 mM Mg^{2+} in the presence of transcribed RNAP and dCas9.
(A) 3D reconstruction of the plasmid particle (index no. 201). The first column shows seven representative tilt images from –45° to +45° in step of 15°. The second, third, and fourth columns show 3D projections of the particle with spherical, ellipsoidal (thinner along the z-dimension), and particle-shaped masks, respectively. The fifth column displays the 3D projections of the enhanced and IsoNet missing-wedge-corrected particle. The sixth and seventh columns present the final 3D map and the flexibly fitted model, respectively. (B) Two random cross-sectional views (12 nm thickness) of the plasmid density map along its plectoneme axis are shown in the left-middle panel. The intensity profile along the line crossing the two high-density DNA spots is displayed in the right panel. (C) Resolution assessment of the final 3D map using Fourier shell correlation (FSC). Two criteria are shown: FSC between two half-maps reconstructed from even and odd frames (evaluated at 0.143) and FSC between the final 3D map and the fitted model (evaluated at 0.5). (D) Zoomed-in views of the final 3D density map from panel A, displayed at two contour levels. (E) Superimposition of the high-contour level map from panel D onto its flexibly fitted model.

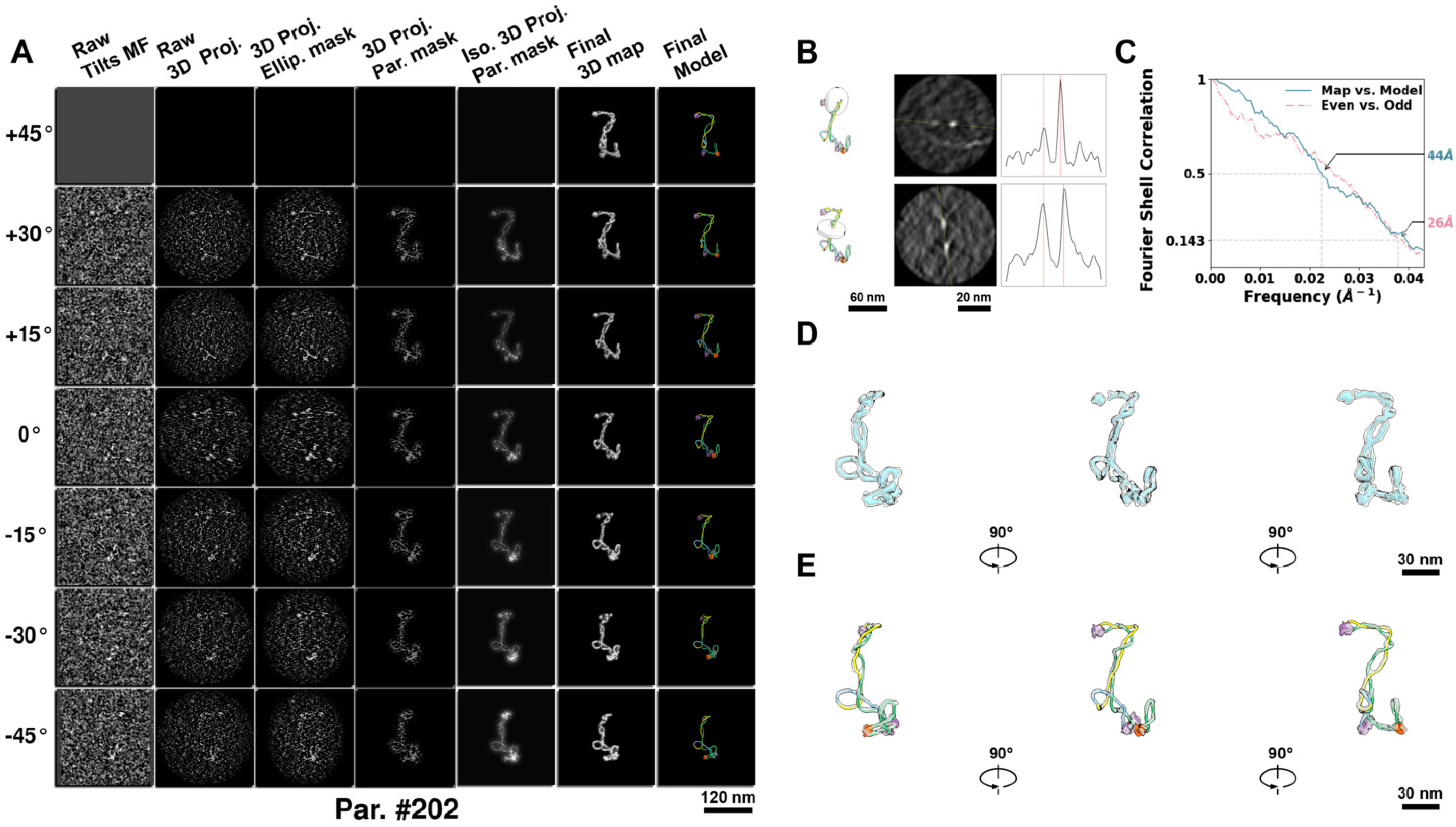

Supplementary Figure 202. Cryo-electron tomography (cryo-ET) 3D reconstruction of an individual plasmid particle in 40 mM K$^+$, 5 mM Mg^{2+} in the presence of transcribed RNAP and dCas9.
(A) 3D reconstruction of the plasmid particle (index no. 202). The first column shows seven representative tilt images from –45° to +45° in step of 15°. The second, third, and fourth columns show 3D projections of the particle with spherical, ellipsoidal (thinner along the z-dimension), and particle-shaped masks, respectively. The fifth column displays the 3D projections of the enhanced and IsoNet missing-wedge-corrected particle. The sixth and seventh columns present the final 3D map and the flexibly fitted model, respectively. (B) Two random cross-sectional views (12 nm thickness) of the plasmid density map along its plectoneme axis are shown in the left-middle panel. The intensity profile along the line crossing the two high-density DNA spots is displayed in the right panel. (C) Resolution assessment of the final 3D map using Fourier shell correlation (FSC). Two criteria are shown: FSC between two half-maps reconstructed from even and odd frames (evaluated at 0.143) and FSC between the final 3D map and the fitted model (evaluated at 0.5). (D) Zoomed-in views of the final 3D density map from panel A, displayed at two contour levels. (E) Superimposition of the high-contour level map from panel D onto its flexibly fitted model.

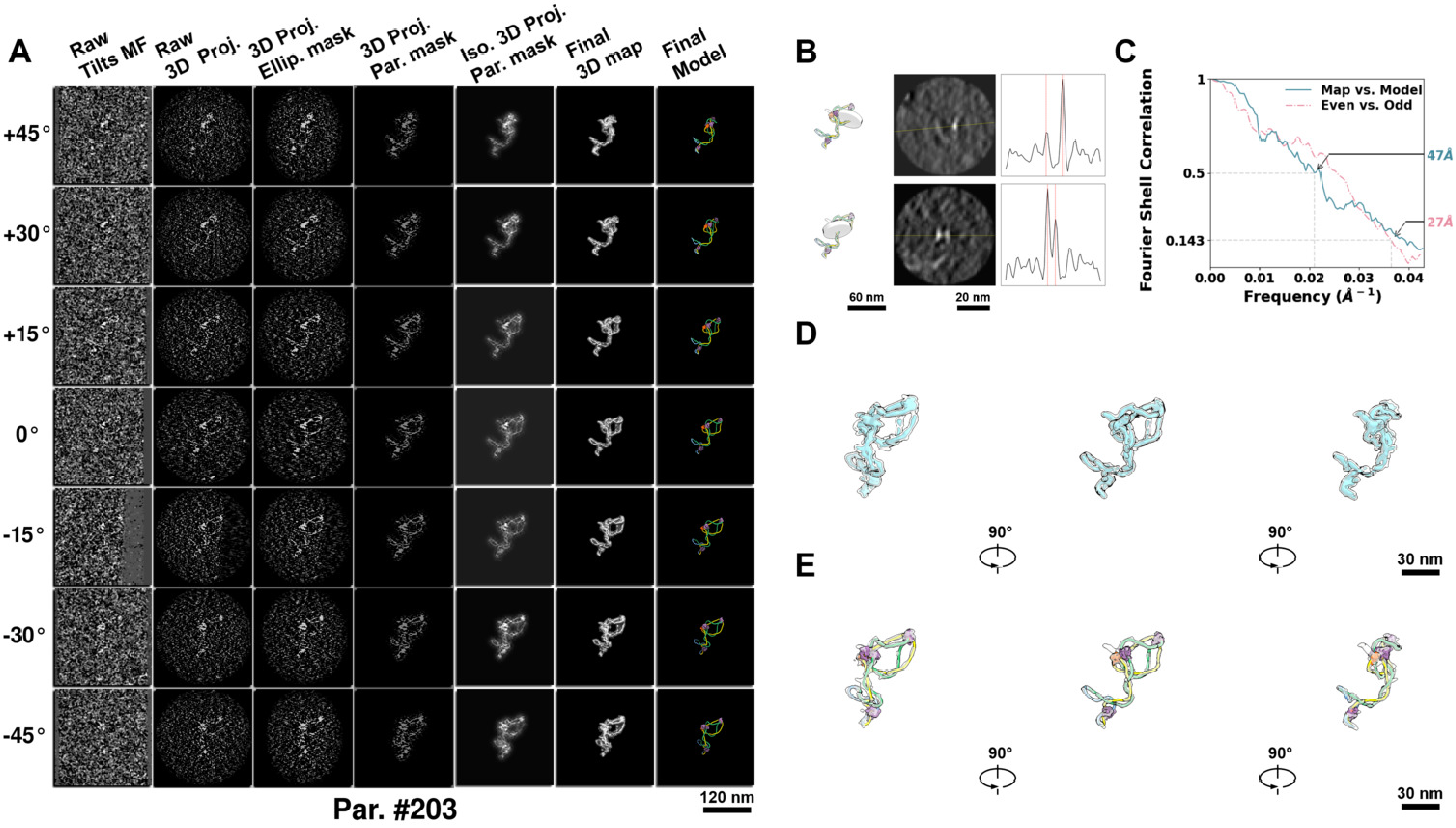

Supplementary Figure 203. Cryo-electron tomography (cryo-ET) 3D reconstruction of an individual plasmid particle in 40 mM K⁺, 5 mM Mg²⁺ in the presence of transcribed RNAP and dCas9.
(A) 3D reconstruction of the plasmid particle (index no. 203). The first column shows seven representative tilt images from –45° to +45° in step of 15°. The second, third, and fourth columns show 3D projections of the particle with spherical, ellipsoidal (thinner along the z-dimension), and particle-shaped masks, respectively. The fifth column displays the 3D projections of the enhanced and IsoNet missing-wedge-corrected particle. The sixth and seventh columns present the final 3D map and the flexibly fitted model, respectively. (B) Two random cross-sectional views (12 nm thickness) of the plasmid density map along its plectoneme axis are shown in the left-middle panel. The intensity profile along the line crossing the two high-density DNA spots is displayed in the right panel. (C) Resolution assessment of the final 3D map using Fourier shell correlation (FSC). Two criteria are shown: FSC between two half-maps reconstructed from even and odd frames (evaluated at 0.143) and FSC between the final 3D map and the fitted model (evaluated at 0.5). (D) Zoomed-in views of the final 3D density map from panel A, displayed at two contour levels. (E) Superimposition of the high-contour level map from panel D onto its flexibly fitted model.

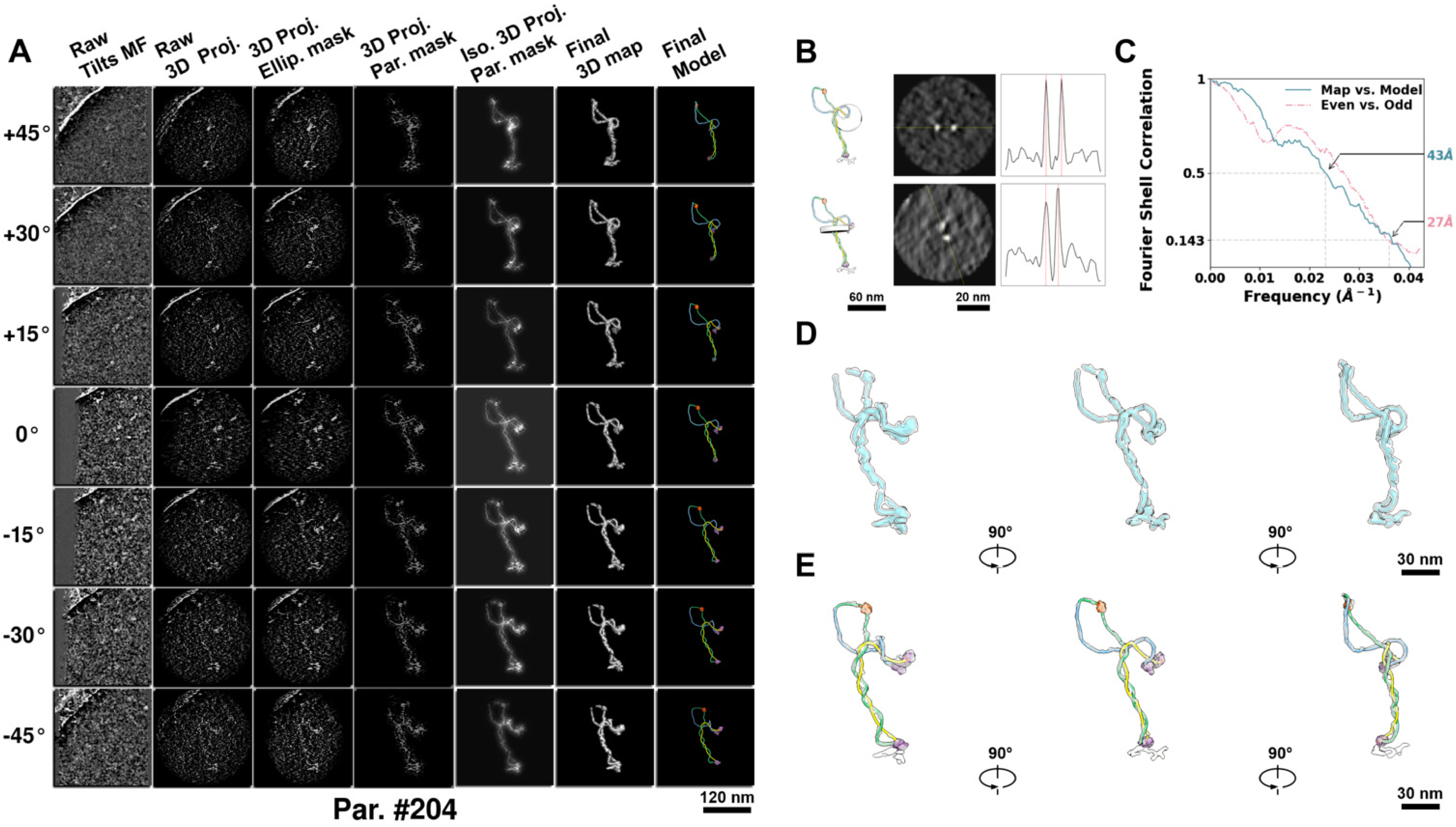

Supplementary Figure 204. Cryo-electron tomography (cryo-ET) 3D reconstruction of an individual plasmid particle in 40 mM K⁺, 5 mM Mg²⁺ in the presence of transcribed RNAP and dCas9.
(A) 3D reconstruction of the plasmid particle (index no. 204). The first column shows seven representative tilt images from –45° to +45° in step of 15°. The second, third, and fourth columns show 3D projections of the particle with spherical, ellipsoidal (thinner along the z-dimension), and particle-shaped masks, respectively. The fifth column displays the 3D projections of the enhanced and IsoNet missing-wedge-corrected particle. The sixth and seventh columns present the final 3D map and the flexibly fitted model, respectively. (B) Two random cross-sectional views (12 nm thickness) of the plasmid density map along its plectoneme axis are shown in the left-middle panel. The intensity profile along the line crossing the two high-density DNA spots is displayed in the right panel. (C) Resolution assessment of the final 3D map using Fourier shell correlation (FSC). Two criteria are shown: FSC between two half-maps reconstructed from even and odd frames (evaluated at 0.143) and FSC between the final 3D map and the fitted model (evaluated at 0.5). (D) Zoomed-in views of the final 3D density map from panel A, displayed at two contour levels. (E) Superimposition of the high-contour level map from panel D onto its flexibly fitted model.

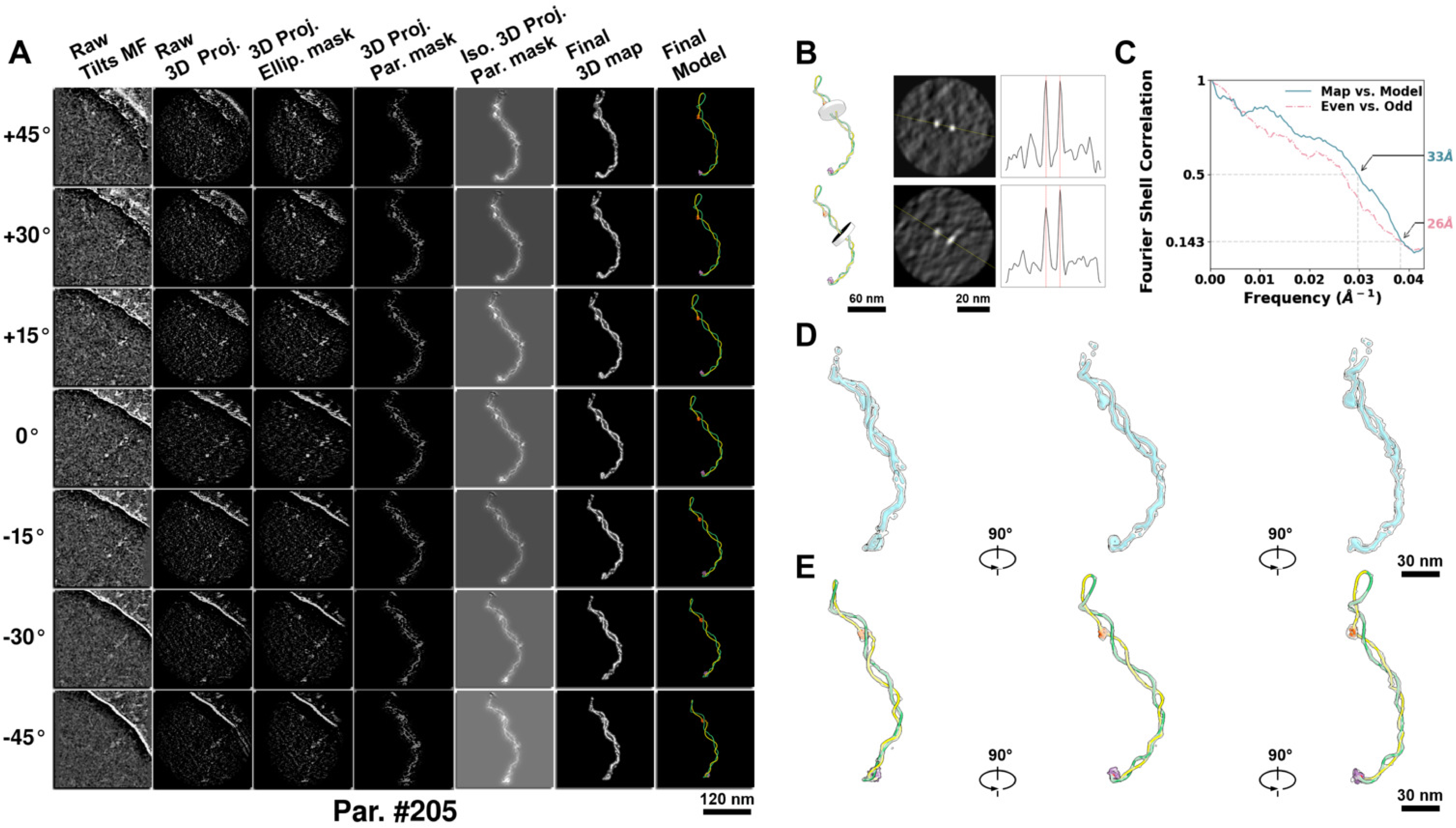

Supplementary Figure 205. Cryo-electron tomography (cryo-ET) 3D reconstruction of an individual plasmid particle in 40 mM K⁺, 5 mM Mg²⁺ in the presence of transcribed RNAP and dCas9.
(A) 3D reconstruction of the plasmid particle (index no. 205). The first column shows seven representative tilt images from –45° to +45° in step of 15°. The second, third, and fourth columns show 3D projections of the particle with spherical, ellipsoidal (thinner along the z-dimension), and particle-shaped masks, respectively. The fifth column displays the 3D projections of the enhanced and IsoNet missing-wedge-corrected particle. The sixth and seventh columns present the final 3D map and the flexibly fitted model, respectively. (B) Two random cross-sectional views (12 nm thickness) of the plasmid density map along its plectoneme axis are shown in the left-middle panel. The intensity profile along the line crossing the two high-density DNA spots is displayed in the right panel. (C) Resolution assessment of the final 3D map using Fourier shell correlation (FSC). Two criteria are shown: FSC between two half-maps reconstructed from even and odd frames (evaluated at 0.143) and FSC between the final 3D map and the fitted model (evaluated at 0.5). (D) Zoomed-in views of the final 3D density map from panel A, displayed at two contour levels. (E) Superimposition of the high-contour level map from panel D onto its flexibly fitted model.

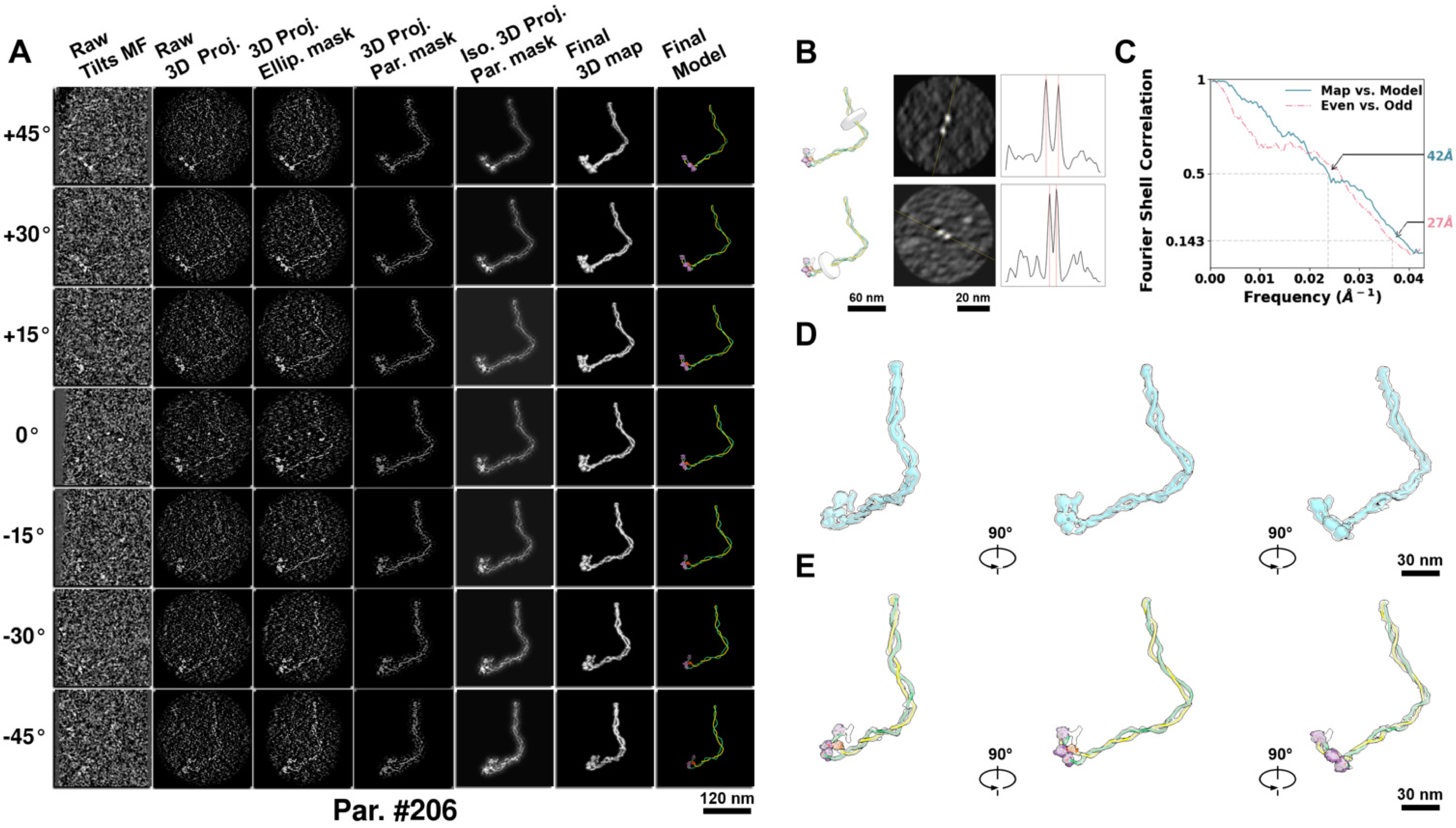

Supplementary Figure 206. Cryo-electron tomography (cryo-ET) 3D reconstruction of an individual plasmid particle in 40 mM K⁺, 5 mM Mg²⁺ in the presence of transcribed RNAP and dCas9.
(A) 3D reconstruction of the plasmid particle (index no. 206). The first column shows seven representative tilt images from –45° to +45° in step of 15°. The second, third, and fourth columns show 3D projections of the particle with spherical, ellipsoidal (thinner along the z-dimension), and particle-shaped masks, respectively. The fifth column displays the 3D projections of the enhanced and IsoNet missing-wedge-corrected particle. The sixth and seventh columns present the final 3D map and the flexibly fitted model, respectively. (B) Two random cross-sectional views (12 nm thickness) of the plasmid density map along its plectoneme axis are shown in the left-middle panel. The intensity profile along the line crossing the two high-density DNA spots is displayed in the right panel. (C) Resolution assessment of the final 3D map using Fourier shell correlation (FSC). Two criteria are shown: FSC between two half-maps reconstructed from even and odd frames (evaluated at 0.143) and FSC between the final 3D map and the fitted model (evaluated at 0.5). (D) Zoomed-in views of the final 3D density map from panel A, displayed at two contour levels. (E) Superimposition of the high-contour level map from panel D onto its flexibly fitted model.

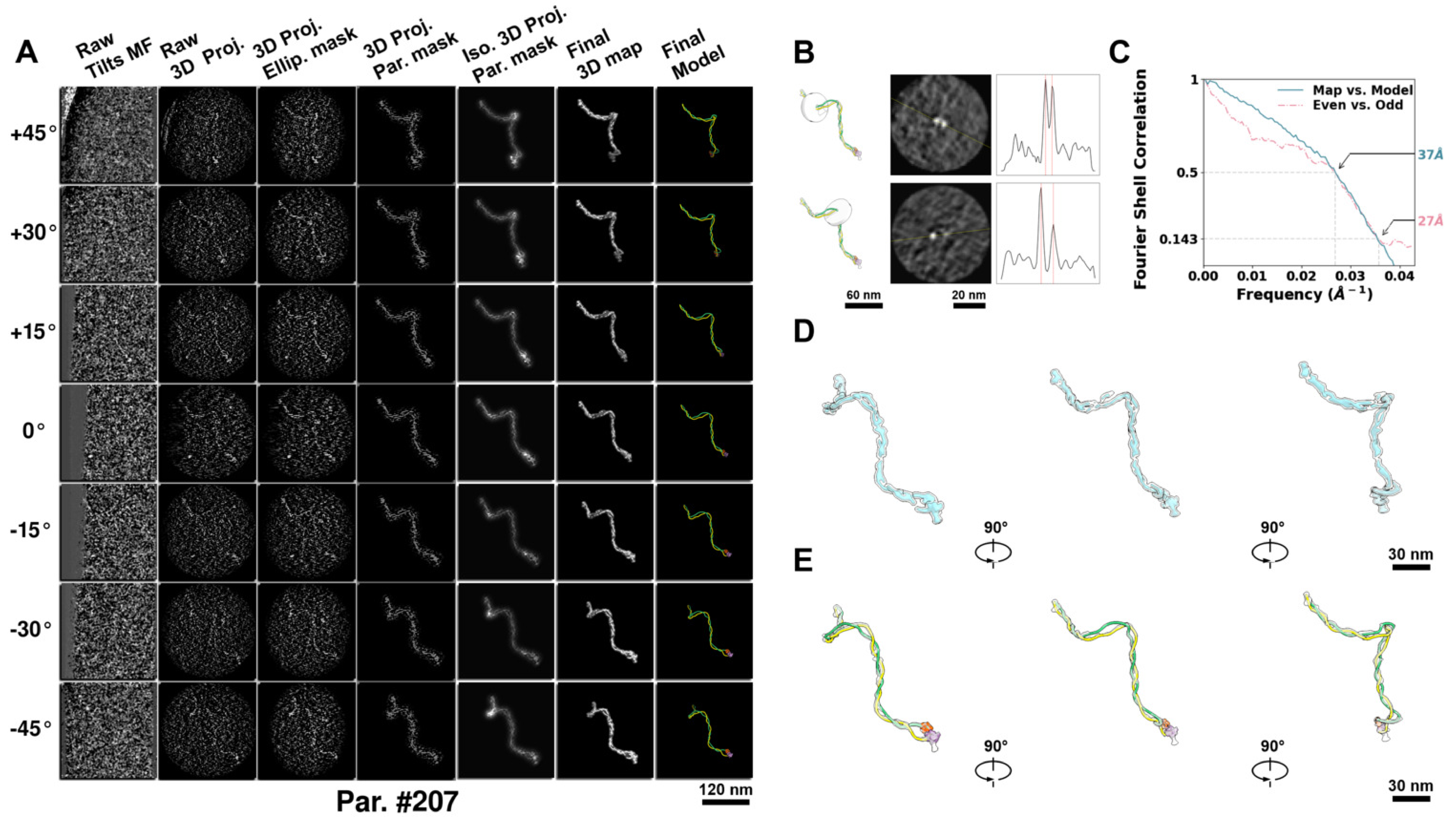

Supplementary Figure 207. Cryo-electron tomography (cryo-ET) 3D reconstruction of an individual plasmid particle in 40 mM K^{+}, 5 mM Mg^{2+} in the presence of transcribed RNAP and dCas9.
(A) 3D reconstruction of the plasmid particle (index no. 207). The first column shows seven representative tilt images from –45° to +45° in step of 15°. The second, third, and fourth columns show 3D projections of the particle with spherical, ellipsoidal (thinner along the z-dimension), and particle-shaped masks, respectively. The fifth column displays the 3D projections of the enhanced and IsoNet missing-wedge-corrected particle. The sixth and seventh columns present the final 3D map and the flexibly fitted model, respectively. (B) Two random cross-sectional views (12 nm thickness) of the plasmid density map along its plectoneme axis are shown in the left-middle panel. The intensity profile along the line crossing the two high-density DNA spots is displayed in the right panel. (C) Resolution assessment of the final 3D map using Fourier shell correlation (FSC). Two criteria are shown: FSC between two half-maps reconstructed from even and odd frames (evaluated at 0.143) and FSC between the final 3D map and the fitted model (evaluated at 0.5). (D) Zoomed-in views of the final 3D density map from panel A, displayed at two contour levels. (E) Superimposition of the high-contour level map from panel D onto its flexibly fitted model.

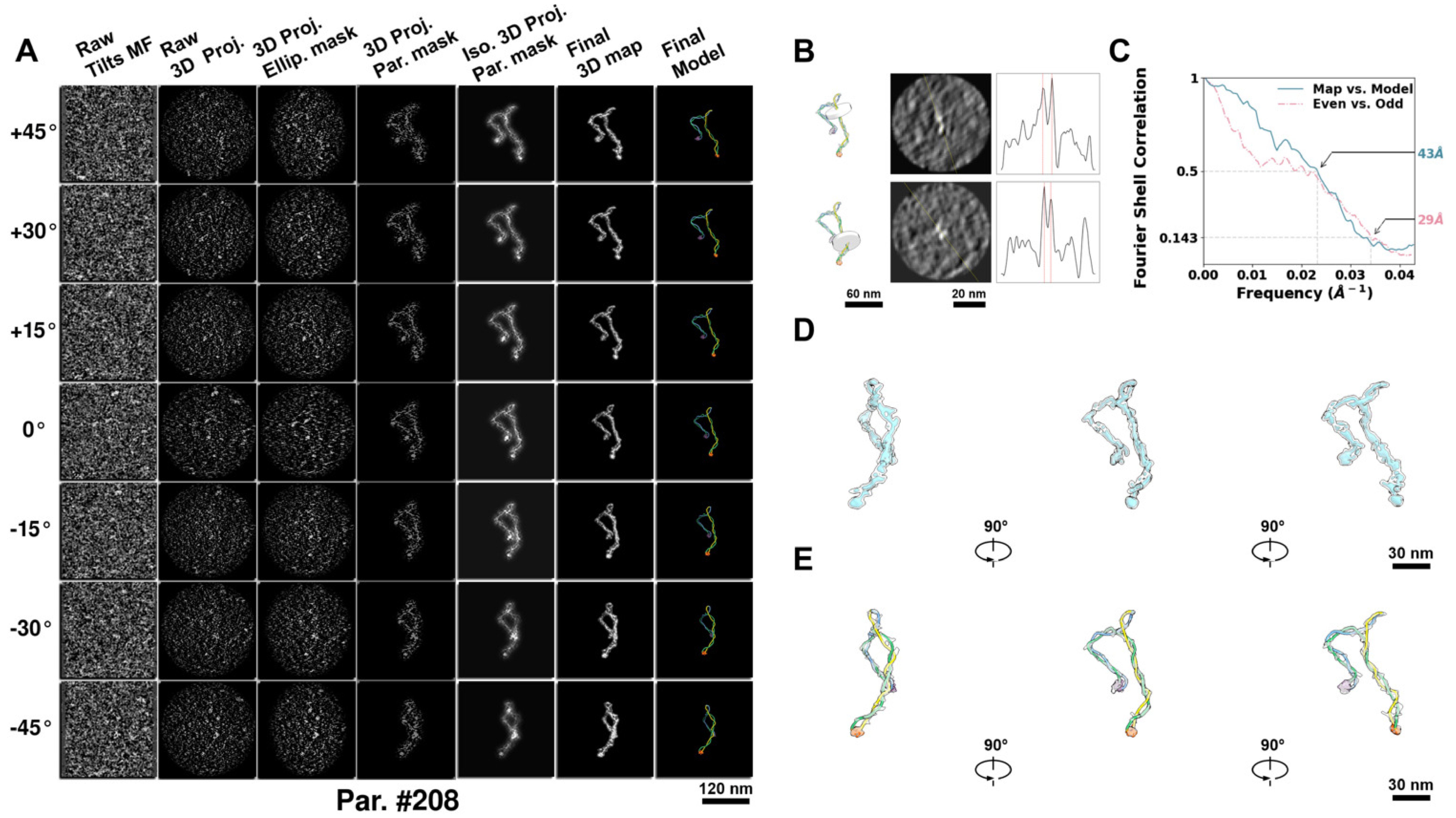

Supplementary Figure 208. Cryo-electron tomography (cryo-ET) 3D reconstruction of an individual plasmid particle in 40 mM K^{+}, 5 mM Mg^{2+} in the presence of transcribed RNAP and dCas9.
(A) 3D reconstruction of the plasmid particle (index no. 208). The first column shows seven representative tilt images from –45° to +45° in step of 15°. The second, third, and fourth columns show 3D projections of the particle with spherical, ellipsoidal (thinner along the z-dimension), and particle-shaped masks, respectively. The fifth column displays the 3D projections of the enhanced and IsoNet missing-wedge-corrected particle. The sixth and seventh columns present the final 3D map and the flexibly fitted model, respectively. (B) Two random cross-sectional views (12 nm thickness) of the plasmid density map along its plectoneme axis are shown in the left-middle panel. The intensity profile along the line crossing the two high-density DNA spots is displayed in the right panel. (C) Resolution assessment of the final 3D map using Fourier shell correlation (FSC). Two criteria are shown: FSC between two half-maps reconstructed from even and odd frames (evaluated at 0.143) and FSC between the final 3D map and the fitted model (evaluated at 0.5). (D) Zoomed-in views of the final 3D density map from panel A, displayed at two contour levels. (E) Superimposition of the high-contour level map from panel D onto its flexibly fitted model.

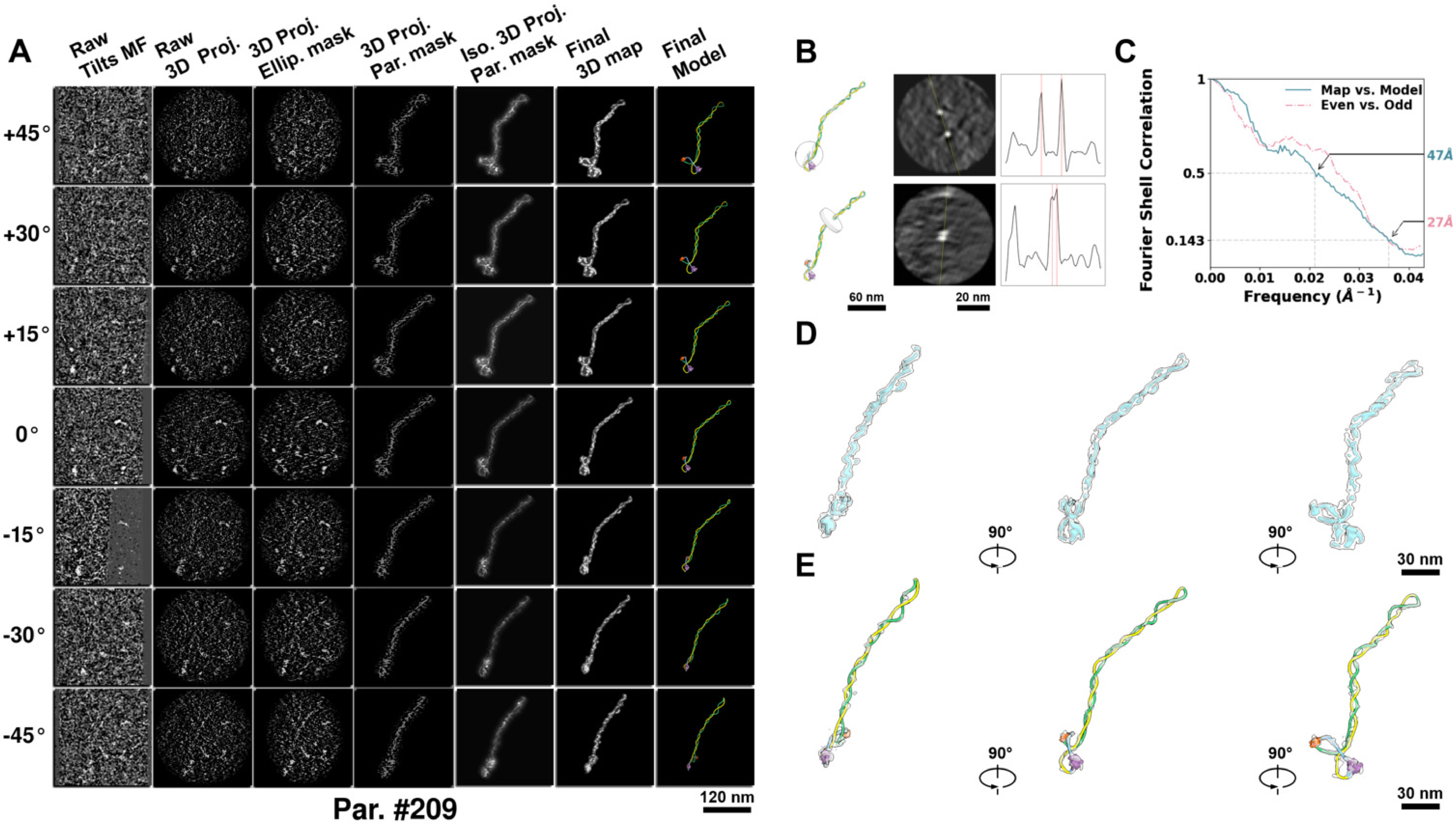

Supplementary Figure 209. Cryo-electron tomography (cryo-ET) 3D reconstruction of an individual plasmid particle in 40 mM K^+, 5 mM Mg^{2+} in the presence of transcribed RNAP and dCas9.
(A) 3D reconstruction of the plasmid particle (index no. 209). The first column shows seven representative tilt images from –45° to +45° in step of 15°. The second, third, and fourth columns show 3D projections of the particle with spherical, ellipsoidal (thinner along the z-dimension), and particle-shaped masks, respectively. The fifth column displays the 3D projections of the enhanced and IsoNet missing-wedge-corrected particle. The sixth and seventh columns present the final 3D map and the flexibly fitted model, respectively. (B) Two random cross-sectional views (12 nm thickness) of the plasmid density map along its plectoneme axis are shown in the left-middle panel. The intensity profile along the line crossing the two high-density DNA spots is displayed in the right panel. (C) Resolution assessment of the final 3D map using Fourier shell correlation (FSC). Two criteria are shown: FSC between two half-maps reconstructed from even and odd frames (evaluated at 0.143) and FSC between the final 3D map and the fitted model (evaluated at 0.5). (D) Zoomed-in views of the final 3D density map from panel A, displayed at two contour levels. (E) Superimposition of the high-contour level map from panel D onto its flexibly fitted model.

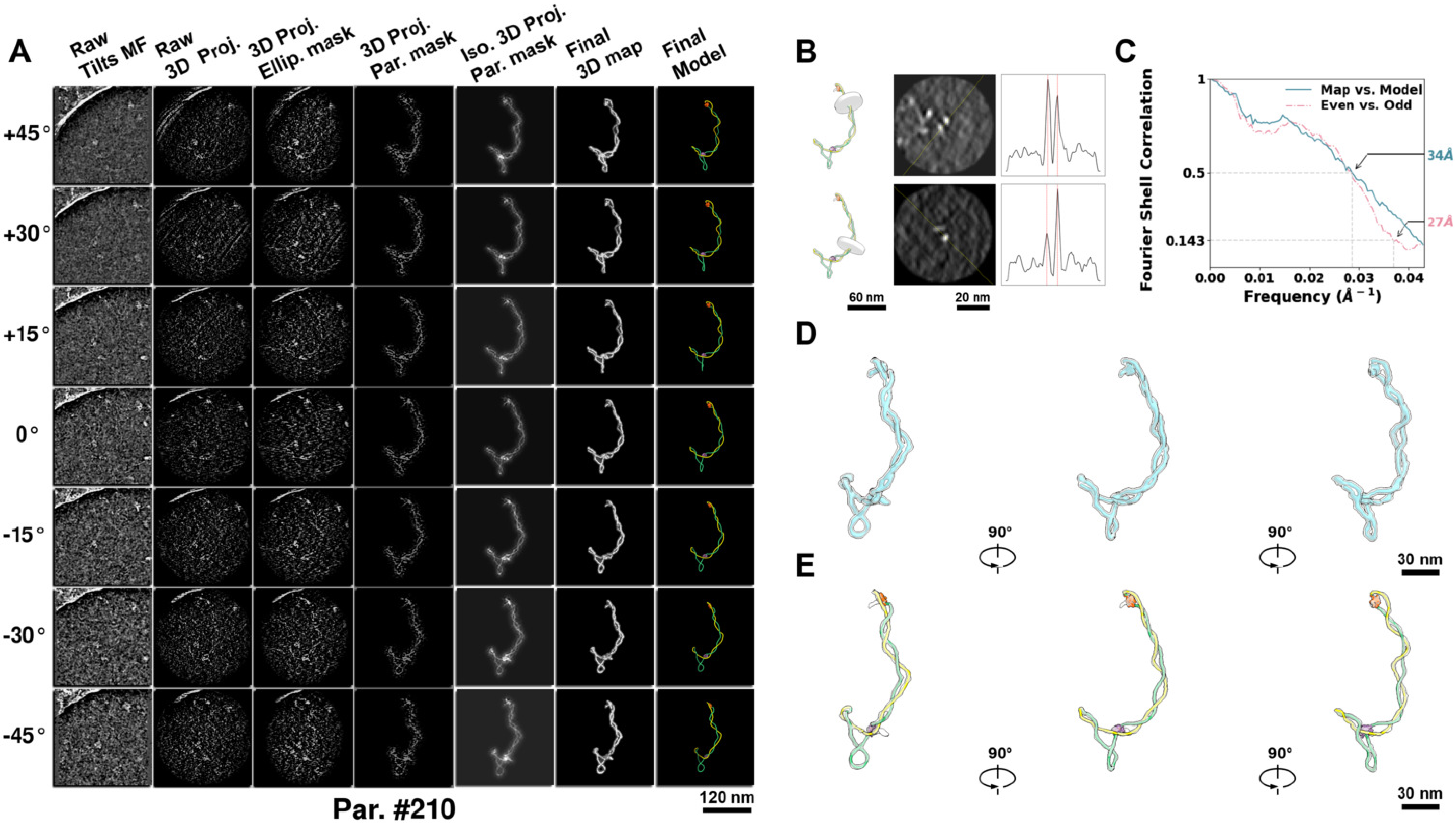

Supplementary Figure 210. Cryo-electron tomography (cryo-ET) 3D reconstruction of an individual plasmid particle in 40 mM K^+, 5 mM Mg^{2+} in the presence of transcribed RNAP and dCas9.
(A) 3D reconstruction of the plasmid particle (index no. 210). The first column shows seven representative tilt images from –45° to +45° in step of 15°. The second, third, and fourth columns show 3D projections of the particle with spherical, ellipsoidal (thinner along the z-dimension), and particle-shaped masks, respectively. The fifth column displays the 3D projections of the enhanced and IsoNet missing-wedge-corrected particle. The sixth and seventh columns present the final 3D map and the flexibly fitted model, respectively. (B) Two random cross-sectional views (12 nm thickness) of the plasmid density map along its plectoneme axis are shown in the left-middle panel. The intensity profile along the line crossing the two high-density DNA spots is displayed in the right panel. (C) Resolution assessment of the final 3D map using Fourier shell correlation (FSC). Two criteria are shown: FSC between two half-maps reconstructed from even and odd frames (evaluated at 0.143) and FSC between the final 3D map and the fitted model (evaluated at 0.5). (D) Zoomed-in views of the final 3D density map from panel A, displayed at two contour levels. (E) Superimposition of the high-contour level map from panel D onto its flexibly fitted model.

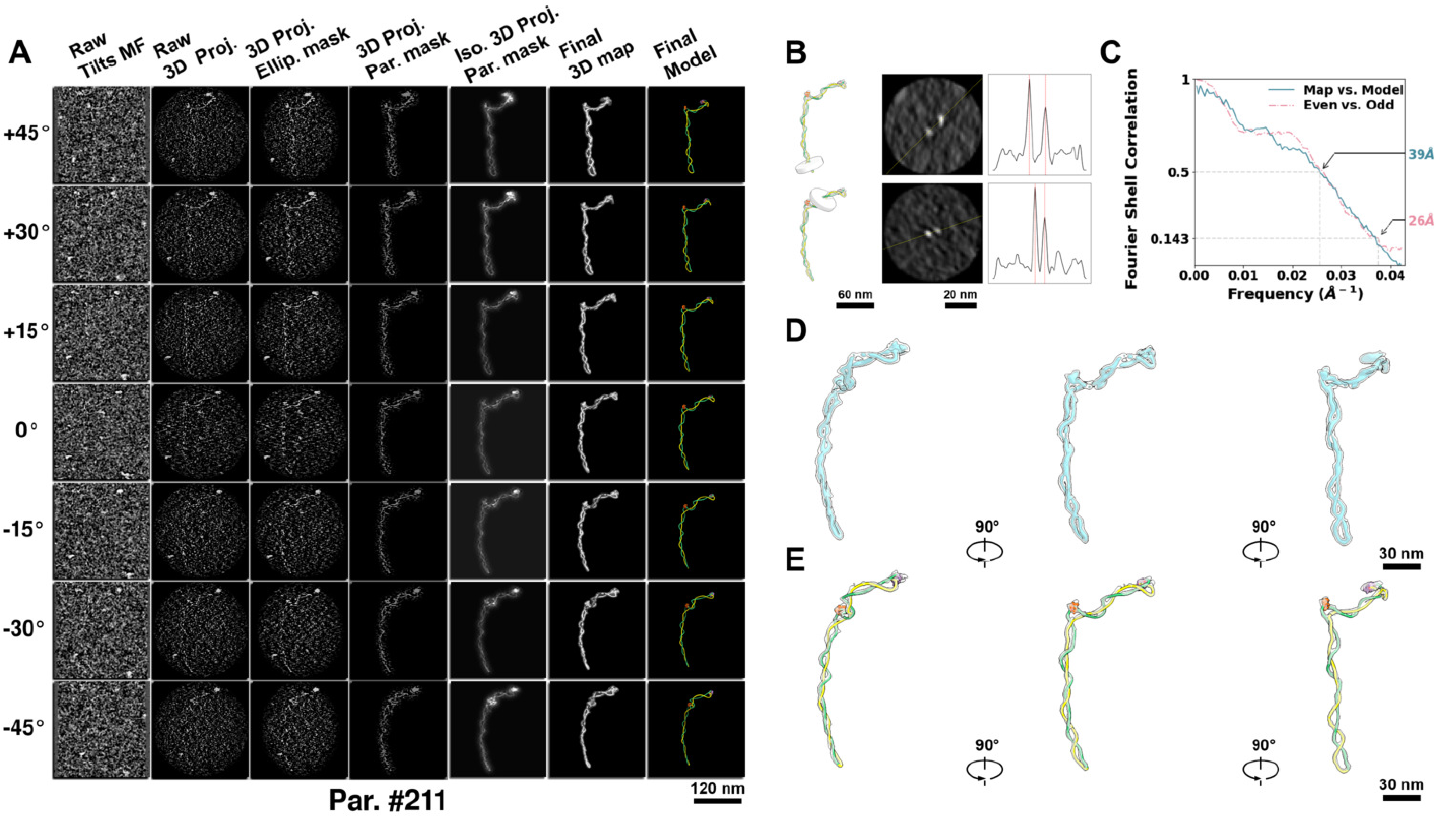

Supplementary Figure 211. Cryo-electron tomography (cryo-ET) 3D reconstruction of an individual plasmid particle in 40 mM K⁺, 5 mM Mg²⁺ in the presence of transcribed RNAP and dCas9.
(A) 3D reconstruction of the plasmid particle (index no. 211). The first column shows seven representative tilt images from –45° to +45° in step of 15°. The second, third, and fourth columns show 3D projections of the particle with spherical, ellipsoidal (thinner along the z-dimension), and particle-shaped masks, respectively. The fifth column displays the 3D projections of the enhanced and IsoNet missing-wedge-corrected particle. The sixth and seventh columns present the final 3D map and the flexibly fitted model, respectively. (B) Two random cross-sectional views (12 nm thickness) of the plasmid density map along its plectoneme axis are shown in the left-middle panel. The intensity profile along the line crossing the two high-density DNA spots is displayed in the right panel. (C) Resolution assessment of the final 3D map using Fourier shell correlation (FSC). Two criteria are shown: FSC between two half-maps reconstructed from even and odd frames (evaluated at 0.143) and FSC between the final 3D map and the fitted model (evaluated at 0.5). (D) Zoomed-in views of the final 3D density map from panel A, displayed at two contour levels. (E) Superimposition of the high-contour level map from panel D onto its flexibly fitted model.

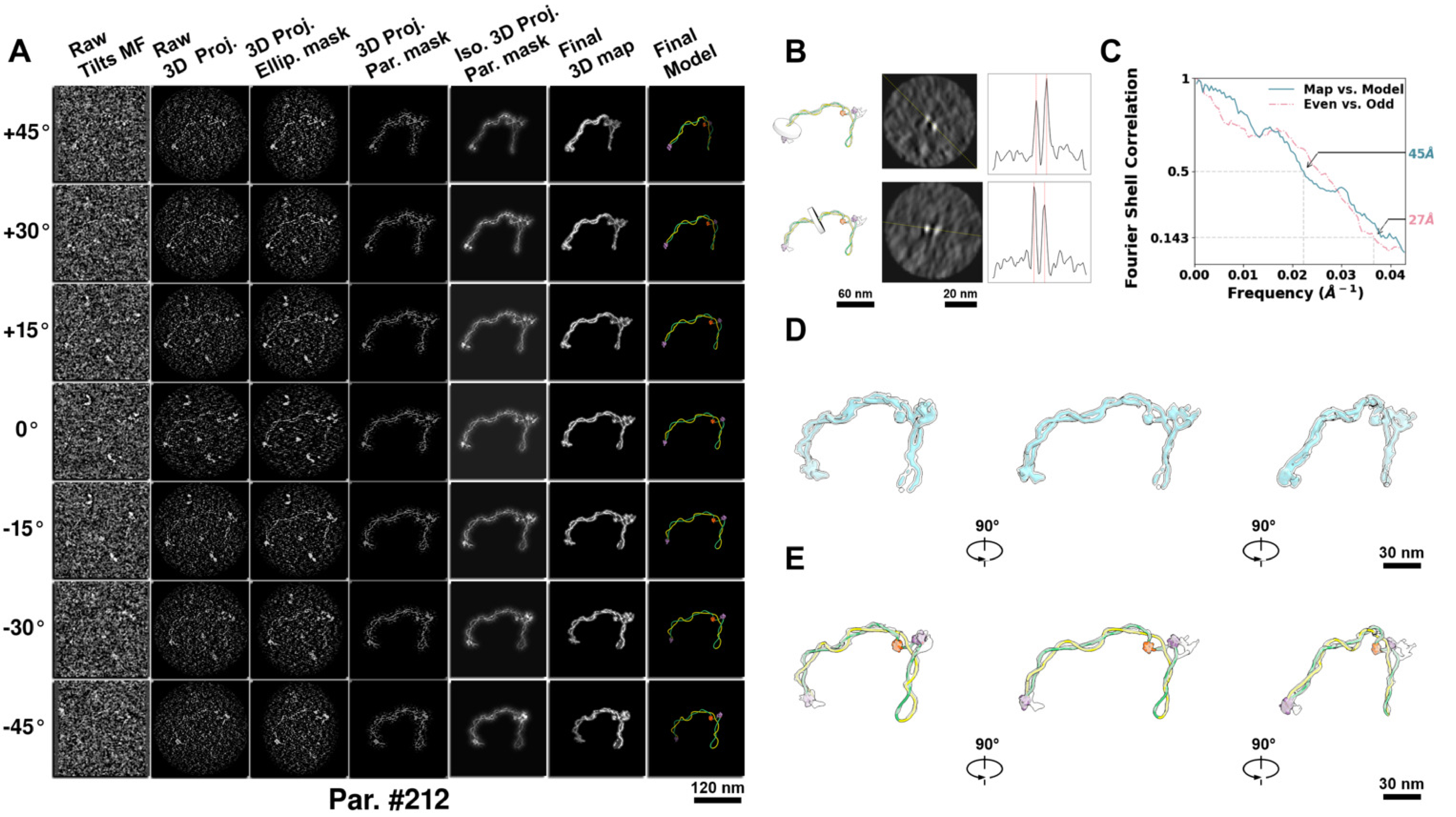

Supplementary Figure 212. Cryo-electron tomography (cryo-ET) 3D reconstruction of an individual plasmid particle in 40 mM K⁺, 5 mM Mg²⁺ in the presence of transcribed RNAP and dCas9.
(A) 3D reconstruction of the plasmid particle (index no. 212). The first column shows seven representative tilt images from –45° to +45° in step of 15°. The second, third, and fourth columns show 3D projections of the particle with spherical, ellipsoidal (thinner along the z-dimension), and particle-shaped masks, respectively. The fifth column displays the 3D projections of the enhanced and IsoNet missing-wedge-corrected particle. The sixth and seventh columns present the final 3D map and the flexibly fitted model, respectively. (B) Two random cross-sectional views (12 nm thickness) of the plasmid density map along its plectoneme axis are shown in the left-middle panel. The intensity profile along the line crossing the two high-density DNA spots is displayed in the right panel. (C) Resolution assessment of the final 3D map using Fourier shell correlation (FSC). Two criteria are shown: FSC between two half-maps reconstructed from even and odd frames (evaluated at 0.143) and FSC between the final 3D map and the fitted model (evaluated at 0.5). (D) Zoomed-in views of the final 3D density map from panel A, displayed at two contour levels. (E) Superimposition of the high-contour level map from panel D onto its flexibly fitted model.

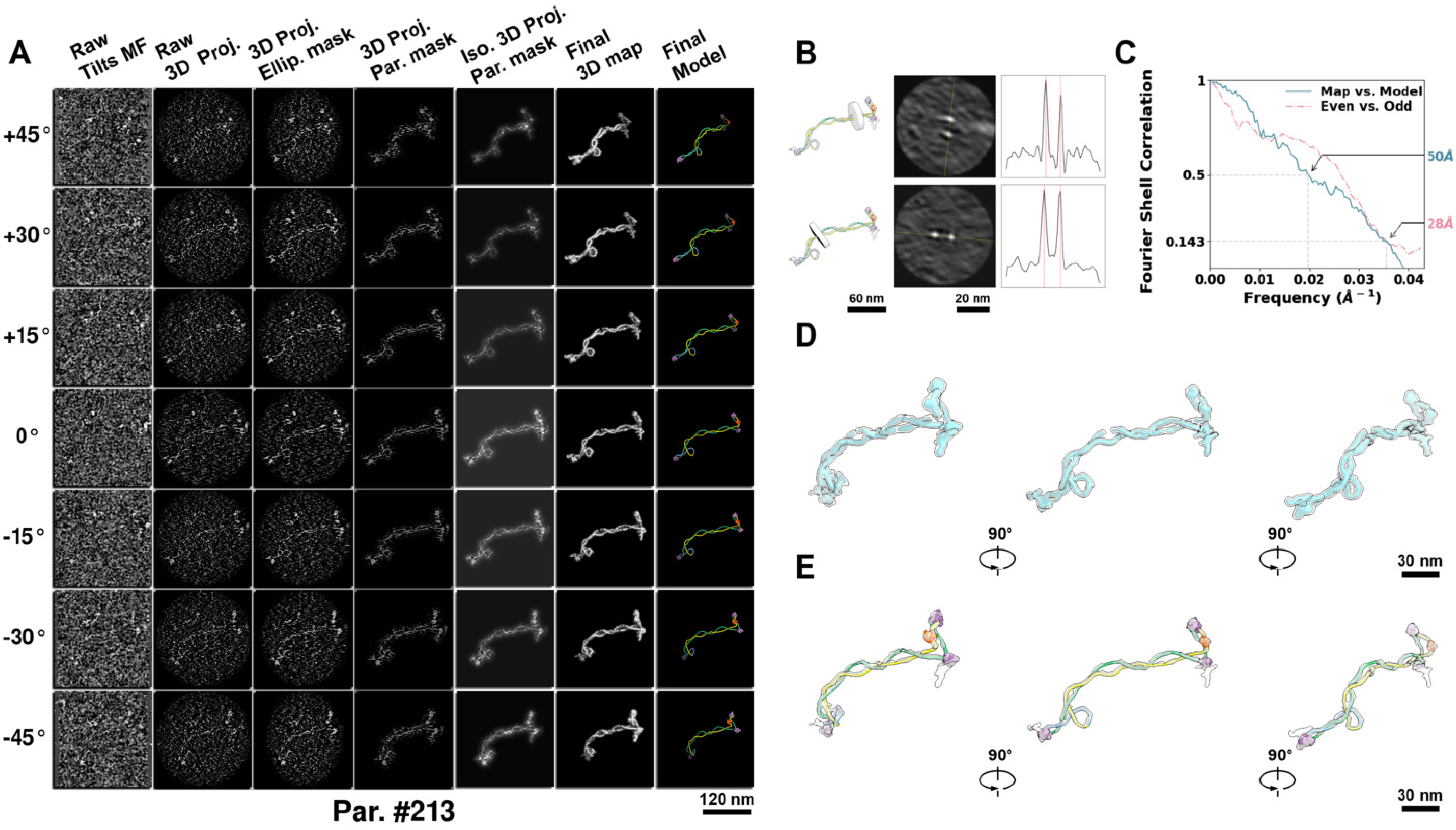

Supplementary Figure 213. Cryo-electron tomography (cryo-ET) 3D reconstruction of an individual plasmid particle in 40 mM K^+, 5 mM Mg^{2+} in the presence of transcribed RNAP and dCas9.
(A) 3D reconstruction of the plasmid particle (index no. 213). The first column shows seven representative tilt images from –45° to +45° in step of 15°. The second, third, and fourth columns show 3D projections of the particle with spherical, ellipsoidal (thinner along the z-dimension), and particle-shaped masks, respectively. The fifth column displays the 3D projections of the enhanced and IsoNet missing-wedge-corrected particle. The sixth and seventh columns present the final 3D map and the flexibly fitted model, respectively. (B) Two random cross-sectional views (12 nm thickness) of the plasmid density map along its plectoneme axis are shown in the left-middle panel. The intensity profile along the line crossing the two high-density DNA spots is displayed in the right panel. (C) Resolution assessment of the final 3D map using Fourier shell correlation (FSC). Two criteria are shown: FSC between two half-maps reconstructed from even and odd frames (evaluated at 0.143) and FSC between the final 3D map and the fitted model (evaluated at 0.5). (D) Zoomed-in views of the final 3D density map from panel A, displayed at two contour levels. (E) Superimposition of the high-contour level map from panel D onto its flexibly fitted model.

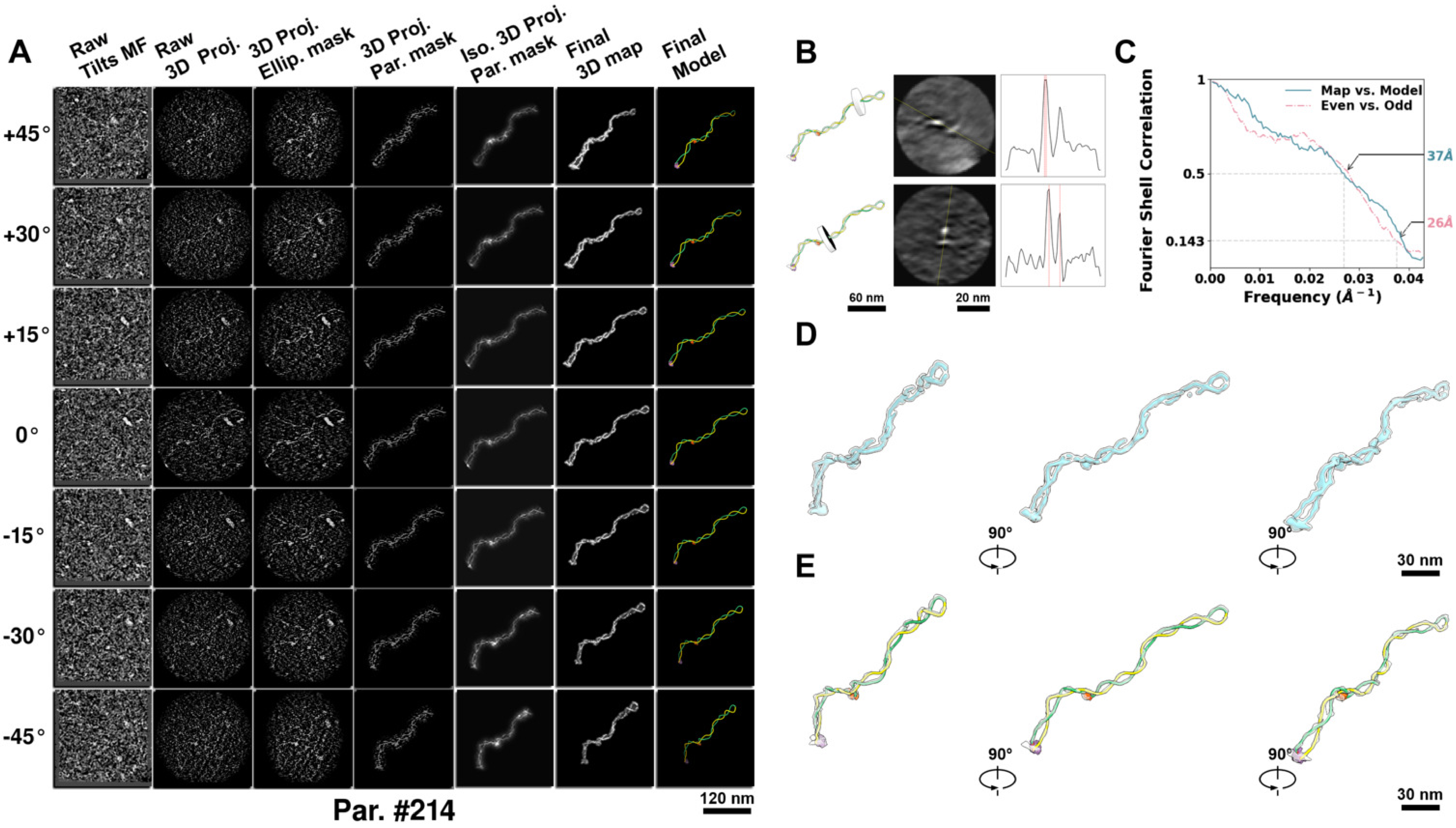

Supplementary Figure 214. Cryo-electron tomography (cryo-ET) 3D reconstruction of an individual plasmid particle in 40 mM K^+, 5 mM Mg^{2+} in the presence of transcribed RNAP and dCas9.
(A) 3D reconstruction of the plasmid particle (index no. 214). The first column shows seven representative tilt images from –45° to +45° in step of 15°. The second, third, and fourth columns show 3D projections of the particle with spherical, ellipsoidal (thinner along the z-dimension), and particle-shaped masks, respectively. The fifth column displays the 3D projections of the enhanced and IsoNet missing-wedge-corrected particle. The sixth and seventh columns present the final 3D map and the flexibly fitted model, respectively. (B) Two random cross-sectional views (12 nm thickness) of the plasmid density map along its plectoneme axis are shown in the left-middle panel. The intensity profile along the line crossing the two high-density DNA spots is displayed in the right panel. (C) Resolution assessment of the final 3D map using Fourier shell correlation (FSC). Two criteria are shown: FSC between two half-maps reconstructed from even and odd frames (evaluated at 0.143) and FSC between the final 3D map and the fitted model (evaluated at 0.5). (D) Zoomed-in views of the final 3D density map from panel A, displayed at two contour levels. (E) Superimposition of the high-contour level map from panel D onto its flexibly fitted model.

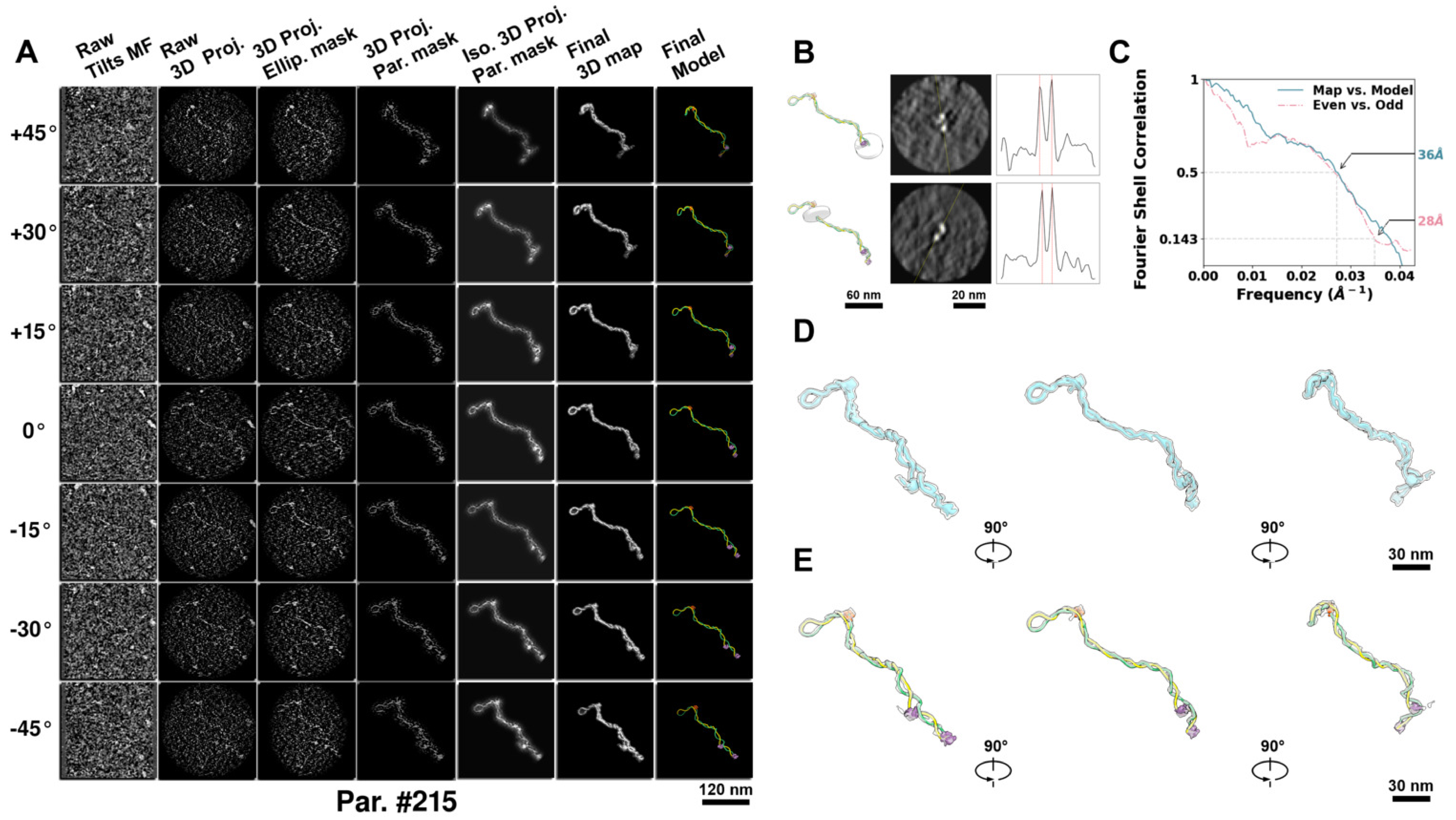

Supplementary Figure 215. Cryo-electron tomography (cryo-ET) 3D reconstruction of an individual plasmid particle in 40 mM K⁺, 5 mM Mg^{2+} in the presence of transcribed RNAP and dCas9.
(A) 3D reconstruction of the plasmid particle (index no. 215). The first column shows seven representative tilt images from –45° to +45° in step of 15°. The second, third, and fourth columns show 3D projections of the particle with spherical, ellipsoidal (thinner along the z-dimension), and particle-shaped masks, respectively. The fifth column displays the 3D projections of the enhanced and IsoNet missing-wedge-corrected particle. The sixth and seventh columns present the final 3D map and the flexibly fitted model, respectively. (B) Two random cross-sectional views (12 nm thickness) of the plasmid density map along its plectoneme axis are shown in the left-middle panel. The intensity profile along the line crossing the two high-density DNA spots is displayed in the right panel. (C) Resolution assessment of the final 3D map using Fourier shell correlation (FSC). Two criteria are shown: FSC between two half-maps reconstructed from even and odd frames (evaluated at 0.143) and FSC between the final 3D map and the fitted model (evaluated at 0.5). (D) Zoomed-in views of the final 3D density map from panel A, displayed at two contour levels. (E) Superimposition of the high-contour level map from panel D onto its flexibly fitted model.

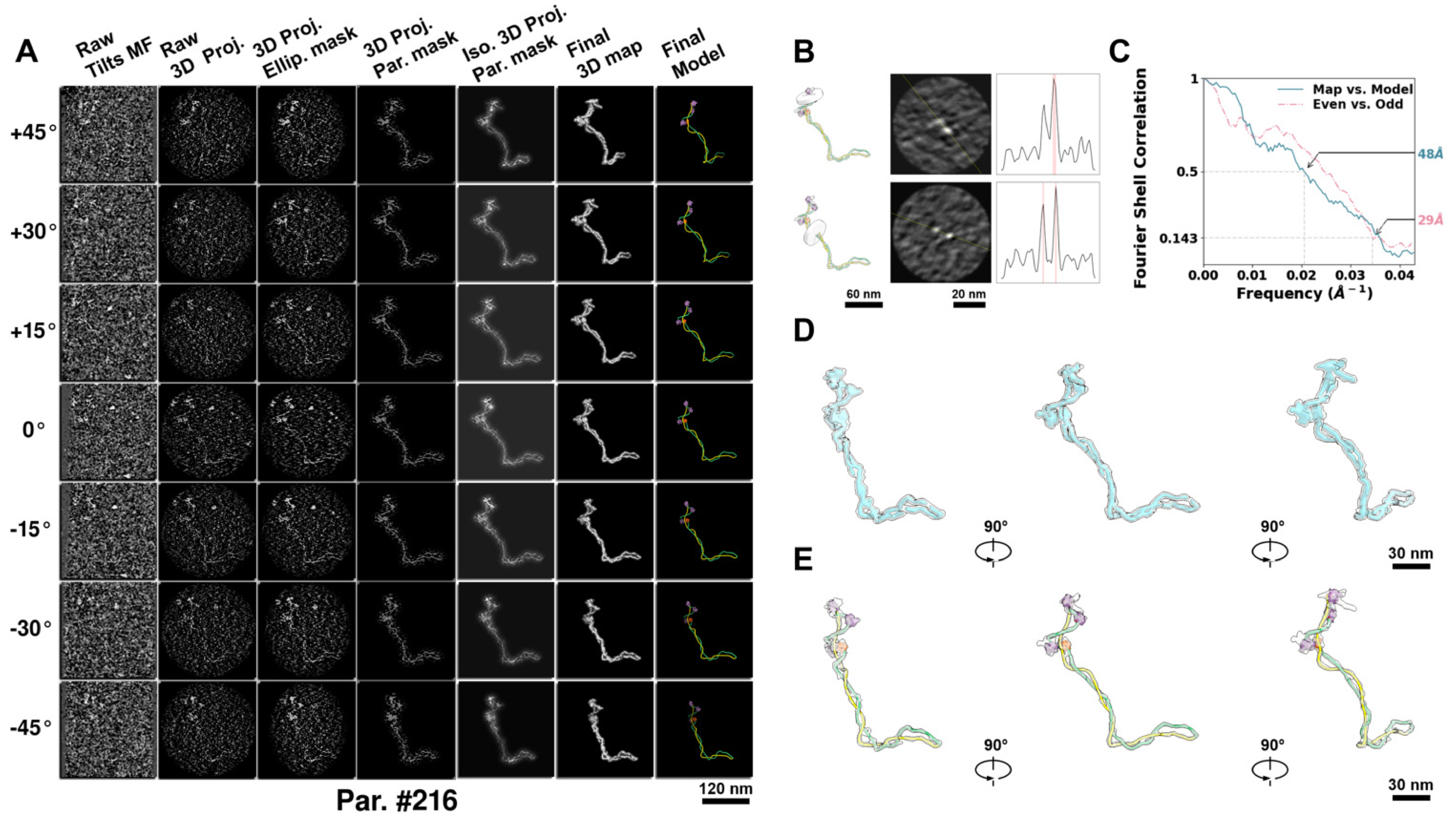

Supplementary Figure 216. Cryo-electron tomography (cryo-ET) 3D reconstruction of an individual plasmid particle in 40 mM K⁺, 5 mM Mg^{2+} in the presence of transcribed RNAP and dCas9.
(A) 3D reconstruction of the plasmid particle (index no. 216). The first column shows seven representative tilt images from –45° to +45° in step of 15°. The second, third, and fourth columns show 3D projections of the particle with spherical, ellipsoidal (thinner along the z-dimension), and particle-shaped masks, respectively. The fifth column displays the 3D projections of the enhanced and IsoNet missing-wedge-corrected particle. The sixth and seventh columns present the final 3D map and the flexibly fitted model, respectively. (B) Two random cross-sectional views (12 nm thickness) of the plasmid density map along its plectoneme axis are shown in the left-middle panel. The intensity profile along the line crossing the two high-density DNA spots is displayed in the right panel. (C) Resolution assessment of the final 3D map using Fourier shell correlation (FSC). Two criteria are shown: FSC between two half-maps reconstructed from even and odd frames (evaluated at 0.143) and FSC between the final 3D map and the fitted model (evaluated at 0.5). (D) Zoomed-in views of the final 3D density map from panel A, displayed at two contour levels. (E) Superimposition of the high-contour level map from panel D onto its flexibly fitted model.

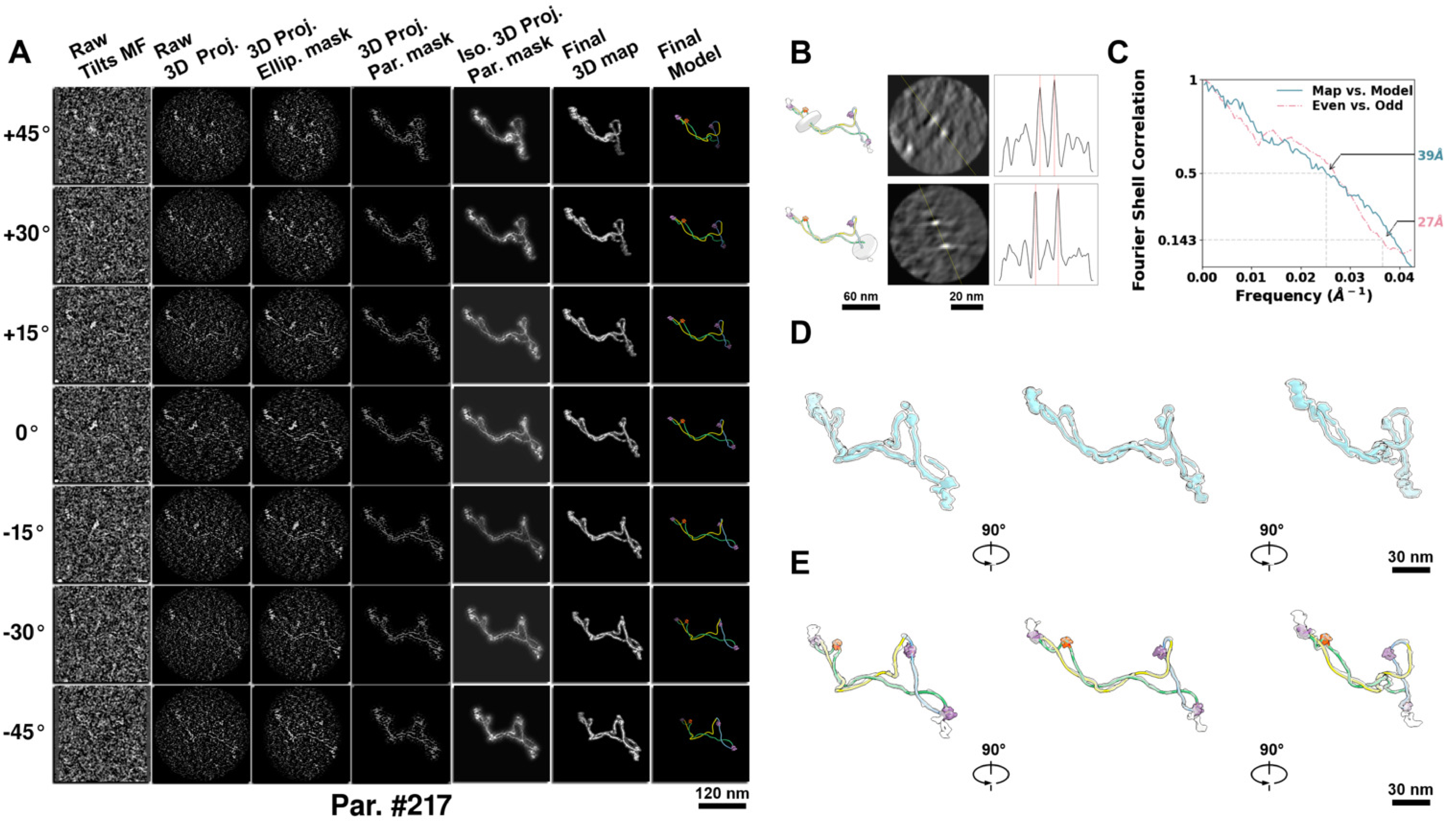

Supplementary Figure 217. Cryo-electron tomography (cryo-ET) 3D reconstruction of an individual plasmid particle in 40 mM K^+, 5 mM Mg^{2+} in the presence of transcribed RNAP and dCas9.
(A) 3D reconstruction of the plasmid particle (index no. 217). The first column shows seven representative tilt images from –45° to +45° in step of 15°. The second, third, and fourth columns show 3D projections of the particle with spherical, ellipsoidal (thinner along the z-dimension), and particle-shaped masks, respectively. The fifth column displays the 3D projections of the enhanced and IsoNet missing-wedge-corrected particle. The sixth and seventh columns present the final 3D map and the flexibly fitted model, respectively. (B) Two random cross-sectional views (12 nm thickness) of the plasmid density map along its plectoneme axis are shown in the left-middle panel. The intensity profile along the line crossing the two high-density DNA spots is displayed in the right panel. (C) Resolution assessment of the final 3D map using Fourier shell correlation (FSC). Two criteria are shown: FSC between two half-maps reconstructed from even and odd frames (evaluated at 0.143) and FSC between the final 3D map and the fitted model (evaluated at 0.5). (D) Zoomed-in views of the final 3D density map from panel A, displayed at two contour levels. (E) Superimposition of the high-contour level map from panel D onto its flexibly fitted model.

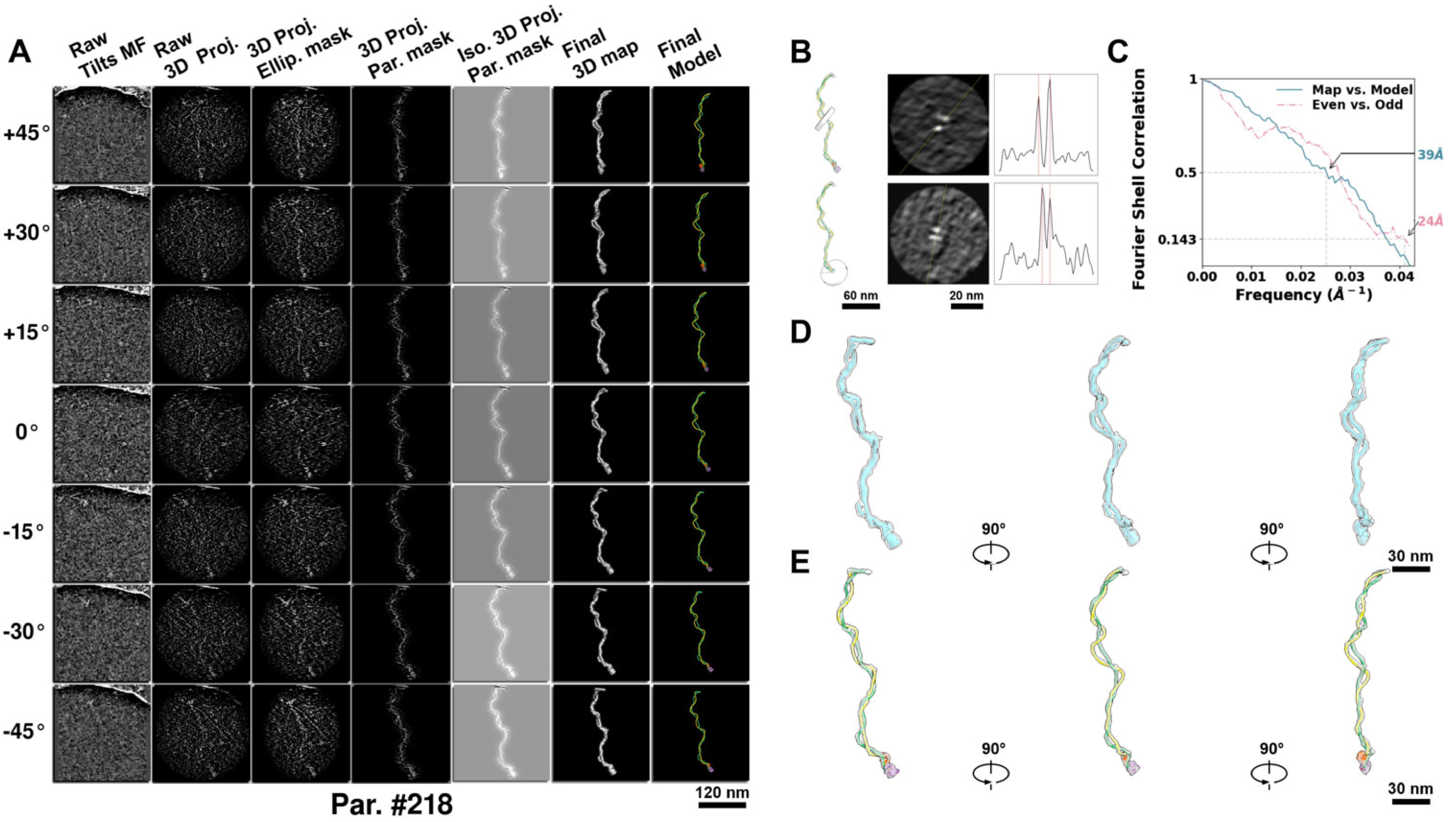

Supplementary Figure 218. Cryo-electron tomography (cryo-ET) 3D reconstruction of an individual plasmid particle in 40 mM K^+, 5 mM Mg^{2+} in the presence of transcribed RNAP and dCas9.
(A) 3D reconstruction of the plasmid particle (index no. 218). The first column shows seven representative tilt images from –45° to +45° in step of 15°. The second, third, and fourth columns show 3D projections of the particle with spherical, ellipsoidal (thinner along the z-dimension), and particle-shaped masks, respectively. The fifth column displays the 3D projections of the enhanced and IsoNet missing-wedge-corrected particle. The sixth and seventh columns present the final 3D map and the flexibly fitted model, respectively. (B) Two random cross-sectional views (12 nm thickness) of the plasmid density map along its plectoneme axis are shown in the left-middle panel. The intensity profile along the line crossing the two high-density DNA spots is displayed in the right panel. (C) Resolution assessment of the final 3D map using Fourier shell correlation (FSC). Two criteria are shown: FSC between two half-maps reconstructed from even and odd frames (evaluated at 0.143) and FSC between the final 3D map and the fitted model (evaluated at 0.5). (D) Zoomed-in views of the final 3D density map from panel A, displayed at two contour levels. (E) Superimposition of the high-contour level map from panel D onto its flexibly fitted model.

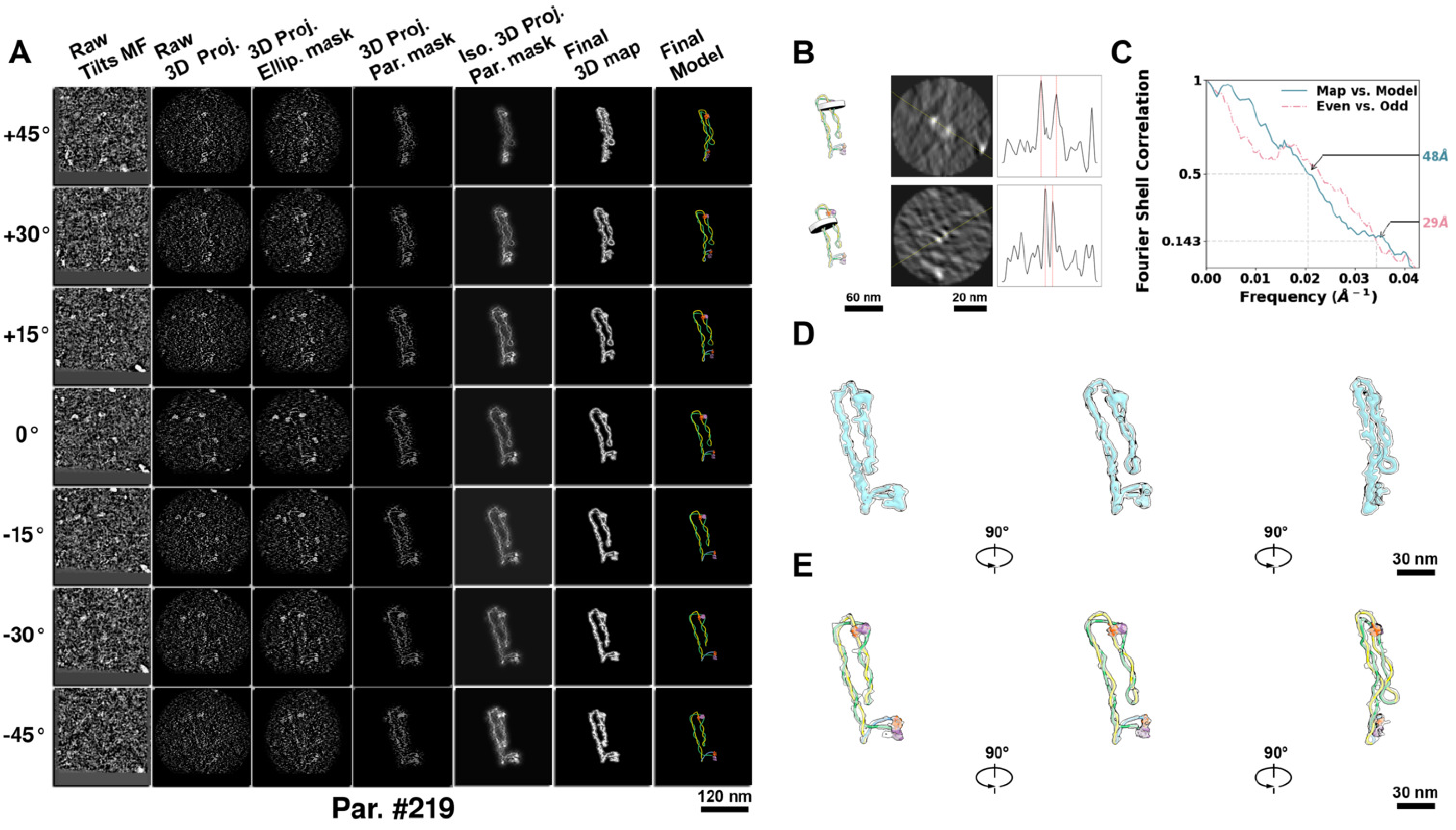

Supplementary Figure 219. Cryo-electron tomography (cryo-ET) 3D reconstruction of an individual plasmid particle in 40 mM K⁺, 5 mM Mg²⁺ in the presence of transcribed RNAP and dCas9.
(A) 3D reconstruction of the plasmid particle (index no. 219). The first column shows seven representative tilt images from –45° to +45° in step of 15°. The second, third, and fourth columns show 3D projections of the particle with spherical, ellipsoidal (thinner along the z-dimension), and particle-shaped masks, respectively. The fifth column displays the 3D projections of the enhanced and IsoNet missing-wedge-corrected particle. The sixth and seventh columns present the final 3D map and the flexibly fitted model, respectively. (B) Two random cross-sectional views (12 nm thickness) of the plasmid density map along its plectoneme axis are shown in the left-middle panel. The intensity profile along the line crossing the two high-density DNA spots is displayed in the right panel. (C) Resolution assessment of the final 3D map using Fourier shell correlation (FSC). Two criteria are shown: FSC between two half-maps reconstructed from even and odd frames (evaluated at 0.143) and FSC between the final 3D map and the fitted model (evaluated at 0.5). (D) Zoomed-in views of the final 3D density map from panel A, displayed at two contour levels. (E) Superimposition of the high-contour level map from panel D onto its flexibly fitted model.

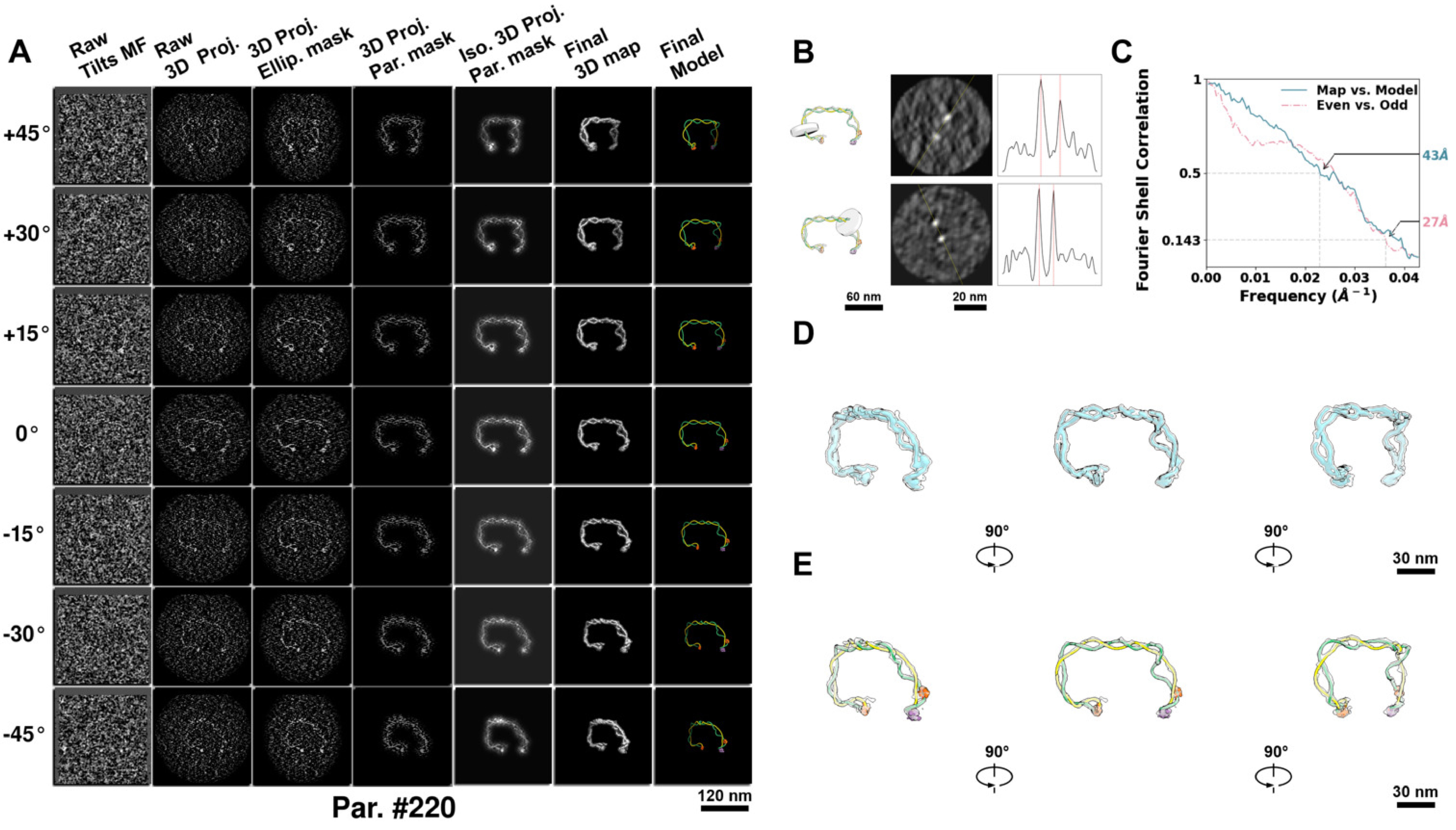

Supplementary Figure 220. Cryo-electron tomography (cryo-ET) 3D reconstruction of an individual plasmid particle in 40 mM K⁺, 5 mM Mg²⁺ in the presence of transcribed RNAP and dCas9.
(A) 3D reconstruction of the plasmid particle (index no. 220). The first column shows seven representative tilt images from –45° to +45° in step of 15°. The second, third, and fourth columns show 3D projections of the particle with spherical, ellipsoidal (thinner along the z-dimension), and particle-shaped masks, respectively. The fifth column displays the 3D projections of the enhanced and IsoNet missing-wedge-corrected particle. The sixth and seventh columns present the final 3D map and the flexibly fitted model, respectively. (B) Two random cross-sectional views (12 nm thickness) of the plasmid density map along its plectoneme axis are shown in the left-middle panel. The intensity profile along the line crossing the two high-density DNA spots is displayed in the right panel. (C) Resolution assessment of the final 3D map using Fourier shell correlation (FSC). Two criteria are shown: FSC between two half-maps reconstructed from even and odd frames (evaluated at 0.143) and FSC between the final 3D map and the fitted model (evaluated at 0.5). (D) Zoomed-in views of the final 3D density map from panel A, displayed at two contour levels. (E) Superimposition of the high-contour level map from panel D onto its flexibly fitted model.

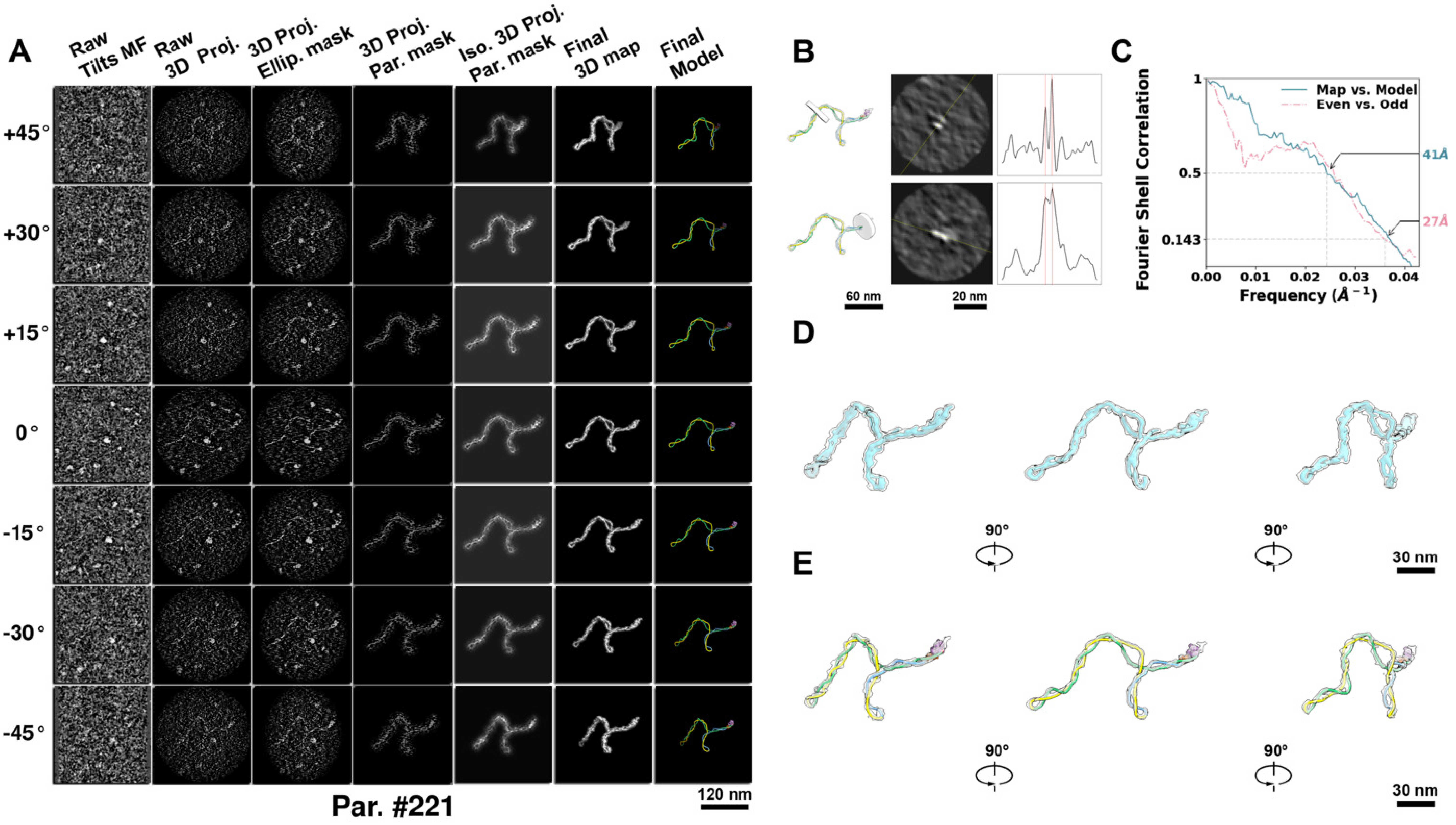

Supplementary Figure 221. Cryo-electron tomography (cryo-ET) 3D reconstruction of an individual plasmid particle in 40 mM K$^+$, 5 mM Mg^{2+} in the presence of transcribed RNAP and dCas9.
(A) 3D reconstruction of the plasmid particle (index no. 221). The first column shows seven representative tilt images from –45° to +45° in step of 15°. The second, third, and fourth columns show 3D projections of the particle with spherical, ellipsoidal (thinner along the z-dimension), and particle-shaped masks, respectively. The fifth column displays the 3D projections of the enhanced and IsoNet missing-wedge-corrected particle. The sixth and seventh columns present the final 3D map and the flexibly fitted model, respectively. (B) Two random cross-sectional views (12 nm thickness) of the plasmid density map along its plectoneme axis are shown in the left-middle panel. The intensity profile along the line crossing the two high-density DNA spots is displayed in the right panel. (C) Resolution assessment of the final 3D map using Fourier shell correlation (FSC). Two criteria are shown: FSC between two half-maps reconstructed from even and odd frames (evaluated at 0.143) and FSC between the final 3D map and the fitted model (evaluated at 0.5). (D) Zoomed-in views of the final 3D density map from panel A, displayed at two contour levels. (E) Superimposition of the high-contour level map from panel D onto its flexibly fitted model.

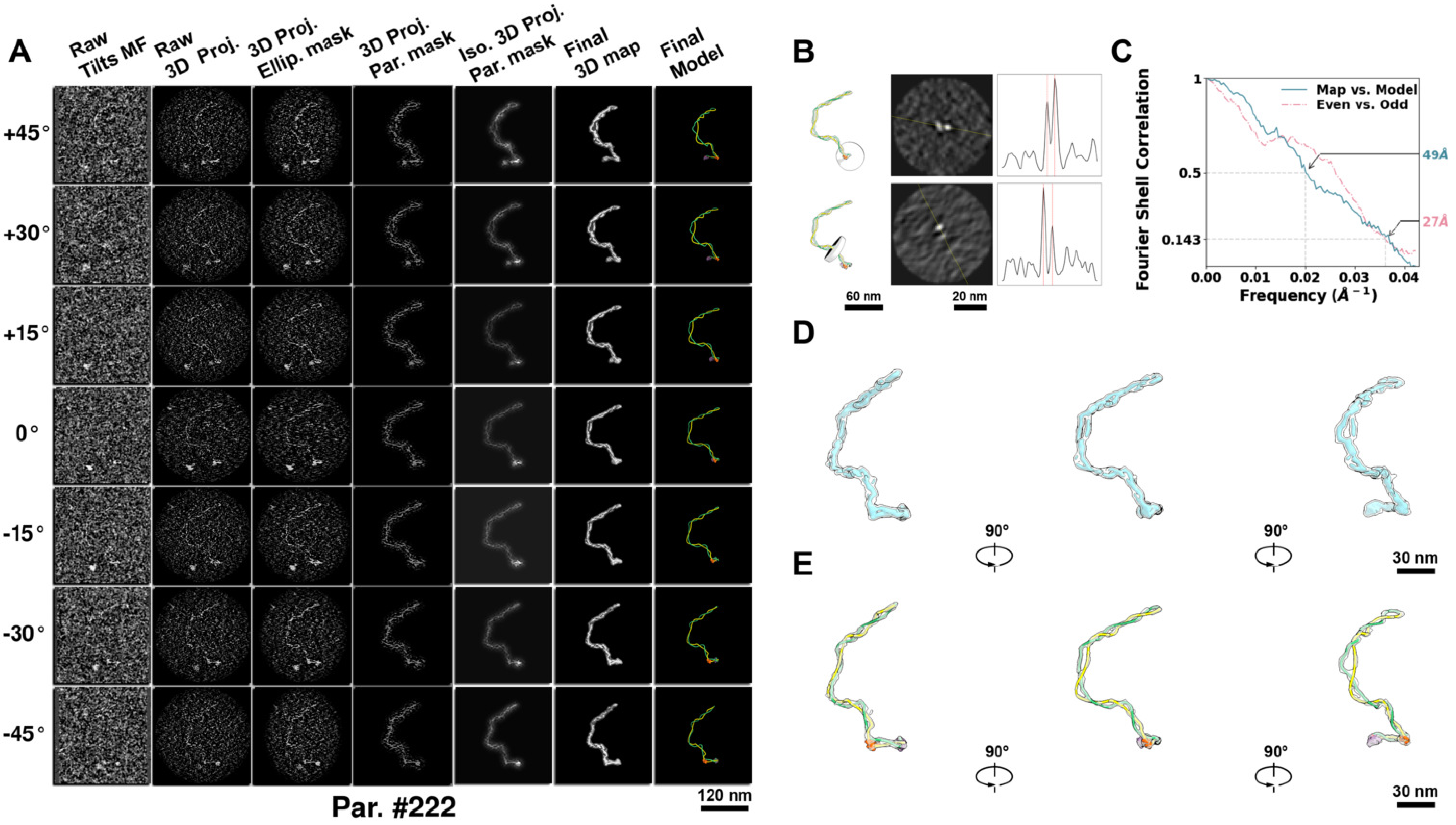

Supplementary Figure 222. Cryo-electron tomography (cryo-ET) 3D reconstruction of an individual plasmid particle in 40 mM K$^+$, 5 mM Mg^{2+} in the presence of transcribed RNAP and dCas9.
(A) 3D reconstruction of the plasmid particle (index no. 222). The first column shows seven representative tilt images from –45° to +45° in step of 15°. The second, third, and fourth columns show 3D projections of the particle with spherical, ellipsoidal (thinner along the z-dimension), and particle-shaped masks, respectively. The fifth column displays the 3D projections of the enhanced and IsoNet missing-wedge-corrected particle. The sixth and seventh columns present the final 3D map and the flexibly fitted model, respectively. (B) Two random cross-sectional views (12 nm thickness) of the plasmid density map along its plectoneme axis are shown in the left-middle panel. The intensity profile along the line crossing the two high-density DNA spots is displayed in the right panel. (C) Resolution assessment of the final 3D map using Fourier shell correlation (FSC). Two criteria are shown: FSC between two half-maps reconstructed from even and odd frames (evaluated at 0.143) and FSC between the final 3D map and the fitted model (evaluated at 0.5). (D) Zoomed-in views of the final 3D density map from panel A, displayed at two contour levels. (E) Superimposition of the high-contour level map from panel D onto its flexibly fitted model.

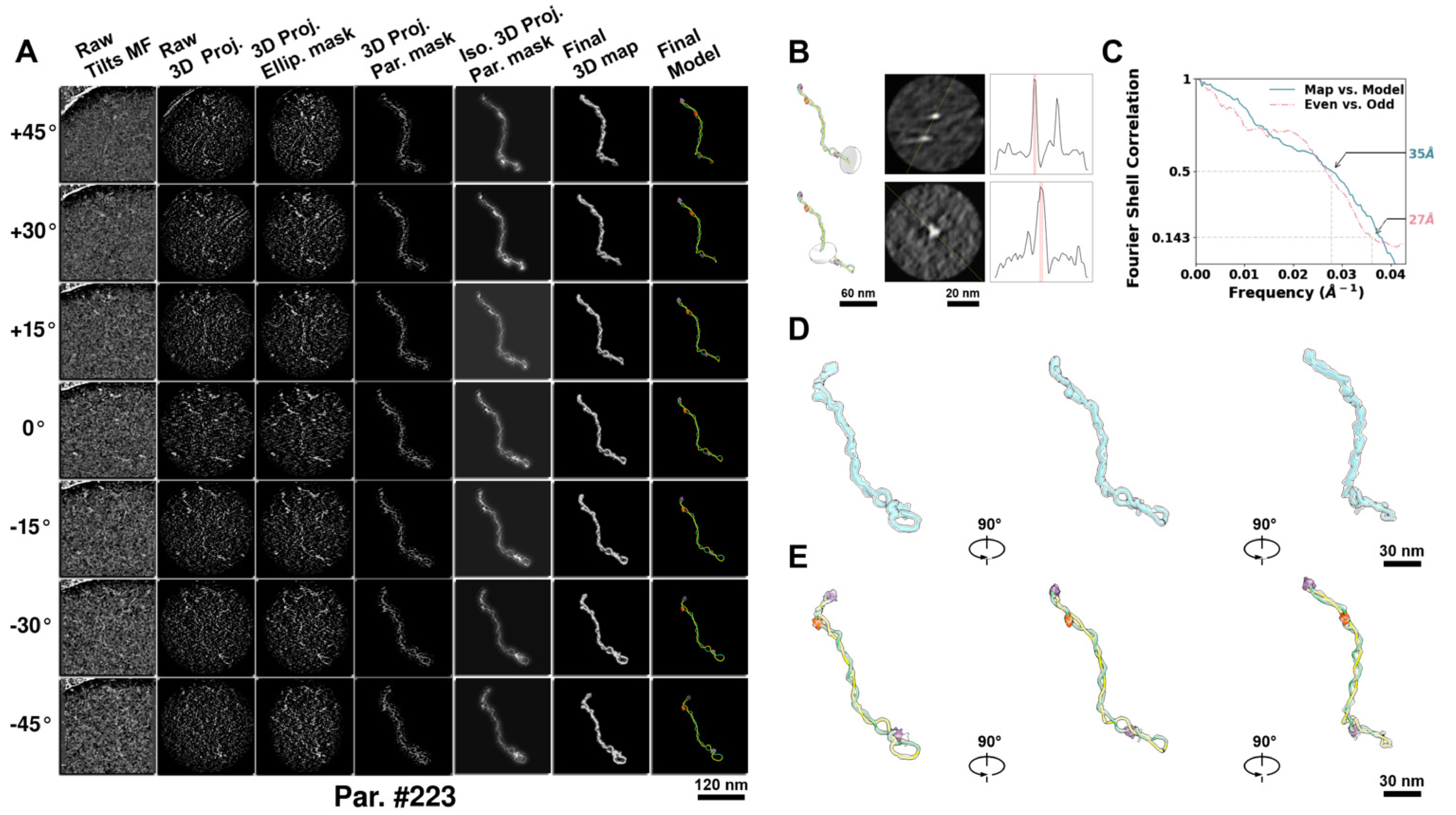

Supplementary Figure 223. Cryo-electron tomography (cryo-ET) 3D reconstruction of an individual plasmid particle in 40 mM K⁺, 5 mM Mg^{2+} in the presence of transcribed RNAP and dCas9.
(A) 3D reconstruction of the plasmid particle (index no. 223). The first column shows seven representative tilt images from –45° to +45° in step of 15°. The second, third, and fourth columns show 3D projections of the particle with spherical, ellipsoidal (thinner along the z-dimension), and particle-shaped masks, respectively. The fifth column displays the 3D projections of the enhanced and IsoNet missing-wedge-corrected particle. The sixth and seventh columns present the final 3D map and the flexibly fitted model, respectively. (B) Two random cross-sectional views (12 nm thickness) of the plasmid density map along its plectoneme axis are shown in the left-middle panel. The intensity profile along the line crossing the two high-density DNA spots is displayed in the right panel. (C) Resolution assessment of the final 3D map using Fourier shell correlation (FSC). Two criteria are shown: FSC between two half-maps reconstructed from even and odd frames (evaluated at 0.143) and FSC between the final 3D map and the fitted model (evaluated at 0.5). (D) Zoomed-in views of the final 3D density map from panel A, displayed at two contour levels. (E) Superimposition of the high-contour level map from panel D onto its flexibly fitted model.

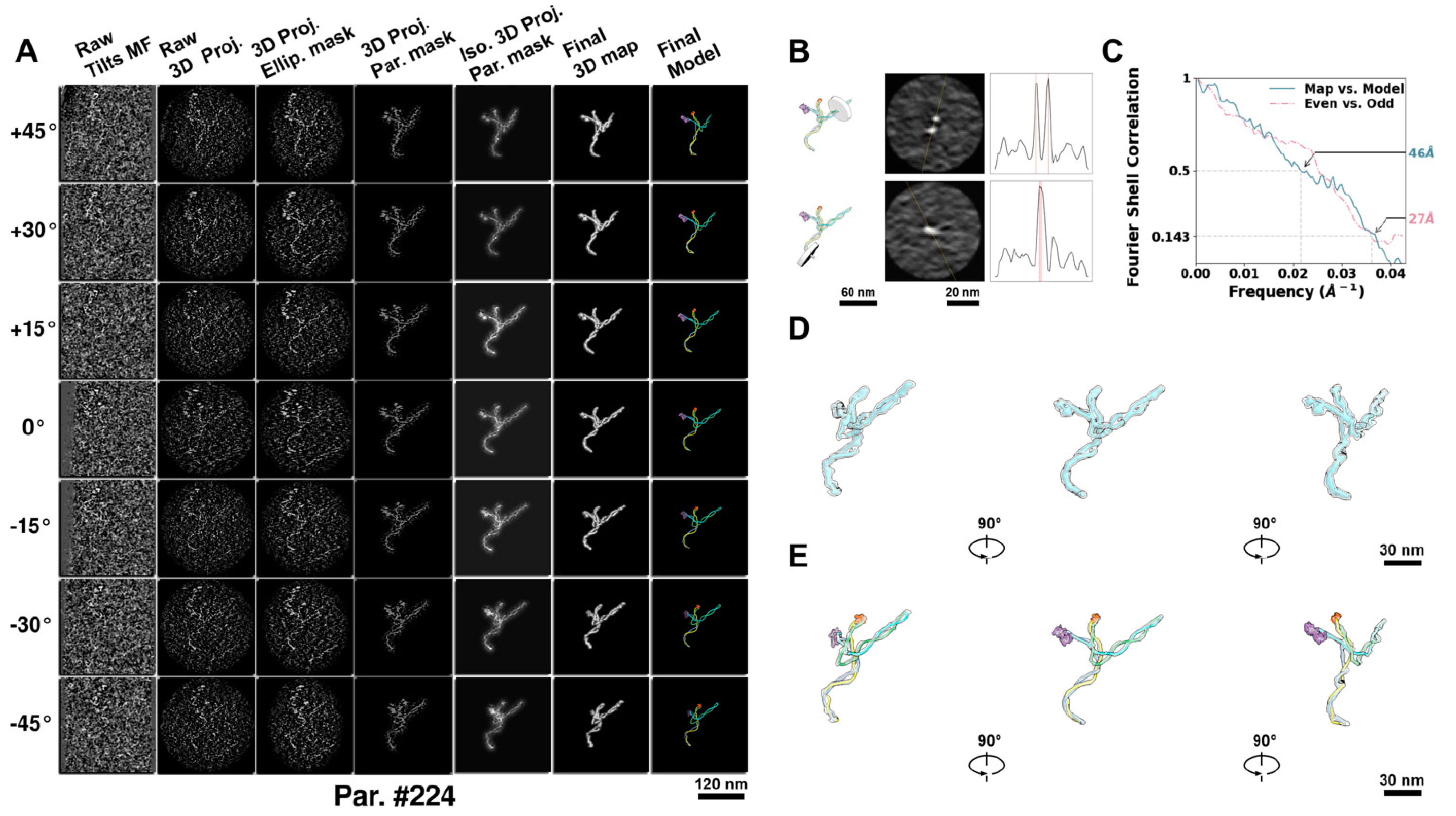

Supplementary Figure 224. Cryo-electron tomography (cryo-ET) 3D reconstruction of an individual plasmid particle in 40 mM K⁺, 5 mM Mg^{2+} in the presence of transcribed RNAP and dCas9.
(A) 3D reconstruction of the plasmid particle (index no. 224). The first column shows seven representative tilt images from –45° to +45° in step of 15°. The second, third, and fourth columns show 3D projections of the particle with spherical, ellipsoidal (thinner along the z-dimension), and particle-shaped masks, respectively. The fifth column displays the 3D projections of the enhanced and IsoNet missing-wedge-corrected particle. The sixth and seventh columns present the final 3D map and the flexibly fitted model, respectively. (B) Two random cross-sectional views (12 nm thickness) of the plasmid density map along its plectoneme axis are shown in the left-middle panel. The intensity profile along the line crossing the two high-density DNA spots is displayed in the right panel. (C) Resolution assessment of the final 3D map using Fourier shell correlation (FSC). Two criteria are shown: FSC between two half-maps reconstructed from even and odd frames (evaluated at 0.143) and FSC between the final 3D map and the fitted model (evaluated at 0.5). (D) Zoomed-in views of the final 3D density map from panel A, displayed at two contour levels. (E) Superimposition of the high-contour level map from panel D onto its flexibly fitted model.

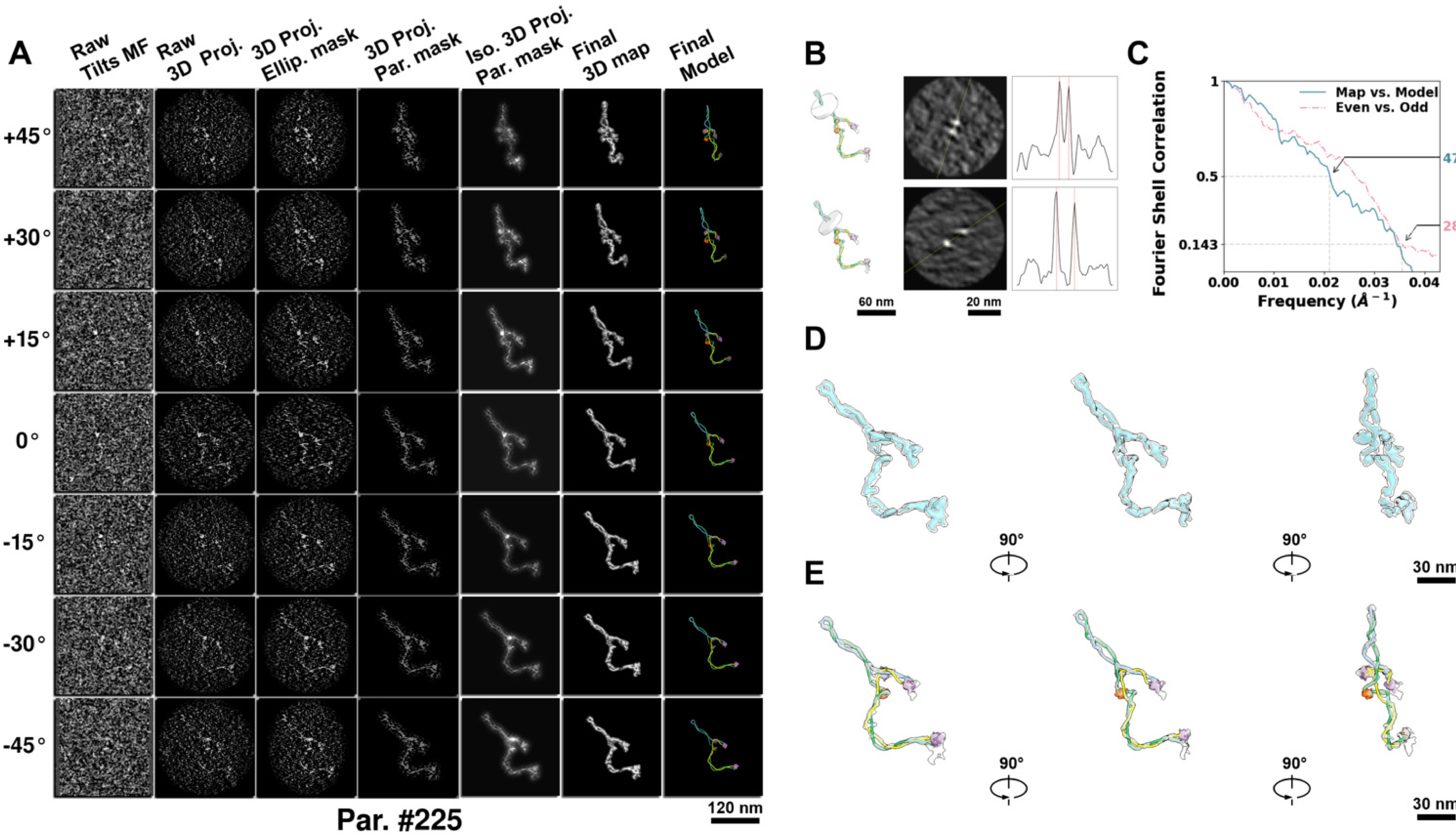

Supplementary Figure 225. Cryo-electron tomography (cryo-ET) 3D reconstruction of an individual plasmid particle in 40 mM K^+, 5 mM Mg^{2+} in the presence of transcribed RNAP and dCas9.
(A) 3D reconstruction of the plasmid particle (index no. 225). The first column shows seven representative tilt images from –45° to +45° in step of 15°. The second, third, and fourth columns show 3D projections of the particle with spherical, ellipsoidal (thinner along the z-dimension), and particle-shaped masks, respectively. The fifth column displays the 3D projections of the enhanced and IsoNet missing-wedge-corrected particle. The sixth and seventh columns present the final 3D map and the flexibly fitted model, respectively. (B) Two random cross-sectional views (12 nm thickness) of the plasmid density map along its plectoneme axis are shown in the left-middle panel. The intensity profile along the line crossing the two high-density DNA spots is displayed in the right panel. (C) Resolution assessment of the final 3D map using Fourier shell correlation (FSC). Two criteria are shown: FSC between two half-maps reconstructed from even and odd frames (evaluated at 0.143) and FSC between the final 3D map and the fitted model (evaluated at 0.5). (D) Zoomed-in views of the final 3D density map from panel A, displayed at two contour levels. (E) Superimposition of the high-contour level map from panel D onto its flexibly fitted model.

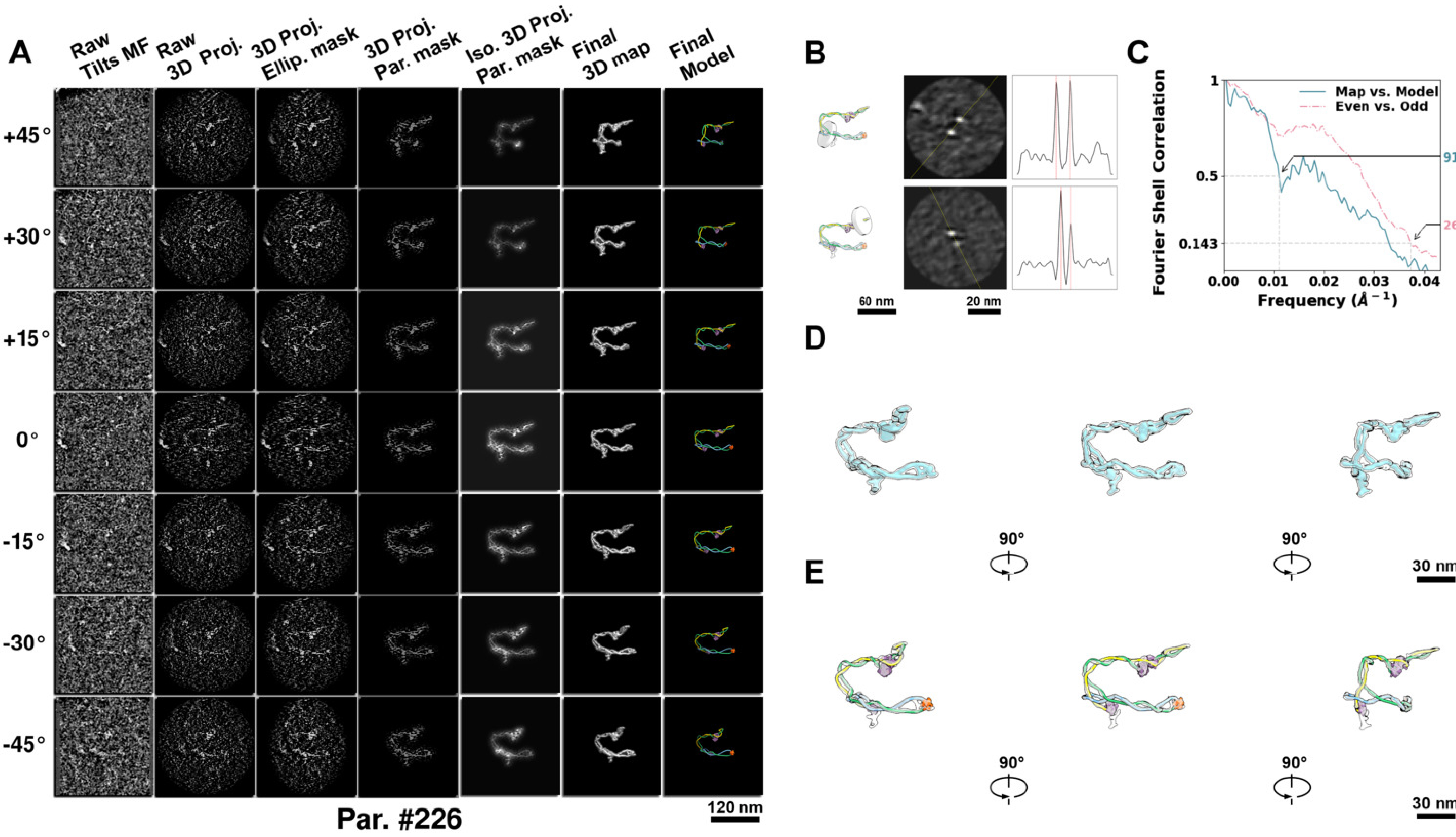

Supplementary Figure 226. Cryo-electron tomography (cryo-ET) 3D reconstruction of an individual plasmid particle in 40 mM K^+, 5 mM Mg^{2+} in the presence of transcribed RNAP and dCas9.
(A) 3D reconstruction of the plasmid particle (index no. 226). The first column shows seven representative tilt images from –45° to +45° in step of 15°. The second, third, and fourth columns show 3D projections of the particle with spherical, ellipsoidal (thinner along the z-dimension), and particle-shaped masks, respectively. The fifth column displays the 3D projections of the enhanced and IsoNet missing-wedge-corrected particle. The sixth and seventh columns present the final 3D map and the flexibly fitted model, respectively. (B) Two random cross-sectional views (12 nm thickness) of the plasmid density map along its plectoneme axis are shown in the left-middle panel. The intensity profile along the line crossing the two high-density DNA spots is displayed in the right panel. (C) Resolution assessment of the final 3D map using Fourier shell correlation (FSC). Two criteria are shown: FSC between two half-maps reconstructed from even and odd frames (evaluated at 0.143) and FSC between the final 3D map and the fitted model (evaluated at 0.5). (D) Zoomed-in views of the final 3D density map from panel A, displayed at two contour levels. (E) Superimposition of the high-contour level map from panel D onto its flexibly fitted model.

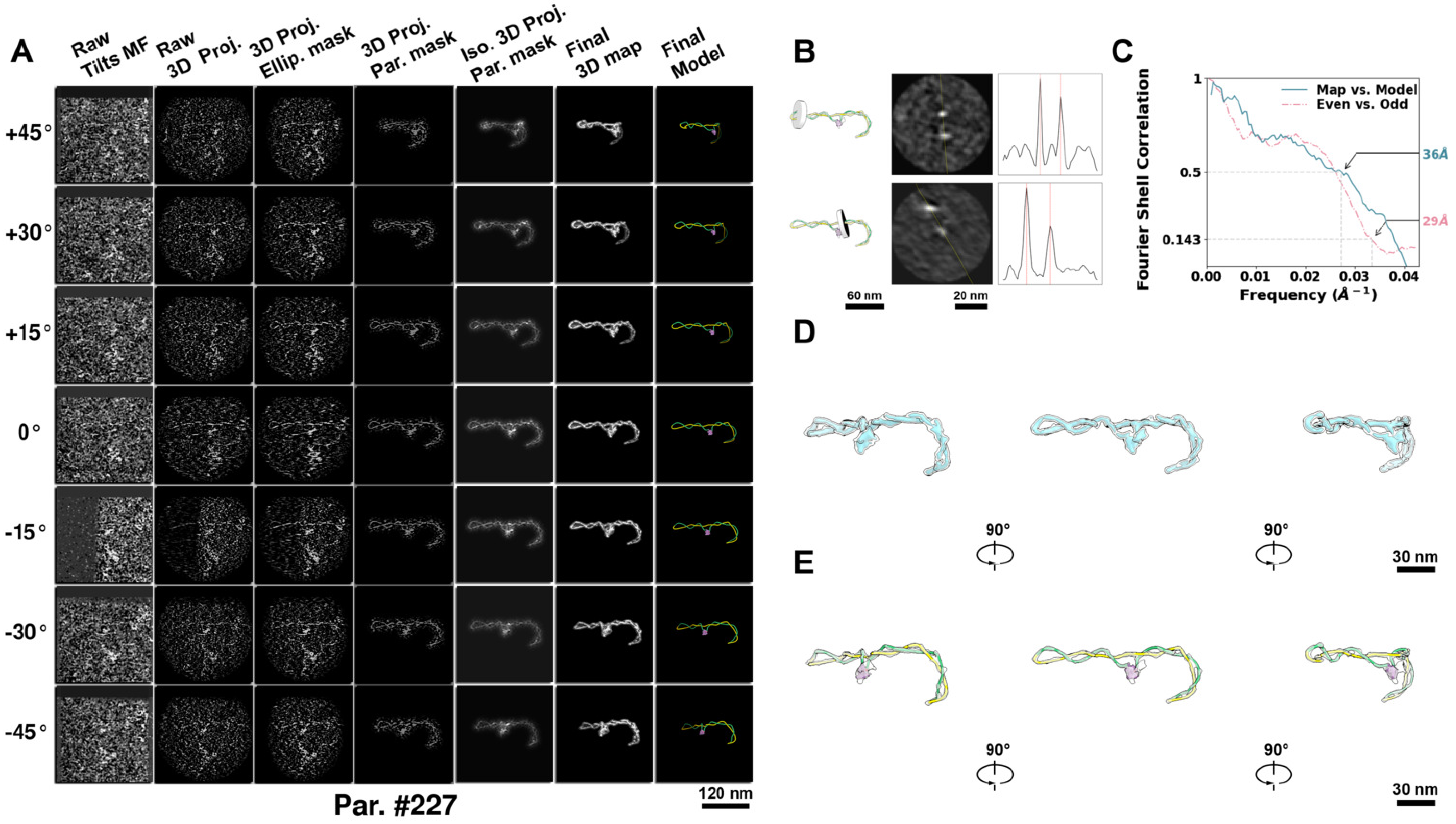

Supplementary Figure 227. Cryo-electron tomography (cryo-ET) 3D reconstruction of an individual plasmid particle in 40 mM K^{+}, 5 mM Mg^{2+} in the presence of transcribed RNAP and TopI.
(A) 3D reconstruction of the plasmid particle (index no. 227). The first column shows seven representative tilt images from –45° to +45° in step of 15°. The second, third, and fourth columns show 3D projections of the particle with spherical, ellipsoidal (thinner along the z-dimension), and particle-shaped masks, respectively. The fifth column displays the 3D projections of the enhanced and IsoNet missing-wedge-corrected particle. The sixth and seventh columns present the final 3D map and the flexibly fitted model, respectively. (B) Two random cross-sectional views (12 nm thickness) of the plasmid density map along its plectoneme axis are shown in the left-middle panel. The intensity profile along the line crossing the two high-density DNA spots is displayed in the right panel. (C) Resolution assessment of the final 3D map using Fourier shell correlation (FSC). Two criteria are shown: FSC between two half-maps reconstructed from even and odd frames (evaluated at 0.143) and FSC between the final 3D map and the fitted model (evaluated at 0.5). (D) Zoomed-in views of the final 3D density map from panel A, displayed at two contour levels. (E) Superimposition of the high-contour level map from panel D onto its flexibly fitted model.

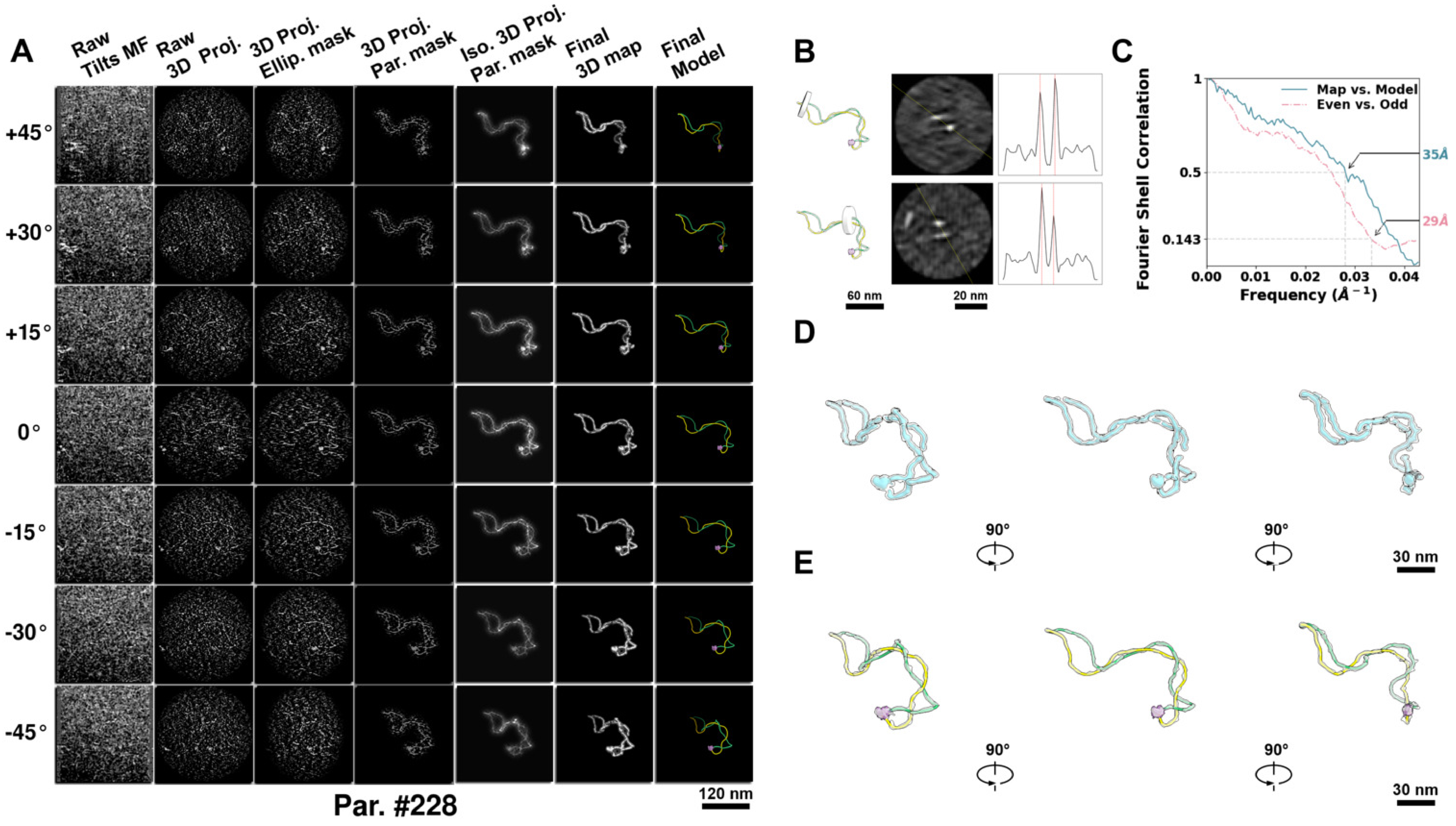

Supplementary Figure 228. Cryo-electron tomography (cryo-ET) 3D reconstruction of an individual plasmid particle in 40 mM K^{+}, 5 mM Mg^{2+} in the presence of transcribed RNAP and TopI.
(A) 3D reconstruction of the plasmid particle (index no. 228). The first column shows seven representative tilt images from –45° to +45° in step of 15°. The second, third, and fourth columns show 3D projections of the particle with spherical, ellipsoidal (thinner along the z-dimension), and particle-shaped masks, respectively. The fifth column displays the 3D projections of the enhanced and IsoNet missing-wedge-corrected particle. The sixth and seventh columns present the final 3D map and the flexibly fitted model, respectively. (B) Two random cross-sectional views (12 nm thickness) of the plasmid density map along its plectoneme axis are shown in the left-middle panel. The intensity profile along the line crossing the two high-density DNA spots is displayed in the right panel. (C) Resolution assessment of the final 3D map using Fourier shell correlation (FSC). Two criteria are shown: FSC between two half-maps reconstructed from even and odd frames (evaluated at 0.143) and FSC between the final 3D map and the fitted model (evaluated at 0.5). (D) Zoomed-in views of the final 3D density map from panel A, displayed at two contour levels. (E) Superimposition of the high-contour level map from panel D onto its flexibly fitted model.

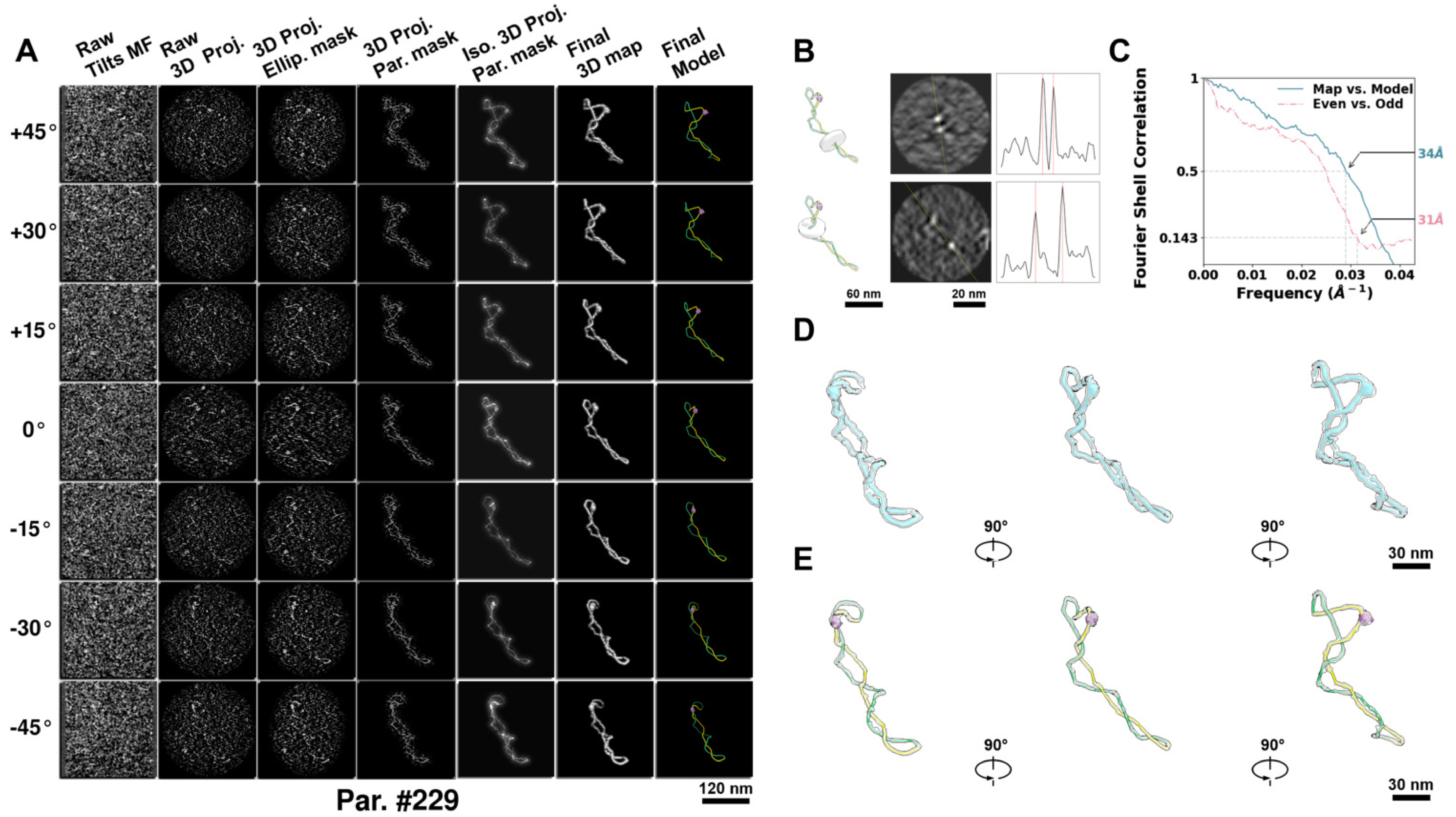

Supplementary Figure 229. Cryo-electron tomography (cryo-ET) 3D reconstruction of an individual plasmid particle in 40 mM K$^+$, 5 mM Mg^{2+} in the presence of transcribed RNAP and TopI.
(A) 3D reconstruction of the plasmid particle (index no. 229). The first column shows seven representative tilt images from –45° to +45° in step of 15°. The second, third, and fourth columns show 3D projections of the particle with spherical, ellipsoidal (thinner along the z-dimension), and particle-shaped masks, respectively. The fifth column displays the 3D projections of the enhanced and IsoNet missing-wedge-corrected particle. The sixth and seventh columns present the final 3D map and the flexibly fitted model, respectively. (B) Two random cross-sectional views (12 nm thickness) of the plasmid density map along its plectoneme axis are shown in the left-middle panel. The intensity profile along the line crossing the two high-density DNA spots is displayed in the right panel. (C) Resolution assessment of the final 3D map using Fourier shell correlation (FSC). Two criteria are shown: FSC between two half-maps reconstructed from even and odd frames (evaluated at 0.143) and FSC between the final 3D map and the fitted model (evaluated at 0.5). (D) Zoomed-in views of the final 3D density map from panel A, displayed at two contour levels. (E) Superimposition of the high-contour level map from panel D onto its flexibly fitted model.

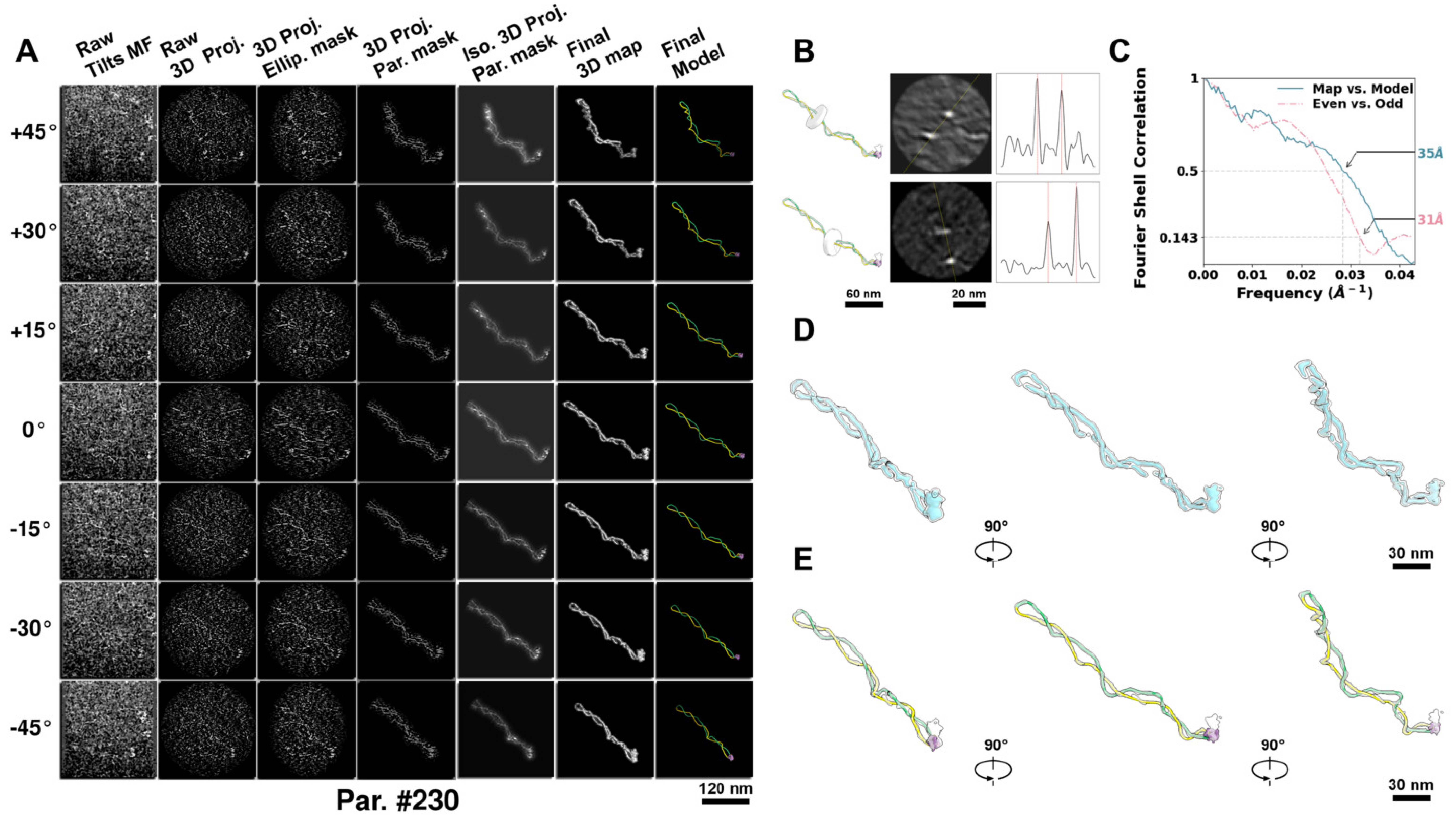

Supplementary Figure 230. Cryo-electron tomography (cryo-ET) 3D reconstruction of an individual plasmid particle in 40 mM K$^+$, 5 mM Mg^{2+} in the presence of transcribed RNAP and TopI.
(A) 3D reconstruction of the plasmid particle (index no. 230). The first column shows seven representative tilt images from –45° to +45° in step of 15°. The second, third, and fourth columns show 3D projections of the particle with spherical, ellipsoidal (thinner along the z-dimension), and particle-shaped masks, respectively. The fifth column displays the 3D projections of the enhanced and IsoNet missing-wedge-corrected particle. The sixth and seventh columns present the final 3D map and the flexibly fitted model, respectively. (B) Two random cross-sectional views (12 nm thickness) of the plasmid density map along its plectoneme axis are shown in the left-middle panel. The intensity profile along the line crossing the two high-density DNA spots is displayed in the right panel. (C) Resolution assessment of the final 3D map using Fourier shell correlation (FSC). Two criteria are shown: FSC between two half-maps reconstructed from even and odd frames (evaluated at 0.143) and FSC between the final 3D map and the fitted model (evaluated at 0.5). (D) Zoomed-in views of the final 3D density map from panel A, displayed at two contour levels. (E) Superimposition of the high-contour level map from panel D onto its flexibly fitted model.

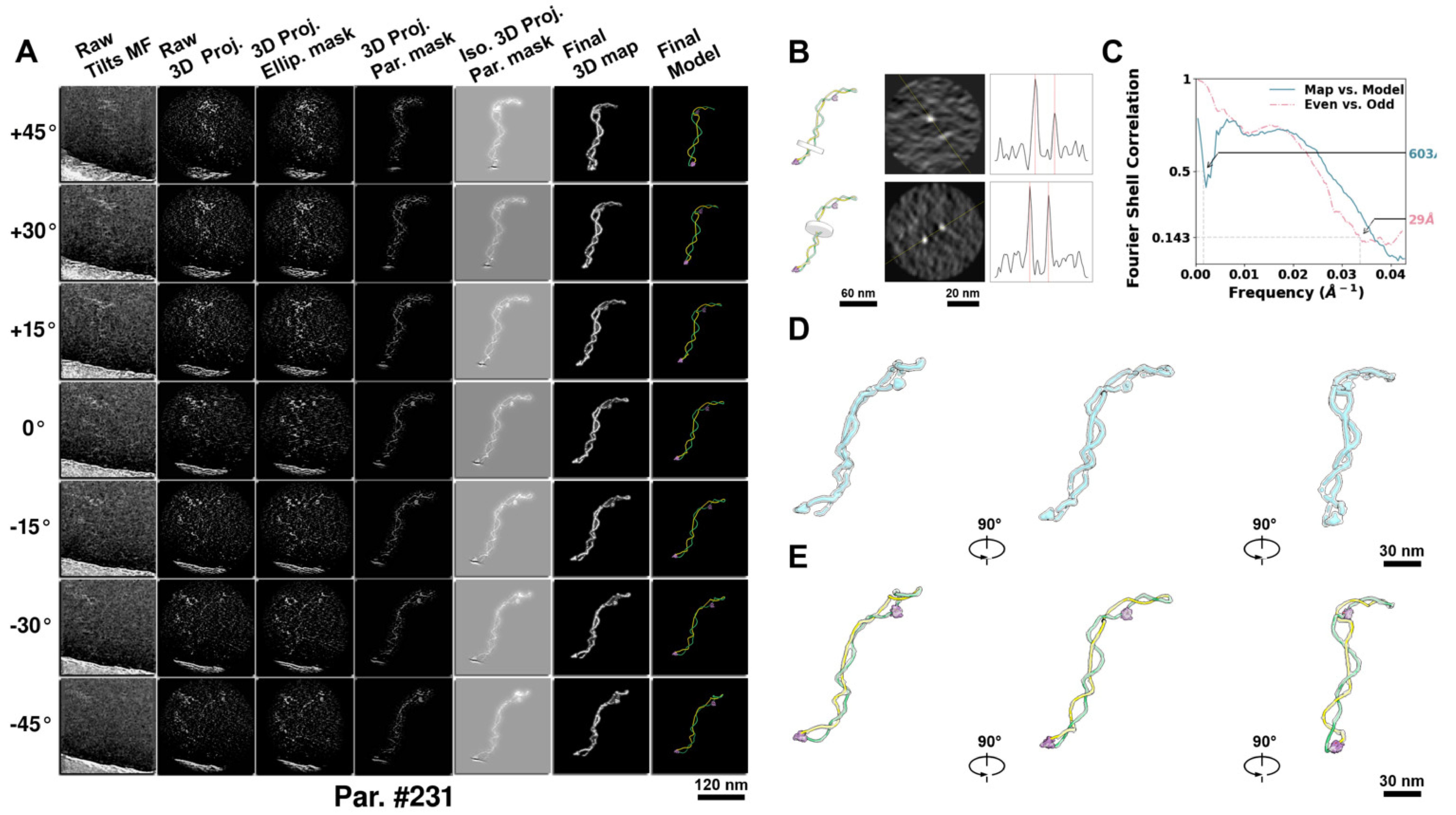

Supplementary Figure 231. Cryo-electron tomography (cryo-ET) 3D reconstruction of an individual plasmid particle in 40 mM K⁺, 5 mM Mg^{2+} in the presence of transcribed RNAP and TopI.
(A) 3D reconstruction of the plasmid particle (index no. 231). The first column shows seven representative tilt images from –45° to +45° in step of 15°. The second, third, and fourth columns show 3D projections of the particle with spherical, ellipsoidal (thinner along the z-dimension), and particle-shaped masks, respectively. The fifth column displays the 3D projections of the enhanced and IsoNet missing-wedge-corrected particle. The sixth and seventh columns present the final 3D map and the flexibly fitted model, respectively. (B) Two random cross-sectional views (12 nm thickness) of the plasmid density map along its plectoneme axis are shown in the left-middle panel. The intensity profile along the line crossing the two high-density DNA spots is displayed in the right panel. (C) Resolution assessment of the final 3D map using Fourier shell correlation (FSC). Two criteria are shown: FSC between two half-maps reconstructed from even and odd frames (evaluated at 0.143) and FSC between the final 3D map and the fitted model (evaluated at 0.5). (D) Zoomed-in views of the final 3D density map from panel A, displayed at two contour levels. (E) Superimposition of the high-contour level map from panel D onto its flexibly fitted model.

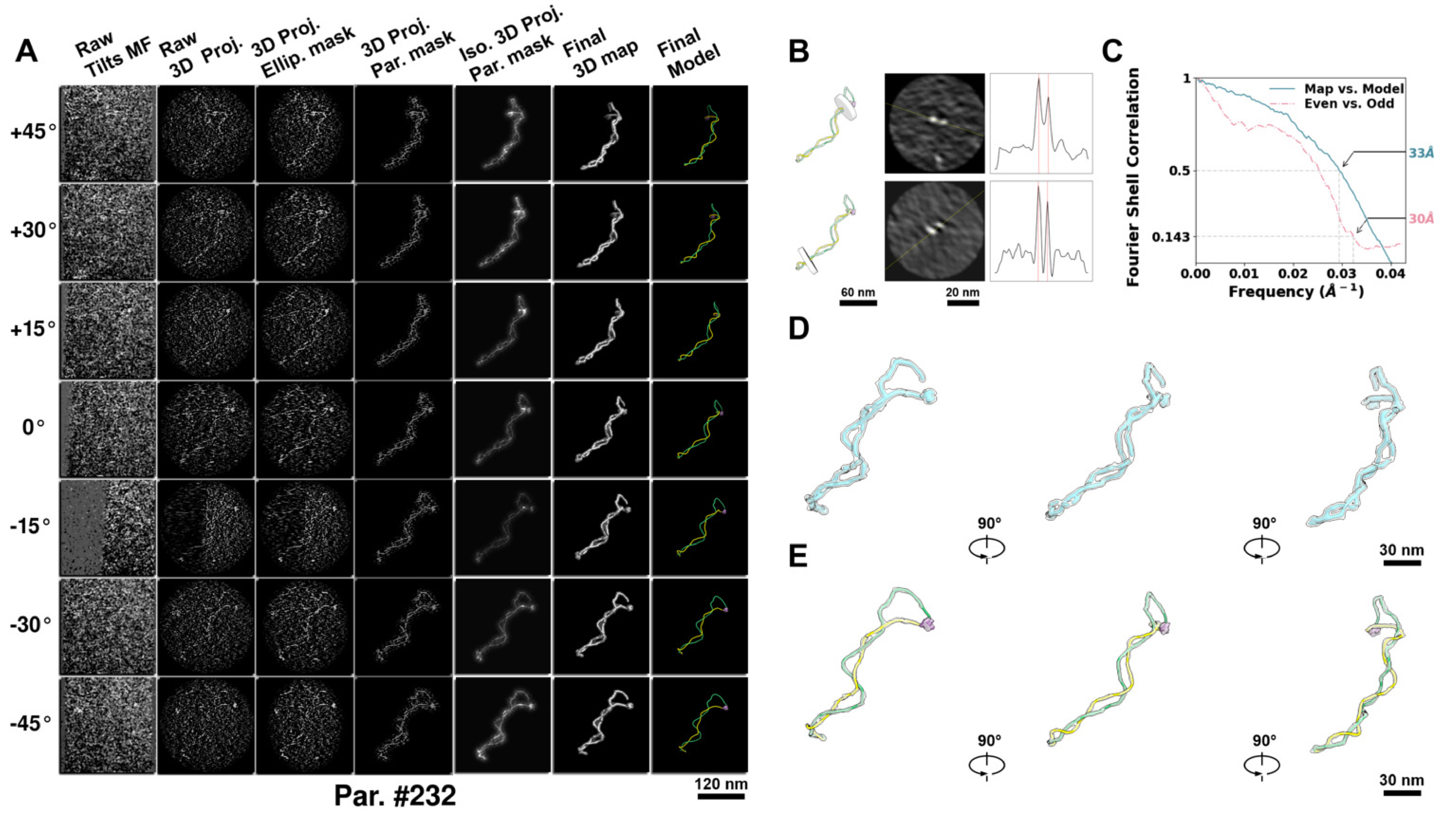

Supplementary Figure 232. Cryo-electron tomography (cryo-ET) 3D reconstruction of an individual plasmid particle in 40 mM K⁺, 5 mM Mg^{2+} in the presence of transcribed RNAP and TopI.
(A) 3D reconstruction of the plasmid particle (index no. 232). The first column shows seven representative tilt images from –45° to +45° in step of 15°. The second, third, and fourth columns show 3D projections of the particle with spherical, ellipsoidal (thinner along the z-dimension), and particle-shaped masks, respectively. The fifth column displays the 3D projections of the enhanced and IsoNet missing-wedge-corrected particle. The sixth and seventh columns present the final 3D map and the flexibly fitted model, respectively. (B) Two random cross-sectional views (12 nm thickness) of the plasmid density map along its plectoneme axis are shown in the left-middle panel. The intensity profile along the line crossing the two high-density DNA spots is displayed in the right panel. (C) Resolution assessment of the final 3D map using Fourier shell correlation (FSC). Two criteria are shown: FSC between two half-maps reconstructed from even and odd frames (evaluated at 0.143) and FSC between the final 3D map and the fitted model (evaluated at 0.5). (D) Zoomed-in views of the final 3D density map from panel A, displayed at two contour levels. (E) Superimposition of the high-contour level map from panel D onto its flexibly fitted model.

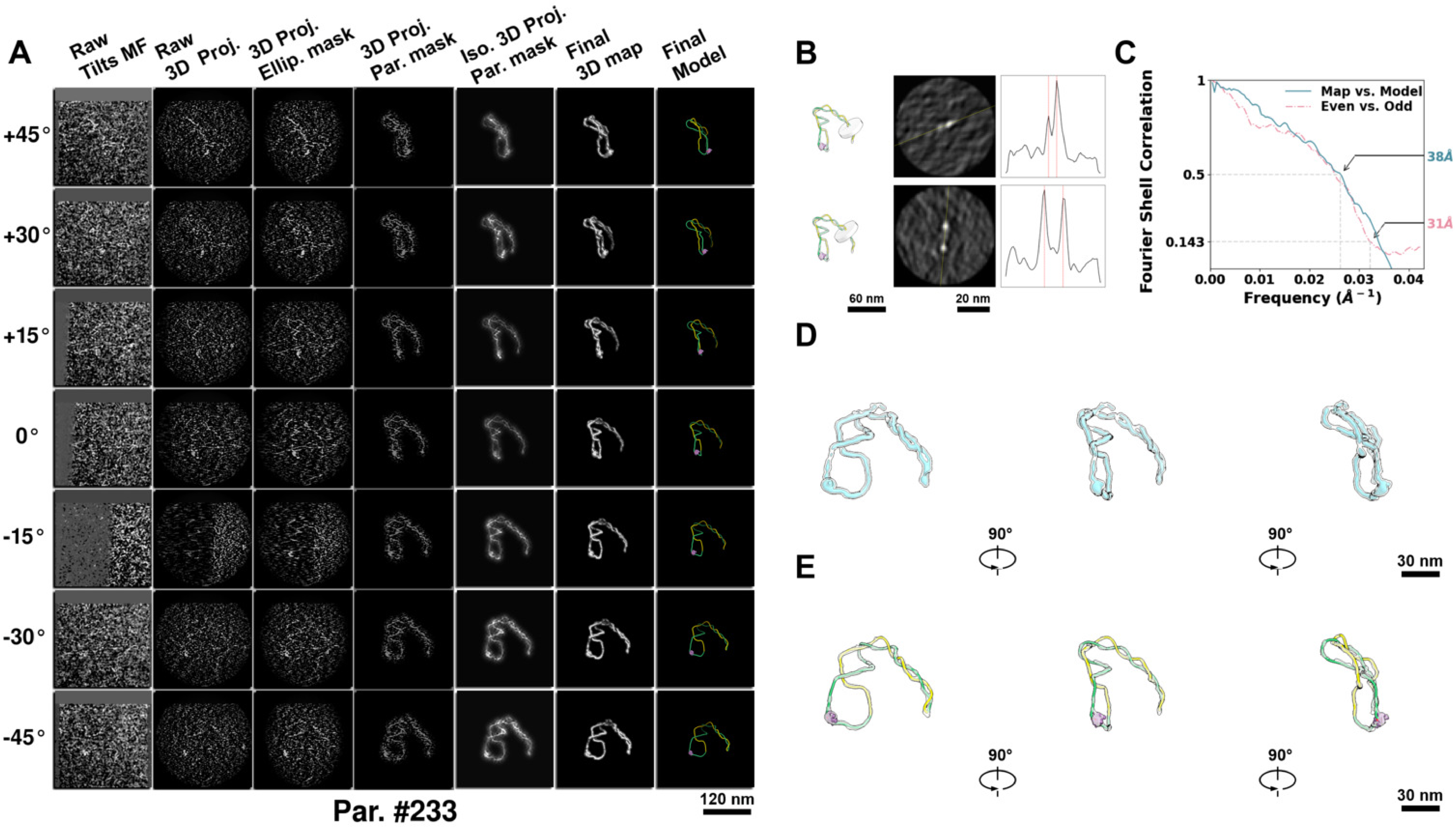

Supplementary Figure 233. Cryo-electron tomography (cryo-ET) 3D reconstruction of an individual plasmid particle in 40 mM K+, 5 mM Mg2+ in the presence of transcribed RNAP and TopI.
(A) 3D reconstruction of the plasmid particle (index no. 233). The first column shows seven representative tilt images from –45° to +45° in step of 15°. The second, third, and fourth columns show 3D projections of the particle with spherical, ellipsoidal (thinner along the z-dimension), and particle-shaped masks, respectively. The fifth column displays the 3D projections of the enhanced and IsoNet missing-wedge-corrected particle. The sixth and seventh columns present the final 3D map and the flexibly fitted model, respectively. (B) Two random cross-sectional views (12 nm thickness) of the plasmid density map along its plectoneme axis are shown in the left-middle panel. The intensity profile along the line crossing the two high-density DNA spots is displayed in the right panel. (C) Resolution assessment of the final 3D map using Fourier shell correlation (FSC). Two criteria are shown: FSC between two half-maps reconstructed from even and odd frames (evaluated at 0.143) and FSC between the final 3D map and the fitted model (evaluated at 0.5). (D) Zoomed-in views of the final 3D density map from panel A, displayed at two contour levels. (E) Superimposition of the high-contour level map from panel D onto its flexibly fitted model.

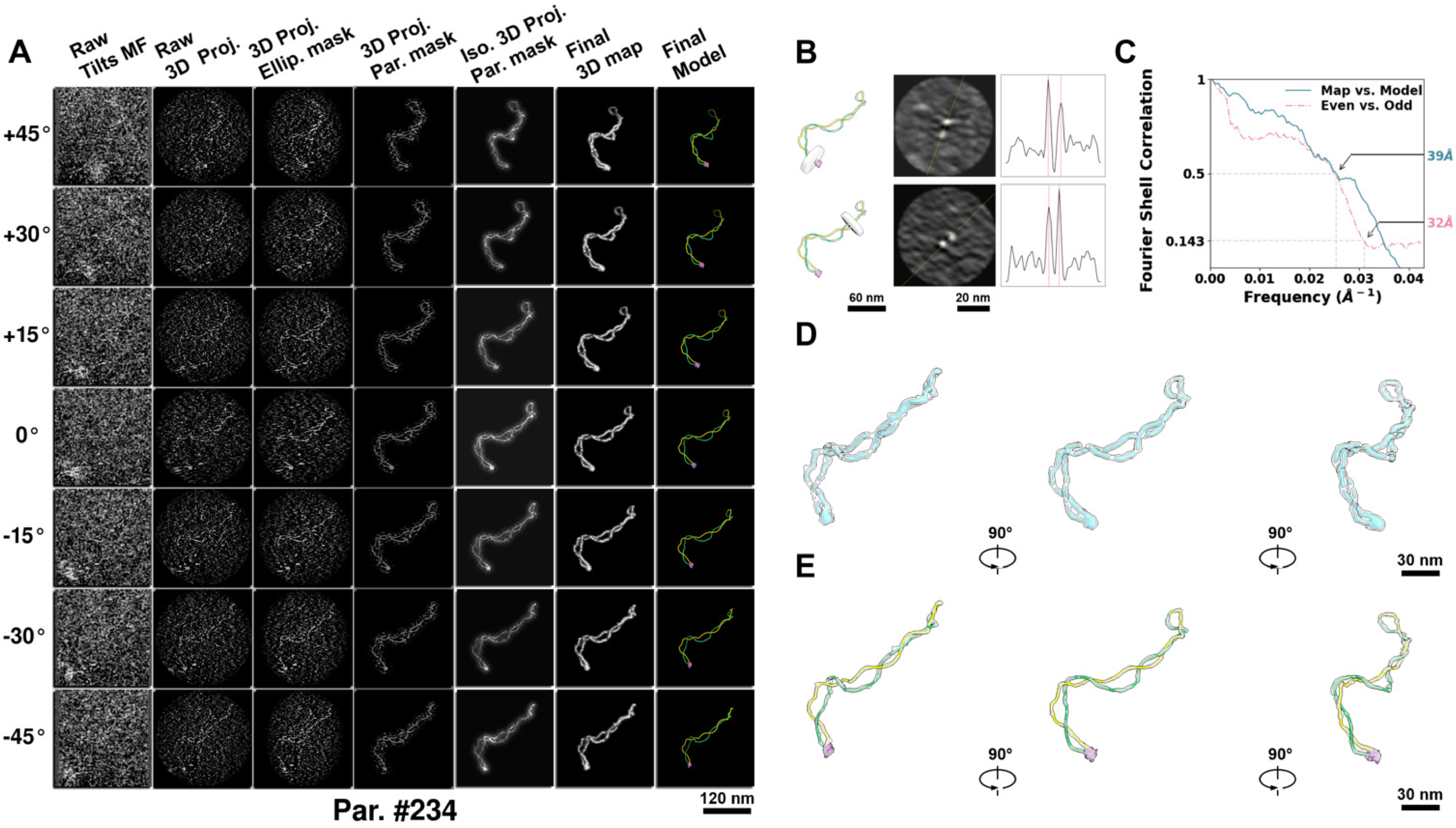

Supplementary Figure 234. Cryo-electron tomography (cryo-ET) 3D reconstruction of an individual plasmid particle in 40 mM K+, 5 mM Mg2+ in the presence of transcribed RNAP and TopI.
(A) 3D reconstruction of the plasmid particle (index no. 234). The first column shows seven representative tilt images from –45° to +45° in step of 15°. The second, third, and fourth columns show 3D projections of the particle with spherical, ellipsoidal (thinner along the z-dimension), and particle-shaped masks, respectively. The fifth column displays the 3D projections of the enhanced and IsoNet missing-wedge-corrected particle. The sixth and seventh columns present the final 3D map and the flexibly fitted model, respectively. (B) Two random cross-sectional views (12 nm thickness) of the plasmid density map along its plectoneme axis are shown in the left-middle panel. The intensity profile along the line crossing the two high-density DNA spots is displayed in the right panel. (C) Resolution assessment of the final 3D map using Fourier shell correlation (FSC). Two criteria are shown: FSC between two half-maps reconstructed from even and odd frames (evaluated at 0.143) and FSC between the final 3D map and the fitted model (evaluated at 0.5). (D) Zoomed-in views of the final 3D density map from panel A, displayed at two contour levels. (E) Superimposition of the high-contour level map from panel D onto its flexibly fitted model.

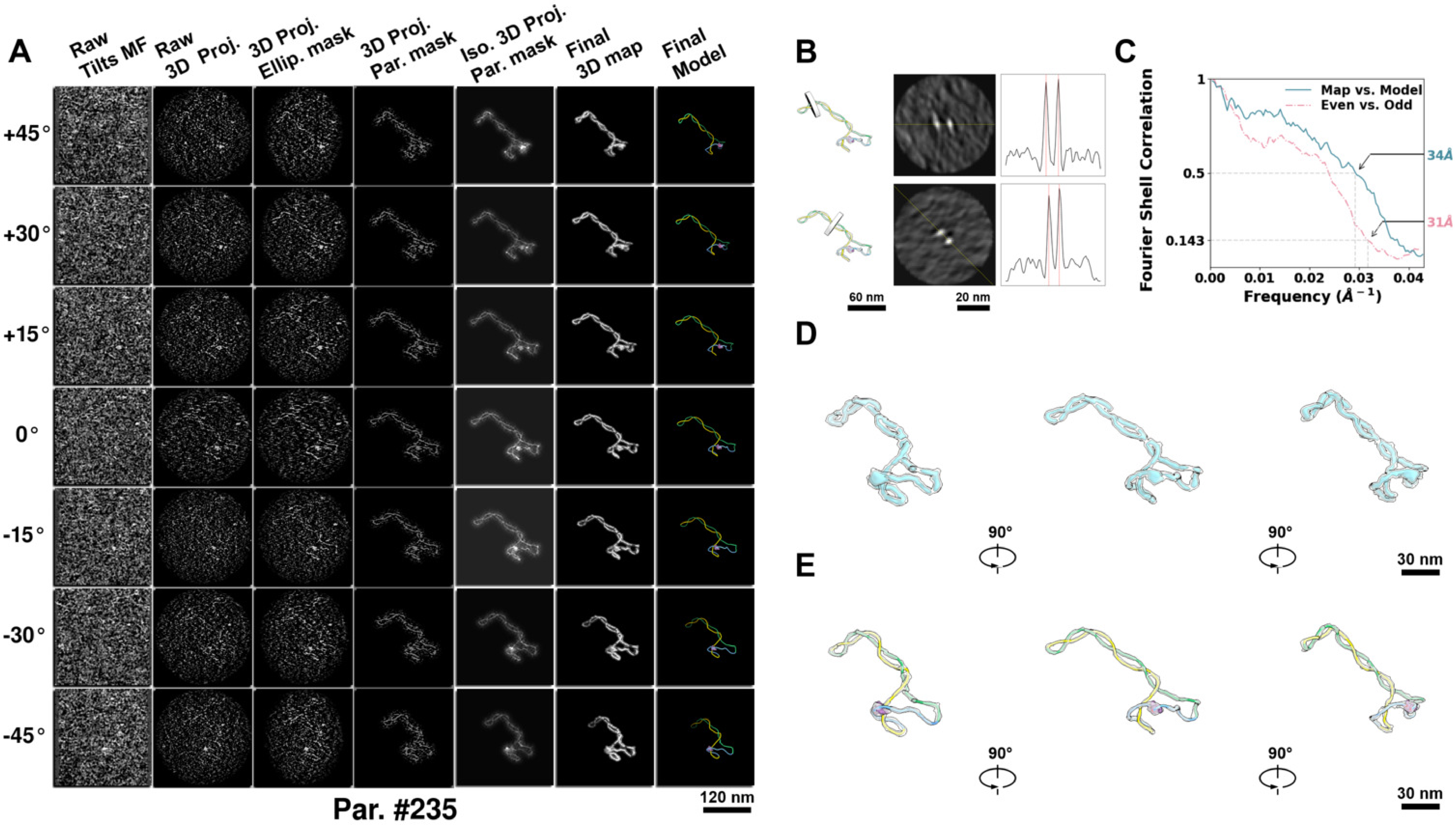

Supplementary Figure 235. Cryo-electron tomography (cryo-ET) 3D reconstruction of an individual plasmid particle in 40 mM K^+, 5 mM Mg^{2+} in the presence of transcribed RNAP and TopI.
(A) 3D reconstruction of the plasmid particle (index no. 235). The first column shows seven representative tilt images from –45° to +45° in step of 15°. The second, third, and fourth columns show 3D projections of the particle with spherical, ellipsoidal (thinner along the z-dimension), and particle-shaped masks, respectively. The fifth column displays the 3D projections of the enhanced and IsoNet missing-wedge-corrected particle. The sixth and seventh columns present the final 3D map and the flexibly fitted model, respectively. (B) Two random cross-sectional views (12 nm thickness) of the plasmid density map along its plectoneme axis are shown in the left-middle panel. The intensity profile along the line crossing the two high-density DNA spots is displayed in the right panel. (C) Resolution assessment of the final 3D map using Fourier shell correlation (FSC). Two criteria are shown: FSC between two half-maps reconstructed from even and odd frames (evaluated at 0.143) and FSC between the final 3D map and the fitted model (evaluated at 0.5). (D) Zoomed-in views of the final 3D density map from panel A, displayed at two contour levels. (E) Superimposition of the high-contour level map from panel D onto its flexibly fitted model.

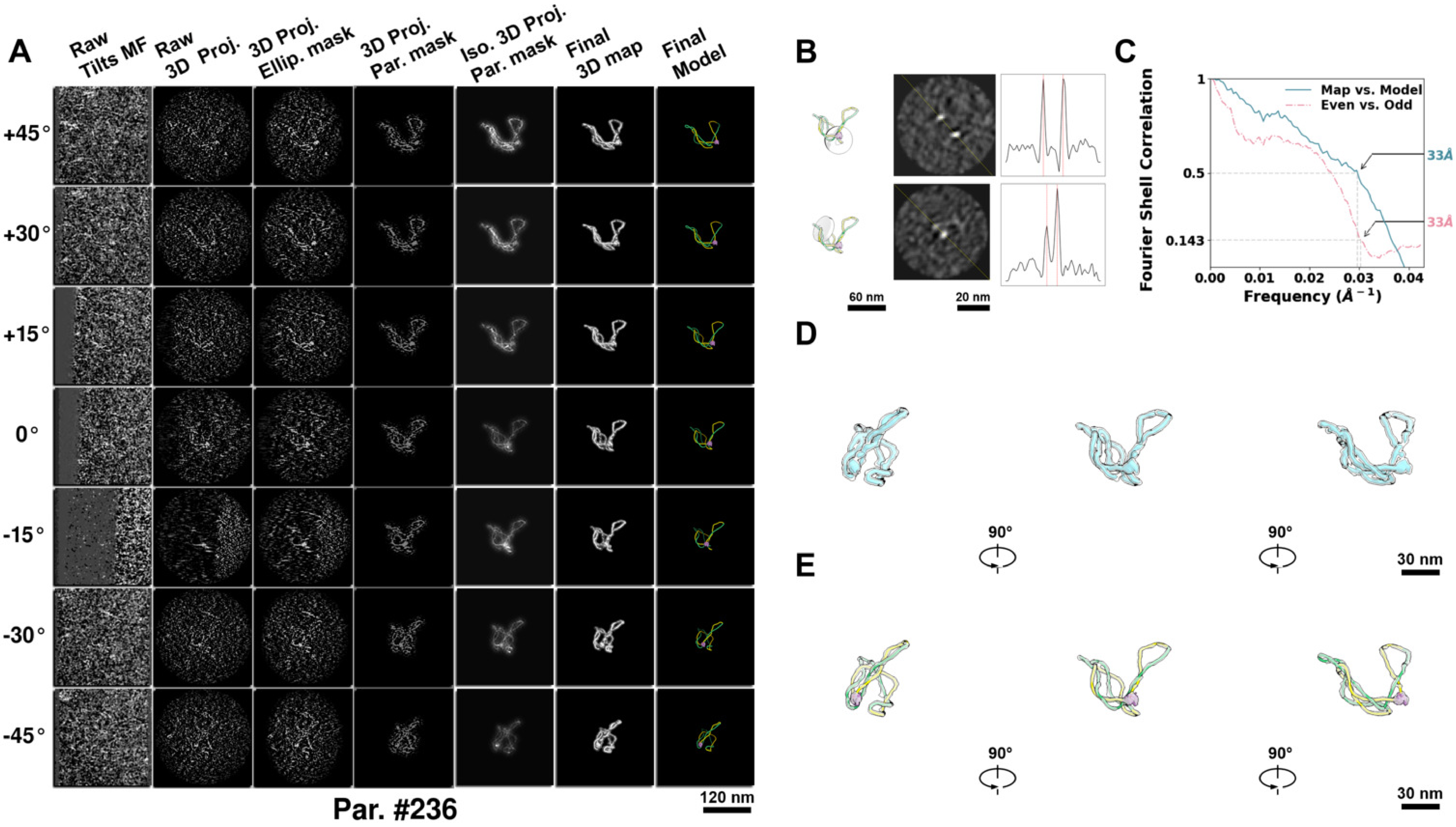

Supplementary Figure 236. Cryo-electron tomography (cryo-ET) 3D reconstruction of an individual plasmid particle in 40 mM K^+, 5 mM Mg^{2+} in the presence of transcribed RNAP and TopI.
(A) 3D reconstruction of the plasmid particle (index no. 236). The first column shows seven representative tilt images from –45° to +45° in step of 15°. The second, third, and fourth columns show 3D projections of the particle with spherical, ellipsoidal (thinner along the z-dimension), and particle-shaped masks, respectively. The fifth column displays the 3D projections of the enhanced and IsoNet missing-wedge-corrected particle. The sixth and seventh columns present the final 3D map and the flexibly fitted model, respectively. (B) Two random cross-sectional views (12 nm thickness) of the plasmid density map along its plectoneme axis are shown in the left-middle panel. The intensity profile along the line crossing the two high-density DNA spots is displayed in the right panel. (C) Resolution assessment of the final 3D map using Fourier shell correlation (FSC). Two criteria are shown: FSC between two half-maps reconstructed from even and odd frames (evaluated at 0.143) and FSC between the final 3D map and the fitted model (evaluated at 0.5). (D) Zoomed-in views of the final 3D density map from panel A, displayed at two contour levels. (E) Superimposition of the high-contour level map from panel D onto its flexibly fitted model.

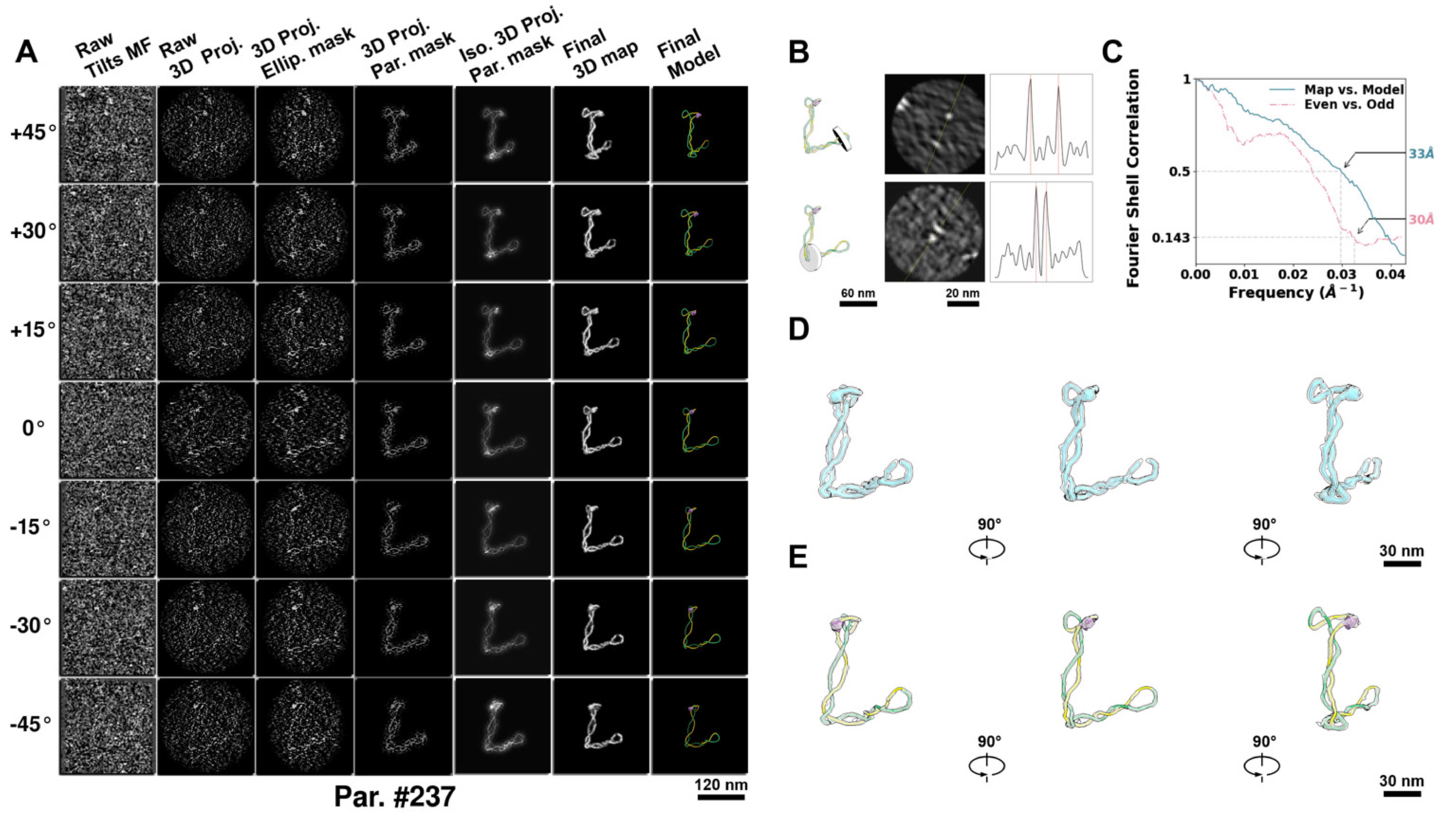

Supplementary Figure 237. Cryo-electron tomography (cryo-ET) 3D reconstruction of an individual plasmid particle in 40 mM K^+, 5 mM Mg^{2+} in the presence of transcribed RNAP and TopI.
(A) 3D reconstruction of the plasmid particle (index no. 237). The first column shows seven representative tilt images from –45° to +45° in step of 15°. The second, third, and fourth columns show 3D projections of the particle with spherical, ellipsoidal (thinner along the z-dimension), and particle-shaped masks, respectively. The fifth column displays the 3D projections of the enhanced and IsoNet missing-wedge-corrected particle. The sixth and seventh columns present the final 3D map and the flexibly fitted model, respectively. (B) Two random cross-sectional views (12 nm thickness) of the plasmid density map along its plectoneme axis are shown in the left-middle panel. The intensity profile along the line crossing the two high-density DNA spots is displayed in the right panel. (C) Resolution assessment of the final 3D map using Fourier shell correlation (FSC). Two criteria are shown: FSC between two half-maps reconstructed from even and odd frames (evaluated at 0.143) and FSC between the final 3D map and the fitted model (evaluated at 0.5). (D) Zoomed-in views of the final 3D density map from panel A, displayed at two contour levels. (E) Superimposition of the high-contour level map from panel D onto its flexibly fitted model.

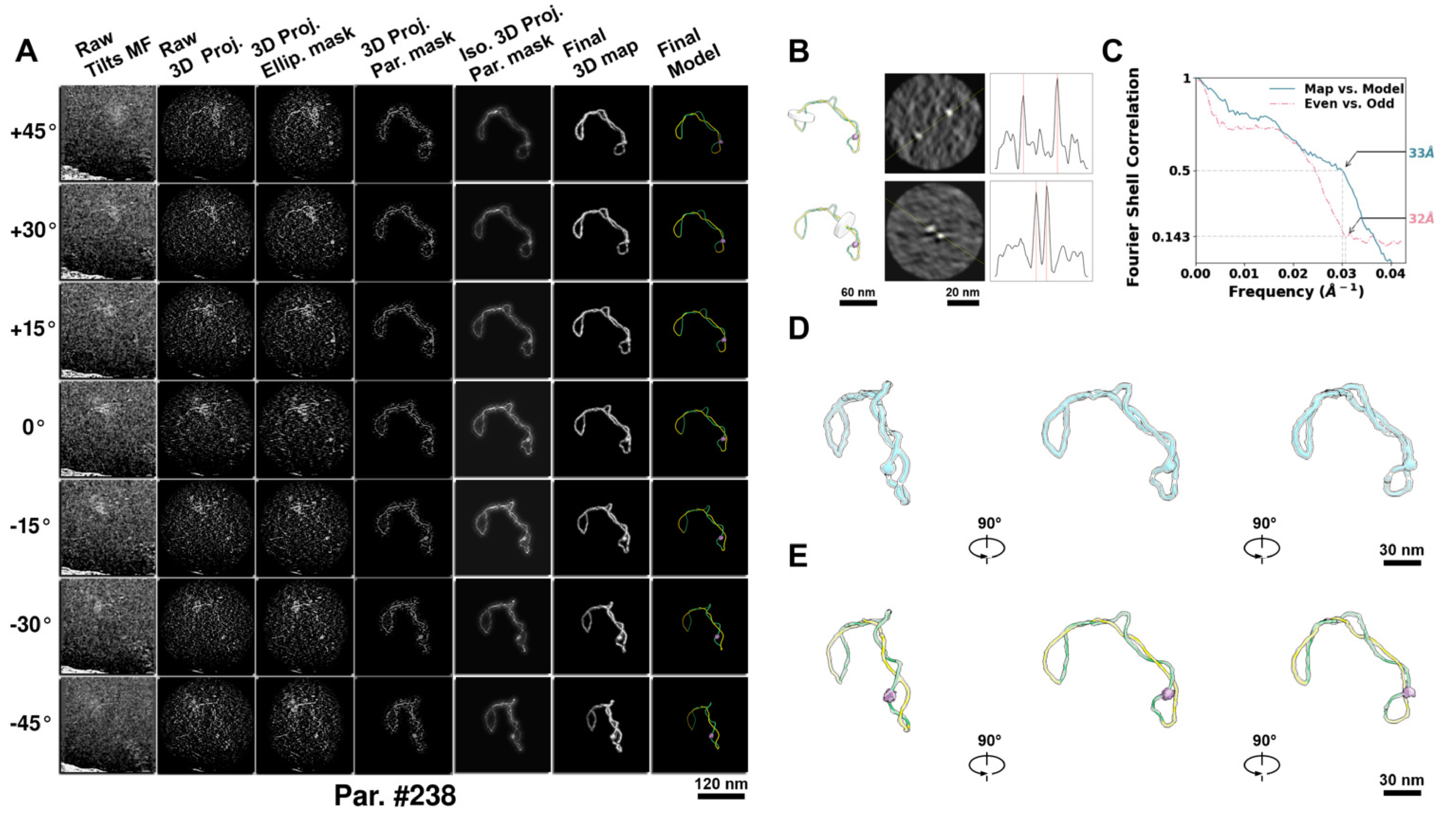

Supplementary Figure 238. Cryo-electron tomography (cryo-ET) 3D reconstruction of an individual plasmid particle in 40 mM K^+, 5 mM Mg^{2+} in the presence of transcribed RNAP and TopI.
(A) 3D reconstruction of the plasmid particle (index no. 238). The first column shows seven representative tilt images from –45° to +45° in step of 15°. The second, third, and fourth columns show 3D projections of the particle with spherical, ellipsoidal (thinner along the z-dimension), and particle-shaped masks, respectively. The fifth column displays the 3D projections of the enhanced and IsoNet missing-wedge-corrected particle. The sixth and seventh columns present the final 3D map and the flexibly fitted model, respectively. (B) Two random cross-sectional views (12 nm thickness) of the plasmid density map along its plectoneme axis are shown in the left-middle panel. The intensity profile along the line crossing the two high-density DNA spots is displayed in the right panel. (C) Resolution assessment of the final 3D map using Fourier shell correlation (FSC). Two criteria are shown: FSC between two half-maps reconstructed from even and odd frames (evaluated at 0.143) and FSC between the final 3D map and the fitted model (evaluated at 0.5). (D) Zoomed-in views of the final 3D density map from panel A, displayed at two contour levels. (E) Superimposition of the high-contour level map from panel D onto its flexibly fitted model.

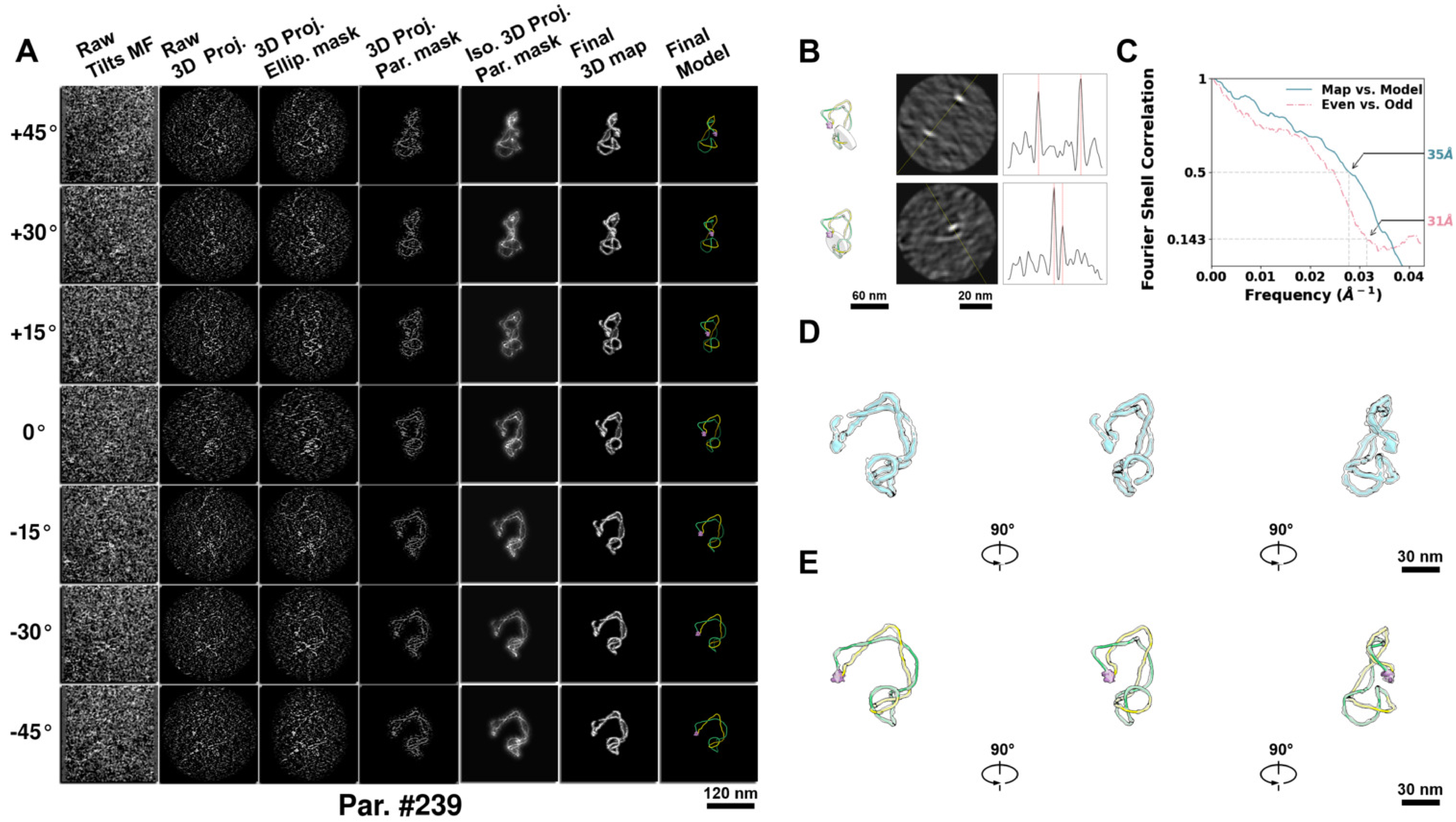

Supplementary Figure 239. Cryo-electron tomography (cryo-ET) 3D reconstruction of an individual plasmid particle in 40 mM K⁺, 5 mM Mg²⁺ in the presence of transcribed RNAP and TopI.
(A) 3D reconstruction of the plasmid particle (index no. 239). The first column shows seven representative tilt images from –45° to +45° in step of 15°. The second, third, and fourth columns show 3D projections of the particle with spherical, ellipsoidal (thinner along the z-dimension), and particle-shaped masks, respectively. The fifth column displays the 3D projections of the enhanced and IsoNet missing-wedge-corrected particle. The sixth and seventh columns present the final 3D map and the flexibly fitted model, respectively. (B) Two random cross-sectional views (12 nm thickness) of the plasmid density map along its plectoneme axis are shown in the left-middle panel. The intensity profile along the line crossing the two high-density DNA spots is displayed in the right panel. (C) Resolution assessment of the final 3D map using Fourier shell correlation (FSC). Two criteria are shown: FSC between two half-maps reconstructed from even and odd frames (evaluated at 0.143) and FSC between the final 3D map and the fitted model (evaluated at 0.5). (D) Zoomed-in views of the final 3D density map from panel A, displayed at two contour levels. (E) Superimposition of the high-contour level map from panel D onto its flexibly fitted model.

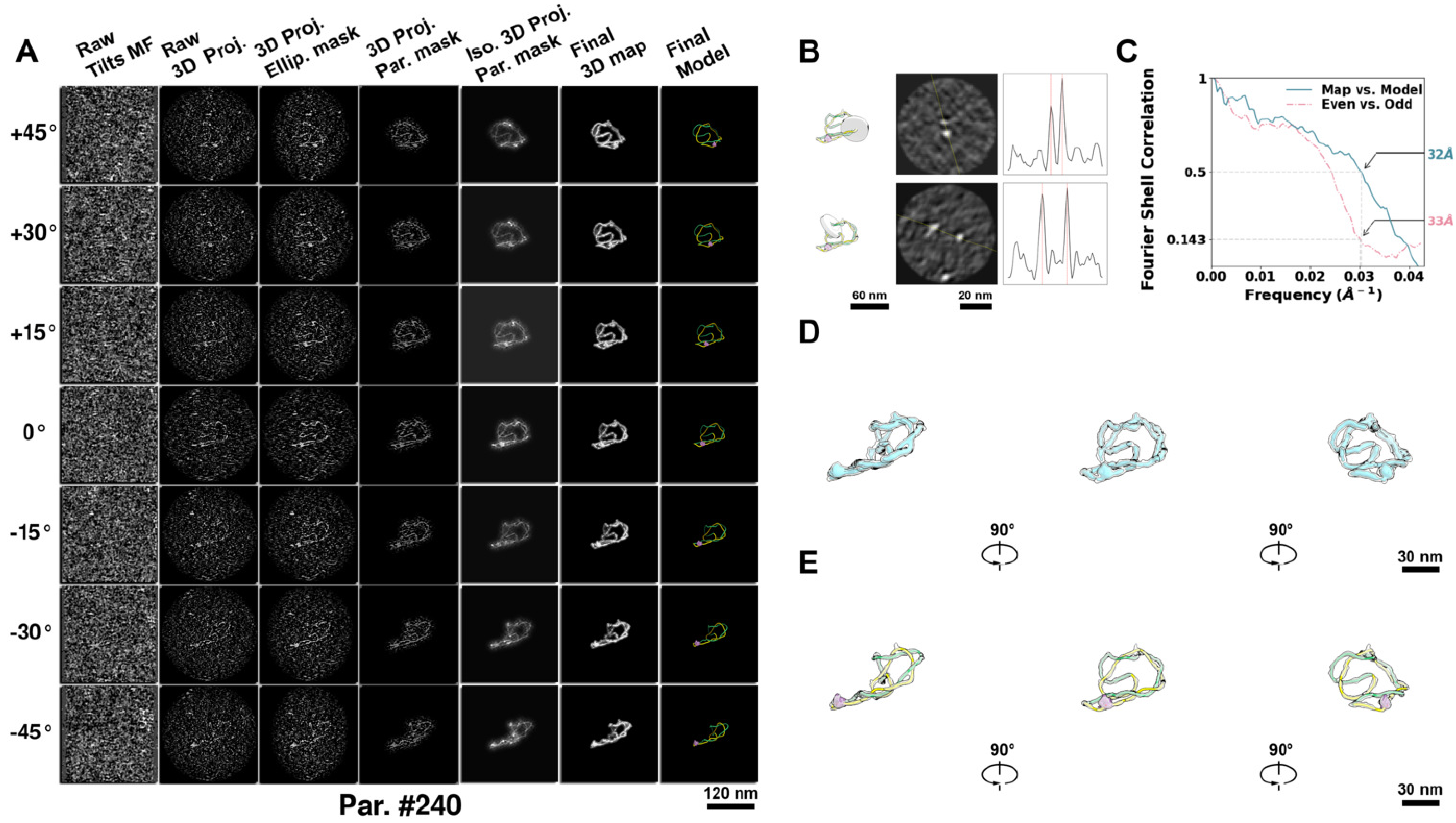

Supplementary Figure 240. Cryo-electron tomography (cryo-ET) 3D reconstruction of an individual plasmid particle in 40 mM K⁺, 5 mM Mg²⁺ in the presence of transcribed RNAP and TopI.
(A) 3D reconstruction of the plasmid particle (index no. 240). The first column shows seven representative tilt images from –45° to +45° in step of 15°. The second, third, and fourth columns show 3D projections of the particle with spherical, ellipsoidal (thinner along the z-dimension), and particle-shaped masks, respectively. The fifth column displays the 3D projections of the enhanced and IsoNet missing-wedge-corrected particle. The sixth and seventh columns present the final 3D map and the flexibly fitted model, respectively. (B) Two random cross-sectional views (12 nm thickness) of the plasmid density map along its plectoneme axis are shown in the left-middle panel. The intensity profile along the line crossing the two high-density DNA spots is displayed in the right panel. (C) Resolution assessment of the final 3D map using Fourier shell correlation (FSC). Two criteria are shown: FSC between two half-maps reconstructed from even and odd frames (evaluated at 0.143) and FSC between the final 3D map and the fitted model (evaluated at 0.5). (D) Zoomed-in views of the final 3D density map from panel A, displayed at two contour levels. (E) Superimposition of the high-contour level map from panel D onto its flexibly fitted model.

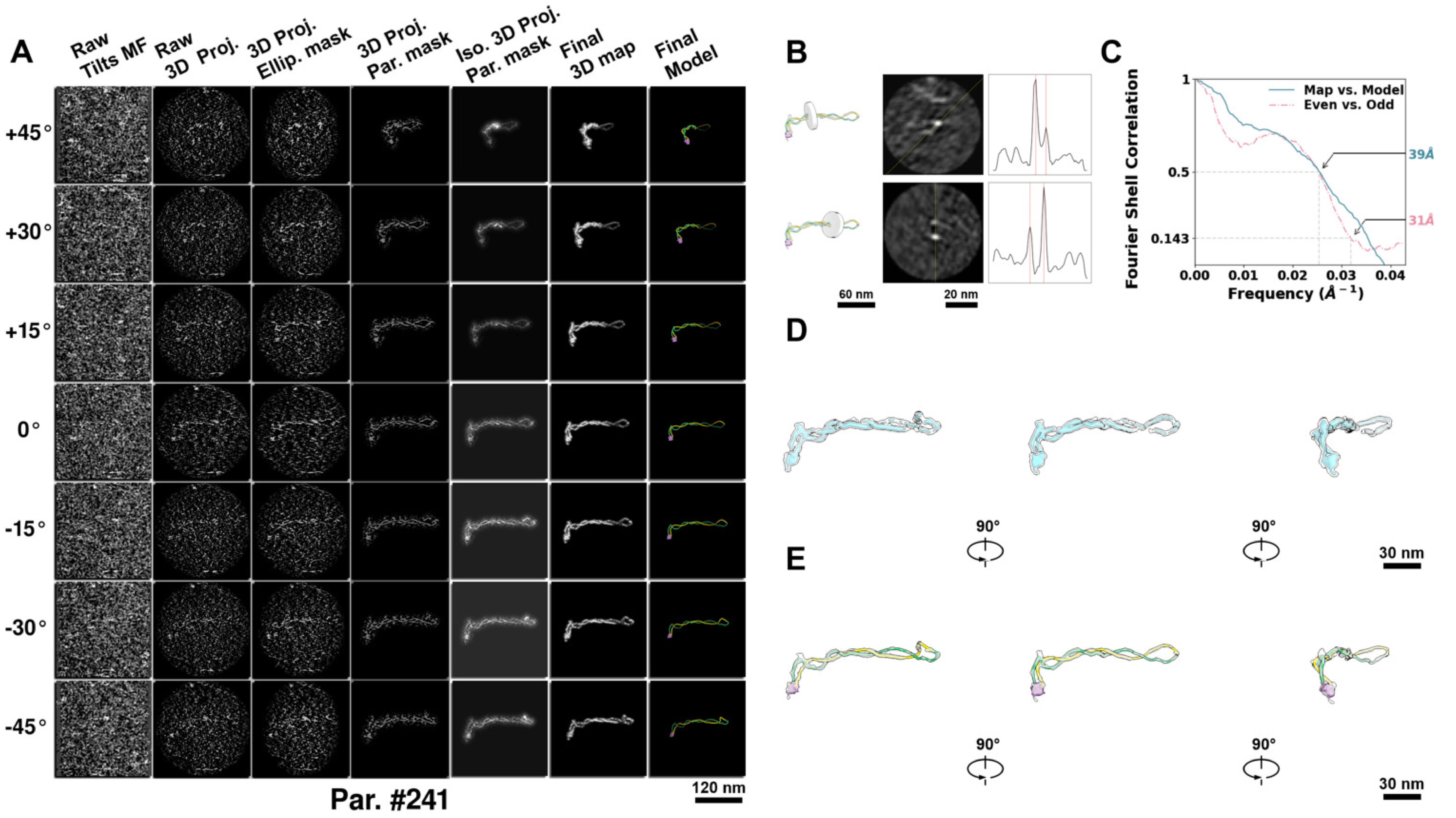

Supplementary Figure 241. Cryo-electron tomography (cryo-ET) 3D reconstruction of an individual plasmid particle in 40 mM K$^+$, 5 mM Mg^{2+} in the presence of transcribed RNAP and TopI.
(A) 3D reconstruction of the plasmid particle (index no. 241). The first column shows seven representative tilt images from –45° to +45° in step of 15°. The second, third, and fourth columns show 3D projections of the particle with spherical, ellipsoidal (thinner along the z-dimension), and particle-shaped masks, respectively. The fifth column displays the 3D projections of the enhanced and IsoNet missing-wedge-corrected particle. The sixth and seventh columns present the final 3D map and the flexibly fitted model, respectively. (B) Two random cross-sectional views (12 nm thickness) of the plasmid density map along its plectoneme axis are shown in the left-middle panel. The intensity profile along the line crossing the two high-density DNA spots is displayed in the right panel. (C) Resolution assessment of the final 3D map using Fourier shell correlation (FSC). Two criteria are shown: FSC between two half-maps reconstructed from even and odd frames (evaluated at 0.143) and FSC between the final 3D map and the fitted model (evaluated at 0.5). (D) Zoomed-in views of the final 3D density map from panel A, displayed at two contour levels. (E) Superimposition of the high-contour level map from panel D onto its flexibly fitted model.

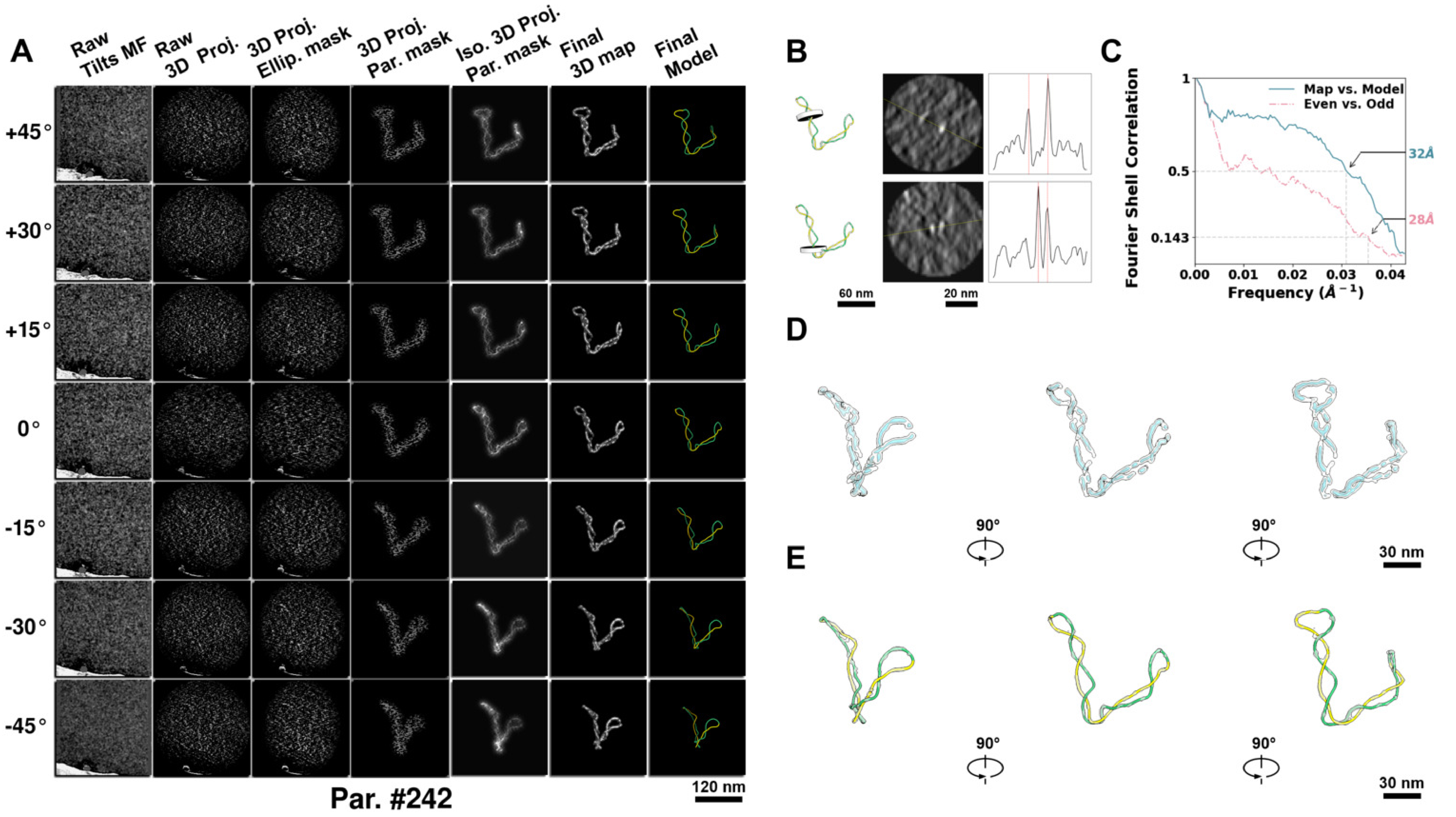

Supplementary Figure 242. Cryo-electron tomography (cryo-ET) 3D reconstruction of an individual plasmid particle in 40 mM K$^+$, 5 mM Mg^{2+} in the presence of transcribed RNAP and TopI.
(A) 3D reconstruction of the plasmid particle (index no. 242). The first column shows seven representative tilt images from –45° to +45° in step of 15°. The second, third, and fourth columns show 3D projections of the particle with spherical, ellipsoidal (thinner along the z-dimension), and particle-shaped masks, respectively. The fifth column displays the 3D projections of the enhanced and IsoNet missing-wedge-corrected particle. The sixth and seventh columns present the final 3D map and the flexibly fitted model, respectively. (B) Two random cross-sectional views (12 nm thickness) of the plasmid density map along its plectoneme axis are shown in the left-middle panel. The intensity profile along the line crossing the two high-density DNA spots is displayed in the right panel. (C) Resolution assessment of the final 3D map using Fourier shell correlation (FSC). Two criteria are shown: FSC between two half-maps reconstructed from even and odd frames (evaluated at 0.143) and FSC between the final 3D map and the fitted model (evaluated at 0.5). (D) Zoomed-in views of the final 3D density map from panel A, displayed at two contour levels. (E) Superimposition of the high-contour level map from panel D onto its flexibly fitted model.

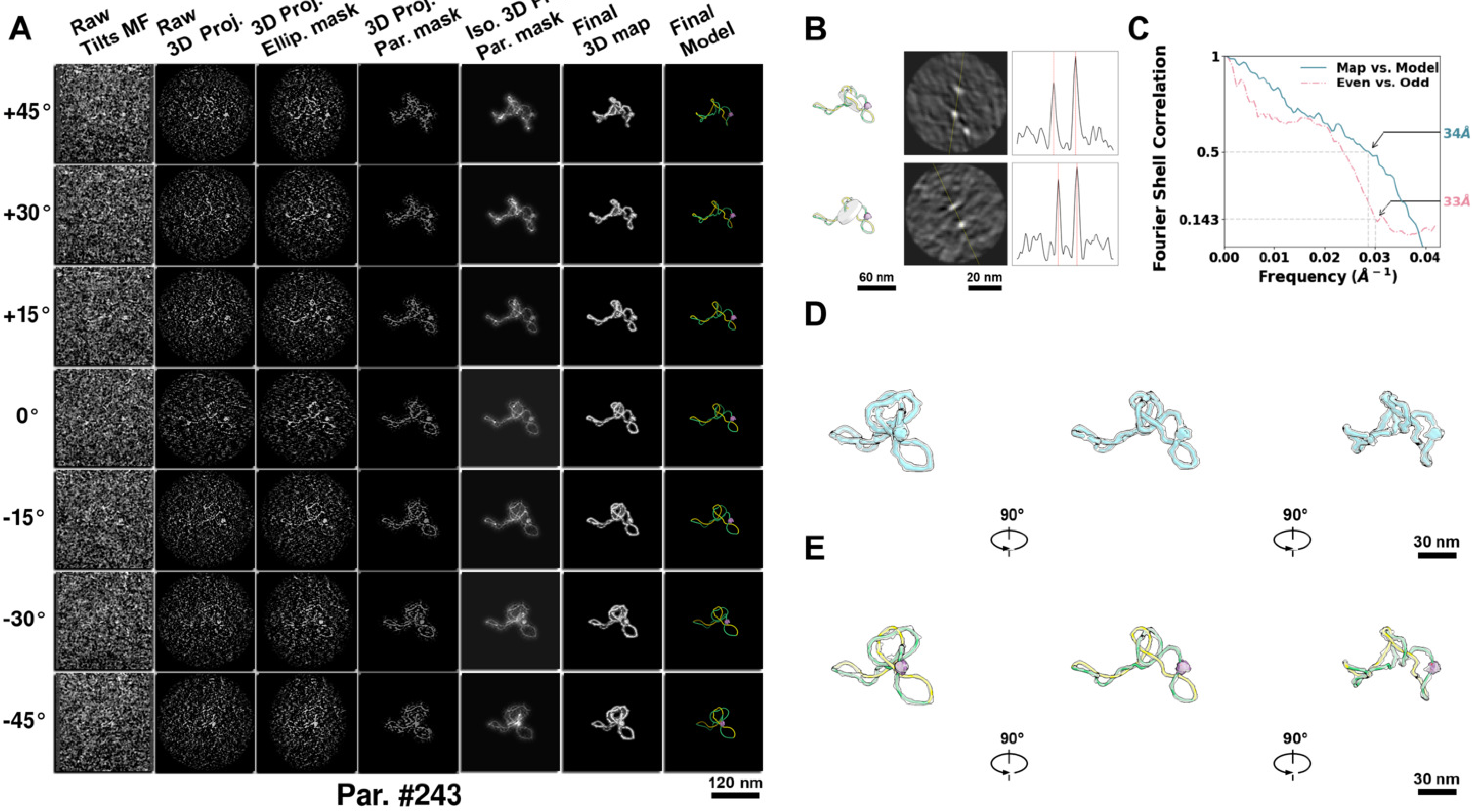

Supplementary Figure 243. Cryo-electron tomography (cryo-ET) 3D reconstruction of an individual plasmid particle in 40 mM K$^+$, 5 mM Mg^{2+} in the presence of transcribed RNAP and TopI.
(A) 3D reconstruction of the plasmid particle (index no. 243). The first column shows seven representative tilt images from –45° to +45° in step of 15°. The second, third, and fourth columns show 3D projections of the particle with spherical, ellipsoidal (thinner along the z-dimension), and particle-shaped masks, respectively. The fifth column displays the 3D projections of the enhanced and IsoNet missing-wedge-corrected particle. The sixth and seventh columns present the final 3D map and the flexibly fitted model, respectively. (B) Two random cross-sectional views (12 nm thickness) of the plasmid density map along its plectoneme axis are shown in the left-middle panel. The intensity profile along the line crossing the two high-density DNA spots is displayed in the right panel. (C) Resolution assessment of the final 3D map using Fourier shell correlation (FSC). Two criteria are shown: FSC between two half-maps reconstructed from even and odd frames (evaluated at 0.143) and FSC between the final 3D map and the fitted model (evaluated at 0.5). (D) Zoomed-in views of the final 3D density map from panel A, displayed at two contour levels. (E) Superimposition of the high-contour level map from panel D onto its flexibly fitted model.

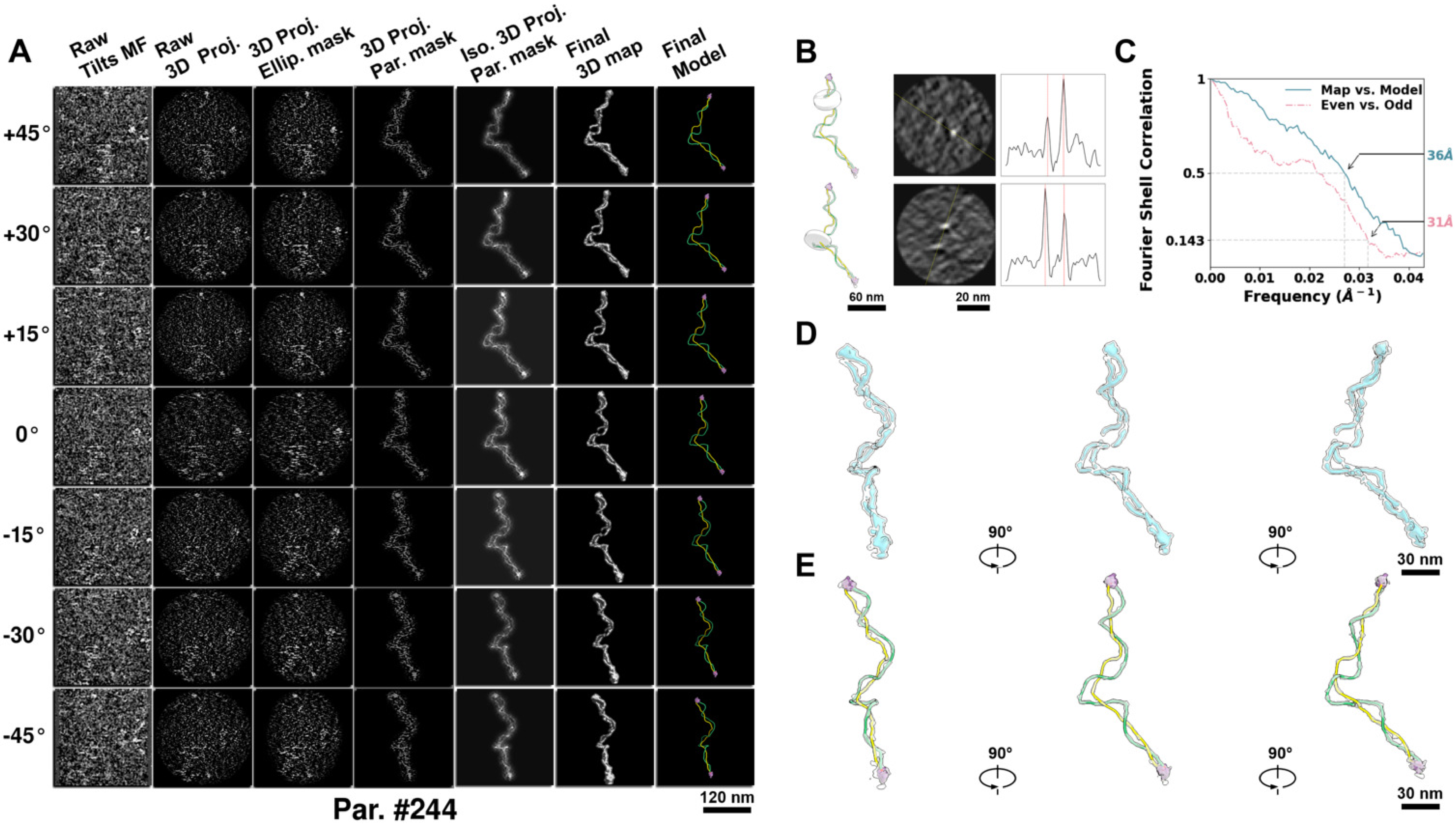

Supplementary Figure 244. Cryo-electron tomography (cryo-ET) 3D reconstruction of an individual plasmid particle in 40 mM K$^+$, 5 mM Mg^{2+} in the presence of transcribed RNAP and TopI.
(A) 3D reconstruction of the plasmid particle (index no. 244). The first column shows seven representative tilt images from –45° to +45° in step of 15°. The second, third, and fourth columns show 3D projections of the particle with spherical, ellipsoidal (thinner along the z-dimension), and particle-shaped masks, respectively. The fifth column displays the 3D projections of the enhanced and IsoNet missing-wedge-corrected particle. The sixth and seventh columns present the final 3D map and the flexibly fitted model, respectively. (B) Two random cross-sectional views (12 nm thickness) of the plasmid density map along its plectoneme axis are shown in the left-middle panel. The intensity profile along the line crossing the two high-density DNA spots is displayed in the right panel. (C) Resolution assessment of the final 3D map using Fourier shell correlation (FSC). Two criteria are shown: FSC between two half-maps reconstructed from even and odd frames (evaluated at 0.143) and FSC between the final 3D map and the fitted model (evaluated at 0.5). (D) Zoomed-in views of the final 3D density map from panel A, displayed at two contour levels. (E) Superimposition of the high-contour level map from panel D onto its flexibly fitted model.

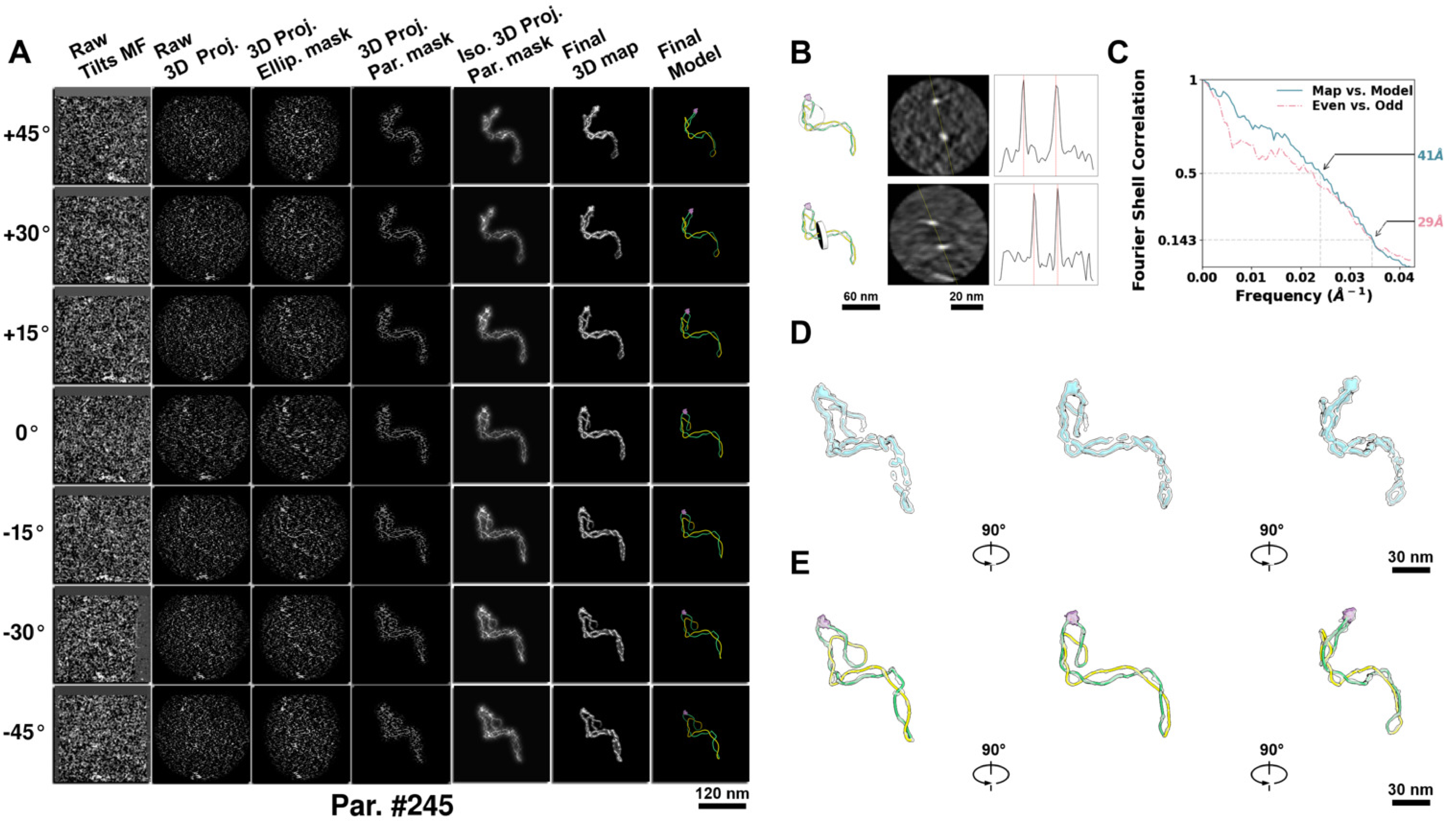

Supplementary Figure 245. Cryo-electron tomography (cryo-ET) 3D reconstruction of an individual plasmid particle in 40 mM K+, 5 mM Mg^{2+} in the presence of transcribed RNAP and TopI.
(A) 3D reconstruction of the plasmid particle (index no. 245). The first column shows seven representative tilt images from –45° to +45° in step of 15°. The second, third, and fourth columns show 3D projections of the particle with spherical, ellipsoidal (thinner along the z-dimension), and particle-shaped masks, respectively. The fifth column displays the 3D projections of the enhanced and IsoNet missing-wedge-corrected particle. The sixth and seventh columns present the final 3D map and the flexibly fitted model, respectively. (B) Two random cross-sectional views (12 nm thickness) of the plasmid density map along its plectoneme axis are shown in the left-middle panel. The intensity profile along the line crossing the two high-density DNA spots is displayed in the right panel. (C) Resolution assessment of the final 3D map using Fourier shell correlation (FSC). Two criteria are shown: FSC between two half-maps reconstructed from even and odd frames (evaluated at 0.143) and FSC between the final 3D map and the fitted model (evaluated at 0.5). (D) Zoomed-in views of the final 3D density map from panel A, displayed at two contour levels. (E) Superimposition of the high-contour level map from panel D onto its flexibly fitted model.

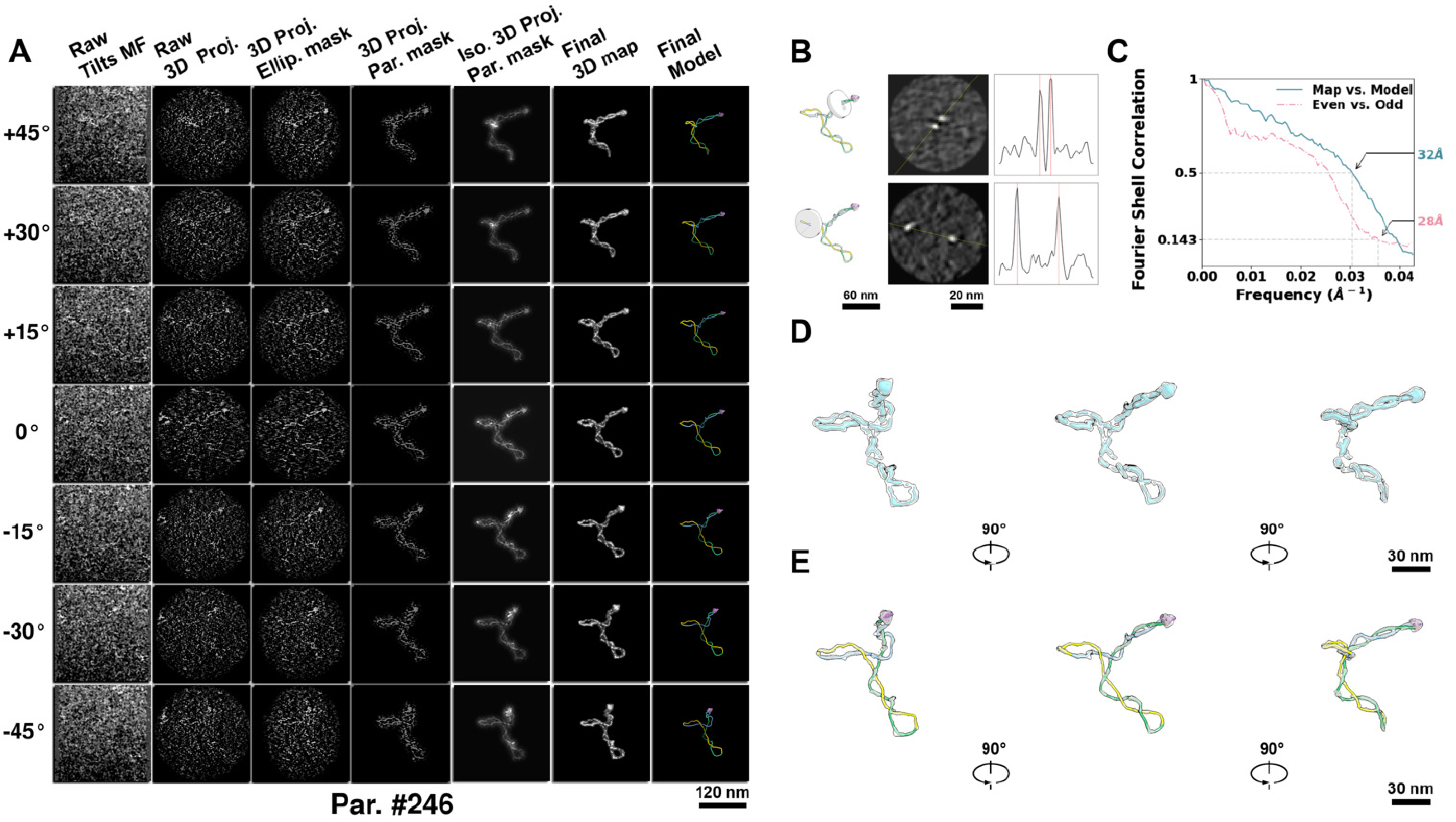

Supplementary Figure 246. Cryo-electron tomography (cryo-ET) 3D reconstruction of an individual plasmid particle in 40 mM K+, 5 mM Mg^{2+} in the presence of transcribed RNAP and TopI.
(A) 3D reconstruction of the plasmid particle (index no. 246). The first column shows seven representative tilt images from –45° to +45° in step of 15°. The second, third, and fourth columns show 3D projections of the particle with spherical, ellipsoidal (thinner along the z-dimension), and particle-shaped masks, respectively. The fifth column displays the 3D projections of the enhanced and IsoNet missing-wedge-corrected particle. The sixth and seventh columns present the final 3D map and the flexibly fitted model, respectively. (B) Two random cross-sectional views (12 nm thickness) of the plasmid density map along its plectoneme axis are shown in the left-middle panel. The intensity profile along the line crossing the two high-density DNA spots is displayed in the right panel. (C) Resolution assessment of the final 3D map using Fourier shell correlation (FSC). Two criteria are shown: FSC between two half-maps reconstructed from even and odd frames (evaluated at 0.143) and FSC between the final 3D map and the fitted model (evaluated at 0.5). (D) Zoomed-in views of the final 3D density map from panel A, displayed at two contour levels. (E) Superimposition of the high-contour level map from panel D onto its flexibly fitted model.

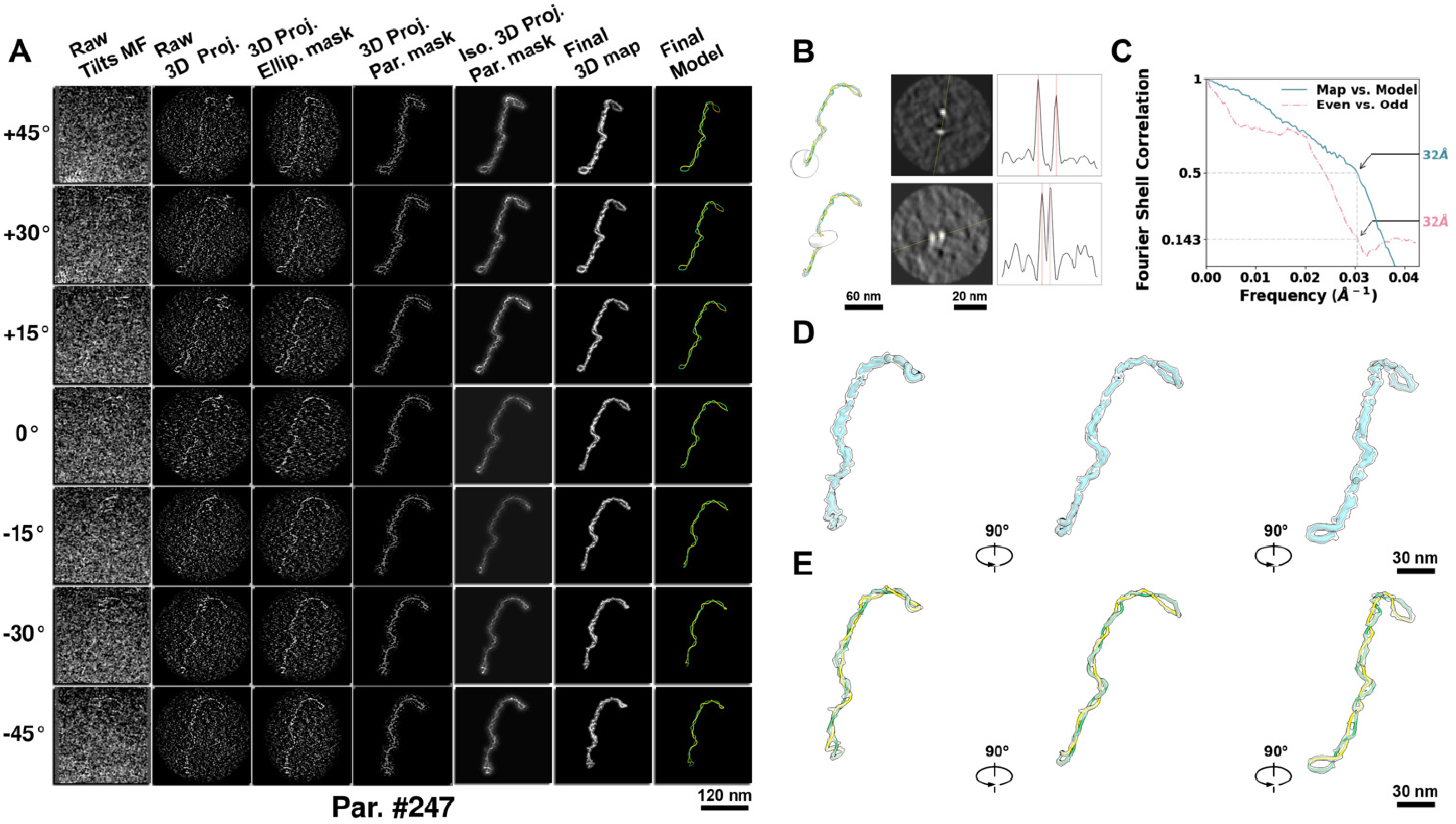

Supplementary Figure 247. Cryo-electron tomography (cryo-ET) 3D reconstruction of an individual plasmid particle in 40 mM K⁺, 5 mM Mg^{2+} in the presence of transcribed RNAP and TopI.
(A) 3D reconstruction of the plasmid particle (index no. 247). The first column shows seven representative tilt images from –45° to +45° in step of 15°. The second, third, and fourth columns show 3D projections of the particle with spherical, ellipsoidal (thinner along the z-dimension), and particle-shaped masks, respectively. The fifth column displays the 3D projections of the enhanced and IsoNet missing-wedge-corrected particle. The sixth and seventh columns present the final 3D map and the flexibly fitted model, respectively. (B) Two random cross-sectional views (12 nm thickness) of the plasmid density map along its plectoneme axis are shown in the left-middle panel. The intensity profile along the line crossing the two high-density DNA spots is displayed in the right panel. (C) Resolution assessment of the final 3D map using Fourier shell correlation (FSC). Two criteria are shown: FSC between two half-maps reconstructed from even and odd frames (evaluated at 0.143) and FSC between the final 3D map and the fitted model (evaluated at 0.5). (D) Zoomed-in views of the final 3D density map from panel A, displayed at two contour levels. (E) Superimposition of the high-contour level map from panel D onto its flexibly fitted model.

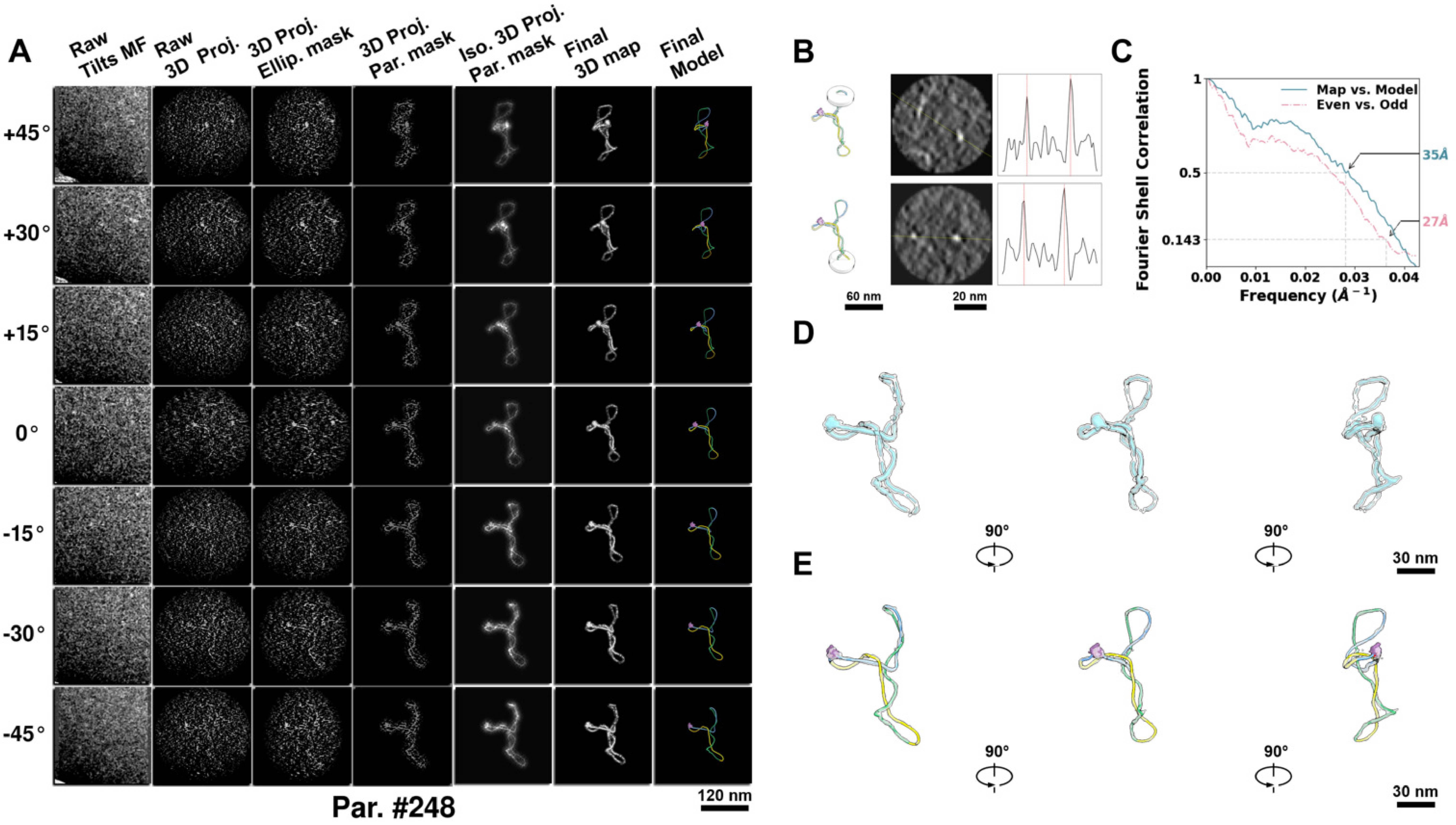

Supplementary Figure 248. Cryo-electron tomography (cryo-ET) 3D reconstruction of an individual plasmid particle in 40 mM K⁺, 5 mM Mg^{2+} in the presence of transcribed RNAP and TopI.
(A) 3D reconstruction of the plasmid particle (index no. 248). The first column shows seven representative tilt images from –45° to +45° in step of 15°. The second, third, and fourth columns show 3D projections of the particle with spherical, ellipsoidal (thinner along the z-dimension), and particle-shaped masks, respectively. The fifth column displays the 3D projections of the enhanced and IsoNet missing-wedge-corrected particle. The sixth and seventh columns present the final 3D map and the flexibly fitted model, respectively. (B) Two random cross-sectional views (12 nm thickness) of the plasmid density map along its plectoneme axis are shown in the left-middle panel. The intensity profile along the line crossing the two high-density DNA spots is displayed in the right panel. (C) Resolution assessment of the final 3D map using Fourier shell correlation (FSC). Two criteria are shown: FSC between two half-maps reconstructed from even and odd frames (evaluated at 0.143) and FSC between the final 3D map and the fitted model (evaluated at 0.5). (D) Zoomed-in views of the final 3D density map from panel A, displayed at two contour levels. (E) Superimposition of the high-contour level map from panel D onto its flexibly fitted model.

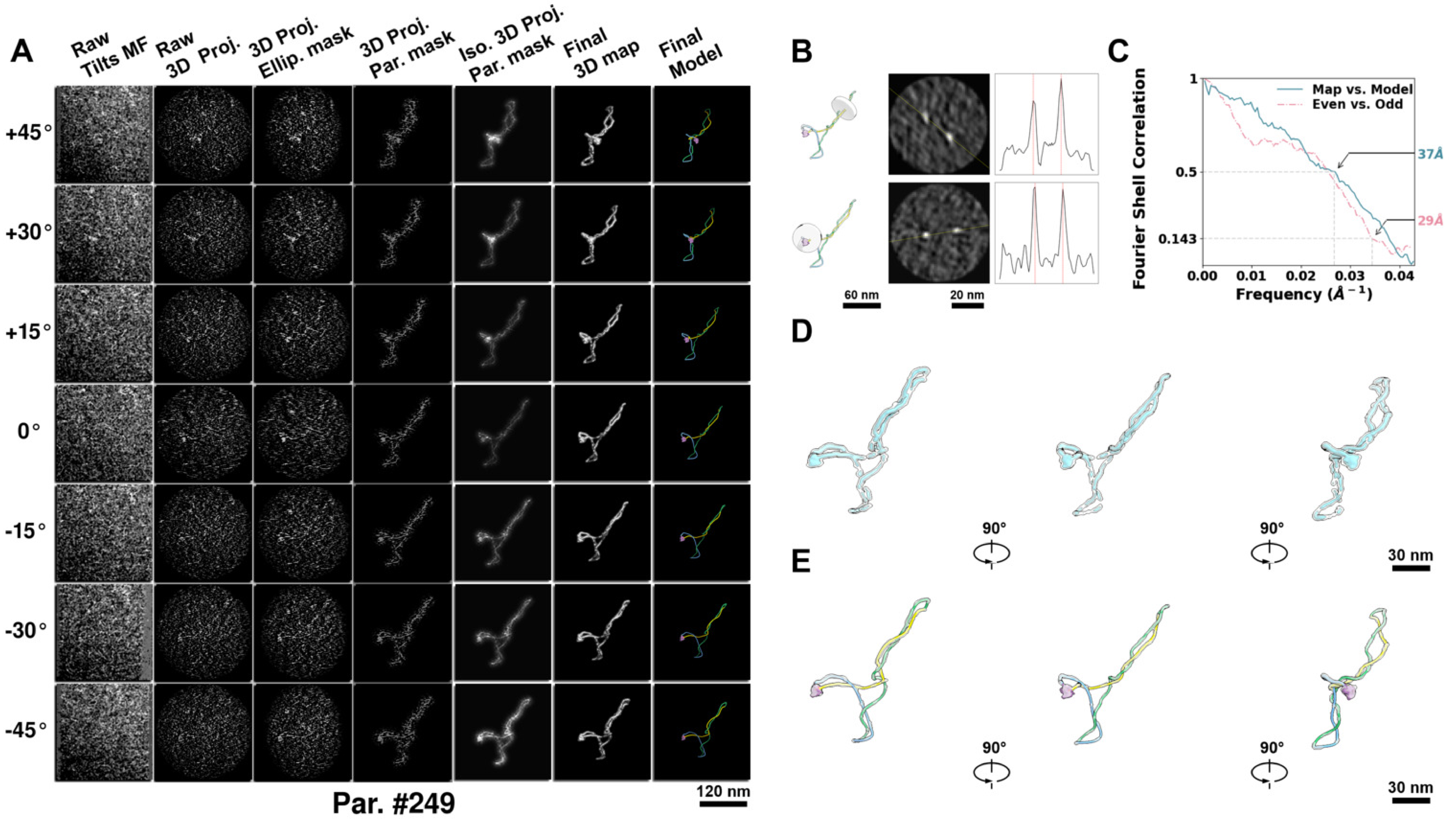

Supplementary Figure 249. Cryo-electron tomography (cryo-ET) 3D reconstruction of an individual plasmid particle in 40 mM K^+, 5 mM Mg^{2+} in the presence of transcribed RNAP and TopI.
(A) 3D reconstruction of the plasmid particle (index no. 249). The first column shows seven representative tilt images from –45° to +45° in step of 15°. The second, third, and fourth columns show 3D projections of the particle with spherical, ellipsoidal (thinner along the z-dimension), and particle-shaped masks, respectively. The fifth column displays the 3D projections of the enhanced and IsoNet missing-wedge-corrected particle. The sixth and seventh columns present the final 3D map and the flexibly fitted model, respectively. (B) Two random cross-sectional views (12 nm thickness) of the plasmid density map along its plectoneme axis are shown in the left-middle panel. The intensity profile along the line crossing the two high-density DNA spots is displayed in the right panel. (C) Resolution assessment of the final 3D map using Fourier shell correlation (FSC). Two criteria are shown: FSC between two half-maps reconstructed from even and odd frames (evaluated at 0.143) and FSC between the final 3D map and the fitted model (evaluated at 0.5). (D) Zoomed-in views of the final 3D density map from panel A, displayed at two contour levels. (E) Superimposition of the high-contour level map from panel D onto its flexibly fitted model.

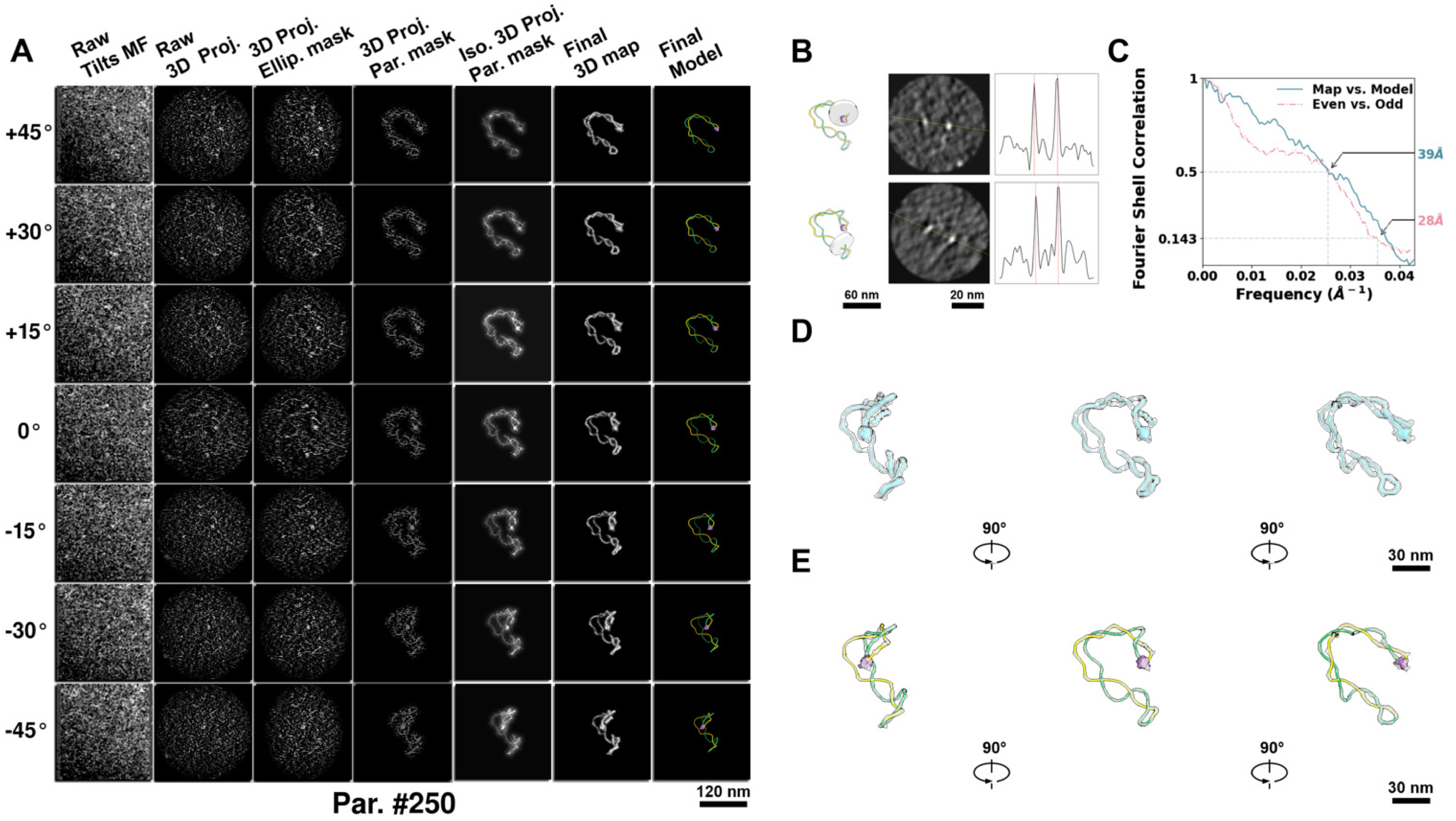

Supplementary Figure 250. Cryo-electron tomography (cryo-ET) 3D reconstruction of an individual plasmid particle in 40 mM K^+, 5 mM Mg^{2+} in the presence of transcribed RNAP and TopI.
(A) 3D reconstruction of the plasmid particle (index no. 250). The first column shows seven representative tilt images from –45° to +45° in step of 15°. The second, third, and fourth columns show 3D projections of the particle with spherical, ellipsoidal (thinner along the z-dimension), and particle-shaped masks, respectively. The fifth column displays the 3D projections of the enhanced and IsoNet missing-wedge-corrected particle. The sixth and seventh columns present the final 3D map and the flexibly fitted model, respectively. (B) Two random cross-sectional views (12 nm thickness) of the plasmid density map along its plectoneme axis are shown in the left-middle panel. The intensity profile along the line crossing the two high-density DNA spots is displayed in the right panel. (C) Resolution assessment of the final 3D map using Fourier shell correlation (FSC). Two criteria are shown: FSC between two half-maps reconstructed from even and odd frames (evaluated at 0.143) and FSC between the final 3D map and the fitted model (evaluated at 0.5). (D) Zoomed-in views of the final 3D density map from panel A, displayed at two contour levels. (E) Superimposition of the high-contour level map from panel D onto its flexibly fitted model.

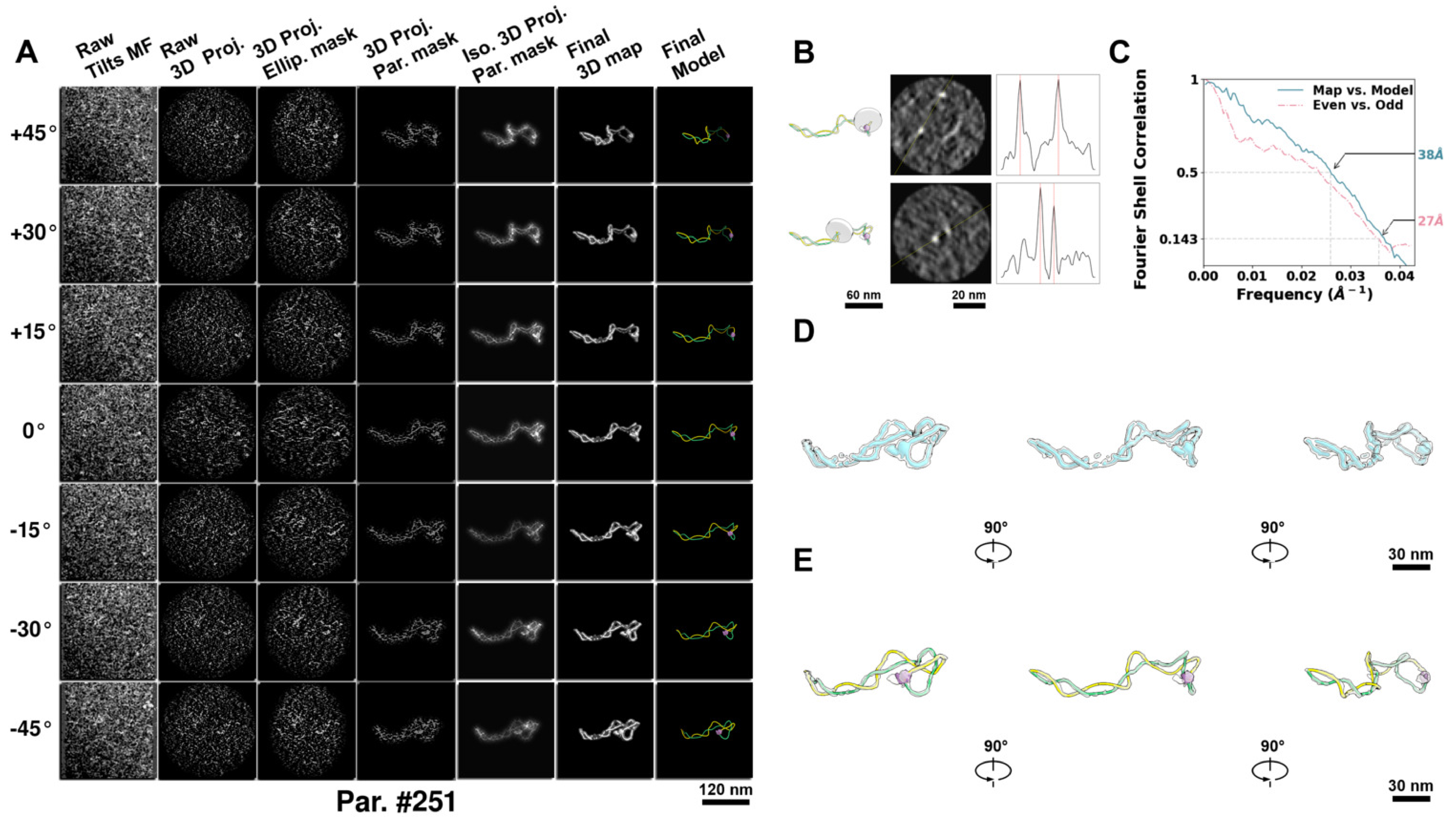

Supplementary Figure 251. Cryo-electron tomography (cryo-ET) 3D reconstruction of an individual plasmid particle in 40 mM K^{+}, 5 mM Mg^{2+} in the presence of transcribed RNAP and TopI.
(A) 3D reconstruction of the plasmid particle (index no. 251). The first column shows seven representative tilt images from –45° to +45° in step of 15°. The second, third, and fourth columns show 3D projections of the particle with spherical, ellipsoidal (thinner along the z-dimension), and particle-shaped masks, respectively. The fifth column displays the 3D projections of the enhanced and IsoNet missing-wedge-corrected particle. The sixth and seventh columns present the final 3D map and the flexibly fitted model, respectively. (B) Two random cross-sectional views (12 nm thickness) of the plasmid density map along its plectoneme axis are shown in the left-middle panel. The intensity profile along the line crossing the two high-density DNA spots is displayed in the right panel. (C) Resolution assessment of the final 3D map using Fourier shell correlation (FSC). Two criteria are shown: FSC between two half-maps reconstructed from even and odd frames (evaluated at 0.143) and FSC between the final 3D map and the fitted model (evaluated at 0.5). (D) Zoomed-in views of the final 3D density map from panel A, displayed at two contour levels. (E) Superimposition of the high-contour level map from panel D onto its flexibly fitted model.

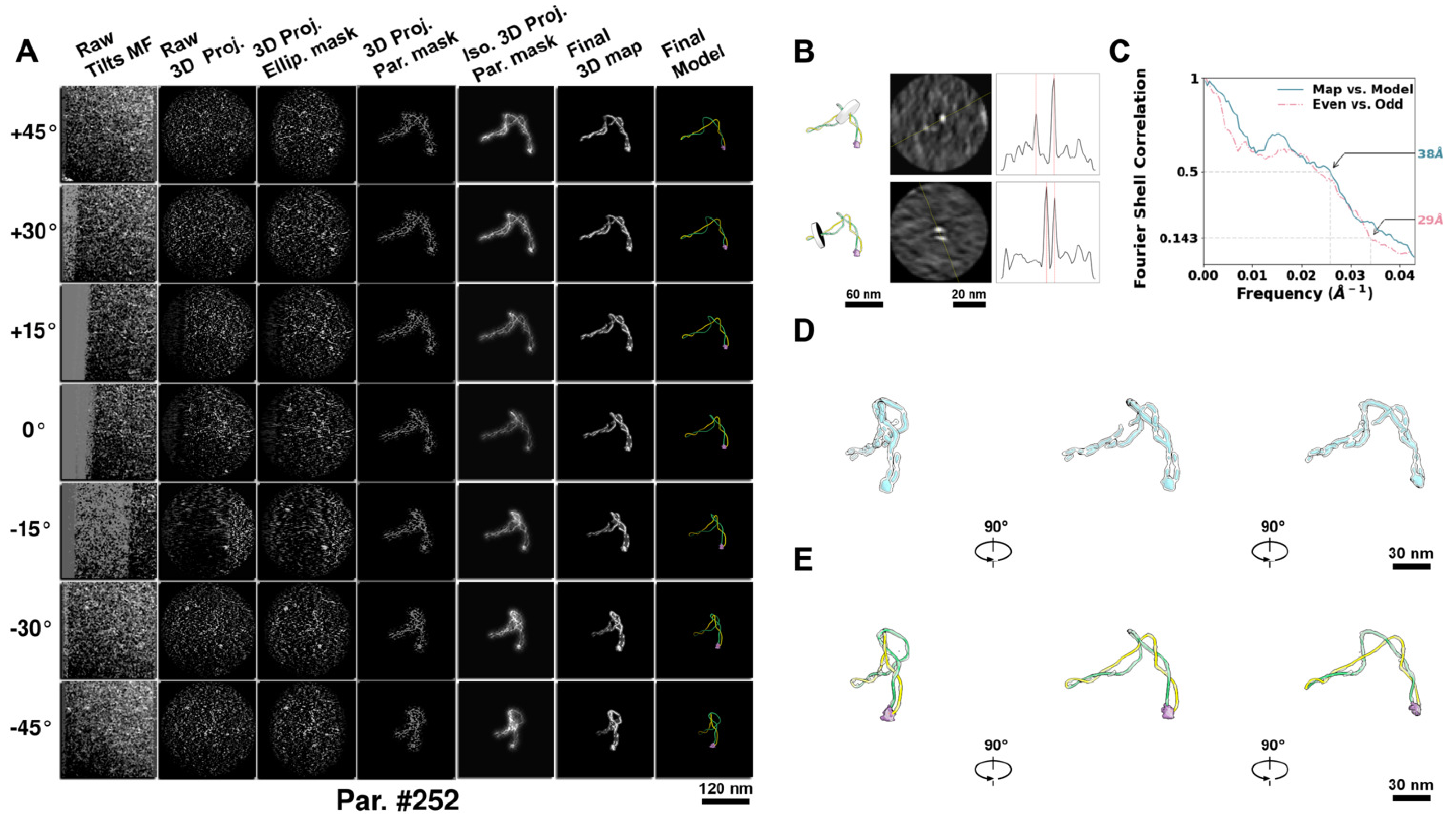

Supplementary Figure 252. Cryo-electron tomography (cryo-ET) 3D reconstruction of an individual plasmid particle in 40 mM K^{+}, 5 mM Mg^{2+} in the presence of transcribed RNAP and TopI.
(A) 3D reconstruction of the plasmid particle (index no. 252). The first column shows seven representative tilt images from –45° to +45° in step of 15°. The second, third, and fourth columns show 3D projections of the particle with spherical, ellipsoidal (thinner along the z-dimension), and particle-shaped masks, respectively. The fifth column displays the 3D projections of the enhanced and IsoNet missing-wedge-corrected particle. The sixth and seventh columns present the final 3D map and the flexibly fitted model, respectively. (B) Two random cross-sectional views (12 nm thickness) of the plasmid density map along its plectoneme axis are shown in the left-middle panel. The intensity profile along the line crossing the two high-density DNA spots is displayed in the right panel. (C) Resolution assessment of the final 3D map using Fourier shell correlation (FSC). Two criteria are shown: FSC between two half-maps reconstructed from even and odd frames (evaluated at 0.143) and FSC between the final 3D map and the fitted model (evaluated at 0.5). (D) Zoomed-in views of the final 3D density map from panel A, displayed at two contour levels. (E) Superimposition of the high-contour level map from panel D onto its flexibly fitted model.

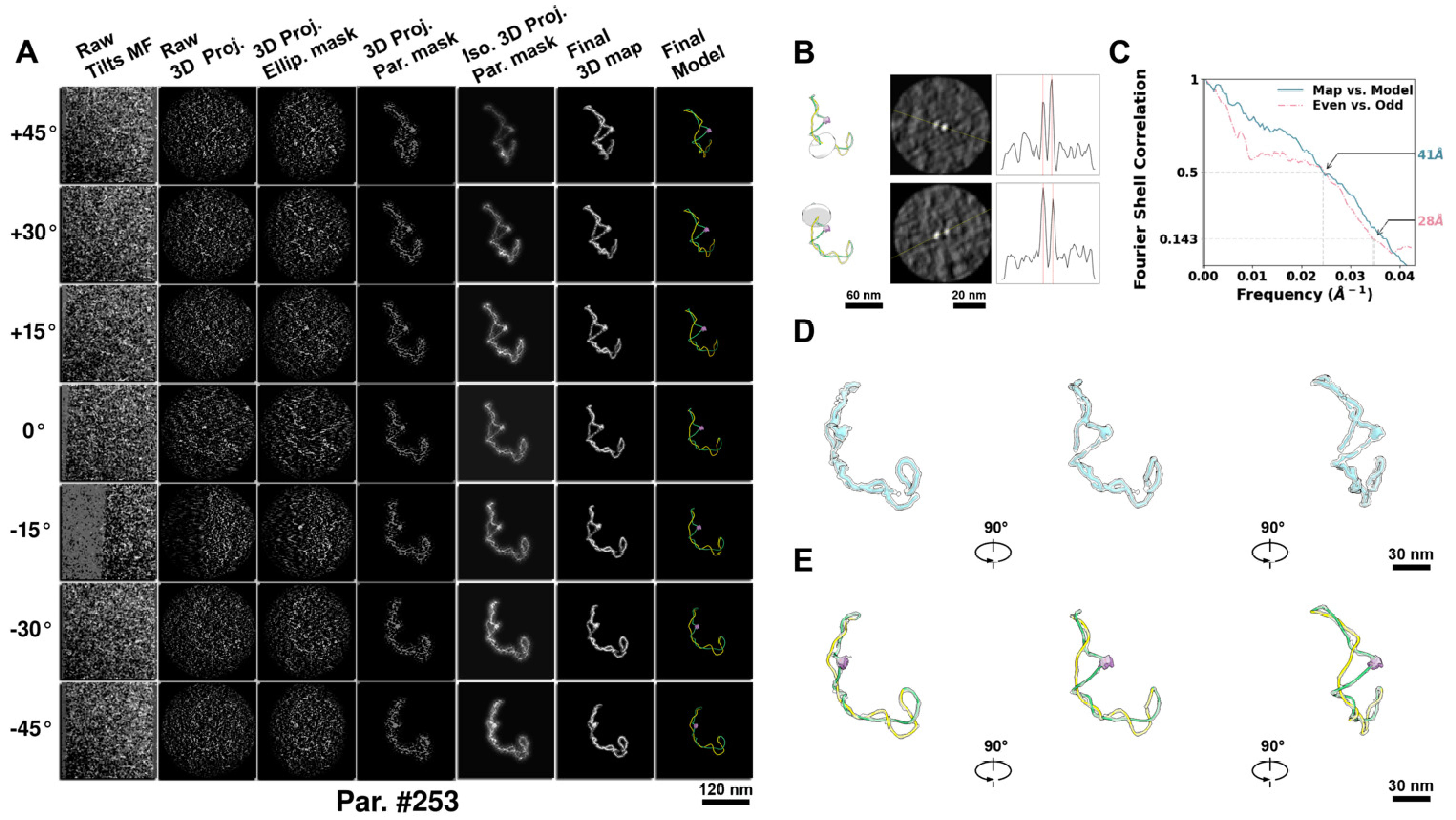

Supplementary Figure 253. Cryo-electron tomography (cryo-ET) 3D reconstruction of an individual plasmid particle in 40 mM K$^+$, 5 mM Mg^{2+} in the presence of transcribed RNAP and TopI.
(A) 3D reconstruction of the plasmid particle (index no. 253). The first column shows seven representative tilt images from –45° to +45° in step of 15°. The second, third, and fourth columns show 3D projections of the particle with spherical, ellipsoidal (thinner along the z-dimension), and particle-shaped masks, respectively. The fifth column displays the 3D projections of the enhanced and IsoNet missing-wedge-corrected particle. The sixth and seventh columns present the final 3D map and the flexibly fitted model, respectively. (B) Two random cross-sectional views (12 nm thickness) of the plasmid density map along its plectoneme axis are shown in the left-middle panel. The intensity profile along the line crossing the two high-density DNA spots is displayed in the right panel. (C) Resolution assessment of the final 3D map using Fourier shell correlation (FSC). Two criteria are shown: FSC between two half-maps reconstructed from even and odd frames (evaluated at 0.143) and FSC between the final 3D map and the fitted model (evaluated at 0.5). (D) Zoomed-in views of the final 3D density map from panel A, displayed at two contour levels. (E) Superimposition of the high-contour level map from panel D onto its flexibly fitted model.

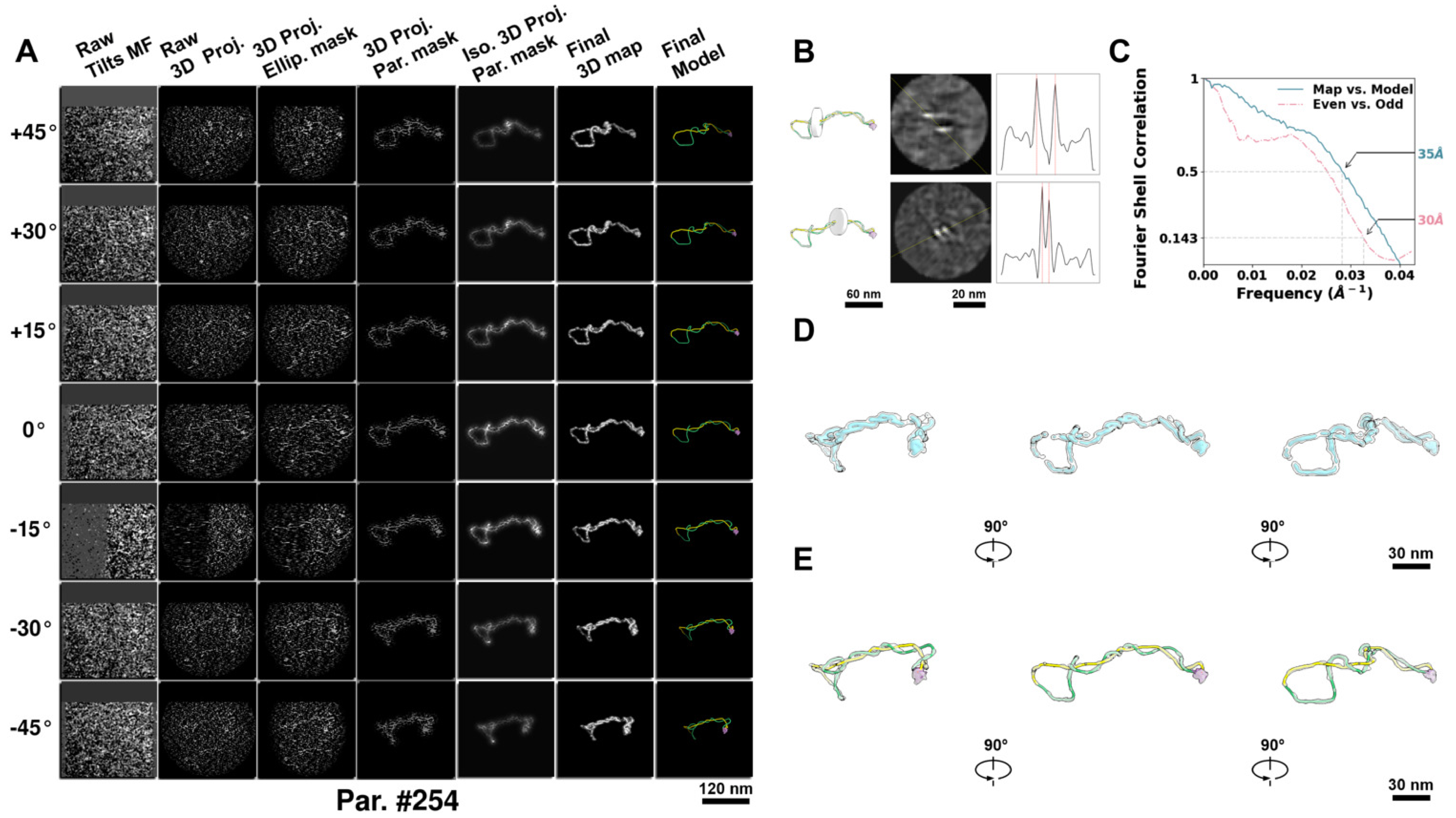

Supplementary Figure 254. Cryo-electron tomography (cryo-ET) 3D reconstruction of an individual plasmid particle in 40 mM K$^+$, 5 mM Mg^{2+} in the presence of transcribed RNAP and TopI.
(A) 3D reconstruction of the plasmid particle (index no. 254). The first column shows seven representative tilt images from –45° to +45° in step of 15°. The second, third, and fourth columns show 3D projections of the particle with spherical, ellipsoidal (thinner along the z-dimension), and particle-shaped masks, respectively. The fifth column displays the 3D projections of the enhanced and IsoNet missing-wedge-corrected particle. The sixth and seventh columns present the final 3D map and the flexibly fitted model, respectively. (B) Two random cross-sectional views (12 nm thickness) of the plasmid density map along its plectoneme axis are shown in the left-middle panel. The intensity profile along the line crossing the two high-density DNA spots is displayed in the right panel. (C) Resolution assessment of the final 3D map using Fourier shell correlation (FSC). Two criteria are shown: FSC between two half-maps reconstructed from even and odd frames (evaluated at 0.143) and FSC between the final 3D map and the fitted model (evaluated at 0.5). (D) Zoomed-in views of the final 3D density map from panel A, displayed at two contour levels. (E) Superimposition of the high-contour level map from panel D onto its flexibly fitted model.

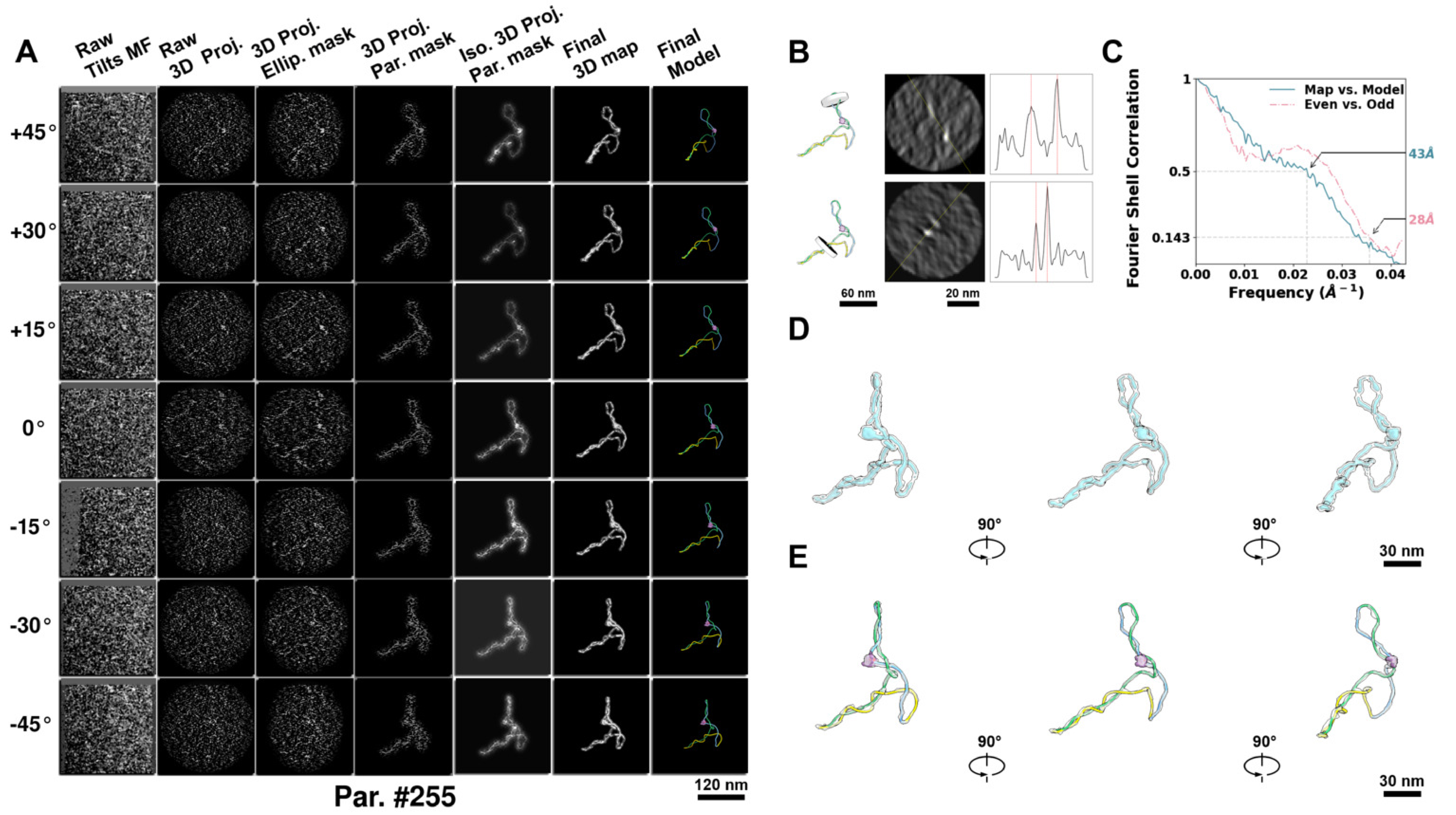

Supplementary Figure 255. Cryo-electron tomography (cryo-ET) 3D reconstruction of an individual plasmid particle in 40 mM K$^+$, 5 mM Mg^{2+} in the presence of transcribed RNAP and TopI.
(A) 3D reconstruction of the plasmid particle (index no. 255). The first column shows seven representative tilt images from –45° to +45° in step of 15°. The second, third, and fourth columns show 3D projections of the particle with spherical, ellipsoidal (thinner along the z-dimension), and particle-shaped masks, respectively. The fifth column displays the 3D projections of the enhanced and IsoNet missing-wedge-corrected particle. The sixth and seventh columns present the final 3D map and the flexibly fitted model, respectively. (B) Two random cross-sectional views (12 nm thickness) of the plasmid density map along its plectoneme axis are shown in the left-middle panel. The intensity profile along the line crossing the two high-density DNA spots is displayed in the right panel. (C) Resolution assessment of the final 3D map using Fourier shell correlation (FSC). Two criteria are shown: FSC between two half-maps reconstructed from even and odd frames (evaluated at 0.143) and FSC between the final 3D map and the fitted model (evaluated at 0.5). (D) Zoomed-in views of the final 3D density map from panel A, displayed at two contour levels. (E) Superimposition of the high-contour level map from panel D onto its flexibly fitted model.

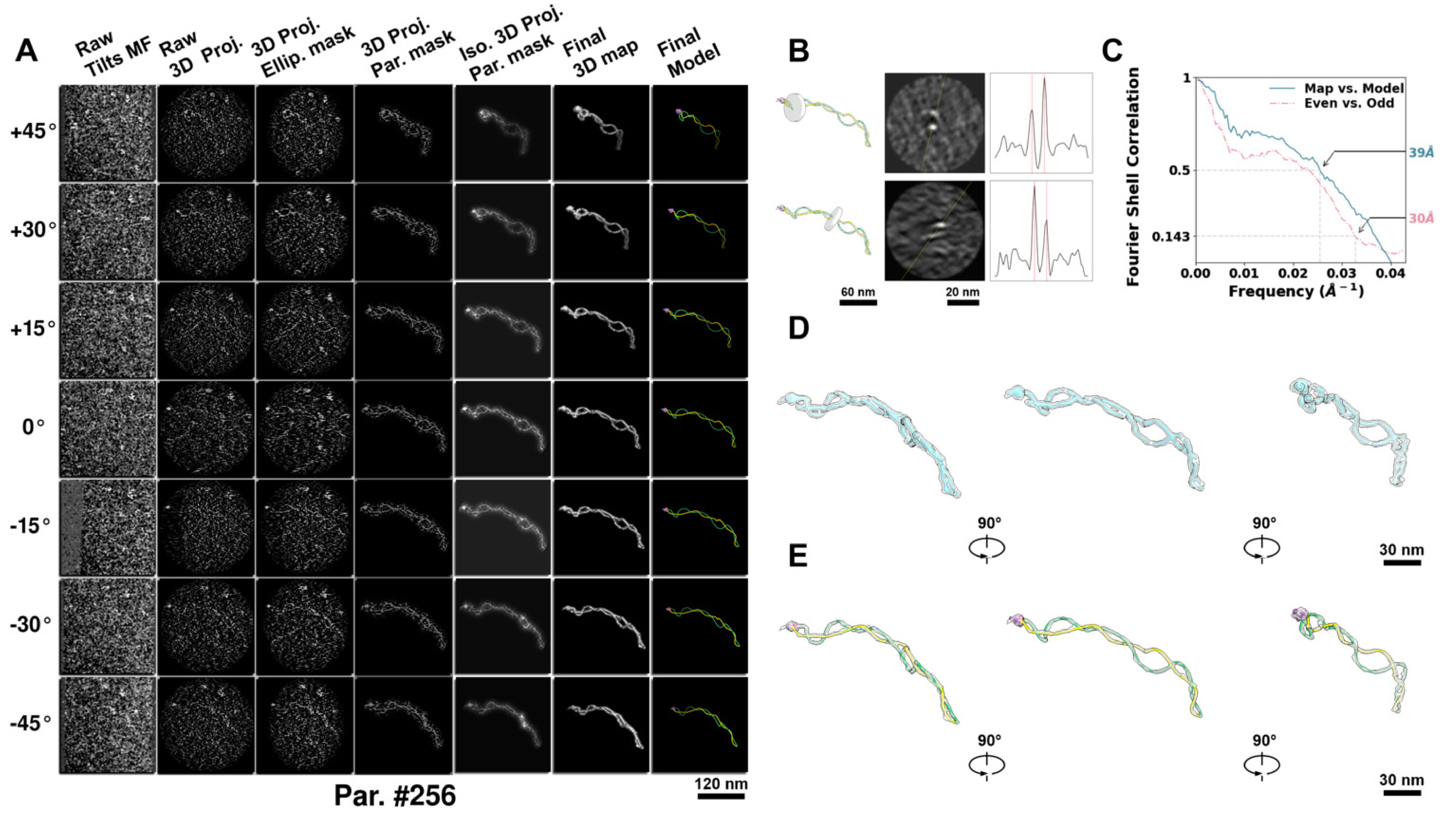

Supplementary Figure 256. Cryo-electron tomography (cryo-ET) 3D reconstruction of an individual plasmid particle in 40 mM K$^+$, 5 mM Mg^{2+} in the presence of transcribed RNAP and TopI.
(A) 3D reconstruction of the plasmid particle (index no. 256). The first column shows seven representative tilt images from –45° to +45° in step of 15°. The second, third, and fourth columns show 3D projections of the particle with spherical, ellipsoidal (thinner along the z-dimension), and particle-shaped masks, respectively. The fifth column displays the 3D projections of the enhanced and IsoNet missing-wedge-corrected particle. The sixth and seventh columns present the final 3D map and the flexibly fitted model, respectively. (B) Two random cross-sectional views (12 nm thickness) of the plasmid density map along its plectoneme axis are shown in the left-middle panel. The intensity profile along the line crossing the two high-density DNA spots is displayed in the right panel. (C) Resolution assessment of the final 3D map using Fourier shell correlation (FSC). Two criteria are shown: FSC between two half-maps reconstructed from even and odd frames (evaluated at 0.143) and FSC between the final 3D map and the fitted model (evaluated at 0.5). (D) Zoomed-in views of the final 3D density map from panel A, displayed at two contour levels. (E) Superimposition of the high-contour level map from panel D onto its flexibly fitted model.

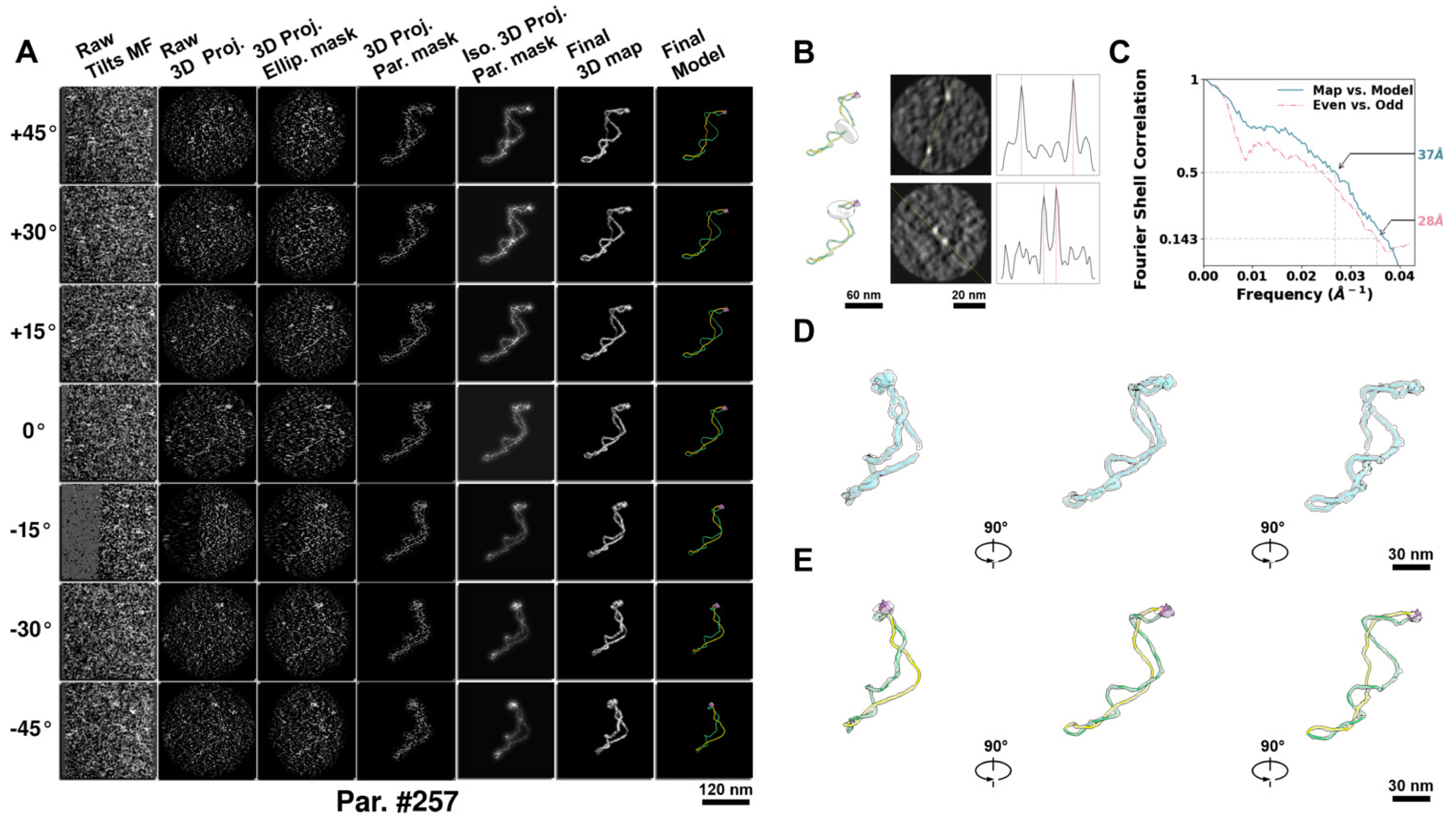

Supplementary Figure 257. Cryo-electron tomography (cryo-ET) 3D reconstruction of an individual plasmid particle in 40 mM K$^+$, 5 mM Mg^{2+} in the presence of transcribed RNAP and TopI.
(A) 3D reconstruction of the plasmid particle (index no. 257). The first column shows seven representative tilt images from –45° to +45° in step of 15°. The second, third, and fourth columns show 3D projections of the particle with spherical, ellipsoidal (thinner along the z-dimension), and particle-shaped masks, respectively. The fifth column displays the 3D projections of the enhanced and IsoNet missing-wedge-corrected particle. The sixth and seventh columns present the final 3D map and the flexibly fitted model, respectively. (B) Two random cross-sectional views (12 nm thickness) of the plasmid density map along its plectoneme axis are shown in the left-middle panel. The intensity profile along the line crossing the two high-density DNA spots is displayed in the right panel. (C) Resolution assessment of the final 3D map using Fourier shell correlation (FSC). Two criteria are shown: FSC between two half-maps reconstructed from even and odd frames (evaluated at 0.143) and FSC between the final 3D map and the fitted model (evaluated at 0.5). (D) Zoomed-in views of the final 3D density map from panel A, displayed at two contour levels. (E) Superimposition of the high-contour level map from panel D onto its flexibly fitted model.

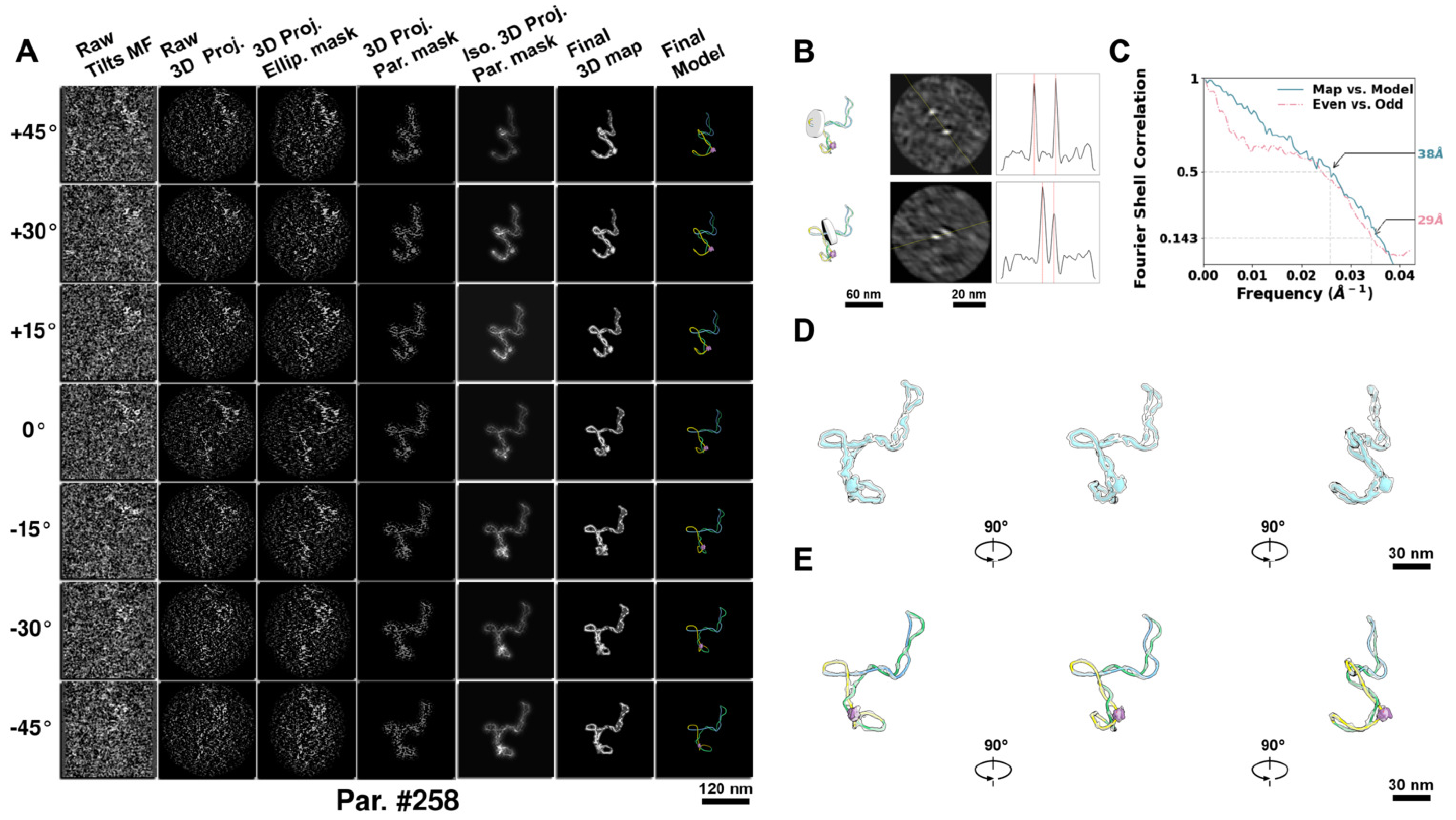

Supplementary Figure 258. Cryo-electron tomography (cryo-ET) 3D reconstruction of an individual plasmid particle in 40 mM K$^+$, 5 mM Mg^{2+} in the presence of transcribed RNAP and TopI.
(A) 3D reconstruction of the plasmid particle (index no. 258). The first column shows seven representative tilt images from –45° to +45° in step of 15°. The second, third, and fourth columns show 3D projections of the particle with spherical, ellipsoidal (thinner along the z-dimension), and particle-shaped masks, respectively. The fifth column displays the 3D projections of the enhanced and IsoNet missing-wedge-corrected particle. The sixth and seventh columns present the final 3D map and the flexibly fitted model, respectively. (B) Two random cross-sectional views (12 nm thickness) of the plasmid density map along its plectoneme axis are shown in the left-middle panel. The intensity profile along the line crossing the two high-density DNA spots is displayed in the right panel. (C) Resolution assessment of the final 3D map using Fourier shell correlation (FSC). Two criteria are shown: FSC between two half-maps reconstructed from even and odd frames (evaluated at 0.143) and FSC between the final 3D map and the fitted model (evaluated at 0.5). (D) Zoomed-in views of the final 3D density map from panel A, displayed at two contour levels. (E) Superimposition of the high-contour level map from panel D onto its flexibly fitted model.

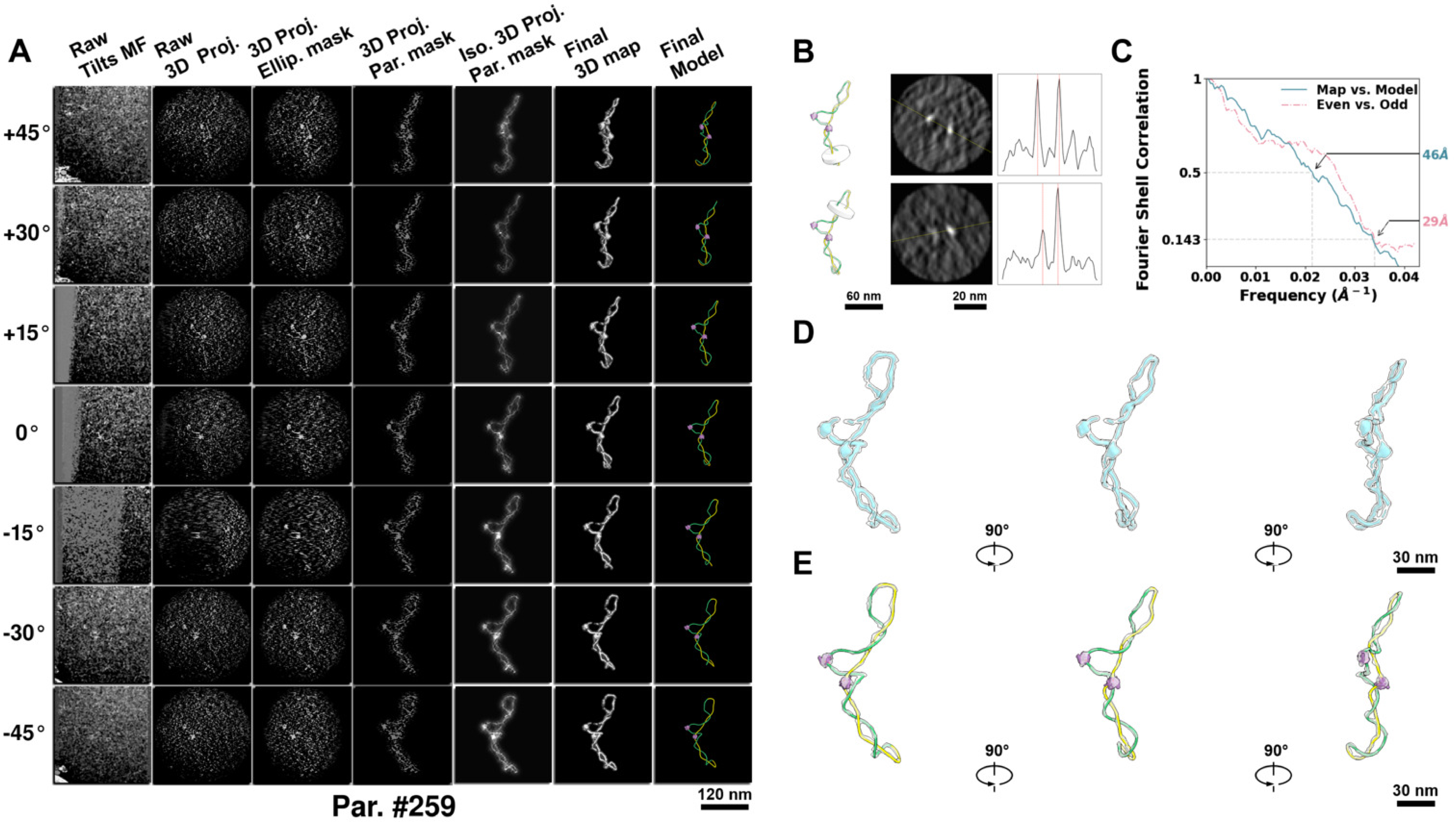

Supplementary Figure 259. Cryo-electron tomography (cryo-ET) 3D reconstruction of an individual plasmid particle in 40 mM K⁺, 5 mM Mg^{2+} in the presence of transcribed RNAP and TopI.
(A) 3D reconstruction of the plasmid particle (index no. 259). The first column shows seven representative tilt images from –45° to +45° in step of 15°. The second, third, and fourth columns show 3D projections of the particle with spherical, ellipsoidal (thinner along the z-dimension), and particle-shaped masks, respectively. The fifth column displays the 3D projections of the enhanced and IsoNet missing-wedge-corrected particle. The sixth and seventh columns present the final 3D map and the flexibly fitted model, respectively. (B) Two random cross-sectional views (12 nm thickness) of the plasmid density map along its plectoneme axis are shown in the left-middle panel. The intensity profile along the line crossing the two high-density DNA spots is displayed in the right panel. (C) Resolution assessment of the final 3D map using Fourier shell correlation (FSC). Two criteria are shown: FSC between two half-maps reconstructed from even and odd frames (evaluated at 0.143) and FSC between the final 3D map and the fitted model (evaluated at 0.5). (D) Zoomed-in views of the final 3D density map from panel A, displayed at two contour levels. (E) Superimposition of the high-contour level map from panel D onto its flexibly fitted model.

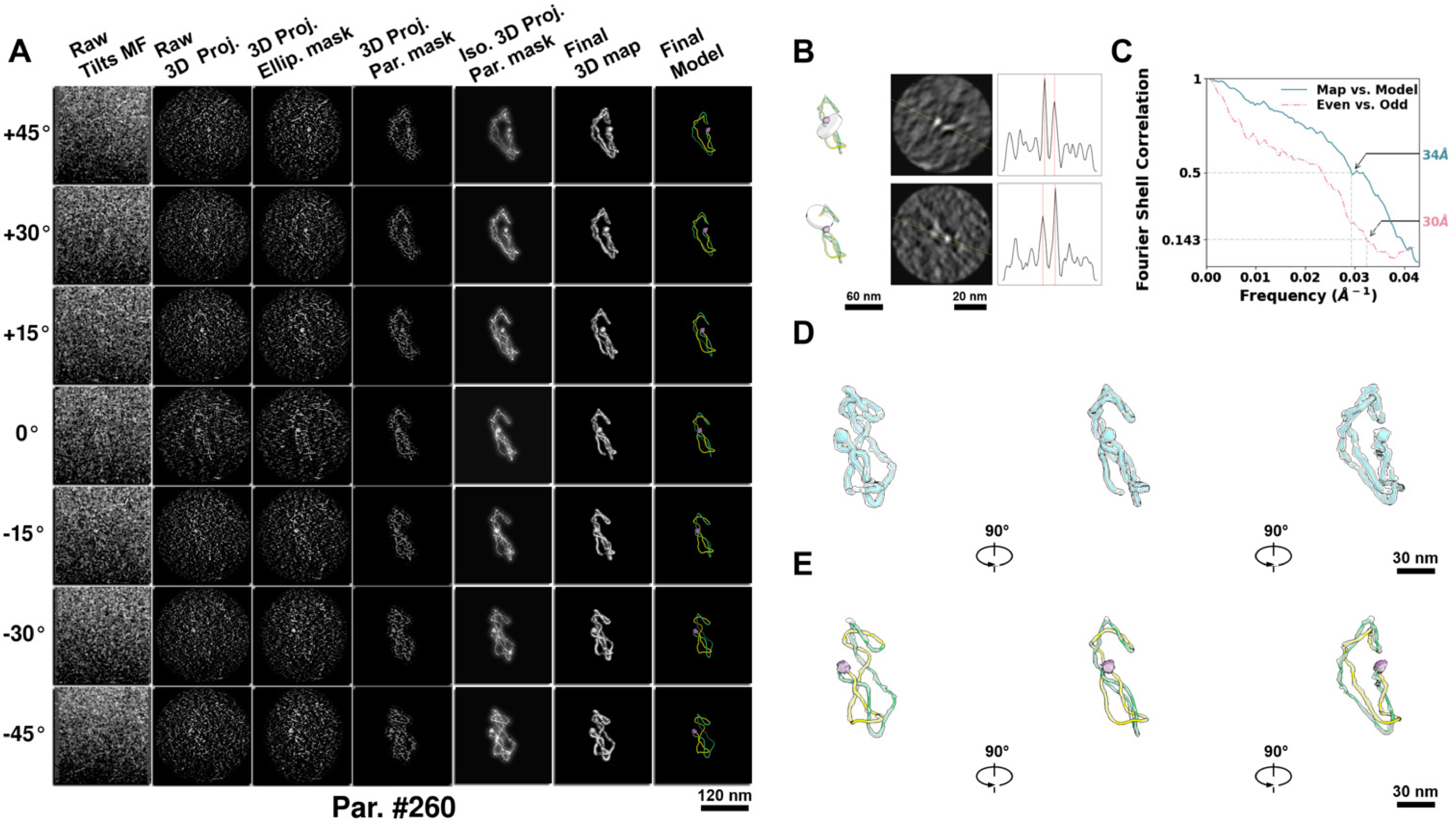

Supplementary Figure 260. Cryo-electron tomography (cryo-ET) 3D reconstruction of an individual plasmid particle in 40 mM K⁺, 5 mM Mg^{2+} in the presence of transcribed RNAP and TopI.
(A) 3D reconstruction of the plasmid particle (index no. 260). The first column shows seven representative tilt images from –45° to +45° in step of 15°. The second, third, and fourth columns show 3D projections of the particle with spherical, ellipsoidal (thinner along the z-dimension), and particle-shaped masks, respectively. The fifth column displays the 3D projections of the enhanced and IsoNet missing-wedge-corrected particle. The sixth and seventh columns present the final 3D map and the flexibly fitted model, respectively. (B) Two random cross-sectional views (12 nm thickness) of the plasmid density map along its plectoneme axis are shown in the left-middle panel. The intensity profile along the line crossing the two high-density DNA spots is displayed in the right panel. (C) Resolution assessment of the final 3D map using Fourier shell correlation (FSC). Two criteria are shown: FSC between two half-maps reconstructed from even and odd frames (evaluated at 0.143) and FSC between the final 3D map and the fitted model (evaluated at 0.5). (D) Zoomed-in views of the final 3D density map from panel A, displayed at two contour levels. (E) Superimposition of the high-contour level map from panel D onto its flexibly fitted model.

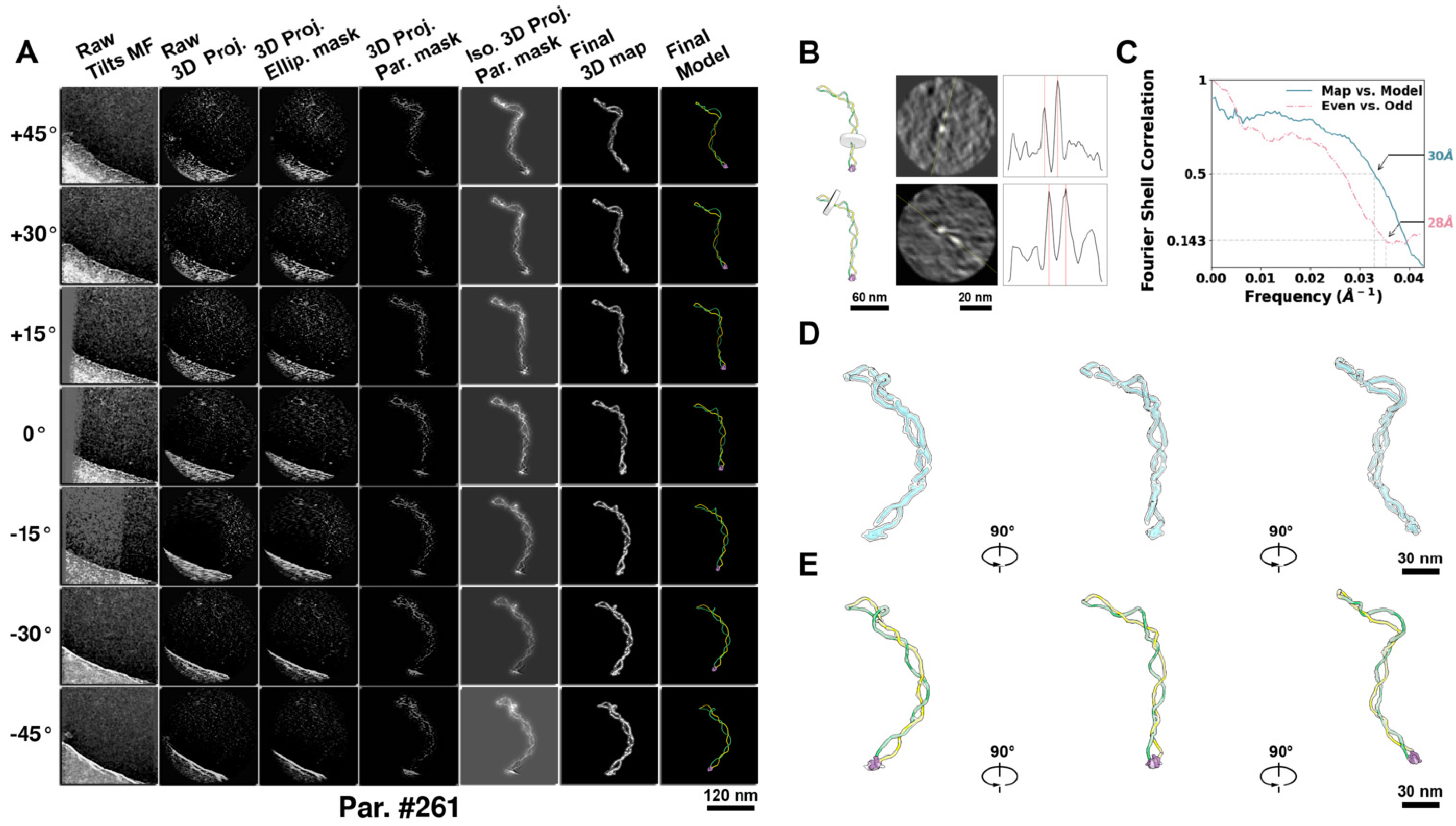

Supplementary Figure 261. Cryo-electron tomography (cryo-ET) 3D reconstruction of an individual plasmid particle in 40 mM K$^+$, 5 mM Mg^{2+} in the presence of transcribed RNAP and TopI.
(A) 3D reconstruction of the plasmid particle (index no. 261). The first column shows seven representative tilt images from –45° to +45° in step of 15°. The second, third, and fourth columns show 3D projections of the particle with spherical, ellipsoidal (thinner along the z-dimension), and particle-shaped masks, respectively. The fifth column displays the 3D projections of the enhanced and IsoNet missing-wedge-corrected particle. The sixth and seventh columns present the final 3D map and the flexibly fitted model, respectively. (B) Two random cross-sectional views (12 nm thickness) of the plasmid density map along its plectoneme axis are shown in the left-middle panel. The intensity profile along the line crossing the two high-density DNA spots is displayed in the right panel. (C) Resolution assessment of the final 3D map using Fourier shell correlation (FSC). Two criteria are shown: FSC between two half-maps reconstructed from even and odd frames (evaluated at 0.143) and FSC between the final 3D map and the fitted model (evaluated at 0.5). (D) Zoomed-in views of the final 3D density map from panel A, displayed at two contour levels. (E) Superimposition of the high-contour level map from panel D onto its flexibly fitted model.

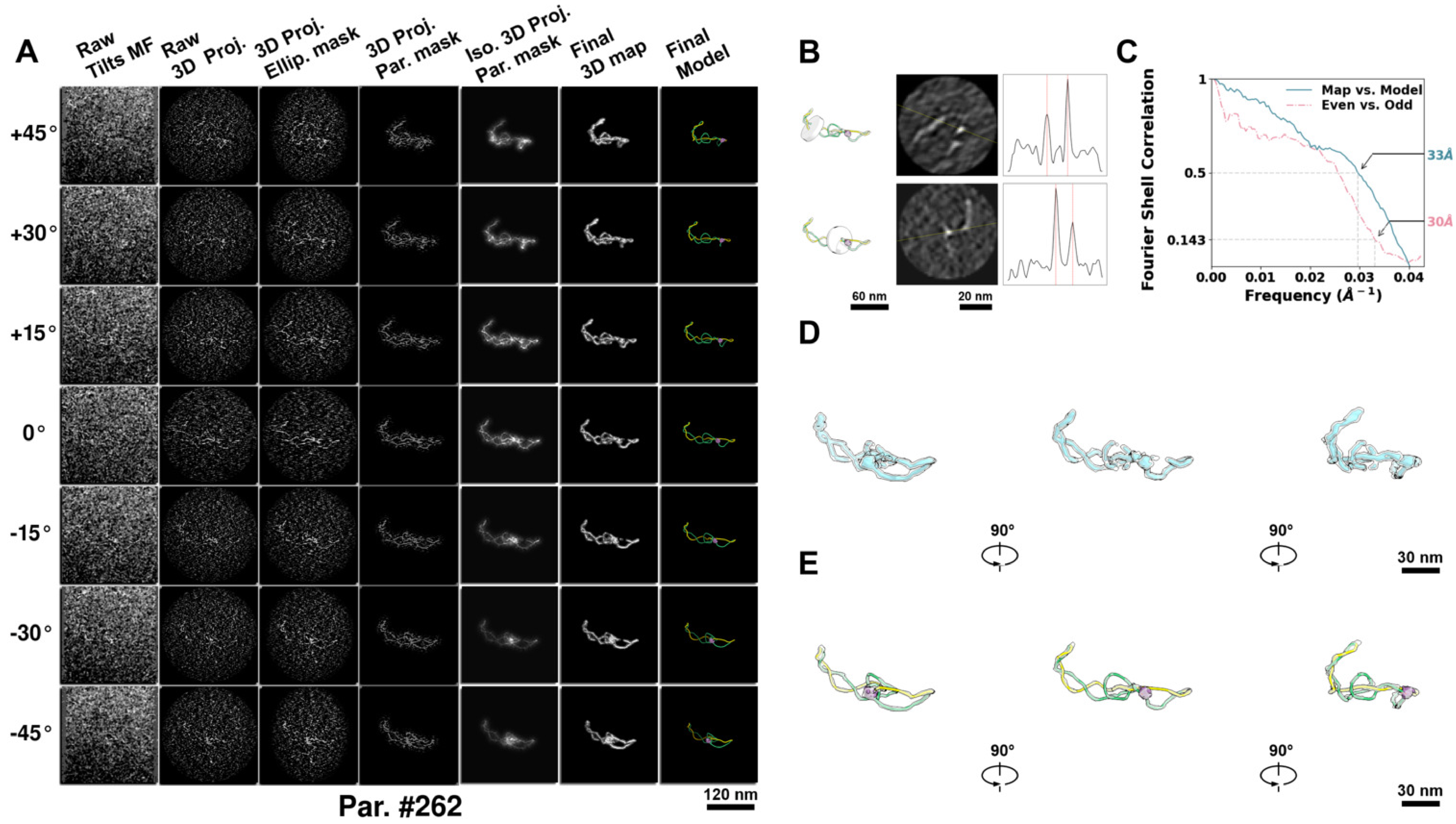

Supplementary Figure 262. Cryo-electron tomography (cryo-ET) 3D reconstruction of an individual plasmid particle in 40 mM K$^+$, 5 mM Mg^{2+} in the presence of transcribed RNAP and TopI.
(A) 3D reconstruction of the plasmid particle (index no. 262). The first column shows seven representative tilt images from –45° to +45° in step of 15°. The second, third, and fourth columns show 3D projections of the particle with spherical, ellipsoidal (thinner along the z-dimension), and particle-shaped masks, respectively. The fifth column displays the 3D projections of the enhanced and IsoNet missing-wedge-corrected particle. The sixth and seventh columns present the final 3D map and the flexibly fitted model, respectively. (B) Two random cross-sectional views (12 nm thickness) of the plasmid density map along its plectoneme axis are shown in the left-middle panel. The intensity profile along the line crossing the two high-density DNA spots is displayed in the right panel. (C) Resolution assessment of the final 3D map using Fourier shell correlation (FSC). Two criteria are shown: FSC between two half-maps reconstructed from even and odd frames (evaluated at 0.143) and FSC between the final 3D map and the fitted model (evaluated at 0.5). (D) Zoomed-in views of the final 3D density map from panel A, displayed at two contour levels. (E) Superimposition of the high-contour level map from panel D onto its flexibly fitted model.

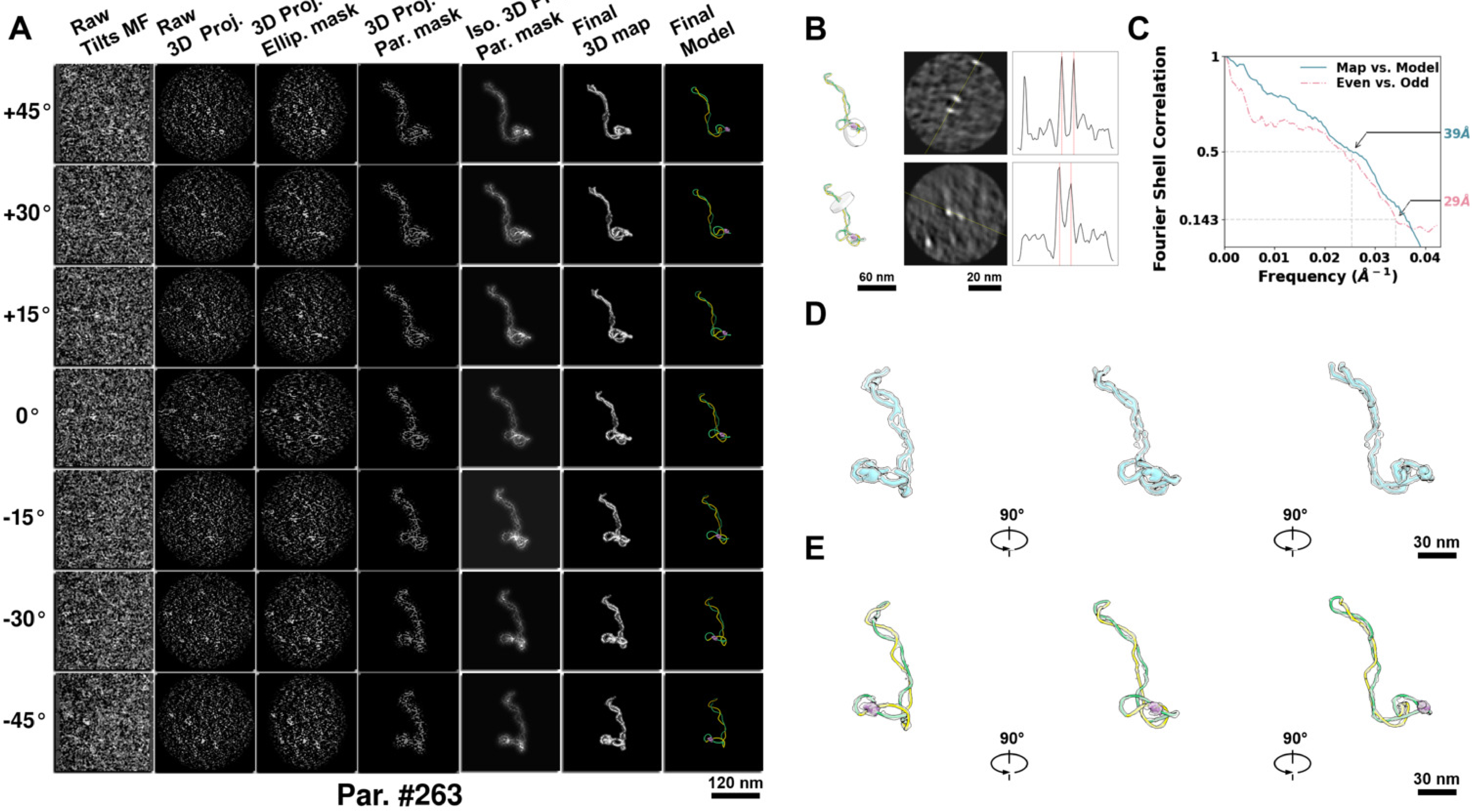

Supplementary Figure 263. Cryo-electron tomography (cryo-ET) 3D reconstruction of an individual plasmid particle in 40 mM K⁺, 5 mM Mg^{2+} in the presence of transcribed RNAP and TopI.
(A) 3D reconstruction of the plasmid particle (index no. 263). The first column shows seven representative tilt images from –45° to +45° in step of 15°. The second, third, and fourth columns show 3D projections of the particle with spherical, ellipsoidal (thinner along the z-dimension), and particle-shaped masks, respectively. The fifth column displays the 3D projections of the enhanced and IsoNet missing-wedge-corrected particle. The sixth and seventh columns present the final 3D map and the flexibly fitted model, respectively. (B) Two random cross-sectional views (12 nm thickness) of the plasmid density map along its plectoneme axis are shown in the left-middle panel. The intensity profile along the line crossing the two high-density DNA spots is displayed in the right panel. (C) Resolution assessment of the final 3D map using Fourier shell correlation (FSC). Two criteria are shown: FSC between two half-maps reconstructed from even and odd frames (evaluated at 0.143) and FSC between the final 3D map and the fitted model (evaluated at 0.5). (D) Zoomed-in views of the final 3D density map from panel A, displayed at two contour levels. (E) Superimposition of the high-contour level map from panel D onto its flexibly fitted model.

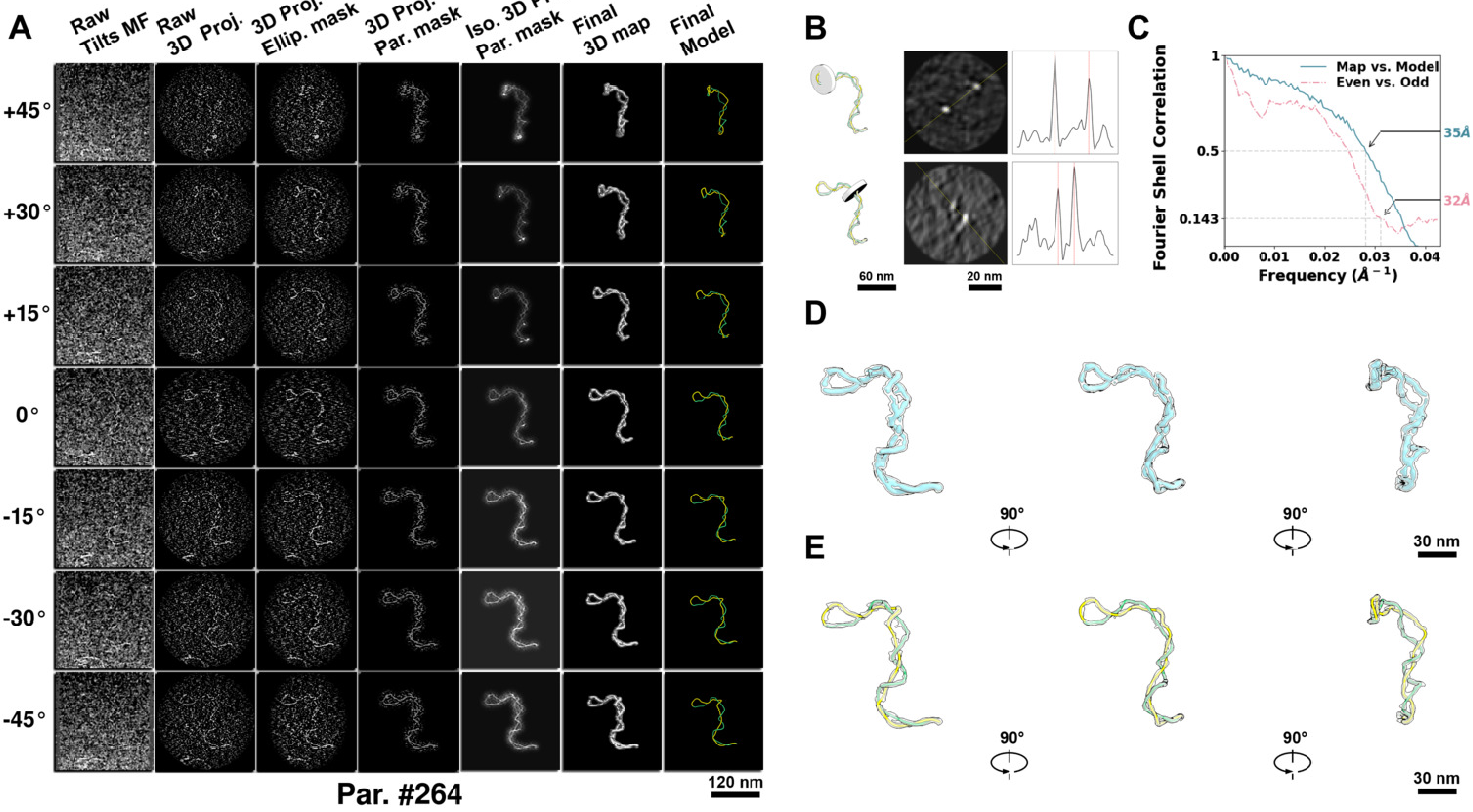

Supplementary Figure 264. Cryo-electron tomography (cryo-ET) 3D reconstruction of an individual plasmid particle in 40 mM K⁺, 5 mM Mg^{2+} in the presence of transcribed RNAP and TopI.
(A) 3D reconstruction of the plasmid particle (index no. 264). The first column shows seven representative tilt images from –45° to +45° in step of 15°. The second, third, and fourth columns show 3D projections of the particle with spherical, ellipsoidal (thinner along the z-dimension), and particle-shaped masks, respectively. The fifth column displays the 3D projections of the enhanced and IsoNet missing-wedge-corrected particle. The sixth and seventh columns present the final 3D map and the flexibly fitted model, respectively. (B) Two random cross-sectional views (12 nm thickness) of the plasmid density map along its plectoneme axis are shown in the left-middle panel. The intensity profile along the line crossing the two high-density DNA spots is displayed in the right panel. (C) Resolution assessment of the final 3D map using Fourier shell correlation (FSC). Two criteria are shown: FSC between two half-maps reconstructed from even and odd frames (evaluated at 0.143) and FSC between the final 3D map and the fitted model (evaluated at 0.5). (D) Zoomed-in views of the final 3D density map from panel A, displayed at two contour levels. (E) Superimposition of the high-contour level map from panel D onto its flexibly fitted model.
